# Supplementary material for: Paired electrolysis-enabled nickel-catalyzed enantioselective reductive cross-coupling between α-chloroesters and aryl bromides
Source: Nat Commun. 2022 Nov 28;13:7318. doi: 10.1038/s41467-022-35073-z (PMC9705544; doi:10.1038/s41467-022-35073-z)
Supplement: Supplementary file 1 — Supplementary Information [file 41467_2022_35073_MOESM1_ESM.pdf]

Paired Electrolysis-Enabled Nickel-Catalyzed Enantioselective Reductive Cross-Coupling Between α-Chloroesters and Aryl Bromides

（Supplementary Information）

Dong Liu[1, ‡], Zhao-Ran Liu[1, ‡], Zhen-Hua Wang[1, ‡], Cong Ma[1], Simon Herbert[2], Hartmut Schirok[2], Tian-Sheng Mei[1]*

[1]State Key Laboratory of Organometallic Chemistry, Center for Excellence in Molecular Synthesis, Shanghai Institute of Organic Chemistry, University of Chinese Academy of Sciences, Chinese Academy of Science, 345 Lingling Lu, Shanghai 200032, China.

[2] Pharmaceuticals, Research and Development, Bayer AG, Berlin 13353, Germany.

Table of Contents

1. Supplementary Notes 2

2. Supplementary Discussion 3

2.1 Optimization of the Reaction Conditions.....................................3

2.2 Preparation of Substrate 10

2.3. Suboptimal and Unsuccessful Substrates..................................18

2.4 General Procedure for Electrochemical C-C Coupling 19

2.5 Preparation and X-ray Diffraction of Nickel Pre-Catalyst 23

2.6 Cyclic Voltammetry...27

2.7 Characterization Data for the Products 31

2.8 Radical Probe Experiments and Structral Confirmation of Cyclized Products 70

2.9 EPR Experiments and Data 72

3. Supplementary Figures 74

3.1 X-Ray Structures and Data..74

3.2 NMR Spectra and Chromatograms..78

3.3 HPLC Spectra Data 160

4. Supplementary References 226

1. Supplementary Notes

Commercially available materials were used without further purification. Column chromatography was performed using either 100-200 Mesh or 300-400 Mesh silica gel. Visualization of spots on the TLC plate was accomplished with UV light (254 nm) and staining over the I_2 chamber.

All commercial reagents were purchased from TCI, Sigma-Aldrich, Adamas-beta, J&K, and Energy Chemical of the highest purity grade. They were used without further purification unless specified. Tetrabutylammonium bromide (TBAB, 99.0%) was purchased from TCI Chemical and was used without further purification. Nickel(II) bromide ethylene glycol dimethyl ether (≥97.0%) was purchased from Sigma-Aldrich and was used as received. 2,6-Lutidine, Pyridine, DBU (1,8-Diazabicyclo[5.4.0]undec-7-ene), DBN (1,5-Diazabicyclo[4.3.0]non-5-ene), DABCO (bicyclo(2.2.2)-1,4-diazaoctane), DIPEA (*N, N*-diisopropylethylamine), DMEDA (*N, N*-dimethyl ethylene diamines), TTMSS (tri(trimethylsilyl)silane) was purchased from Alfa Aesar and was used without further purification; DMAc (99.8%, SuperDry) was purchased from Acros and was used without further purification. ^{1}H NMR and ^{13}C NMR spectra were recorded on Agilent AV 400, Varian Inova 400 (400 MHz and 100 MHz, respectively). ^{19}F NMR spectra were recorded on Agilent AV 400, Varian Inova 400 (376 MHz) instrument and were reported relative to the $CFCl_3$ as the internal standard. The peaks were internally referenced to TMS (0.00 ppm) or residual undeuterated solvent signal. The following abbreviations were used to explain multiplicities: s = singlet, d = doublet, t = triplet, q = quartet, m = multiplet, and br = broad. Infrared spectra were obtained on a Bio-Rad FTS-185 instrument. High-resolution mass spectra were recorded at the Center for Mass Spectrometry, Shanghai Institute of Organic Chemistry. Analytical and spectral data of all those known compounds were exactly matching with the reported values.

2. Supplementary Discussion

2.1 Optimization of the Reaction Conditions

Supplementary Table 1: Optimization of the racemic reaction conditions.

Screening of electrodes:

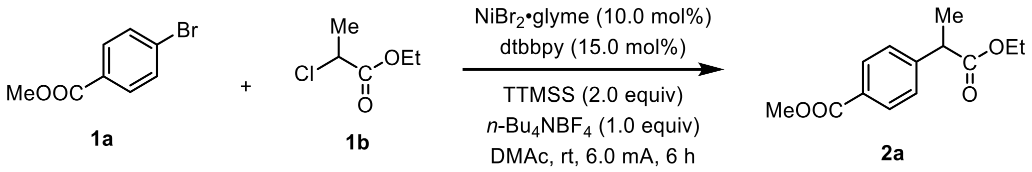

Entry	Electrode (Anode-Cathode)	Yield (%)[a]
1	RVC (1.5 x 1.5 cm)-Ni foam (2.0 x 3.0 cm)	15
2	Fe-Ni foam (2.0 x 3.0 cm)	NR
3	Al-Ni foam (2.0 x 3.0 cm)	NR
4	Mg-Ni foam (2.0 x 3.0 cm)	NR
5	C (1.5 x 1.5 cm)-Ni foam (2.0 x 3.0 cm)	8
6	Pt (1.0 x 1.0 cm)- Ni foam (2.0 x 3.0 cm)	20
7	RVC (1.5 x 1.5 cm)-Pt (1.0 x 1.0 cm)	trace

Optimization of the reaction conditions. [a]Reaction conditions: **1a** (0.2 mmol), **1b** (2.0 equiv), $NiBr_2$•glyme (10.0 mol%), dtbbpy (15.0 mol%), Tris(trimethylsilyl)silane (2.0 equiv), *n*-Bu_4NBF_4 (1.0 equiv), and DMAc (2.0 mL) at rt, in an undivided cell subject to 6.0 mA of current for 6.0 h using different electrodes, argon. Yield was determined by ^1H NMR using CH_2Br_2 as an internal standard.

Supplementary Table 2: Optimization of the racemic reaction conditions.

Screening of bases:

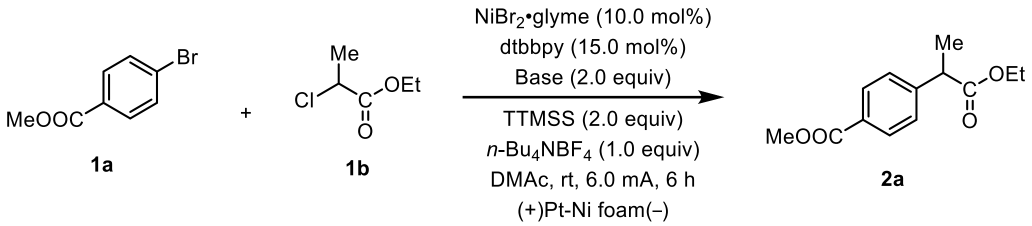

Entry	Base	Yield (%)[a]
1	DBU	NR
2	DBN	NR
3	Pyridine	<5
4	2,6-lutidine	91
5	LiOH	<5
6	Na_2CO_3	14
7	TMG	<5

Optimization of the reaction conditions. [a]Reaction conditions: **1a** (0.2 mmol), **1b** (2.0 equiv), $NiBr_2$•glyme (10.0 mol%), dtbbpy (15.0 mol%), Base (2.0 equiv), Tris(trimethylsilyl)silane (2.0 equiv), *n*-Bu_4NBF_4 (1.0 equiv), and DMAc (2.0 mL) at rt, in an undivided cell subject to 6.0 mA of current for 6.0 h using platinum electrodes (1.0×1.0 cm^2) and Ni foam (2.0×3.0 cm^2) electrodes, argon. Yield was determined by ^1H NMR using CH_2Br_2 as an internal standard.

Supplementary Table 3: Procedure for chiral ligand evaluation[a]

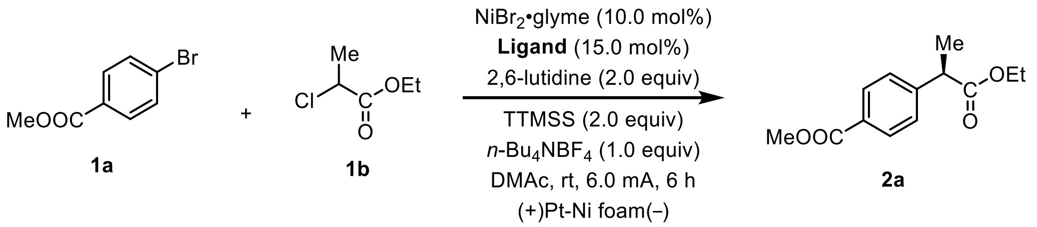

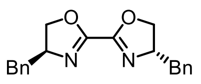

L1
10% yield, 44% ee

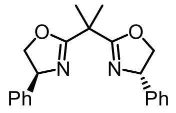

L9
< 5% yield, --

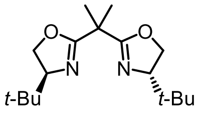

L10
10% yield, <5% ee

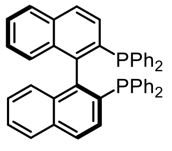

L11
< 5% yield, --

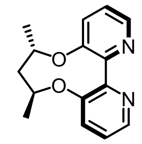

L12
20% yield, 20% ee

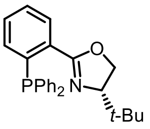

L13
< 5% yield, --

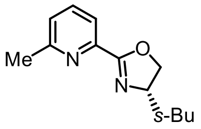

L14
< 5% yield, 13% ee

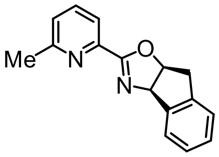

L15
10% yield, 15% ee

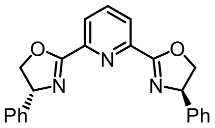

L16, < 5%, --

Optimization of the reaction conditions. [a]Reaction conditions: **1a** (0.2 mmol), (*rac*)-**1b** (2.0 equiv), $NiBr_2$•glyme (10.0 mol%), **Ligand** (15.0 mol%), 2,6-lutidine (2.0 equiv), Tris(trimethylsilyl)silane (2.0 equiv), *n*-Bu_4NBF_4 (1.0 equiv), and DMAc (2.0 mL) at rt, in an undivided cell subject to 6.0 mA of current for 6.0 h using platinum electrodes (1.0×1.0 cm^2) and Ni foam (2.0×3.0 cm^2) electrodes, argon. Yield was determined by ^1H NMR using CH_2Br_2 as an internal standard. Enantioselectivities were determined by chiral HPLC analysis.

Supplementary Table 4: Procedure for chiral Biox-ligand evaluation[a]

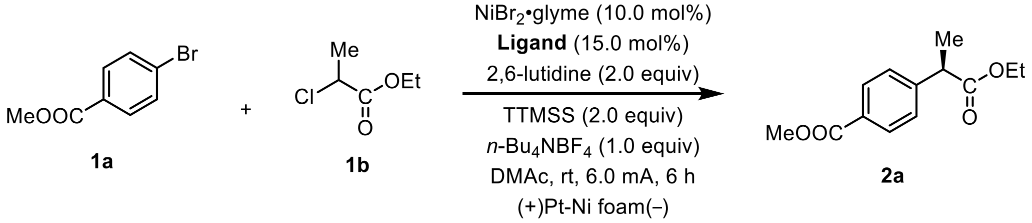

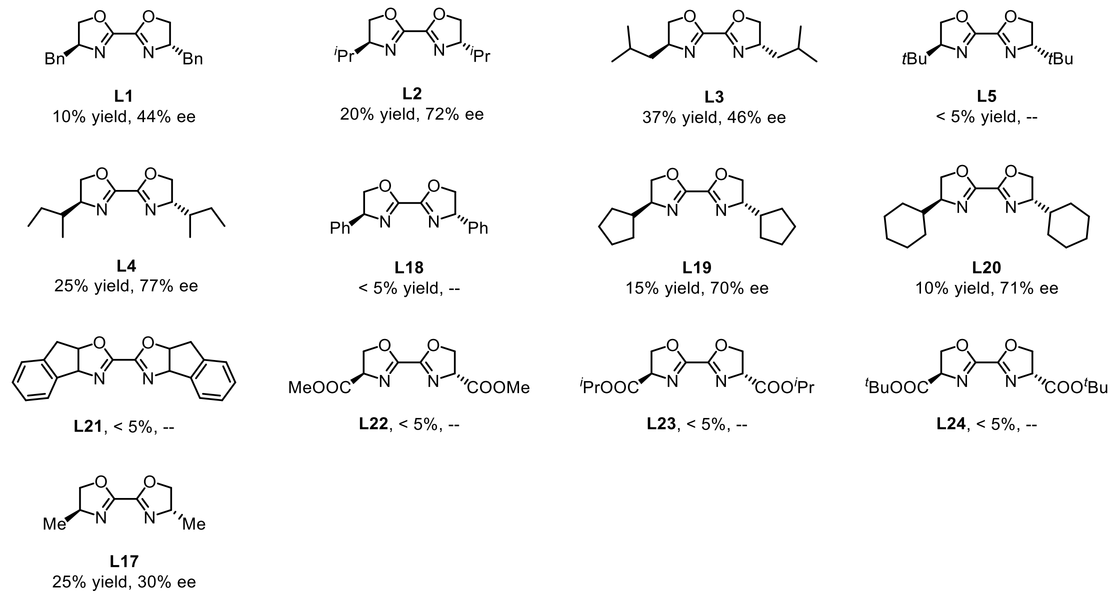

Optimization of the reaction conditions. [a]Reaction conditions: **1a** (0.2 mmol), (*rac*)-**1b** (2.0 equiv), $NiBr_2$•glyme (10.0 mol%), **Ligand** (15.0 mol%), 2,6-lutidine (2.0 equiv), Tris(trimethylsilyl)silane (2.0 equiv), *n*-Bu_4NBF_4 (1.0 equiv), and DMAc (2.0 mL) at rt, in an undivided cell subject to 6.0 mA of current for 6.0 h using platinum electrodes (1.0×1.0 cm^2) and Ni foam (2.0×3.0 cm^2) electrodes, argon. Yield was determined by ^1H NMR using CH_2Br_2 as an internal standard. Enantioselectivities were determined by chiral HPLC analysis.

Supplementary Table 5: Procedure for α-chloro ester evaluation[a]

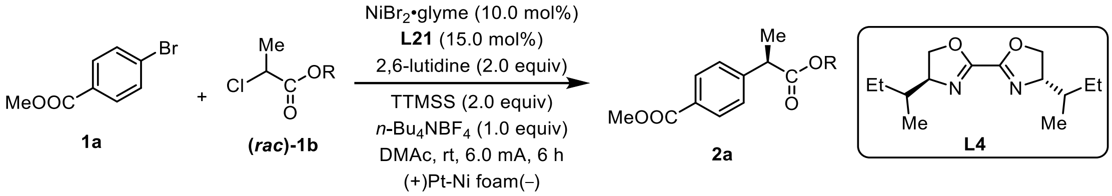

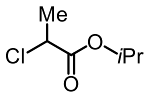

1b-2
15% yield, 76% ee

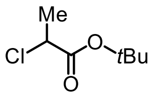

1b-3
15% yield, 77% ee

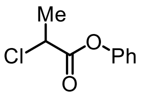

1b-4
< 5%, --

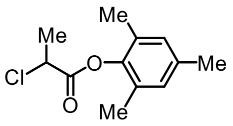

1b-5
< 5%, --

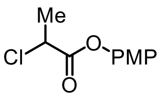

1b-6
20% yield, 67% ee

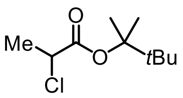

1b-7
10% yield, 84% ee

Optimization of the reaction conditions. [a]Reaction conditions: **1a** (0.2 mmol), (rac)-**1b** (2.0 equiv), $NiBr_2$•glyme (10.0 mol%), **L21** (15.0 mol%), 2,6-lutidine (2.0 equiv), Tris(trimethylsilyl)silane (2.0 equiv), *n*-Bu_4NBF_4 (1.0 equiv), and DMAc (2.0 mL) at rt, in an undivided cell subject to 6.0 mA of current for 6.0 h using platinum electrodes (1.0×1.0 cm^2) and Ni foam (2.0×3.0 cm^2) electrodes, argon. Yield was determined by ^{1}H NMR using CH_2Br_2 as an internal standard. Enantioselectivities were determined by chiral HPLC analysis.

Supplementary Table 6: Optimization of the reaction conditions.

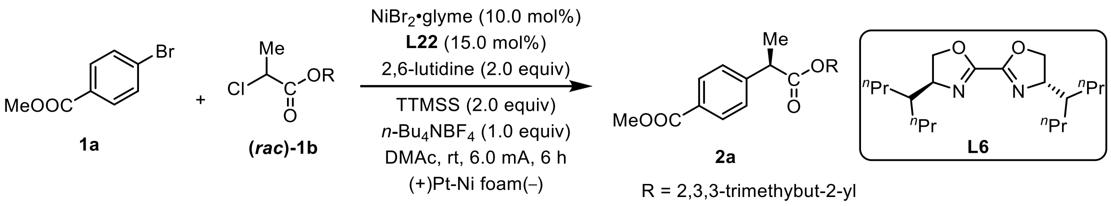

entry	deviation from above conditions	yield (%)[a]
1	none	11 (89% ee)
2	U_{cell} 2.4 V in lieu of 6.0 mA	22.5
3	U_{cell} 2.5 V in lieu of 6.0 mA	31.5
4	U_{cell} 2.6 V in lieu of 6.0 mA	42
5	U_{cell} 2.7 V in lieu of 6.0 mA	40
6	U_{cell} 2.8 V in lieu of 6.0 mA	39
7	U_{cell} 2.9 V in lieu of 6.0 mA	33
8	U_{cell} 3.0 V in lieu of 6.0 mA	33
9	1a:1b = 2:1 in lieu of 1a:1b = 1:2	20
10	1a:1b = 1:3 in lieu of 1a:1b = 1:2	30
11	8.0 mA in lieu of 6.0 mA	28

Optimization of the reaction conditions. [a]Reaction conditions: **1a** (0.2 mmol, 1.0 equiv), (*rac*)-**1b**, $NiBr_2$•glyme (10.0 mol%), **L22** (15.0 mol%), 2,6-lutidine (2.0 equiv), Tris(trimethylsilyl)silane (2.0 equiv), *n*-Bu_4NBF_4 (1.0 equiv), and DMAc (2.0 mL) at rt, in an undivided cell for 6.0 h using platinum electrodes (1.0×1.0 cm^2) and Ni foam (2.0×3.0 cm^2) electrodes, argon. Yield was determined by ^{1}H NMR using CH_2Br_2 as an internal standard. Enantioselectivities were determined by chiral HPLC analysis.

Supplementary Table 7: Optimization of the reaction conditions.

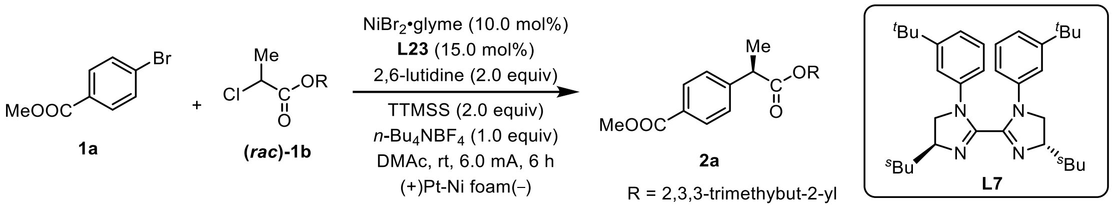

entry	deviation from above conditions	yield (%)[a]
1	none	30 (90% ee)
2	U_{cell} 2.8 V in lieu of 6.0 mA	30
3	U_{cell} 2.9 V in lieu of 6.0 mA	33
4	U_{cell} 3.0 V in lieu of 6.0 mA	33
	---------------------------- U_{cell} = 2.9 V ----------------------------	
5	3.0 equiv TTMSS in lieu of 2.0 equiv TTMSS	60
6	3.5 equiv TTMSS in lieu of 2.0 equiv TTMSS	69 (90% ee)
7	4.0 equiv TTMSS in lieu of 2.0 equiv TTMSS	61
	--------------------- U_{cell} = 2.9 V, 3.5 equiv TTMSS ----------------------	
8	2.5 equiv Lutidine in lieu of 2.5 equiv Lutidine	66 (90% ee)
9	3.0 equiv Lutidine in lieu of 2.5 equiv Lutidine	66 (91% ee)
10	3.5 equiv Lutidine in lieu of 2.5 equiv Lutidine	56
11	$Ni(cod)_2$ in stead of $NiBr_2$•glyme	46 (91% ee)

Optimization of the reaction conditions. [a]Reaction conditions: **1a** (0.2 mmol, 1.0 equiv), (*rac*)-**1b** (2.0 equiv), $NiBr_2$•glyme (10.0 mol%), **L7** (15.0 mol%), 2,6-lutidine (2.0 equiv), Tris(trimethylsilyl)silane (2.0 equiv), *n*-Bu_4NBF_4 (1.0 equiv), and DMAc (2.0 mL) at rt, in an undivided cell for 6.0 h using platinum electrodes (1.0×1.0 cm^2) and Ni foam (2.0×3.0 cm^2) electrodes, argon. Yield was determined by ^1H NMR using CH_2Br_2 as an internal standard. Enantioselectivities were determined by chiral HPLC analysis.

2.2 Preparation of Substrate

The compound was prepared according to the reported literature procedure[1]：

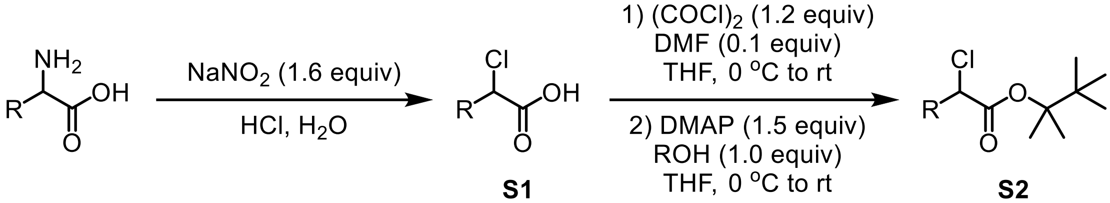

S1 **S2**

In a 250.0 mL over dried round-bottomed flask equipped with a Teflon-coated stir bar were added HCl (6 *N*, 65.0 mL), DL-Amino acid (50.0 mmol). The mixture is cooled to 0 °C in an ice/sodium chloride bath and a precooled solution of 5.5 g (1.6 mmol) of sodium nitrite in 20.0 mL of water is added dropwise at a rate of about 2.0 mL/min under vigorous stirring and efficient cooling so that the temperature of the reaction mixture is kept below 5 °C. After 5.0 h, the bath is removed and the reaction is allowed overnight at room temperature. The mixture was diluted with water. The aqueous layer was extracted with EA and the combined organic layer were washed with brine and dried (Na_2SO_4), filtered, and concentrated, get the crude **S1** and used without further purification.

$(COCl)_2$ (2.03 mL, 1.2 equiv) was added dropwise to a THF solution (15.0 mL) of **S1** (24.0 mmol) and DMF (0.16 mL, 0.10 equiv) at 0 °C, and the mixture was stirred at room temperature for 1 h. After removal of the solvent under reduced pressure (ca. 100 hPa), the crude acyl chloride was obtained as a yellow oil and used without further purification.

2,3,3-Trimethylbutan-2-ol (2.32 g, 20 mmol), DMAP (3.67 g, 1.5 equiv), and THF (30.0 mL) were added to another flask and cooled to 0 °C. The presynthesized acyl chloride was added to this mixture dropwise at 0 °C, and the resulting slurry mixture was stirred at 20 °C for 16.0 h. After the addition of Et_2O (50.0 mL), the organic layer was washed sequentially with 1.0 M aqueous HCl, saturated aqueous $NaHCO_3$, and brine. The organic layer was dried with Na_2SO_4, filtered, and concentrated, and the residue was purified by silica-gel column chromatography.

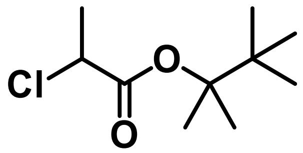

Prepared according to the general procedure using 2-Chloropropionyl chloride and purified by flash column chromatography (SiO_2, dry loaded, PE) to afford the 2,3,3-trimethylbutan-2-yl 2-chloropropanoate.

^{1}H NMR (400 MHz, $CDCl_3$) δ 4.31 (q, *J* = 7.0 Hz, 1H), 1.66 (d, *J* = 6.8 Hz, 3H), 1.52 (s, 3H), 1.52 (s, 3H), 0.99 (s, 9H).

All the resonances in ^{1}H NMR spectrum were consistent with the reported values.[1]

^{1}H and ^{13}C NMR spectra are provided below.

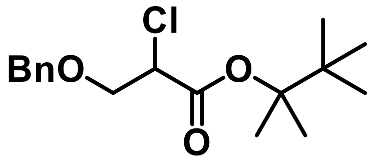

2,3,3-trimethylbutan-2-yl 3-(benzyloxy)-2-chloropropanoate

Prepared according to the general procedure using O-Benzyl-DL-Serine and purified by flash column chromatography (SiO_2, dry loaded, PE) to afford the 2,3,3-trimethylbutan-2-yl 3-(benzyloxy)-2-chloropropanoate.

^{1}H NMR (400 MHz, $CDCl_3$) δ 7.37 – 7.27 (m, 5H), 4.59 (s, 2H), 4.33 (t, *J* = 6.2 Hz, 1H), 3.88 (dd, *J* = 10.2, 6.5 Hz, 1H), 3.78 (dd, *J* = 10.2, 6.0 Hz, 1H), 1.52 (s, 3H), 1.52 (s, 3H), 0.97 (s, 9H).

^{13}C NMR (101 MHz, $CDCl_3$) δ 166.7, 137.3, 128.3, 127.8, 127.6, 89.6, 73.4, 71.2, 56.3, 38.3, 25.0, 20.2.

HRMS (ESI) calcd for $C_{17}H_{25}O_3Cl_1Na_1$ $[M+Na]^+$: 335.1384, found: 335.1382.

^{1}H and ^{13}C NMR spectra are provided below.

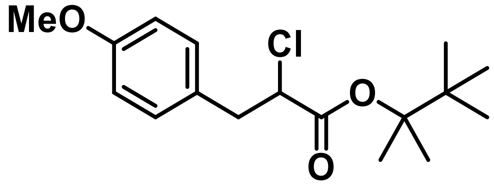

Prepared according to the general procedure using DL-homophenylalanine and purified by flash column chromatography (SiO_2, dry loaded, PE) to afford the 2,3,3-trimethylbutan-2-yl 2-chloro-3-(4-methoxyphenyl)propanoate.

^{1}H NMR (400 MHz, $CDCl_3$) δ 7.19 – 7.12 (m, 2H), 6.87 – 6.81 (m, 2H), 4.30 (t, *J* = 7.4 Hz, 1H), 3.79 (s, 3H), 3.26 (dd, *J* = 14.0, 7.4 Hz, 1H), 3.09 (dd, *J* = 14.0, 7.4 Hz, 1H), 1.48 (s, 3H), 1.42 (s, 3H), 0.92 (s, 9H).

^{13}C NMR (101 MHz, $CDCl_3$) δ 168.2, 158.8, 130.4, 128.1, 113.9, 89.5, 59.2, 55.3, 40.5, 38.4, 25.1, 20.3, 20.2.

HRMS (ESI) calcd for $C_{17}H_{25}O_3Cl_1Na_1$ $[M+Na]^+$: 335.1384, found: 335.1382.

^{1}H and ^{13}C NMR spectra are provided below.

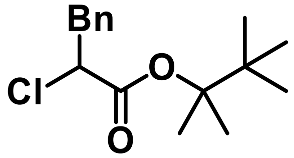

Prepared according to the general procedure using DL-phenylalanine and purified by flash column chromatography (SiO_2, dry loaded, PE) to afford the 2,3,3-trimethylbutan-2-yl 2-chloro-3-phenylpropanoate.

^{1}H NMR (400 MHz, $CDCl_3$) δ 7.34 – 7.28 (m, 2H), 7.28 – 7.20 (m, 3H), 4.36 (t, *J* = 7.4 Hz, 1H), 3.32 (dd, *J* = 14.0, 7.4 Hz, 1H), 3.15 (dd, *J* = 14.0, 7.4 Hz, 1H), 1.48 (s, 3H), 1.41 (s, 3H), 0.91 (s, 9H).

^{13}C NMR (101 MHz, $CDCl_3$) δ 168.12, 136.1, 129.3, 128.5, 127.2, 89.6, 59.0, 41.4, 38.4, 25.0, 20.3, 20.2.

All the resonances in ^{1}H NMR spectrum were consistent with the reported values.[2] ^{1}H and ^{13}C NMR spectra are provided below.

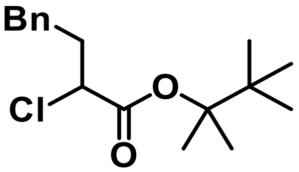

Prepared according to the general procedure using DL-homophenylalanine and purified by flash column chromatography (SiO_2, dry loaded, PE) to afford the 2,3,3-

trimethylbutan-2-yl 2-chloro-4-phenylbutanoate.

^{1}H NMR (400 MHz, CDCl$_3$) δ 7.33 – 7.27 (m, 2H), 7.24 – 7.18 (m, 3H), 4.15 (dd, *J* = 8.2, 5.6 Hz, 1H), 2.85 – 2.72 (m, 2H), 2.36 – 2.27 (m, 1H), 2.34 – 2.15 (m, 1H), 1.53 (s, 6H), 0.99 (s, 9H).

^{13}C NMR (101 MHz, CDCl$_3$) δ 168.39, 140.11, 128.57, 128.50, 126.31, 89.39, 58.23, 38.49, 36.59, 32.04, 25.11, 20.32.

All the resonances in ^{1}H NMR spectrum were consistent with the reported values.[2] ^{1}H and ^{13}C NMR spectra are provided below.

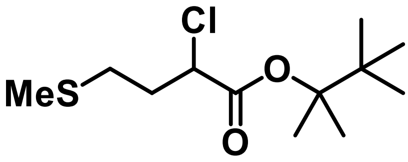

Prepared according to the general procedure using 2-Chloropentanoyl chloride and purified by flash column chromatography (SiO_2, dry loaded, PE) to afford the 2,3,3-trimethylbutan-2-yl 2-chloro-4-(methylthio)butanoate.

^{1}H NMR (400 MHz, CDCl$_3$) δ 4.40 (dd, *J* = 8.6, 5.2 Hz, 1H), 2.71 – 2.57 (m, 2H), 2.32 – 2.22 (m, 1H), 2.21 – 2.13 (m, 1H), 2.11 (s, 3H), 1.53 (s, 3H), 1.52 (s, 3H), 0.99 (s, 9H).

^{13}C NMR (101 MHz, CDCl$_3$) δ 168.2, 89.7, 57.5, 38.5, 34.2, 30.3, 25.1, 20.4, 20.3, 15.4.

HRMS (ESI) calcd for $C_{12}H_{23}O_2Cl_1S_1Na_1$ $[M+Na]^+$: 289.0999, found: 289.0996.

^{1}H and ^{13}C NMR spectra are provided below.

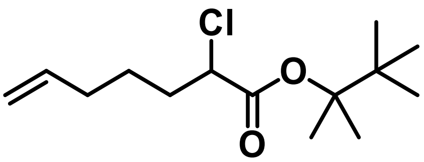

Prepared according to the general procedure using 2-Chloropentanoyl chloride and purified by flash column chromatography (SiO_2, dry loaded, PE) to afford the 2,3,3-trimethylbutan-2-yl 2-chlorohept-6-enoate.

^{1}H NMR (400 MHz, CDCl$_3$) δ 5.83 – 5.72 (m, 2H), 5.22 – 4.87 (m, 2H), 4.19 (dd, *J* = 7.8, 6.2 Hz, 1H), 2.16 – 2.04 (m, 2H), 2.04 – 1.83 (m, 2H), 1.61 – 1.48 (m, 2H), 1.52 (s, 6H), 0.99 (s, 9H).

^{13}C NMR (101 MHz, CDCl$_3$) δ 168.6, 137.8, 115.3, 89.3, 58.9, 38.5, 34.4, 32.9, 25.09, 25.08, 20.32, 20.30.

All the resonances in ^{1}H NMR spectrum were consistent with the reported values.[1]

^{1}H and ^{13}C NMR spectra are provided below.

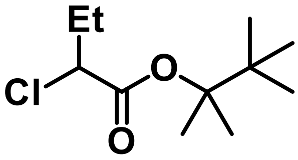

Prepared according to the general procedure using 2-Chlorobutyryl chloride and purified by flash column chromatography (SiO_2, dry loaded, PE) to afford the 2,3,3-trimethylbutan-2-yl 2-chlorobutanoate.

^{1}H NMR (400 MHz, CDCl$_3$) δ 4.14 (dd, *J* = 7.2, 6.0 Hz, 1H), 2.07 – 1.98 (m, 1H), 1.97 – 1.89 (m, 1H), 1.52 (s, 3H), 1.52 (s, 3H), 1.03 (t, *J* = 7.4 Hz, 3H), 0.99 (s, 9H).

^{13}C NMR (101 MHz, CDCl$_3$) δ 168.5, 89.2, 60.5, 38.5, 28.5, 25.1, 20.32, 20.28, 10.4.

All the resonances in ^{1}H NMR spectrum were consistent with the reported values.[1]

^{1}H and ^{13}C NMR spectra are provided below.

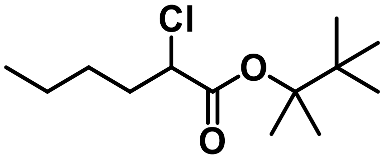

Prepared according to the general procedure using 2-Chloropentanoyl chloride and purified by flash column chromatography (SiO_2, dry loaded, PE) to afford the 2,3,3-trimethylbutan-2-yl 2-chlorohexanoate.

^{1}H NMR (400 MHz, CDCl$_3$) δ 4.17 (dd, *J* = 7.6, 6.4 Hz, 1H), 2.04 – 1.94 (m, 1H), 1.94 – 1.81 (m, 1H), 1.53 (s, 3H), 1.52 (s, 3H), 1.47 – 1.28 (m, 4H), 0.99 (s, 9H), 0.91 (t, *J* = 7.2 Hz, 3H).

^{13}C NMR (101 MHz, $CDCl_3$) δ 168.7, 89.2, 59.1, 38.5, 34.8, 28.0, 25.1, 22.0, 20.3, 20.2, 13.8.

All the resonances in ^{1}H NMR spectrum were consistent with the reported values.[1]

^{1}H and ^{13}C NMR spectra are provided below.

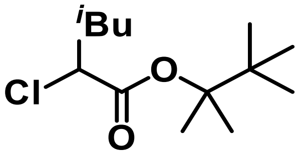

Prepared according to the general procedure using DL-Leucine and purified by flash column chromatography (SiO_2, dry loaded, PE) to afford the 2,3,3-trimethylbutan-2-yl 2-chloro-4-methylpentanoate.

^{1}H NMR (400 MHz, $CDCl_3$) δ 4.23 – 4.16 (m, 1H), 1.84 – 1.75 (m, 3H), 1.52 (s, 3H), 1.51 (s, 3H), 0.99 (s, 9H), 0.97 – 0.94 (m, 3H), 0.94 – 0.91 (m, 3H).

All the resonances in ^{1}H NMR spectrum were consistent with the reported values.[1]

^{1}H and ^{13}C NMR spectra are provided below.

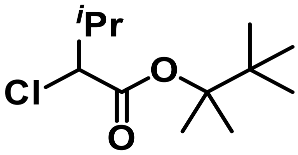

Prepared according to the general procedure using 2-Chloro-3-methylbutanoyl chloride and purified by flash column chromatography (SiO_2, dry loaded, PE) to afford the 2,3,3-trimethylbutan-2-yl 2-chloro-3-methylbutanoate.

^{1}H NMR (400 MHz, $CDCl_3$) δ 4.00 (d, J = 6.8 Hz, 1H), 2.31 – 2.22 (m, 1H), 1.53 (s, 3H), 1.53 (s, 3H), 1.04 (s, 3H), 1.03 (s, 3H), 0.99 (s, 9H).

All the resonances in ^{1}H NMR spectrum were consistent with the reported values.[2]

^{1}H and ^{13}C NMR spectra are provided below.

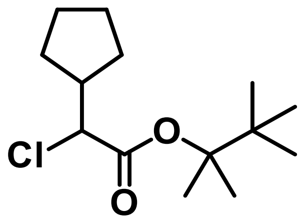

Prepared according to the general procedure using DL-cyclopentylglycine and purified by flash column chromatography (SiO_2, dry loaded, PE) to afford the 2,3,3-trimethylbutan-2-yl 2-chloro-2-cyclopentylacetate.

^{1}H NMR (400 MHz, $CDCl_3$) δ 4.01 (d, *J* = 8.4 Hz, 1H), 2.50 – 2.39 (m, 1H), 1.92 – 1.82 (m, 1H), 1.79 – 1.71 (m, 1H), 1.70 – 1.55 (m, 4H), 1.52 (s, 6H), 1.49 – 1.44 (m, 1H), 1.37 – 1.26 (m, 1H), 0.99 (s, 9H).

^{13}C NMR (101 MHz, $CDCl_3$) δ 168.6, 89.1, 63.8, 44.1, 38.5, 30.3, 29.8, 29.7, 25.8, 25.2, 25.1, 20.3, 20.2.

All the resonances in ^{1}H NMR spectrum were consistent with the reported values.[2]

^{1}H and ^{13}C NMR spectra are provided below.

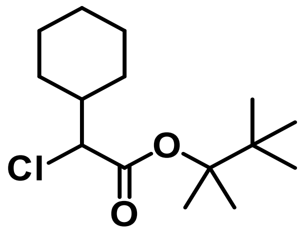

Prepared according to the general procedure using DL-cyclohexylalanine and purified by flash column chromatography (SiO_2, dry loaded, PE) to afford the 2,3,3-trimethylbutan-2-yl 2-chloro-2-cyclohexylacetate.

^{1}H NMR (400 MHz, $CDCl_3$) δ 3.95 (d, *J* = 7.6 Hz, 1H), 2.01 – 1.84 (m, 2H), 1.81 – 1.74 (m, 2H), 1.70 – 1.63 (m, 2H), 1.53 (s, 3H), 1.52 (s, 3H), 1.30 – 1.06 (m, 5H), 0.99 (s, 9H).

^{13}C NMR (101 MHz, $CDCl_3$) δ 168.3, 89.2, 64.8, 41.9, 38.45, 29.9, 28.6, 25.9, 25.8, 25.6, 25.1, 20.3, 20.2.

HRMS (ESI) calcd for $C_{15}H_{27}O_2Cl_1Na_1$ $[M+Na]^+$: 297.1592, found: 297.1589.

^{1}H and ^{13}C NMR spectra are provided below.

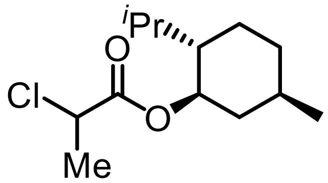

Prepared according to the general procedure using L-Menthol and purified by flash column chromatography (SiO_2, dry loaded, PE) to afford the (1*R*,2*S*,5*R*)-2-isopropyl-5-methylcyclohexyl 2-chloropropanoate.

^{1}H NMR (400 MHz, $CDCl_3$) δ 4.75 – 4.68 (m, 1H), 4.36 (q, *J* = 7.0 Hz, 1H), 2.03 – 1.94 (m, 1H), 1.93 – 1.83 (m, 1H), 1.72 – 1.69 (m, 1H), 1.69 – 1.66 (m, 4H), 1.55 – 1.40 (m, 2H), 1.11 – 0.96 (m, 2H), 0.92 – 0.88 (m, 6H), 0.77 – 0.74 (m, 3H).

^{13}C NMR (101 MHz, $CDCl_3$) δ 169.6, 76.0, 53.0, 47.0, 40.5, 34.1, 31.4, 26.1, 23.3, 22.0, 21.5, 20.7, 16.1.

HRMS (ESI) calcd for $C_{13}H_{23}O_2Cl_1Na_1$ $[M+Na]^+$: 269.1284, found: 269.1286.

2.3. Suboptimal and Unsuccessful Substrates

Supplementary Table 8: Suboptimal and unsuccessful substrates

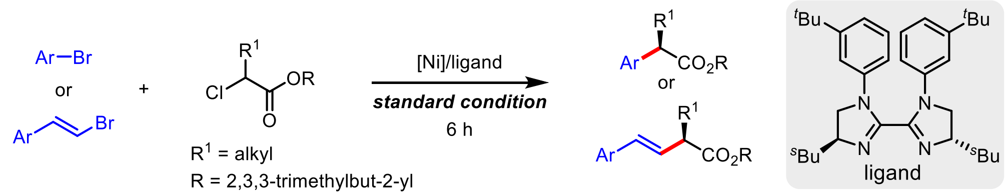

Substrates that did not provide desired products: (low conversion)

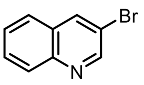

<5% yield

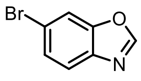

<5% yield

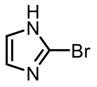

<5% yield

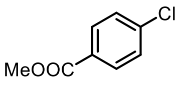

5% yield, 85% ee

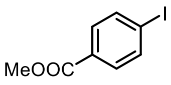

14% yield, 91% ee

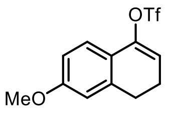

<5% yield

Substrates that reacted: (low yield or moderate enantioselective)

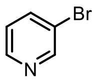

30% yield, 80% ee

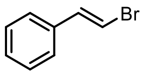

36% yield, 52% ee

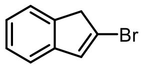

50% yield, 72% ee

a-chloroesters derived from complex molecules: (low yield or moderate enantioselective)

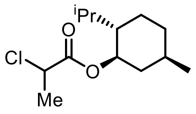

60% yield
50% *dr*
react with Methyl 4-bromobenzoate

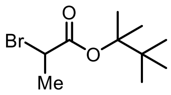

16% yield
91% *ee*
react with Methyl 4-bromobenzoate

Standard condition: aryl bromide/iodine/chloride (0.2 mmol), (*rac*)-*a*-chloroesters (2.0 equiv), $NiBr_2$·glyme (10.0 mol%), Ligand (15.0 mol%), DMAc (0.1 M), *n*-Bu_4NBF_4 (1.0 equiv), TTMSS (3.5 equiv), 2,6-lutidine (3.0 equiv), platinum electrodes (+) and Ni foam (-), constant voltage (U_{cell} = 2.9 V, 6.0 h for 0.2 mmol scale), rt, isolated yield.

2.4 General Procedure for Electrochemical C-C Coupling

Procedure for the electrochemical, nickel catalyzed C–C coupling from Aryl Bromide

Racemic Reaction:

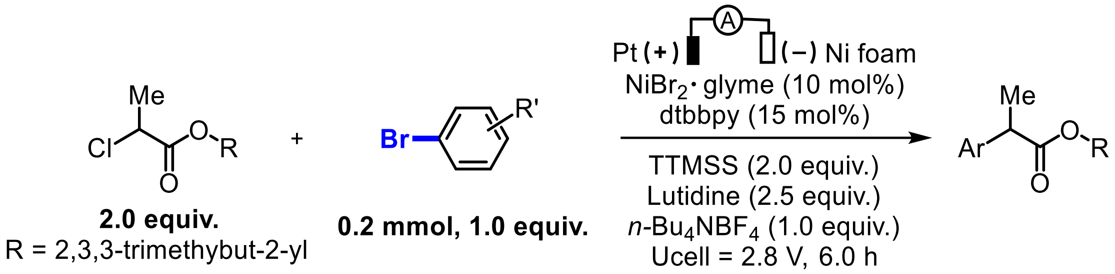

Preparation of nickel/ligand stock solution in DMAc (0.01 M, solution A): a screw-capped tube was charged with $NiBr_2$•glyme (0.1 mmol, 31.0 mg) and 4,4'-di-tert-butyl-2,2'-bipyridine (0.12 mmol, 26.0 mg). This tube was evacuated and backfilled with an argon balloon. DMAc (10 mL) was then added; the resulting mixture was stirred until a homogeneous solution was obtained (heating at 60 °C was found to aid the homogenization process in some cases).

Procedure: In a nitrogen-filled glove box, an over-dried 10 mL hydrogenation tube charged with a stir bar, Aryl bromide (0.2 mmol), **solution A** (1.0 mL), $TBABF_4$ (62.0 mg, 0.2 mmol) were added to the electrochemical cell and the mixture was stirred for over 15 mins. Then 2,3,3-trimethylbutan-2-yl 2-chloropropanoate (90.0 mg, 0.4 mmol), 2,6-lutidine (54.0 mg, 0.5 mmol), TTMSS (100.0 mg, 0.4 mmol), DMAc (1.0 mL) were added to the electrochemical cell. The tube was installed with a Ni foam (2.0 x 3.0 cm^2) as the cathode and the Pt (1.0 x 1.0 x 0.3 cm^3) as the anode. The seal-tube was sealed and removed from the glovebox. The reaction mixture was electrolyzed under a constant current of 2.8 V until the complete consumption of the starting materials as judged by TLC (about 6 hours). After the reaction, The aqueous layer was extracted with EtOAc (3 x equal volume) and the combined organics were washed with sat. brine (4 x equal volume), dried over $MgSO_4$, filtered, and concentrated *in vacuo*. The crude

product was purified by column chromatography to furnish the desired product.

Chiral Reaction:

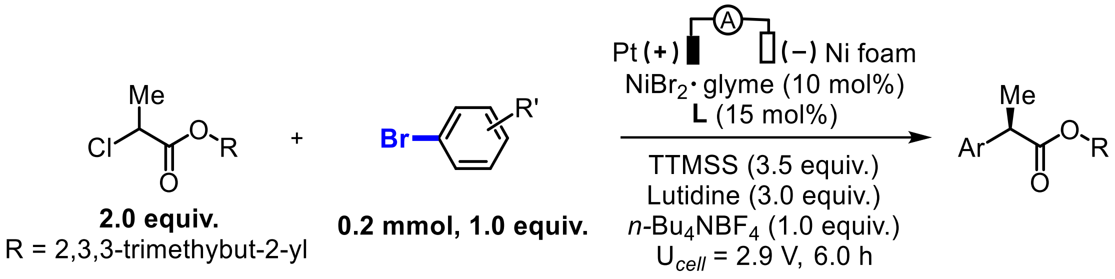

Photographic Guide for Electrochemical C-C coupling

Easily hand-made electrochemical cell

(0.2 mmol for the Aryl halides, 2.0 mL DMAc, U_{cell} = 2.9 V for 6.0 h)

Procedure: In a nitrogen-filled glove box, an over-dried 10 mL hydrogenation tube charged with a stir bar, Aryl bromide (0.2 mmol), $NiBr_2$•glyme (10.0 mol%, 6.2 mg), **L** (15.0 mol%, 16.0 mg). $TBABF_4$ (62.0 mg, 0.2 mmol) were added to the electrochemical cell and the mixture was stirred for over 20 mins.

Then 2,3,3-trimethylbutan-2-yl 2-chloropropanoate (90.0 mg, 0.4 mmol), 2,6-lutidine (62.0 mg, 0.6 mmol), TTMSS (175.0 mg, 0.70 mmol), DMAc (1.0 mL) were added to the electrochemical cell. The tube was installed with a Ni foam (2.0 x 3.0 cm^2) as the cathode and the Pt (1.0 x 1.0 x 0.3 cm^3) as the anode. The seal-tube was sealed and removed from the glovebox. The reaction mixture was electrolyzed under a constant current of 2.9 V until the complete consumption of the starting materials as judged by TLC (about 6 hours). After the reaction, The aqueous layer was extracted with EtOAc (3 x equal volume) and the combined organics were washed with sat. brine (4 x equal volume), dried over $MgSO_4$, filtered, and concentrated *in vacuo*. The crude product was purified by column chromatography to furnish the desired product.

Step 0. Overview of materials used.

From left to right: 1) an Pt electrode. 2) The Ni foam (as the cathode).

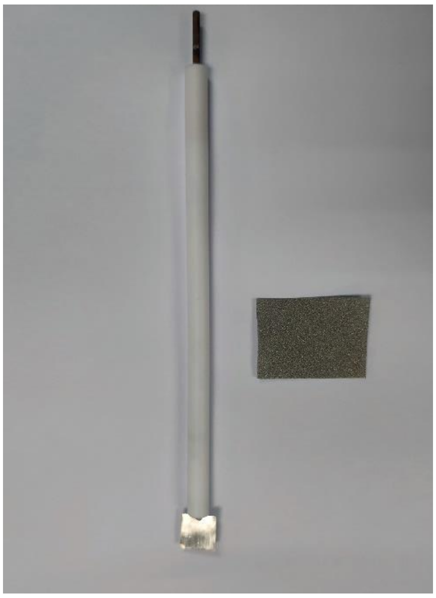

Step 1. Preparation of the electrode

Wrap the Ni foam with copper wire. (as the cathode)

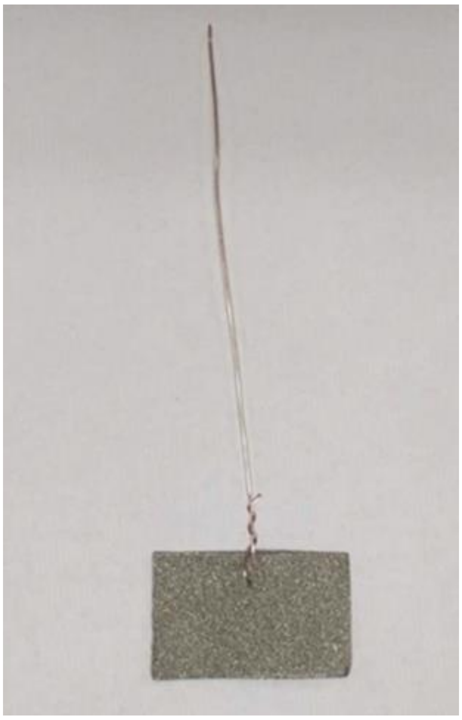

Step 2. Assembling the cell

The stopper was fitted into the tube.

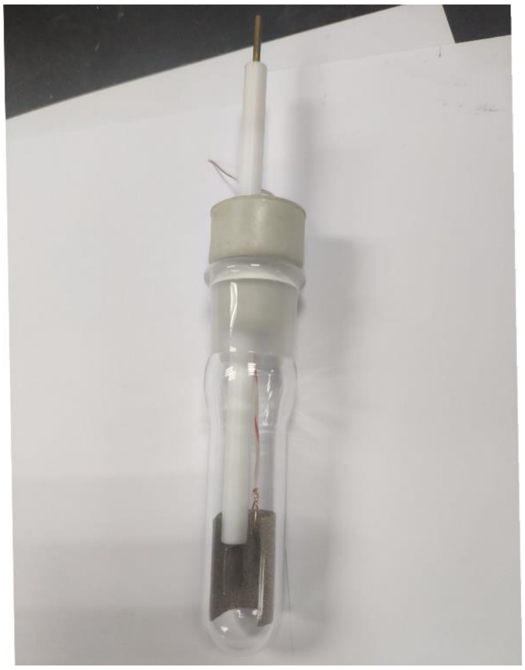

Step 3. Electrolysis

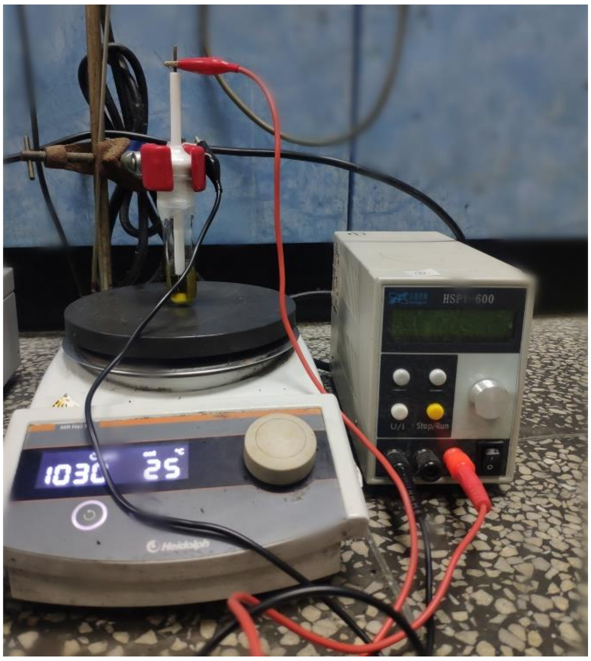

…….. (electrolysis)

2.5 Preparation and X-ray Diffraction of Nickel Pre-Catalyst

Ni pre-catalyst synthesis:

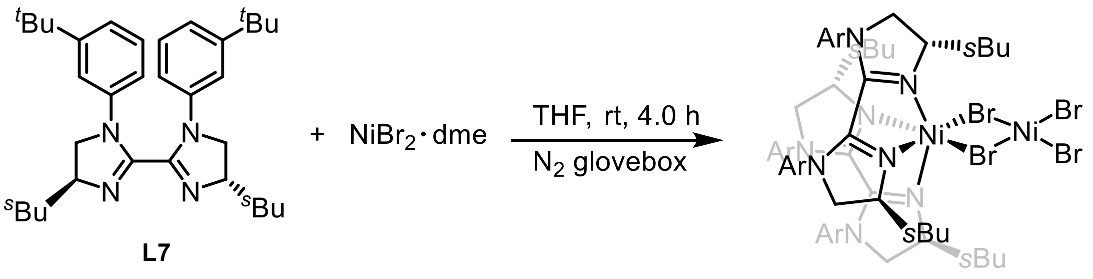

Ni-1

Ar = 3-*t*BuC$_6$H$_4$

Procedure: To an overdried 20.0 mL Vial was added NiBr$_2$•DME (0.1 mmol, 31.0 mg), **L7** (0.11 mol, 56.6 mg), and THF (5.0 mL) in a N$_2$-filled glovebox. The mixture was stirred at room temperature for 4 hours to afford a green solution. The resulting solution was filtered and removed under reduced pressure. The remaining oil was washed with Et$_2$O (3.0 X 4.0 mL) and then triturated with Et$_2$O until Nickel pre-catalyst (70% yield, 51.3 mg) was isolated as a pale green solid. The layering of a THF solution with Et$_2$O at an N$_2$-filled glovebox resulted in the formation of a green crystal of Nickel pre-catalyst.

CCDC Number of Nickel pre-catalyst:

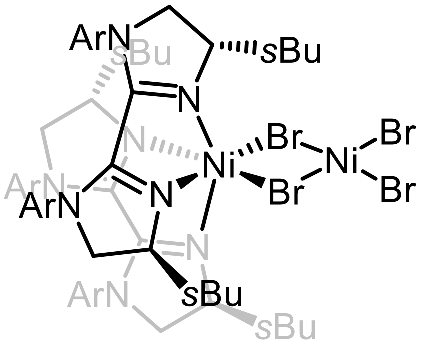

Ni-1

Ar = 3-*t*BuC$_6$H$_4$

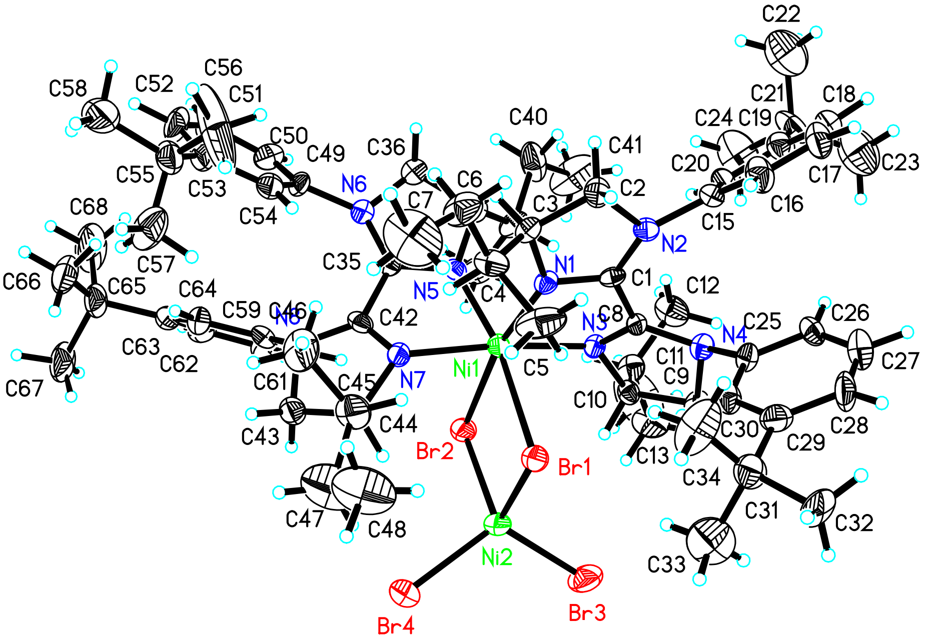

X-ray diffraction of Nickel pre-catalyst.

CCDC number: 2160390

Table 1. Crystal data and structure refinement for mo_d8v22142_0m.

Identification code	mo_d8v22142_0m
Empirical formula	C68 H100 Br4 N8 Ni2
Formula weight	1466.61
Temperature	213(2) K
Wavelength	0.71073 Å
Crystal system	Monoclinic

Space group	P 21
Unit cell dimensions	a = 14.5404(8) Å = 90°.
	b = 19.8947(12) Å
	=108.836(2)°.
	c = 15.4738(9) Å = 90°.
Volume	4236.5(4) $Å^3$
Z	2
Density (calculated)	1.150 Mg/m^3
Absorption coefficient	2.367 mm^{-1}
F(000)	1520
Crystal size	0.120 x 0.100 x 0.050 mm^3
Theta range for data collection	2.526 to 25.498°.
Index ranges	-17<=h<=17, -24<=k<=24, -17<=l<=18
Reflections collected	46554
Independent reflections	15704 [R(int) = 0.0842]
Completeness to theta = 25.242°	99.8 %
Absorption correction	Semi-empirical from equivalents
Max. and min. transmission	0.7456 and 0.5945
Refinement method	Full-matrix least-squares on F^2
Data / restraints / parameters	15704 / 99 / 798
Goodness-of-fit on F^2	0.904
Final R indices [I>2sigma(I)]	R1 = 0.0595, wR2 = 0.1262
R indices (all data)	R1 = 0.1111, wR2 = 0.1446
Absolute structure parameter	0.016(9)
Extinction coefficient	n/a
Largest diff. peak and hole	0.403 and -0.444 $e.Å^{-3}$

Involvement of the Nickel pre-catalyst complex in the reaction

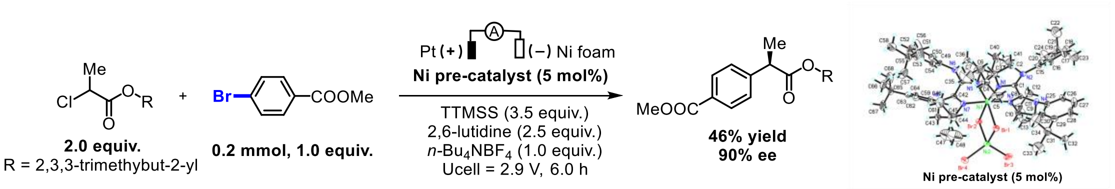

Procedure: In a nitrogen-filled glove box, an over-dried 10 mL hydrogenation tube charged with a stir bar, Aryl bromide (0.2 mmol), Ni-pre-catalyst (5.0 mol%, 7.4 mg), $TBABF_4$ (62.0 mg, 0.2 mmol) were added to the electrochemical cell and the mixture was stirred for over 20 mins.

Then 2,3,3-trimethylbutan-2-yl 2-chloropropanoate (90.0 mg, 0.4 mmol), 2,6-lutidine (62.0 mg, 0.6 mmol), TTMSS (175.0 mg, 0.70 mmol), DMAc (1.0 mL) were added to the electrochemical cell. The tube was installed with a Ni foam (2.0 x 3.0 cm^2) as the cathode and the Pt (1.0 x 1.0 x 0.3 cm^3) as the anode. The seal-tube was sealed and removed from the glovebox. The reaction mixture was electrolyzed under a constant current of 2.9 V until the complete consumption of the starting materials as judged by TLC (about 6 hours). After the reaction, The aqueous layer was extracted with EtOAc (3 x equal volume) and the combined organics were washed with sat. brine (4 x equal volume), dried over $MgSO_4$, filtered, and concentrated *in vacuo*. The crude product was purified by column chromatography to furnish the desired product as a colorless oil (28.0 mg, 46% yield, 90% ee).

2.6 Cyclic Voltammetry

Cyclic voltammograms were recorded with a CHI660E potentiostat at room temperature in DMAc. *n*-Bu_4NPF_6 (0.1 M) was used as the supporting electrolyte, and a Glass Carbon electrode was used as the working electrode. The auxiliary electrode was a Pt electrode. All potentials are referenced against the $Ag/AgNO_3$ (0.1 M in DMAc) redox couple. The scan rate was 100 mV s^{-1}.

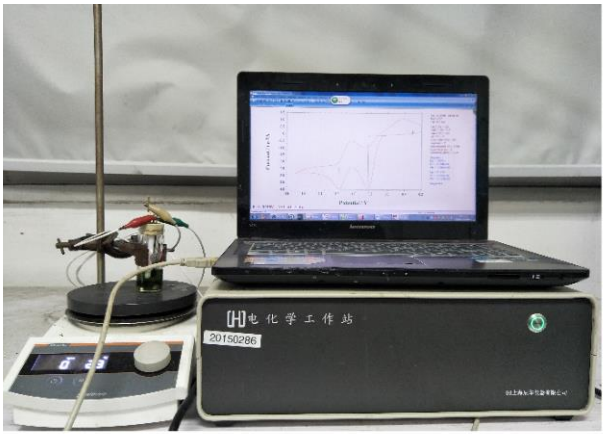

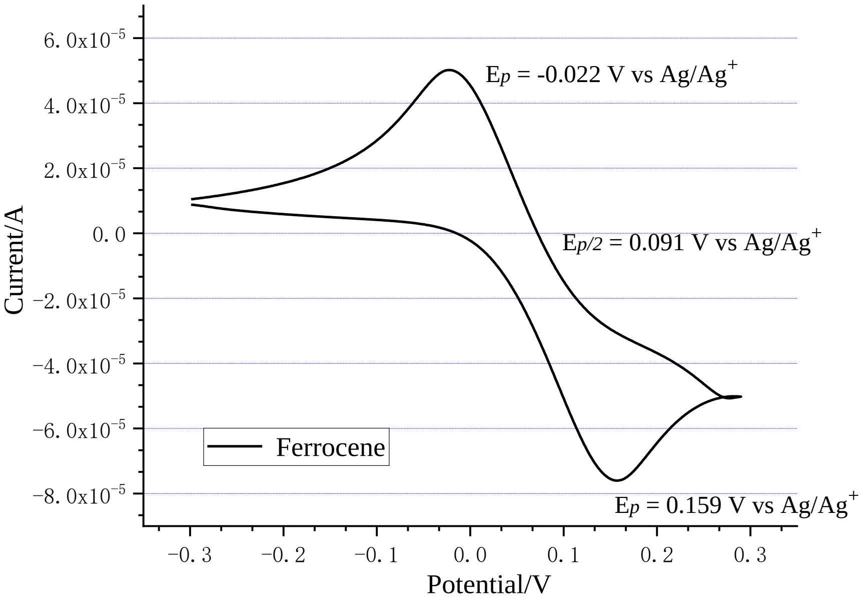

Supplementary Figure 1. Cyclic voltammograms of 5mM Ferrocene, DMAc solvent, 0.1M *n*-Bu_4NPF_6 supporting electrolyte, GC working electrode, Pt electrode as counter electrode, 100 mV/s scan rate.

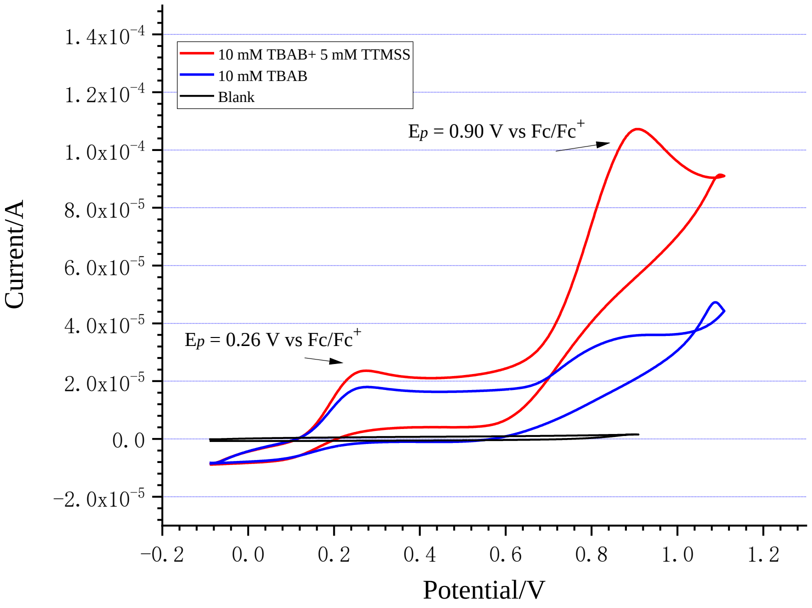

Supplementary Figure 2. Cyclic voltammograms of 10mM TBAB (Blue line), 5mM TTMSS (Red line), DMAc solvent, 0.1M *n*-Bu_4NPF_6 supporting electrolyte, GC working electrode, Pt electrode as counter electrode, 100 mV/s scan rate.

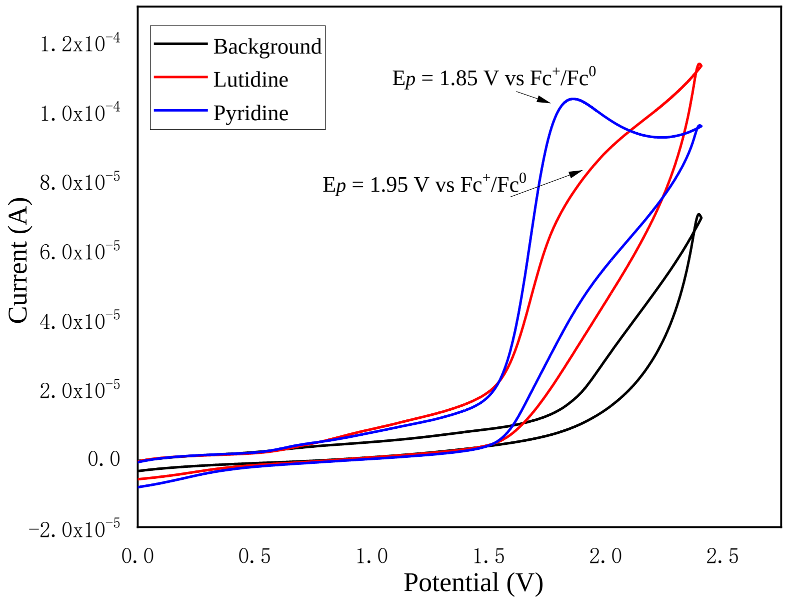

Supplementary Figure 3. Cyclic voltammograms of 10mM Pyridine (Blue line) and 2,6-lutidine (Red line), MeCN solvent, 0.1M *n*-Bu_4NPF_6 supporting electrolyte, GC working electrode, Pt electrode as the counter electrode, 100 mV/s scan rate.

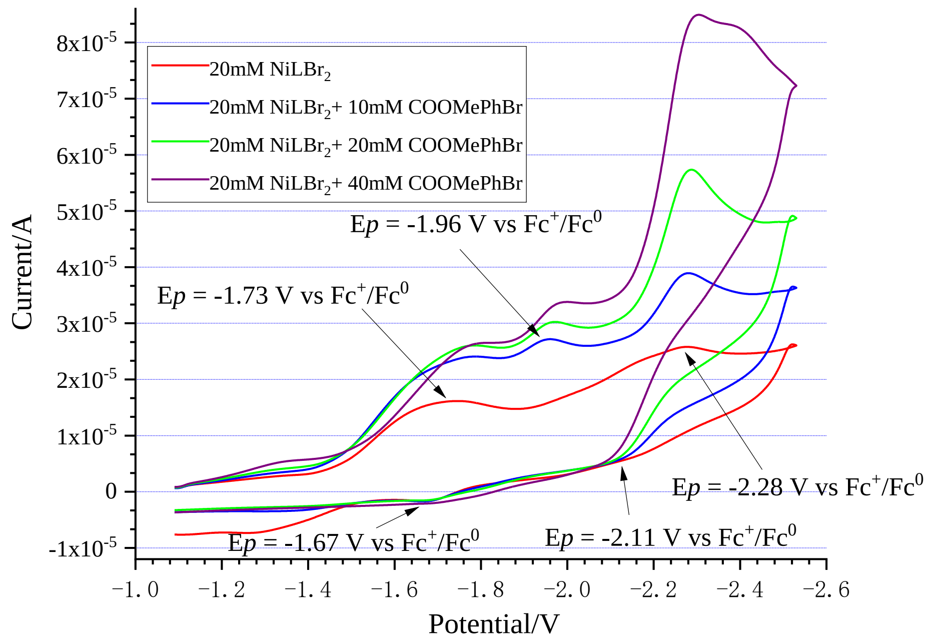

Supplementary Figure 4. Cyclic voltammograms of 20.0 mM $NiBr_2$•glyme, 20.0 mM Ligand (red line), and 10.0 mM *p*-COOMePhBr (blue line), 20.0 mM *p*-COOMePhBr (green line), 40.0 mM *p*-COOMePhBr (Purple line), DMAc solvent, 0.1M *n*-Bu_4NPF_6 supporting electrolyte, GC working electrode, Pt electrode as counter electrode, 100 mV/s scan rate.

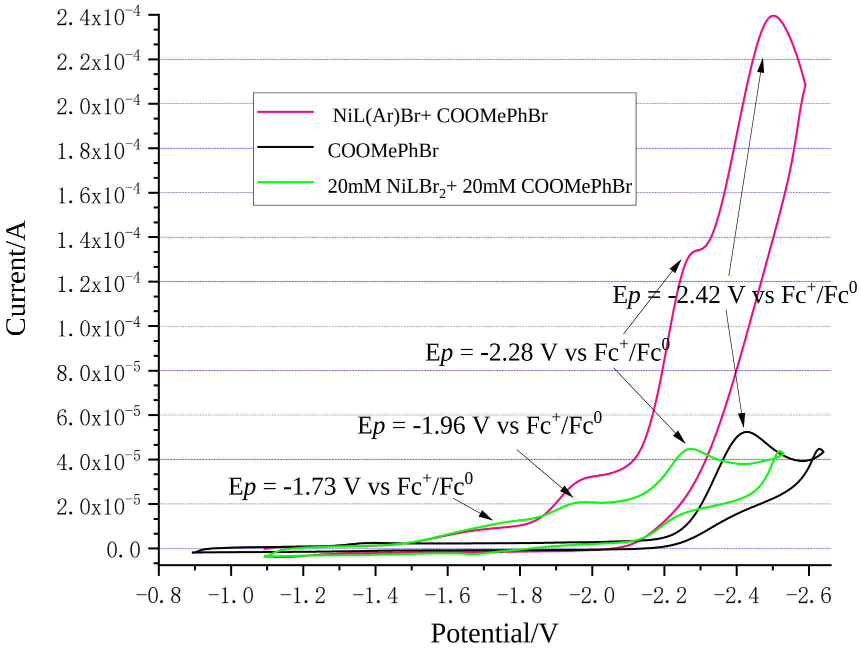

Supplementary Figure 5. Cyclic voltammograms of 20.0 mM $NiBr_2$•glyme, 20.0 mM Ligand and 20.0 mM *p*-COOMePhBr (green line), *p*-COOMePhBr (Black line), [(L)Ni(II)(*p*-COOMePh)Br] and *p*-COOMePhBr (Pink line), DMAc solvent, 0.1M *n*-Bu_4NPF_6 supporting electrolyte, GC working electrode, Pt electrode as counter electrode, 100 mV/s scan rate.

2.7 Characterization Data for the Products

Methyl (*R*)-4-(1-oxo-1-((2,3,3-trimethylbutan-2-yl)oxy)propan-2-yl)benzoate (2a)

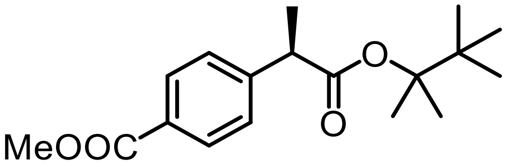

A colorless oil (40.1 mg, 66% yield, 91% ee) from methyl 4-bromobenzoate (43.0 mg, 0.2 mmol).

^{1}H NMR (400 MHz, CDCl$_3$) δ 7.97 (d, *J* = 8.4 Hz, 2H), 7.33 (d, *J* = 8.4 Hz, 2H), 3.89 (s, 3H), 3.68 (q, *J* = 7.2 Hz, 1H), 1.47 (d, *J* = 7.2 Hz, 3H), 1.45 (s, 3H), 1.36 (s, 3H), 0.80 (s, 9H).

^{13}C NMR (101 MHz, CDCl$_3$) δ 172.9, 166.9, 146.4, 129.8, 128.67, 127.5, 87.6, 52.0, 47.0, 38.3, 24.9, 20.4, 20.1, 17.9.

IR (neat): 2977, 1721, 1611, 1435, 1375, 1276, 1109, 1019, 967, 848, 747 cm^{-1}.

HRMS (ESI) calcd for $C_{18}H_{26}O_4Na_1$ [M+Na]$^+$: 329.1723, found: 329.1725.

Enantioselective ratio = 95.5:4.5, determined by HPLC (Daicel Chiralpak IC-H Column, *n*-Hexane: *i*-PrOH = 98:2, flow rate 1.0 mL/min, T = 25 °C, λ = 214 nm): t_R = 8.87 min (major), t_R = 9.69 min (minor).

$[\alpha]_D^{30}$ = -51.05 (c = 0.2, CHCl$_3$).

Methyl (*R*)-3-(1-oxo-1-((2,3,3-trimethylbutan-2-yl)oxy)propan-2-yl)benzoate (2b)

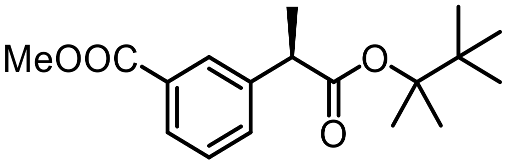

A colorless oil (36.7 mg, 60% yield, 90% ee) from methyl 3-bromobenzoate (43.0 mg, 0.2 mmol).

^{1}H NMR (400 MHz, CDCl$_3$) δ 7.96 – 7.95 (m, 1H), 7.91 – 7.88 (m, 1H), 7.47 – 7.44 (m, 1H), 7.39 – 7.35 (m, 1H), 3.89 (s, 3H), 3.68 (q, *J* = 7.2 Hz, 3H), 1.48 (d, *J* = 7.2 Hz, 3H), 1.45 (s, 3H), 1.37 (s, 3H), 0.82 (s, 9H).

^{13}C NMR (101 MHz, CDCl$_3$) δ 173.1, 166.9, 141.6, 132.1, 130.3, 128.7, 128.5, 128.1, 87.5, 52.0, 46.7, 38.3, 24.9, 20.4, 20.2, 18.0.

IR (neat): 2976, 1721, 1588, 1434, 1282, 1197, 1127, 976, 938, 847, 747 cm^{-1}.

HRMS (ESI) calcd for $C_{18}H_{26}O_4Na_1$ [M+Na]$^+$: 329.1723, found: 329.1725.

Enantioselective ratio = 95.0:5.0, determined by HPLC (Daicel Chiralpak IC-H Column, *n*-Hexane: *i*-PrOH = 98:2, flow rate 1.0 mL/min, T = 25 °C, λ = 214 nm): t_R = 12.41 min (minor), t_R = 13.33 min (major).

[α]$_D^{29.1}$ = -36.68 (c = 0.2, CHCl$_3$).

Methyl (*R*)-2-(1-oxo-1-((2,3,3-trimethylbutan-2-yl)oxy)propan-2-yl)benzoate (2c)

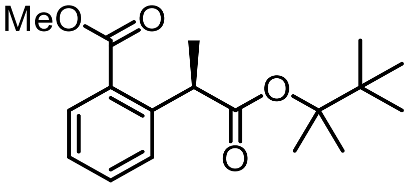

A colorless oil (19.0 mg, 31% yield, 71% ee) from methyl 2-bromobenzoate (43.0 mg, 0.2 mmol).

^{1}H NMR (400 MHz, CDCl$_3$) δ 7.88 (dd, *J* = 8.0, 1.6 Hz, 1H), 7.49 – 7.45 (m, 1H), 7.36 – 7.33 (m, 1H), 7.30 – 7.26 (m, 1H), 4.53 (q, *J* = 7.2 Hz, 1H), 3.88 (s, 3H), 1.50 (d, *J* = 7.2 Hz, 3H), 1.46 (s, 3H), 1.40 (s, 3H), 0.72 (s, 9H).

^{13}C NMR (101 MHz, CDCl$_3$) δ 173.6, 168.0, 142.8, 132.1, 130.6, 129.5, 128.5, 126.45, 87.0, 52.0, 43.4, 38.2, 24.8, 20.4, 20.1, 17.9.

IR (neat): 2976, 1719, 1601, 1433, 1374, 1242, 1178, 1090, 1058, 848, 711 cm^{-1}.

HRMS (ESI) calcd for $C_{18}H_{26}O_4Na_1$ [M+Na]$^+$: 329.1723, found: 329.1725.

Enantioselective ratio = 14.5:85.5, determined by HPLC (Daicel Chiralpak IC-H Column, *n*-Hexane: *i*-PrOH = 98:2, flow rate 1.0 mL/min, T = 25 °C, λ = 214 nm): t_R = 6.50 min (minor), t_R = 6.97 min (major).

[α]$_D^{29.3}$ = -22.11 (c = 0.2, CHCl$_3$).

Tert-butyl (*R*)-4-(1-oxo-1-((2,3,3-trimethylbutan-2-yl)oxy)propan-2-yl)benzoate (2d)

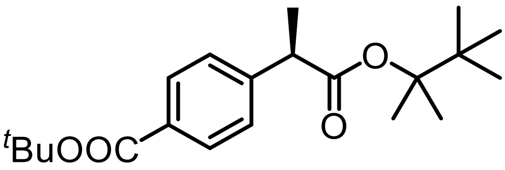

A colorless oil (47.0 mg, 65% yield, 90% ee) from tert-butyl 4-bromobenzoate (51.0 mg, 0.2 mmol).

^{1}H NMR (400 MHz, CDCl$_3$) δ 7.93 (d, J = 8.4 Hz, 2H), 7.31 (d, J = 8.0 Hz, 2H), 3.68 (q, J = 7.2 Hz, 1H), 1.58 (s, 9H), 1.46 (t, J = 3.6 Hz, 6H), 1.37 (s, 3H), 0.83 (s, 9H).

^{13}C NMR (101 MHz, CDCl$_3$) δ 173.0, 165.6, 145.9, 130.6, 129.7, 127.31, 87.5, 80.9, 47.0, 38.3, 28.17, 25.0, 20.4, 20.2, 18.0.

IR (neat): 2976, 1712, 1610, 1368, 1289, 1111, 1018, 847, 776, 759 cm^{-1}.

HRMS (ESI) calcd for $C_{21}H_{32}O_4Na_1$ $[M+Na]^+$: 371.2193, found: 371.2191.

Enantioselective ratio = 95.0:5.0, determined by HPLC (Daicel Chiralpak IC-H Column, *n*-Hexane: *i*-PrOH = 98.0:2.0, flow rate 1.0 mL/min, T = 25 °C, λ = 214 nm): t_R = 5.68 min (major), t_R = 6.14 min (minor).

$[\alpha]_D^{30.2}$ = -9.35 (c = 1.0, CHCl$_3$).

2,3,3-trimethylbutan-2-yl (*R*)-2-(4-benzoylphenyl)propanoate (2e)

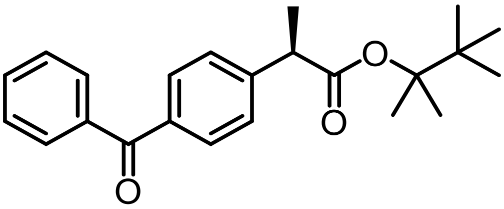

A colorless oil (43.0 mg, 61% yield, 88% ee) from 4-bromobenzophenone (53.0 mg, 0.2 mmol).

^{1}H NMR (400 MHz, CDCl$_3$) δ 7.80 – 7.76 (m, 2H), 7.73 – 7.71 (m, 1H), 7.67 – 7.64 (m, 1H), 7.60 – 7.55 (m, 1H), 7.52 – 7.40 (m, 4H), 3.71 (q, J = 7.2 Hz, 1H), 1.49 (d, J = 7.2 Hz, 3H), 1.46 (s, 3H), 1.40 (s, 3H), 0.83 (s, 9H).

^{13}C NMR (101 MHz, CDCl$_3$) δ 173.1, 141.6, 137.8, 137.6, 132.4, 131.5, 130.0, 129.1, 128.6, 128.4, 128.2, 87.5, 46.8, 38.3, 24.9, 20.4, 20.2, 18.1.

IR (neat): 2975, 1722, 1659, 1448, 1312, 1174, 1055, 937, 786, 699 cm^{-1}.

HRMS (ESI) calcd for $C_{23}H_{28}O_3Na_1$ $[M+Na]^+$: 375.1931, found: 375.1933.

Enantioselective ratio = 94.0:6.0, determined by HPLC (Daicel Chiralpak IC-H Column, *n*-Hexane: *i*-PrOH = 98.0:2.0, flow rate 0.7 mL/min, T = 25 °C, λ = 214 nm): t_R = 25.03 min (major), t_R = 26.40 min (minor).

$[\alpha]_D^{26.8}$ = -4.78 (c = 1.0, $CHCl_3$).

2,3,3-trimethylbutan-2-yl (*R*)-2-(4-acetylphenyl)propanoate (2f)

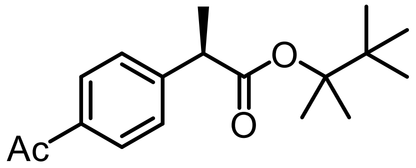

A colorless oil (42.9 mg, 74% yield, 90% ee) from 4-bromoacetophenone (40.0 mg, 0.2 mmol).

^{1}H NMR (400 MHz, $CDCl_3$) δ 7.90 (d, *J* = 8.4 Hz, 2H), 7.36 (d, *J* = 8.4 Hz, 2H), 3.69 (q, *J* = 7.2 Hz, 1H), 2.58 (s, 3H), 1.47 (d, *J* = 7.2 Hz, 3H), 1.45 (s, 3H), 1.37 (s, 3H), 0.82 (s, 9H).

^{13}C NMR (101 MHz, $CDCl_3$) δ 197.7, 172.8, 146.7, 135.8, 128.6, 127.7, 87.6, 47.0, 38.3, 26.6, 25.0, 20.4, 20.2, 17.9.

Enantioselective ratio = 95.0:5.0, determined by HPLC (Daicel Chiralpak IC-H Column, *n*-Hexane: *i*-PrOH = 99:1, flow rate 1.0 mL/min, T = 25 °C, λ = 214 nm): t_R = 23.96 min (major), t_R = 26.03 min (minor).

$[\alpha]_D^{29.3}$ = -36.07 (c = 0.2, $CHCl_3$).

All the resonances in ^{1}H NMR spectrum were consistent with the reported values.[2]

^{1}H and ^{13}C NMR spectra are provided below.

2,3,3-trimethylbutan-2-yl (*R*)-2-(4-(dimethylcarbamoyl)phenyl)propanoate (2g)

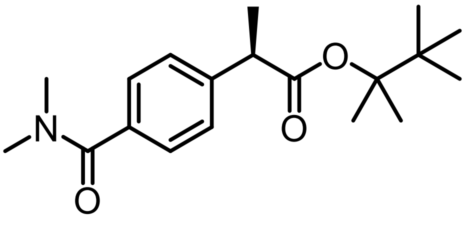

A colorless oil (41.5 mg, 65% yield, 90% ee) from 4-bromo-N,N-dimethylbenzamide (46.0 mg, 0.2 mmol).

^{1}H NMR (400 MHz, $CDCl_3$) δ 7.36 – 7.32 (m, 2H), 7.28 – 7.26 (m, 2H), 3.63 (q, J = 7.2 Hz, 1H), 3.00 (s, 6H), 1.43 (d, J = 7.2 Hz, 3H), 1.44 (s, 3H), 1.36 (s, 3H), 0.80 (s, 9H).

^{13}C NMR (101 MHz, $CDCl_3$) δ 173.1, 171.4, 142.8, 134.7, 127.4, 127.3, 87.3, 46.8, 38.3, 24.9, 20.4, 20.1, 17.9.

IR(neat): 2975, 1721, 1631, 1376, 1264, 1127, 1019, 920, 787, 729 cm^{-1}.

HRMS (ESI) calcd for $C_{19}H_{30}O_3N_1$ $[M+H]^+$: 320.2220, found: 320.2218.

Enantioselective ratio = 95.0:5.0, determined by HPLC (Daicel Chiralpak IC-H Column, *n*-Hexane: *i*-PrOH = 95.0:5.0, flow rate 1.0 mL/min, T = 25 °C, λ = 214 nm): t_R = 14.51 min (major), t_R = 17.70 min (minor).

$[\alpha]_D^{26.6}$ = -10.73 (c = 0.4, $CHCl_3$).

2-(methylthio)ethyl (*R*)-4-(1-oxo-1-((2,3,3-trimethylbutan-2-yl)oxy)propan-2-yl)benzoate (2h)

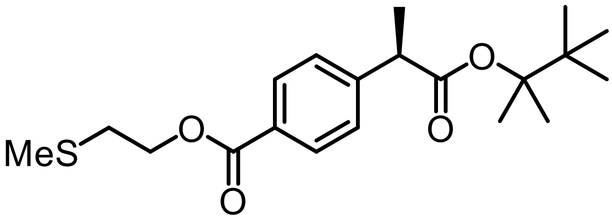

A colorless oil (57.1 mg, 78% yield, 90% ee) from 2-(methylthio)ethyl 4-bromobenzoate (57.0 mg, 0.2 mmol).

^{1}H NMR (400 MHz, $CDCl_3$) δ 8.00 – 7.96 (m, 2H), 7.36 – 7.32 (m, 2H), 4.47 (t, *J* = 7.0 Hz, 2H), 3.68 (q, *J* = 7.2 Hz, 1H), 2.85 (t, *J* = 6.8 Hz, 2H), 2.19 (s, 3H), 1.46 (d, *J* = 7.2 Hz, 3H), 1.45 (s, 3H), 1.36 (s, 3H), 0.81 (s, 9H).

^{13}C NMR (101 MHz, $CDCl_3$) δ 172.81, 166.18, 146.62, 129.87, 128.55, 127.56, 87.57, 63.57, 46.98, 38.30, 32.62, 24.94, 20.41, 20.14, 17.89, 15.86.

IR (neat): 2975, 1718, 1610, 1461, 1375, 1268, 1176, 1103, 939, 847, 774 cm^{-1}.

HRMS (ESI) calcd for $C_{20}H_{30}O_4S_1Na_1$ $[M+Na]^+$: 389.1757, found: 389.1755.

Enantioselective ratio = 95:5, determined by HPLC (Daicel Chiralpak IC-H Column, *n*-Hexane: *i*-PrOH = 98.0:2.0, flow rate 1.0 mL/min, T = 25 °C, λ = 214 nm): t_R = 11.14 min (major), t_R = 11.81 min (minor).

$[\alpha]_D^{26.4}$ = -31.98 (c = 0.1, $CHCl_3$).

2,3,3-trimethylbutan-2-yl (*R*)-2-(3-methoxy-4-methylphenyl)propanoate (2i)

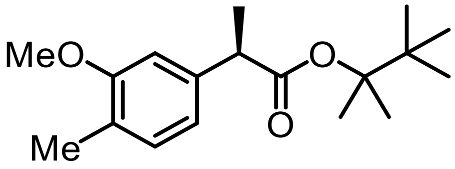

A colorless oil (36.0 mg, 65% yield, 84% ee) from 4-bromo-2-methoxy-1-methylbenzene (41.0 mg, 0.2 mmol).

^{1}H NMR (400 MHz, $CDCl_3$) δ 7.05 (d, *J* = 7.4 Hz, 1H), 6.77 (dd, *J* = 7.4, 1.6 Hz, 1H), 6.74 (d, *J* = 1.6 Hz, 1H), 3.82 (s, 3H), 3.59 (q, *J* = 7.2 Hz, 1H), 2.18 (s, 3H), 1.47 (s, 3H), 1.46 (d, *J* = 7.2 Hz, 3H), 1.40 (s, 3H), 0.87 (s, 9H).

^{13}C NMR (101 MHz, $CDCl_3$) δ 173.8, 157.6, 140.0, 130.5, 125.0, 119.3, 109.1, 87.0, 55.2, 47.0, 38.4, 25.0, 20.5, 20.2, 18.2, 15.9.

IR (neat): 2973, 1723, 1613, 1509, 1414, 1329, 1176, 1041, 938, 848, 728 cm^{-1}.

HRMS (ESI) calcd for $C_{18}H_{28}O_3Na_1$ $[M+Na]^+$: 315.1931, found: 315.1930.

Enantioselective ratio = 8.0:92.0, determined by HPLC (Daicel Chiralpak OJ-H Column, *n*-Hexane: *i*-PrOH = 99.5:0.5, flow rate 0.5 mL/min, T = 25 °C, λ = 214 nm): t_R = 12.16 min (minor), t_R = 17.47 min (major).

$[\alpha]_D^{26.5}$ = -5.47 (c = 1.0, $CHCl_3$).

2,3,3-trimethylbutan-2-yl (*R*)-2-(4-(tert-butyl)phenyl)propanoate (2j)

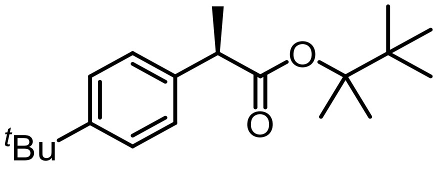

A colorless oil (43.2 mg, 71% yield, 87% ee) from 1-bromo-4-tert-butylbenzene (43.0 mg, 0.2 mmol).

^{1}H NMR (400 MHz, $CDCl_3$) δ 7.35 – 7.30 (m, 2H), 7.22 – 7.18 (m, 2H), 3.62 (q, *J* = 7.2 Hz, 1H), 1.47 (d, *J* = 7.2 Hz, 3H), 1.47 (s, 3H), 1.40 (s, 3H), 1.31 (s, 9H), 0.85 (s, 9H).

^{13}C NMR (101 MHz, CDCl$_3$) δ 173.9, 149.6, 138.2, 127.1, 125.3, 87.0, 46.5, 38.3, 34.4, 31.3, 25.0, 20.4, 20.2, 18.1.

Enantioselective ratio = 94:6, determined by HPLC (Daicel Chiralpak OJ-H Column, *n*-Hexane: *i*-PrOH = 99.5:0.5, flow rate 0.5 mL/min, T = 25 °C, λ = 214 nm): t_R = 7.30 min (major), t_R = 8.70 min (minor).

$[\alpha]_D^{25.6}$ = -3.89 (c = 1.0, CHCl$_3$).

All the resonances in ^{1}H NMR spectrum were consistent with the reported values.[2]

^{1}H and ^{13}C NMR spectra are provided below.

2,3,3-trimethylbutan-2-yl (*R*)-2-([1,1'-biphenyl]-4-yl)propanoate (2k)

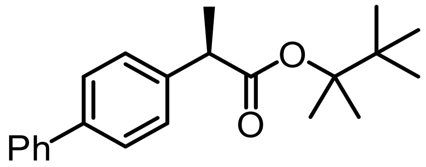

A white solid (53.1 mg, 82% yield, 86% ee) from 4-bromobiphenyl (47.0 mg, 0.2 mmol).

^{1}H NMR (400 MHz, CDCl$_3$) δ 7.62 – 7.57 (m, 4H), 7.46 – 7.42 (m, 2H), 7.38 – 7.32 (m, 3H), 3.70 (q, *J* = 7.2 Hz, 1H), 1.53 (d, *J* = 7.2 Hz, 3H), 1.50 (s, 3H), 1.43 (s, 3H), 0.88 (s, 9H).

^{13}C NMR (101 MHz, CDCl$_3$) δ 173.6, 140.8, 140.3, 139.7, 128.7, 127.9, 127.1, 127.0, 87.2, 46.6, 38.4, 25.0, 20.5, 20.2, 18.1.

Enantioselective ratio = 92.7:7.3, determined by HPLC (Daicel Chiralpak IC-H Column, *n*-Hexane: *i*-PrOH = 99:1, flow rate 1.0 mL/min, T = 25 °C, λ = 214 nm): t_R = 5.14 min (major), t_R = 5.44 min (minor).

$[\alpha]_D^{30.0}$ = -28.66 (c = 0.2, CHCl$_3$).

All the resonances in ^{1}H NMR spectrum were consistent with the reported values.[2]

^{1}H and ^{13}C NMR spectra are provided below.

2,3,3-trimethylbutan-2-yl (*R*)-2-(4-(methylthio)phenyl)propanoate (2l)

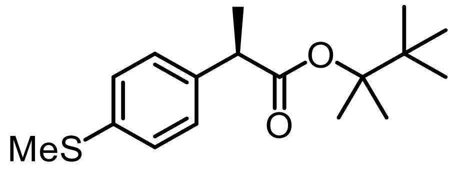

A colorless oil (37.4 mg, 75% yield, 89% ee) from 4-bromothioanisole (41.0 mg, 0.2 mmol).

^{1}H NMR (400 MHz, CDCl$_3$) δ 7.20 – 7.18 (m, 4H), 3.59 (q, J = 7.2 Hz, 1H), 2.46 (s, 3H), 1.46 (s, 3H), 1.44 (d, J = 7.2 Hz, 3H), 1.38 (s, 3H), 0.85 (s, 9H).

^{13}C NMR (101 MHz, CDCl$_3$) δ 173.5, 138.2, 136.6, 127.9, 126.9, 87.2, 46.4, 38.3, 25.0, 20.4, 20.2, 18.0, 16.0.

IR (neat): 2975, 1722, 1493, 1463, 1373, 1205, 1172, 1095, 938, 898, 786 cm^{-1}.

HRMS (ESI) calcd for $C_{17}H_{26}O_2S_1Na_1$ [M+Na]$^+$: 317.1546, found: 317.1544.

Enantioselective ratio = 94.5:5.5, determined by HPLC (Daicel Chiralpak IC-H Column, *n*-Hexane: *i*-PrOH = 98.0:2.0, flow rate 0.7 mL/min, T = 25 °C, λ = 214 nm): t_R = 6.89 min (major), t_R = 7.13 min (minor).

$[\alpha]_D^{26.4}$ = -8.01 (c = 1.0, CHCl$_3$).

2,3,3-trimethylbutan-2-yl (*R*)-2-(4-((trifluoromethyl)thio)phenyl)propanoate(2m)

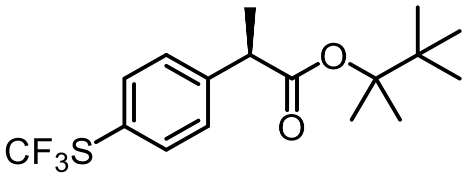

A colorless oil (48.7 mg, 70% yield, 92% ee) from 1-bromo-4-(trifluoromethylthio)benzene (52.0 mg, 0.2 mmol).

^{1}H NMR (400 MHz, CDCl$_3$) δ 7.60 (d, *J* = 8.2 Hz, 2H), 7.32 (d, *J* = 8.2 Hz, 2H), 3.67 (q, *J* = 7.2 Hz, 1H), 1.48 (d, *J* = 7.2 Hz, 3H), 1.46 (s, 3H), 1.38 (s, 3H), 0.80 (s, 9H).

^{19}F NMR (376 MHz, CDCl$_3$) δ -43.0 (s, 3F).

^{13}C NMR (101 MHz, CDCl$_3$) δ 172.8, 144.5, 136.5, 129.6 (q, *J* = 309.2 Hz), 128.7, 122.6 (q, *J* = 2.2 Hz), 87.7, 46.7, 38.3, 24.9, 20.4, 20.2, 17.8.

IR (neat): 2977, 1725, 1465, 1376, 1113, 1085, 1015, 845, 786 cm^{-1}.

HRMS (ESI) calcd for $C_{17}H_{23}O_2F_3S_1$ [M]$^+$: 348.1365, found: 348.1372.

Enantioselective ratio = 4.0:96.0, determined by HPLC (Daicel Chiralpak IC-H Column, *n*-Hexane: *i*-PrOH = 100:0, flow rate 0.4 mL/min, T = 25 °C, λ = 214 nm): t_R = 36.63 min (minor), t_R = 37.95 min (major).

[α]D$^{29.3}$ = -4.90 (c = 0.2, $CHCl_3$).

2,3,3-trimethylbutan-2-yl (*R*)-2-(4-(phenylsulfonyl)phenyl)propanoate (2n)

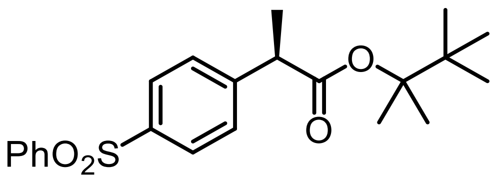

A white solid (55.0 mg, 71% yield, 91% ee) from Phenyl 4-bromophenyl sulfone (60.0 mg, 0.2 mmol). **M. p. :** 54.8 – 65.0 °C

^{1}H NMR (400 MHz, CDCl3) δ 7.95 – 7.82 (m, 4H), 7.56 – 7.52 (m, 1H), 7.50 – 7.45 (m, 2H), 7.42 – 7.36 (m, 2H), 3.67 (q, *J* = 7.2 Hz, 1H), 1.45 (d, *J* = 7.2 Hz, 3H), 1.43 (s, 3H), 1.35 (s, 3H), 0.76 (s, 9H).

^{13}C NMR (101 MHz, CDCl3) δ 172.4, 147.0, 141.7, 139.9, 133.1, 129.2, 128.5, 127.9, 127.5, 87.9, 46.8, 38.3, 24.9, 20.4, 20.2, 17.9.

IR (neat): 2974, 1719, 1596, 1447, 1373, 1210, 1153, 1107, 1068, 930, 839, 754 cm^{-1}.

HRMS (ESI) calcd for $C_{22}H_{28}O_4Na_1S_1$ $[M+Na]^+$: 411.1600, found: 411.1601.

Enantioselective ratio = 95.5:4.5, determined by HPLC (Daicel Chiralpak AD-H Column, *n*-Hexane: *i*-PrOH = 98:2, flow rate 1.0 mL/min, T = 25 °C, λ = 254 nm): t_R = 17.98 min (major), t_R = 21.78 min (minor).

[α]D$^{27.8}$ = -13.6 (c = 0.25, $CHCl_3$).

2,3,3-trimethylbutan-2-yl (*R*)-2-(4-cyanophenyl)propanoate (2o)

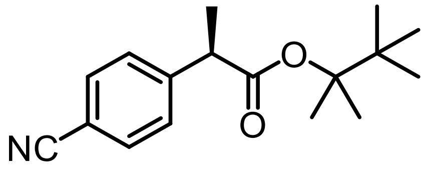

A colorless oil (41.0 mg, 75% yield, 92% ee) from 4-bromobenzonitrile (38.0 mg, 0.2 mmol).

^{1}H NMR (400 MHz, CDCl3) δ 7.60 (d, *J* = 8.2 Hz, 2H), 7.37 (d, *J* = 8.2 Hz, 2H), 3.68 (q, *J* = 7.2 Hz, 1H), 1.47 (d, *J* = 7.2 Hz, 3H), 1.45 (s, 3H), 1.37 (s, 3H), 0.81 (s, 9H).

^{13}C NMR (101 MHz, CDCl3) δ 172.3, 146.6, 132.3, 128.4, 118.8, 110.8, 88.0, 47.0, 38.3, 24.9, 20.4, 20.2, 17.8.

Enantioselective ratio = 96:4, determined by HPLC (Daicel Chiralpak AD-H Column, *n*-Hexane: *i*-PrOH = 98.0:2.0, flow rate 0.5 mL/min, T = 25 °C, λ = 250 nm): t_R = 12.68 min (major), t_R = 13.66 min (minor).

[α]$_D^{24}$ = -16.69 (c = 1.5, $CHCl_3$).

All the resonances in ^{1}H NMR spectrum were consistent with the reported values.[2]

^{1}H and ^{13}C NMR spectra are provided below.

2,3,3-trimethylbutan-2-yl (*R*)-2-(4-((tert-butoxycarbonyl)amino)phenyl)propanoate (2p)

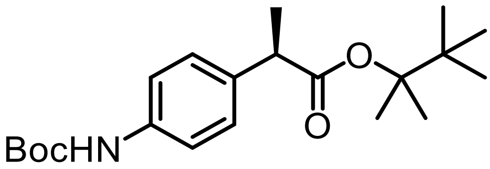

A colorless oil (30.0 mg, 42% yield, 85% ee) from tert-butyl N-(4-bromophenyl)-carbamate (55.0 mg, 0.2 mmol).

^{1}H NMR (400 MHz, $CDCl_3$) δ 7.29 (d, J = 8.2 Hz, 2H), 7.21 – 7.16 (m, 2H), 6.49 (s, 1H), 3.57 (q, J = 7.2 Hz, 1H), 1.51 (s, 9H), 1.45 (s, 3H), 1.42 (d, J = 7.2 Hz, 3H), 1.37 (s, 3H), 0.85 (s, 9H).

^{13}C NMR (101 MHz, $CDCl_3$) δ 173.7, 152.7, 137.0, 135.9, 128.0, 87.1, 46.3, 38.3, 28.3, 25.0, 20.4, 20.2, 18.1.

IR(neat): 2976, 1703, 1596, 1523, 1367, 1231, 1158, 1127, 1051, 839, 772 cm^{-1}.

HRMS (ESI) calcd for $C_{21}H_{33}O_4Na_1$ [M+Na]$^+$: 386.2302, found: 386.2300.

Enantioselective ratio = 92.5:7.5, determined by HPLC (Daicel Chiralpak AD-H Column, *n*-Hexane: *i*-PrOH = 98.0:2.0, flow rate 1.0 mL/min, T = 25 °C, λ = 220 nm): t_R = 11.74 min (major), t_R = 13.80 min (minor).

[α]$_D^{26.5}$ = -2.31 (c = 0.1, $CHCl_3$).

2,3,3-trimethylbutan-2-yl (*R*)-2-(4-acetamidophenyl)propanoate (2q)

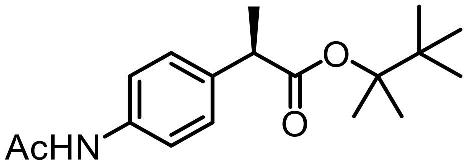

A colorless oil (39.7 mg, 65% yield, 85% ee) from 4-bromoacetanilide (43.0 mg, 0.2 mmol).

^{1}H NMR (400 MHz, $CDCl_3$) δ 7.58 (s, 1H), 7.43 (d, J = 8.4 Hz, 2H), 7.20 (d, J = 8.4 Hz, 2H), 3.59 (q, J = 7.2 Hz, 1H), 2.14 (s, 3H), 1.45 (s, 3H), 1.43 (d, J = 7.2 Hz, 3H), 1.37 (s, 3H), 0.85 (s, 9H).

^{13}C NMR (101 MHz, $CDCl_3$) δ 173.7, 168.4, 137.0, 136.7, 127.9, 119.9, 87.2, 46.4, 38.3, 25.0, 24.5, 20.4, 20.2, 18.1.

IR(neat): 2973, 1722, 1667, 1513, 1316, 1173, 1126, 1003, 841, 784, 732 cm^{-1}.

HRMS (ESI) calcd for $C_{18}H_{27}O_3N_1Na_1$ $[M+Na]^+$: 328.1883, found: 328.1881.

Enantioselective ratio = 92.5:7.5, determined by HPLC (Daicel Chiralpak AD-H Column, *n*-Hexane: *i*-PrOH = 98.0:2.0, flow rate 0.6 mL/min, T = 25 °C, λ = 254 nm): t_R = 36.22 min (major), t_R = 29.97 min (minor).

$[\alpha]_D^{26.5}$ = -7.63 (c = 0.6, $CHCl_3$).

2,3,3-trimethylbutan-2-yl (*R*)-2-(4-(2-oxopyrrolidin-1-yl)phenyl)propanoate (2r)

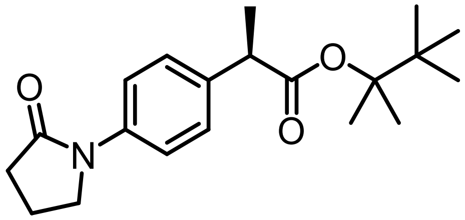

A colorless oil (47.0 mg, 71% yield, 84% ee) from 1-(4-bromophenyl)-2-pyrrolidinone (48.0 mg, 0.2 mmol).

^{1}H NMR (400 MHz, $CDCl_3$) δ 7.57 (d, *J* = 8.8 Hz, 2H), 7.30 – 7.26 (m, 2H), 3.85 (t, *J* = 7.0 Hz, 2H), 3.62 (q, *J* = 7.2 Hz, 1H), 2.60 (t, *J* = 8.0 Hz, 2H), 2.19 – 2.11 (m, 2H), 1.46 (s, 3H), 1.45 (d, *J* = 7.2 Hz, 3H), 1.39 (s, 3H), 0.86 (s, 9H).

^{13}C NMR (101 MHz, $CDCl_3$) δ 174.1, 173.6, 138.1, 137.3, 127.8, 119.8, 87.2, 48.7, 46.4, 38.3, 32.7, 25.0, 20.4, 20.2, 18.2, 18.0.

IR (neat): 2963, 1718, 1690, 1460, 1372, 1300, 1171, 1080, 928, 848, 766, 698 cm^{-1}.

HRMS (ESI) calcd for $C_{20}H_{29}O_3N_1Na_1$ $[M+Na]^+$: 354.2040, found: 354.2038.

Enantioselective ratio = 92:8, determined by HPLC (Daicel Chiralpak AD-H Column, *n*-Hexane: *i*-PrOH = 90:10, flow rate 1.0 mL/min, T = 25 °C, λ = 214 nm): t_R = 9.00 min (major), t_R = 10.39 min (minor).

[α]$_D^{25.8}$ = -1.34 (c = 0.1, $CHCl_3$).

2,3,3-trimethylbutan-2-yl (*R*)-2-(4-((trimethylsilyl)ethynyl)phenyl)propanoate (2s)

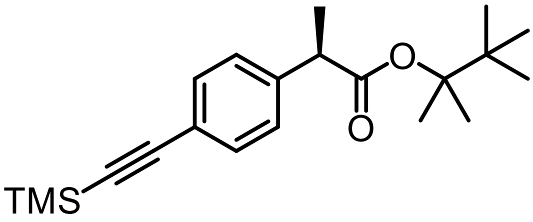

A colorless oil (44.0 mg, 64% yield, 87% ee) from (4-bromophenylethynyl)trimethylsilane (51.0 mg, 0.2 mmol).

^{1}H NMR (400 MHz, $CDCl_3$) δ 7.42 – 7.39 (m, 2H), 7.22 – 7.19 (m, 2H), 3.61 (q, *J* = 7.2 Hz, 1H), 1.45 (s, 3H), 1.44 (d, *J* = 7.2 Hz, 3H), 1.36 (s, 3H), 0.82 (s, 9H), 0.24 (s, 9H).

^{13}C NMR (101 MHz, $CDCl_3$) δ 173.2, 141.7, 132.1, 127.3, 121.5, 105.0, 93.9, 87.3, 46.9, 38.3, 25.0, 20.4, 20.1, 17.8, -0.1.

IR (neat): 2959, 2157, 1725, 1463, 1375, 1249, 1173, 1127, 1071, 862, 840, 759 cm^{-1}.

HRMS (ESI) calcd for $C_{21}H_{32}O_2Si_1Na_1$ $[M+Na]^+$: 367.2064, found: 367.2065.

Enantioselective ratio = 93.5:6.5, determined by HPLC (Daicel Chiralpak IG-H Column, *n*-Hexane: *i*-PrOH = 99.5:0.5, flow rate 0.3 mL/min, T = 25 °C, λ = 214 nm): t_R = 13.16 min (major), t_R = 13.73 min (minor).

[α]$_D^{25.5}$ = -5.38 (c = 0.1, $CHCl_3$).

2,3,3-trimethylbutan-2-yl (*R*)-2-(4-(4,4,5,5-tetramethyl-1,3,2-dioxaborolan-2-yl)phenyl)propanoate (2t)

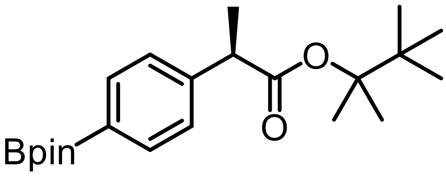

A colorless oil (32.6 mg, 66% yield, 90% ee) from 2-(4-bromo-phenyl)-4,4,5,5-tetramethyl-[1,3,2]dioxaborolane (58.0 mg, 0.2 mmol).

^{1}H NMR (400 MHz, CDCl$_3$) δ 7.75 (d, *J* = 8.0 Hz, 2H), 7.28 (d, *J* = 8.0 Hz, 2H), 3.64 (q, *J* = 7.2 Hz, 1H), 1.46(s, 3H), 1.45 (d, *J* = 7.0 Hz, 3H), 1.37 (s, 3H), 1.34 (s, 12H), 0.83 (s, 9H).

^{13}C NMR (101 MHz, CDCl$_3$) δ 173.4, 144.5, 135.0, 126.9, 87.2, 83.7, 47.2, 38.3, 25.0, 24.9, 24.8, 20.5, 20.2, 18.0.

Enantioselective ratio = 95.0:5.0, determined by HPLC (Daicel Chiralpak IC-H Column, *n*-Hexane: *i*-PrOH = 98.0:2.0, flow rate 0.5 mL/min, T = 25 °C, λ = 214 nm): t_R = 8.49 min (major), t_R = 8.82 min (minor).

$[\alpha]_D^{25.3}$ = -1.38 (c = 0.25, CHCl$_3$).

All the resonances in ^{1}H NMR spectrum were consistent with the reported values.[2]

^{1}H and ^{13}C NMR spectra are provided below.

2,3,3-trimethylbutan-2-yl (*R*)-2-(4-oxochroman-7-yl)propanoate (2u)

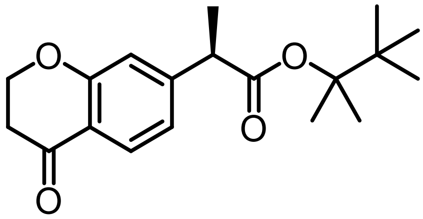

A colorless oil (45.2 mg, 71% yield, 91% ee) from 7-bromo-4-chromanone (46.0 mg, 0.2 mmol).

^{1}H NMR (400 MHz, CDCl$_3$) δ 7.83 (d, *J* = 8.0 Hz, 1H), 6.92 (dd, *J* = 8.0, 1.6 Hz, 1H), 6.88 (d, *J* = 1.6 Hz, 1H), 4.51 (dd, *J* = 6.8, 6.0 Hz, 2H), 3.62 (q, *J* = 7.2 Hz, 1H), 2.78 (dd, *J* = 6.8, 6.0 Hz, 2H), 1.46 (s, 3H), 1.45 (d, *J* = 7.2 Hz, 3H), 1.39 (s, 3H), 0.84 (s, 9H).

^{13}C NMR (101 MHz, CDCl$_3$) δ 191.5, 172.5, 161.9, 150.0, 127.3, 121.0, 120.0, 116.7, 87.8, 67.1, 47.1, 38.3, 37.7, 25.1, 25.1, 25.0, 20.4, 20.2, 17.7.

IR (neat): 2975, 1722, 1691, 1569, 1432, 1254, 1126, 1037, 939, 879, 789 cm^{-1}.

HRMS (ESI) calcd for $C_{19}H_{26}O_4Na_1$ [M+Na]$^+$: 341.1723, found: 341.1721.

Enantioselective ratio = 95.5:4.5, determined by HPLC (Daicel Chiralpak AD-H Column, *n*-Hexane: *i*-PrOH = 98.0:2.0, flow rate 1.0 mL/min, T = 25 °C, λ = 220 nm): t_R = 8.23 min (major), t_R = 8.99 min (minor).

[α]$_D$$^{26.7}$ = -5.24 (c = 1.0, CHCl$_3$).

Ethyl (*R*)-5-(1-oxo-1-((2,3,3-trimethylbutan-2-yl)oxy)propan-2-yl)benzofuran-2-carboxylate (2v)

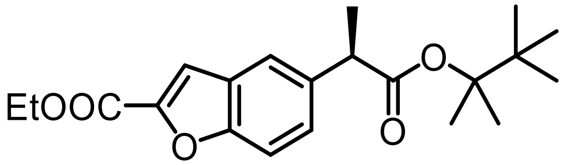

A colorless oil (53.0 mg, 74% yield, 88% ee) from ethyl(5-bromobenzofuran)-2-carboxylate (54.0 mg, 0.2 mmol).

^{1}H NMR (400 MHz, CDCl$_3$) δ 7.57 (d, *J* = 1.8 Hz, 1H), 7.51 (d, *J* = 8.8 Hz, 1H), 7.48 (s, 1H), 7.36 (dd, *J* = 8.8, 1.8 Hz, 1H), 4.49 – 4.35 (m, 2H), 3.73 (q, *J* = 7.2 Hz, 1H), 1.50 (d, *J* = 7.2 Hz, 3H), 1.45 (s, 3H), 1.41 (t, *J* = 7.1 Hz, 3H), 1.35 (s, 3H), 0.81 (s, 9H).

^{13}C NMR (101 MHz, CDCl$_3$) δ 173.6, 159.5, 154.8, 146.0, 137.1, 127.5, 127.1, 121.2, 113.7, 112.2, 87.3, 61.5, 46.7, 38.3, 24.9, 20.4, 20.2, 18.4, 14.3.

IR (neat): 2977, 1719, 1565, 1464, 1370, 1295, 1260, 1176, 1125, 1094, 1016, 944, 846, 765 cm^{-1}.

HRMS (ESI) calcd for $C_{21}H_{28}O_5Na_1$ [M+Na]$^+$: 383.1829, found: 383.1829.

Enantioselective ratio = 93.5:6.5, determined by HPLC (Daicel Chiralpak IC-H Column, *n*-Hexane: *i*-PrOH = 99.0:1.0, flow rate 1.0 mL/min, T = 25 °C, λ = 254 nm): t_R = 16.67 min (major), t_R = 17.60 min (minor).

[α]$_D$$^{30.2}$ = -8.61 (c = 0.1, CHCl$_3$).

2,3,3-trimethylbutan-2-yl (*R*)-2-(benzofuran-3-yl)propanoate (2w)

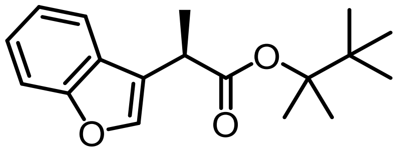

A colorless oil (38.1 mg, 61% yield, 80% ee) from 3-bromo-1-benzofuran (40.0 mg, 0.2 mmol).

^{1}H NMR (400 MHz, $CDCl_3$) δ 7.62 – 7.57 (m, 1H), 7.55 (s, 1H), 7.48 – 7.45 (m, 1H), 7.31 – 7.26 (m, 1H), 7.25 – 7.21 (m, 1H), 3.86 – 3.80 (m, 1H), 1.59 (d, J = 7.2 Hz, 3H), 1.47 (s, 3H), 1.44 (s, 3H), 0.82 (s, 9H).

^{13}C NMR (101 MHz, $CDCl_3$) δ 173.0, 155.3, 141.4, 127.0, 124.3, 122.4, 120.5, 120.0, 111.5, 87.6, 38.3, 37.5, 24.9, 20.34, 20.27, 17.1.

Enantioselective ratio = 90:10, determined by HPLC (Daicel Chiralpak IG-H Column, *n*-Hexane: *i*-PrOH = 99.5:0.5, flow rate 1.0 mL/min, T = 25 °C, λ = 214 nm): t_R = 6.84 min (minor), t_R = 7.33 min (major).

$[\alpha]_D^{27}$ = -6.31 (c = 0.7, $CHCl_3$).

All the resonances in ^{1}H NMR spectrum were consistent with the reported values.[2] ^{1}H and ^{13}C NMR spectra are provided below.

2,3,3-trimethylbutan-2-yl (*R*)-2-(dibenzo[*b,d*]thiophen-3-yl)propanoate (2x)

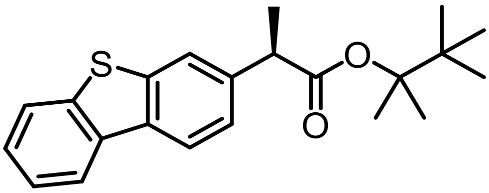

A colorless oil (55.3 mg, 78% yield, 89% ee) from 3-bromodibenzo[b,d]thiophene (53.0 mg, 0.2 mmol).

^{1}H NMR (400 MHz, $CDCl_3$) δ 8.15 – 8.11 (m, 1H), 8.10 (d, J = 8.2 Hz, 1H), 7.88 – 7.82 (m, 1H), 7.81 – 7.78 (m, 1H), 7.47 – 7.43 (m, 2H), 7.41 – 7.38 (m, 1H), 3.81 (q, J = 7.2 Hz, 1H), 1.58 (d, J = 7.2 Hz, 3H), 1.50 (s, 3H), 1.41 (s, 3H), 0.86 (s, 9H).

^{13}C NMR (101 MHz, $CDCl_3$) δ 173.4, 140.1, 139.7, 139.4, 135.3, 126.5, 124.3, 124.1, 122.7, 121.5, 121.5, 121.4, 87.4, 47.1, 38.3, 25.0, 20.5, 20.2, 18.2.

IR (neat): 2974, 1723, 1443, 1373, 1235, 1126, 1038, 846, 786, 748, 613 cm^{-1}.

HRMS (ESI) calcd for $C_{22}H_{26}O_2S_1Na_1$ [M+Na]$^+$: 377.1546, found: 377.1545.

Enantioselective ratio = 94.5:5.5, determined by HPLC (Daicel Chiralpak IC-H Column, *n*-Hexane: *i*-PrOH = 98.0:2.0, flow rate 1.0 mL/min, T = 25 °C, λ = 214 nm): t_R = 5.73 min (major), t_R = 6.05 min (minor).

[α]$_D^{26}$ = -8.21 (c = 0.2, $CHCl_3$).

2,3,3-trimethylbutan-2-yl (*R*)-2-(2-(methylthio)pyrimidin-5-yl)propanoate (2y)

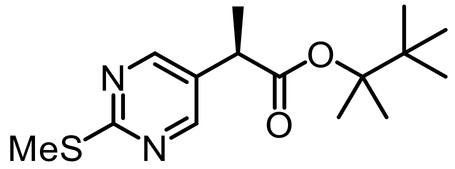

A colorless oil (38.5 mg, 65% yield, 86% ee) from 5-bromo-2-(methylthio)pyrimidine (43.0 mg, 0.2 mmol).

^{1}H NMR (400 MHz, $CDCl_3$) δ 8.43 (s, 2H), 3.54 (q, *J* = 7.2 Hz, 1H), 2.53 (s, 3H), 1.46 (d, *J* = 7.3 Hz, 3H), 1.45 (s, 3H), 1.39 (s, 3H), 0.86 (s, 9H).

^{13}C NMR (101 MHz, $CDCl_3$) δ 172.0, 171.2, 156.3, 129.0, 88.4, 42.0, 38.3, 25.0, 20.4, 20.3, 17.7, 14.0.

IR (neat): 2975, 1723, 1582, 1535, 1455, 1397, 1175, 1125, 937, 846, 776 cm^{-1}.

HRMS (ESI) calcd for $C_{15}H_{25}O_2N_2S_1$ [M+H]$^+$: 279.1631, found: 279.1630.

Enantioselective ratio = 7.0:93.0, determined by HPLC (Daicel Chiralpak IC-H Column, *n*-Hexane: *i*-PrOH = 98.0:2.0, flow rate 1.0 mL/min, T = 25 °C, λ = 254 nm): t_R = 24.53 min (minor), t_R = 28.06 min (major).

[α]$_D^{26.1}$ = 0.83 (c = 1.0, $CHCl_3$).

2,3,3-trimethylbutan-2-yl (*R*)-2-(9*H*-fluoren-2-yl)propanoate (2z)

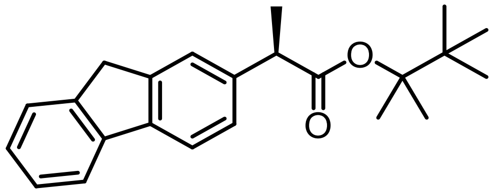

A white solid (60.0 mg, 90% yield, 86% ee) from 2-bromofluorene (50.0 mg, 0.2 mmol).

^{1}H NMR (400 MHz, $CDCl_3$) δ 7.79 – 7.75 (m, 1H), 7.73 – 7.71 (m, 1H), 7.55 – 7.52 (m, 1H), 7.47 (s, 1H), 7.37 (td, *J* = 7.5, 1.1 Hz, 1H), 7.39 – 7.35 (m, 1H), 7.31 – 7.26

(m, 2H), 3.88 (s, 2H), 3.71 (q, J = 7.2 Hz, 1H), 1.52 (d, J = 7.2 Hz, 3H), 1.48 (s, 3H), 1.39 (s, 3H), 0.85 (s, 9H).

^{13}C NMR (101 MHz, CDCl$_3$) δ 173.9, 143.5, 143.3, 141.5, 140.4, 139.9, 126.7, 126.5, 126.2, 125.0, 124.1, 119.8, 119.8, 87.1, 47.1, 38.4, 36.8, 25.0, 20.5, 20.2, 18.2.

Enantioselective ratio = 93.0:7.0, determined by HPLC (Daicel Chiralpak IC-H Column, *n*-Hexane: *i*-PrOH = 98:2, flow rate 1.0 mL/min, T = 25 °C, λ = 214 nm): t_R = 4.87 min (major), t_R = 5.22 min (minor).

$[\alpha]_D^{26.4}$ = -12.1 (c = 0.25, CHCl$_3$).

All the resonances in ^{1}H NMR spectrum were consistent with the reported values.[2]

^{1}H and ^{13}C NMR spectra are provided below.

2,3,3-trimethylbutan-2-yl (*R*,*E*)-4-(4-methoxyphenyl)-2-methylbut-3-enoate (2aa)

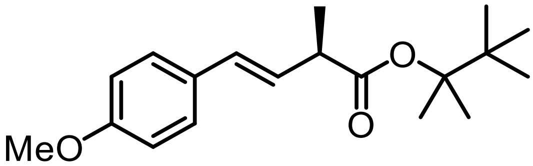

A colorless oil (34.0 mg, 56% yield, 55% ee) from (*E*)-1-(2-bromovinyl)-4-methoxybenzene (43.0 mg, 0.2 mmol).

^{1}H NMR (400 MHz, CDCl$_3$) δ 7.31 – 7.27 (m, 2H), 6.91 – 6.72 (m, 2H), 6.40 (d, J = 15.8 Hz, 1H), 6.12 (dd, J = 15.8, 8.0 Hz, 1H), 3.80 (s, 3H), 3.23 – 3.16 (m, 1H), 1.50 (s, 3H), 1.49 (s, 3H), 1.32 (d, J = 7.0 Hz, 3H), 0.98 (s, 9H).

^{13}C NMR (101 MHz, CDCl$_3$) δ 173.8, 159.0, 130.1, 129.9, 127.3, 127.3, 113.9, 87.1, 55.2, 44.8, 38.4, 25.1, 20.4, 20.4, 17.4.

IR (neat): 2975, 1722, 1505, 1375, 1235, 1169, 1127, 1071, 938, 898, 867, 787 cm^{-1}.

HRMS (ESI) calcd for $C_{19}H_{28}O_3Na_1$ [M+Na]$^+$: 327.1932, found: 327.1931.

Enantioselective ratio = 77.5 : 22.5, determined by HPLC (Daicel Chiralpak OJ-H Column, *n*-Hexane: *i*-PrOH = 98:2, flow rate 0.4 mL/min, T = 25 °C, λ = 214 nm): t_R = 15.850 min (major), t_R = 17.462 min (minor).

$[\alpha]_D^{26.2}$ = 3.7 (c = 1.0, CHCl$_3$).

(*R*)-2,5,7,8-tetramethyl-2-((4*R*,8*R*)-4,8,12-trimethyltridecyl)chroman-6-yl 4-((*R*)-1-oxo-1-((2,3,3-trimethylbutan-2-yl)oxy)propan-2-yl)benzoate (2ab)

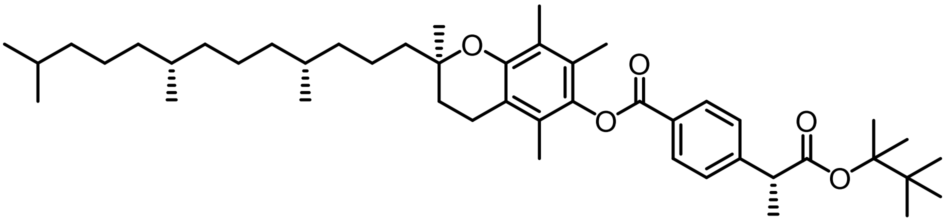

A colorless oil (98.7 mg, 70% yield, 89% dr) from (*R*)-2,5,7,8-tetramethyl-2-((4*R*,8*R*)-4,8,12-trimethyltridecyl)chroman-6-yl 4-bromobenzoate (123.0 mg, 0.2 mmol).

^{1}H NMR (400 MHz, $CDCl_3$) δ 8.23 – 8.20 (m, 2H), 7.46 – 7.43 (m, 2H), 3.76 (q, *J* = 7.2 Hz, 1H), 2.63 (t, *J* = 6.8 Hz, 1H), 2.14 (s, 3H), 2.07 (s, 3H), 2.03 (s, 3H), 1.91 – 1.75 (m, 2H), 1.60 – 1.58 (m, 6H), 1.54 (d, *J* = 7.2 Hz, 3H), 1.51 (s, 3H), 1.44 (s, 3H), 1.48 – 1.36 (m, 4H), 1.38 – 1.20 (m, 11H), 1.19 – 1.03 (m, 6H), 0.89 (s, 9H), 0.91 – 0.85 (m, 12H).

^{13}C NMR (101 MHz, $CDCl_3$) δ 172.8, 165.0, 149.4, 147.0, 140.6, 130.4, 128.2, 127.7, 126.9, 125.1, 123.1, 117.4, 87.7, 75.0, 47.1, 40.4, 39.6, 39.3, 38.3, 37.4, 37.3, 32.8, 32.7, 32.6, 31.2, 31.0, 27.9, 25.0, 24.8, 24.4, 24.2, 23.7, 22.7, 22.6, 21.0, 20.6, 20.4, 20.2, 19.7, 19.6, 18.0, 13.0, 12.1, 11.8.

IR (neat): 2925, 1729, 1460, 1376, 1270, 1176, 1129, 1091, 938, 850, 735 cm^{-1}.

HRMS (ESI) calcd for $C_{46}H_{72}O_5Na_1$ $[M+Na]^+$: 727.5272, found: 727.5276.

Enantioselective ratio = 94.5:5.5, determined by HPLC (Daicel Chiralpak IG-H Column, *n*-Hexane: *i*-PrOH = 98.0:2.0, flow rate 0.4 mL/min, T = 25 °C, λ = 214 nm): t_R = 13.16 min (major), t_R = 14.20 min (minor).

$[\alpha]_D^{27}$ = 0.94 (c = 1.0, $CHCl_3$).

2,3,3-trimethylbutan-2-yl (*R*)-2-(3-((5-(4-fluorophenyl)thiophen-2-yl)methyl)-4-methylphenyl)propanoate (2ac)

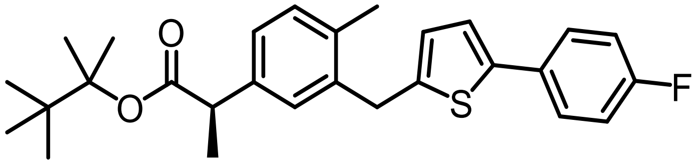

A colorless oil (68.5 mg, 76% yield, 86% ee) from 2-(5-bromo-2-Methylbenzyl)-5-(4-fluorophenyl)thiophene (72.0 mg, 0.2 mmol).

^{1}H NMR (400 MHz, CDCl$_3$) δ 7.51 – 7.44 (m, 2H), 7.19 – 7.10 (m, 3H), 7.06 – 7.00 (m, 3H), 6.65 – 6.63 (m, 1H), 4.12 (s, 2H), 3.62 (q, *J* = 7.2 Hz, 1H), 2.30 (s, 3H), 1.48 (s, 3H), 1.47 (d, *J* = 7.0 Hz, 3H), 1.40 (s, 3H), 0.86 (s, 9H).

^{19}F NMR (377 MHz, CDCl$_3$) δ -115.23 – -115.11 (m, 1F).

^{13}C NMR (101 MHz, CDCl$_3$) δ 173.7, 139.2, 138.1, 134.8, 130.6, 128.6, 127.1, 127.0, 125.9, 125.8, 122.6, 122.5, 115.7, 115.5, 87.0, 46.5, 38.3, 34.1, 25.0, 20.4, 20.2, 19.0, 18.2.

IR (neat): 2973, 1721, 1508, 1464, 1374, 1230, 1128, 1013, 891, 831, 798, 733 cm^{-1}.

HRMS (ESI) calcd for $C_{28}H_{33}O_2F_1S_1Na_1$ [M+Na]$^+$: 475.2078, found: 475.2078.

Enantioselective ratio = 7:93, determined by HPLC (Daicel Chiralpak IG-H Column, *n*-Hexane: *i*-PrOH = 98.0:2.0, flow rate 0.4 mL/min, T = 25 °C, λ = 214 nm): t_R = 17.38 min (major), t_R = 18.67 min (minor).

[α]$_D$$^{25.4}$ = -12.10 (c = 0.1, CHCl$_3$)

2,3,3-trimethylbutan-2-yl (*R*)-2-(4-(*N*-(2,6-dimethoxypyrimidin-4-yl)-*N*-methylsulfamoyl)phenyl)propanoate (2ad)

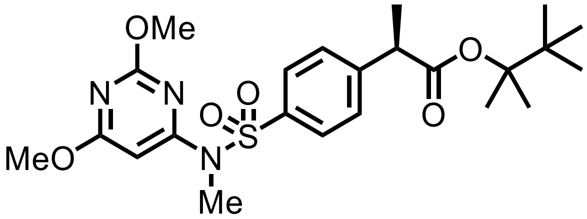

A colorless oil (77.5 mg, 81% yield, 91% ee) from 4-bromo-*N*-(2,6-dimethoxypyrimidin-4-yl)-*N*-methylbenzenesulfonamide (78.0 mg, 0.2 mmol).

^{1}H NMR (400 MHz, CDCl$_3$) δ 7.74 – 7.69 (m, 2H), 7.37 – 7.33 (m, 2H), 6.60 (s, 1H), 3.89 (s, 3H), 3.82 (s, 3H), 3.66 (q, *J* = 7.2 Hz, 1H), 3.42 (s, 3H), 1.43 (d, *J* = 7.2 Hz, 3H), 1.41 (s, 3H), 1.31 (s, 3H), 0.73 (s, 9H).

^{13}C NMR (101 MHz, CDCl$_3$) δ 172.5, 172.2, 164.2, 161.2, 147.1, 136.9, 128.3, 127.3, 89.6, 87.9, 54.6, 54.0, 46.8, 38.2, 34.3, 24.8, 20.3, 20.1, 17.7.

IR (neat): 2950, 1718, 1565, 1452, 1349, 1277, 1127, 1091, 1039, 967, 774, 594 cm^{-1}.

HRMS (ESI) calcd for $C_{23}H_{34}O_6N_3S_1$ $[M+H]^+$: 480.2163, found: 480.2162.

Enantioselective ratio = 95.5:4.5, determined by HPLC (Daicel Chiralpak IC-H Column, *n*-Hexane: *i*-PrOH = 90.0:10.0, flow rate 1.0 mL/min, T = 25 °C, λ = 254 nm): t_R = 23.76 min (major), t_R = 25.4 min (minor).

$[\alpha]_D^{25.6}$ = -2.89 (c = 0.1, $CHCl_3$).

2,3,3-trimethylbutan-2-yl (*R*)-2-(4-(5-methoxy-3-(2-methoxy-2-oxoethyl)-2-methyl-1*H*-indole-1-carbonyl)phenyl)propanoate (2ae)

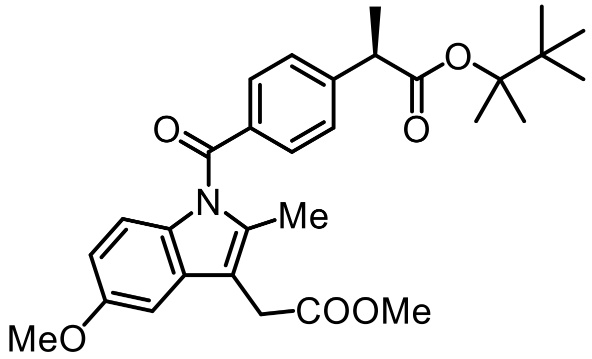

A colorless oil (76.0 mg, 75% yield, 92% ee) from 2-(1-(4-bromobenzoyl)-5-methoxy-2- methyl-1*H*-indol-3-yl)acetate (83.4 mg, 0.2 mmol).

^{1}H NMR (400 MHz, $CDCl_3$) δ 7.70 – 7.66 (m, 2H), 7.40 – 7.37 (m, 2H), 6.95 (d, *J* = 2.4 Hz, 1H), 6.86 (d, *J* = 9.0 Hz, 1H), 6.62 (dd, *J* = 9.0, 2.4 Hz, 1H), 3.83 (s, 3H), 3.73 (q, *J* = 7.2 Hz, 1H), 3.70 (s, 3H), 3.67 (s, 2H), 2.37 (s, 3H), 1.50 (d, *J* = 7.2 Hz, 3H), 1.48 (s, 3H), 1.40 (s, 3H), 0.85 (s, 9H).

^{13}C NMR (101 MHz, $CDCl_3$) δ 172.7, 171.4, 155.8, 146.6, 136.0, 134.0, 130.9, 130.5, 130.1, 127.8, 114.9, 112.1, 111.5, 101.0, 87.6, 55.6, 52.1, 47.0, 38.4, 30.1, 25.0, 20.4, 20.1, 17.8, 13.2.

IR (neat): 2959, 1731, 1686, 1475, 1371, 1263, 1178, 1127, 1066, 922, 846, 793 cm^{-1}.

HRMS (ESI) calcd for $C_{30}H_{38}O_6N_1$ $[M+H]^+$: 508.2694, found: 508.2698.

Enantioselective ratio = 96:4, determined by HPLC (Daicel Chiralpak AD-H Column, *n*-Hexane: *i*-PrOH = 90.0:10.0, flow rate 1.0 mL/min, T = 25 °C, λ = 250 nm): t_R = 11.91 min (major), t_R = 15.37 min (minor).

$[\alpha]_D^{26.5}$ = -8.86 (c =1.0, $CHCl_3$).

(1*R*,2*S*,5*R*)-2-isopropyl-5-methylcyclohexyl 4-((*R*)-1-oxo-1-((2,3,3-trimethylbutan-2-yl)oxy)propan-2-yl)benzoate (2af)

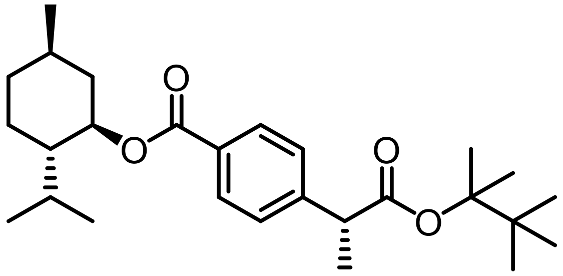

A colorless oil (64.5 mg, 75% yield, 92% dr) from (1*R*,2*S*,5*R*)-2-isopropyl-5-methylcyclohexyl 4-bromobenzoate (67.8 mg, 0.2 mmol).

^{1}H NMR (400 MHz, CDCl$_3$) δ 8.00 – 7.97 (m, 2H), 7.35 – 7.32 (m, 2H), 4.95 – 4.80 (m, 1H), 3.69 (q, *J* = 7.2 Hz, 1H), 2.14 – 2.07 (m, 1H), 1.98 – 1.88 (m, 1H), 1.75 – 1.69 (m, 2H), 1.60 – 1.50 (m, 2H), 1.47 (d, *J* = 7.2 Hz, 3H), 1.46 (s, 3H), 1.38 (s, 3H), 1.15 – 1.07 (m, 2H), 0.92 – 0.89 (m, 7H), 0.85 (s, 9H), 0.78 (d, *J* = 6.8 Hz, 3H).

^{13}C NMR (101 MHz, CDCl$_3$) δ 172.9&172.8, 165.9, 146.2&146.1, 129.8, 129.4, 127.4, 87.6, 74.7, 47.2, 47.0&46.9, 40.9, 38.3, 34.3, 31.4, 26.5, 25.0, 23.6, 22.0, 20.7, 20.4, 20.2&20.1, 18.1&18.0, 16.5.

IR (neat): 2956, 2871, 1712, 1610, 1455, 1371, 1269, 1176, 982, 962, 846, 746 cm^{-1}.

HRMS (ESI) calcd for $C_{27}H_{42}O_4Na_1$ $[M+H]^+$: 453.2975, found: 453.2974.

Enantioselective ratio = 96:4, determined by HPLC (Daicel Chiralpak IG-H Column, *n*-Hexane: *i*-PrOH = 98.0:2.0, flow rate 1.0 mL/min, T = 25 °C, λ = 214 nm): t_R = 7.24 min (major), t_R = 8.96 min (minor).

$[\alpha]_D^{26.4}$ = -43.14 (c = 1.125, $CHCl_3$).

(3a*R*,5*R*,6*S*,6a*R*)-5-((*R*)-2,2-dimethyl-1,3-dioxolan-4-yl)-2,2-dimethyltetrahydrofuro[2,3-*d*][1,3]dioxol-6-yl 4-((*R*)-1-oxo-1-((2,3,3-trimethylbutan-2-yl)oxy)propan-2-yl)benzoate (2ag)

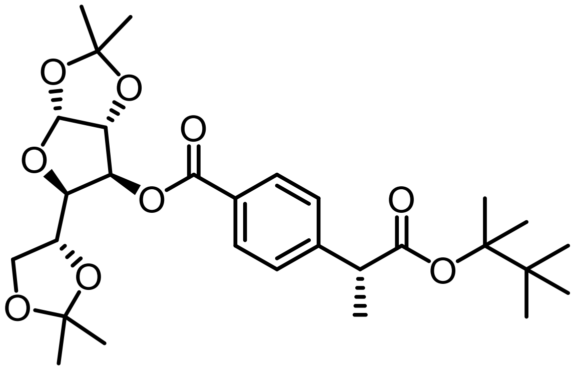

A colorless oil (74.8 mg, 70% yield, 90% dr) from (3a*R*,5*R*,6*S*,6a*R*)-5-((*R*)-2,2-dimethyl-1,3-dioxolan-4-yl)-2,2-dimethyltetrahydrofuro[2,3-*d*][1,3]dioxol6-yl 4-bromobenzoate (88.6 mg, 0.2 mmol).

^{1}H NMR (400 MHz, $CDCl_3$) δ 7.98 – 7.94 (m, 2H), 7.36 – 7.32 (m, 2H), 5.93 (d, *J* = 3.6 Hz, 1H), 5.48 (d, *J* = 2.6 Hz, 1H), 4.61 (d, *J* = 3.6 Hz, 1H), 4.37 – 4.29 (m, 2H), 4.12 – 4.04 (m, 2H), 3.69 (q, *J* = 7.2 Hz, 1H), 1.54 (s, 3H), 1.46 (d, *J* = 7.2 Hz, 3H), 1.45 (s, 3H), 1.39 (s, 3H), 1.36 (s, 3H), 1.30 (s, 3H), 1.24 (s, 3H), 0.82 (s, 9H).

^{13}C NMR (101 MHz, $CDCl_3$) δ 172.7&172.6, 164.9, 147.1&147.0, 130.0, 127.7, 112.2, 109.3, 105.0, 87.6, 83.3, 80.0, 76.5, 72.5, 67.1, 46.9&46.8, 38.3, 26.7, 26.6, 26.1, 25.1, 24.9, 20.4, 20.1&20.0, 17.9&17.8.

IR (neat): 2980, 1722, 1610, 1374, 1263, 1166, 1072, 1017, 846, 734, 702 cm^{-1}.

HRMS (ESI) calcd for $C_{29}H_{42}O_9Na_1$ $[M+Na]^+$: 557.2721, found: 557.2720.

Enantioselective ratio = 95:5, determined by HPLC (Daicel Chiralpak AD-H Column, *n*-Hexane: *i*-PrOH = 98.0:2.0, flow rate 0.7 mL/min, T = 25 °C, λ = 214 nm): t_R = 10.19 min (major), t_R = 11.62 min (minor).

$[\alpha]_D^{27.4}$ = -39.29 (c = 2.0, $CHCl_3$).

2,3,3-trimethylbutan-2-yl (*R*)-2-(6-methoxynaphthalen-2-yl)propanoate (2ah)

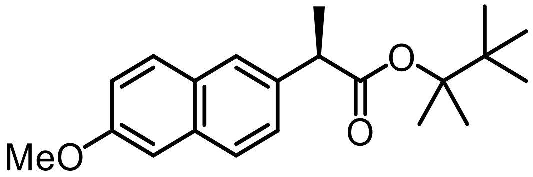

A colorless oil (39.0 mg, 60% yield, 87% ee) from 2-bromo-6-methoxynaphthalene (50.0 mg, 0.2 mmol).

^{1}H NMR (400 MHz, CDCl$_3$) δ 7.71 – 7.65 (m, 3H), 7.41 – 7.38 (m, 1H), 7.15 – 7.11 (m, 2H), 3.91 (s, 1H), 3.78 (q, J = 7.2 Hz, 1H), 1.56 (d, J = 7.2 Hz, 3H), 1.49 (s, 3H), 1.39 (s, 3H), 0.85 (s, 9H).

^{13}C NMR (101 MHz, CDCl$_3$) δ 173.8, 157.4, 136.4, 133.5, 129.2, 128.9, 126.9, 126.4, 125.8, 118.8, 118.8, 105.5, 87.1, 55.3, 46.9, 38.3, 25.0, 20.5, 20.2, 18.2.

Enantioselective ratio = 93.5:6.5, determined by HPLC (Daicel Chiralpak IC-H Column, *n*-Hexane: *i*-PrOH = 99.0:1.0, flow rate 0.5 mL/min, T = 25 °C, λ = 254 nm): t_R = 18.43 min (major), t_R = 19.7 min (minor).

$[\alpha]_D^{29.0}$ = -24.71 (c = 0.2, CHCl$_3$).

All the resonances in ^{1}H NMR spectrum were consistent with the reported values.[2] ^{1}H and ^{13}C NMR spectra are provided below.

2,3,3-trimethylbutan-2-yl (*R*)-2-(4-isobutylphenyl)propanoate (2ai)

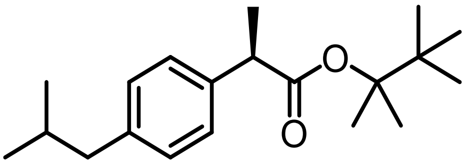

A colorless oil (39.5 mg, 65% yield, 86% ee) from 1-bromo-4-isobutylbenzene (43.0 mg, 0.2 mmol).

^{1}H NMR (400 MHz, CDCl$_3$) δ 7.18 – 7.15 (m, 2H), 7.09 – 7.06 (m, 2H), 3.60 (q, J = 7.2 Hz, 1H), 2.44 (d, J = 7.2 Hz, 2H), 1.84 (dp, J = 13.5, 6.8 Hz, 1H), 1.46 (t, J = 3.6 Hz, 6H), 1.38 (s, 3H), 0.88 (d, J = 6.8 Hz, 6H), 0.82 (s, 9H).

^{13}C NMR (101 MHz, CDCl$_3$) δ 173.9, 140.1, 138.6, 129.1, 127.1, 86.9, 46.6, 45.0, 38.3, 30.2, 24.9, 22.3, 20.4, 20.2, 18.0.

Enantioselective ratio = 93:7.0, determined by HPLC (Daicel Chiralpak IC-H Column, *n*-Hexane: *i*-PrOH = 98.0:2.0, flow rate 1.0 mL/min, T = 25 °C, λ = 214 nm): t_R = 7.86 min (major), t_R = 9.41 min (minor).

$[\alpha]_D^{27}$ = -8.25 (c = 1.0, CHCl$_3$).

All the resonances in ^{1}H NMR spectrum were consistent with the reported values.[1] ^{1}H and ^{13}C NMR spectra are provided below.

2,3,3-trimethylbutan-2-yl (*R*)-2-(2-fluoro-[1,1'-biphenyl]-4-yl)propanoate (2aj)

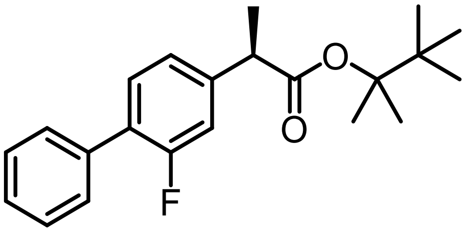

A colorless oil (53.0 mg, 78% yield, 90% ee) from 4-bromo-2-fluorobiphenyl (50.0 mg, 0.2 mmol).

^{1}H NMR (400 MHz, $CDCl_3$) δ 7.38 – 7.34 (m, 2H), 7.29 – 7.14 (m, 4H), 6.97 – 6.90 (m, 2H), 3.50 (q, *J* = 7.2 Hz, 1H), 1.33 (d, *J* = 7.2 Hz, 3H), 1.32 (s, 3H), 1.26 (s, 3H), 0.71 (s, 9H).

^{19}F NMR (376 MHz, $CDCl_3$) δ -118.04 – -118.00 (m, 1F).

^{13}C NMR (101 MHz, $CDCl_3$) δ 173.1, 159.6 (d, *J* = 248.1 Hz), 142.6 (d, *J* = 7.7 Hz), 135.6, 130.6 (d, *J* = 4.0 Hz), 128.9 (d, *J* = 3.0 Hz), 128.4, 127.6, 127.4 (d, *J* = 13.5 Hz), 123.6 (d, *J* = 3.4 Hz), 115.2 (d, *J* = 23.5 Hz), 87.6, 46.5 (d, *J* = 1.5 Hz), 38.4, 25.0, 20.5, 20.3, 18.0.

Enantioselective ratio = 94.8:5.2, determined by HPLC (Daicel Chiralpak AD-H Column, *n*-Hexane: *i*-PrOH = 99.8:0.2, flow rate 0.5 mL/min, T = 25 °C, λ = 254 nm): t_R = 12.88 min (major), t_R = 14.86 min (minor).

$[\alpha]_D^{30.3}$ = -24.61 (c = 0.2, $CHCl_3$).

All the resonances in ^{1}H NMR spectrum were consistent with the reported values.[2]

^{1}H and ^{13}C NMR spectra are provided below.

2,3,3-trimethylbutan-2-yl (*R*)-2-(3-benzoylphenyl)propanoate (2ak)

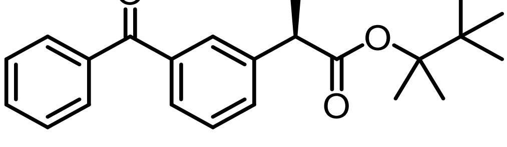

A colorless oil (45.8 mg, 65% yield, 92% ee) from 3-bromobenzophenone (53.0 mg, 0.2 mmol).

^{1}H NMR (400 MHz, $CDCl_3$) δ 7.79 – 7.75 (m, 4H), 7.60 – 7.55 (m, 1H), 7.49 – 7.45 (m, 2H), 7.40 – 7.36 (m, 2H), 3.73 (q, J = 7.2 Hz, 1H), 1.51 (d, J = 7.2 Hz, 3H), 1.48 (s, 3H), 1.40 (s, 3H), 0.84 (s, 9H).

^{13}C NMR (101 MHz, $CDCl_3$) δ 196.3, 172.9, 146.0, 137.7, 136.1, 132.3, 130.4, 129.9, 128.2, 127.5, 87.6, 47.0, 38.3, 25.0, 20.4, 20.2, 17.9.

IR (neat): 2975, 1722, 1659, 1448, 1281, 1126, 954, 897, 784, 643 cm^{-1}.

HRMS (ESI) calcd for $C_{23}H_{28}O_3Na_1$ $[M+Na]^+$: 375.1931, found: 375.1933.

Enantioselective ratio = 96.0:4.0, determined by HPLC (Daicel Chiralpak IC-H Column, *n*-Hexane: *i*-PrOH = 98.0:2.0, flow rate 1.0 mL/min, T = 25 °C, λ = 214 nm): t_R = 18.03 min (minor), t_R = 23.03 min (major).

$[\alpha]_D^{25.9}$ = -13.09 (c = 1.0, $CHCl_3$).

2,3,3-trimethylbutan-2-yl (*R*)-2-(3-phenoxyphenyl)propanoate (2al)

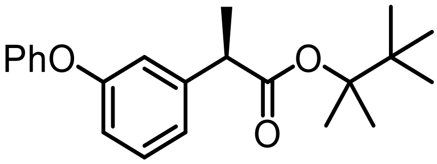

A colorless oil (48.2 mg, 71% yield, 90% ee) from 3-bromophenoxybenzene (50.0 mg, 0.2 mmol).

^{1}H NMR (400 MHz, $CDCl_3$) δ 7.32 – 7.26 (m, 2H), 7.24 – 7.22 (m, 1H), 7.08 – 7.04 (m, 1H), 7.01 – 6.98 (m, 1H), 6.97 – 6.94 (m, 2H), 6.92 – 6.84 (m, 1H), 6.87 – 6.83 (m, 1H), 3.57 (q, *J* = 7.2 Hz, 1H), 1.43 (s, 3H), 1.41 (d, *J* = 7.2 Hz, 3H), 1.36 (s, 3H), 0.81 (s, 9H).

^{13}C NMR (101 MHz, $CDCl_3$) δ 173.2, 157.3, 143.4, 129.5, 129.7, 123.1, 122.4, 118.8, 118.1, 117.3, 87.2, 46.8, 38.3, 31.5, 30.1, 25.0, 20.4, 20.2, 18.0.

Enantioselective ratio = 95.0:5.0, determined by HPLC (Daicel Chiralpak IC-H Column, *n*-Hexane: *i*-PrOH = 98.0:2.0, flow rate 1.0 mL/min, T = 25 °C, λ = 214 nm): t_R = 5.76 min (minor), t_R = 6.43 min (major).

$[\alpha]_D^{25.9}$ = -5.16 (c = 1.0, $CHCl_3$).

All the resonances in ^{1}H NMR spectrum were consistent with the reported values.[2]

^{1}H and ^{13}C NMR spectra are provided below.

2,3,3-trimethylbutan-2-yl (*R*)-2-(4-phenoxyphenyl)propanoate (2am)

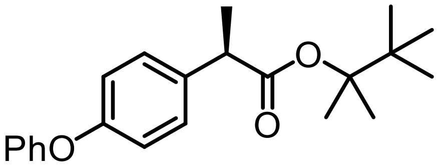

A colorless oil (51.5 mg, 76% yield, 87% ee) from 4-bromophenoxybenzene (50.0 mg, 0.2 mmol).

^{1}H NMR (400 MHz, $CDCl_3$) δ 7.35 – 7.29 (m, 1H), 7.25 – 7.21 (m, 2H), 7.11 – 7.06 (m, 1H), 7.00 – 6.94 (m, 4H), 3.63 (q, *J* = 7.2 Hz, 1H), 1.48 (d, *J* = 7.2 Hz, 3H), 1.47 (s, 3H), 1.40 (s, 3H), 0.86 (s, 9H).

^{13}C NMR (101 MHz, $CDCl_3$) δ 173.7, 155.8, 136.3, 129.7, 128.8, 123.0, 119.1, 118.5, 87.2, 46.3, 38.4, 25.0, 20.4, 20.2, 18.1.

IR (neat): 2975, 1723, 1590, 1487, 1235, 1127, 1071, 870, 787, 691 cm^{-1}.

HRMS (ESI) calcd for $C_{22}H_{29}O_3$ $[M+H]^+$: 341.2117, found: 341.2119.

Enantioselective ratio = 93.5:6.5, determined by HPLC (Daicel Chiralpak IC-H Column, *n*-Hexane: *i*-PrOH = 99:1, flow rate 1.0 mL/min, T = 25 °C, λ = 214 nm): t_R = 9.88 min (major), t_R = 10.36 min (minor).

$[\alpha]_D^{28.2}$ = -9.06 (c = 0.1, $CHCl_3$).

methyl (*S*)-4-(3-(benzyloxy)-1-oxo-1-((2,3,3-trimethylbutan-2-yl)oxy)propan-2-yl)benzoate (2an)

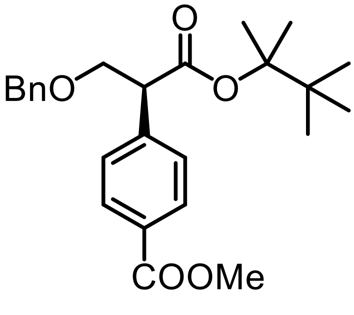

A colorless oil (57.6 mg, 71% yield, 66% ee) from methyl 4-bromobenzoate (43.0 mg, 0.2 mmol).

^{1}H NMR (400 MHz, CDCl$_3$) δ 8.00 – 7.96 (m, 2H), 7.38 – 7.34 (m, 2H), 7.34 – 7.23 (m, 5H), 4.59 – 4.48 (m, 2H), 4.04 (dd, *J* = 9.2, 8.0 Hz, 1H), 3.91 (s, 3H), 3.73 (dd, *J* = 9.2, 6.2 Hz, 1H), 1.48 (s, 3H), 1.38 (s, 3H), 0.84 (s, 9H).

^{13}C NMR (101 MHz, CDCl$_3$) δ 170.6, 142.1, 137.9, 129.8, 129.2, 128.3, 128.2, 127.6, 127.6, 88.2, 73.3, 71.1, 53.4, 52.1, 38.3, 25.0, 20.6, 20.2.

IR (neat): 2958, 1721, 1611, 1454, 1367, 1276, 1106, 1020, 938, 847 cm^{-1}.

HRMS (ESI) calcd for $C_{25}H_{32}O_5Na_1$ $[M+Na]^+$: 435.2142, found: 435.2143.

Enantioselective ratio = 83.0:17.0, determined by HPLC (Daicel Chiralpak IG-H Column, *n*-Hexane: *i*-PrOH = 98:2, flow rate 1.0 mL/min, T = 25 °C, λ = 214 nm): t_R = 16.80 min (major), t_R = 22.96 min (minor).

$[\alpha]_D^{26.1}$ = -2.9 (c = 1.0, $CHCl_3$).

Methyl (*R*)-4-(3-(4-methoxyphenyl)-1-oxo-1-((2,3,3-trimethylbutan-2-yl)oxy)propan-2-yl)benzoate (2ao)

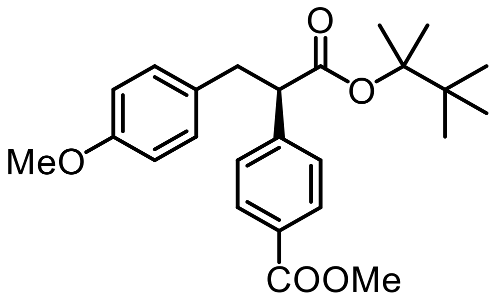

A colorless oil (42.0 mg, 52% yield, 77% ee) from methyl 4-bromobenzoate (43.0 mg, 0.2 mmol).

^{1}H NMR (400 MHz, CDCl$_3$) δ 8.00 – 7.95 (m, 2H), 7.33 – 7.26 (m, 2H), 7.05 – 7.02 (m, 2H), 6.91 – 6.86 (m, 2H), 3.90 (s, 3H), 3.78 (t, *J* = 7.8 Hz, 1H), 3.75 (s, 3H), 3.34 (dd, *J* = 14.0, 8.0 Hz, 1H), 2.96 (dd, *J* = 14.0, 7.6 Hz, 1H), 1.37 (s, 3H), 1.33 (s, 3H), 0.80 (s, 9H).

^{13}C NMR (101 MHz, CDCl$_3$) δ 171.9, 158.1, 144.5, 130.8, 129.8, 129.7, 128.9, 128.1, 113.7, 87.8, 55.2, 55.0, 52.0, 38.2, 38.2, 25.0, 20.4, 20.1.

IR (neat): 2959, 1715, 1611, 1514, 1439, 1351, 1275, 1210, 1115, 967, 854, 746 cm^{-1}.

HRMS (ESI) calcd for $C_{25}H_{32}O_5Na_1$ $[M+Na]^+$: 435.2142, found: 435.2143.

Enantioselective ratio = 89:11, determined by HPLC (Daicel Chiralpak IG-H Column, *n*-Hexane: *i*-PrOH = 99.0:1.0, flow rate 0.8 mL/min, T = 25 °C, λ = 214 nm): t_R = 17.17 min (major), t_R = 18.49 min (minor).

[α]$_D^{26.5}$ = -39.88 (c = 1.0, $CHCl_3$).

Methyl (*R*)-4-(1-oxo-3-phenyl-1-((2,3,3-trimethylbutan-2-yl)oxy)propan-2-yl)benzoate (2ap)

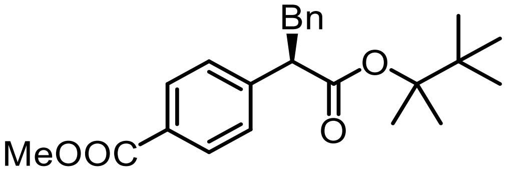

A colorless oil (49.7 mg, 65% yield, 80% ee) from methyl 4-bromobenzoate (43.0 mg, 0.2 mmol).

^{1}H NMR (400 MHz, $CDCl_3$) δ 7.98 – 7.95 (m, 2H), 7.36 – 7.33 (m, 2H), 7.24 – 7.18 (m, 2H), 7.18 – 7.13 (m, 1H), 7.11 – 7.07 (m, 2H), 3.90 (s, 3H), 3.83 (t, J = 7.8 Hz, 1H), 3.41 (dd, J = 13.8, 8.0 Hz, 1H), 3.02 (dd, J = 13.8, 7.6 Hz, 1H), 1.36 (s, 3H), 1.33 (s, 3H), 0.79 (s, 9H).

^{13}C NMR (101 MHz, $CDCl_3$) δ 171.9, 166.9, 144.4, 138.7, 129.8, 128.9, 128.9, 128.3, 128.0, 126.4, 87.9, 54.8, 52.0, 39.1, 38.3, 25.0, 20.4, 20.1.

IR (neat): 2948, 1712, 1609, 1436, 1366, 1275, 1207, 1112, 1024, 874, 784, 698 cm^{-1}.

HRMS (ESI) calcd for $C_{24}H_{30}O_4Na_1$ $[M+Na]^+$: 405.2036, found: 405.2035.

Enantioselective ratio = 90:10, determined by HPLC (Daicel Chiralpak IC-H Column, *n*-Hexane: *i*-PrOH = 98.0:2.0, flow rate 1.0 mL/min, T = 25 °C, λ = 214 nm): t_R = 9.37 min (major), t_R = 9.94 min (minor)

[α]$_D^{26.6}$ = -12.65 (c = 1.0, $CHCl_3$).

Methyl (*R*)-4-(1-oxo-4-phenyl-1-((2,3,3-trimethylbutan-2-yl)oxy)butan-2-yl)benzoate (2aq)

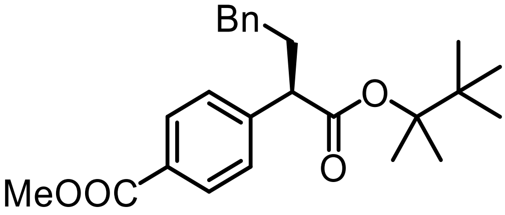

A colorless oil (65.0 mg, 82% yield, 87% ee) from methyl 4-bromobenzoate (43.0 mg, 0.2 mmol).

^{1}H NMR (400 MHz, $CDCl_3$) δ 8.10 – 7.98 (m, 2H), 7.38 – 7.33 (m, 2H), 7.31 – 7.26 (m, 2H), 7.22 – 7.18 (m, 1H), 7.16 – 7.12 (m, 2H), 3.91 (s, 3H), 3.53 (t, J = 7.6 Hz, 1H), 2.65 – 2.52 (m, 2H), 2.50 – 2.37 (m, 1H), 2.11 – 2.00 (m, 1H), 1.47 (s, 3H), 1.38 (s, 3H), 0.84 (s, 9H).

^{13}C NMR (101 MHz, $CDCl_3$) δ 172.1, 166.9, 144.7, 141.1, 129.8, 128.9, 128.4, 128.4, 128.0, 126.0, 87.8, 52.3, 52.0, 38.3, 34.1, 33.5, 25.0, 20.5, 20.2.

IR (neat): 2954, 1721, 1610, 1369, 1276, 1178, 1108, 1019, 966, 848, 744, 699 cm^{-1}.

HRMS (ESI) calcd for $C_{25}H_{32}O_4Na_1$ $[M+Na]^+$: 419.2193, found: 419.2191.

Enantioselective ratio = 93.5:6.5, determined by HPLC (Daicel Chiralpak IG-H Column, *n*-Hexane: *i*-PrOH = 98.0:2.0, flow rate 1.0 mL/min, T = 25 °C, λ = 214 nm): t_R = 12.05 min (major), t_R = 21.25 min (minor).

$[\alpha]_D^{26}$ = -17.98 (c = 1.0, $CHCl_3$).

Methyl (*R*)-4-(4-(methylthio)-1-oxo-1-((2,3,3-trimethylbutan-2-yl)oxy)butan-2-yl)benzoate (2ar)

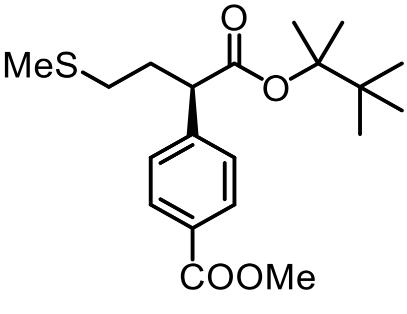

A colorless oil (54.1 mg, 74% yield, 84% ee) from methyl 4-bromobenzoate (43.0 mg, 0.2 mmol).

^{1}H NMR (400 MHz, $CDCl_3$) δ 8.00 – 7.95 (m, 2H), 7.34 – 7.32 (m, 2H), 3.88 (s, 3H), 3.76 – 3.70 (m, 1H), 2.45 – 2.29 (m, 3H), 2.05 (s, 3H), 2.03 – 1.95 (m, 1H), 1.44 (s, 3H), 1.33 (s, 3H), 0.80 (s, 9H).

^{13}C NMR (101 MHz, $CDCl_3$) δ 171.8, 166.8, 144.3, 129.9, 129.0, 128.0, 87.9, 52.0, 51.4, 38.2, 31.7, 31.6, 24.9, 20.4, 20.1, 15.2.

IR (neat): 2956, 1719, 1610, 1435, 1370, 1275, 1177, 1109, 1019, 963, 847, 742, 703 cm^{-1}.

HRMS (ESI) calcd for $C_{20}H_{30}O_4S_1Na_1$ $[M+Na]^+$: 389.1757, found: 389.1758.

Enantioselective ratio = 92:8, determined by HPLC (Daicel Chiralpak IG-H Column, *n*-Hexane: *i*-PrOH = 98.0:2.0, flow rate 0.9 mL/min, T = 25 °C, λ = 214 nm): t_R = 12.33 min (major), t_R = 13.25 min (minor).

$[\alpha]_D^{26}$ = -9.34 (c = 0.2, $CHCl_3$).

Methyl (*R*)-4-(1-oxo-1-((2,3,3-trimethylbutan-2-yl)oxy)hept-6-en-2-yl)benzoate (2as)

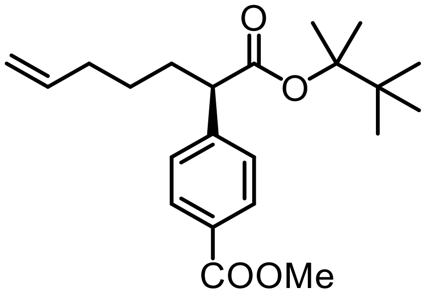

A colorless oil (22.0 mg, 30% yield, 91% ee) from methyl 4-bromobenzoate (43.0 mg, 0.2 mmol).

^{1}H NMR (400 MHz, $CDCl_3$) δ 8.01 – 7.96 (m, 2H), 7.35 – 7.32 (m, 2H), 5.79 – 5.69 (m, 1H), 5.01 – 4.95 (m, 1H), 4.95 – 4.91 (m, 1H), 3.90 (s, 3H), 3.51 (t, *J* = 7.6 Hz, 1H), 2.13 – 2.01 (m, 2H), 1.79 – 1.70 (m, 1H), 1.45 (s, 3H), 1.36 (s, 3H), 1.43 – 1.24 (m, 3H), 0.84 (s, 9H).

^{13}C NMR (101 MHz, $CDCl_3$) δ 172.4, 166.9, 145.1, 138.2, 129.8, 128.8, 127.9, 114.8, 87.7, 53.1, 52.0, 38.3, 33.4, 32.2, 26.7, 25.0, 20.5, 20.2.

IR (neat): 2952, 1721, 1611, 1435, 1370, 1275, 1176, 1110, 911, 846, 742 cm^{-1}.

HRMS (ESI) calcd for $C_{22}H_{32}O_4Na_1$ $[M+Na]^+$: 383.2193, found: 383.2196.

Enantioselective ratio = 95.5:4.5, determined by HPLC (Daicel Chiralpak IC-H Column, *n*-Hexane: *i*-PrOH = 99.0:1.0, flow rate 0.8 mL/min, T = 25 °C, λ = 214 nm): t_R = 12.47 min (major), t_R = 13.59 min (minor)

$[\alpha]_D^{26.3}$ = -6.42 (c = 1.0, $CHCl_3$).

Methyl (*R*)-4-(1-oxo-1-((2,3,3-trimethylbutan-2-yl)oxy)butan-2-yl)benzoate (2at)

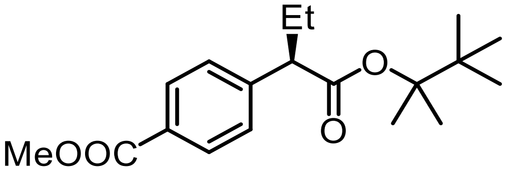

A colorless oil (41.5 mg, 66% yield, 92% ee) from methyl 4-bromobenzoate (43.0 mg, 0.2 mmol).

^{1}H NMR (400 MHz, $CDCl_3$) δ 8.01 – 7.96 (m, 2H), 7.35 – 7.31 (m, 2H), 3.89 (s, 3H), 3.41 (t, J = 7.8 Hz, 1H), 2.15 – 2.04 (m, 1H), 1.81 – 1.70 (m, 1H), 1.44 (s, 3H), 1.36 (s, 3H), 0.88 (t, J = 7.2 Hz, 3H), 0.84 (s, 9H).

^{13}C NMR (101 MHz, $CDCl_3$) δ 172.4, 166.9, 145.0, 129.7, 128.8, 127.9, 87.6, 55.0, 52.0, 38.3, 25.9, 25.0, 20.4, 20.1, 12.0.

IR (neat): 2965, 1721, 1611, 1377, 1275, 1175, 1110, 1020, 967, 847, 775, 703 cm^{-1}.

HRMS (ESI) calcd for $C_{19}H_{28}O_4Na_1$ $[M+Na]^+$: 343.1880, found: 343.1879.

Enantioselective ratio = 96:4, determined by HPLC (Daicel Chiralpak IC-H Column, *n*-Hexane: *i*-PrOH = 98.0:2.0, flow rate 0.5 mL/min, T = 25 °C, λ = 214 nm): t_R = 18.45 min (major), t_R = 19.39 min (minor)

$[\alpha]_D^{26.6}$ = -12.65 (c = 1.0, $CHCl_3$).

Methyl (R)-4-(1-oxo-1-((2,3,3-trimethylbutan-2-yl)oxy)hexan-2-yl)benzoate (2au)

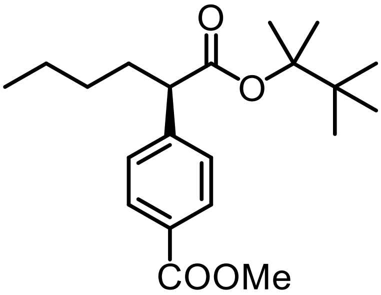

A colorless oil (43.4 mg, 65% yield, 92% ee) from methyl 4-bromobenzoate (43.0 mg, 0.2 mmol).

^{1}H NMR (400 MHz, $CDCl_3$) δ 8.00 – 7.95 (m, 2H), 7.36 – 7.33 (m, 2H), 3.89 (s, 3H), 3.49 (t, J = 7.6 Hz, 1H), 2.11 – 2.02 (m, 1H), 1.79 – 1.68 (m, 1H), 1.44 (s, 3H), 1.36 (s, 3H), 1.34 – 1.11 (m, 4H), 0.86 (t, *J* = 7.2 Hz, 3H), 0.84 (s, 9H).

^{13}C NMR (101 MHz, $CDCl_3$) δ 172.5, 167.0, 145.20, 129.8, 128.7, 127.9, 87.6, 53.2, 52.0, 38.3, 32.4, 29.6, 25.0, 22.4, 20.4, 20.2, 13.9.

IR (neat): 2956, 1722, 1611, 1435, 1375, 1275, 1175, 1110, 1019, 965, 847, 746 cm^{-1}.

HRMS (ESI) calcd for $C_{21}H_{32}O_4Na_1$ $[M+Na]^+$: 371.2193, found: 371.2194.

Enantioselective ratio = 96:4, determined by HPLC (Daicel Chiralpak IG-H Column, *n*-Hexane: *i*-PrOH = 98.0:2.0, flow rate 0.5 mL/min, T = 25 °C, λ = 214 nm): t_R = 12.29 min (major), t_R = 13.62 min (minor).

$[\alpha]_D^{27.6}$ = -20.40 (c = 1.0, $CHCl_3$).

Methyl (*R*)-4-(4-methyl-1-oxo-1-((2,3,3-trimethylbutan-2-yl)oxy)pentan-2-yl)benzoate (2av)

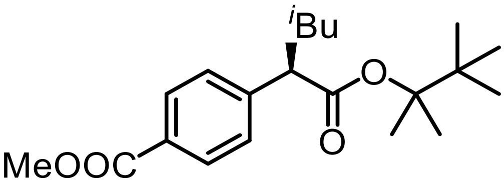

A colorless oil (57.7 mg, 83% yield, 94% ee) from methyl 4-bromobenzoate (43.0 mg, 0.2 mmol).

^{1}H NMR (400 MHz, $CDCl_3$) δ 8.00 – 7.96 (m, 2H), 7.37 – 7.34 (m, 2H), 3.90 (s, 3H), 3.62 (t, *J* = 7.8 Hz, 1H), 2.03 – 1.93 (m, 1H), 1.68 – 1.56 (m, 1H), 1.52 – 1.42 (m, 1H), 1.44 (s, 3H), 1.36 (s, 3H), 0.92 (s, 3H), 0.90 (s, 3H), 0.85 (s, 9H).

^{13}C NMR (101 MHz, $CDCl_3$) δ 172.6, 167.0, 145.3, 129.8, 128.8, 128.0, 87.6, 52.0, 51.2, 41.7, 38.3, 25.9, 25.0, 22.6, 22.3, 20.4, 20.2.

IR (neat): 2957, 1722, 1435, 1369, 1611, 1275, 1110, 938, 847. 746, 703 cm^{-1}

HRMS (ESI) calcd for $C_{21}H_{32}O_4Na_1$ $[M+Na]^+$: 371.2193, found: 371.2189.

Enantioselective ratio = 97.0:3.0, determined by HPLC (Daicel Chiralpak IG-H Column, *n*-Hexane: *i*-PrOH = 99.0:1.0, flow rate 0.4 mL/min, T = 25 °C, λ = 214 nm): t_R = 21.02 min (major), t_R = 22.31 min (minor)

$[\alpha]_D^{26}$ = -9.95 (c = 1.0, $CHCl_3$).

Methyl (*R*)-4-(3-methyl-1-oxo-1-((2,3,3-trimethylbutan-2-yl)oxy)butan-2-yl)benzoate (2aw)

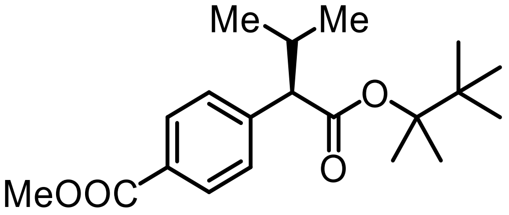

A colorless oil (37.4 mg, 56% yield, 94% ee) from methyl 4-bromobenzoate (43.0 mg, 0.2 mmol).

^{1}H NMR (400 MHz, $CDCl_3$) δ 7.97 (d, J = 8.2 Hz, 2H), 7.40 – 7.37 (m, 2H), 3.90 (s, 3H), 3.11 (d, J = 10.8 Hz, 1H), 2.40 – 2.28 (m, 1H), 1.44 (s, 3H), 1.36 (s, 3H), 1.07 (d, J = 6.4 Hz, 3H), 0.91 (s, 9H), 0.68 (d, J = 6.8 Hz, 3H).

^{13}C NMR (101 MHz, $CDCl_3$) δ 172.3, 167.0, 144.2, 129.7, 128.9, 128.5, 87.7, 61.8, 52.0, 38.3, 31.2, 25.1, 21.4, 20.4, 20.2, 20.1.

IR (neat): 2960, 1721, 1610, 1466, 1369, 1275, 1176, 1100, 937, 853, 774, 702 cm^{-1}.

HRMS (ESI) calcd for $C_{20}H_{30}O_4Na_1$ $[M+Na]^+$: 357.2036, found: 357.2037.

Enantioselective ratio = 97.0:3.0, determined by HPLC (Daicel Chiralpak IG-H Column, *n*-Hexane: *i*-PrOH = 99.0:1.0, flow rate 0.5 mL/min, T = 25 °C, λ = 214 nm): t_R = 20.70 min (major), t_R = 22.42 min (minor).

$[\alpha]_D^{26.3}$ = -1.41 (c = 0.5, $CHCl_3$).

Methyl (*R*)-4-(1-cyclopentyl-2-oxo-2-((2,3,3-trimethylbutan-2-yl)oxy)ethyl)benzoate (2ax)

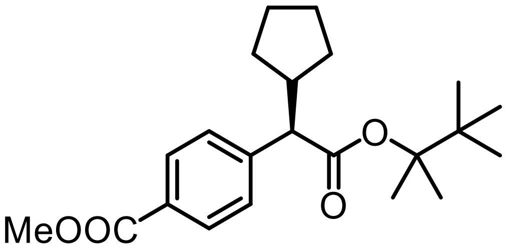

A colorless oil (36.7 mg, 51% yield, 94% ee) from methyl 4-bromobenzoate (43.0 mg, 0.2 mmol).

^{1}H NMR (400 MHz, $CDCl_3$) δ 8.02 – 7.91 (m, 2H), 7.43 – 7.36 (m, 2H), 3.89 (s, 2H), 3.24 (d, J = 11.2 Hz, 1H), 2.60 – 2.49 (m, 1H), 1.99 – 1.90 (m, 1H), 1.73 – 1.52 (m, 3H), 1.48 – 1.44 (m, 1H), 1.43 (s, 3H), 1.39 – 1.24 (m, 2H), 1.36 (s, 3H), 0.99 – 0.91 (m, 1H), 0.89 (s, 9H).

^{13}C NMR (101 MHz, $CDCl_3$) δ 172.32, 166.96, 144.76, 129.65, 128.78, 128.24, 87.58, 59.63, 51.98, 42.78, 38.30, 31.48, 30.63, 25.13, 25.08, 24.84, 20.41, 20.14.

IR (neat): 2954, 1721, 1610, 1435, 1369, 1274, 1175, 1020, 996, 848, 749, 702 cm^{-1}.

HRMS (ESI) calcd for $C_{19}H_{28}O_4Na_1$ $[M+Na]^+$: 343.1880, found: 343.1879.

Enantioselective ratio = 97.0:3.0, determined by HPLC (Daicel Chiralpak IG-H Column, *n*-Hexane: *i*-PrOH = 98.0:2.0, flow rate 0.5 mL/min, T = 25 °C, λ = 214 nm): t_R = 11.94 min (major), t_R = 13.30 min (minor)

$[\alpha]_D^{26}$ = -4.87 (c = 1.0, $CHCl_3$).

Methyl (*R*)-4-(1-cyclohexyl-2-oxo-2-((2,3,3-trimethylbutan-2-yl)oxy)ethyl)benzoate (2ay)

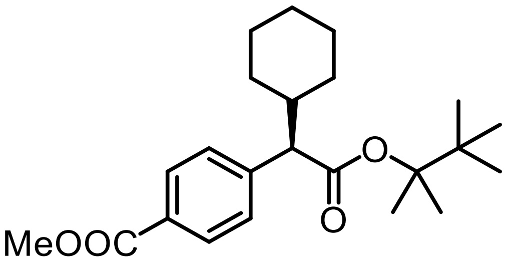

A colorless oil (19.1 mg, 35% yield, 98% ee) from methyl 4-bromobenzoate (43.0 mg, 0.2 mmol).

^{1}H NMR (400 MHz, CDCl$_3$) δ 7.99 – 7.95 (m, 2H), 7.38 – 7.35 (m, 2H), 3.89 (s, 3H), 3.19 (d, J = 10.8 Hz, 1H), 2.07 – 1.97 (m, 1H), 1.91 – 1.86 (m, 1H), 1.77 – 1.70 (m, 1H), 1.65 – 1.58 (m, 2H), 1.43 (s, 3H), 1.36 (s, 3H), 1.33 – 1.23 (m, 2H), 1.22 – 1.09 (m, 2H), 1.08 – 0.99 (m, 1H), 0.91 (s, 9H), 0.78 – 0.66 (m, 1H).

^{13}C NMR (101 MHz, CDCl$_3$) δ 172.2, 167.0, 143.8, 129.6, 128.8, 128.6, 87.7, 60.5, 52.0, 40.4, 38.3, 31.8, 30.3, 26.2, 26.0, 25.9, 25.1, 20.5, 20.1.

IR (neat): 2926, 1721, 1610, 1435, 1370, 1275, 1170, 1020, 938, 848, 775, 750 cm^{-1}.

HRMS (ESI) calcd for $C_{23}H_{34}O_4Na_1$ [M+Na]$^+$: 397.2349, found: 397.2348.

Enantioselective ratio = 99.0:1.0, determined by HPLC (Daicel Chiralpak IC-H Column, *n*-Hexane: *i*-PrOH = 99.0:1.0, flow rate 1.0 mL/min, T = 25 °C, λ = 214 nm): t_R = 23.24 min (major), t_R = 24.55 min (minor).

$[\alpha]_D^{26}$ = -1.41 (c = 0.5, CHCl$_3$).

(*R*)-2-(2-fluoro-[1,1'-biphenyl]-4-yl)propanoic acid (3)

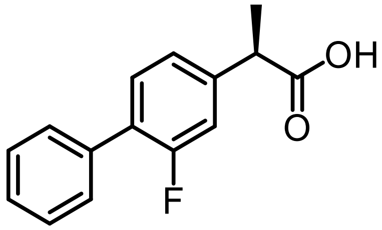

A white solid (48.8 mg, quant. yield, 90% ee) from 2,3,3-trimethylbutan-2-yl (*R*)-2-(2-fluoro-[1,1'-biphenyl]-4-yl)propanoate (72.0 mg, 0.2 mmol).

^{1}H NMR (400 MHz, CDCl$_3$) δ 11.68 (s, 1H), 7.57 – 7.52 (m, 2H), 7.48 – 7.36 (m, 4H), 7.22 – 7.15 (m, 1H), 3.81 (q, *J* = 7.2 Hz, 1H), 1.58 (d, *J* = 7.2 Hz, 3H).

^{19}F NMR (377 MHz, CDCl$_3$) δ -117.32 – -117.50 (m, 1F).

^{13}C NMR (101 MHz, CDCl$_3$) δ 180.7, 159.6 (d, *J* = 248.5 Hz), 140.8 (d, *J* = 7.6 Hz), 135.3 (d, *J* = 1.2 Hz), 130.8 (d, *J* = 4.0 Hz), 128.9 (d, *J* = 3.0 Hz), 128.4, 128.1 (d, *J* = 13.6 Hz), 127.7, 123.7 (d, *J* = 3.4 Hz), 44.8 (d, *J* = 1.6 Hz), 17.9.

All the resonances in ^{1}H NMR spectrum were consistent with the reported values.[3]

^{1}H and ^{13}C NMR spectra are provided below.

Methyl 4-(((1*R*, 2*S*)-2-(((2,3,3-trimethylbutan-2-yl)oxy)carbonyl)cyclopentyl)methyl)benzoate (racemic-6)

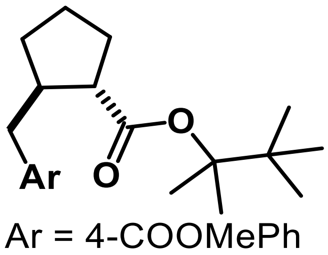

^{1}H NMR (600 MHz, CDCl$_3$) δ 7.94 – 7.93 (m, 2H), 7.22 (d, *J* = 8.0 Hz, 2H), 3.89 (s, 3H), 2.90 (dd, *J* = 13.2, 4.8 Hz, 1H), 2.82 (q, *J* = 7.2 Hz, 1H), 2.47 (dd, *J* = 13.2, 10.8 Hz, 1H), 2.42 – 2.34 (m, 1H), 2.01 – 1.92 (m, 1H), 1.87 – 1.77 (m, 1H), 1.59 – 1.53 (m, 2H), 1.53 (s, 6H), 1.50 – 1.44 (m, 1H), 0.98 (s, 9H).

^{13}C NMR (151 MHz, CDCl$_3$) δ 174.4, 167.1, 147.1, 129.6, 128.8, 127.8, 87.0, 51.9, 49.2, 44.7, 38.3, 36.8, 30.3, 27.8, 25.2, 23.2, 20.6, 20.5.

IR (neat): 2954, 1718, 1610, 1435, 1369, 1275, 1176, 1020, 967, 846, 758 cm^{-1}.

HRMS (ESI) calcd for $C_{22}H_{32}O_4Na_1$ [M+Na]$^+$: 383.2193, found: 383.2196.

Enantioselective ratio = 50:50, determined by HPLC (Daicel Chiralpak IC-H Column, *n*-Hexane: *i*-PrOH = 99.0:1.0, flow rate 1.0 mL/min, T = 25 °C, λ = 214 nm): t_R = 24.94 min (major), t_R = 26.30 min (minor)

^{1}H and ^{13}C NMR spectra are provided below.

Methyl 4-(((1*R*, 2*R*)-2-(((2,3,3-trimethylbutan-2-yl)oxy)carbonyl)cyclopentyl)methyl)benzoate (racemic)

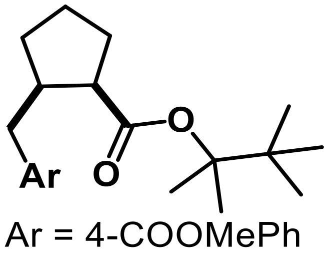

^{1}H NMR (600 MHz, CDCl$_3$) δ 7.93 – 7.91 (m, 2H), 7.23 – 7.21 (m, 2H), 3.89 (s, 3H), 2.90 (dd, *J* = 13.2, 6.0 Hz, 1H), 2.57 (dd, *J* = 13.2, 8.4 Hz, 1H), 2.45 – 2.38 (m, 1H),

2.34 (q, J = 8.4 Hz, 1H), 1.96 – 1.90 (m, 1H), 1.83 – 1.77 (m, 1H), 1.76 – 1.70 (m, 1H), 1.65 – 1.60 (m, 1H), 1.43 (s, 3H), 1.42 (s, 3H), 1.27 – 1.21 (m, 1H), 0.94 (s, 9H).

^{13}C NMR (151 MHz, CDCl$_3$) δ 175.2, 167.1, 146.5, 129.6, 129.0, 127.9, 86.6, 51.9, 51.5, 45.2, 41.1, 38.3, 32.1, 30.1, 25.1, 24.3, 20.4, 20.4.

IR (neat): 2954, 1718, 1610, 1435, 1369, 1275, 1176, 1020, 967, 846, 758 cm^{-1}.

HRMS (ESI) calcd for $C_{22}H_{32}O_4Na_1$ [M+Na]$^+$: 383.2193, found: 383.2196.

Enantioselective ratio = 50.0:50.0, determined by HPLC (Daicel Chiralpak IG-H Column, *n*-Hexane: *i*-PrOH = 99.0:1.0, flow rate 0.7 mL/min, T = 25 °C, λ = 214 nm): t_R = 32.73 min (major), t_R = 34.89 min (minor)

^{1}H and ^{13}C NMR spectra are provided below.

Supplementary: Characterization data for the Suboptimal substrates products

2,3,3-trimethylbutan-2-yl (*R,E*)-2-methyl-4-phenylbut-3-enoate

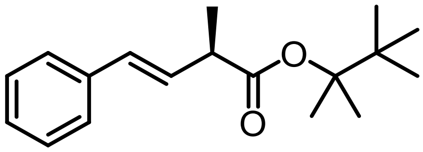

A colorless oil (19.0 mg, 36% yield, 52% ee) from (*E*)-1-(2-bromovinyl)-benzene (36.0 mg, 0.2 mmol).

^{1}H NMR (400 MHz, CDCl$_3$) δ 7.38 – 7.34 (m, 2H), 7.33 – 7.28 (m, 2H), 7.25 – 7.19 (m, 1H), 6.47 (d, J = 16.0 Hz, 1H), 6.27 (dd, J = 16.0, 8.0 Hz, 1H), 3.23 (q, J = 7.2 Hz, 1H), 1.51 (s, 3H), 1.50 (s, 3H), 1.34 (d, J = 7.0 Hz, 3H), 0.99 (s, 9H).

^{13}C NMR (101 MHz, CDCl$_3$) δ 173.6, 137.1, 130.7, 129.4, 128.5, 127.3, 126.2, 87.2, 44.8, 38.4, 25.1, 20.5, 20.4, 17.8.

HRMS (ESI) calcd for $C_{18}H_{26}O_2Na_1$ [M+Na]$^+$: 297.1825, found: 297.1823.

Enantioselective ratio = 76:24, determined by HPLC (Daicel Chiralpak Cellulose-3 Column, MeCN: H_2O = 85:15, flow rate 0.7 mL/min, T = 25 °C, λ = 214 nm): t_R = 6.340 min (major), t_R = 6.913 min (minor).

^{1}H and ^{13}C NMR spectra are provided below.

2,3,3-trimethylbutan-2-yl (*R*)-2-(1*H*-inden-2-yl)propanoate

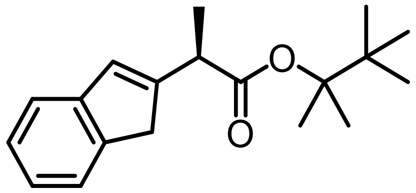

A colorless oil (28.0 mg, 50% yield, 72% ee) from 2-bromoindene (38.0 mg, 0.2 mmol).

^{1}H NMR (400 MHz, $CDCl_3$) δ 7.42 – 7.38 (m, 1H), 7.33 – 7.30 (m, 1H), 7.26 – 7.22 (m, 1H), 7.16 – 7.12 (m, 1H), 7.68 – 7.67 (m, 1H), 3.62 – 3.56 (m, 1H), 3.48 – 3.34 (m, 2H), 1.51 – 1.50 (m, 3H), 1.49 – 1.48 (m, 3H), 1.46 – 1.44 (m, 3H), 0.94 – 0.93 (m, 9H).

^{13}C NMR (101 MHz, $CDCl_3$) δ 173.2, 148.8, 144.8, 143.0, 127.6, 126.3, 124.2, 123.5, 120.5, 87.4, 43.3, 39.6, 38.4, 25.1, 20.4, 20.3, 17.0.

HRMS (ESI) calcd for $C_{19}H_{16}O_2Na_1$ $[M+Na]^+$: 309.1830, found: 309.1831.

Enantioselective ratio = 86.0:14.0, determined by SFC (Daicel Chiralpak IF-3 Column, 2% *i*-PrOH in supercritical CO_2, 2.5 mL/min, T = 25 °C, λ = 230 nm): t_R = 5.66 min (major), t_R = 6.08 min (minor).

^{1}H and ^{13}C NMR spectra are provided below.

2,3,3-trimethylbutan-2-yl (*R*)-2-(pyridin-3-yl)propanoate

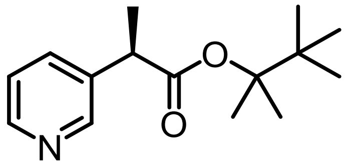

A colorless oil (14.5 mg, 30% yield, 80% ee) from 3-bromopyridine (31.0 mg, 0.2 mmol).

^{1}H NMR (400 MHz, $CDCl_3$) δ 8.54 – 8.52 (m, 1H), 8.50 – 8.48 (m, 1H), 7.61 (dt, *J* = 8.0, 2.0 Hz, 1H), 7.28 – 7.23 (m, 1H), 3.66 (q, *J* = 7.2 Hz, 1H), 1.50 (d, *J* = 7.2 Hz, 3H), 1.47 (s, 3H), 1.39 (s, 3H), 0.84 (s, 9H).

^{13}C NMR (101 MHz, $CDCl_3$) δ 172.8, 149.3, 148.3, 136.7, 134.7, 123.4, 87.9, 44.5, 38.3, 25.0, 20.5, 20.2, 17.9.

HRMS (ESI) calcd for $C_{15}H_{24}O_1N_1$ $[M+H]^+$: 250.1807, found: 250.1809.

Enantioselective ratio = 90:10, determined by HPLC (Daicel Chiralpak OD-H Column, *n*-Hexane: *i*-PrOH = 95:5, flow rate 1.0 mL/min, T = 25 °C, λ = 254 nm): t_R = 5.43 min (minor), t_R = 5.81 min (major).

^{1}H and ^{13}C NMR spectra are provided below.

methyl 4-((*S*)-1-(((1*R*,2*S*,5*R*)-2-isopropyl-5-methylcyclohexyl)oxy)-1-oxopropan-2-yl)benzoate

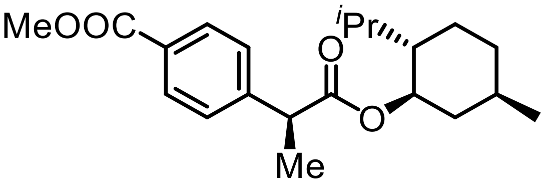

A colorless oil (29.0 mg, 60% yield, 50% dr) from methyl 2-bromobenzoate (43.0 mg, 0.2 mmol).

^{1}H NMR (400 MHz, $CDCl_3$) δ 8.00 – 7.96 (m, 2H), 7.38 – 7.34 (m, 2H), 4.69 – 4.58 (m, 1H), 3.91 (s, 3H), 3.74 (q, *J* = 7.2 Hz, 1H), 2.00 – 1.72 (m, 2H), 1.67 – 1.56 (m, 2H), 1.51 – 1.48 (m, 1H), 1.47 – 1.37 (m, 1H), 1.36 – 1.23 (m, 1H), 1.06 – 0.95 (m, 1H), 0.89 – 0.81 (m, 6H), 0.72 – 0.68 (m, 3H), 0.53 – 0.52 (m, 1H).

^{13}C NMR (101 MHz, $CDCl_3$) δ 173.4, 166.9, 146.0, 129.8, 128.9, 127.6, 74.8, 52.1, 47.0, 46.0, 40.7, 34.2, 31.3, 26.2, 23.4, 22.0, 20.6, 18.3, 16.2, 15.9.

HRMS (ESI) calcd for $C_{21}H_{30}O_4Na_1$ $[M+Na]^+$: 369.2042, found: 369.2044.

Enantioselective ratio = 75:25, determined by SFC (Daicel Chiralpak IF-3 Column, 2% *i*-PrOH in supercritical CO_2, 2.5 mL/min, T = 25 °C, λ = 230 nm): t_R = 3.88 min (major), t_R = 4.26 min (minor).

^{1}H and ^{13}C NMR spectra are provided below.

2.8 Radical Probe Experiments and Structral Confirmation of Cyclized Products

To gain insights into the mechanism and determine whether this stereo-convergent cross-coupling reaction proceeds via a radical intermediate, we studied the cross-coupling reaction using a radical probe substrate with a terminal alkene group, 2,3,3-trimethylbutan-2-yl 2-chlorohept-6-enoate, and methyl 4-bromobenzoate reagent under the standard conditions.

Scheme S1. Crossing-Coupling Reaction Using Radical Probe Substrate (R = 2,3,3-trimethylbut-2-yl)

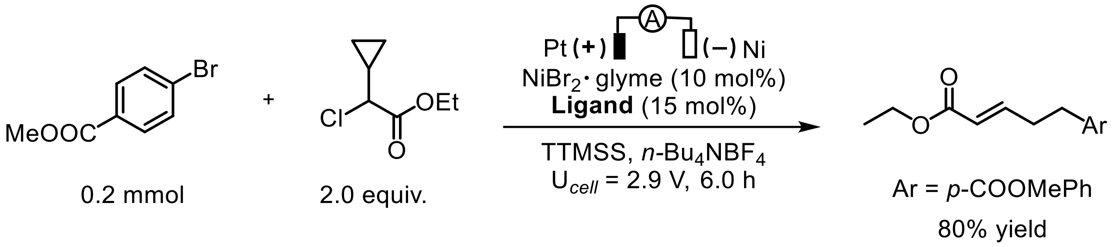

This substrate provided the direct (uncyclized) cross-coupling product in 30% yield with 91% ee, along with the cyclized cross-coupling product in 61% yield, as racemic mixtures of diastereomers.

The formation of cyclized products strongly suggests that the nickel-catalyzed enantioselective cross-coupling reactions take place via alkyl radical intermediates, as proposed for previous achiral cross-coupling reactions catalyzed by nickel catalyst.

For a related mechanistic study using a nickel catalyst, see: (a) Breitenfeld, J.; Wodrich, M. D.; Hu, X. *Organometallics* **2014,** *33,* 5708. (b) Biswas, S.; Weix, D. J. *J. Am. Chem. Soc.* **2013,** *135,* 16192. (c) Schley, N. D.; Fu, G. C. *J. Am. Chem. Soc.* **2014**, *136*, 16558.

Scheme S2. Crossing-Coupling Reaction Using Radical Probe Substrate (ethyl 2-chloro-2-cyclopropylacetate)

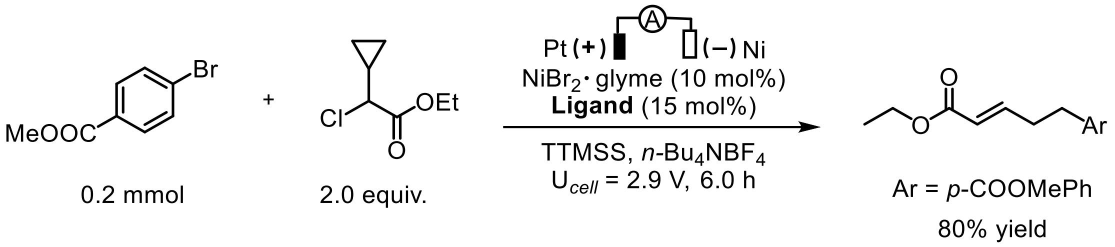

We also studied the cross-coupling reaction using a radical probe substrate with ethyl 2-chloro-2-cyclopropylacetate, and methyl 4-bromobenzoate reagent under the standard conditions.

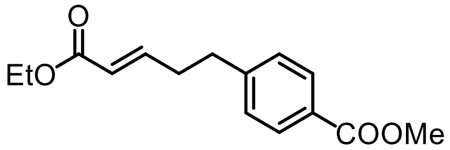

Methyl (*E*)-4-(5-ethoxy-5-oxopent-3-en-1-yl)benzoate (8)

A colorless oil (43.0 mg, 80% yield) from methyl 4-bromobenzoate (43.0 mg, 0.2 mmol).

^{1}H NMR (400 MHz, CDCl$_3$) δ 8.00 – 7.94 (m, 2H), 7.25 – 7.22 (m, 2H), 6.96 (dt, *J* = 15.6, 6.8 Hz, 1H), 5.82 (dt, *J* = 15.6, 1.6 Hz, 1H), 4.17 (q, *J* = 7.2 Hz, 2H), 3.89 (s, 3H), 2.82 (t, *J* = 7.6 Hz, 2H), 2.56 – 2.49 (m, 2H), 1.27 (t, *J* = 7.2 Hz, 3H).

^{13}C NMR (101 MHz, CDCl$_3$) δ 167.0, 166.4, 147.2, 146.1, 129.8, 128.3, 128.2, 122.2, 60.2, 52.0, 34.3, 33.3, 14.2.

IR (neat): 2950, 1713, 1653, 1435, 1274, 1178, 1104, 972, 857, 764, 704 cm^{-1}.

HRMS (ESI) calcd for $C_{15}H_{19}O_4Na_1$ $[M+H]^+$: 263.1278, found: 263.1277.

^{1}H and ^{13}C NMR spectra are provided below.

2.9 EPR Experiments and Data

Electron paramagnetic resonance (EPR) spectra were recorded on a Bruker ELEXSYS E500 spectrometer (X-bond). The spectra were measured at 298 K. The spectra were listed in below Figure.

General procedure:

In a nitrogen-filled glove box, an over-dried 10 mL hydrogenation tube charged with a stir bar, 2,3,3-trimethylbutan-2-yl 2-chloropropanoate (90.0 mg, 0.4 mmol), **solution A** (1.0 mL), $TBABF_4$ (66.0 mg, 0.2 mmol) were added to the electrochemical cell and the mixture was stirred for over 15 mins. Then methyl 4-bromobenzoate **1a** (0.2 mmol, 43.0 mg), 2.6-lutidine (64.0 mg, 0.6 mmol), tris(trimethylsilyl)silane (175.0 mg, 0.70 mmol), 5,5-Dimethyl-1-pyrroline N-oxide (DMPO, 25 mg, 0.2 mmol), DMAc (1.0 mL) were added to the electrochemical cell.

The tube was installed with a Ni foam (2.0 x 3.0 cm^2) as the cathode and Pt (1.0 x 1.0 x 0.3 cm^3) as the anode. The seal-tube was sealed and removed from the glovebox.

The reaction mixture was electrolyzed under a constant current of 6.0 mA.

The examples for EPR detection were collected once per hour during the reaction.

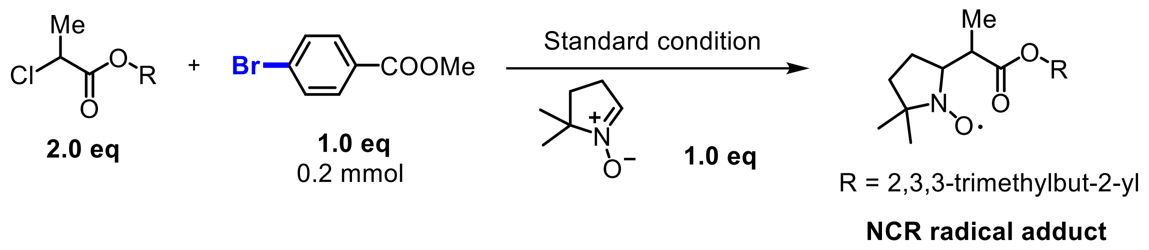

EPR spectra: (1.0 hour):

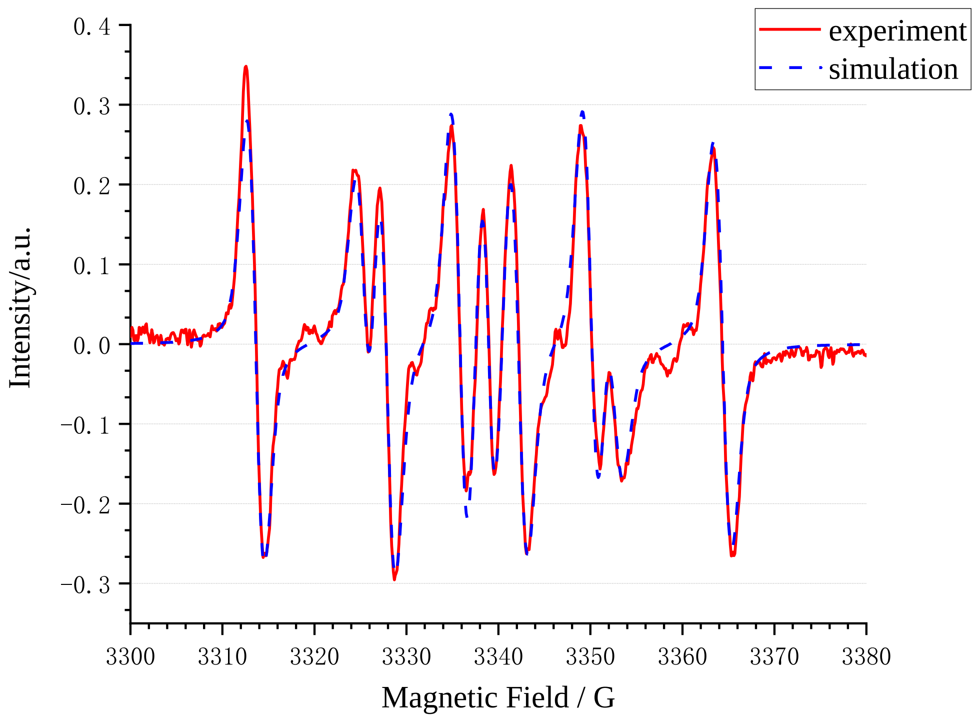

Red: the experiment spectra of NCR radical adduct.

(g = 2.0052, A_H = 14.29 G, A_N = 22.17 G)

Blue: Simulated spectra of NCR radical adduct.

3. Supplementary Figures

3.1 X-Ray Structures and Data

Crystals suitable for X-ray single-crystal diffraction analysis were obtained from Hexane using slow evaporation under air at room temperature.

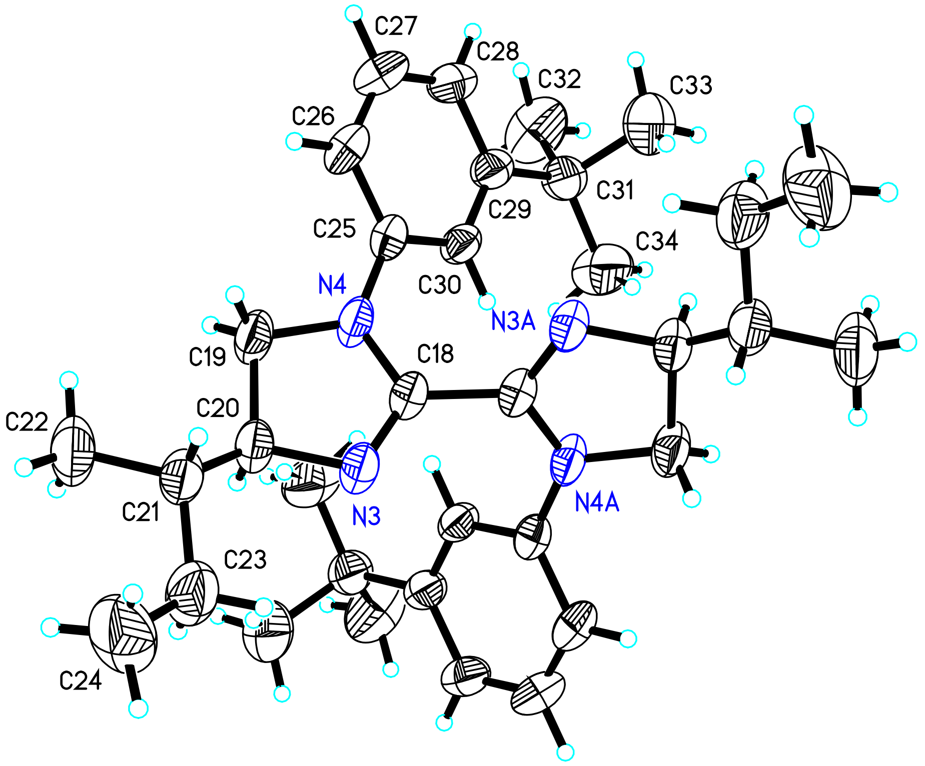

CCDC 2115366

Table 1. Crystal data and structure refinement for **Ligand L11**.

Identification code	mo_d8v21307_0m	
Empirical formula	C34 H50 N4	
Formula weight	514.78	
Temperature	293(2) K	
Wavelength	0.71073 Å	
Crystal system	Monoclinic	
Space group	C 2	
Unit cell dimensions	a = 23.9660(19) Å	= 90°.

	b = 10.9075(7) Å = 98.019(2)°.
	c = 12.8038(9) Å = 90°.
Volume	3314.3(4) Å^3
Z	4
Density (calculated)	1.032 Mg/m^3
Absorption coefficient	0.060 mm^{-1}
F(000)	1128
Crystal size	0.200 x 0.140 x 0.050 mm^3
Theta range for data collection	1.606 to 25.493°.
Index ranges	-28<=h<=28, -13<=k<=13, -15<=l<=15
Reflections collected	22844
Independent reflections	6155 [R(int) = 0.0753]
Completeness to theta = 25.242°	99.6 %
Absorption correction	Semi-empirical from equivalents
Max. and min. transmission	0.7456 and 0.4237
Refinement method	Full-matrix least-squares on F^2
Data / restraints / parameters	6155 / 73 / 414
Goodness-of-fit on F^2	1.025
Final R indices [I>2sigma(I)]	R1 = 0.0746, wR2 = 0.2036
R indices (all data)	R1 = 0.1094, wR2 = 0.2414
Absolute structure parameter	-0.8(10)
Extinction coefficient	0.022(6)
Largest diff. peak and hole	0.197 and -0.166 e.Å^{-3}

Crystals suitable for X-ray single-crystal diffraction analysis were obtained from Toluene/Dichloromethane using slow evaporation under air at room temperature.

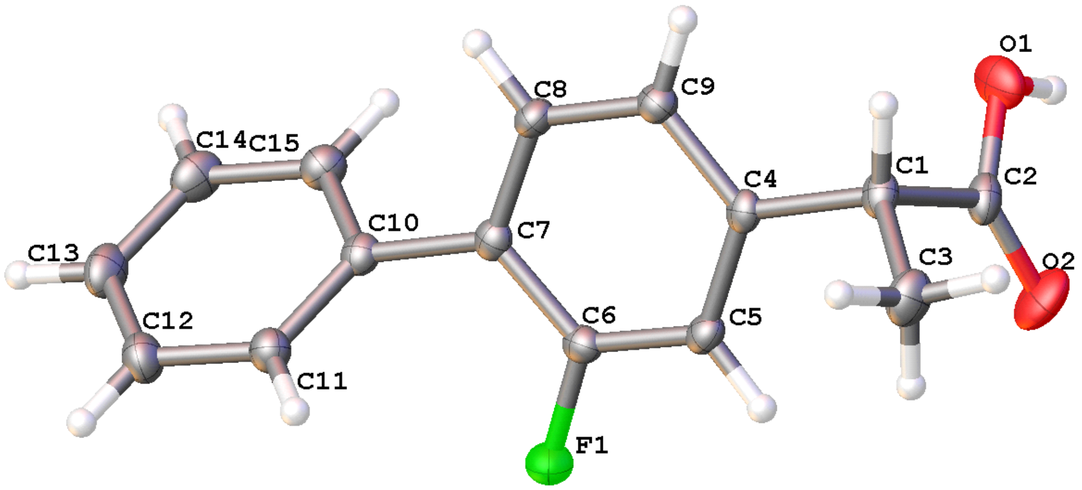

CCDC 931885

Table 2. Crystal data and structure refinement for **3**.

Identification code	mj21485_0m	
Empirical formula	C15 H13 F O2	
Formula weight	244.25	
Temperature	212.99 K	
Wavelength	1.34139 Å	
Crystal system	Orthorhombic	
Space group	$P2_12_12_1$	
Unit cell dimensions	a = 5.69140(10) Å	= 90°.
	b = 13.1880(2) Å	= 90°.
	c = 16.2085(3) Å	= 90°.
Volume	1216.58(4) $Å^3$	
Z	4	
Density (calculated)	1.334 Mg/m^3	
Absorption coefficient	0.516 mm^{-1}	
F(000)	512	
Crystal size	0.15 x 0.08 x 0.07 mm^3	
Theta range for data collection	3.759 to 54.906°.	
Index ranges	-6<=h<=6, -16<=k<=12, -19<=l<=19	
Reflections collected	12382	

Independent reflections	2246 [R(int) = 0.0301]
Completeness to theta = 53.594°	97.2 %
Absorption correction	Semi-empirical from equivalents
Max. and min. transmission	0.7508 and 0.6124
Refinement method	Full-matrix least-squares on F^2
Data / restraints / parameters	2246 / 0 / 165
Goodness-of-fit on F^2	1.050
Final R indices [I>2sigma(I)]	R1 = 0.0341, wR2 = 0.0986
R indices (all data)	R1 = 0.0346, wR2 = 0.0991
Absolute structure parameter	-0.01(5)
Extinction coefficient	n/a
Largest diff. peak and hole	0.449 and -0.188 e.Å^{-3}

3.2 NMR Spectra and Chromatograms

Supplementary Figure 6. ^{1}H NMR Spectrum of 2,3,3-trimethylbutan-2-yl 2-chloropropanoate (400 MHz, $CDCl_3$)

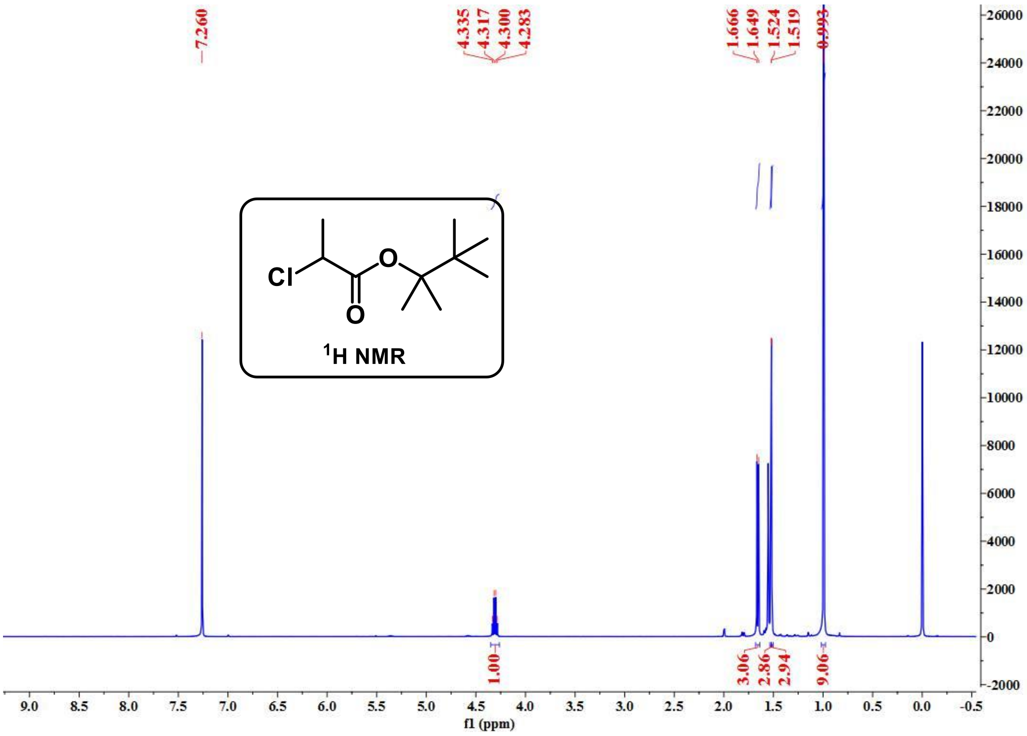

Supplementary Figure 7. ^{1}H NMR Spectrum of 2,3,3-trimethylbutan-2-yl 3-(benzyloxy)-2-chloropropanoate (400 MHz, $CDCl_3$)

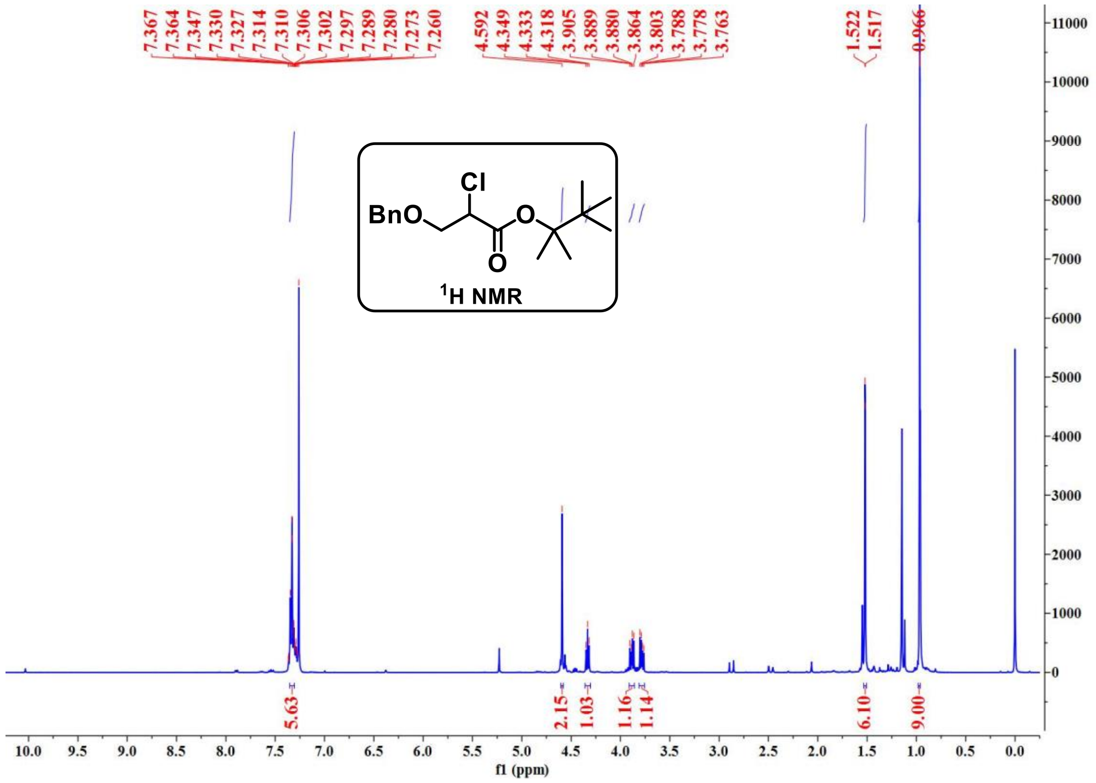

Supplementary Figure 8. ^{13}C NMR Spectrum of 2,3,3-trimethylbutan-2-yl 3-(benzyloxy)-2-chloropropanoate (101 MHz, $CDCl_3$)

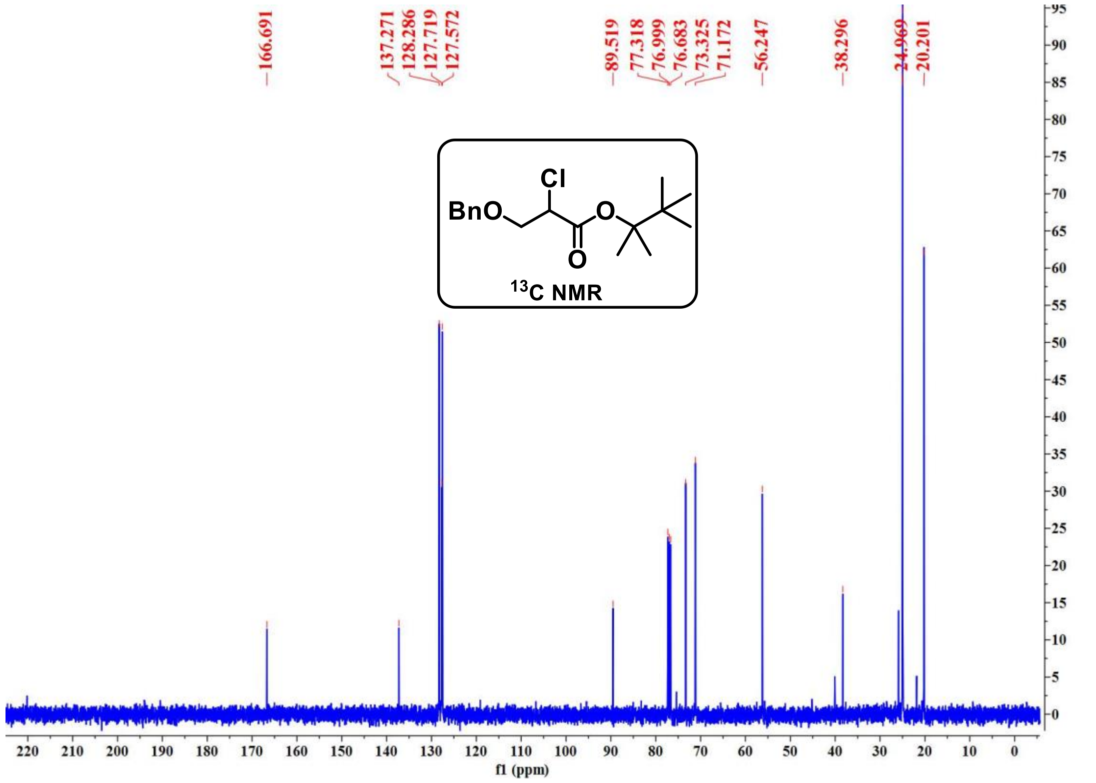

Supplementary Figure 9. ^{1}H NMR Spectrum of 2,3,3-trimethylbutan-2-yl 2-chloro-3-(4-methoxyphenyl)propanoate (400 MHz, $CDCl_3$)

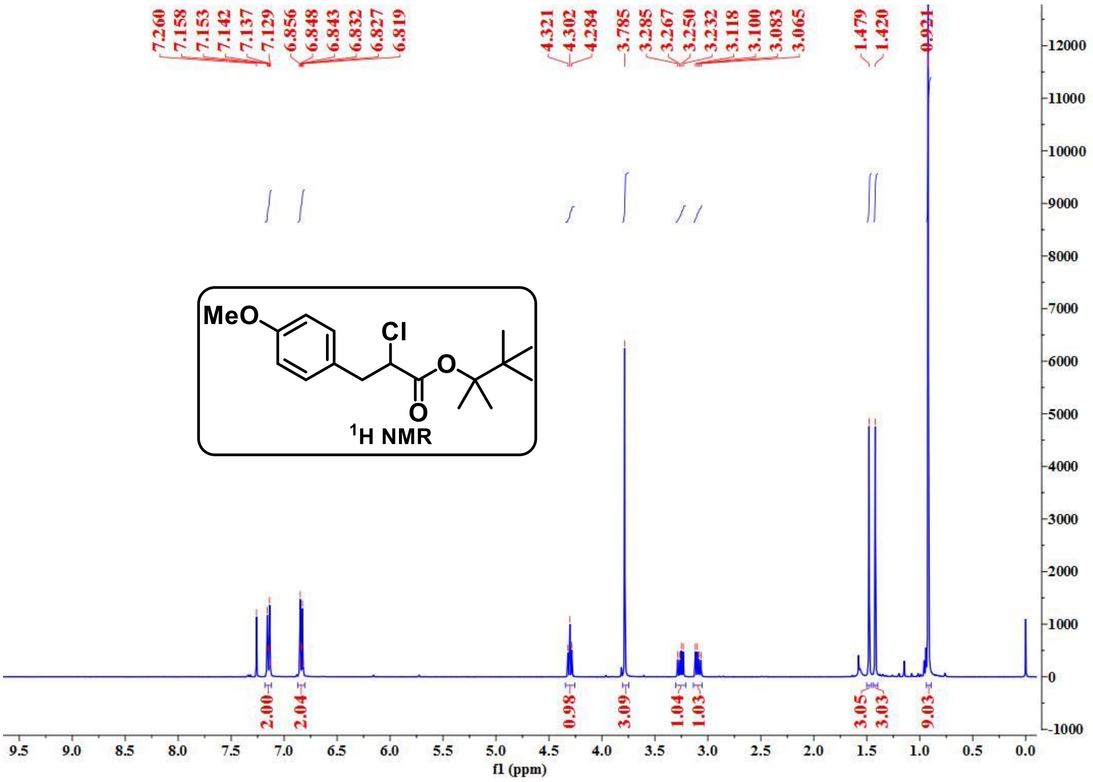

Supplementary Figure 10. ^{13}C NMR Spectrum of 2,3,3-trimethylbutan-2-yl 2-chloro-3-(4-methoxyphenyl)propanoate (101 MHz, $CDCl_3$)

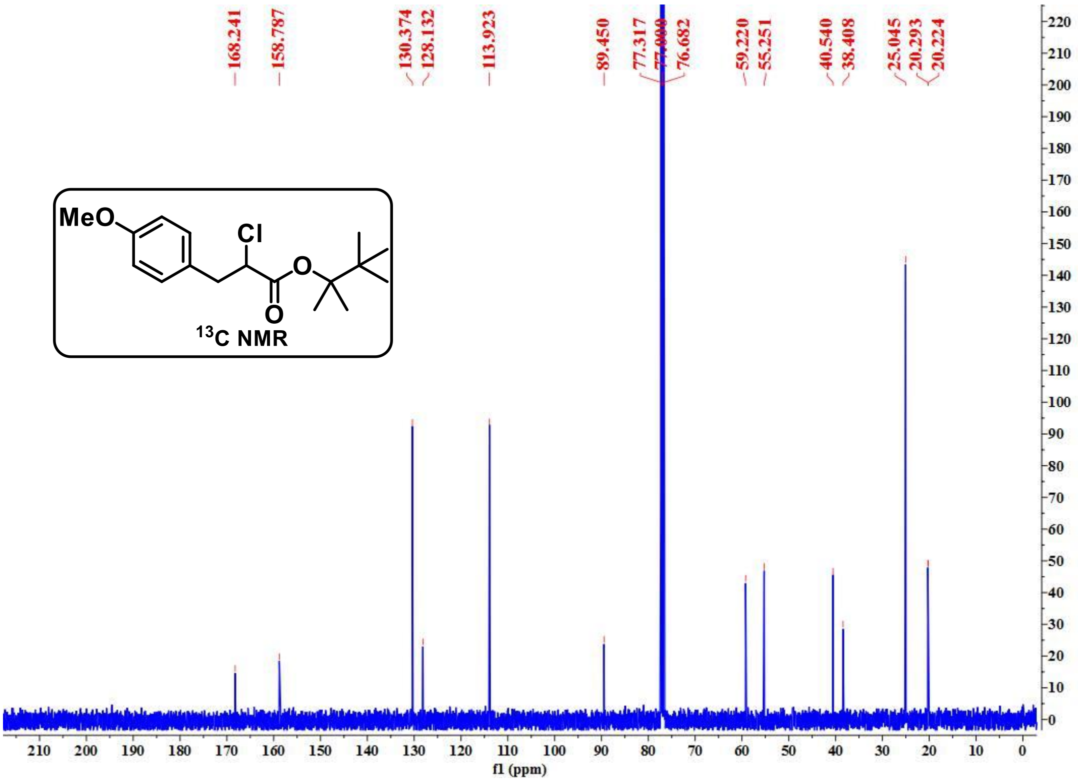

Supplementary Figure 11. ^{1}**H NMR Spectrum of 2,3,3-trimethylbutan-2-yl 2-chloro-3-phenylpropanoate (400 MHz, $CDCl_3$)**

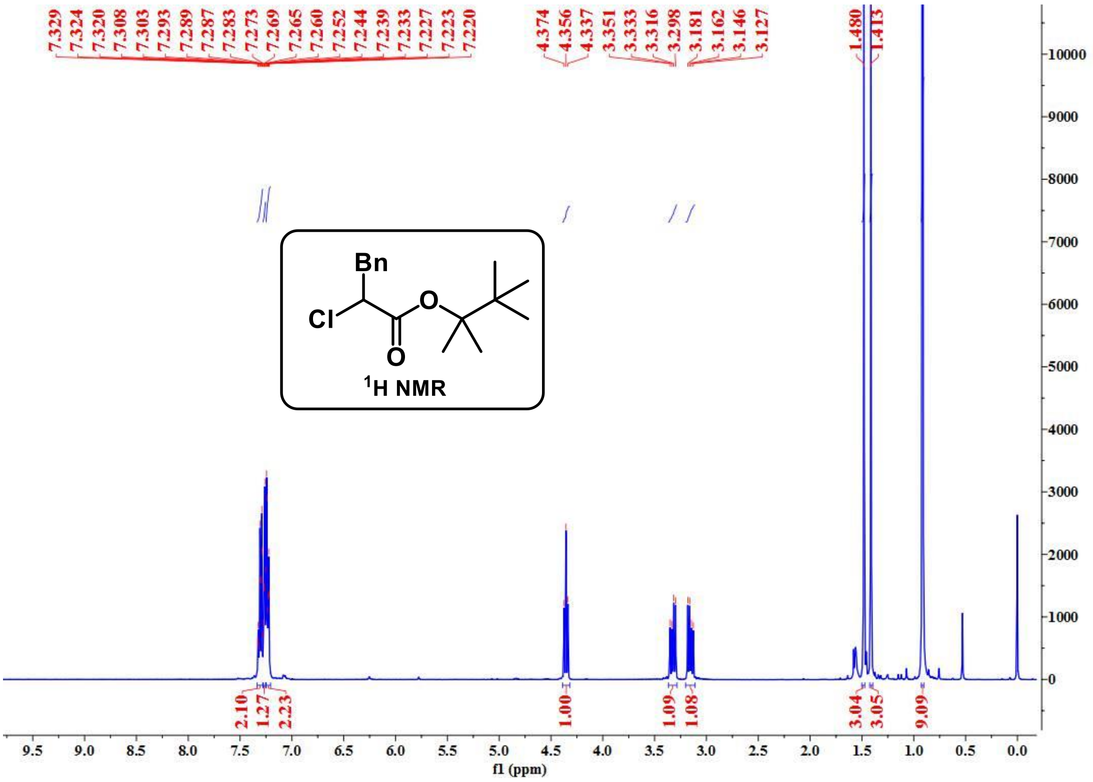

Supplementary Figure 12. ^{13}**C NMR Spectrum of 2,3,3-trimethylbutan-2-yl 2-chloro-3-phenylpropanoate (101 MHz, $CDCl_3$)**

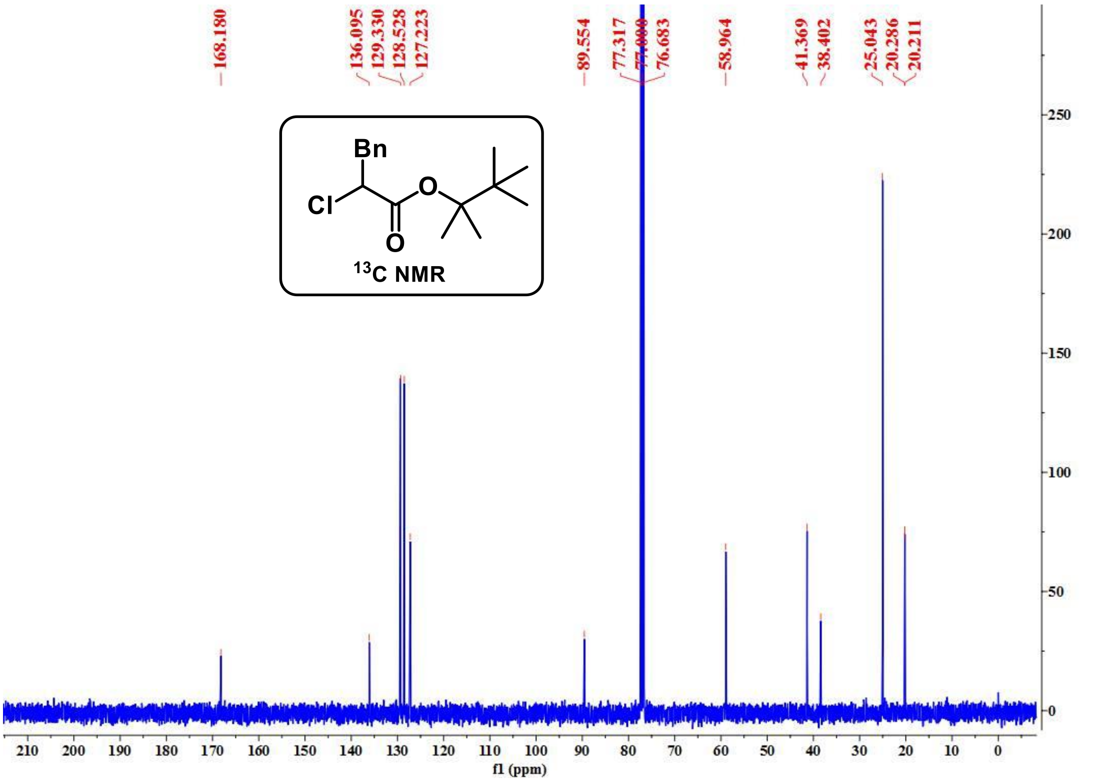

Supplementary Figure 13. ^{1}H **NMR Spectrum of 2,3,3-trimethylbutan-2-yl 2-chloro-4-phenylbutanoate (400 MHz, $CDCl_3$)**

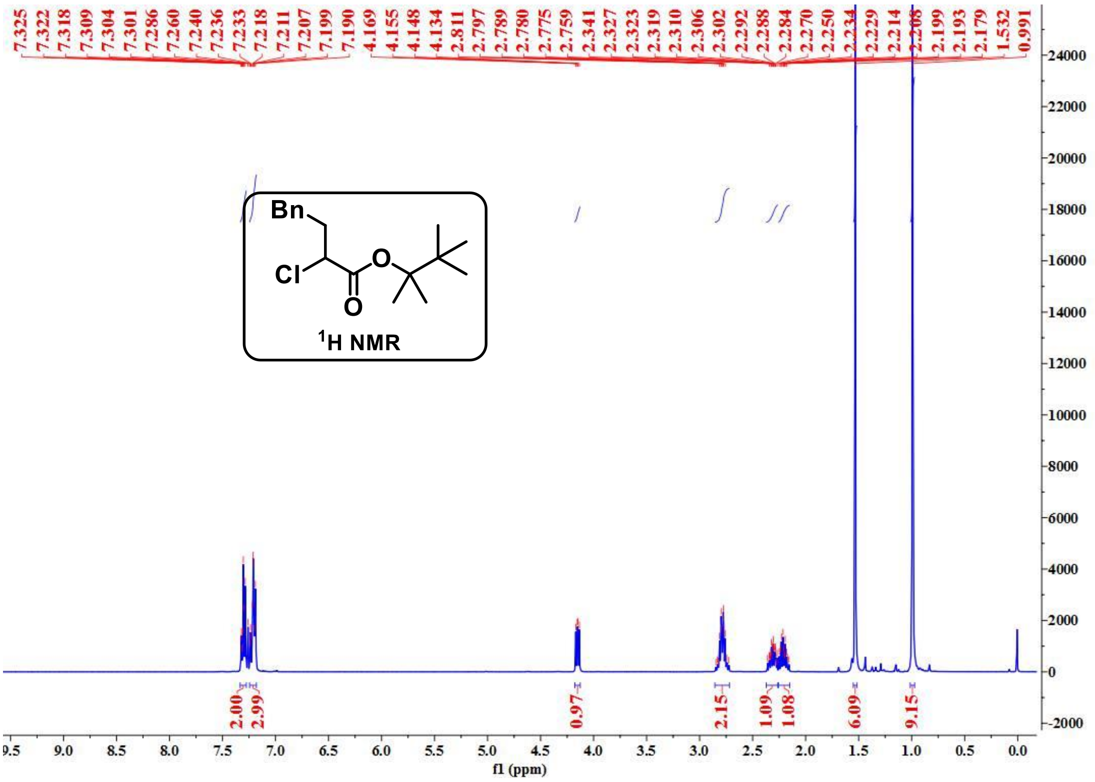

Supplementary Figure 14. ^{13}C **NMR Spectrum of 2,3,3-trimethylbutan-2-yl 2-chloro-4-phenylbutanoate (101 MHz, $CDCl_3$)**

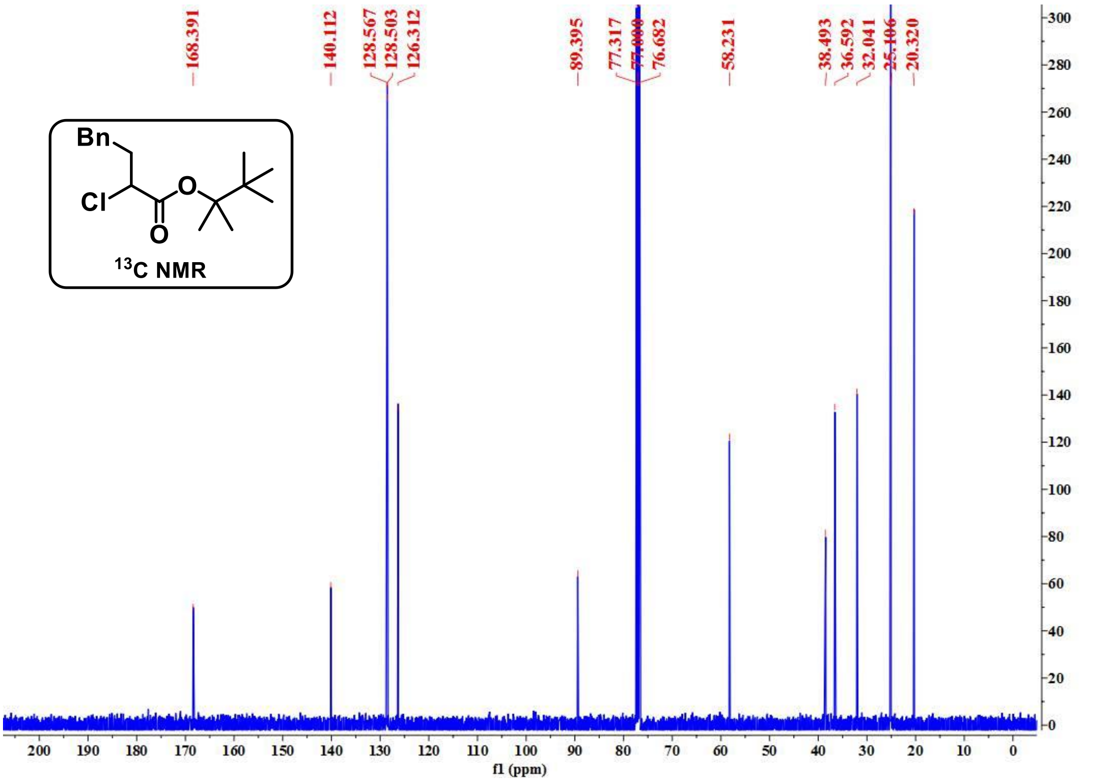

Supplementary Figure 15. ^{1}H **NMR Spectrum of 2,3,3-trimethylbutan-2-yl 2-chloro-4-(methylthio)butanoate (400 MHz, CDCl₃)**

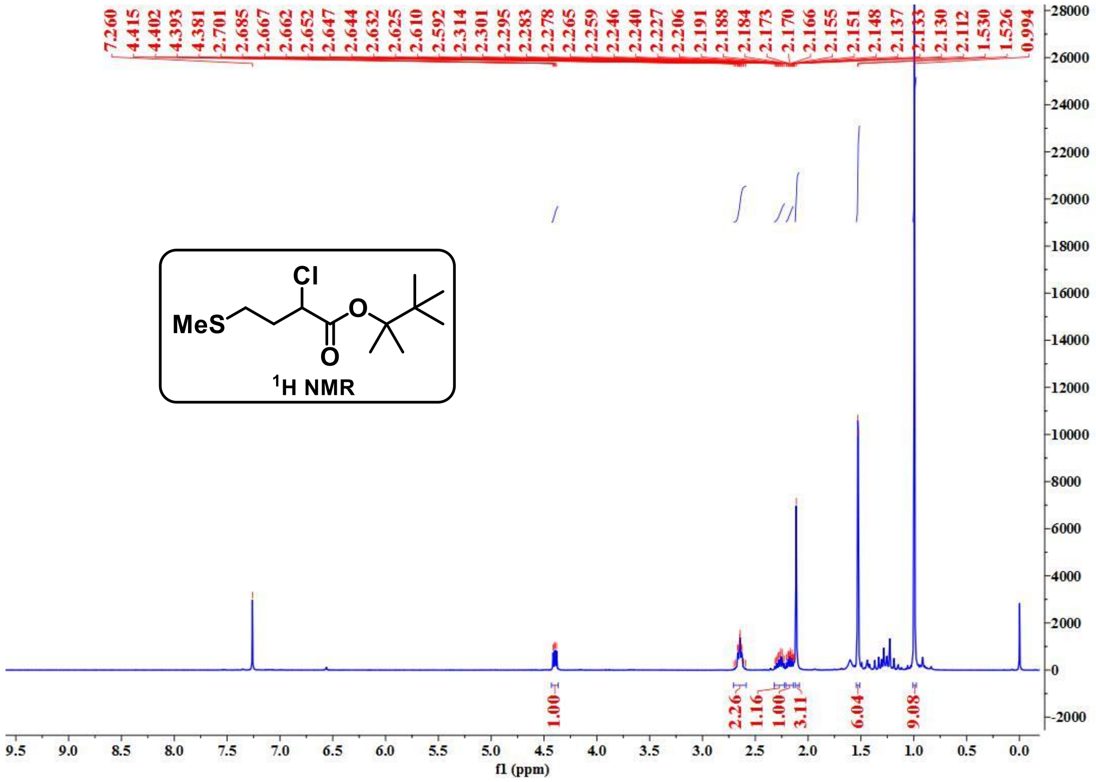

Supplementary Figure 16. ^{13}C **NMR Spectrum of 2,3,3-trimethylbutan-2-yl 2-chloro-4-(methylthio)butanoate (101 MHz, CDCl₃)**

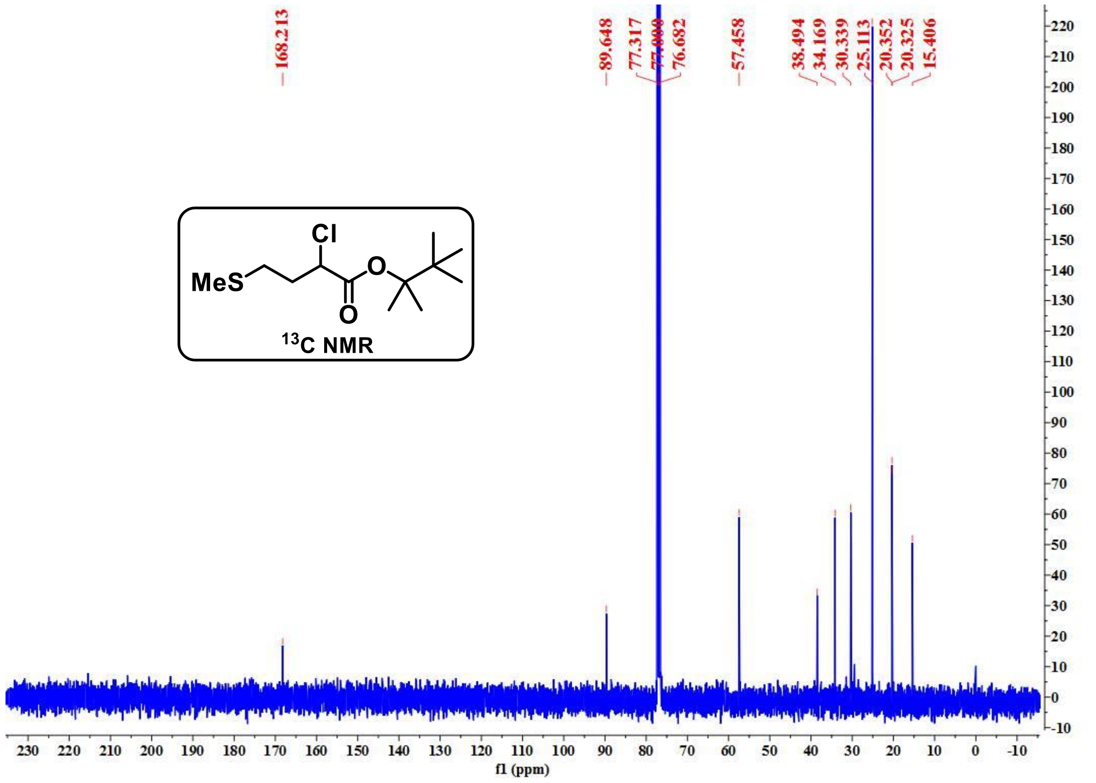

Supplementary Figure 17. ^{1}H NMR Spectrum of 2,3,3-trimethylbutan-2-yl 2-chlorohept-6-enoate (400 MHz, CDCl$_3$)

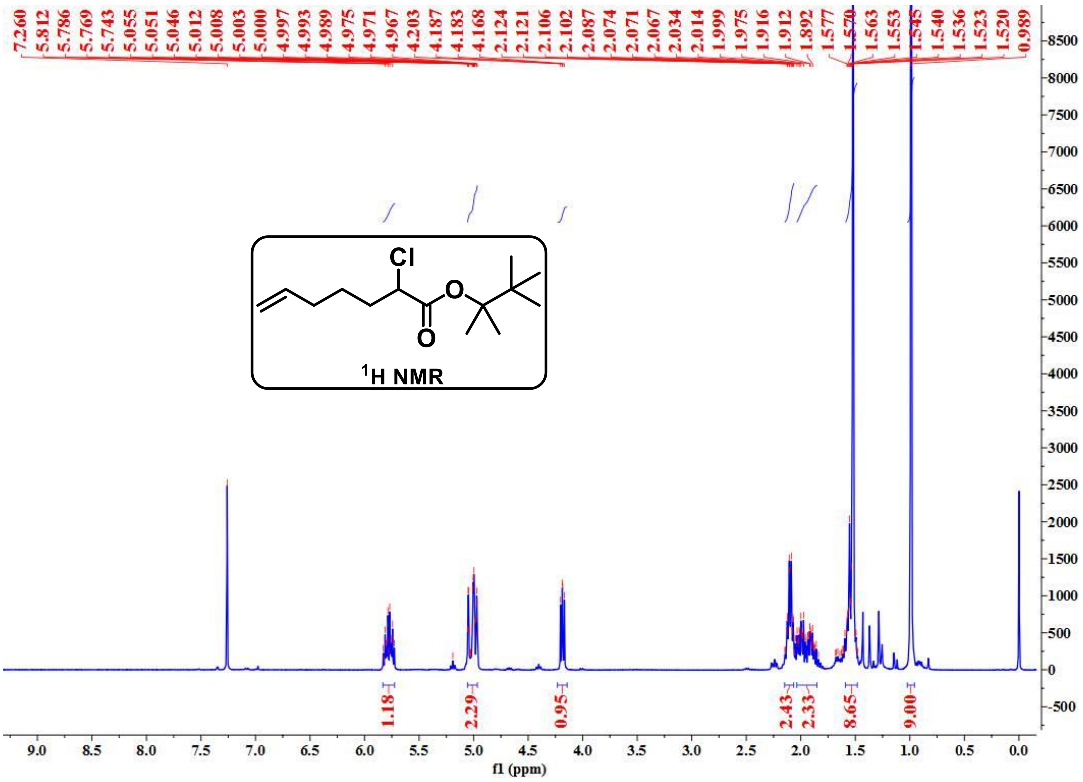

Supplementary Figure 18. ^{13}C NMR Spectrum of 2,3,3-trimethylbutan-2-yl 2-chlorohept-6-enoate (101 MHz, CDCl$_3$)

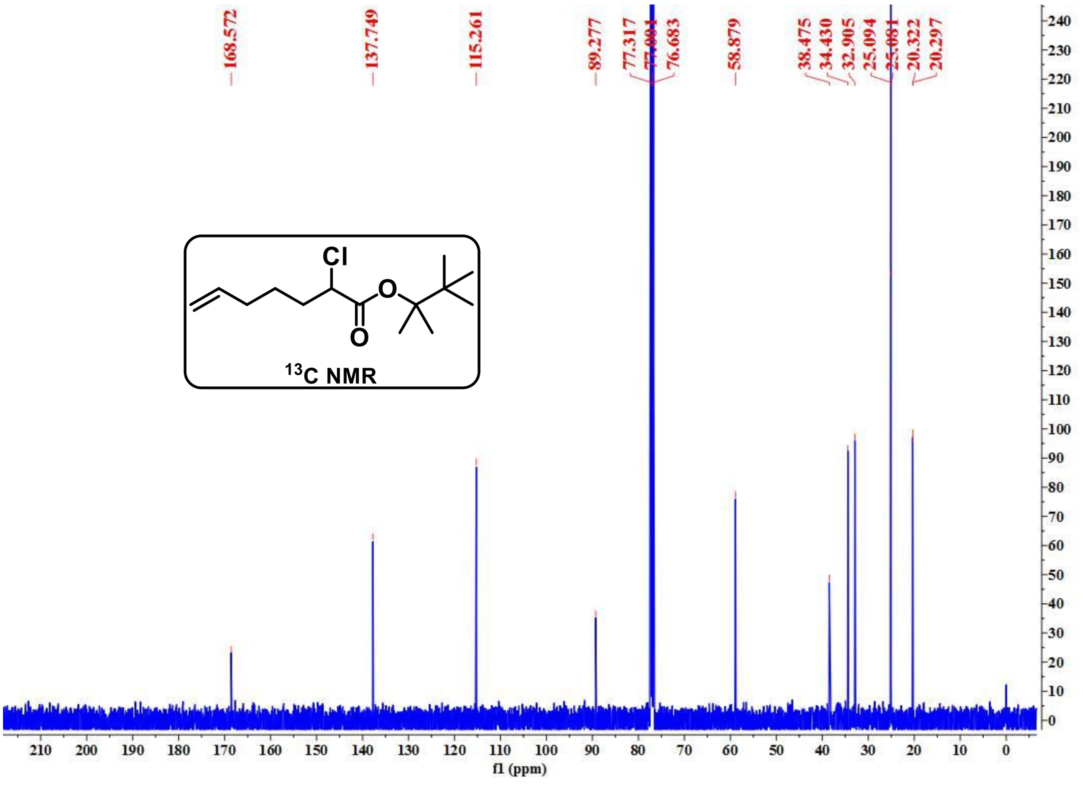

Supplementary Figure 19. ^{1}H NMR Spectrum of 2,3,3-trimethylbutan-2-yl 2-chlorobutanoate (400 MHz, CDCl$_3$)

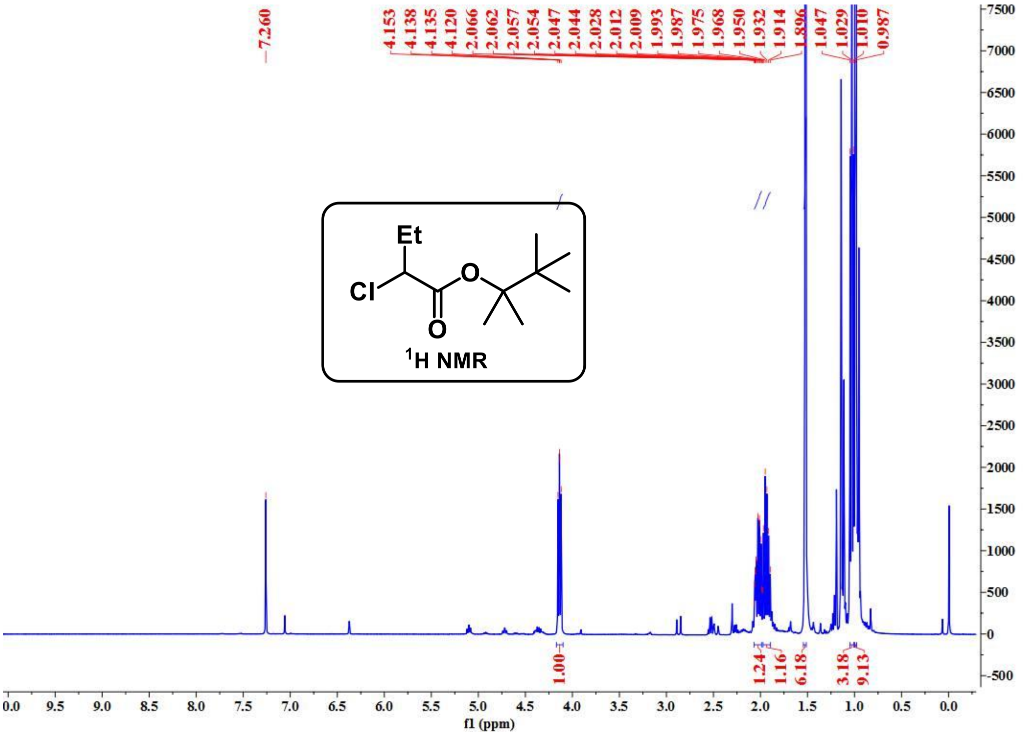

Supplementary Figure 20. ^{13}C NMR Spectrum of 2,3,3-trimethylbutan-2-yl 2-chlorobutanoate (101 MHz, CDCl$_3$)

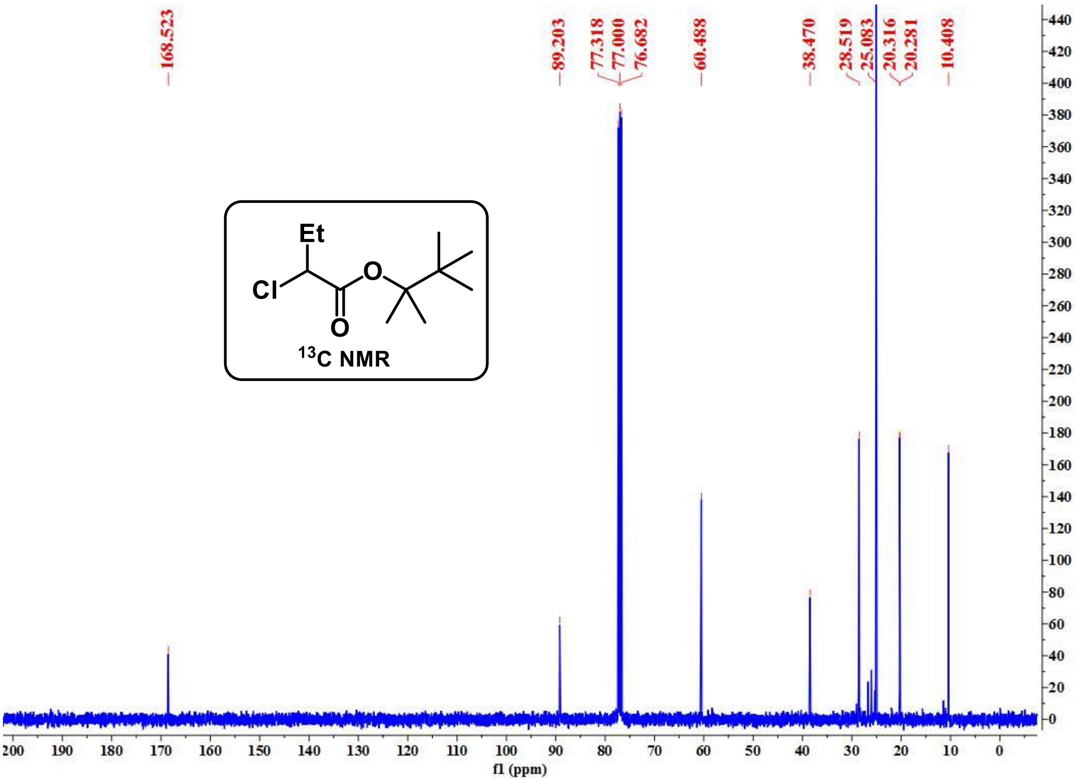

Supplementary Figure 21. ^{1}**H NMR Spectrum of 2,3,3-trimethylbutan-2-yl 2-chlorohexanoate (400 MHz, CDCl$_3$)**

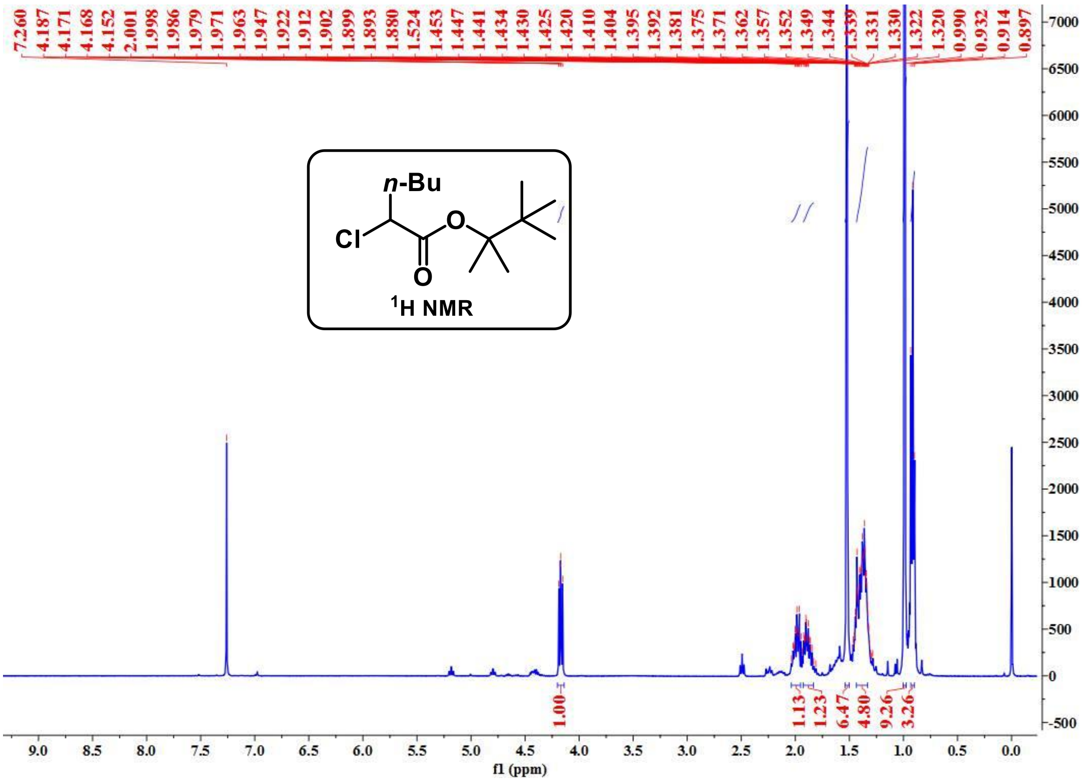

Supplementary Figure 22. ^{13}**C NMR Spectrum of 2,3,3-trimethylbutan-2-yl 2-chlorohexanoate (101 MHz, CDCl$_3$)**

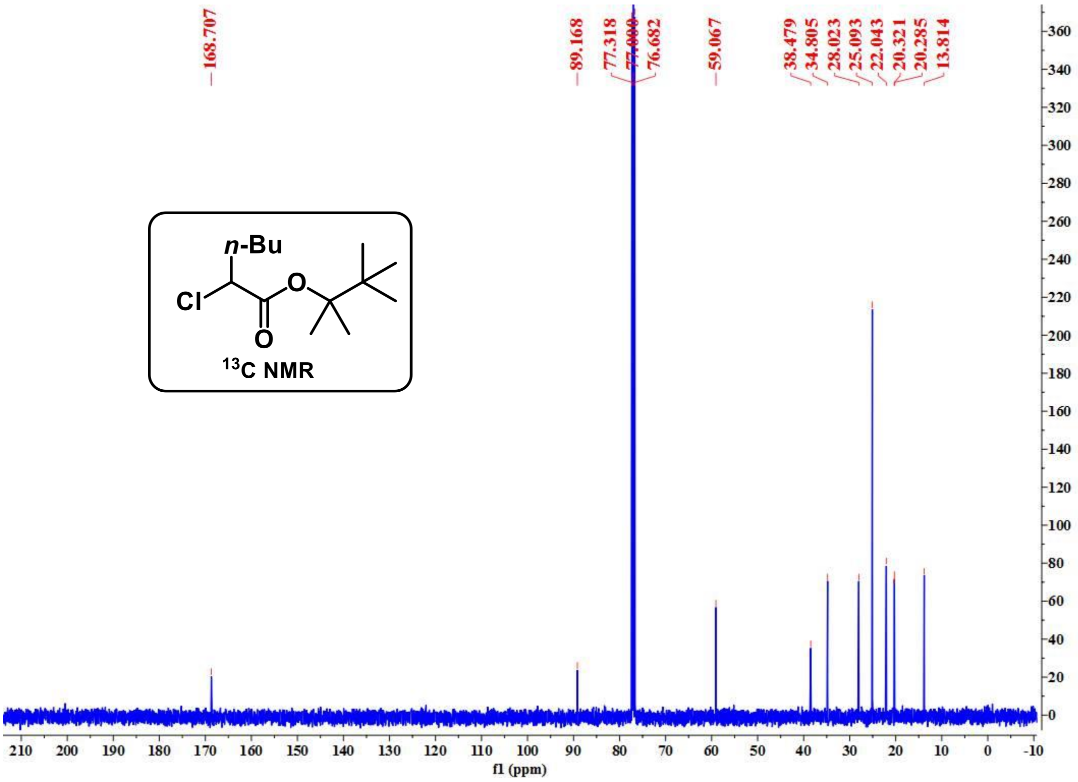

Supplementary Figure 23. ^{1}H NMR Spectrum of 2,3,3-trimethylbutan-2-yl 2-chloro-3-methylbutanoate (400 MHz, $CDCl_3$)

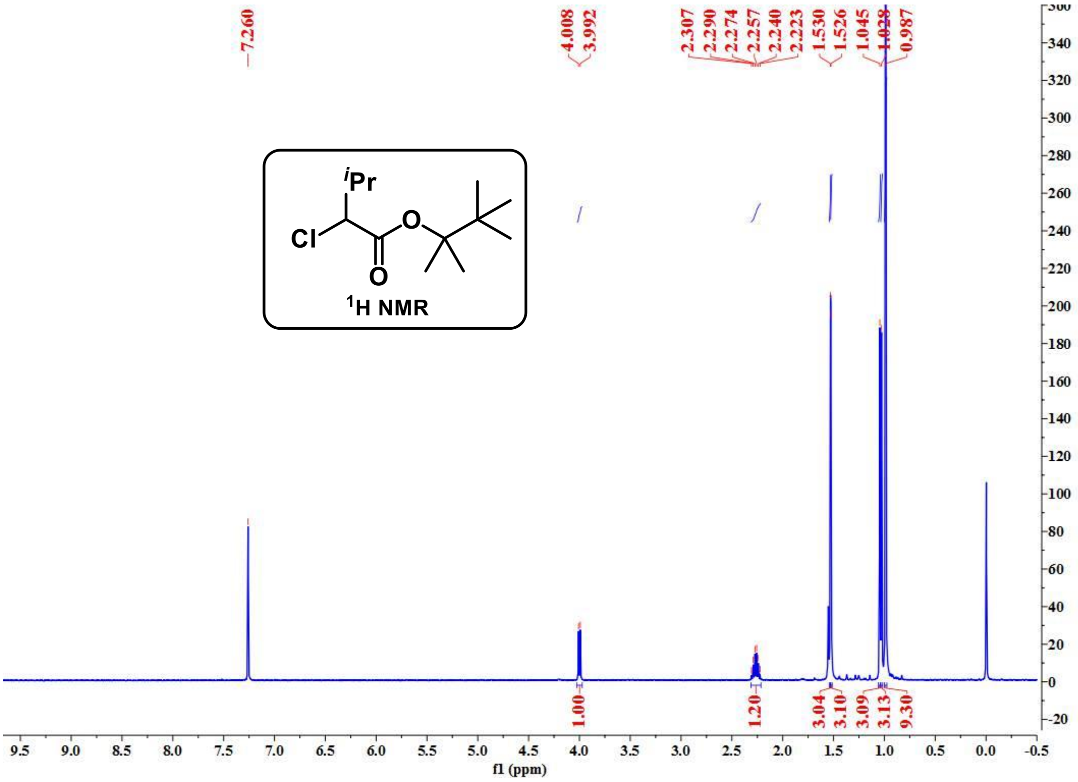

Supplementary Figure 24. ^{1}H NMR Spectrum of 2,3,3-trimethylbutan-2-yl 2-chloro-4-methylpentanoate (400 MHz, $CDCl_3$)

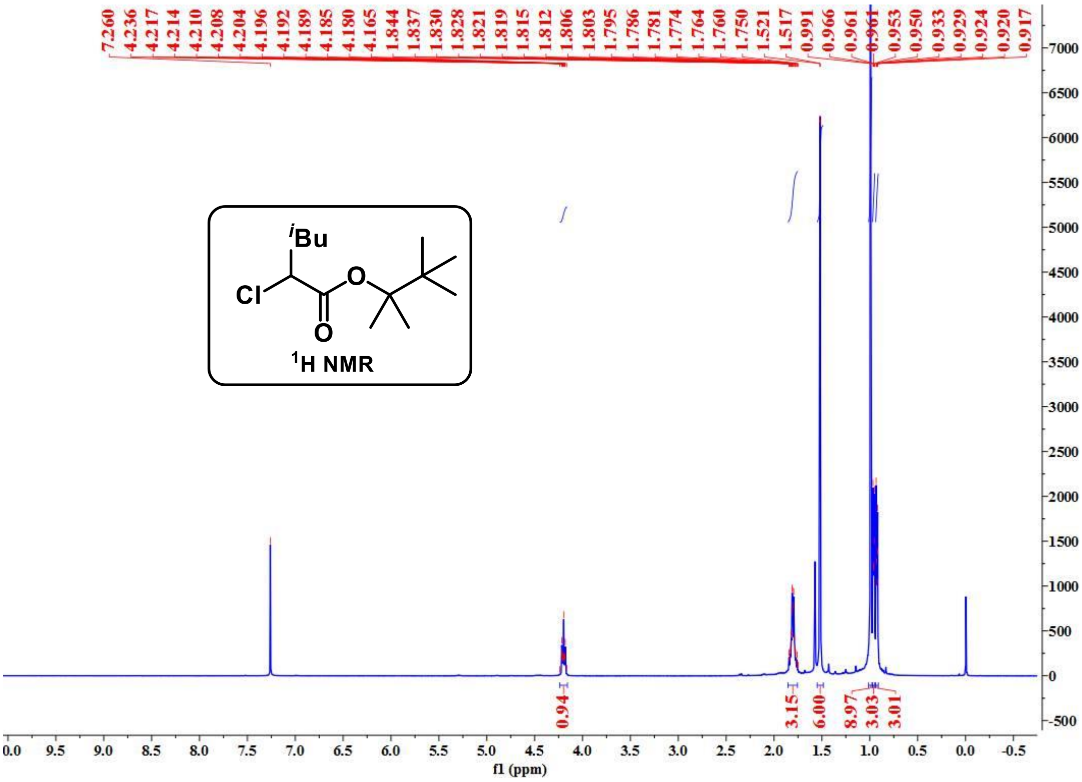

Supplementary Figure 25. ^{1}H NMR Spectrum of 2,3,3-trimethylbutan-2-yl 2-chloro-2-cyclohexylacetate (400 MHz, $CDCl_3$)

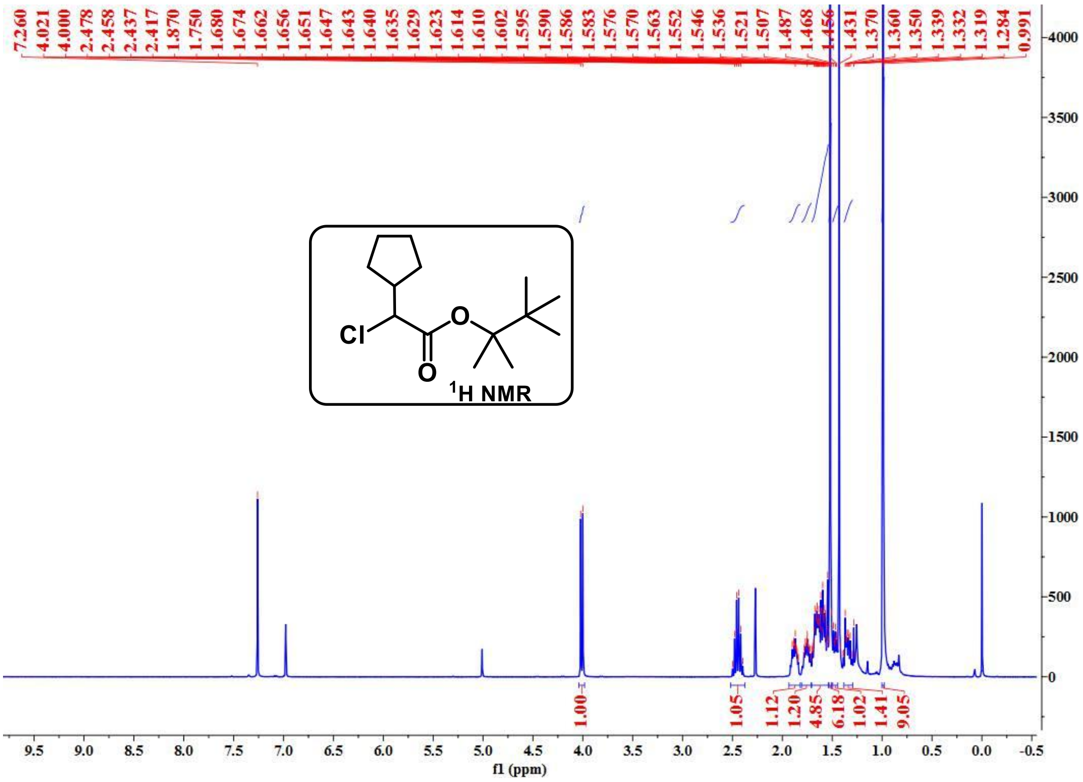

Supplementary Figure 26. ^{13}C NMR Spectrum of 2,3,3-trimethylbutan-2-yl 2-chloro-2-cyclohexylacetate (101 MHz, $CDCl_3$)

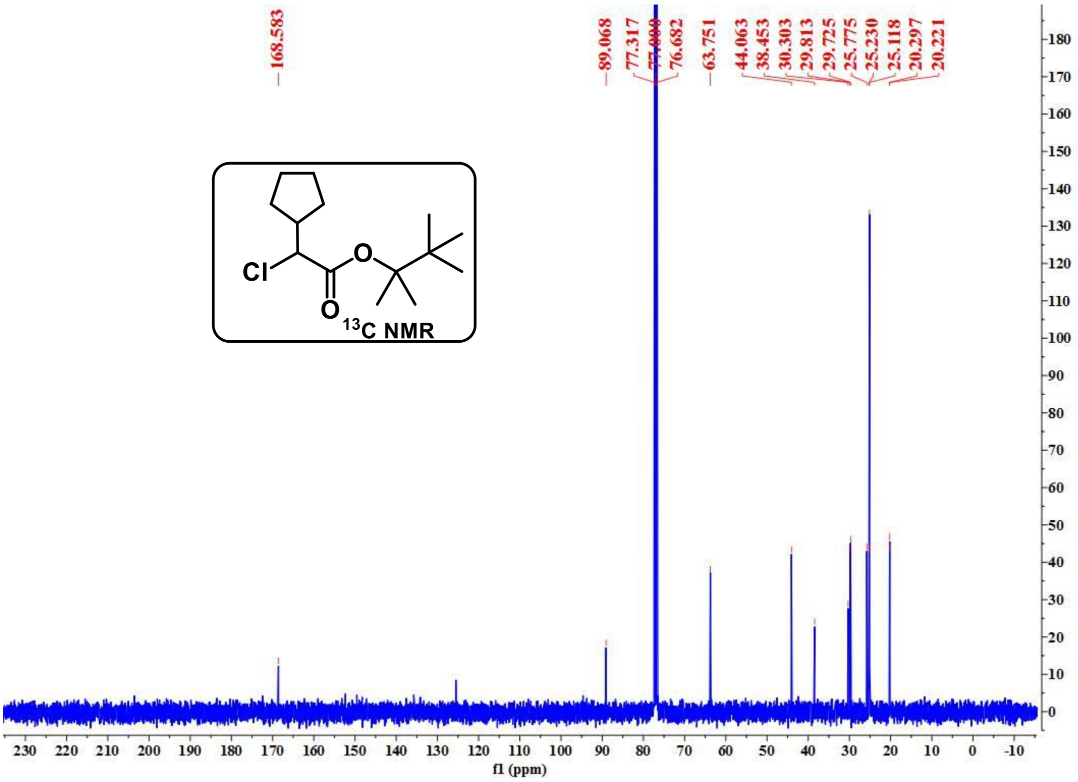

Supplementary Figure 27. ^{1}H NMR Spectrum of 2,3,3-trimethylbutan-2-yl 2-chloro-2-cyclohexylacetate (400 MHz, $CDCl_3$)

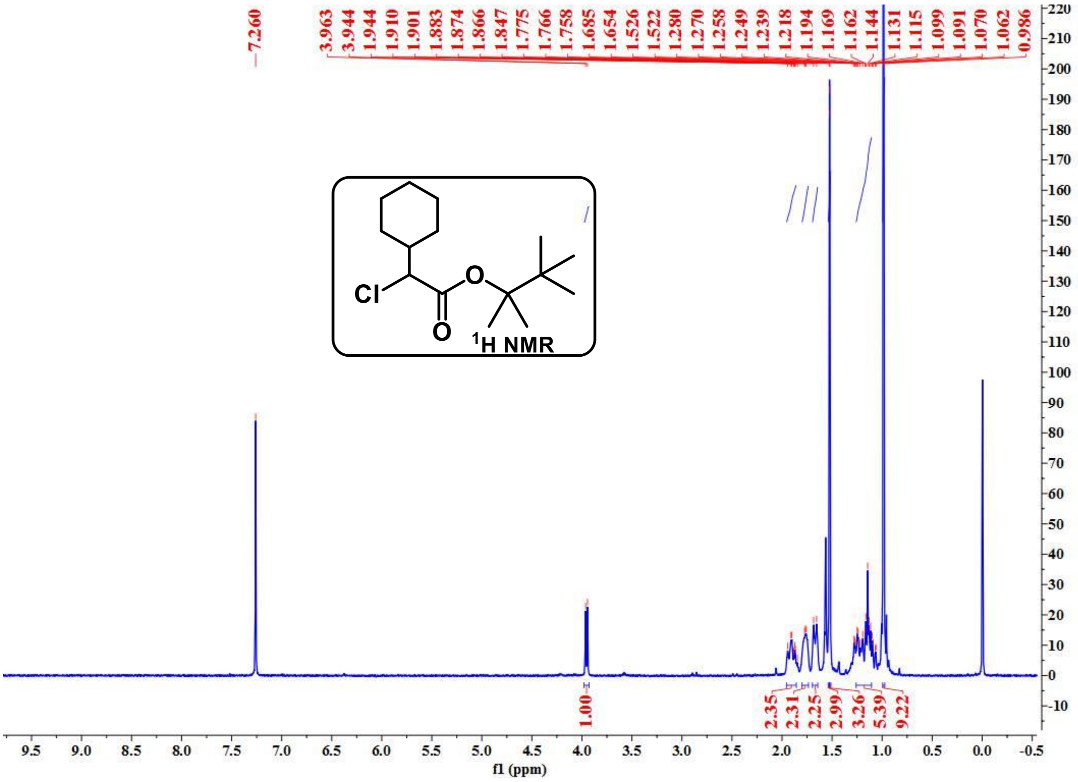

Supplementary Figure 28. ^{1}H NMR Spectrum of 2,3,3-trimethylbutan-2-yl 2-chloro-2-cyclohexylacetate (101 MHz, $CDCl_3$)

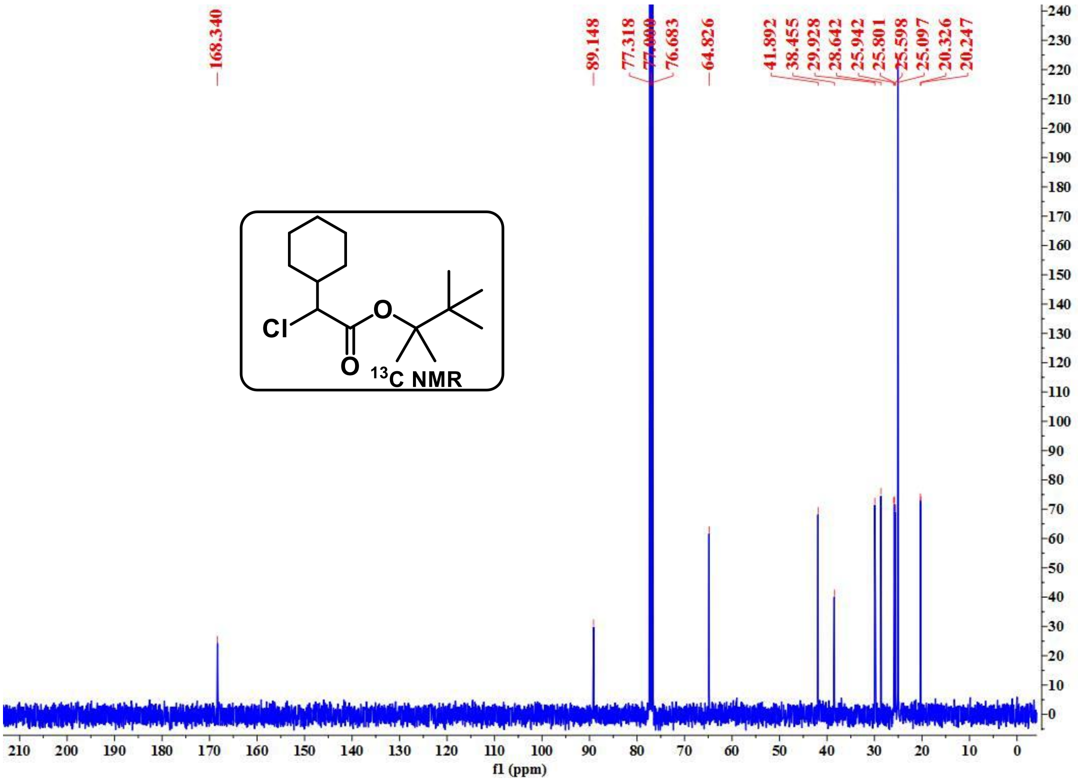

Supplementary Figure 29. ^{1}H NMR Spectrum of (1*R*,2*S*,5*R*)-2-isopropyl-5-methylcyclohexyl 2-chloropropanoate (400 MHz, $CDCl_3$)

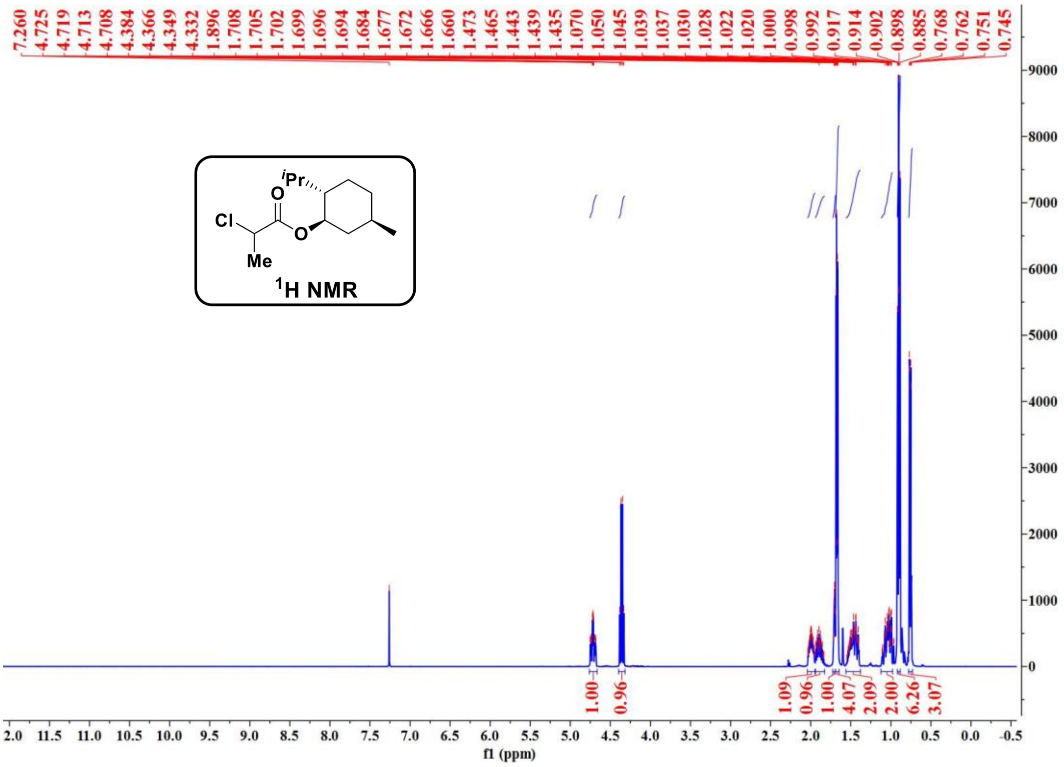

Supplementary Figure 30. ^{13}C NMR Spectrum of (1*R*,2*S*,5*R*)-2-isopropyl-5-methylcyclohexyl 2-chloropropanoate (400 MHz, $CDCl_3$)

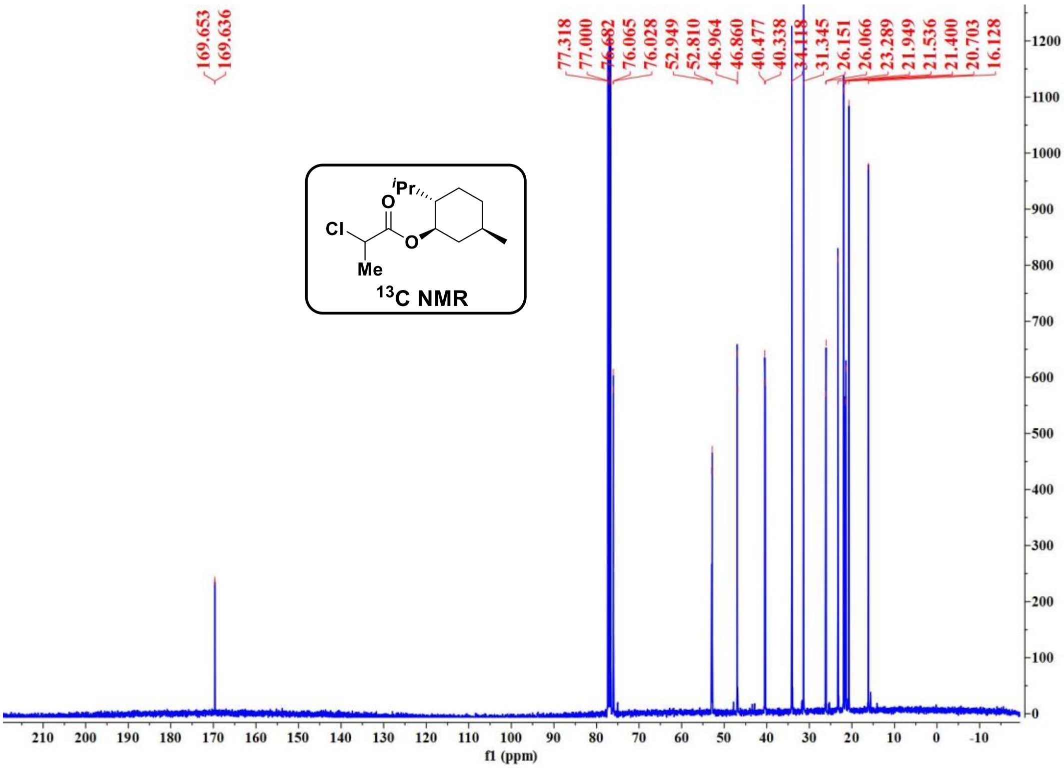

Supplementary Figure 31. ^{1}**H NMR Spectrum of Methyl (*R*)-4-(1-oxo-1-((2,3,3-trimethylbutan-2-yl)oxy)propan-2-yl)benzoate (400 MHz, CDCl**$_3$**)**

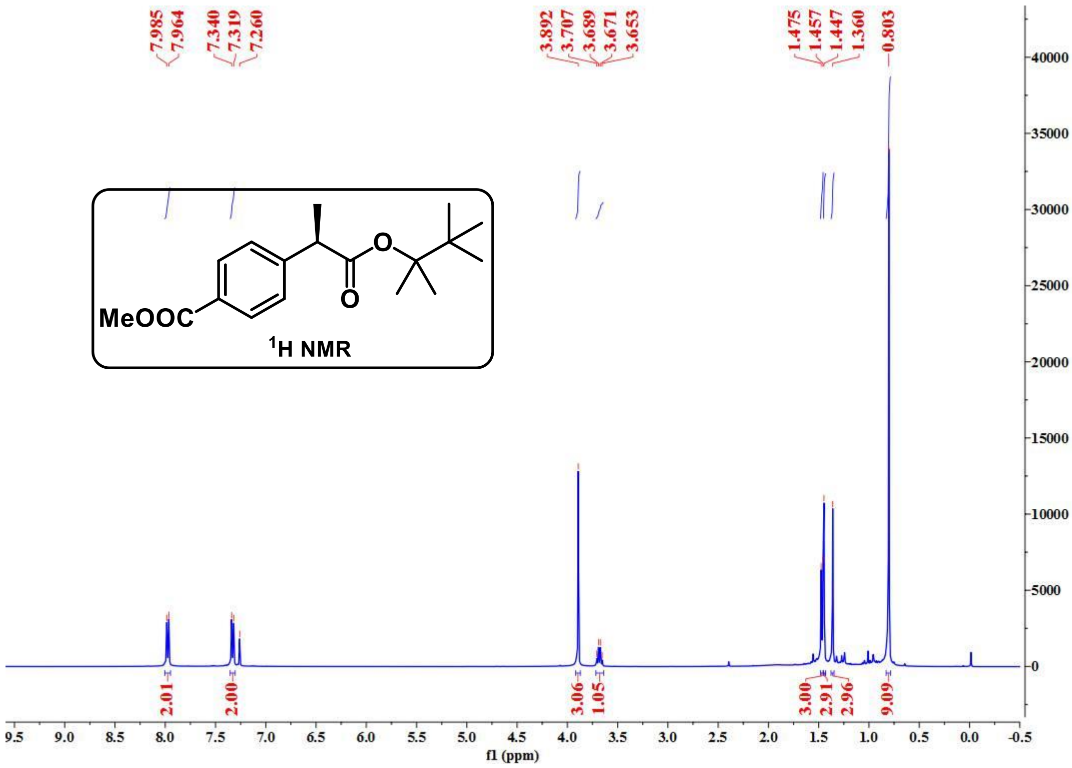

Supplementary Figure 32. ^{13}**C NMR Spectrum of Methyl (*R*)-4-(1-oxo-1-((2,3,3-trimethylbutan-2-yl)oxy)propan-2-yl)benzoate (101 MHz, CDCl**$_3$**)**

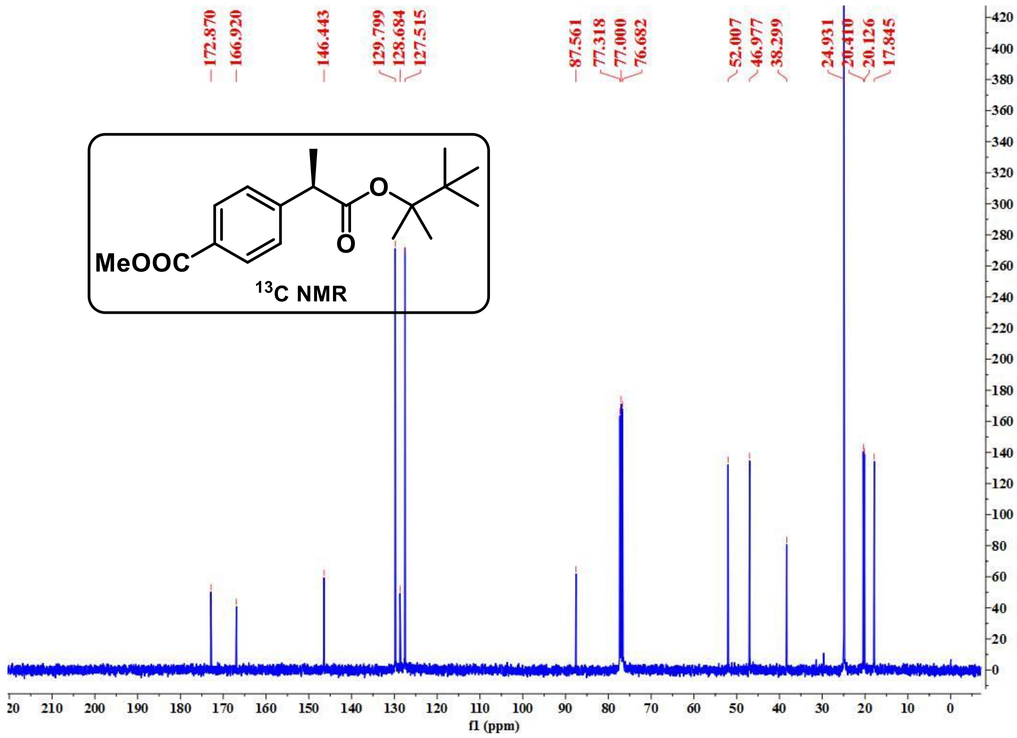

Supplementary Figure 33. ^{1}**H NMR Spectrum of Methyl (*R*)-3-(1-oxo-1-((2,3,3-trimethylbutan-2-yl)oxy)propan-2-yl)benzoate (400 MHz, CDCl$_3$)**

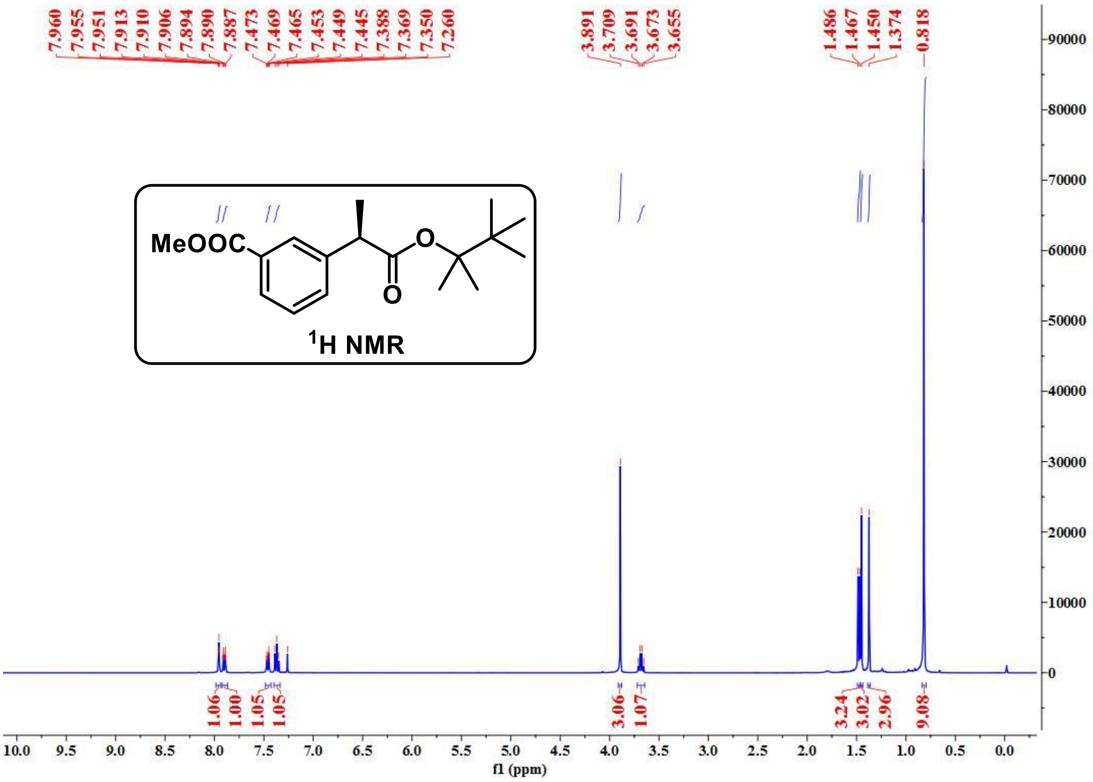

Supplementary Figure 34. ^{13}**C NMR Spectrum of Methyl (*R*)-3-(1-oxo-1-((2,3,3-trimethylbutan-2-yl)oxy)propan-2-yl)benzoate (101 MHz, CDCl$_3$)**

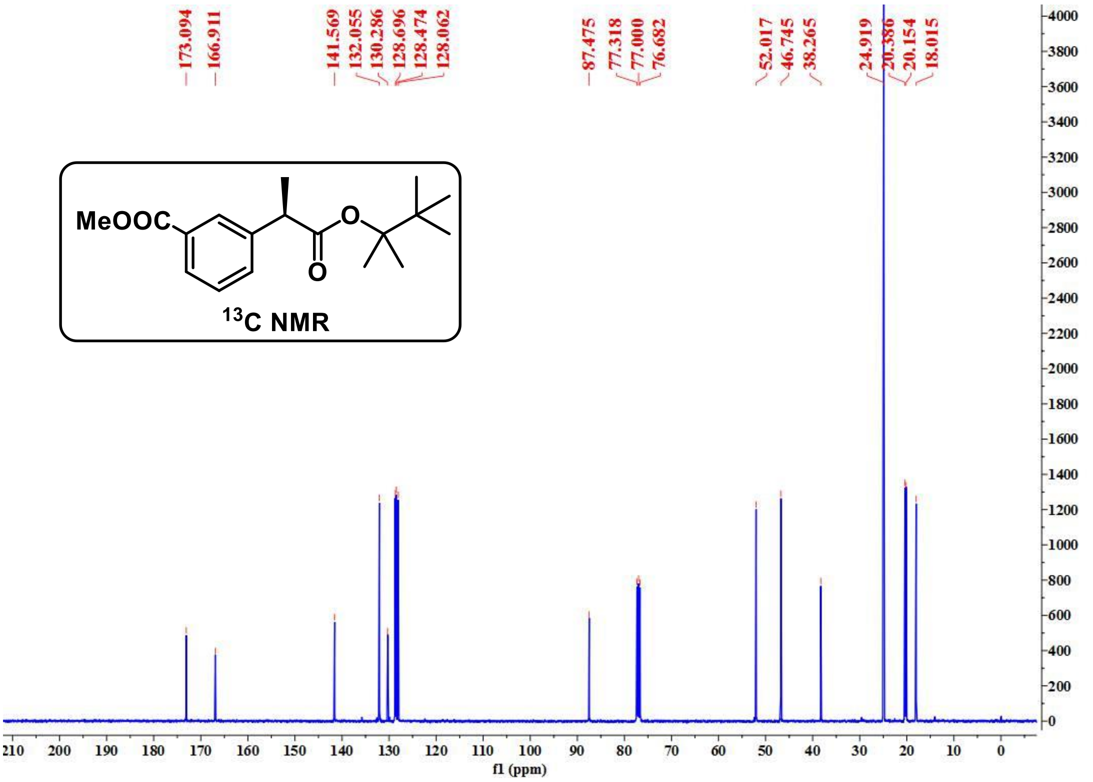

Supplementary Figure 35. ^{1}**H NMR Spectrum of Methyl (*R*)-2-(1-oxo-1-((2,3,3-trimethylbutan-2-yl)oxy)propan-2-yl)benzoate (400 MHz, CDCl₃)**

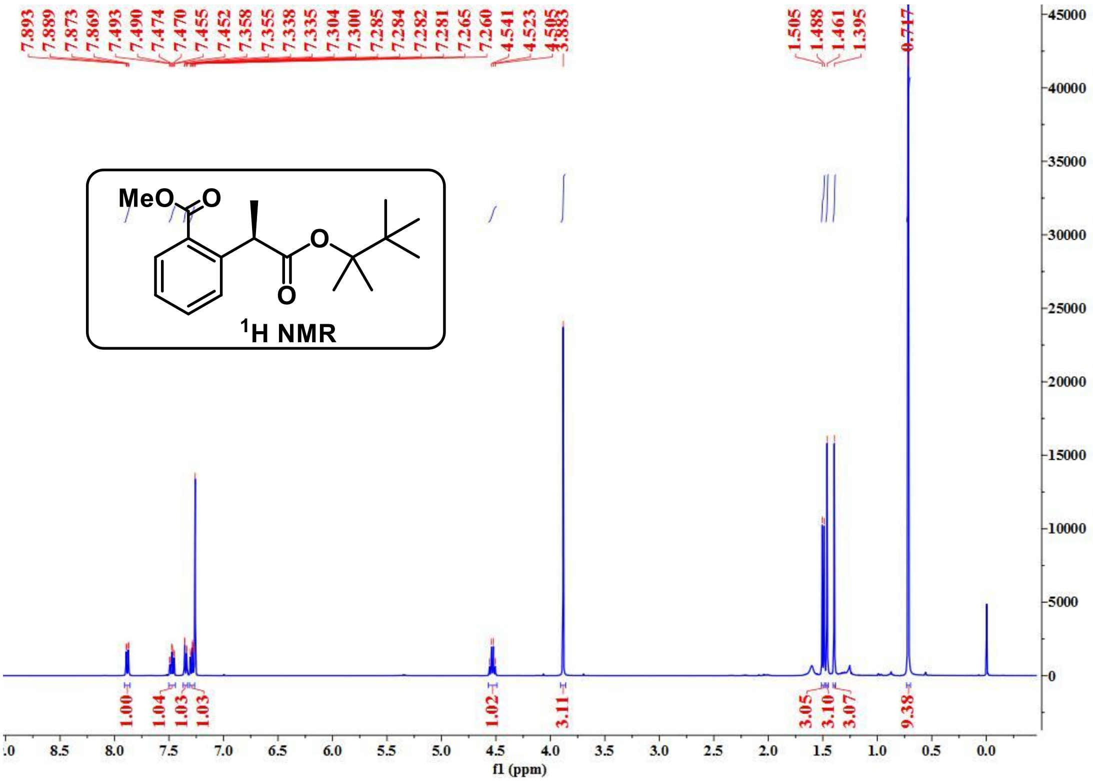

Supplementary Figure 36. ^{13}**C NMR Spectrum of Methyl (*R*)-2-(1-oxo-1-((2,3,3-trimethylbutan-2-yl)oxy)propan-2-yl)benzoate (101 MHz, CDCl₃)**

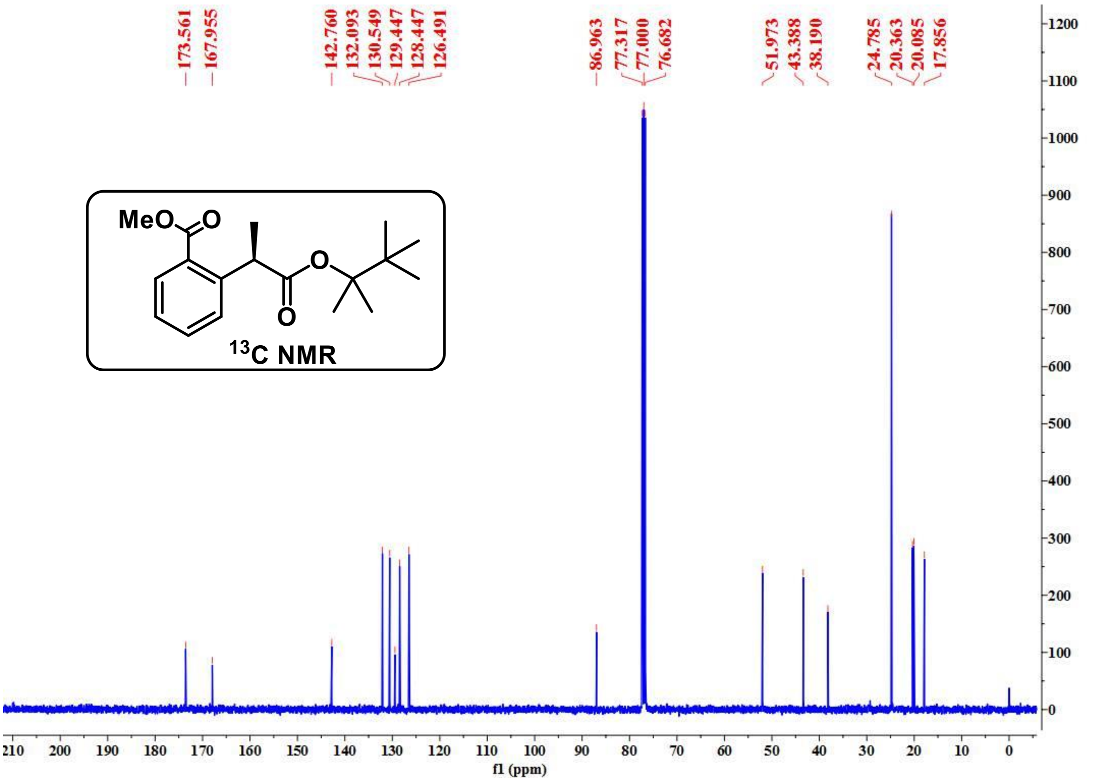

Supplementary Figure 37. ^{1}**H NMR Spectrum of Tert-butyl (*R*)-4-(1-oxo-1-((2,3,3-trimethylbutan-2-yl)oxy)propan-2-yl)benzoate (400 MHz, CDCl₃)**

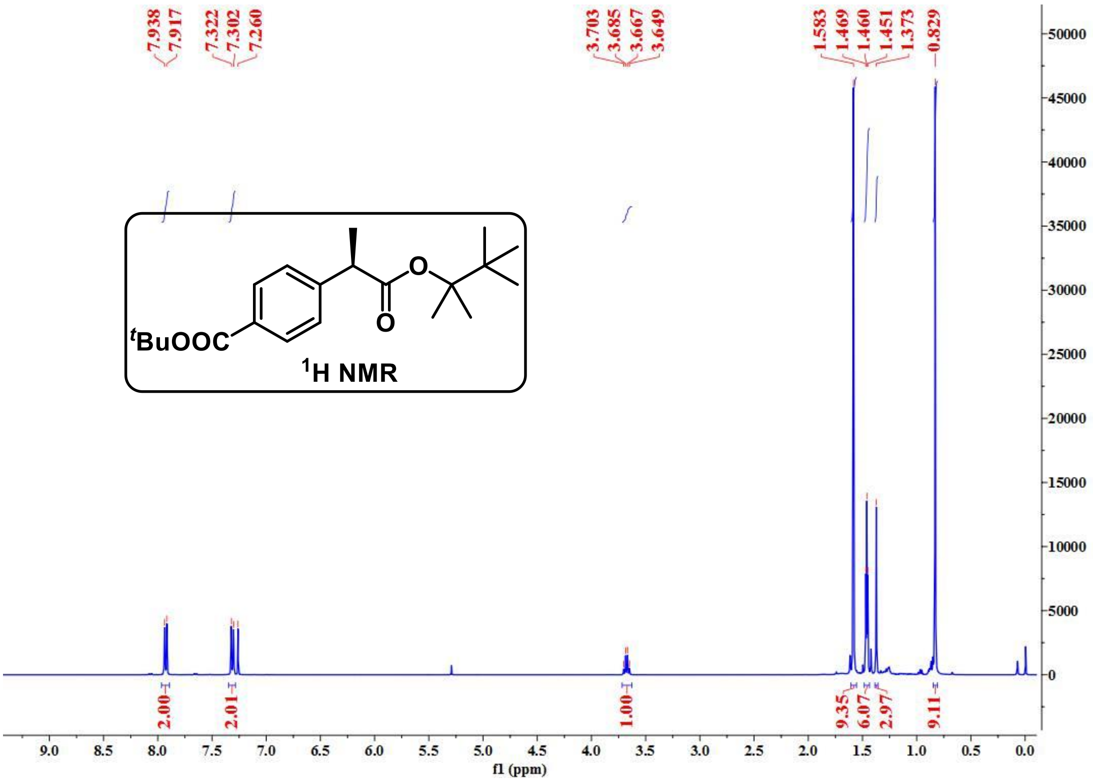

Supplementary Figure 38. ^{13}**C NMR Spectrum of Tert-butyl (*R*)-4-(1-oxo-1-((2,3,3-trimethylbutan-2-yl)oxy)propan-2-yl)benzoate (101 MHz, CDCl₃)**

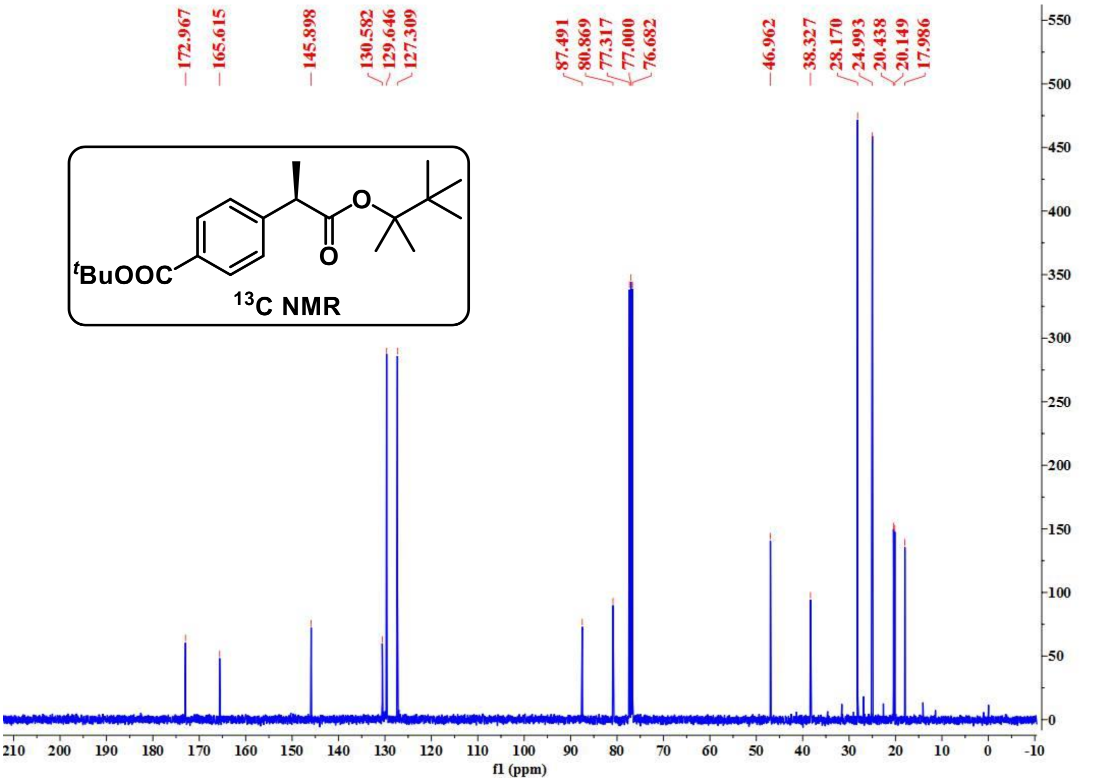

Supplementary Figure 39. ^{1}H NMR Spectrum of 2,3,3-trimethylbutan-2-yl (*R*)-2-(4-benzoylphenyl)propanoate (400 MHz, CDCl$_3$)

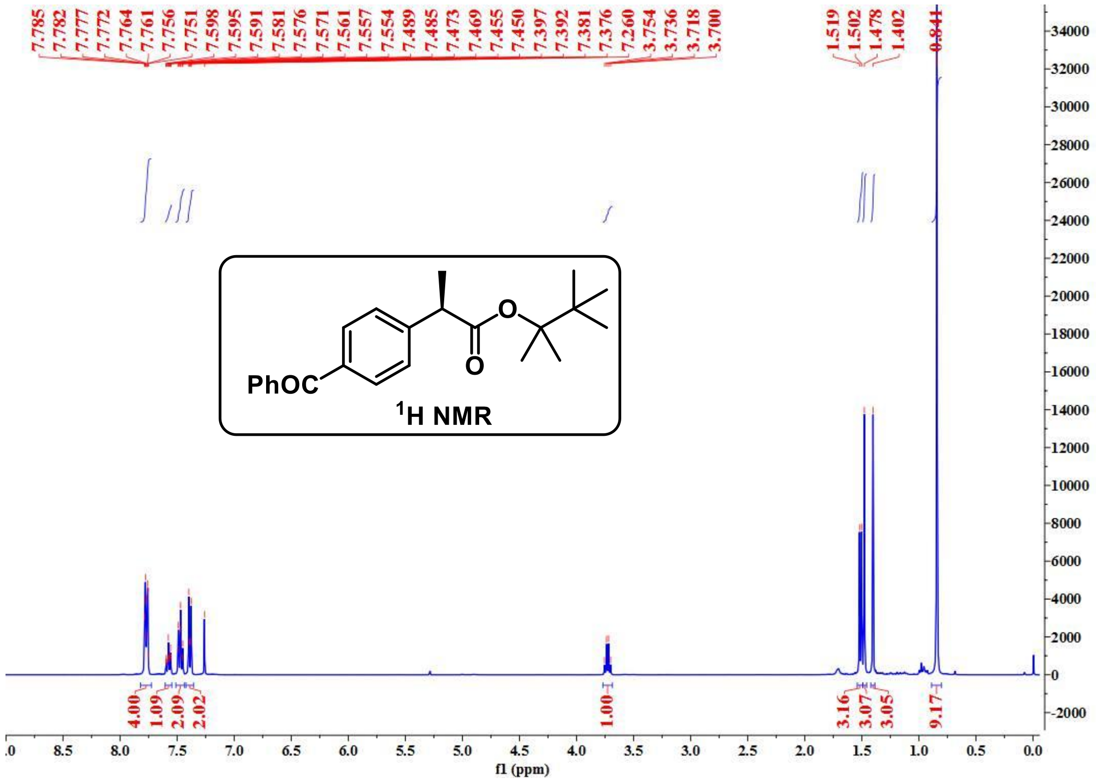

Supplementary Figure 40. ^{13}C NMR Spectrum of 2,3,3-trimethylbutan-2-yl (*R*)-2-(4-benzoylphenyl)propanoate (101 MHz, CDCl$_3$)

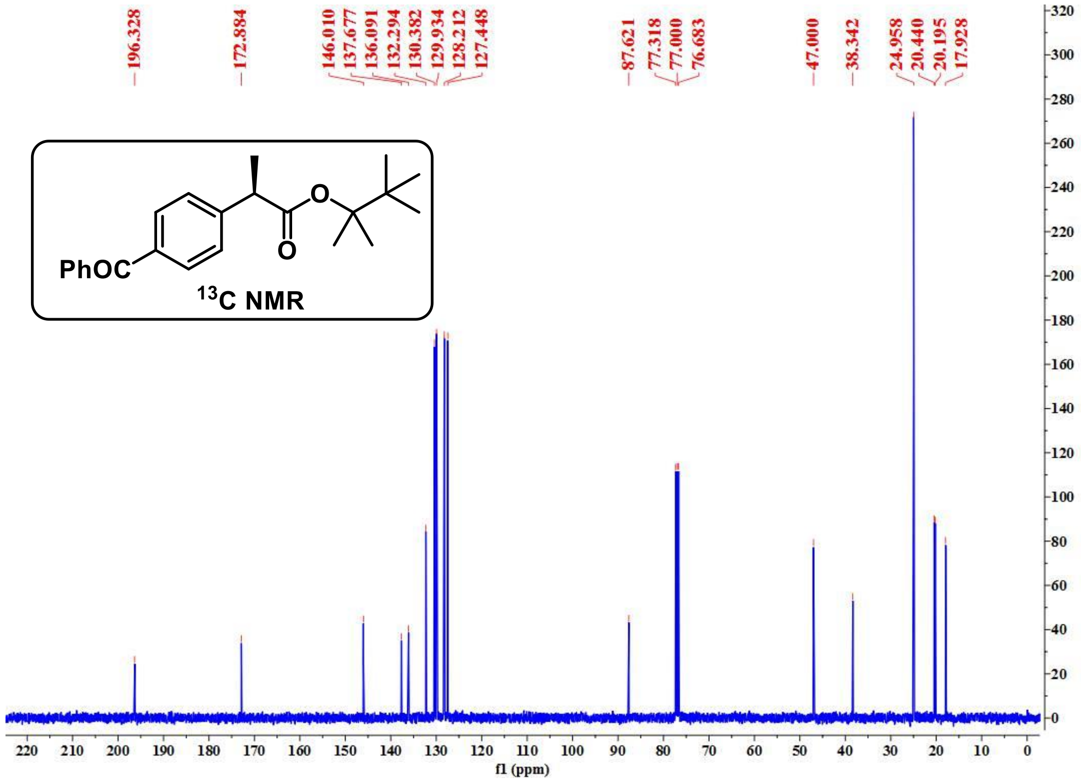

Supplementary Figure 41. ^{1}H NMR Spectrum of 2,3,3-trimethylbutan-2-yl (*R*)-2-(4-acetylphenyl)propanoate (400 MHz, $CDCl_3$)

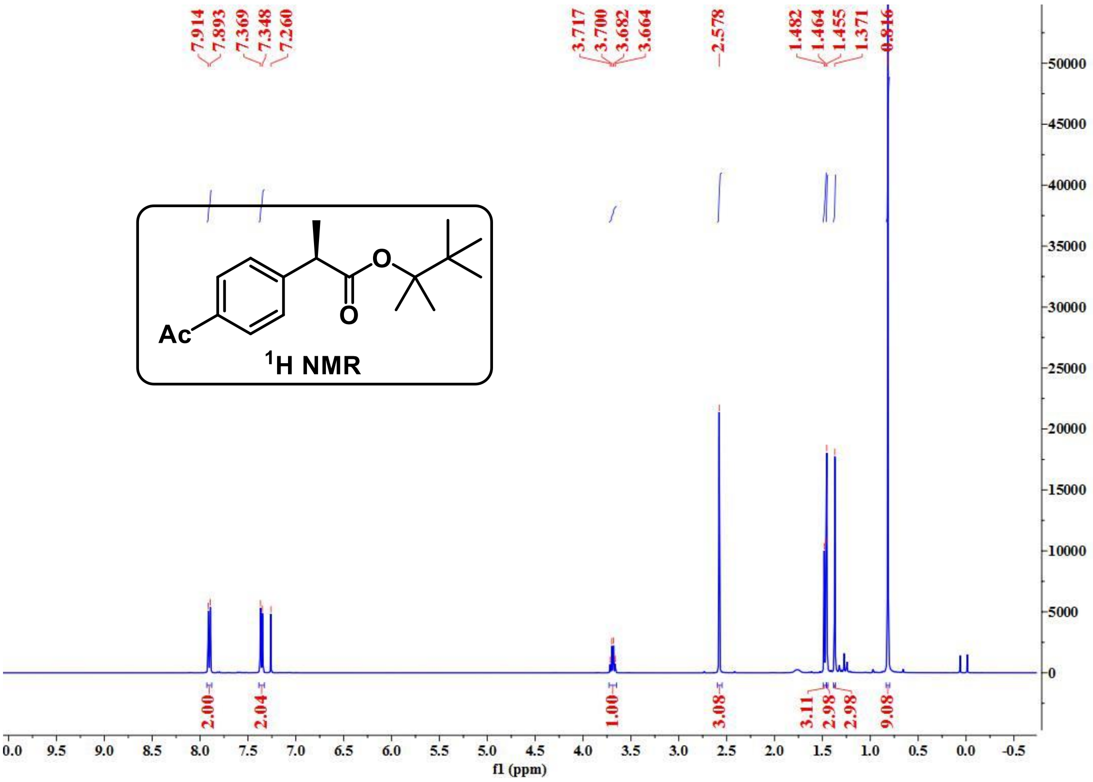

Supplementary Figure 42. ^{13}C NMR Spectrum of 2,3,3-trimethylbutan-2-yl (*R*)-2-(4-acetylphenyl)propanoate (101 MHz, $CDCl_3$)

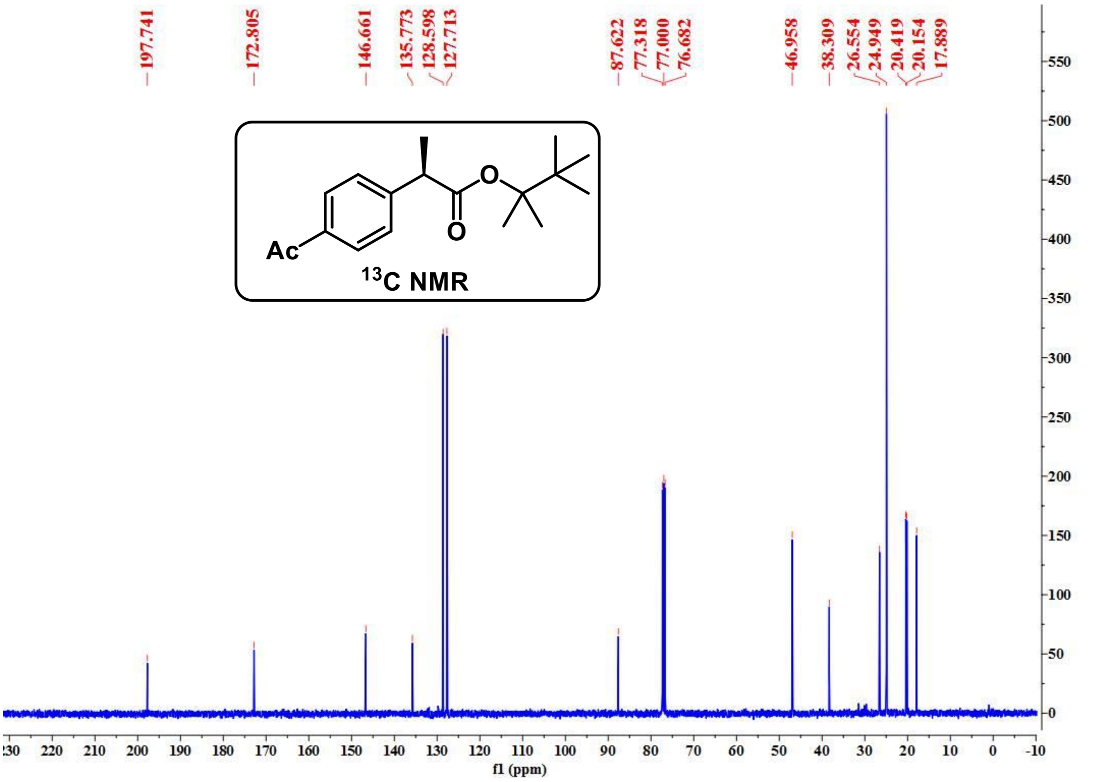

Supplementary Figure 43. ^{1}H NMR Spectrum of 2,3,3-trimethylbutan-2-yl (*R*)-2-(4-(dimethylcarbamoyl)phenyl)propanoate (400 MHz, $CDCl_3$)

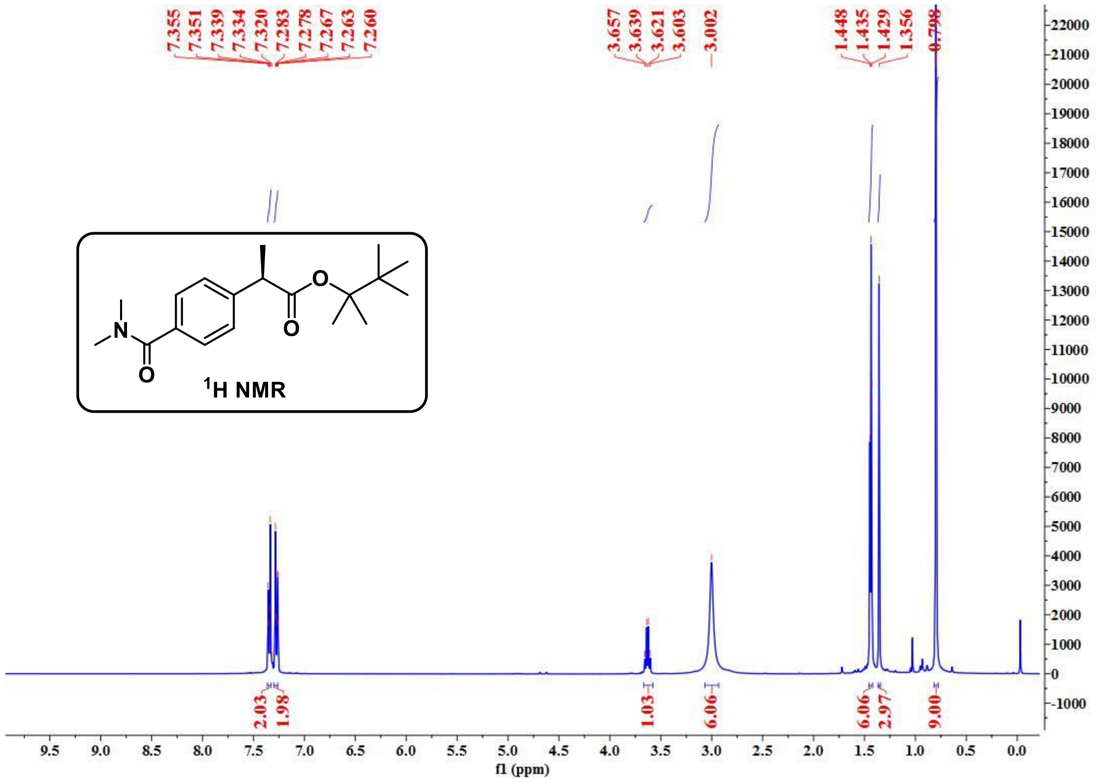

Supplementary Figure 44. ^{13}C NMR Spectrum of 2,3,3-trimethylbutan-2-yl (*R*)-2-(4-(dimethylcarbamoyl)phenyl)propanoate (101 MHz, $CDCl_3$)

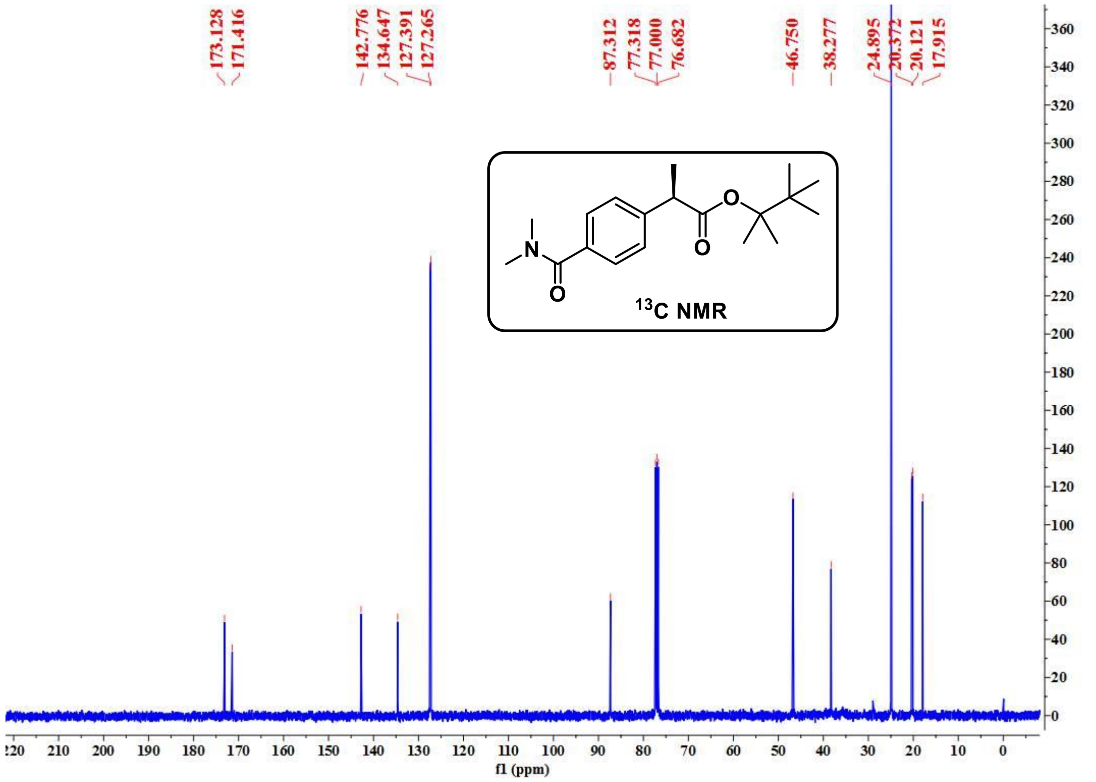

Supplementary Figure 45. ^{1}**H NMR Spectrum of 2-(methylthio)ethyl (*R*)-4-(1-oxo-1-((2,3,3-trimethylbutan-2-yl)oxy)propan-2-yl)benzoate (400 MHz, CDCl$_3$)**

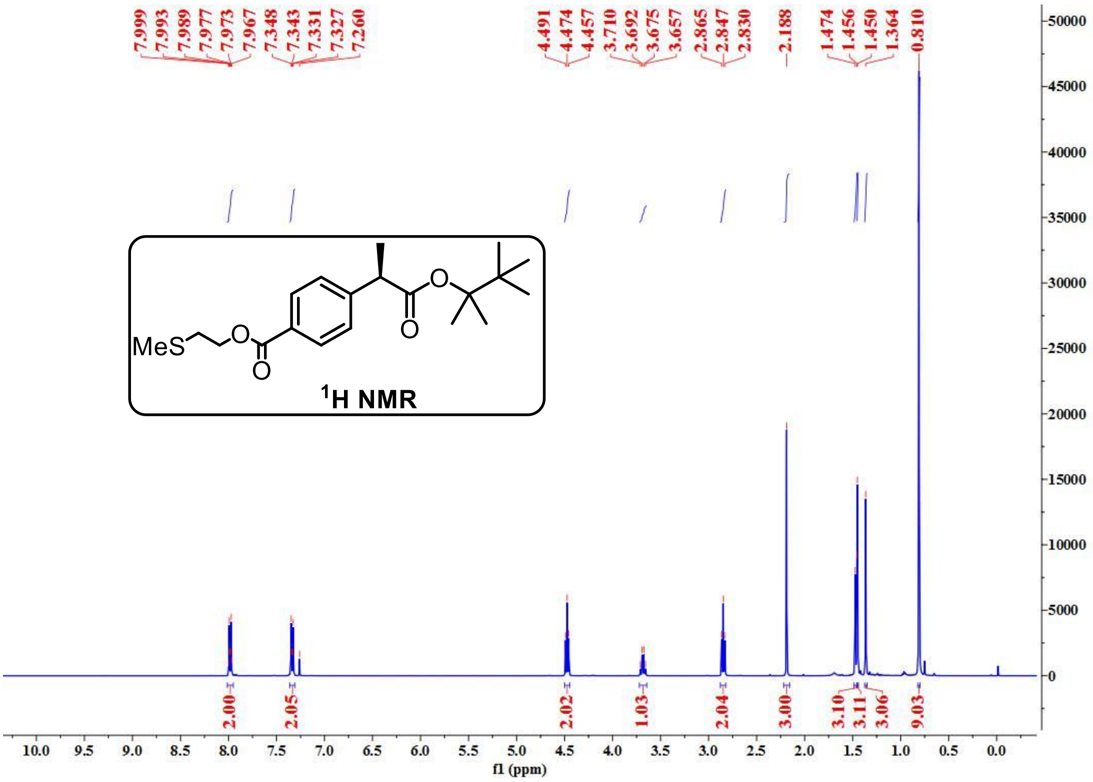

Supplementary Figure 46. ^{13}**C NMR Spectrum of 2-(methylthio)ethyl (*R*)-4-(1-oxo-1-((2,3,3-trimethylbutan-2-yl)oxy)propan-2-yl)benzoate (101 MHz, CDCl$_3$)**

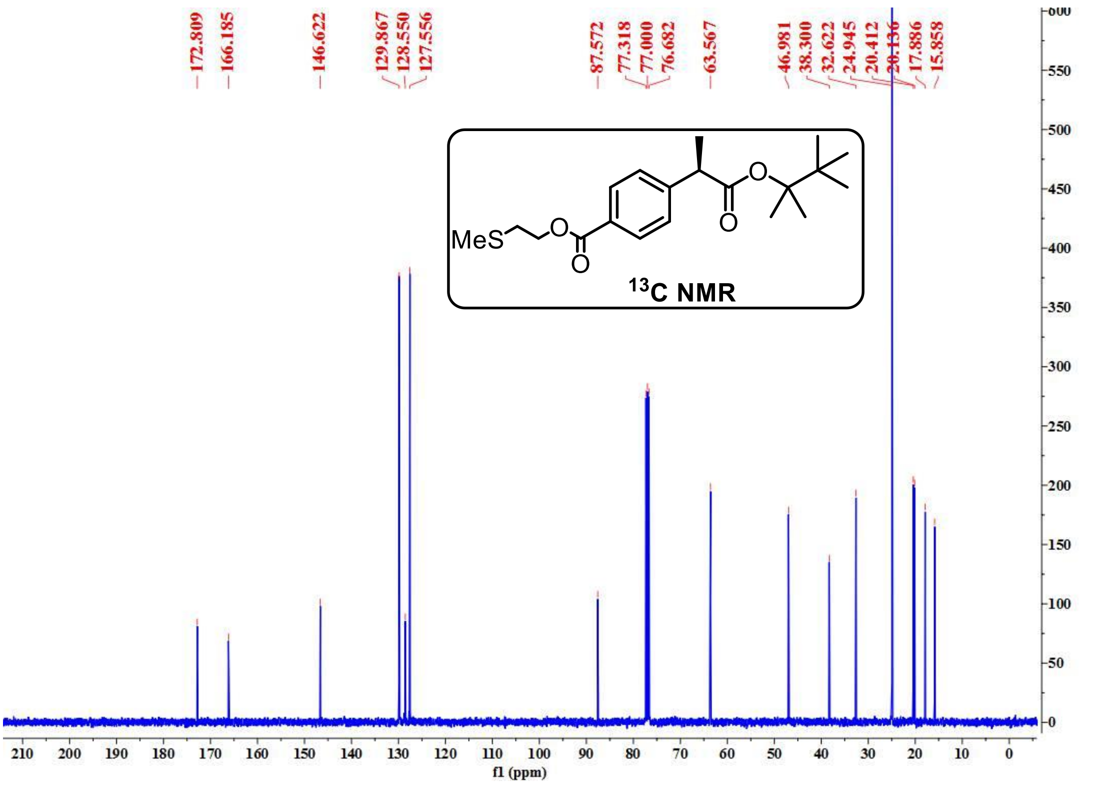

Supplementary Figure 47. ^{1}H NMR Spectrum of 2,3,3-trimethylbutan-2-yl (*R*)-2-(3-methoxy-4-methylphenyl)propanoate (400 MHz, $CDCl_3$)

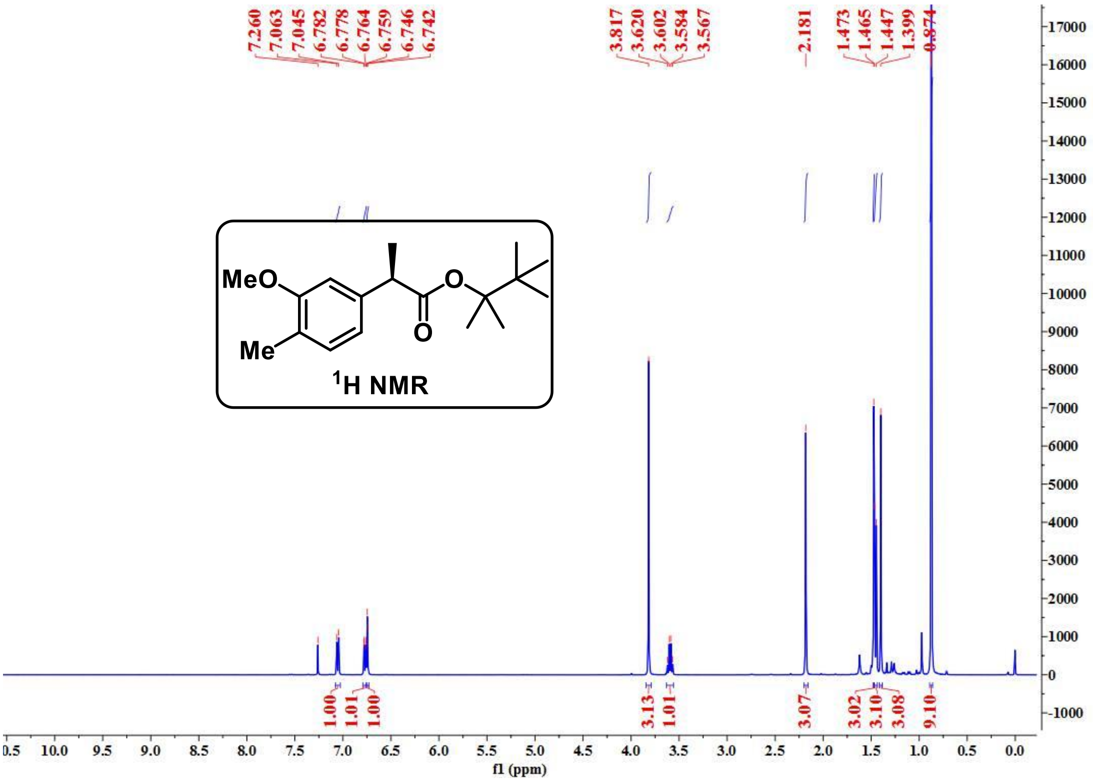

Supplementary Figure 48. ^{13}C NMR Spectrum of 2,3,3-trimethylbutan-2-yl (*R*)-2-(3-methoxy-4-methylphenyl)propanoate (101 MHz, $CDCl_3$)

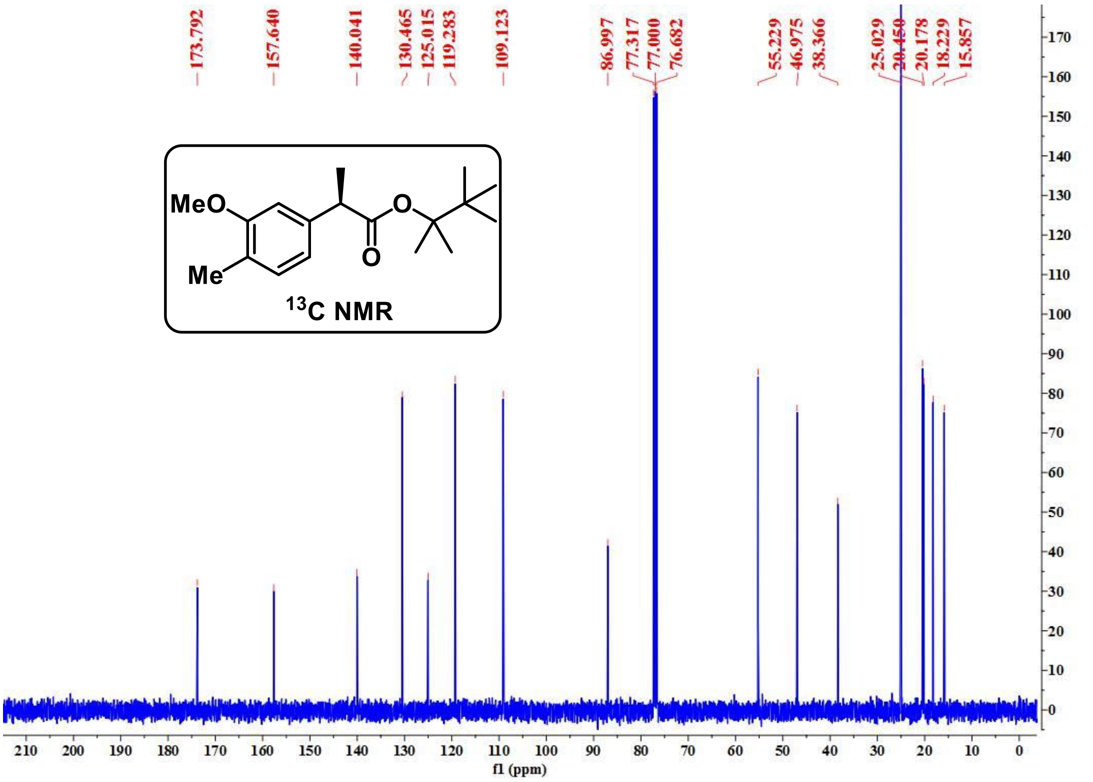

Supplementary Figure 49. ^{1}H NMR Spectrum of 2,3,3-trimethylbutan-2-yl (*R*)-2-(4-(tert-butyl)phenyl)propanoate (400 MHz, CDCl$_3$)

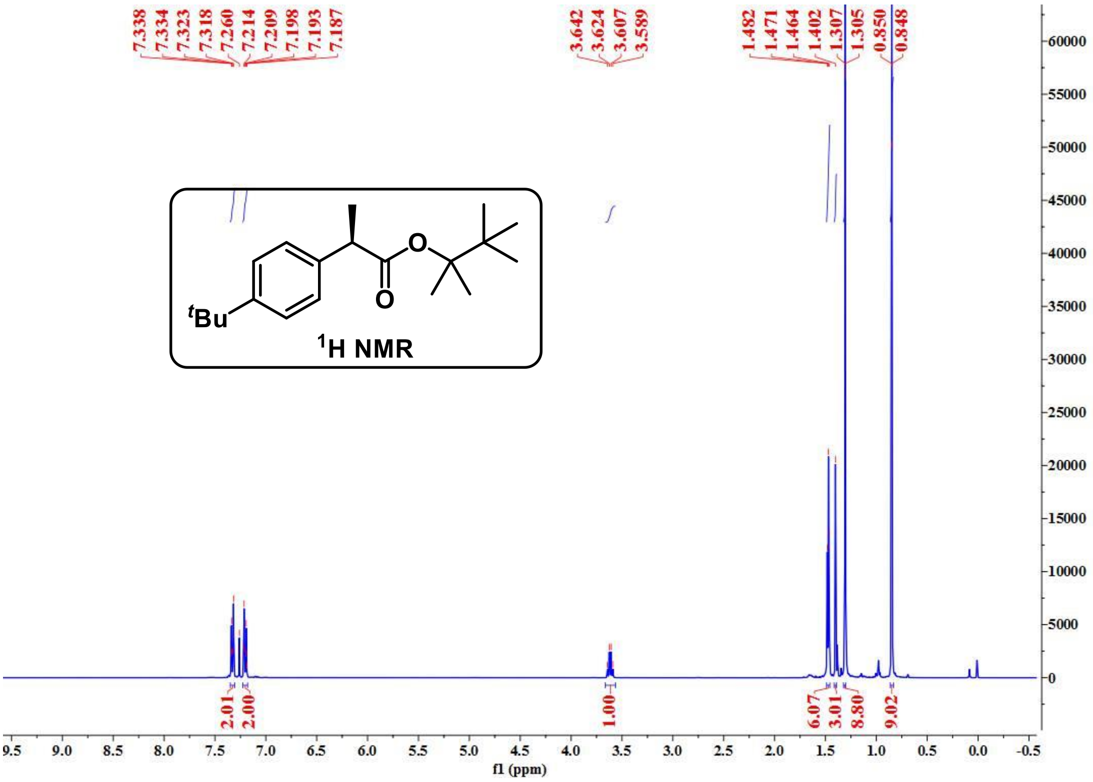

Supplementary Figure 50. ^{13}C NMR Spectrum of 2,3,3-trimethylbutan-2-yl (*R*)-2-(4-(tert-butyl)phenyl)propanoate (101 MHz, CDCl$_3$)

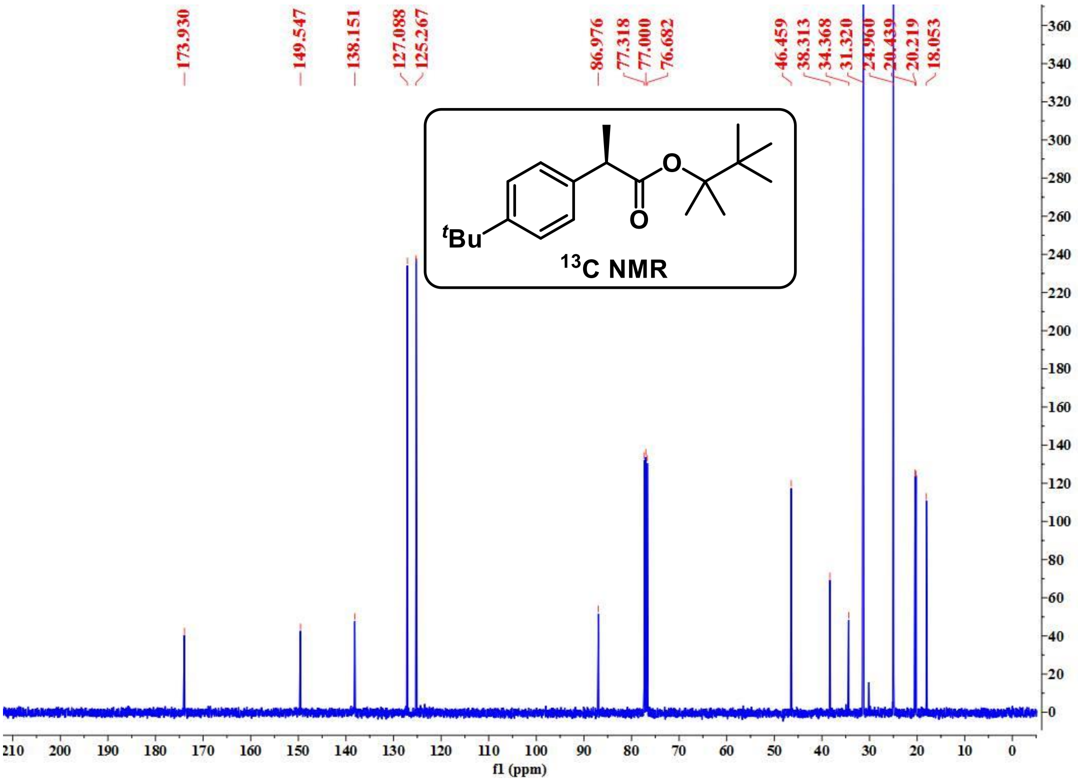

Supplementary Figure 51. **^{1}H NMR Spectrum of 2,3,3-trimethylbutan-2-yl (*R*)-2-([1,1'-biphenyl]-4-yl)propanoate (400 MHz, $CDCl_3$)**

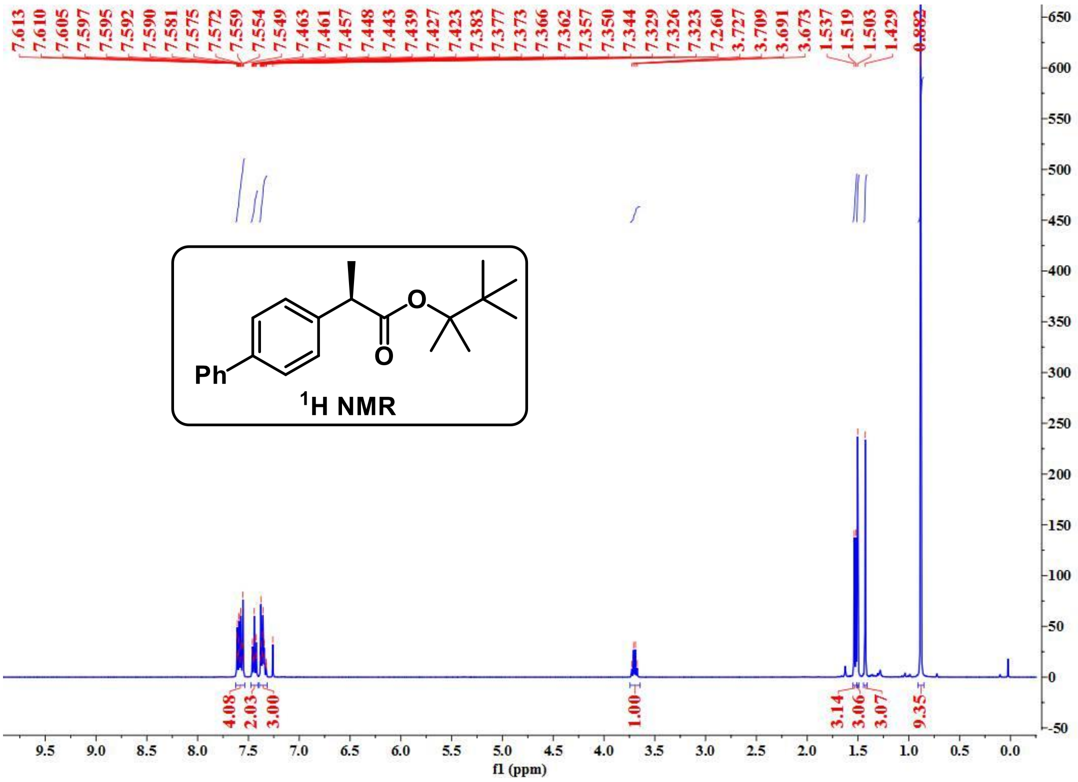

Supplementary Figure 52. **^{13}C NMR Spectrum of 2,3,3-trimethylbutan-2-yl (*R*)-2-([1,1'-biphenyl]-4-yl)propanoate (101 MHz, $CDCl_3$)**

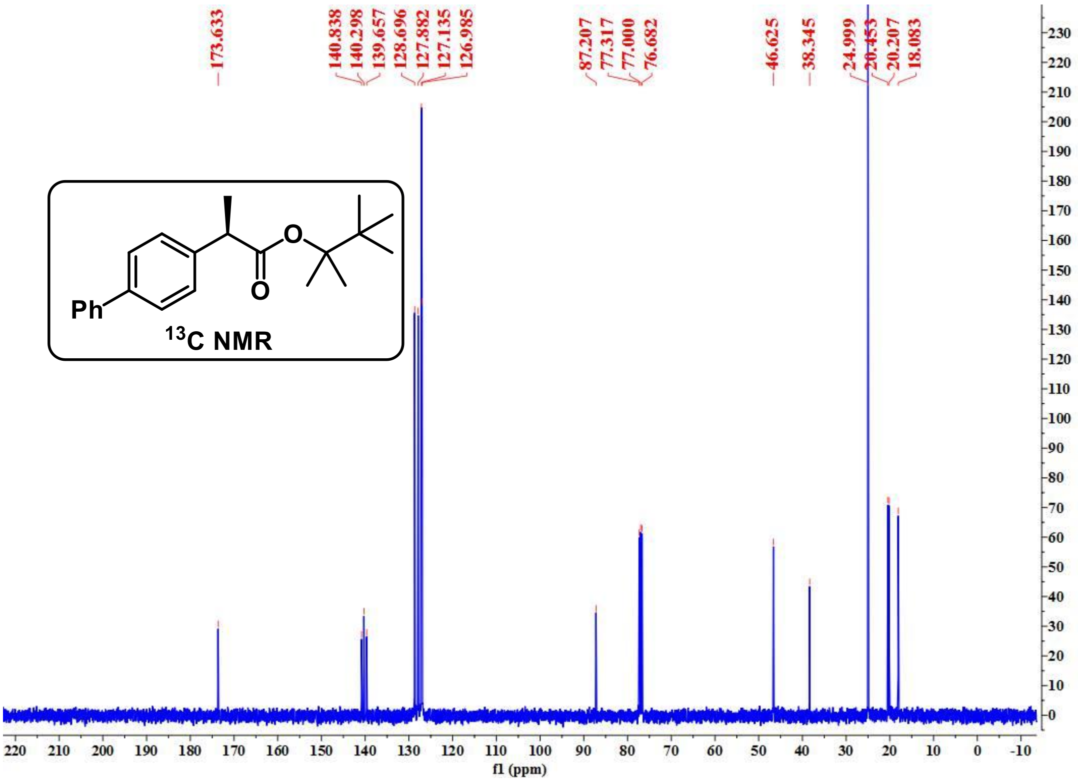

Supplementary Figure 53. ^{1}H NMR Spectrum of 2,3,3-trimethylbutan-2-yl (*R*)-2-(4-(methylthio)phenyl)propanoate (400 MHz, $CDCl_3$)

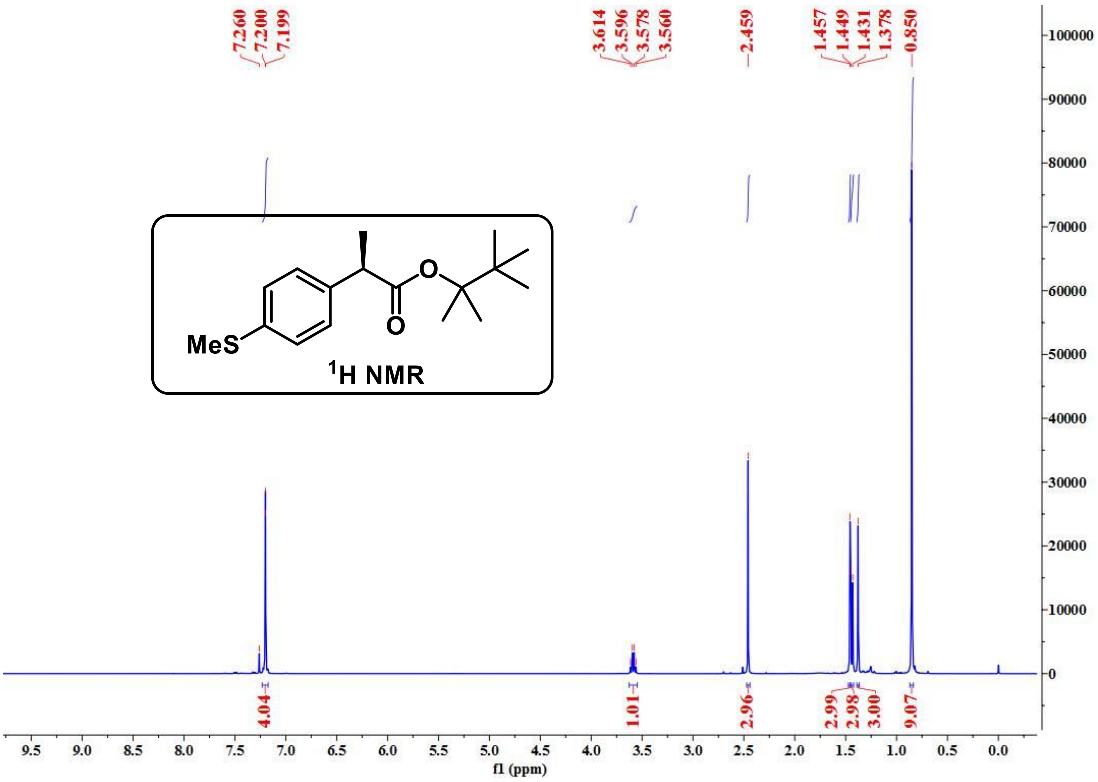

Supplementary Figure 54. ^{13}C NMR Spectrum of 2,3,3-trimethylbutan-2-yl (*R*)-2-(4-(methylthio)phenyl)propanoate (101 MHz, $CDCl_3$)

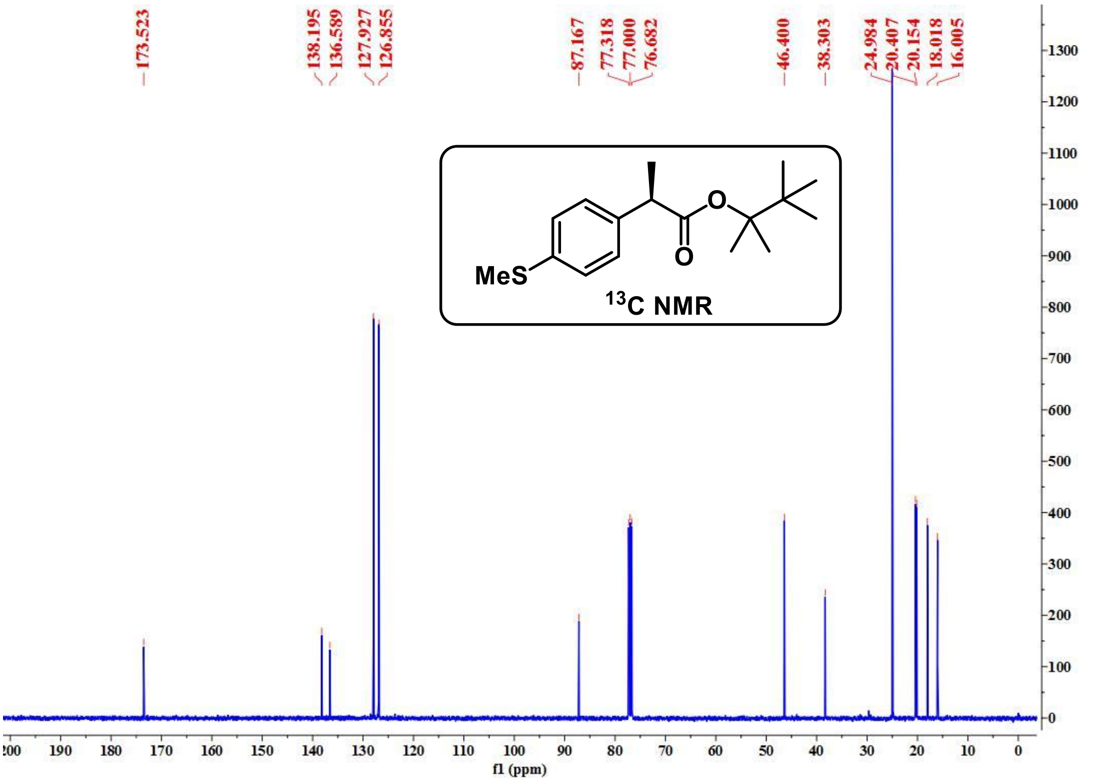

Supplementary Figure 55. ^{1}H NMR Spectrum of 2,3,3-trimethylbutan-2-yl (*R*)-2-(4-((trifluoromethyl)thio)phenyl)propanoate (400 MHz, $CDCl_3$)

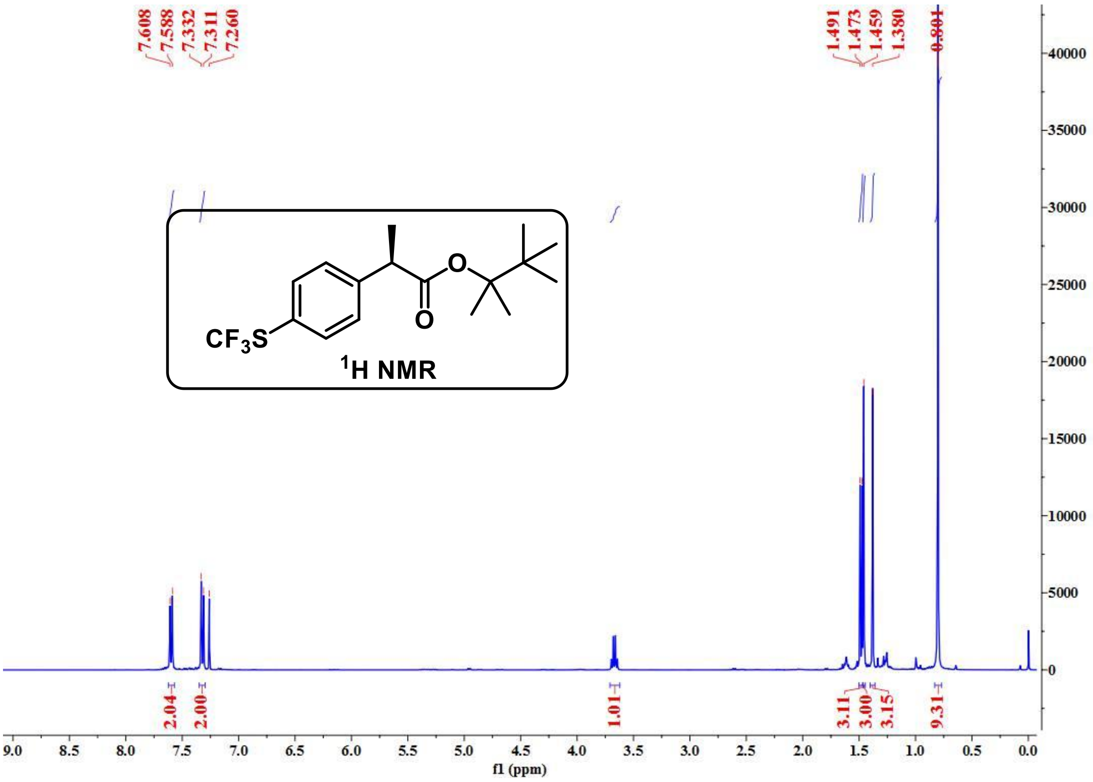

Supplementary Figure 56. ^{19}F NMR Spectrum of 2,3,3-trimethylbutan-2-yl (*R*)-2-(4-((trifluoromethyl)thio)phenyl)propanoate (375 MHz, $CDCl_3$)

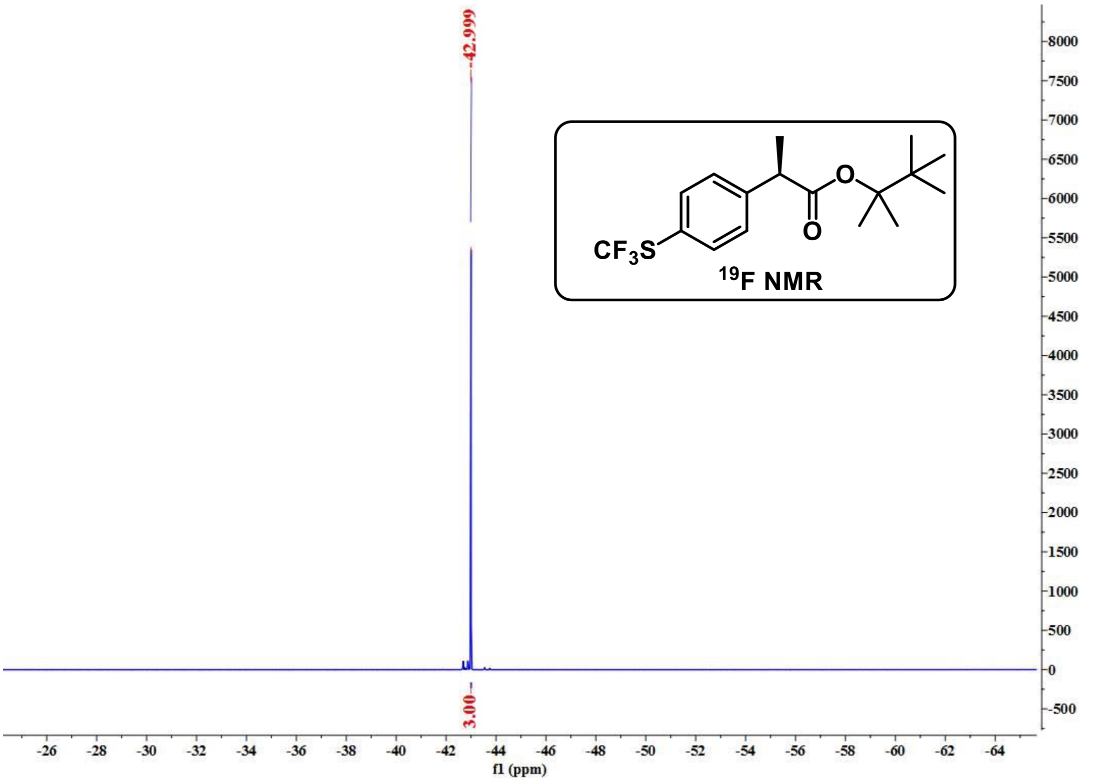

Supplementary Figure 57. ^{13}C NMR Spectrum of 2,3,3-trimethylbutan-2-yl (*R*)-2-(4-((trifluoromethyl)thio)phenyl)propanoate (101 MHz, $CDCl_3$)

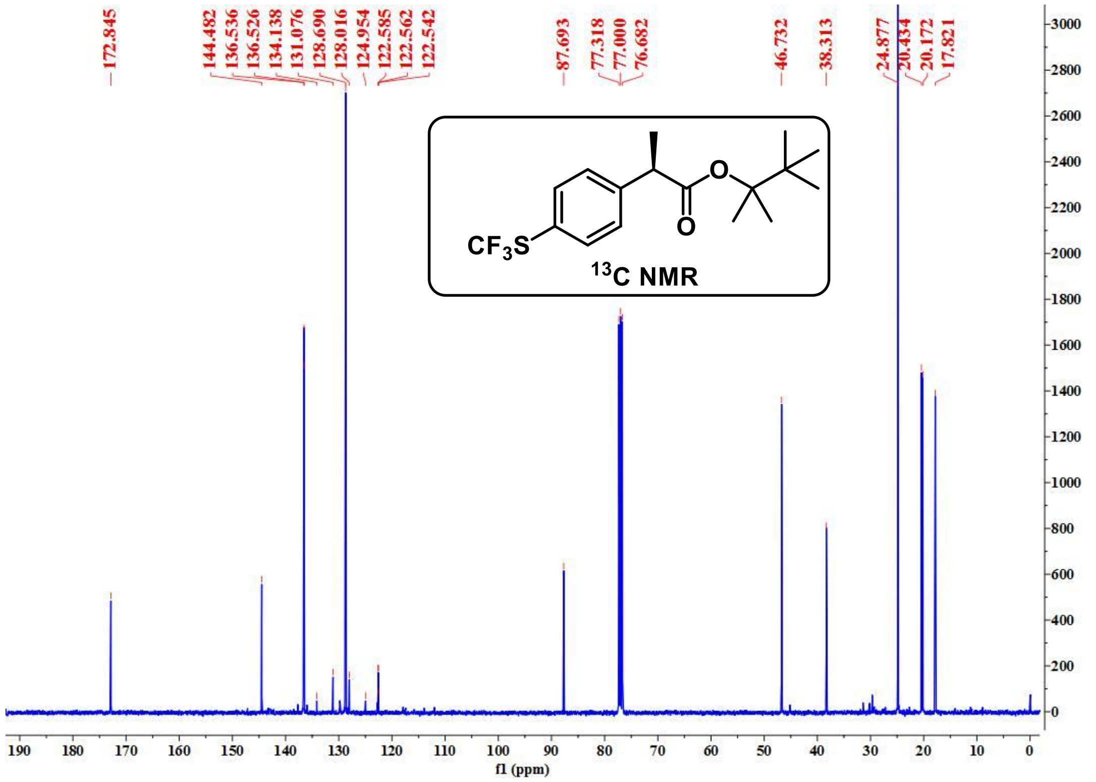

Supplementary Figure 58. ^{1}H NMR Spectrum of 2,3,3-trimethylbutan-2-yl (*R*)-2-(4-(phenylsulfonyl)phenyl)propanoate (400 MHz, $CDCl_3$)

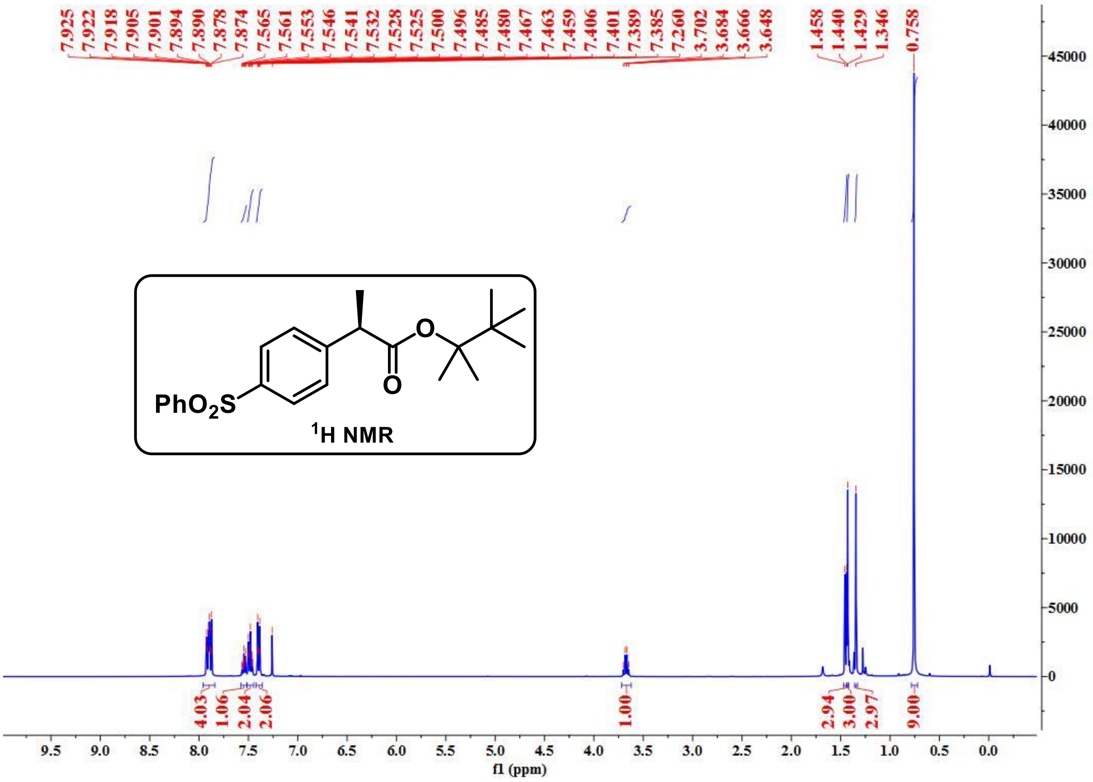

Supplementary Figure 59. ^{13}C NMR Spectrum of 2,3,3-trimethylbutan-2-yl (*R*)-2-(4-(phenylsulfonyl)phenyl)propanoate (101 MHz, $CDCl_3$)

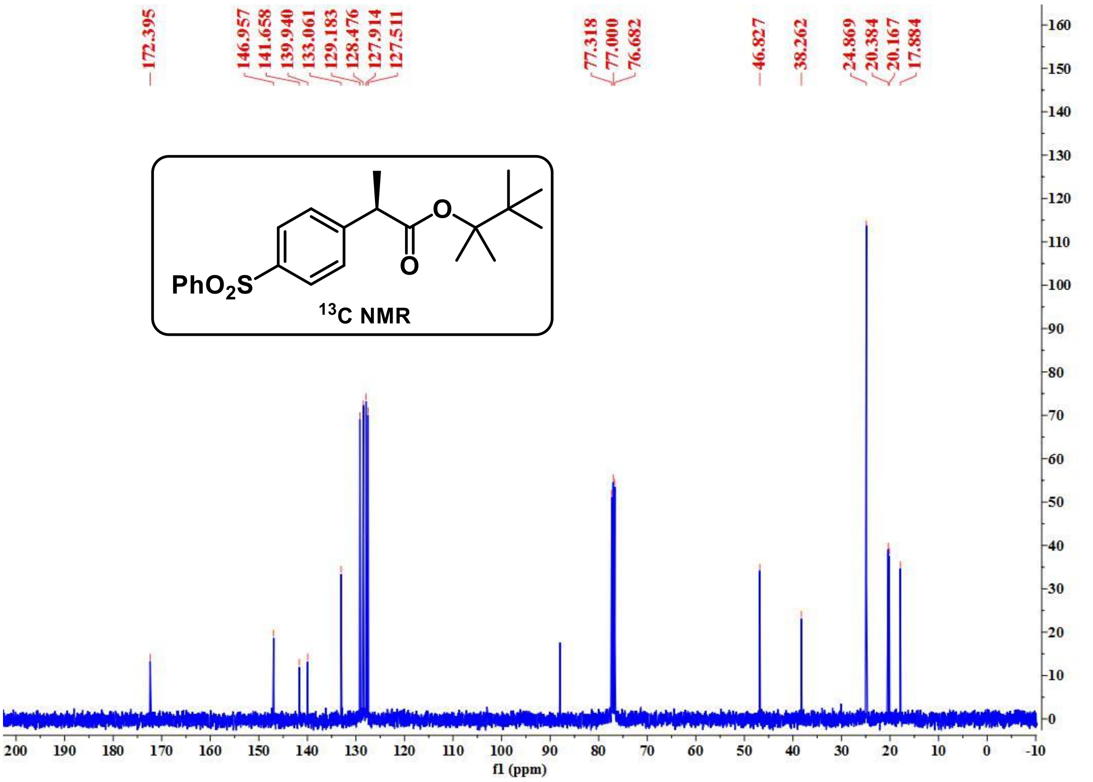

Supplementary Figure 60. ^{1}H NMR Spectrum of 2,3,3-trimethylbutan-2-yl (*R*)-2-(4-cyanophenyl)propanoate (400 MHz, $CDCl_3$)

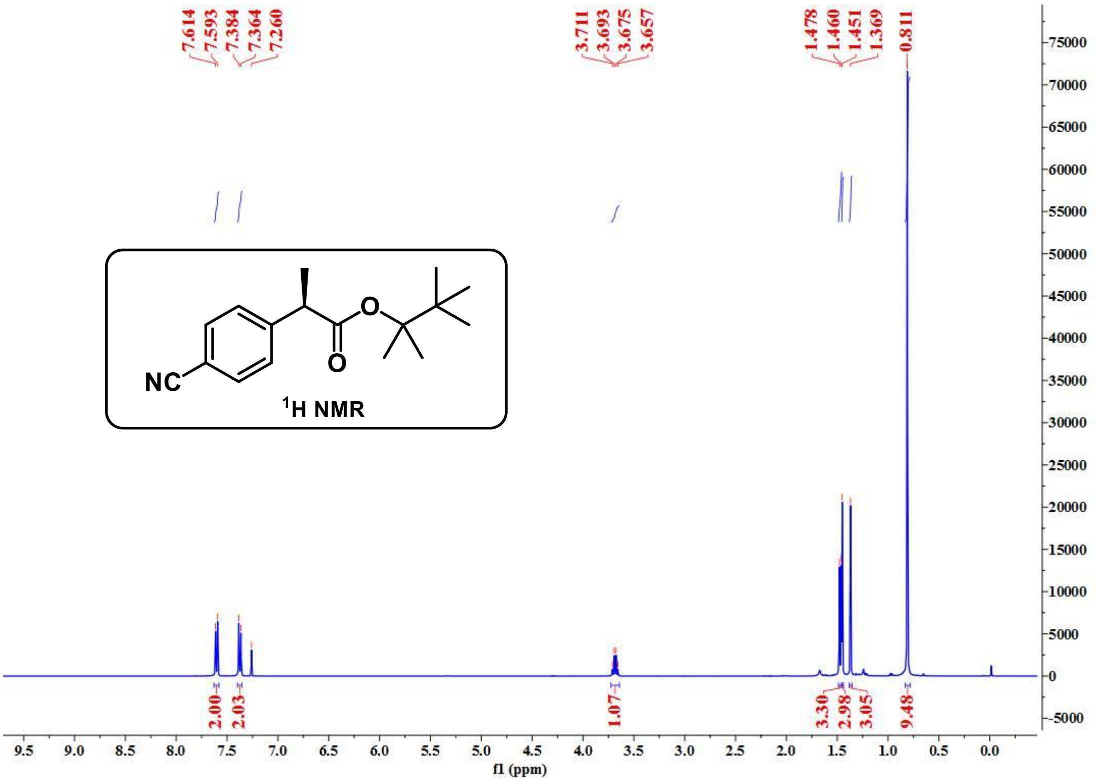

Supplementary Figure 61. ^{13}C NMR Spectrum of 2,3,3-trimethylbutan-2-yl (*R*)-2-(4-cyanophenyl)propanoate (101 MHz, $CDCl_3$)

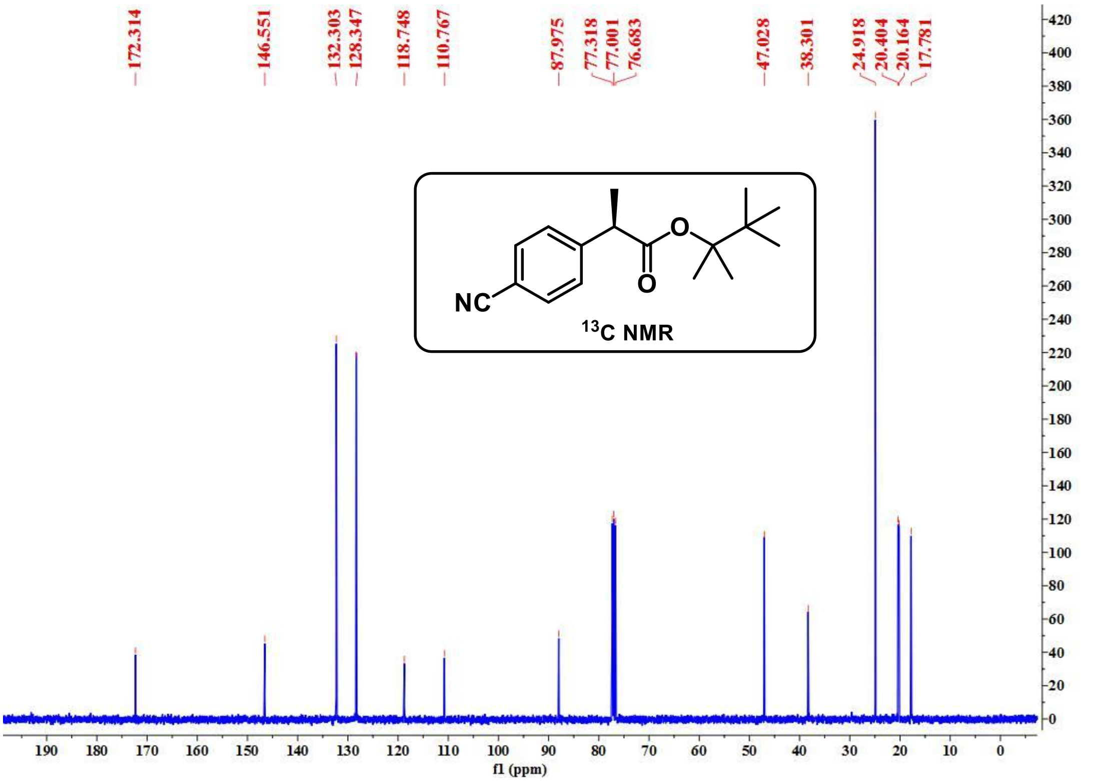

Supplementary Figure 62. ^{1}H NMR Spectrum of 2,3,3-trimethylbutan-2-yl (*R*)-2-(4-((tert-butoxycarbonyl)amino)phenyl)propanoate (400 MHz, $CDCl_3$)

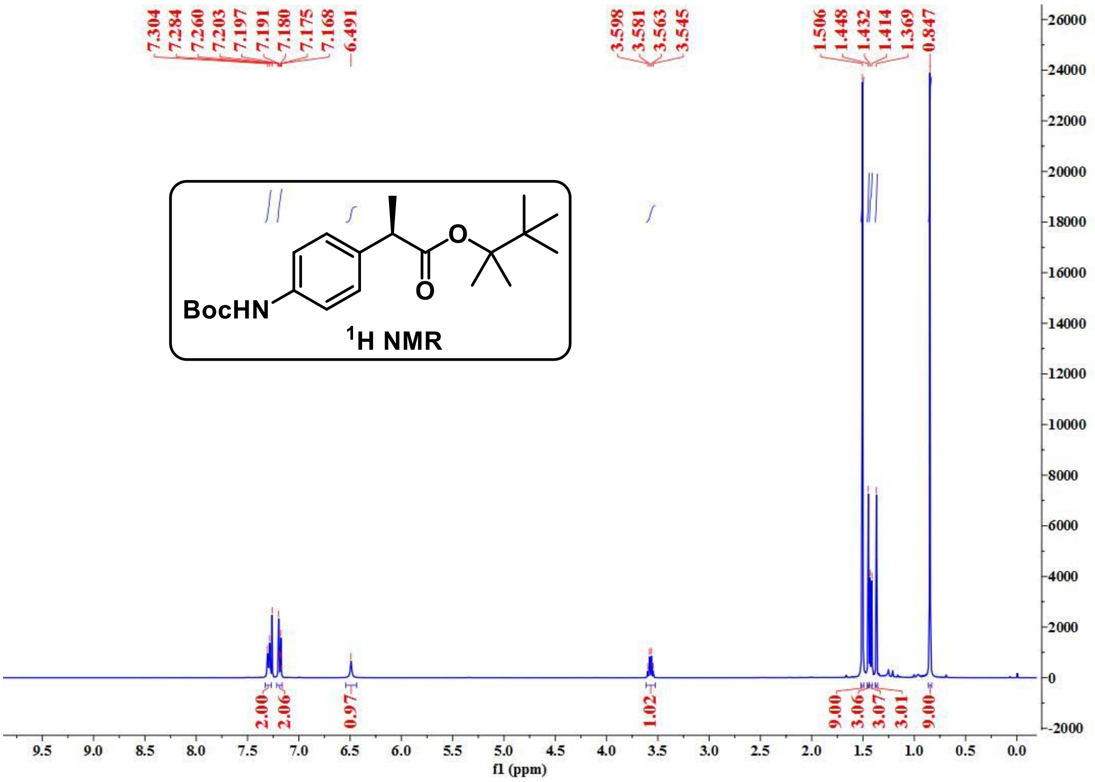

Supplementary Figure 63. ^{13}C NMR Spectrum of 2,3,3-trimethylbutan-2-yl (*R*)-2-(4-((tert-butoxycarbonyl)amino)phenyl)propanoate (101 MHz, $CDCl_3$)

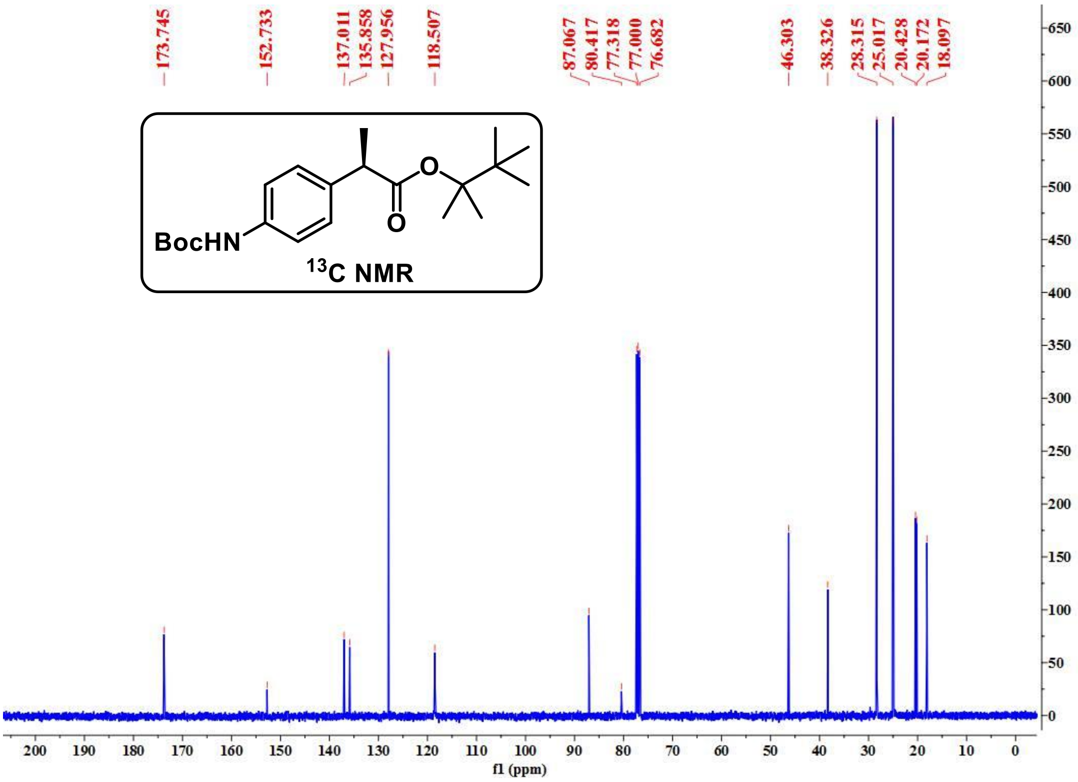

Supplementary Figure 64. ^{1}H NMR Spectrum of 2,3,3-trimethylbutan-2-yl (*R*)-2-(4-acetamidophenyl)propanoate (400 MHz, $CDCl_3$)

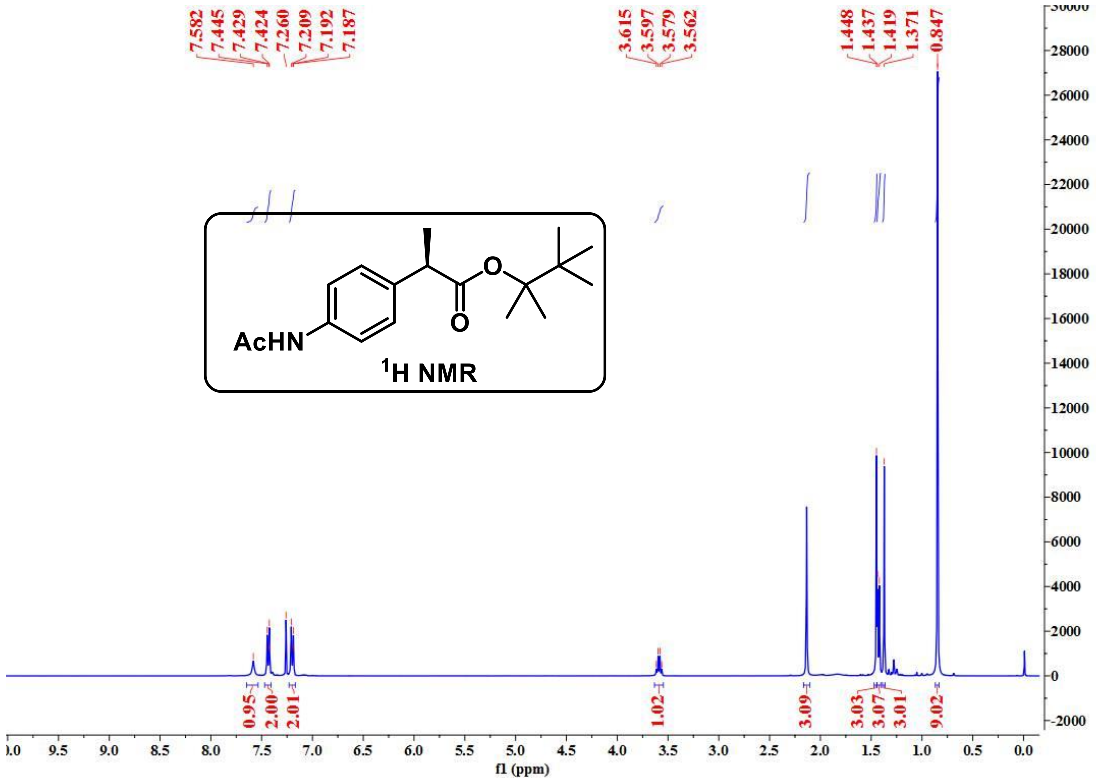

Supplementary Figure 65. ^{13}C NMR Spectrum of 2,3,3-trimethylbutan-2-yl (*R*)-2-(4-acetamidophenyl)propanoate (101 MHz, $CDCl_3$)

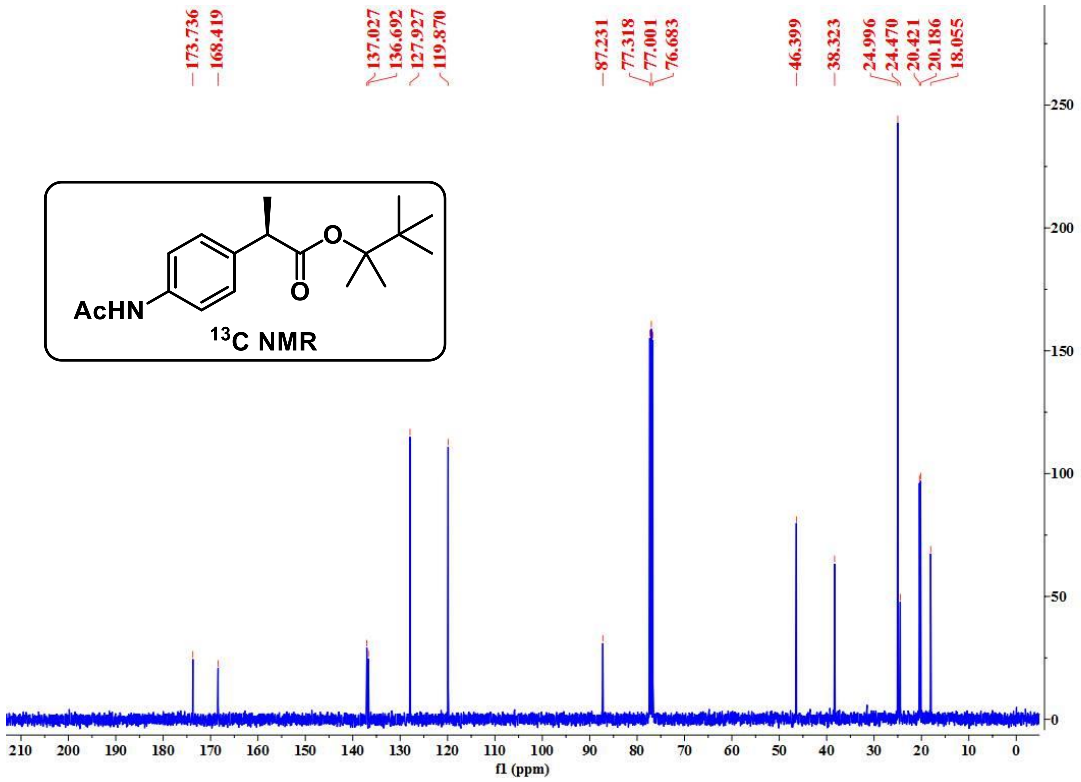

Supplementary Figure 66. ^{1}H NMR Spectrum of 2,3,3-trimethylbutan-2-yl (*R*)-2-(4-(2-oxopyrrolidin-1-yl)phenyl)propanoate (400 MHz, $CDCl_3$)

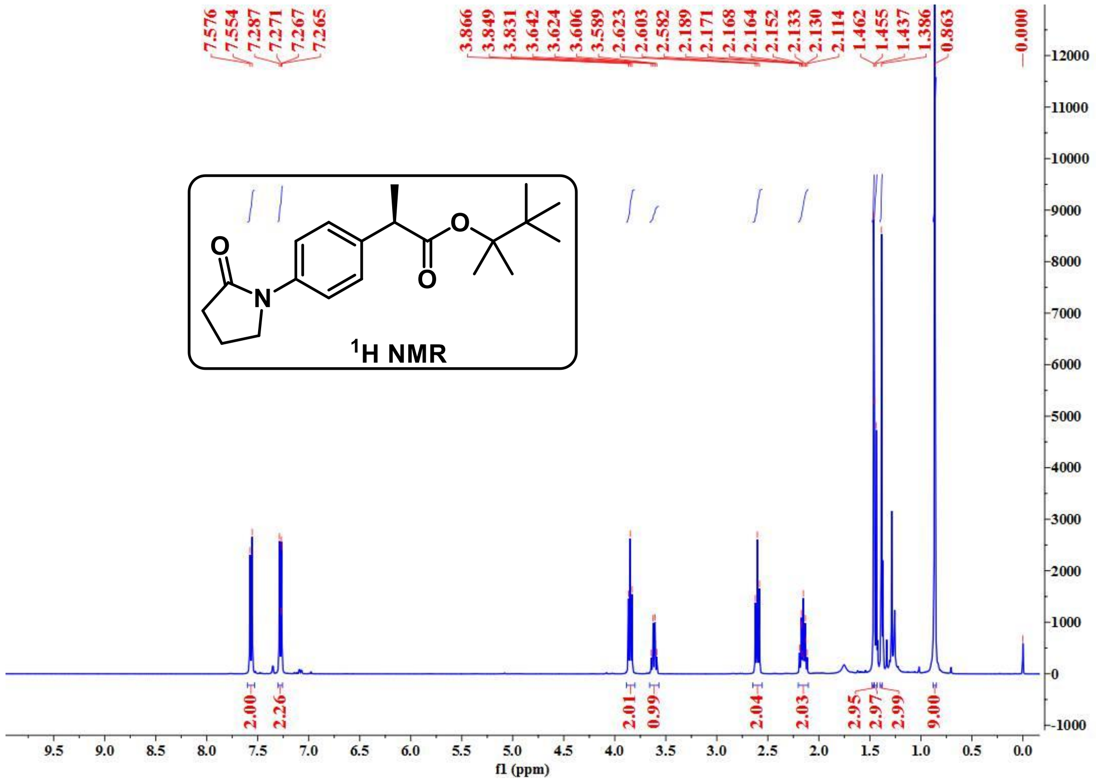

Supplementary Figure 67. ^{13}C NMR Spectrum of 2,3,3-trimethylbutan-2-yl (*R*)-2-(4-(2-oxopyrrolidin-1-yl)phenyl)propanoate (101 MHz, $CDCl_3$)

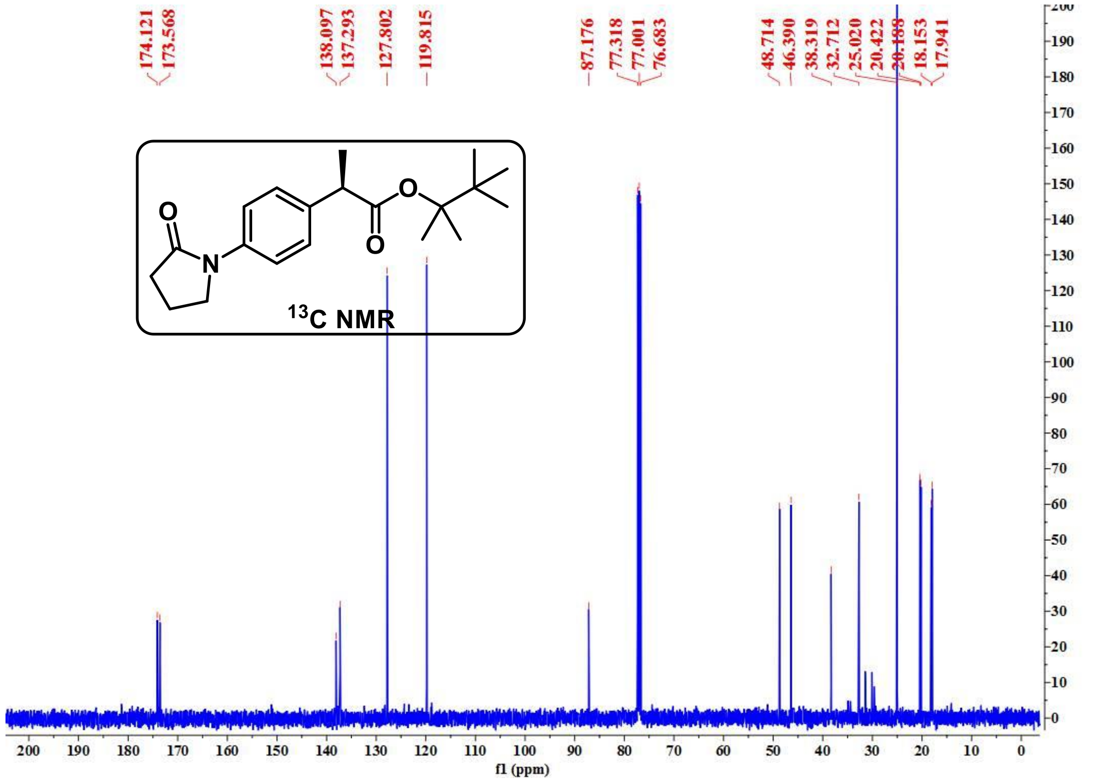

Supplementary Figure 68. ¹H NMR Spectrum of 2,3,3-trimethylbutan-2-yl (*R*)-2-(4-((trimethylsilyl)ethynyl)phenyl)propanoate (400 MHz, $CDCl_3$)

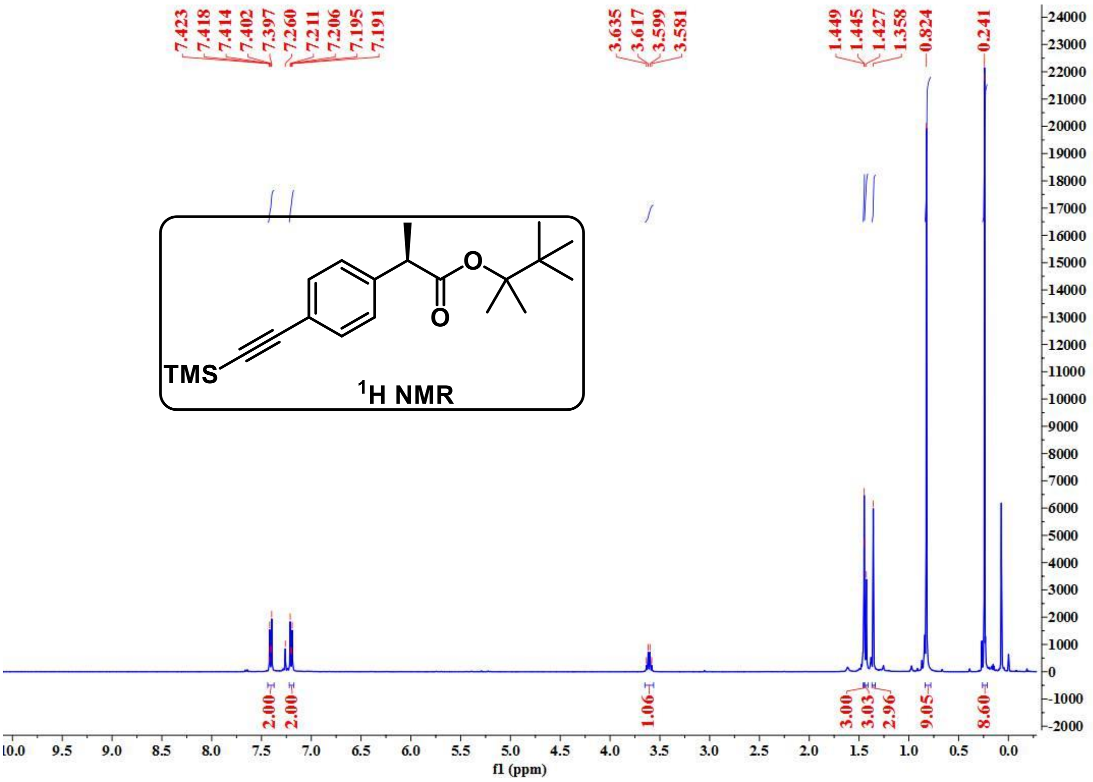

Supplementary Figure 69. ¹³C NMR Spectrum of 2,3,3-trimethylbutan-2-yl (*R*)-2-(4-((trimethylsilyl)ethynyl)phenyl)propanoate (101 MHz, $CDCl_3$)

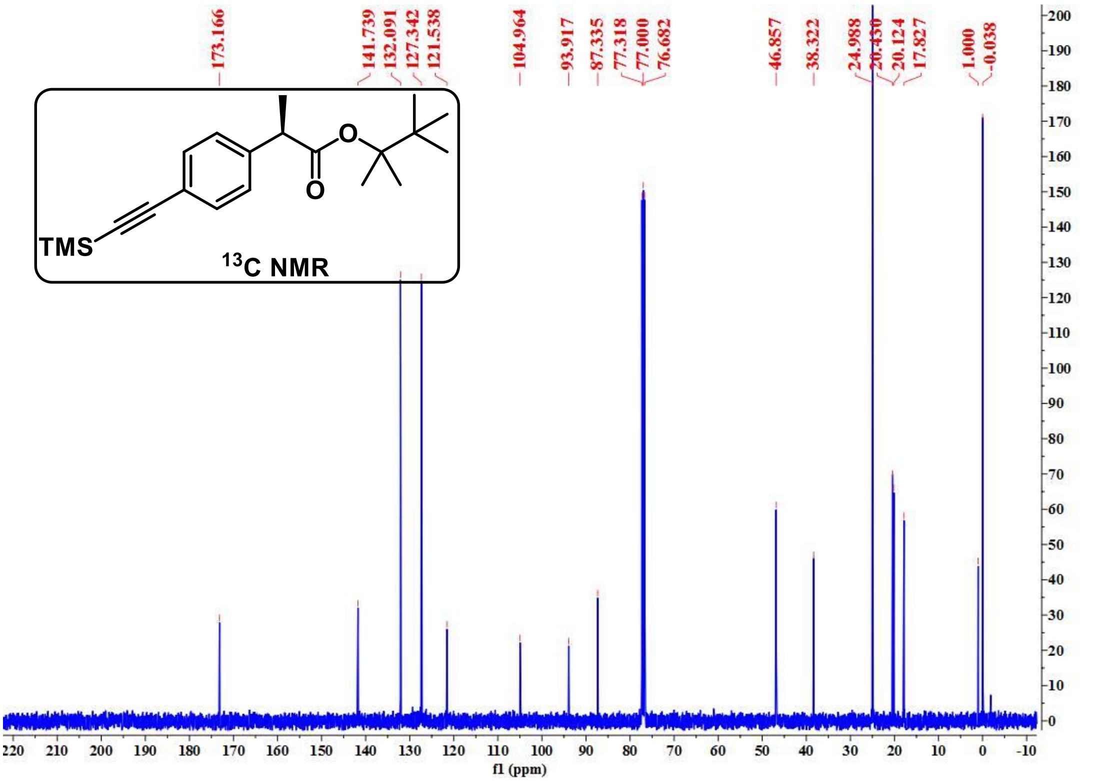

Supplementary Figure 70. ^{1}H NMR Spectrum of 2,3,3-trimethylbutan-2-yl (*R*)-2-(4-(4,4,5,5-tetramethyl-1,3,2-dioxaborolan-2-yl)phenyl)propanoate (400 MHz, CDCl$_3$)

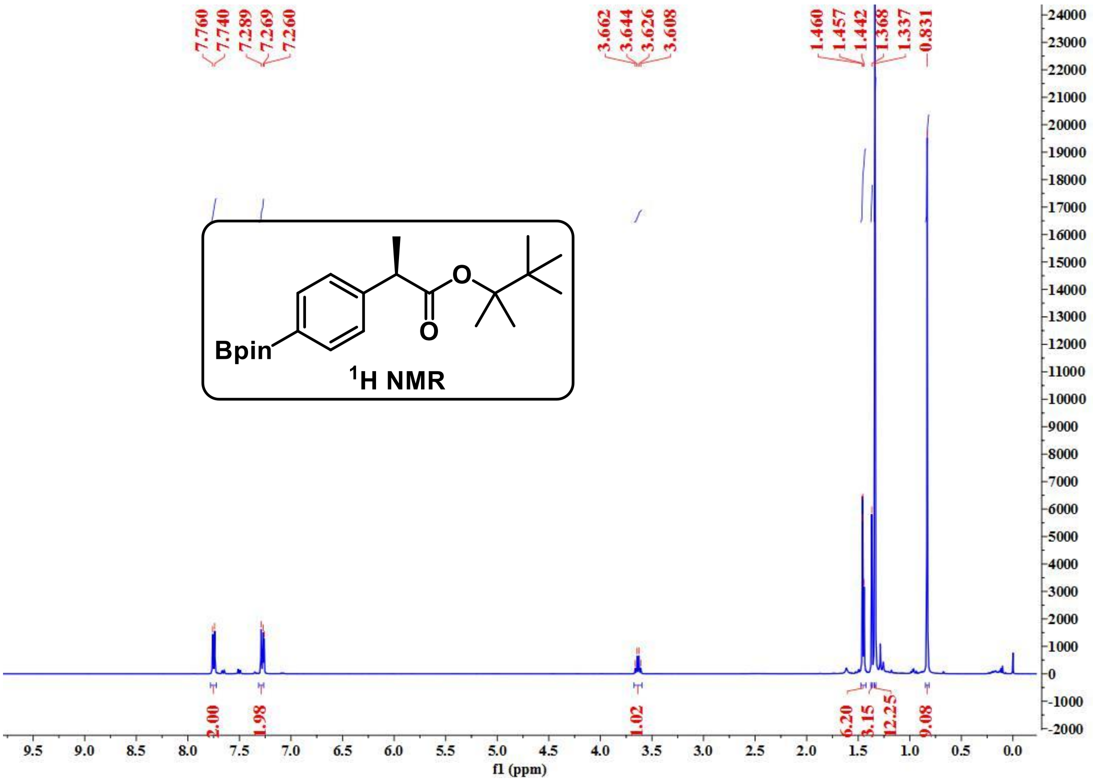

Supplementary Figure 71. ^{13}C NMR Spectrum of 2,3,3-trimethylbutan-2-yl (*R*)-2-(4-(4,4,5,5-tetramethyl-1,3,2-dioxaborolan-2-yl)phenyl)propanoate (101 MHz, CDCl$_3$)

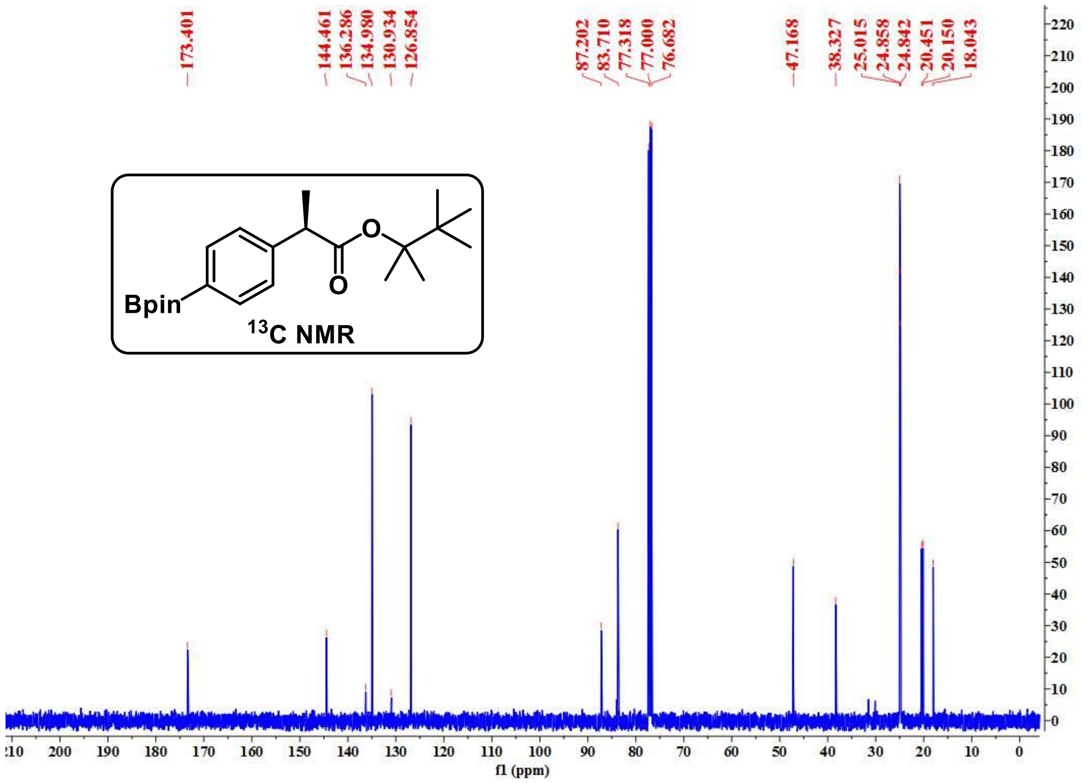
173.401
144.461
136.286
134.980
130.934
126.854
87.202
83.710
77.318
77.000
76.682
47.168
38.327
25.015
24.858
24.842
20.451
20.150
18.043
Bpin
O
O
13C NMR
f1 (ppm)

Supplementary Figure 72. ^{1}H NMR Spectrum of 2,3,3-trimethylbutan-2-yl (*R*)-2-(4-oxochroman-7-yl)propanoate (400 MHz, $CDCl_3$)

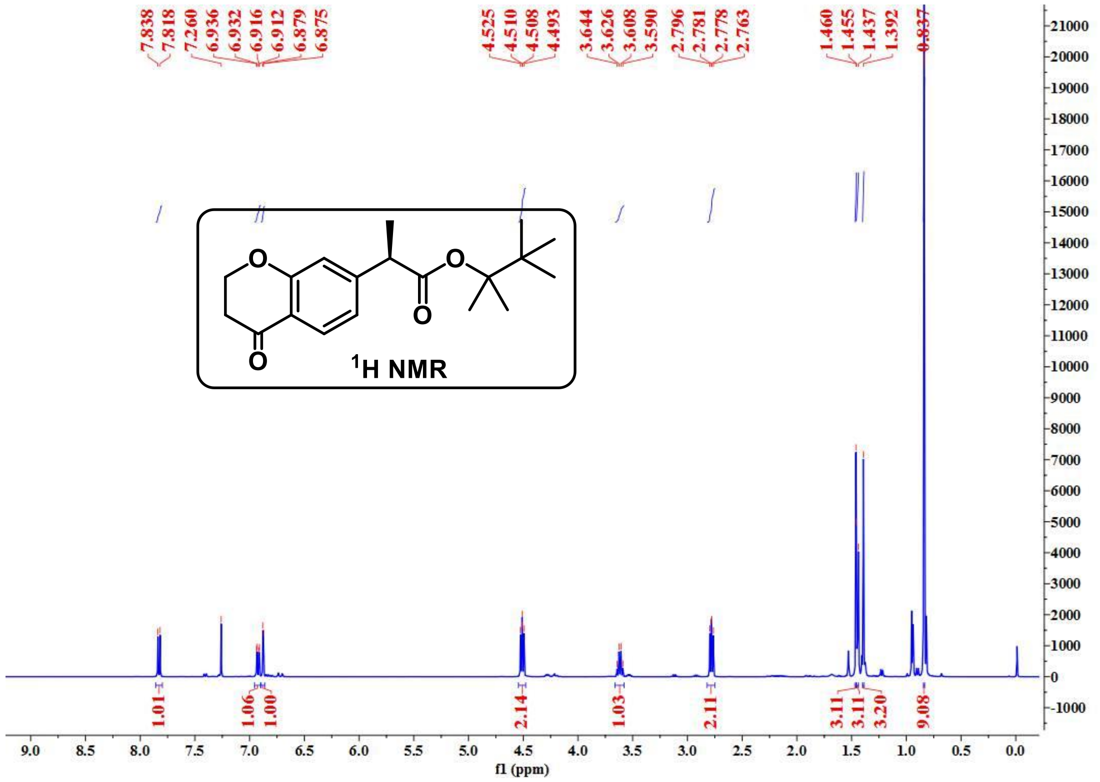

Supplementary Figure 73. ^{13}C NMR Spectrum of 2,3,3-trimethylbutan-2-yl (*R*)-2-(4-oxochroman-7-yl)propanoate (101 MHz, $CDCl_3$)

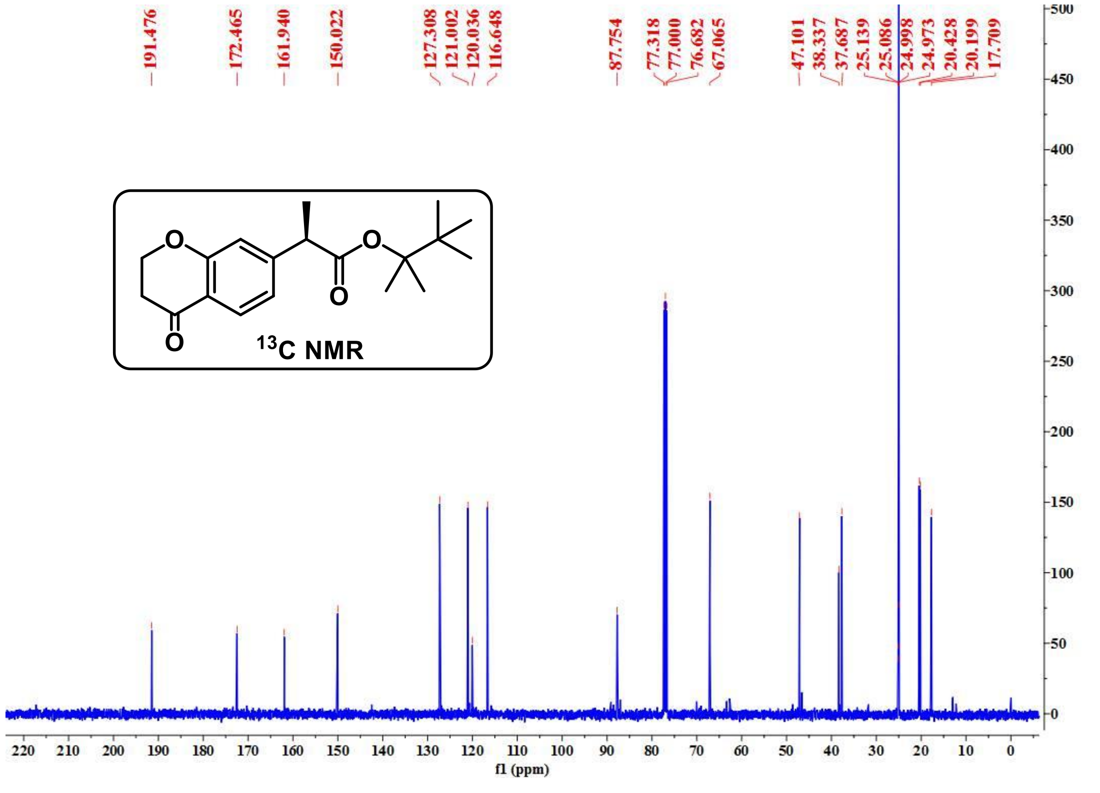

Supplementary Figure 74. ¹H NMR Spectrum of Ethyl (*R*)-5-(1-oxo-1-((2,3,3-trimethylbutan-2-yl)oxy)propan-2-yl)benzofuran-2-carboxylate (400 MHz, $CDCl_3$)

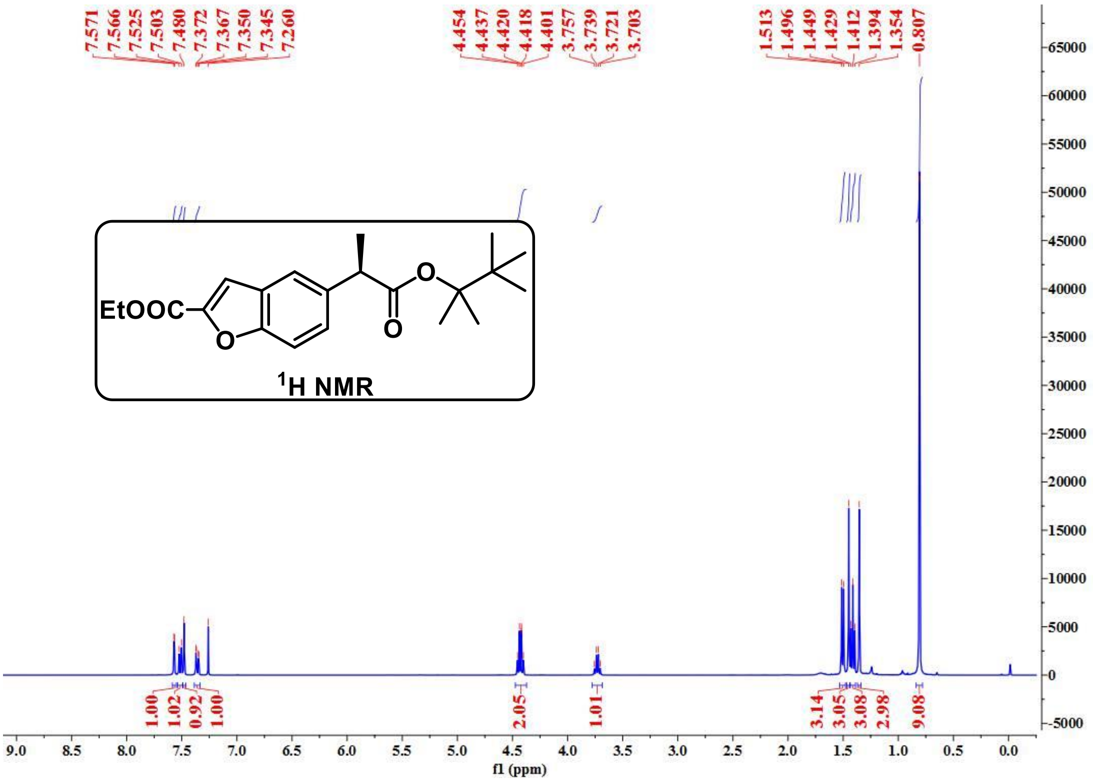

Supplementary Figure 75. ¹³C NMR Spectrum of Ethyl (*R*)-5-(1-oxo-1-((2,3,3-trimethylbutan-2-yl)oxy)propan-2-yl)benzofuran-2-carboxylate (101 MHz, $CDCl_3$)

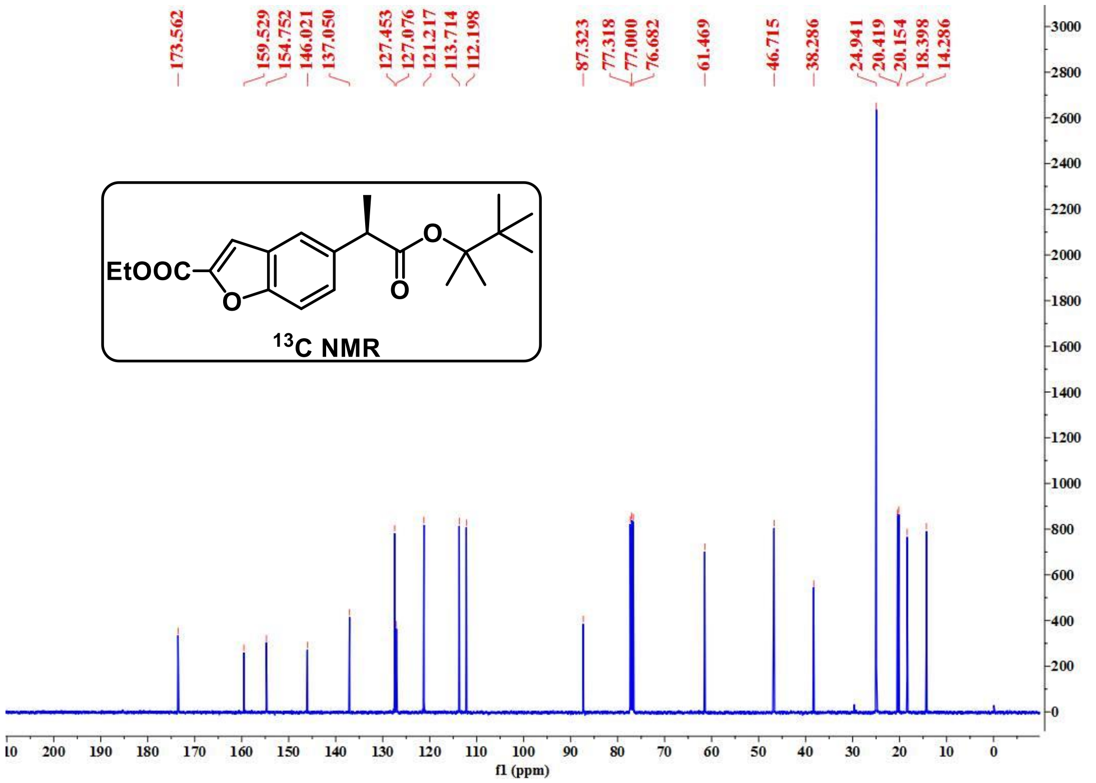

Supplementary Figure 76. ^{1}H NMR Spectrum of 2,3,3-trimethylbutan-2-yl (*R*)-2-(benzofuran-3-yl)propanoate (400 MHz, CDCl$_3$)

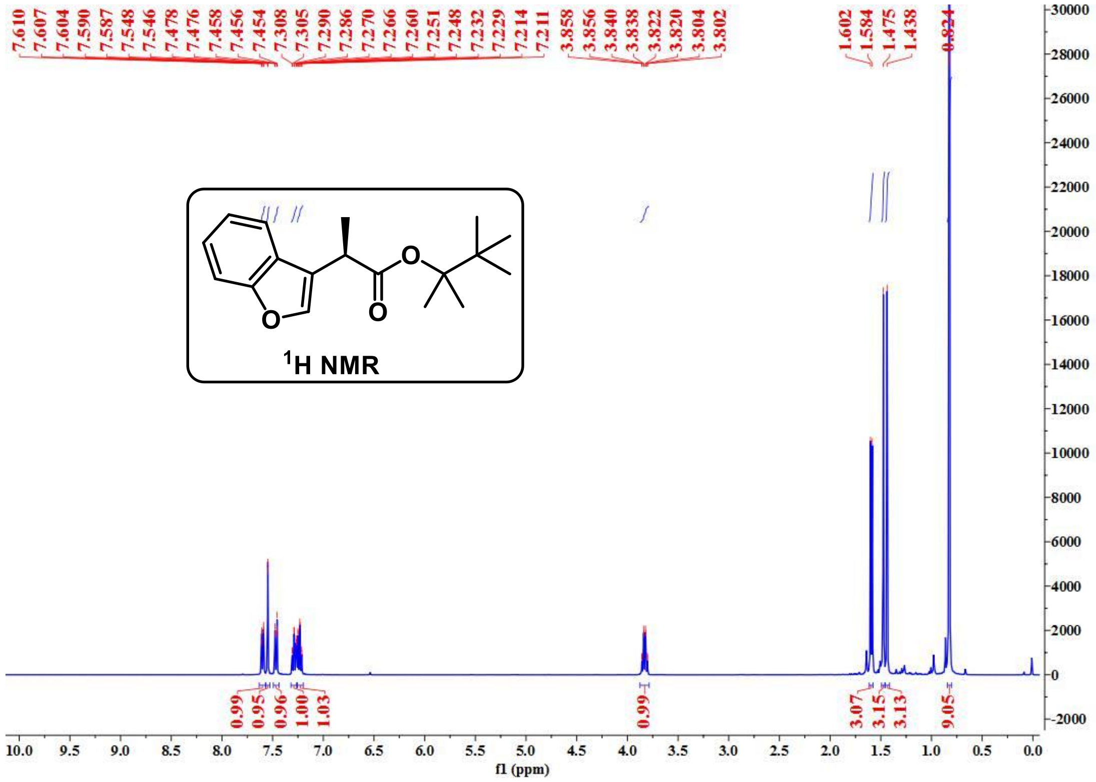

Supplementary Figure 77. ^{13}C NMR Spectrum of 2,3,3-trimethylbutan-2-yl (*R*)-2-(benzofuran-3-yl)propanoate (101 MHz, CDCl$_3$)

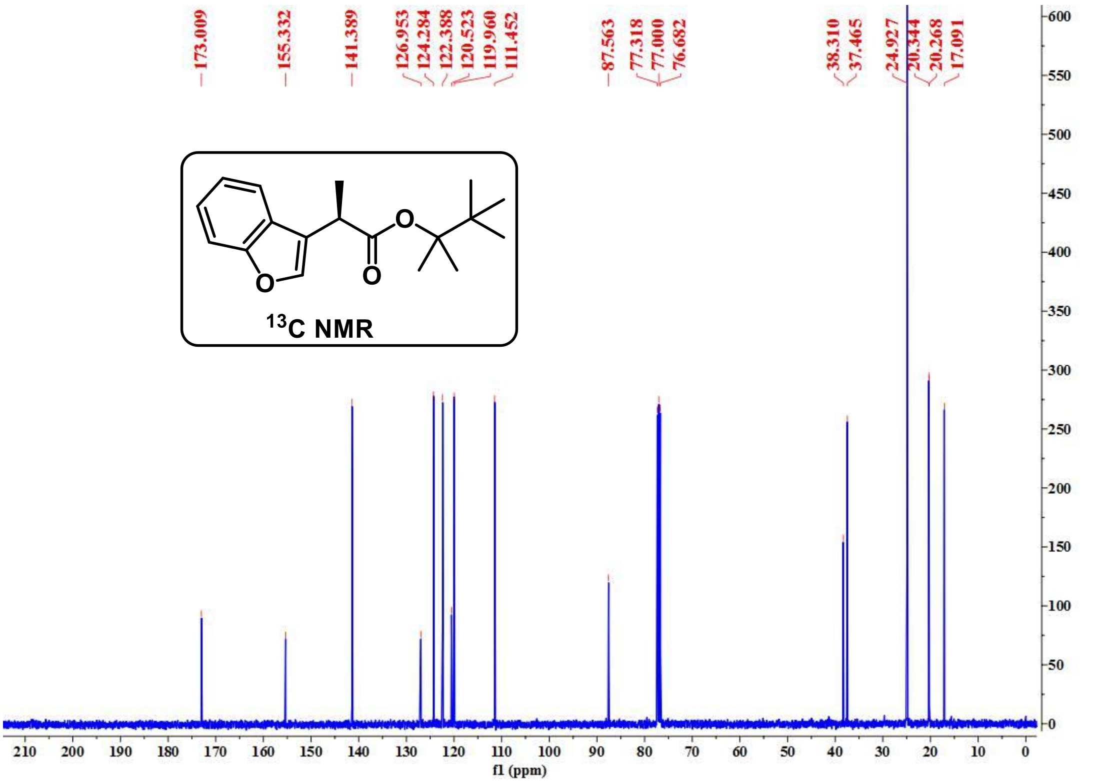

Supplementary Figure 78. ^{1}H NMR Spectrum of 2,3,3-trimethylbutan-2-yl (*R*)-2-(dibenzo[*b*,*d*]thiophen-3-yl)propanoate (400 MHz, $CDCl_3$)

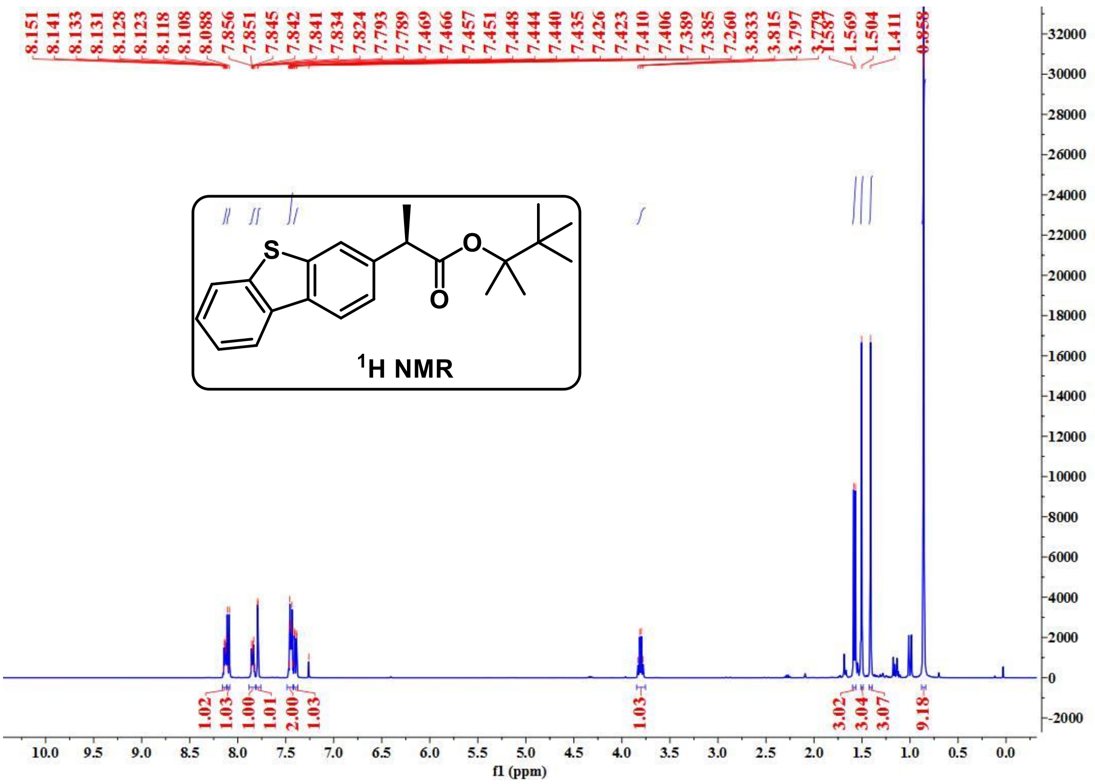

Supplementary Figure 79. ^{13}C NMR Spectrum of 2,3,3-trimethylbutan-2-yl (*R*)-2-(dibenzo[*b*,*d*]thiophen-3-yl)propanoate (101 MHz, $CDCl_3$)

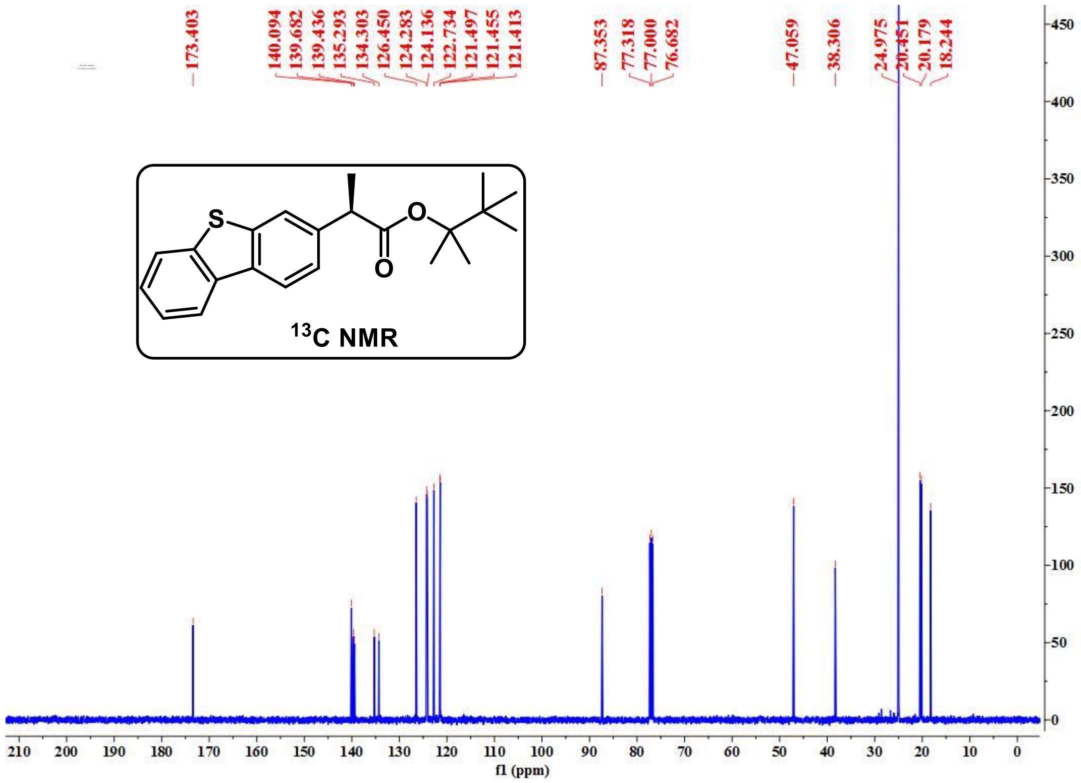

Supplementary Figure 80. ^{1}H NMR Spectrum of 2,3,3-trimethylbutan-2-yl (*R*)-2-(2-(methylthio)pyrimidin-5-yl)propanoate (400 MHz, $CDCl_3$)

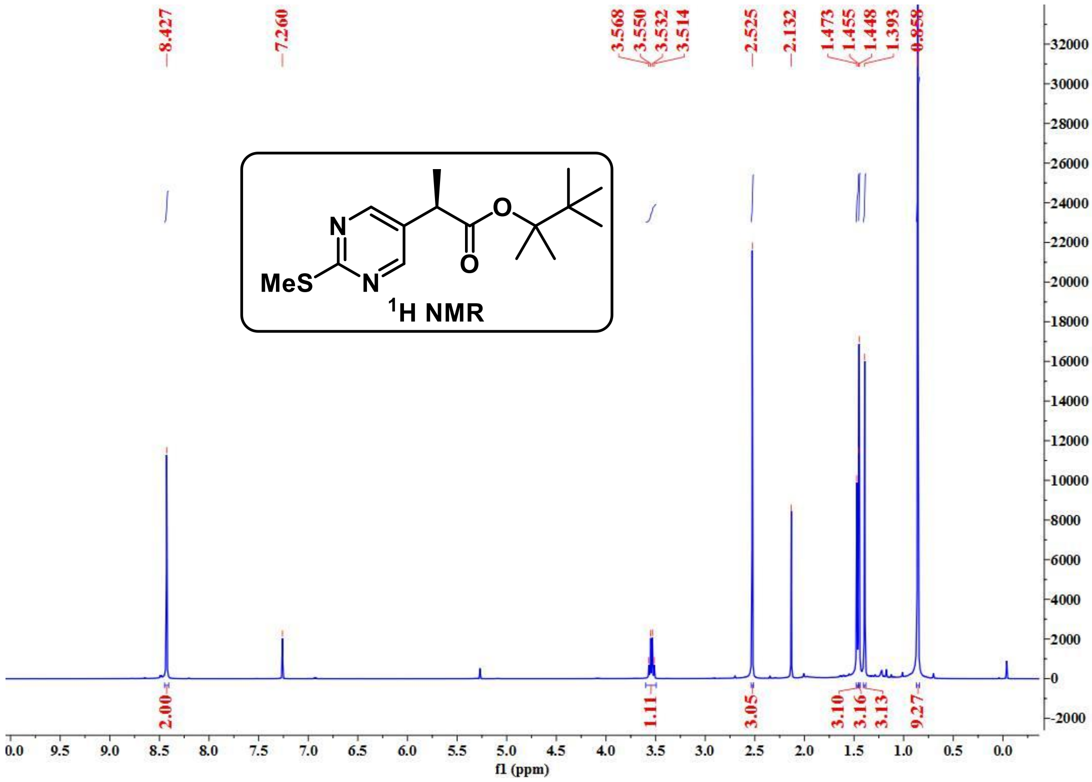

Supplementary Figure 81. ^{13}C NMR Spectrum of 2,3,3-trimethylbutan-2-yl (*R*)-2-(2-(methylthio)pyrimidin-5-yl)propanoate (101 MHz, $CDCl_3$)

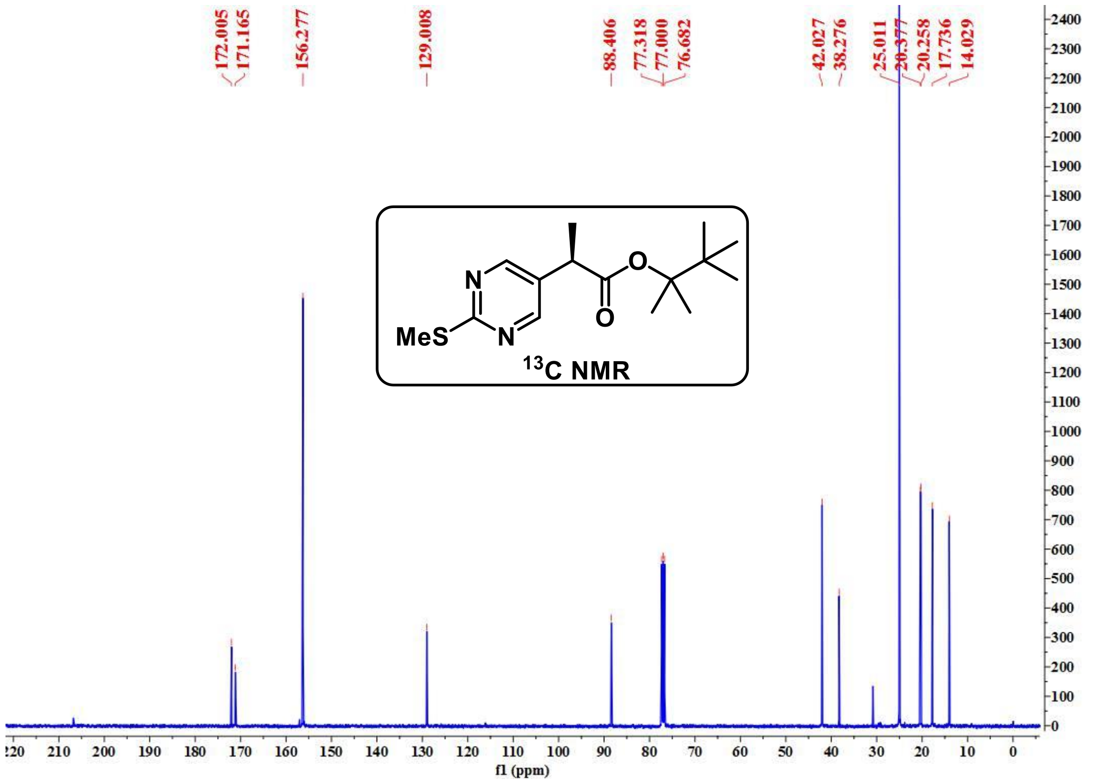

Supplementary Figure 82. ^{1}H NMR Spectrum of 2,3,3-trimethylbutan-2-yl (*R*)-2-(9*H*-fluoren-2-yl)propanoate (400 MHz, CDCl$_3$)

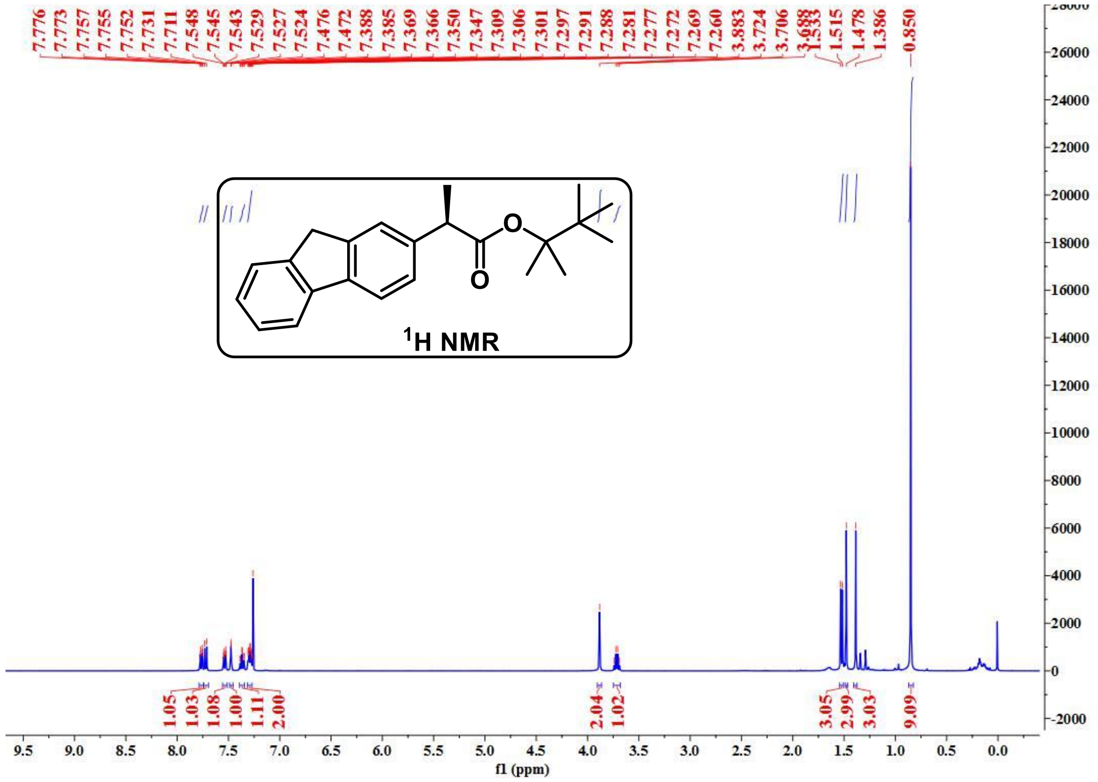

Supplementary Figure 83. ^{13}C NMR Spectrum of 2,3,3-trimethylbutan-2-yl (*R*)-2-(9*H*-fluoren-2-yl)propanoate (101 MHz, CDCl$_3$)

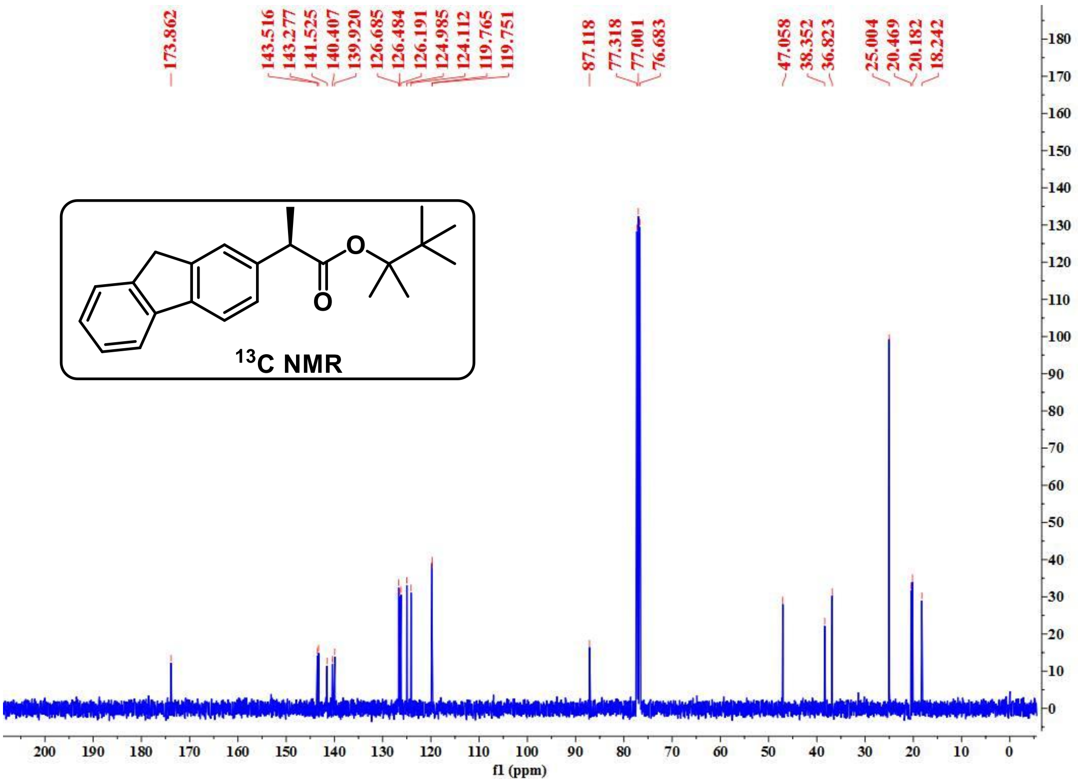

Supplementary Figure 84. ^{1}H NMR Spectrum of 2,3,3-trimethylbutan-2-yl (*R*,*E*)-4-(4-methoxyphenyl)-2-methylbut-3-enoate (400 MHz, $CDCl_3$)

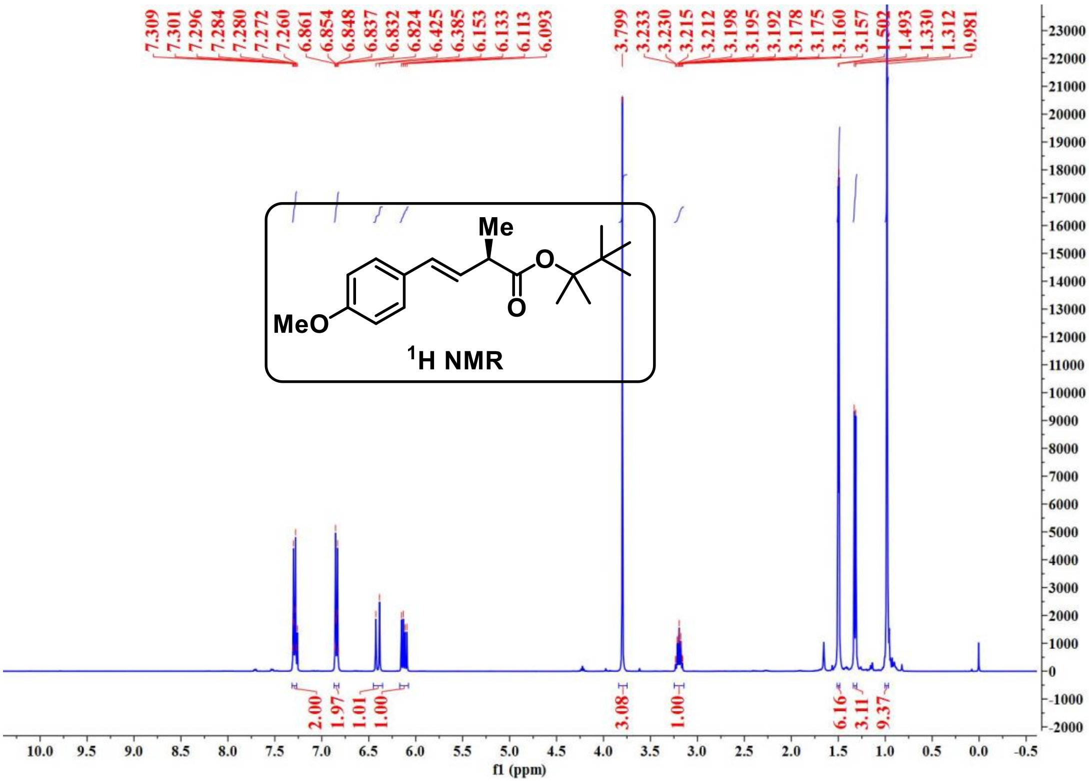

Supplementary Figure 85. ^{13}C NMR Spectrum of 2,3,3-trimethylbutan-2-yl (*R*,*E*)-4-(4-methoxyphenyl)-2-methylbut-3-enoate (101 MHz, $CDCl_3$)

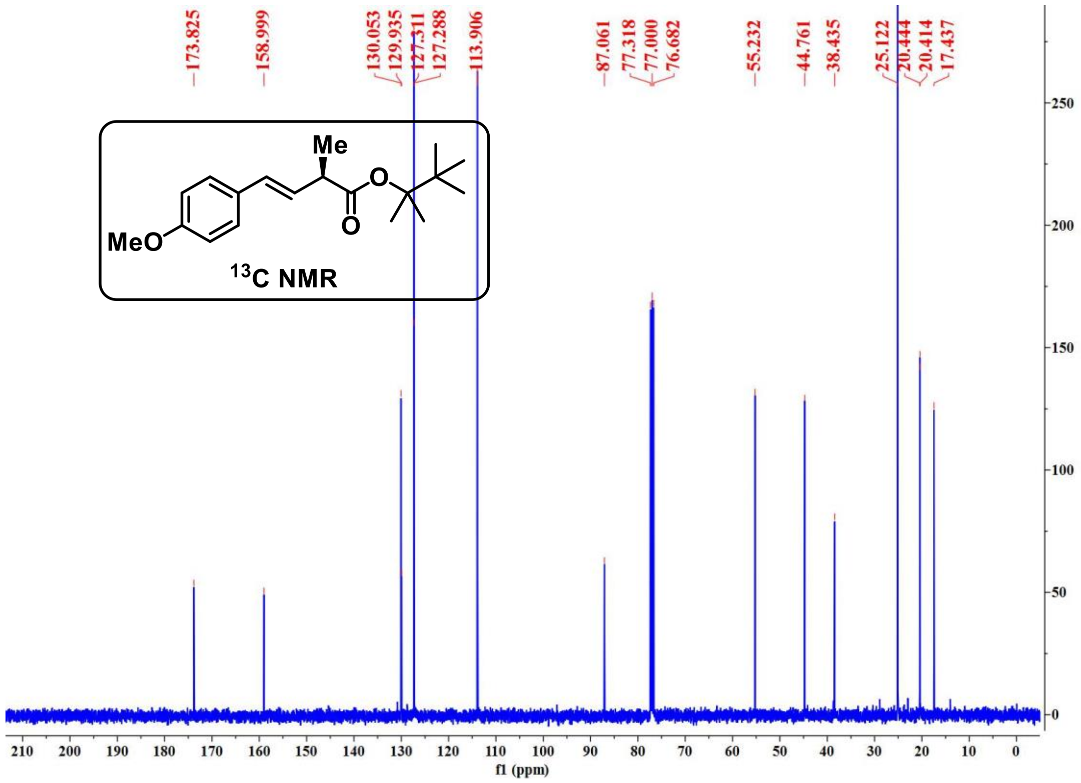
173.825
158.999
130.053
129.935
127.311
127.288
113.906
87.061
77.318
77.000
76.682
55.232
44.761
38.435
25.122
20.444
20.414
17.437
Me
O
O
MeO
13C NMR
f1 (ppm)

Supplementary Figure 86. ^{1}H NMR Spectrum: (*R*)-2,5,7,8-tetramethyl-2-((4*R*,8*R*)-4,8,12-trimethyltridecyl)chroman-6-yl 4-((*R*)-1-oxo-1-((2,3,3-trimethylbutan-2-yl)oxy)propan-2-yl)benzoate (400 MHz, $CDCl_3$)

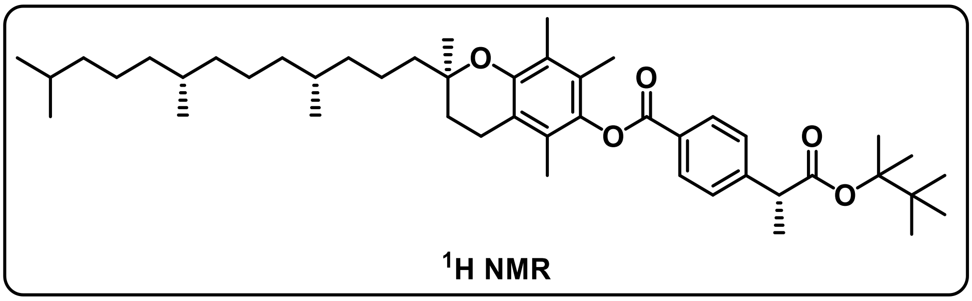

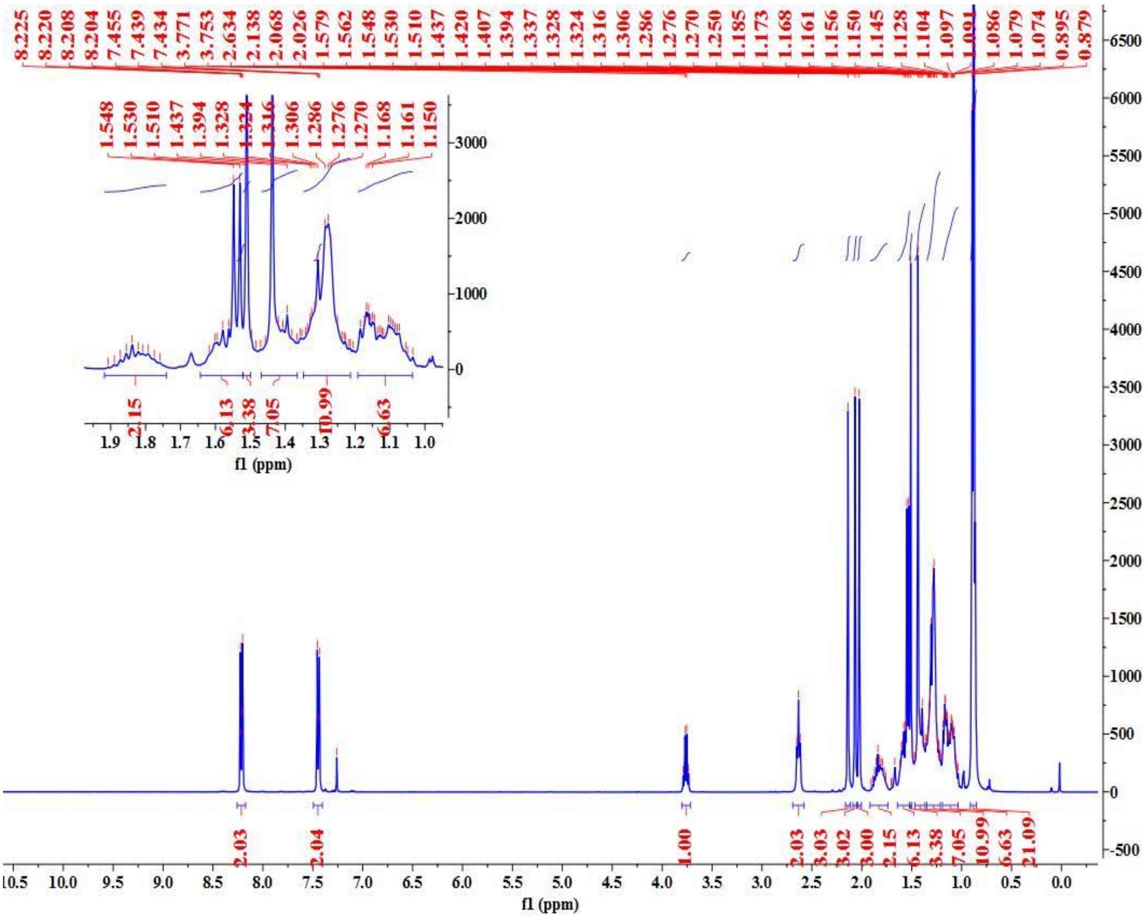

Supplementary Figure 87. ^{13}**C NMR Spectrum: (*R*)-2,5,7,8-tetramethyl-2-((4*R*,8*R*)-4,8,12-trimethyltridecyl)chroman-6-yl 4-((*R*)-1-oxo-1-((2,3,3-trimethylbutan-2-yl)oxy)propan-2-yl)benzoate (101 MHz, $CDCl_3$)**

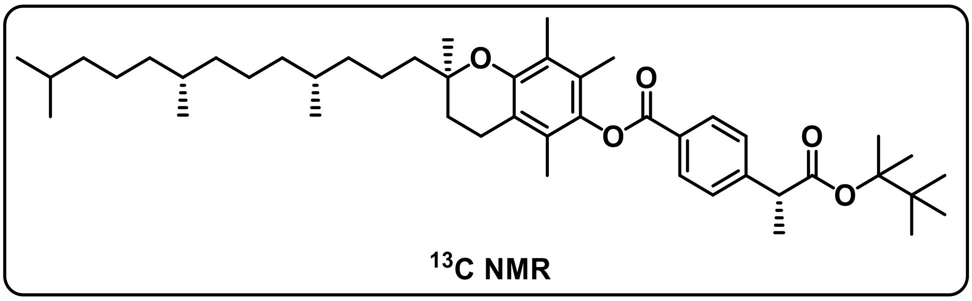

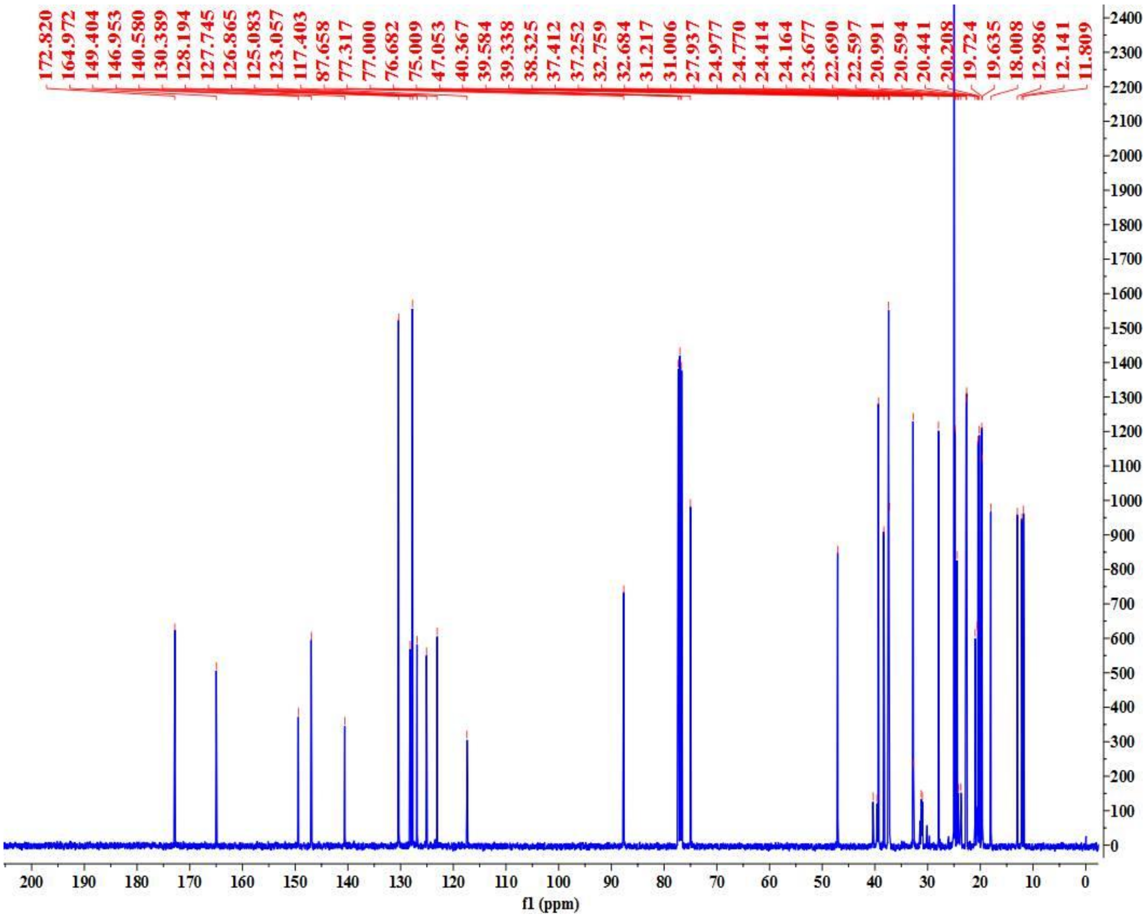

Supplementary Figure 88. ^{1}H NMR Spectrum of 2,3,3-trimethylbutan-2-yl (*R*)-2-(3-((5-(4-fluorophenyl)thiophen-2-yl)methyl)-4-methylphenyl)propanoate (400 MHz)

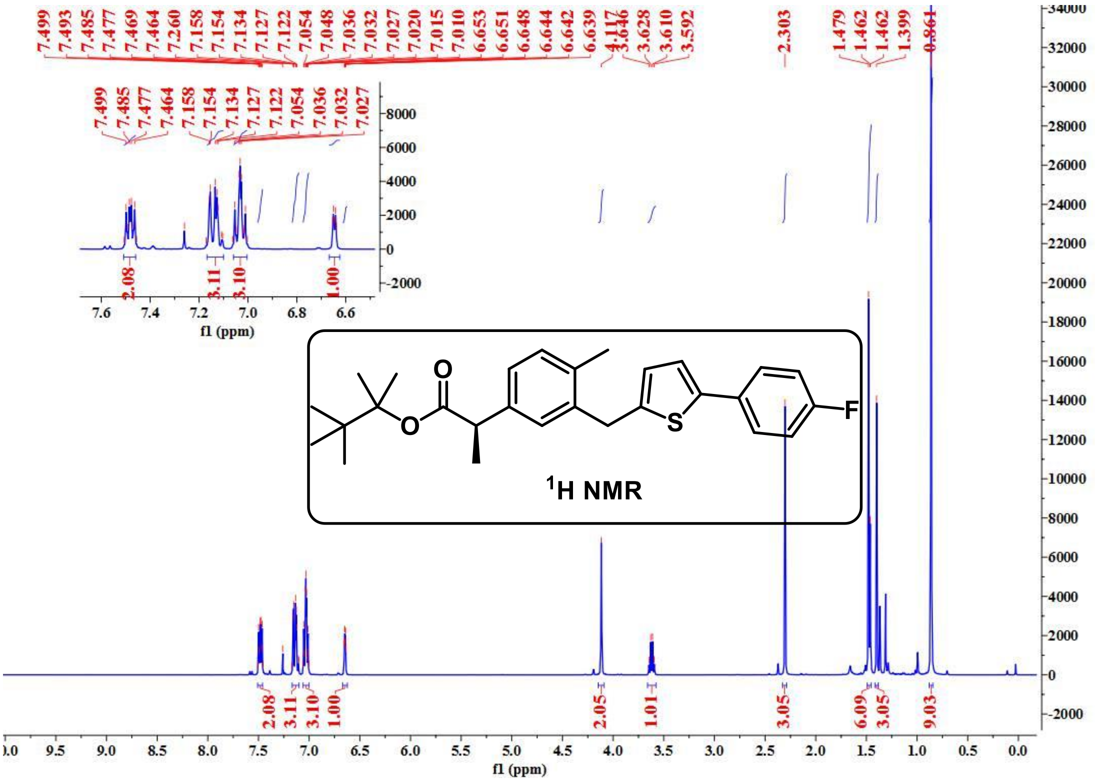

Supplementary Figure 89. ^{19}F NMR Spectrum of 2,3,3-trimethylbutan-2-yl (*R*)-2-(3-((5-(4-fluorophenyl)thiophen-2-yl)methyl)-4-methylphenyl)propanoate (375 MHz)

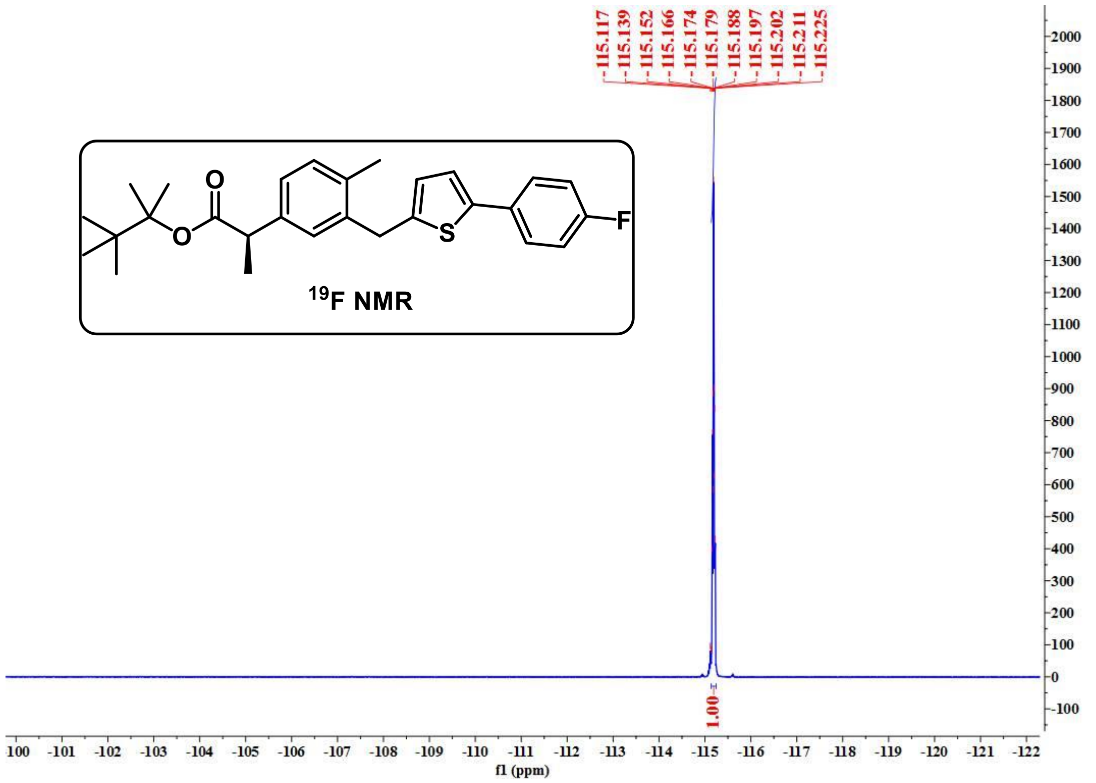

Supplementary Figure 90. ^{13}C NMR Spectrum of 2,3,3-trimethylbutan-2-yl (*R*)-2-(3-((5-(4-fluorophenyl)thiophen-2-yl)methyl)-4-methylphenyl)propanoate (101 MHz)

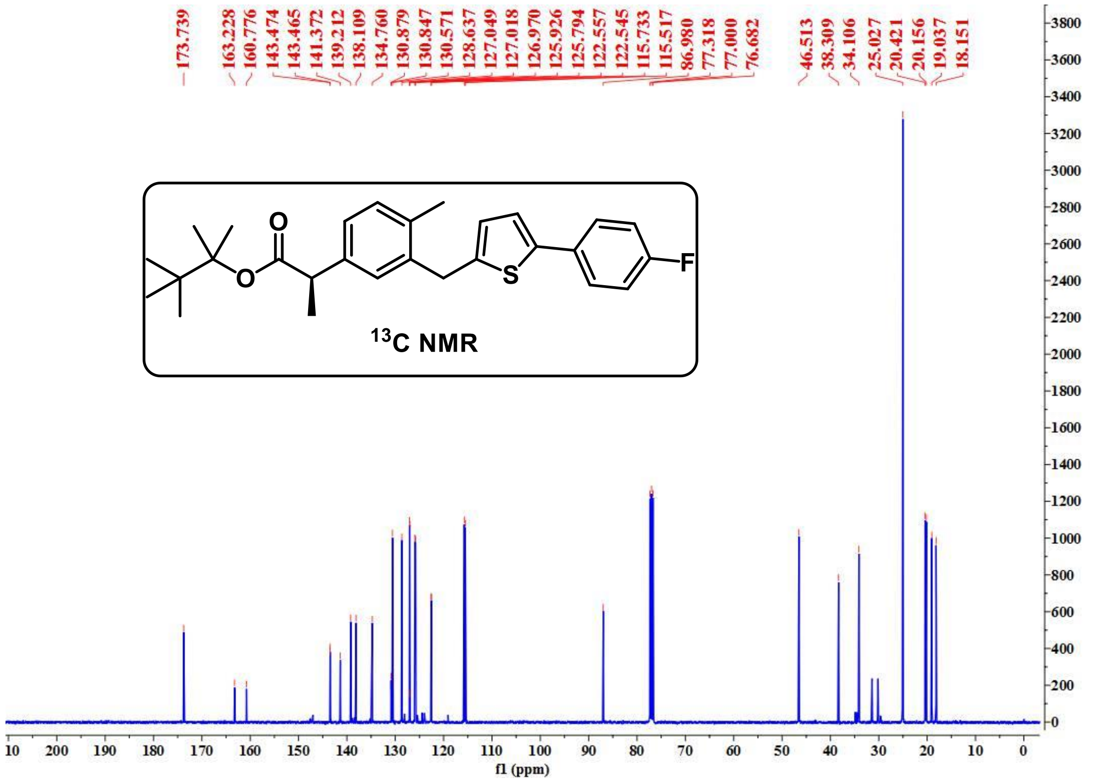

Supplementary Figure 91. ^{1}H NMR Spectrum of 2,3,3-trimethylbutan-2-yl (*R*)-2-(4-(*N*-(2,6-dimethoxypyrimidin-4-yl)-*N*-methylsulfamoyl)phenyl)propanoate (400 MHz)

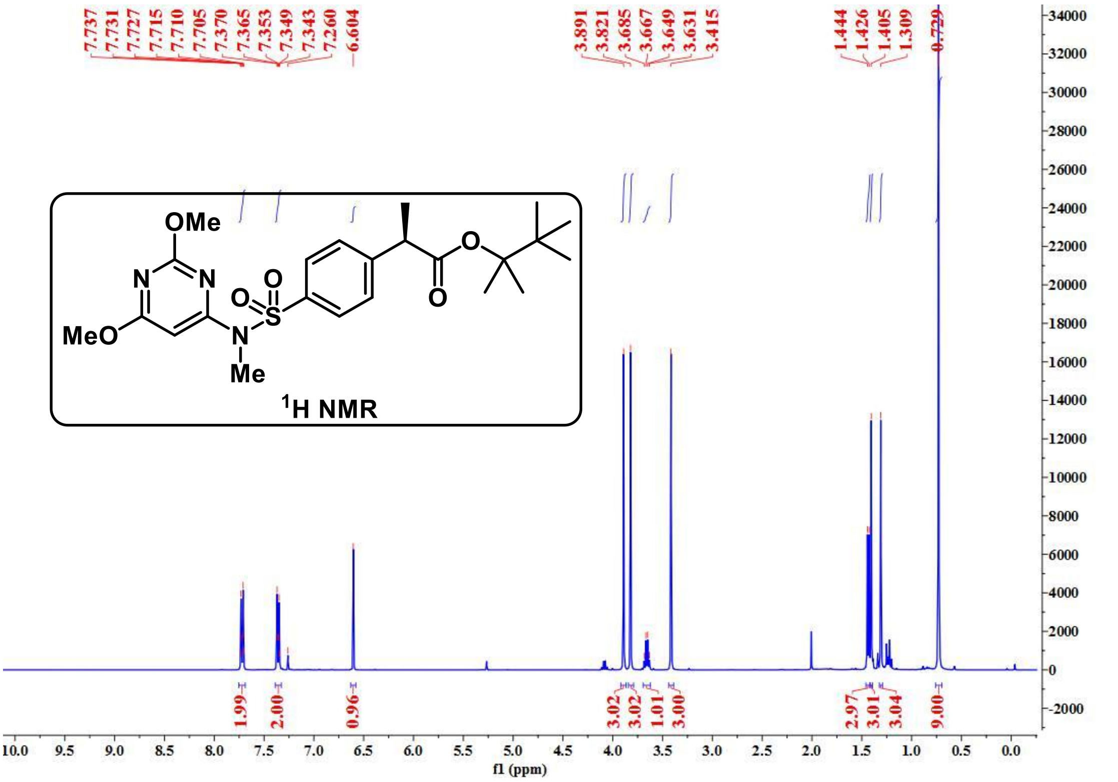

Supplementary Figure 92. ^{13}C NMR Spectrum of 2,3,3-trimethylbutan-2-yl (*R*)-2-(4-(*N*-(2,6-dimethoxypyrimidin-4-yl)-*N*-methylsulfamoyl)phenyl)propanoate (101 MHz)

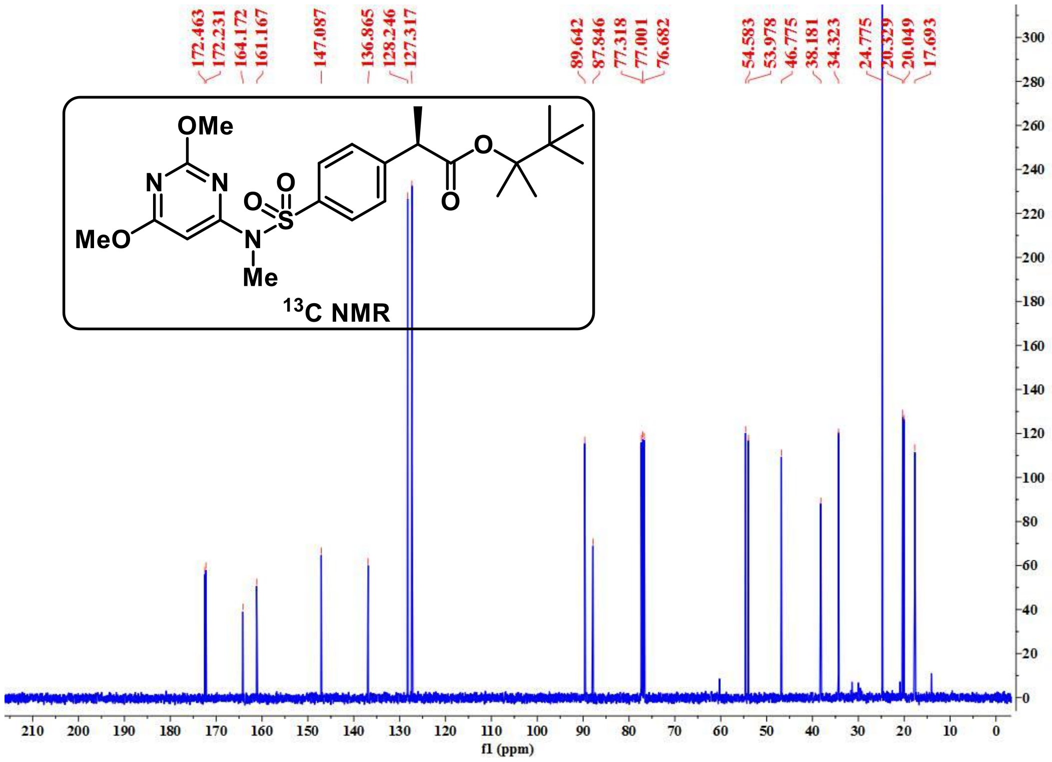

Supplementary Figure 93. ^{1}H NMR Spectrum: 2,3,3-trimethylbutan-2-yl (*R*)-2-(4-(5-methoxy-3-(2-methoxy-2-oxoethyl)-2-methyl-1*H*-indole-1-carbonyl)phenyl)propanoate (400 MHz)

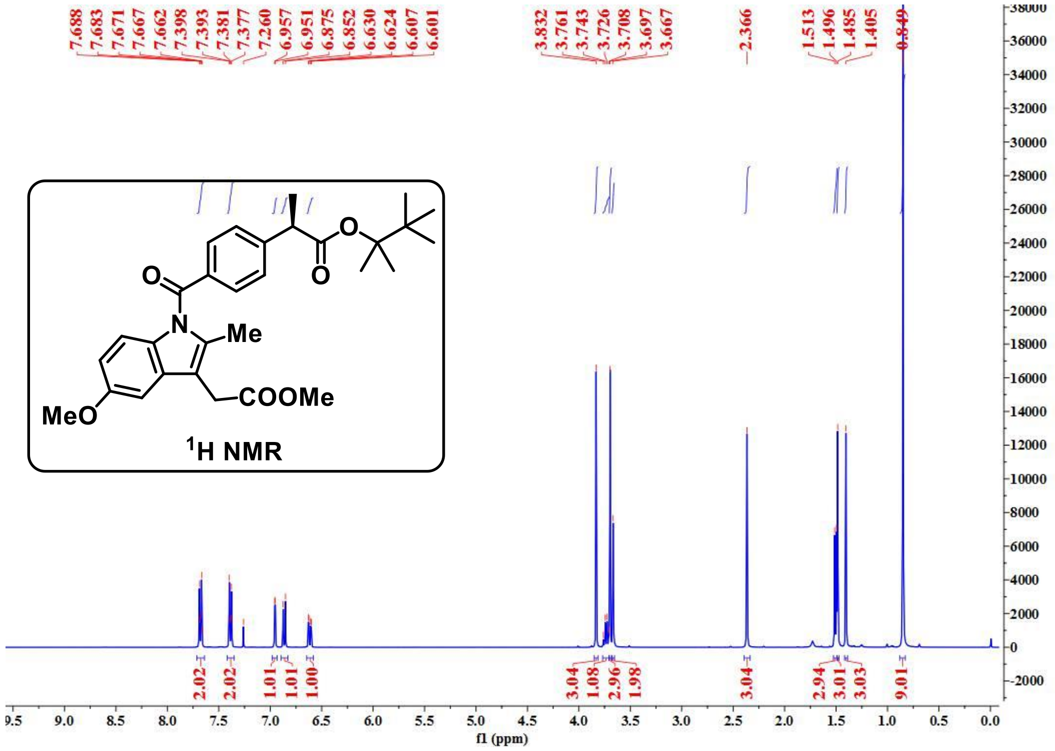

Supplementary Figure 94. ^{13}**C NMR Spectrum: 2,3,3-trimethylbutan-2-yl (*R*)-2-(4-(5-methoxy-3-(2-methoxy-2-oxoethyl)-2-methyl-1*H*-indole-1-carbonyl)phenyl)propanoate**

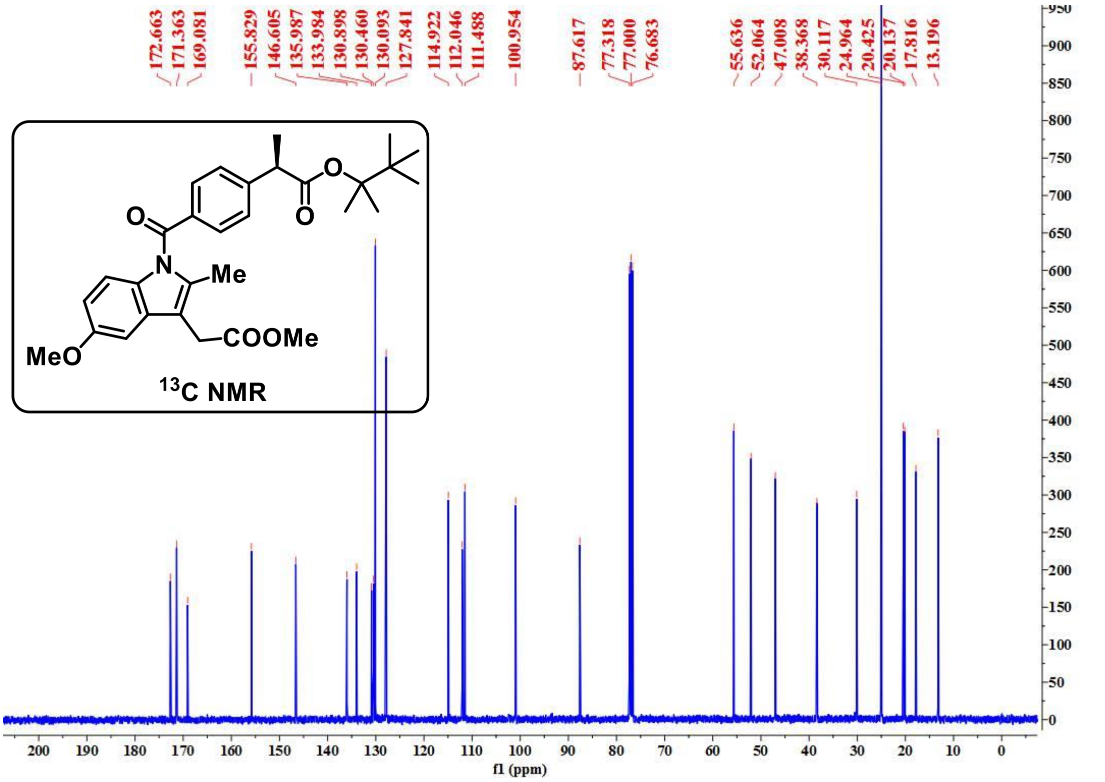

Supplementary Figure 95. ^{1}**H NMR Spectrum: (1*R*,2*S*,5*R*)-2-isopropyl-5-methylcyclohexyl 4-((*R*)-1-oxo-1-((2,3,3-trimethylbutan-2-yl)oxy)propan-2-yl)benzoate (400 MHz, $CDCl_3$)**

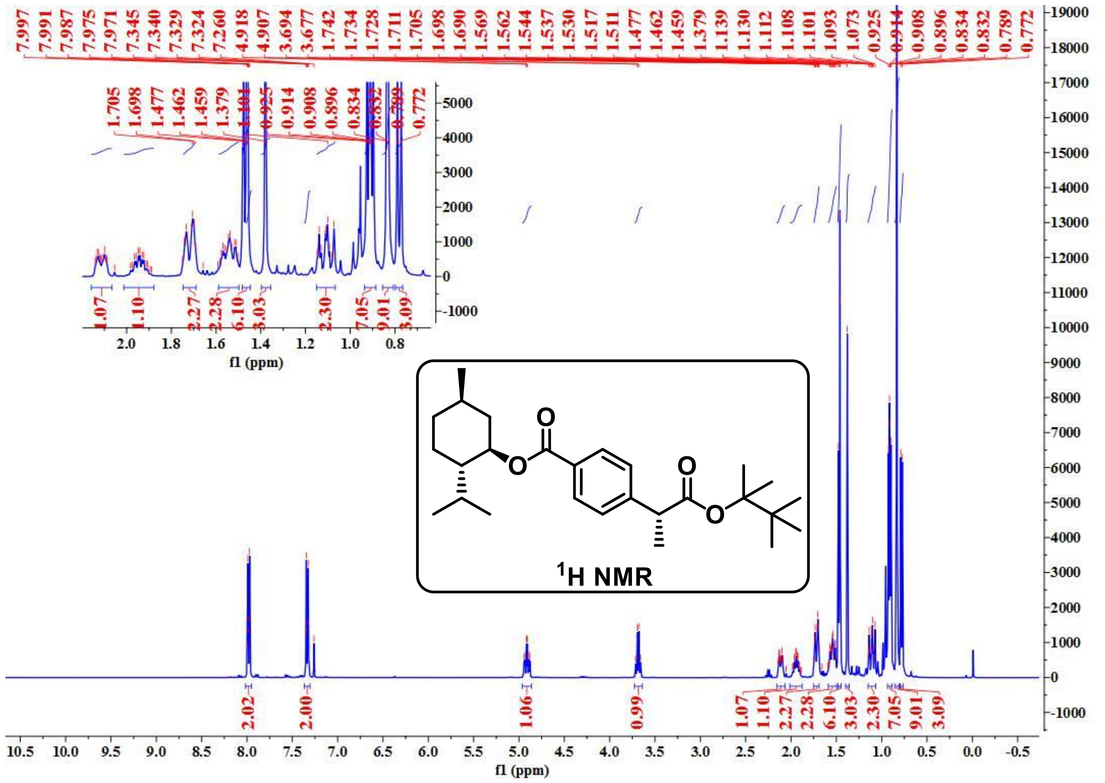

Supplementary Figure 96. ^{13}C NMR Spectrum: (1*R*,2*S*,5*R*)-2-isopropyl-5-methylcyclohexyl 4-((*R*)-1-oxo-1-((2,3,3-trimethylbutan-2-yl)oxy)propan-2-yl)benzoate (101 MHz, $CDCl_3$)

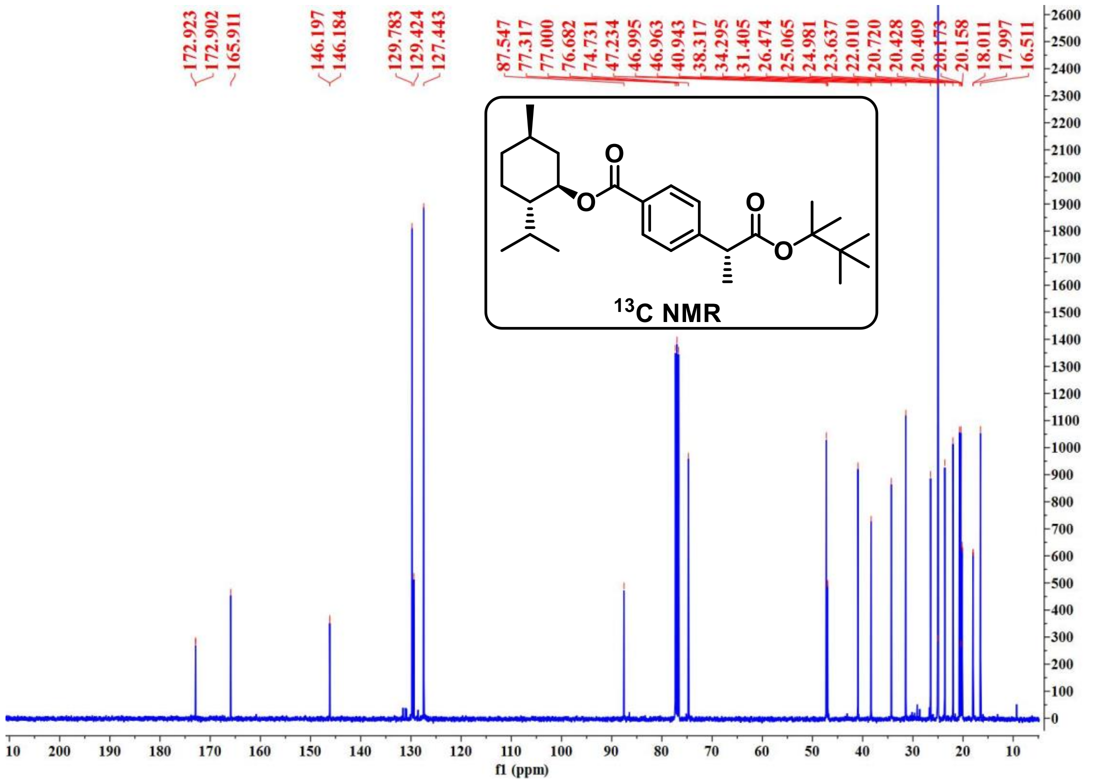

Supplementary Figure 97. ¹H NMR Spectrum of (3a*R*,5*R*,6*S*,6a*R*)-5-((*R*)-2,2-dimethyl-1,3-dioxolan-4-yl)-2,2-dimethyltetrahydrofuro[2,3-*d*][1,3]dioxol-6-yl 4-((*R*)-1-oxo-1-((2,3,3-trimethylbutan-2-yl)oxy)propan-2-yl)benzoate (400 MHz, $CDCl_3$)

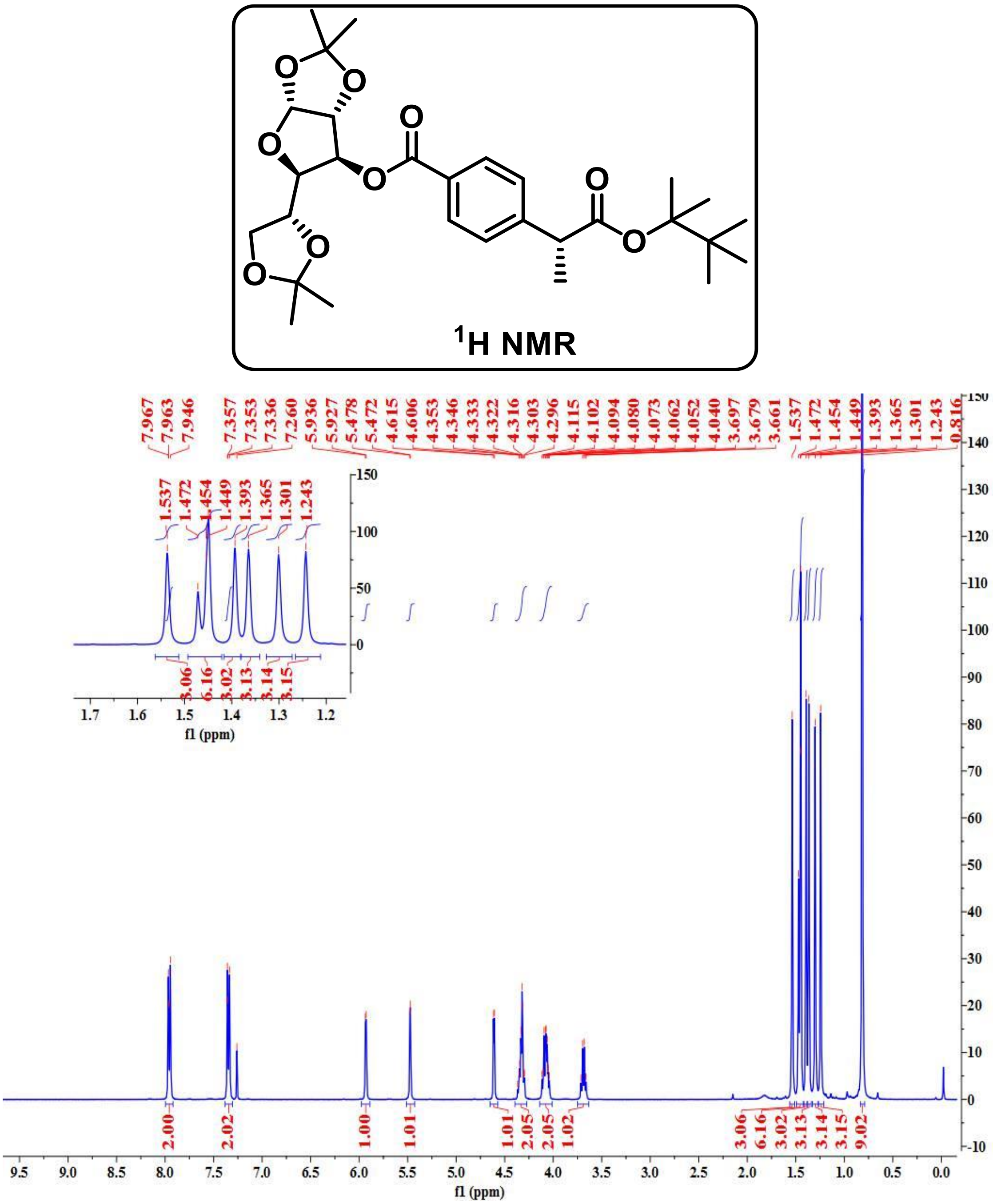

Supplementary Figure 98. ^{13}**C NMR Spectrum of (3a*R*,5*R*,6*S*,6a*R*)-5-((*R*)-2,2-dimethyl-1,3-dioxolan-4-yl)-2,2-dimethyltetrahydrofuro[2,3-*d*][1,3]dioxol-6-yl 4-((*R*)-1-oxo-1-((2,3,3-trimethylbutan-2-yl)oxy)propan-2-yl)benzoate (101 MHz, $CDCl_3$)**

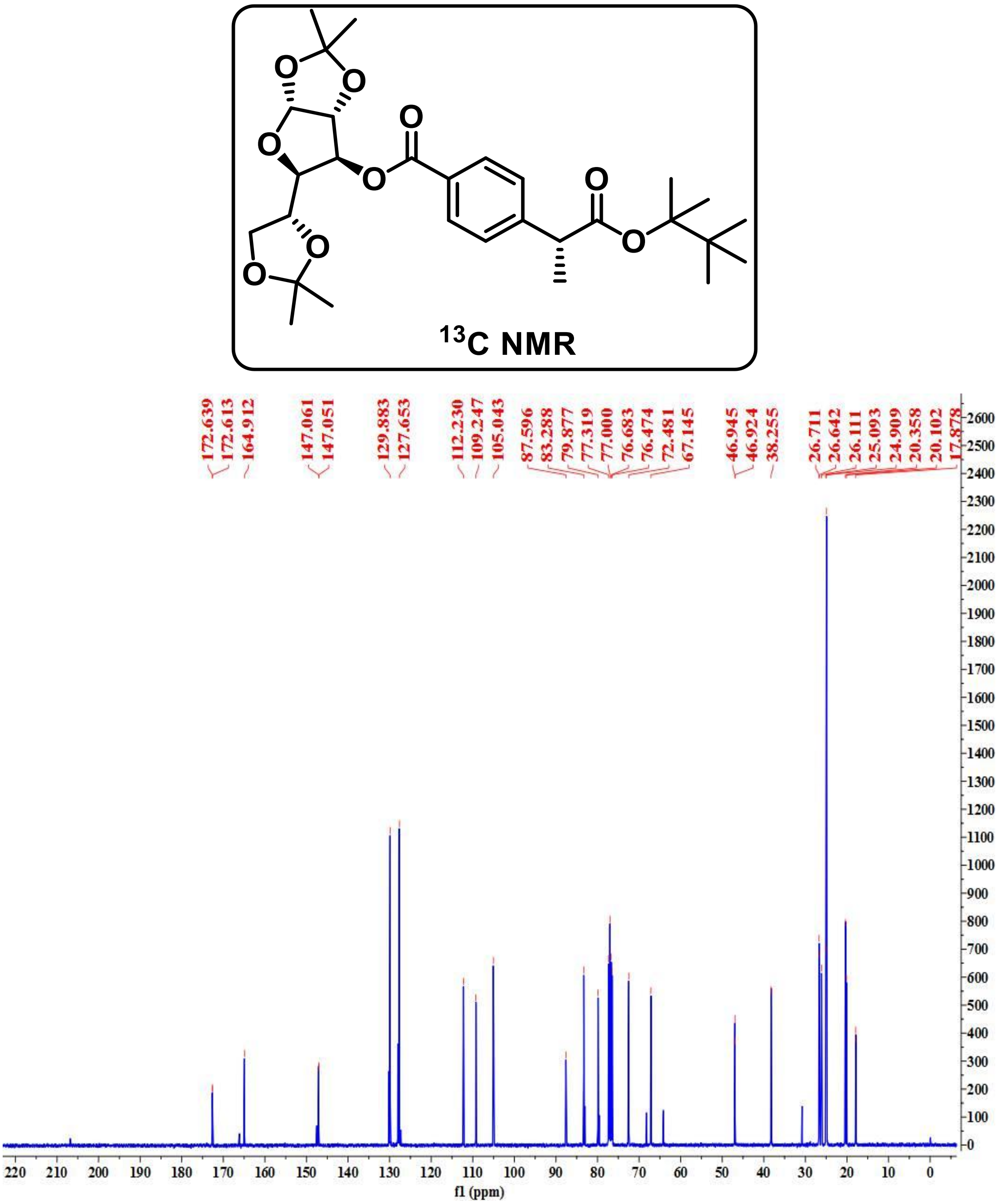

Supplementary Figure 99. ^{1}**H NMR Spectrum of 2,3,3-trimethylbutan-2-yl (*R*)-2-(6-methoxynaphthalen-2-yl)propanoate (400 MHz, $CDCl_3$)**

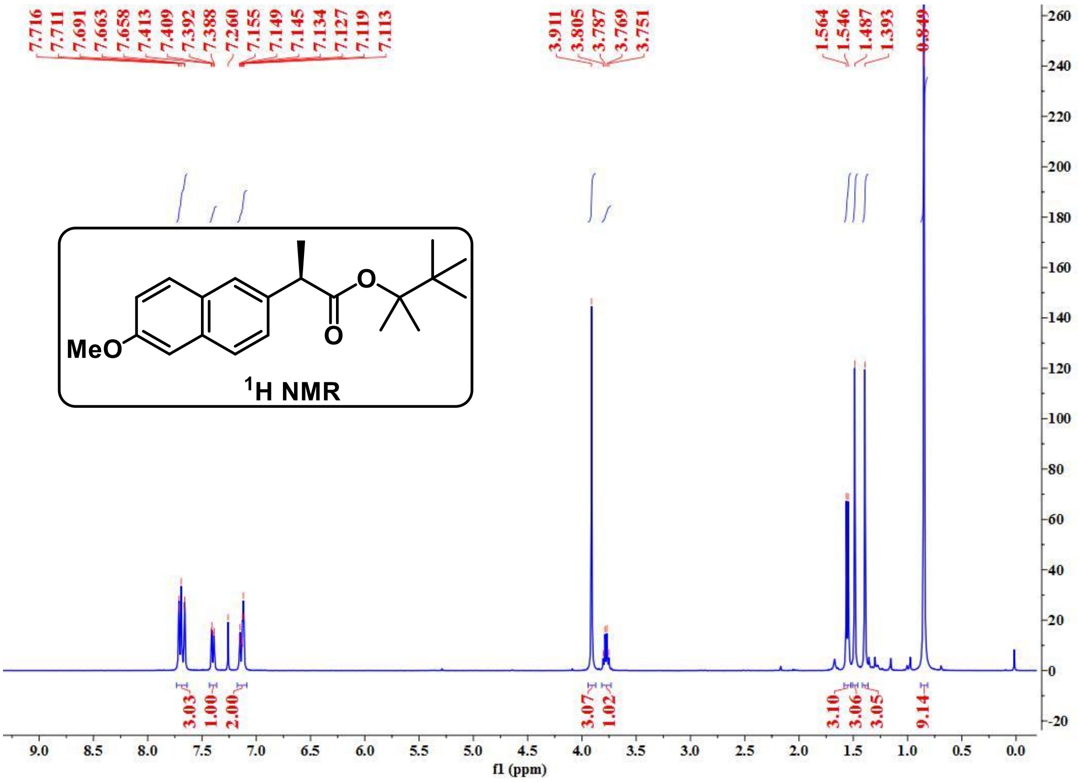

Supplementary Figure 100. ^{13}**C NMR Spectrum of 2,3,3-trimethylbutan-2-yl (*R*)-2-(6-methoxynaphthalen-2-yl)propanoate (101 MHz, $CDCl_3$)**

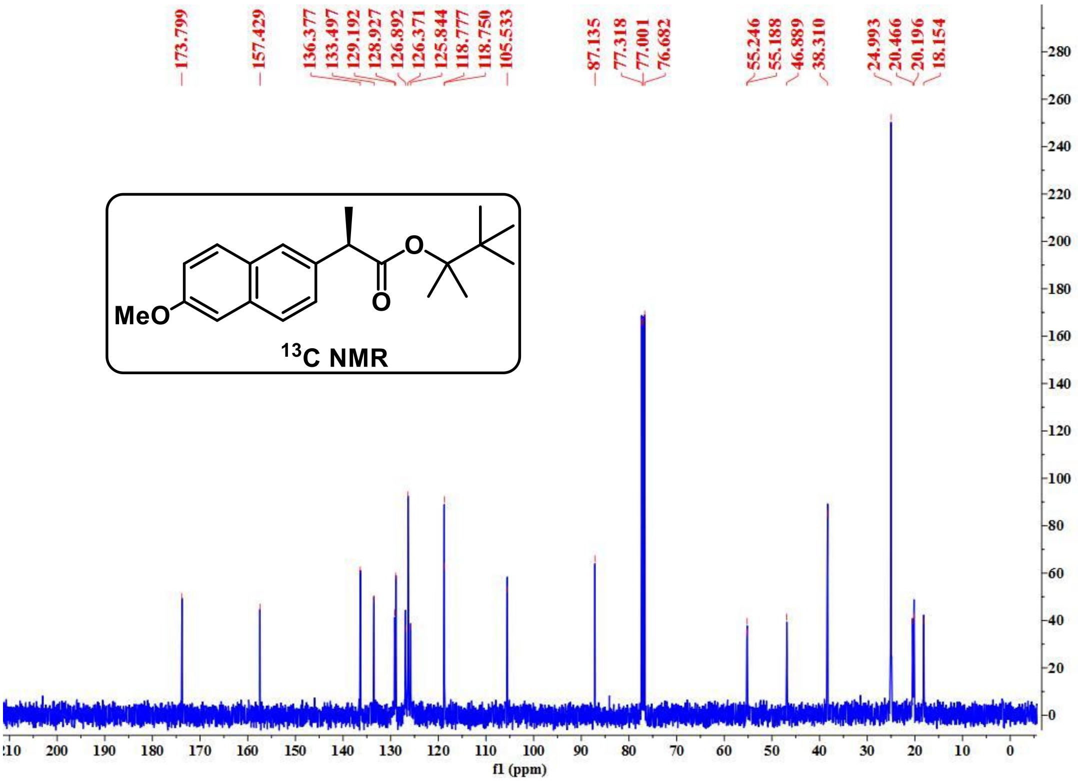

Supplementary Figure 101. ^{1}H NMR Spectrum of 2,3,3-trimethylbutan-2-yl (*R*)-2-(4-isobutylphenyl)propanoate (400 MHz, $CDCl_3$)

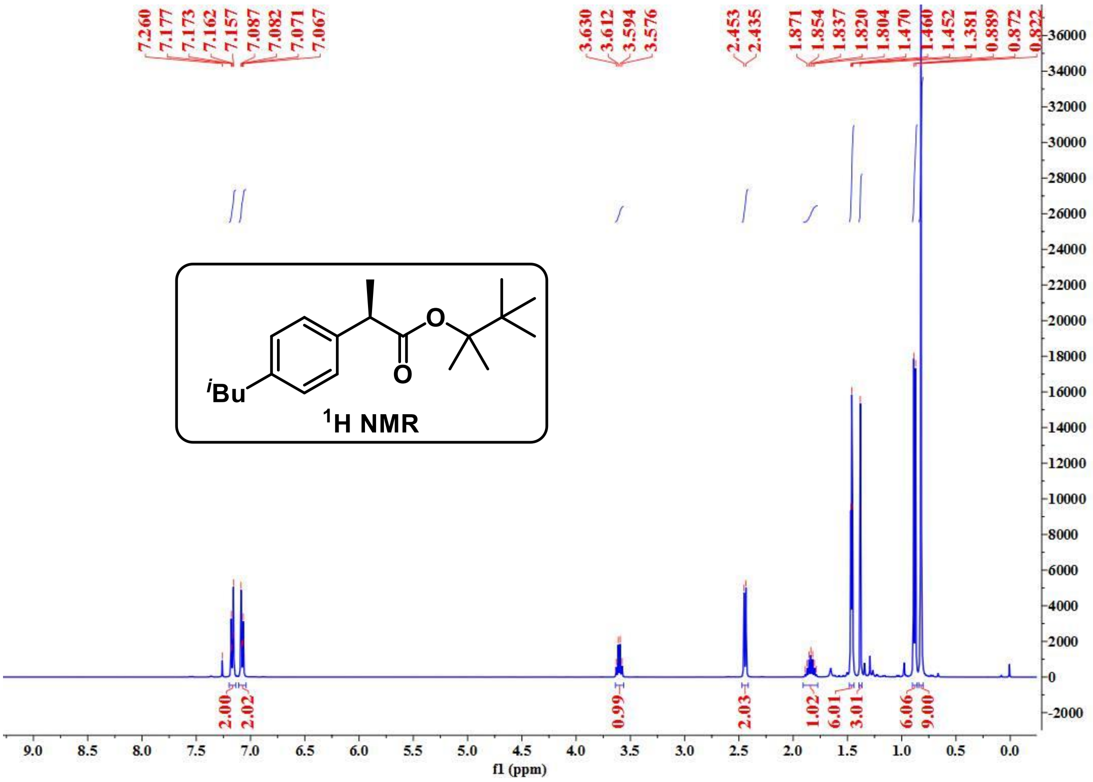

Supplementary Figure 102. ^{13}C NMR Spectrum of 2,3,3-trimethylbutan-2-yl (*R*)-2-(4-isobutylphenyl)propanoate (101 MHz, $CDCl_3$)

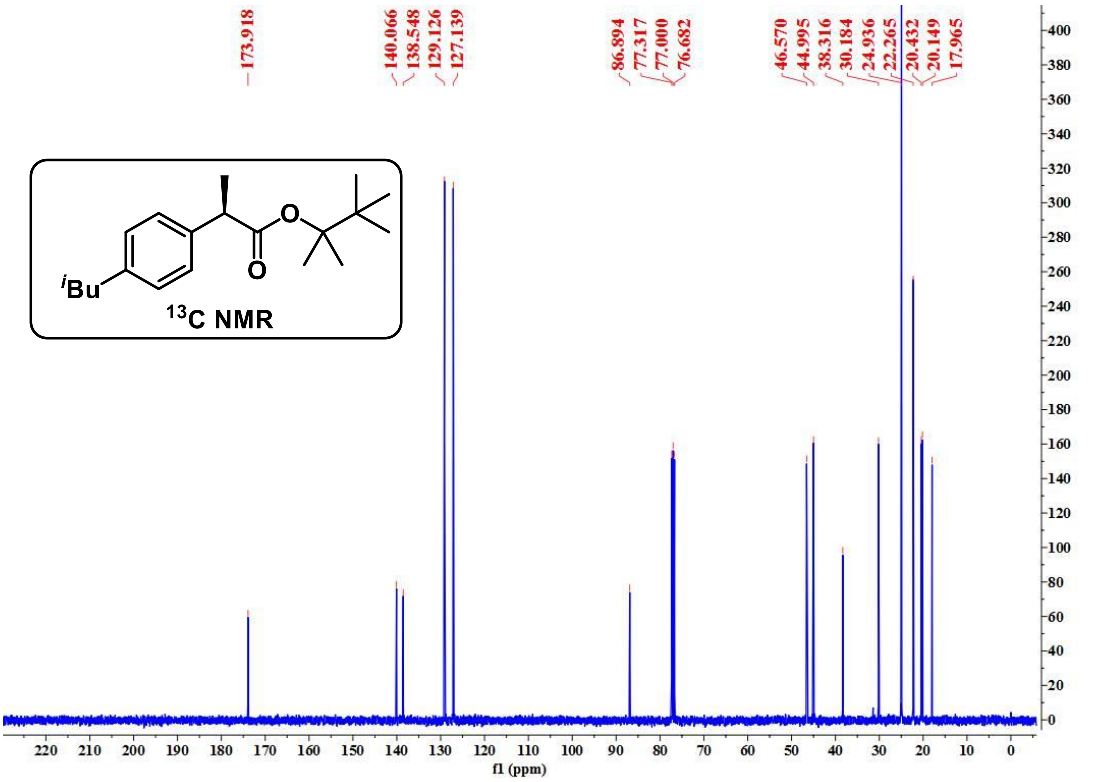

Supplementary Figure 103. ^{1}**H NMR Spectrum of 2,3,3-trimethylbutan-2-yl (*R*)-2-(2-fluoro-[1,1'-biphenyl]-4-yl)propanoate (400 MHz, CDCl$_3$)**

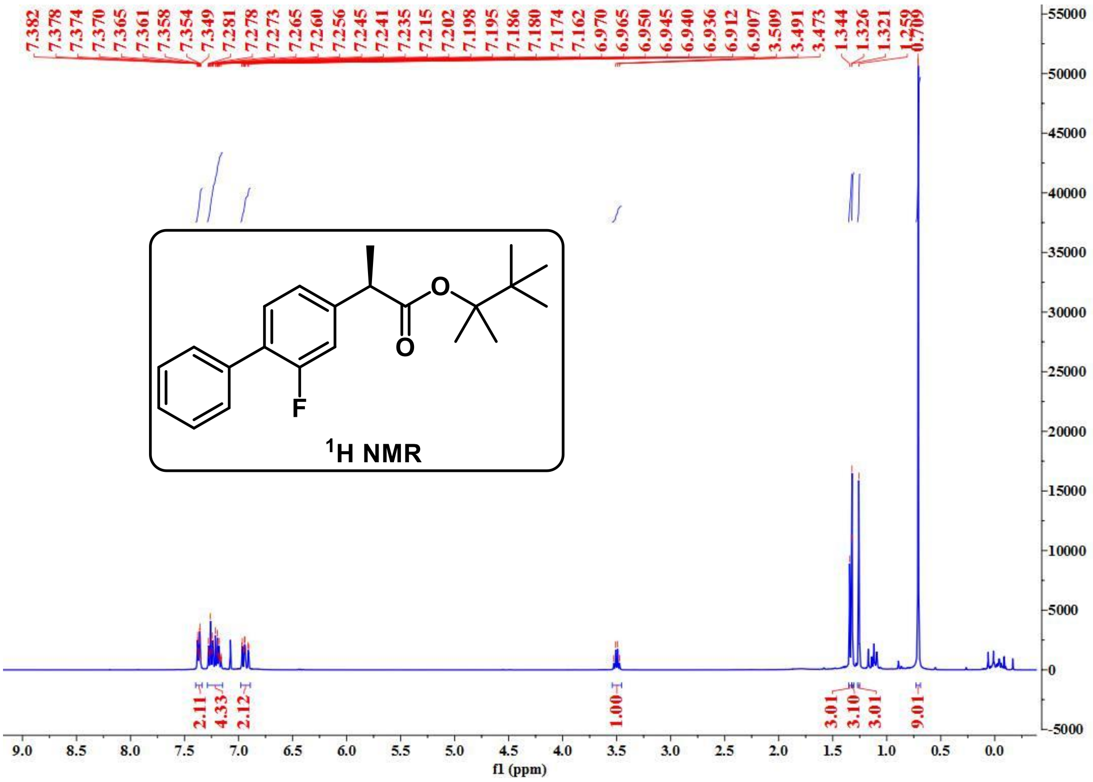

Supplementary Figure 104. ^{19}**F NMR Spectrum of 2,3,3-trimethylbutan-2-yl (*R*)-2-(2-fluoro-[1,1'-biphenyl]-4-yl)propanoate (376 MHz, CDCl$_3$)**

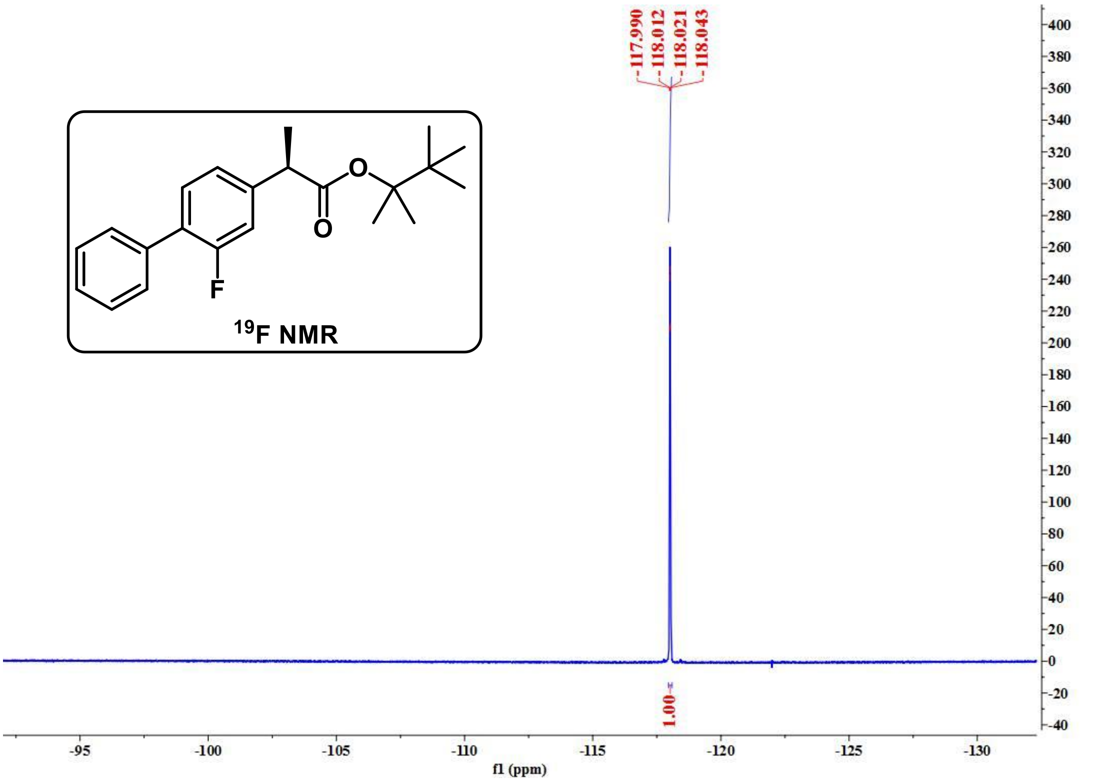

Supplementary Figure 105. ^{13}**C NMR Spectrum of 2,3,3-trimethylbutan-2-yl (*R*)-2-(2-fluoro-[1,1'-biphenyl]-4-yl)propanoate (101 MHz, CDCl**$_3$**)**

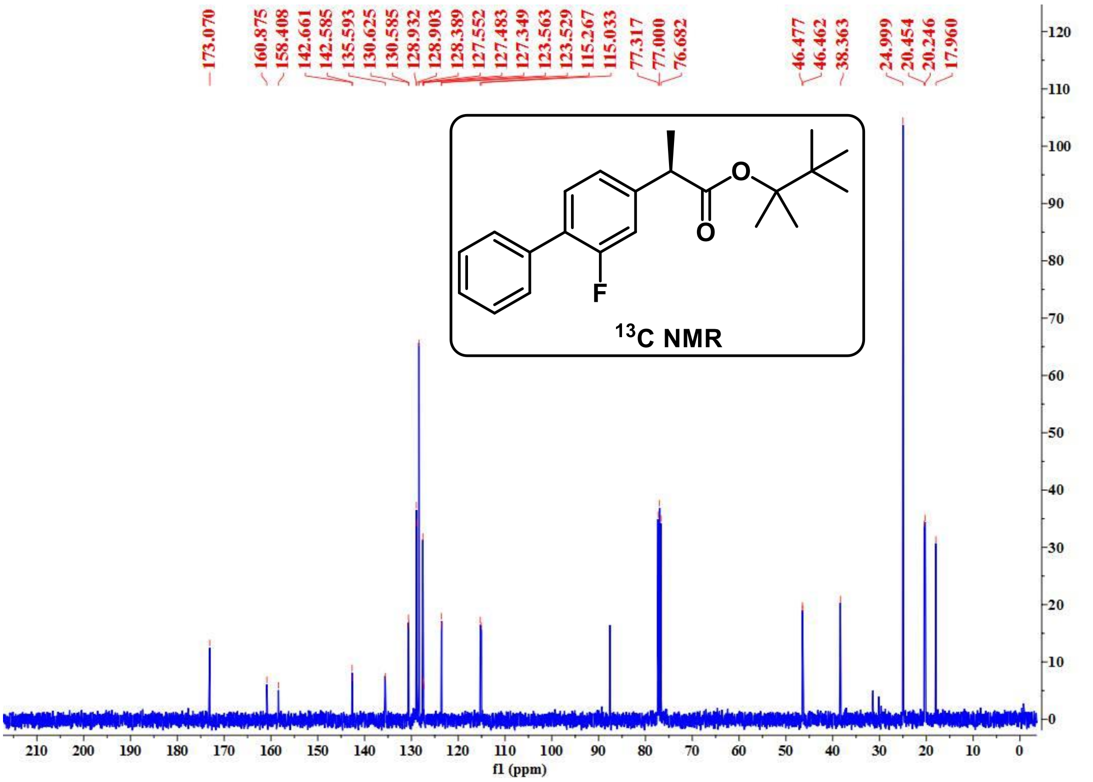

Supplementary Figure 106. ^{1}**H NMR Spectrum of 2,3,3-trimethylbutan-2-yl (*R*)-2-(3-benzoylphenyl)propanoate (400 MHz, CDCl**$_3$**)**

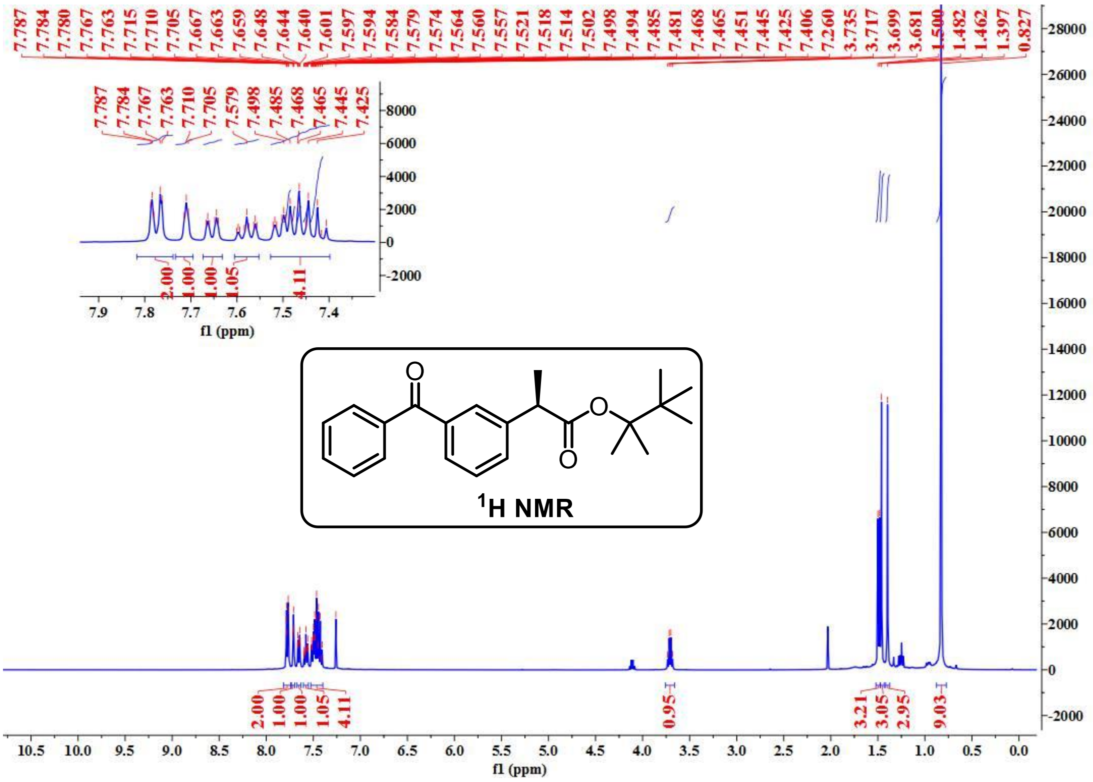

Supplementary Figure 107. ^{13}C NMR Spectrum of 2,3,3-trimethylbutan-2-yl (*R*)-2-(3-benzoylphenyl)propanoate (101 MHz, $CDCl_3$)

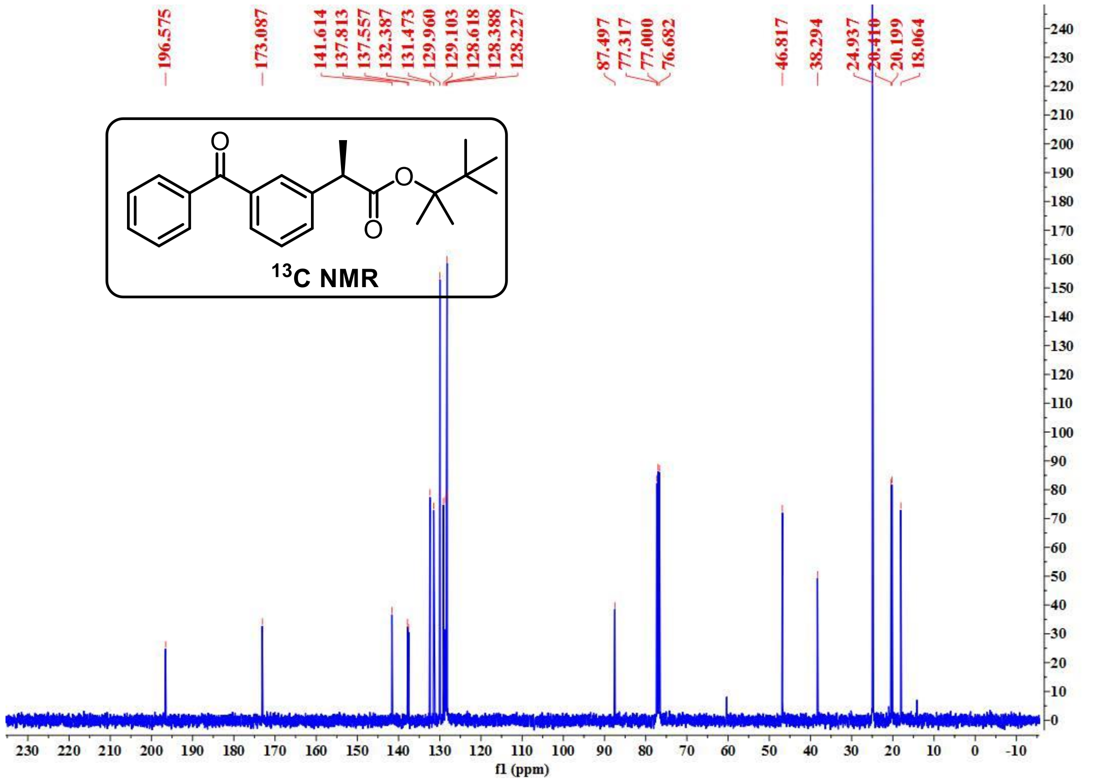

Supplementary Figure 108. ^{1}H NMR Spectrum of 2,3,3-trimethylbutan-2-yl (*R*)-2-(3-phenoxyphenyl)propanoate (400 MHz, $CDCl_3$)

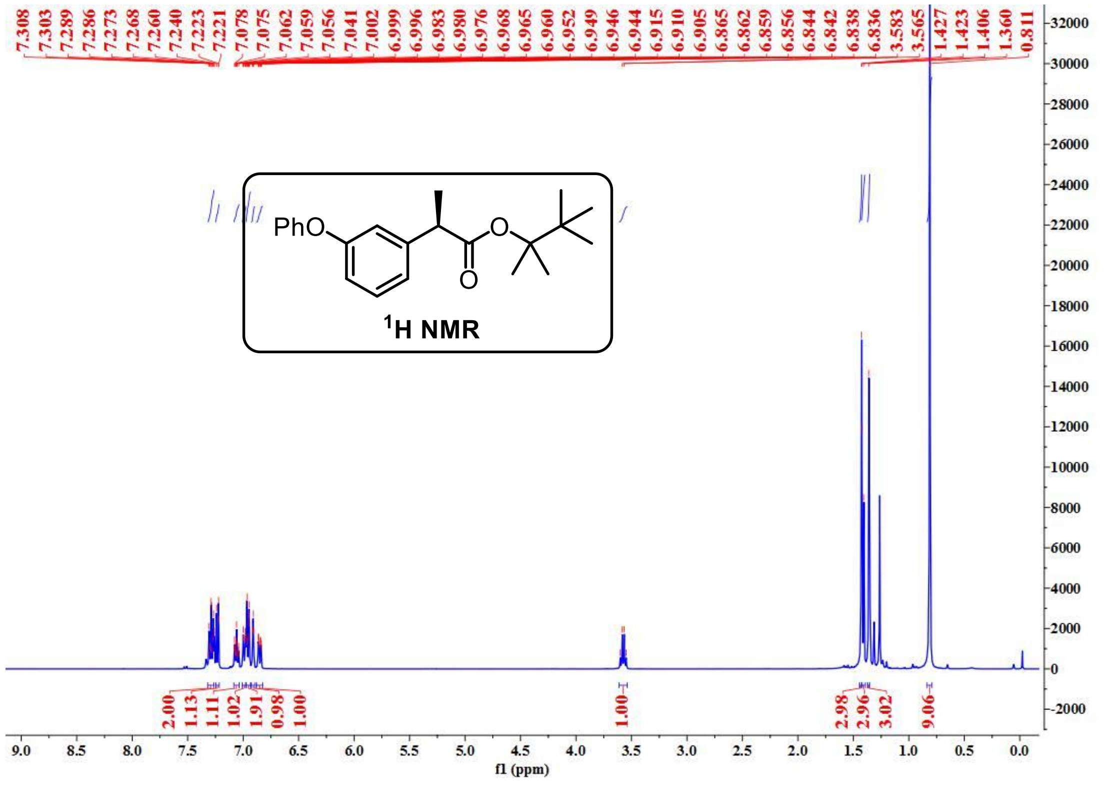

Supplementary Figure 109. ^{13}**C NMR Spectrum of 2,3,3-trimethylbutan-2-yl (*R*)-2-(3-phenoxyphenyl)propanoate (101 MHz, $CDCl_3$)**

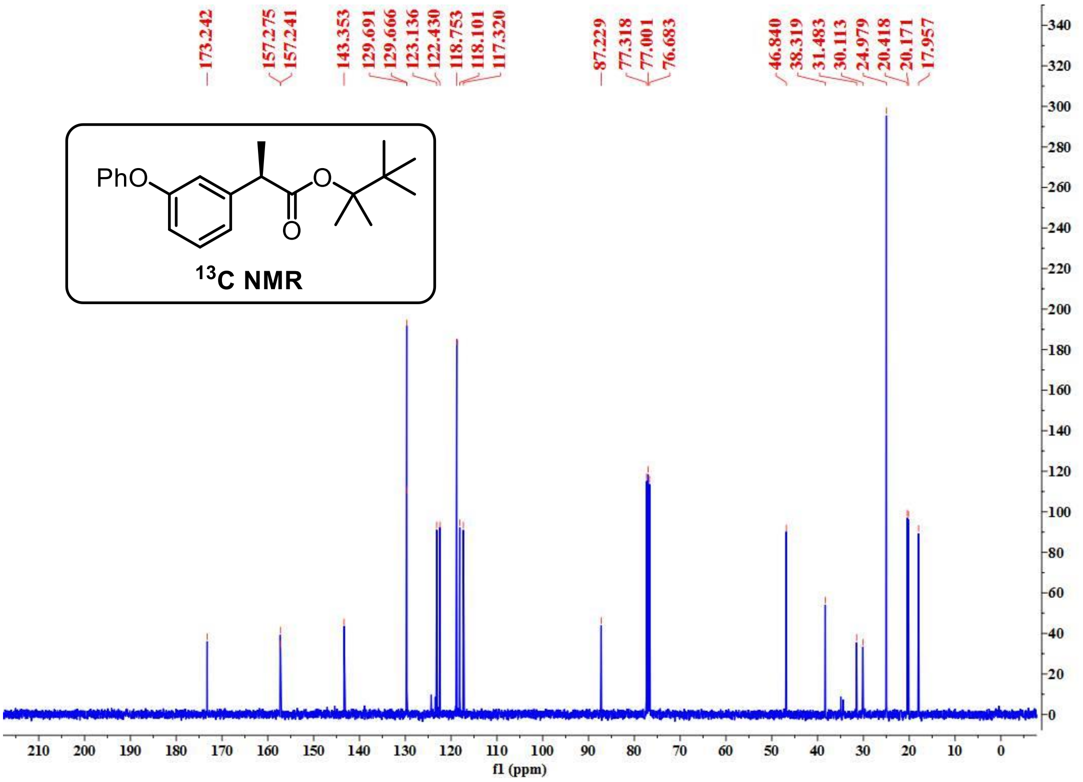

Supplementary Figure 110. ^{1}**H NMR Spectrum of 2,3,3-trimethylbutan-2-yl (*R*)-2-(4-phenoxyphenyl)propanoate (400 MHz, $CDCl_3$)**

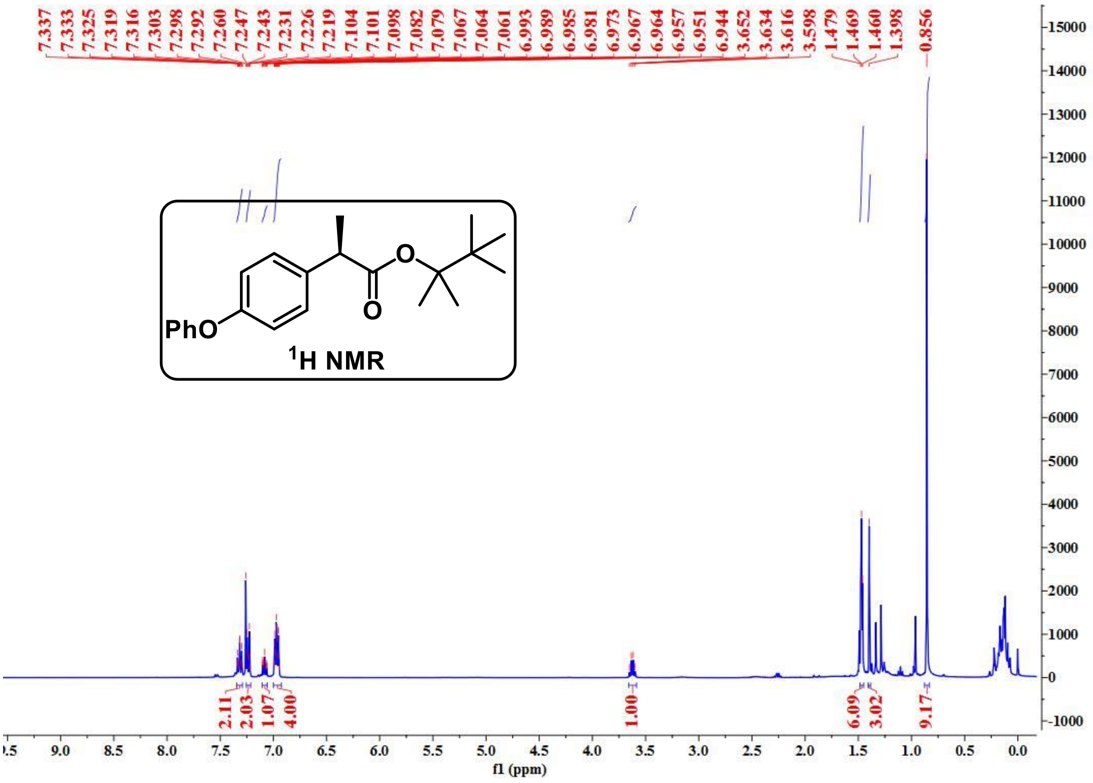

Supplementary Figure 111. ^{13}**C NMR Spectrum of 2,3,3-trimethylbutan-2-yl (*R*)-2-(4-phenoxyphenyl)propanoate (101 MHz, $CDCl_3$)**

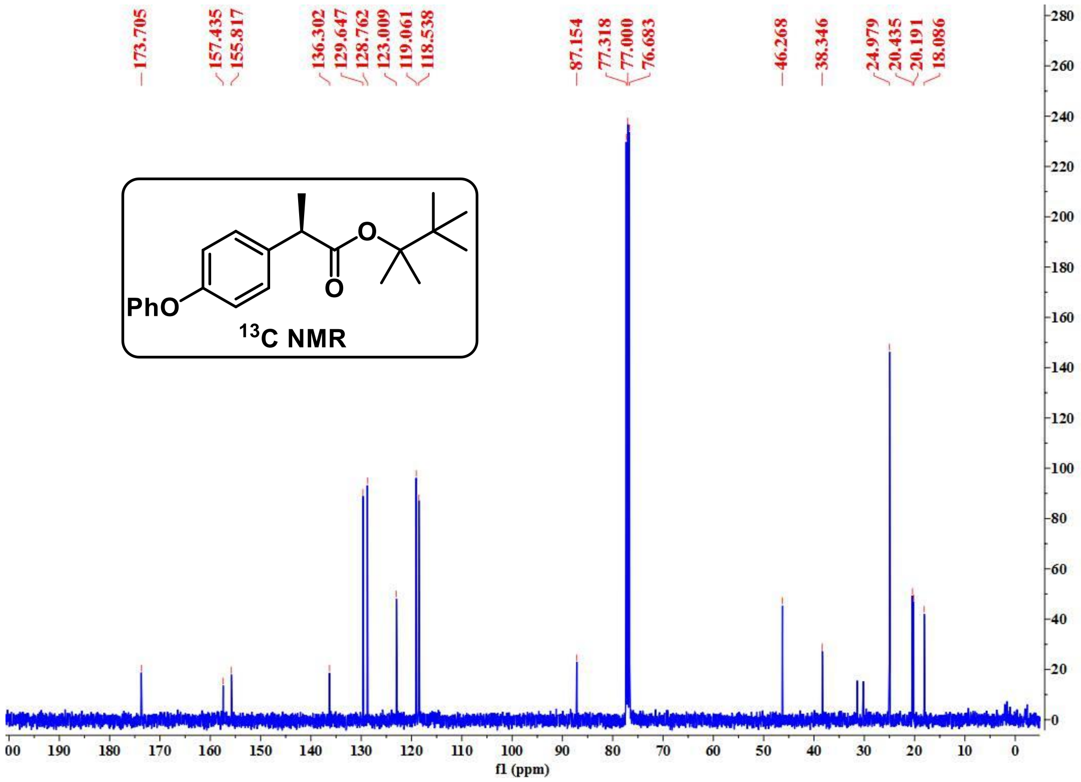

Supplementary Figure 112. ^{1}H NMR Spectrum of Methyl (*S*)-4-(3-(benzyloxy)-1-oxo-1-((2,3,3-trimethylbutan-2-yl)oxy)propan-2-yl)benzoate (400 MHz, $CDCl_3$)

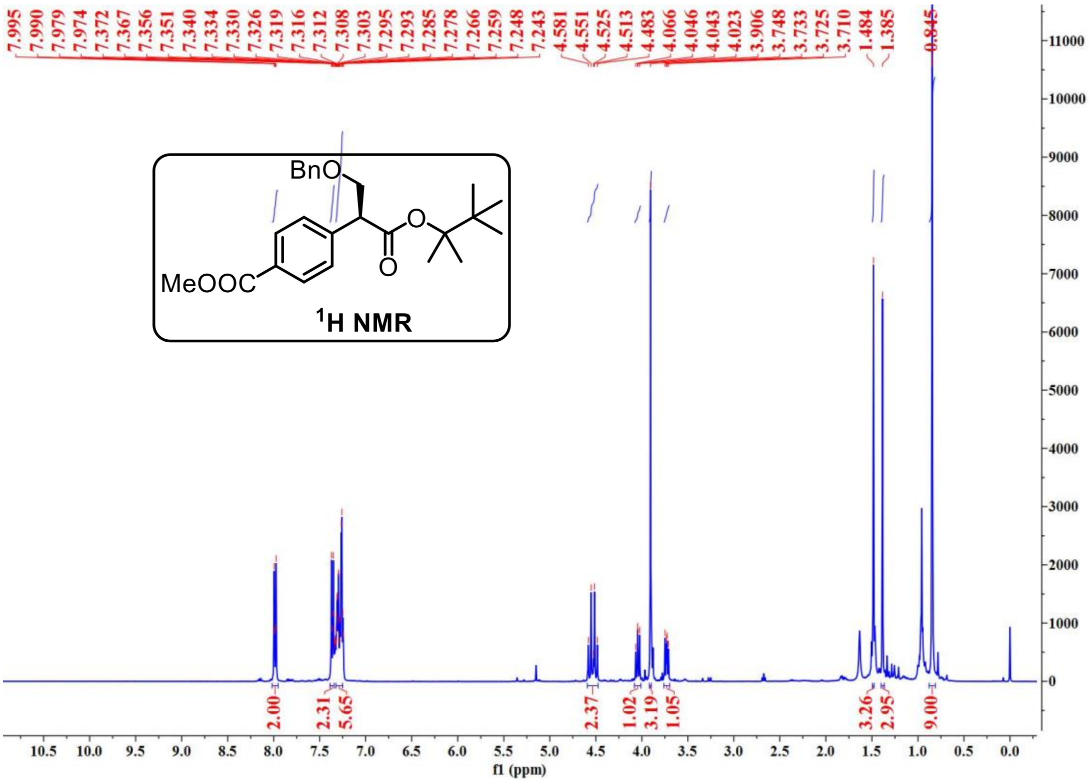

Supplementary Figure 113. ^{13}C NMR Spectrum of Methyl (*S*)-4-(3-(benzyloxy)-1-oxo-1-((2,3,3-trimethylbutan-2-yl)oxy)propan-2-yl)benzoate (101 MHz, $CDCl_3$)

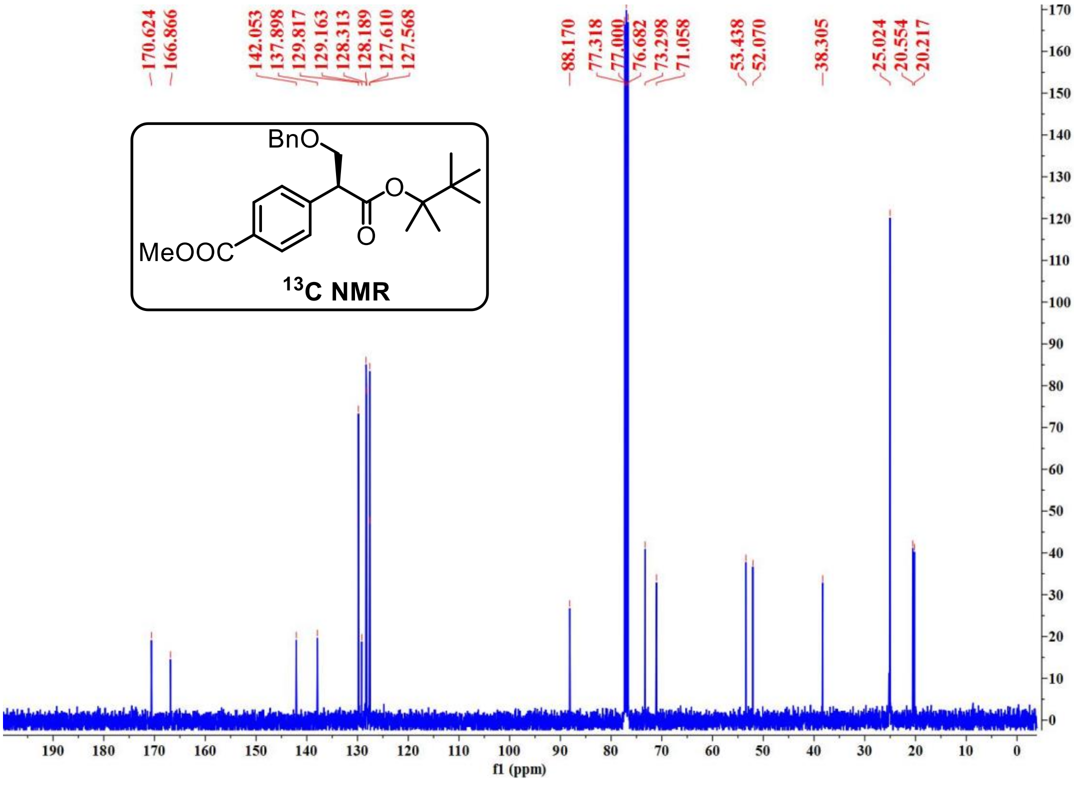

Supplementary Figure 114. ^{1}**H NMR Spectrum of Methyl (*R*)-4-(3-(4-methoxyphenyl)-1-oxo-1-((2,3,3-trimethylbutan-2-yl)oxy)propan-2-yl)benzoate (400 MHz, $CDCl_3$)**

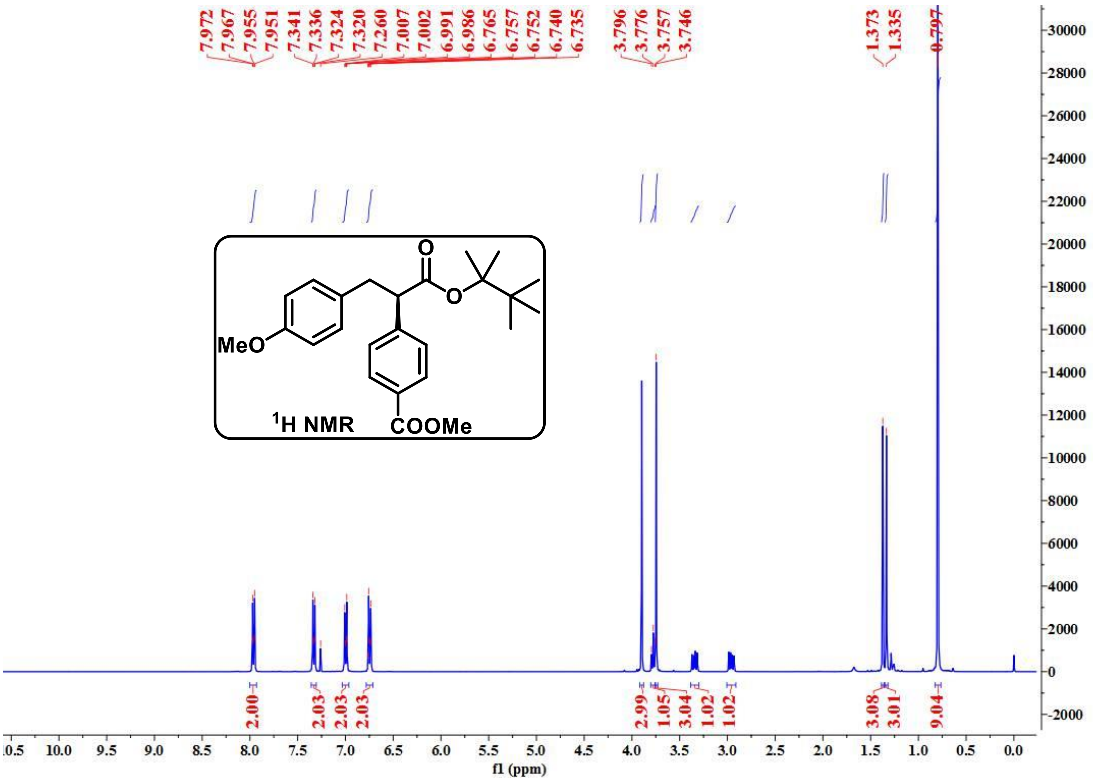

Supplementary Figure 115. ^{13}**C NMR Spectrum of Methyl (*R*)-4-(3-(4-methoxyphenyl)-1-oxo-1-((2,3,3-trimethylbutan-2-yl)oxy)propan-2-yl)benzoate (101 MHz, $CDCl_3$)**

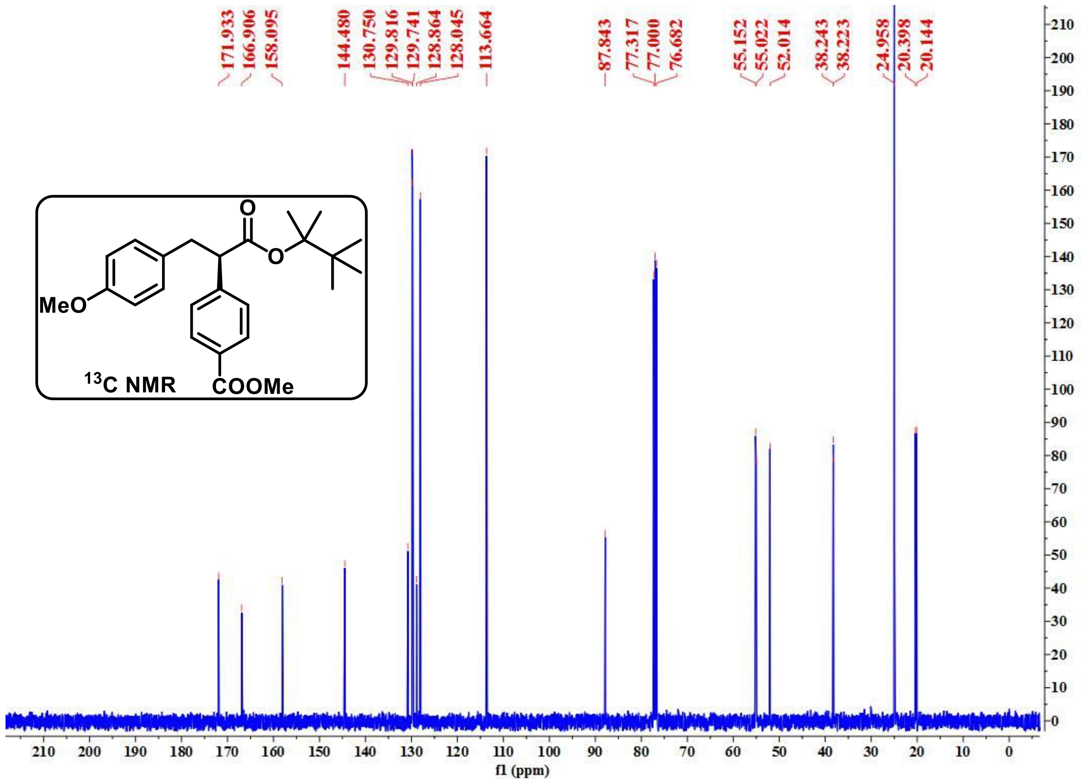

Supplementary Figure 116. ^{1}H NMR Spectrum of Methyl (*R*)-4-(1-oxo-3-phenyl-1-((2,3,3-trimethylbutan-2-yl)oxy)propan-2-yl)benzoate (400 MHz, $CDCl_3$)

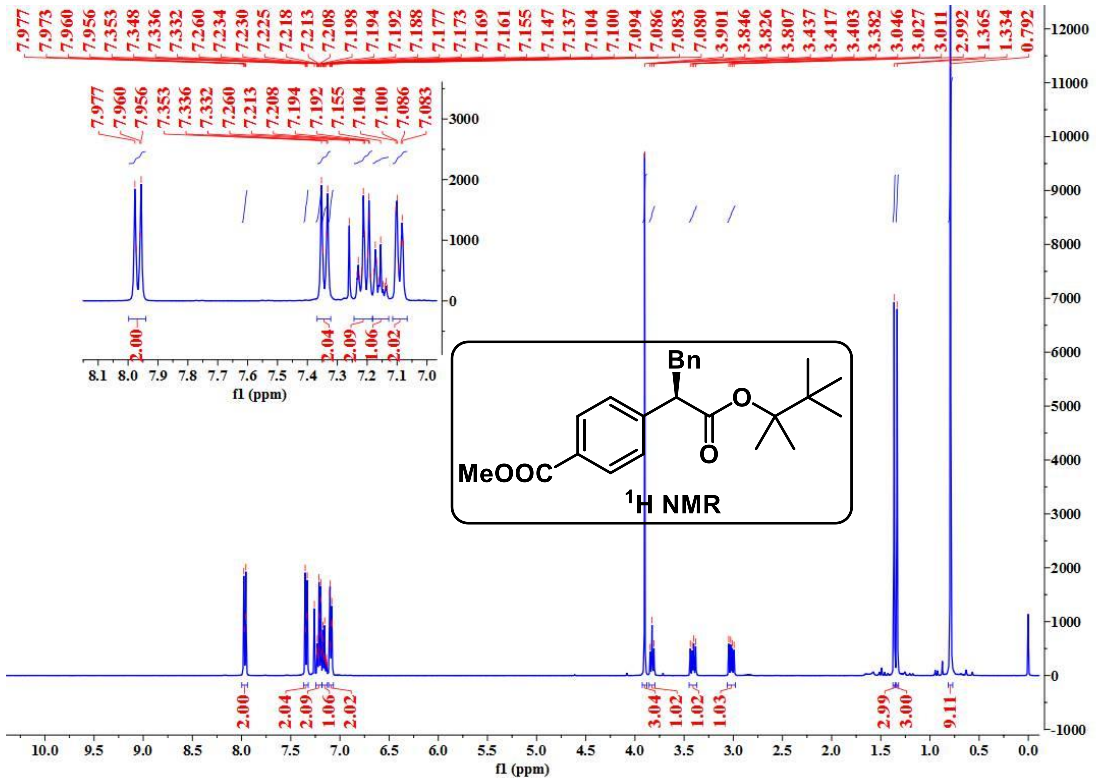

Supplementary Figure 117. ^{13}C NMR Spectrum of Methyl (*R*)-4-(1-oxo-3-phenyl-1-((2,3,3-trimethylbutan-2-yl)oxy)propan-2-yl)benzoate (101 MHz, $CDCl_3$)

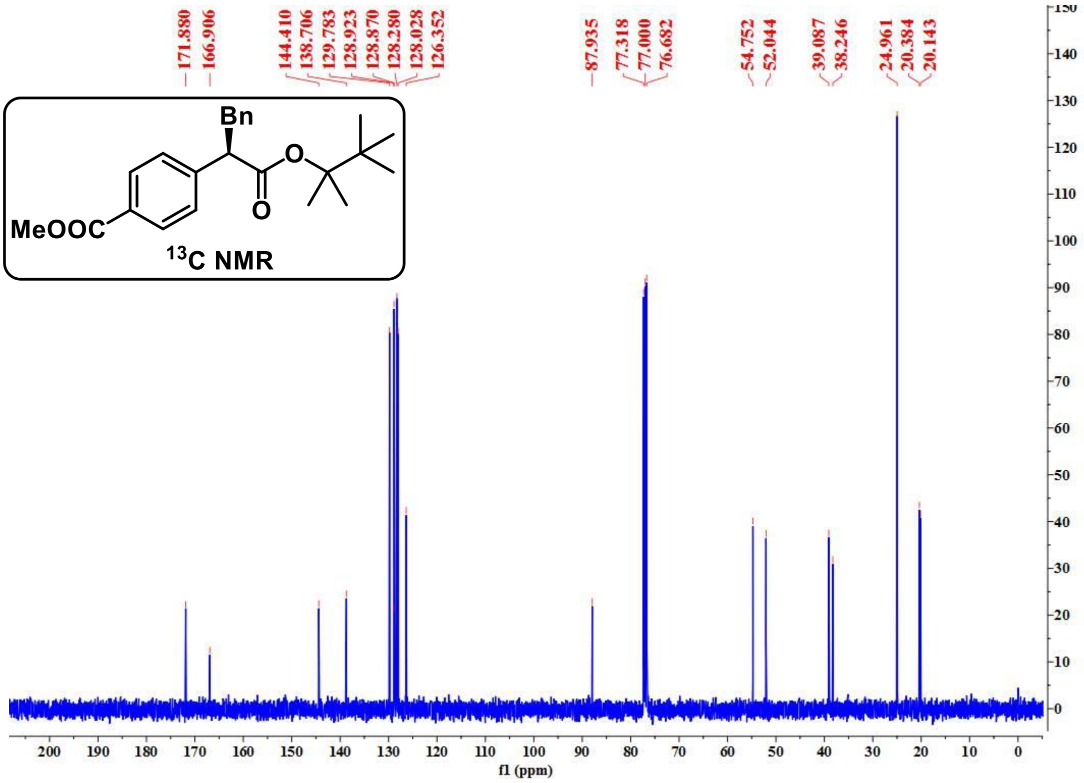

Supplementary Figure 118. **^{1}H NMR Spectrum of Methyl (*R*)-4-(1-oxo-4-phenyl-1-((2,3,3-trimethylbutan-2-yl)oxy)butan-2-yl)benzoate (400 MHz, $CDCl_3$)**

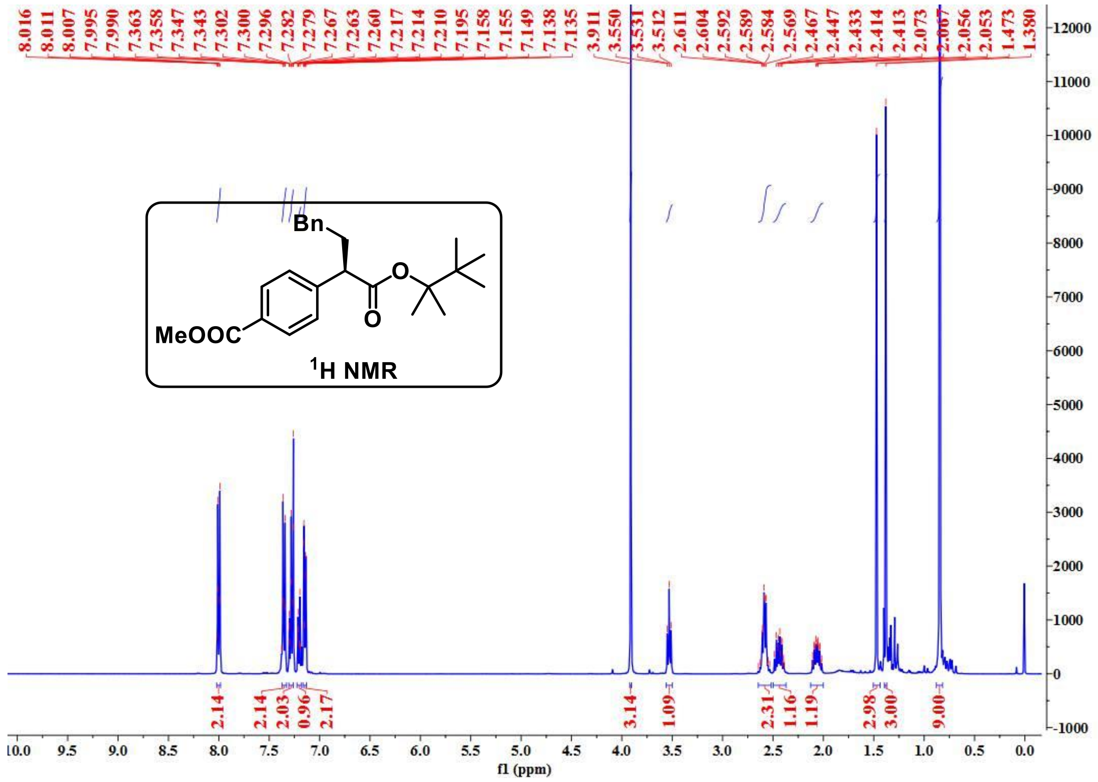

Supplementary Figure 119. **^{13}C NMR Spectrum of Methyl (*R*)-4-(1-oxo-4-phenyl-1-((2,3,3-trimethylbutan-2-yl)oxy)butan-2-yl)benzoate (101 MHz, $CDCl_3$)**

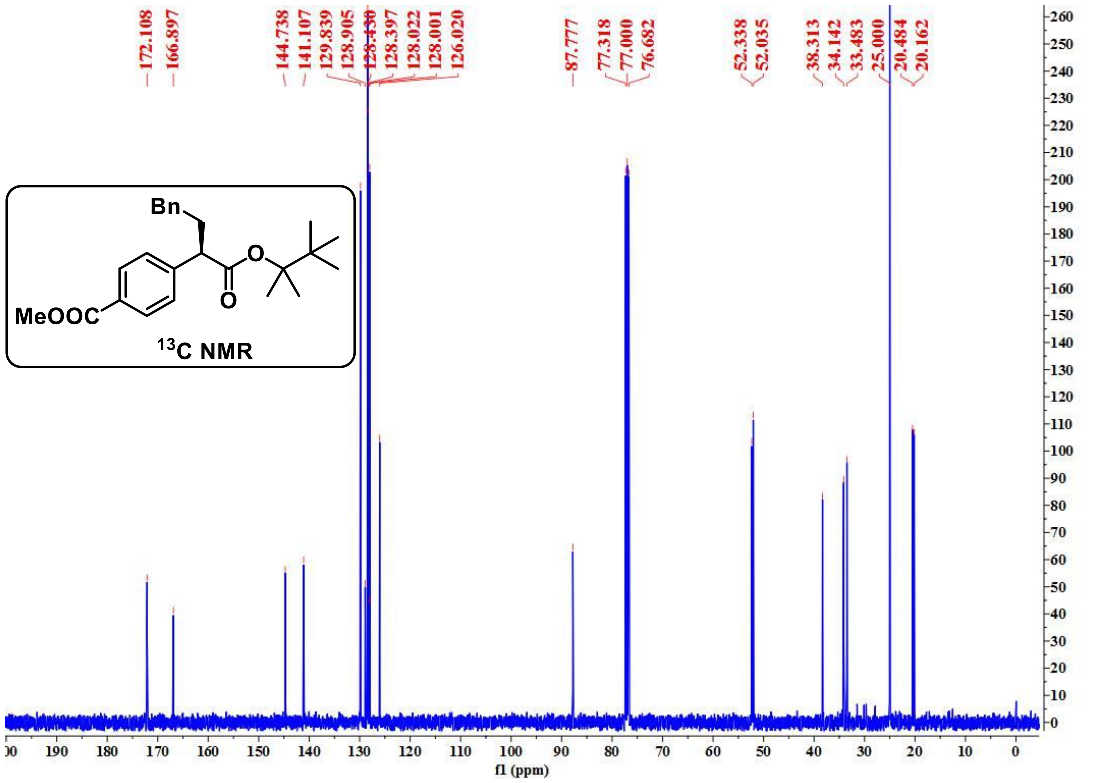

Supplementary Figure 120. ^{1}H NMR Spectrum of Methyl (*R*)-4-(4-(methylthio)-1-oxo-1-((2,3,3-trimethylbutan-2-yl)oxy)butan-2-yl)benzoate (400 MHz, $CDCl_3$)

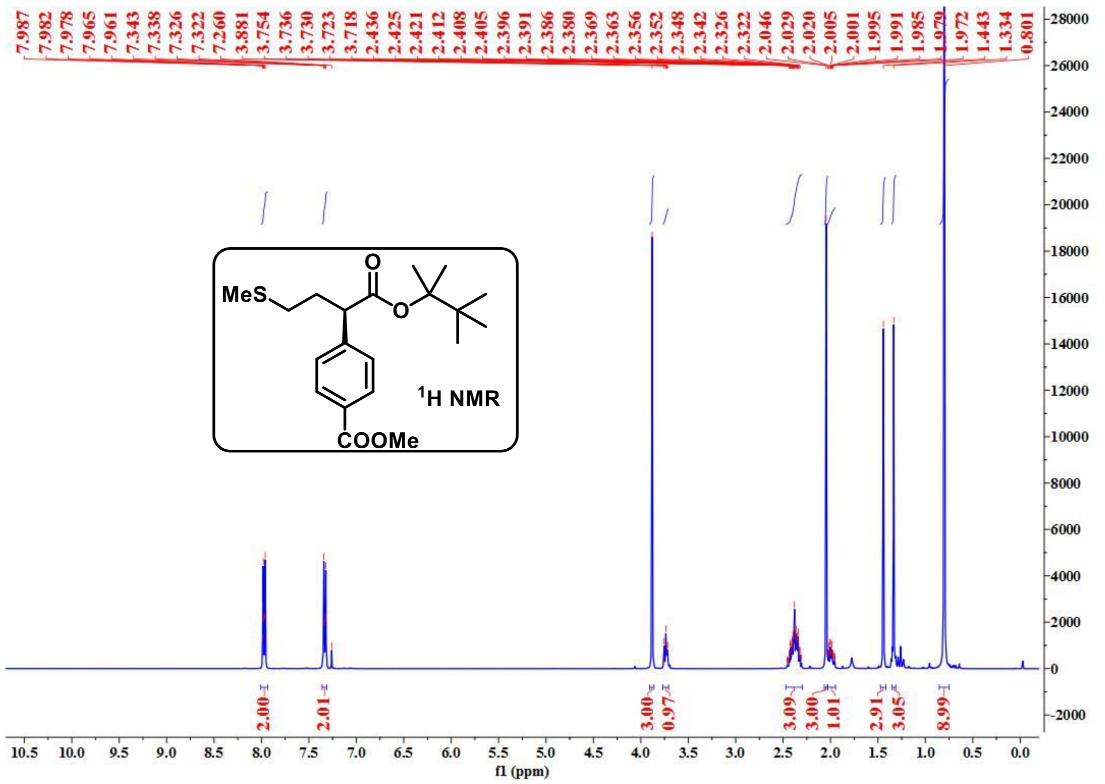

Supplementary Figure 121. ^{13}C NMR Spectrum of Methyl (*R*)-4-(4-(methylthio)-1-oxo-1-((2,3,3-trimethylbutan-2-yl)oxy)butan-2-yl)benzoate (101 MHz, $CDCl_3$)

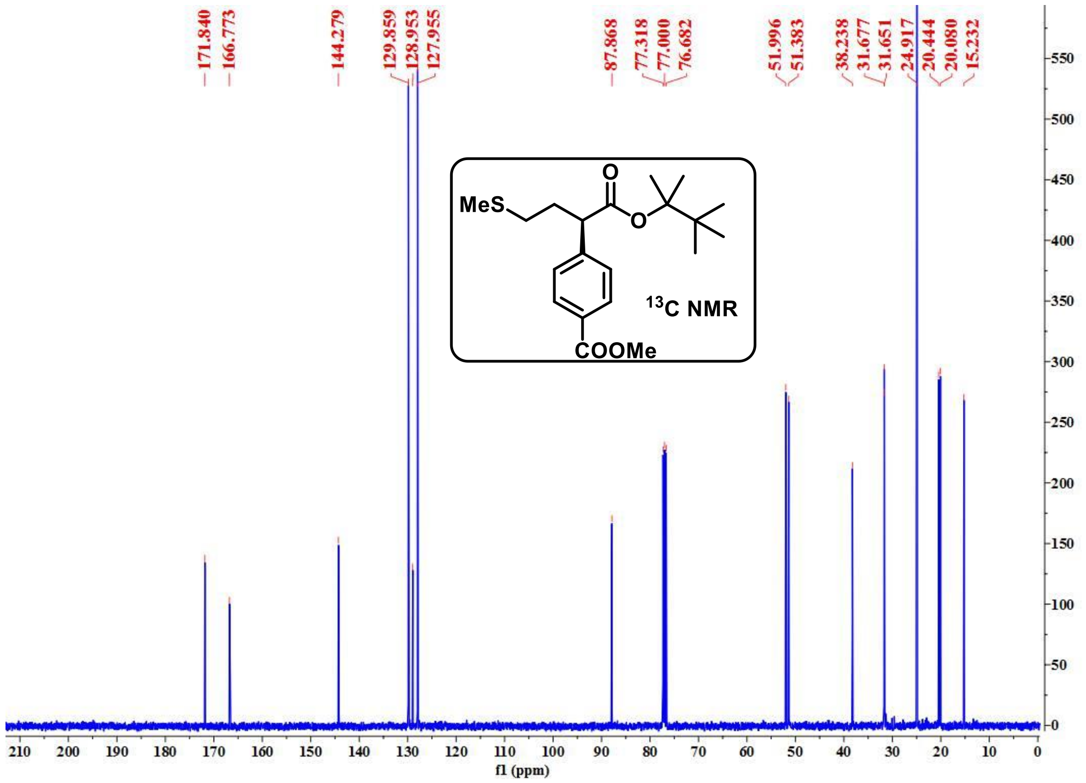

Supplementary Figure 122. ^{1}**H NMR Spectrum of Methyl (*R*)-4-(1-oxo-1-((2,3,3-trimethylbutan-2-yl)oxy)hept-6-en-2-yl)benzoate (400 MHz, CDCl$_3$)**

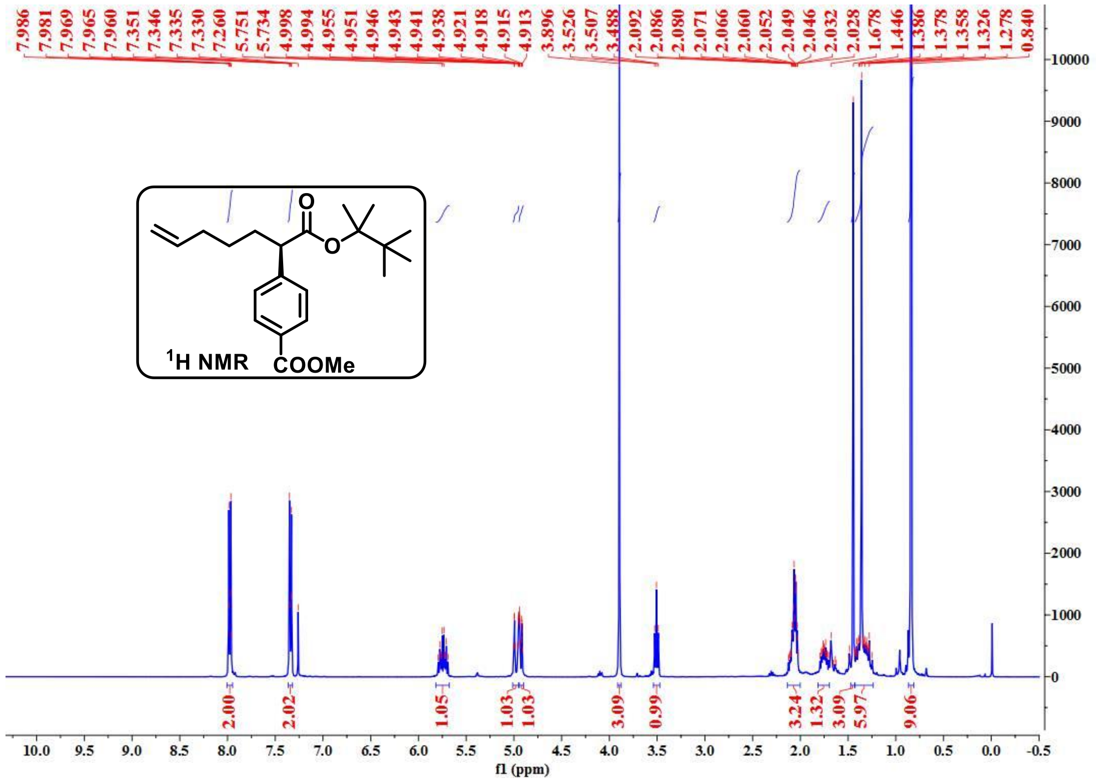

Supplementary Figure 123. ^{13}**C NMR Spectrum of Methyl (*R*)-4-(1-oxo-1-((2,3,3-trimethylbutan-2-yl)oxy)hept-6-en-2-yl)benzoate (101 MHz, CDCl$_3$)**

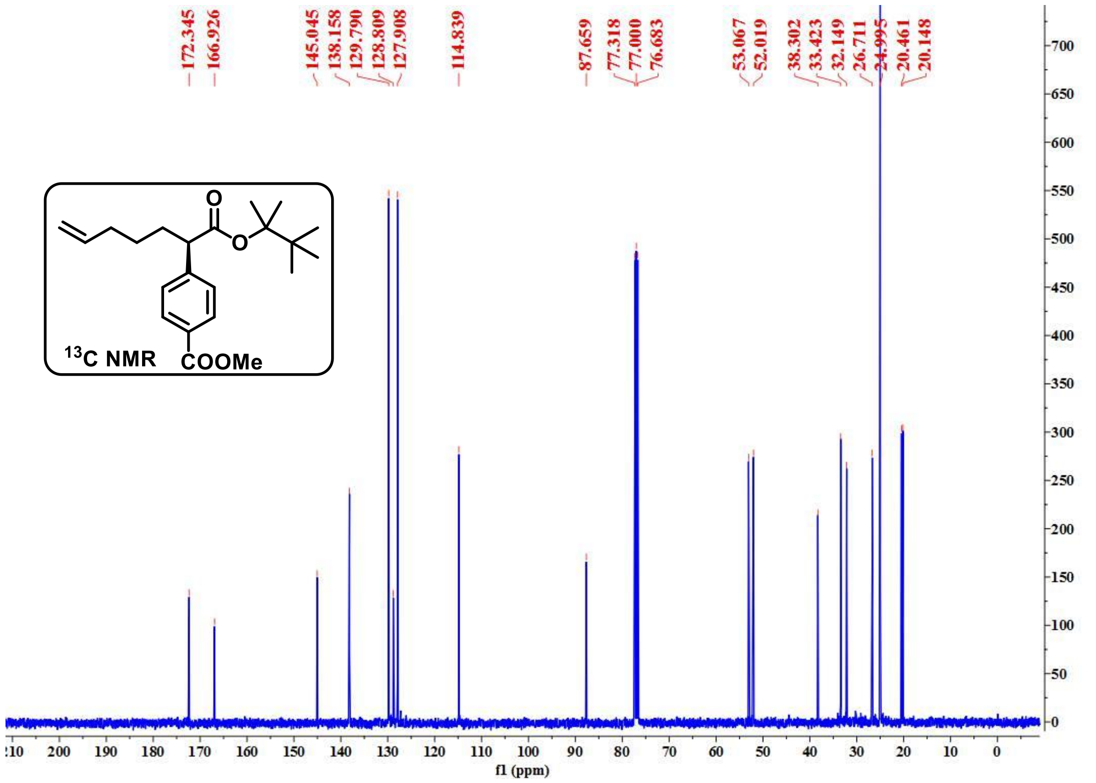

Supplementary Figure 124. ¹H NMR Spectrum of Methyl (*R*)-4-(1-oxo-1-((2,3,3-trimethylbutan-2-yl)oxy)butan-2-yl)benzoate (400 MHz, CDCl₃)

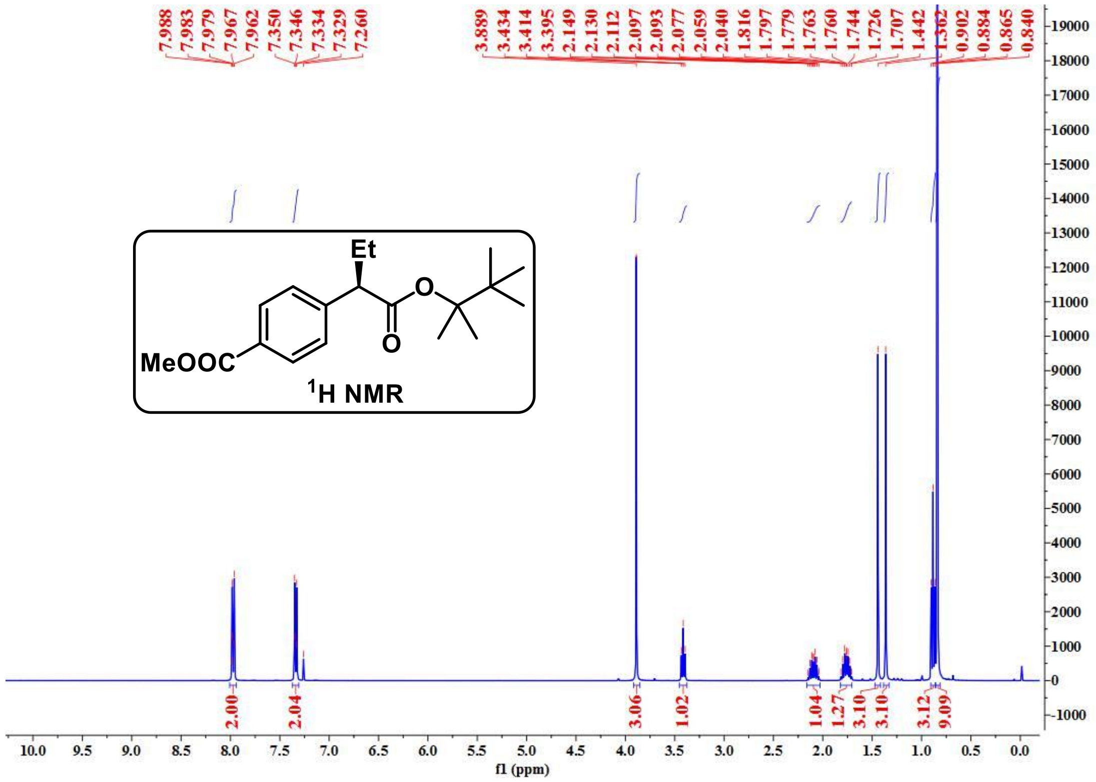

Supplementary Figure 125. ¹³C NMR Spectrum of Methyl (*R*)-4-(1-oxo-1-((2,3,3-trimethylbutan-2-yl)oxy)butan-2-yl)benzoate (101 MHz, CDCl₃)

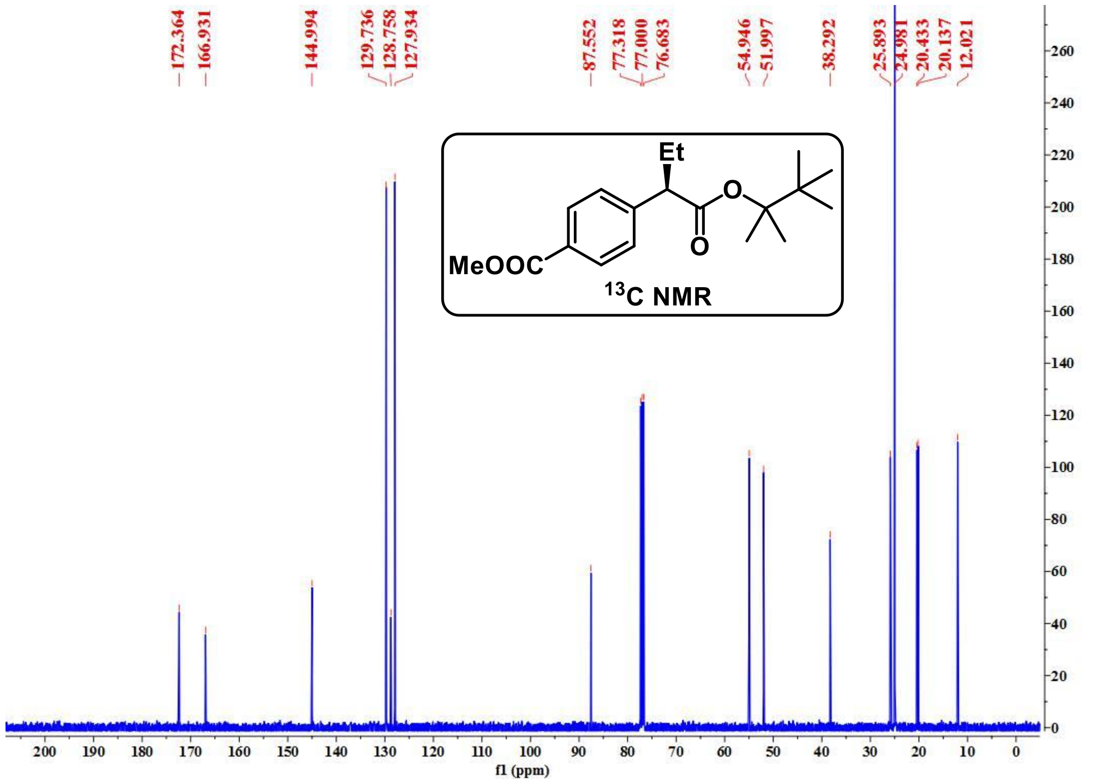

Supplementary Figure 126. ^{1}H NMR Spectrum of methyl (*R*)-4-(1-oxo-1-((2,3,3-trimethylbutan-2-yl)oxy)hexan-2-yl)benzoate (400 MHz, $CDCl_3$)

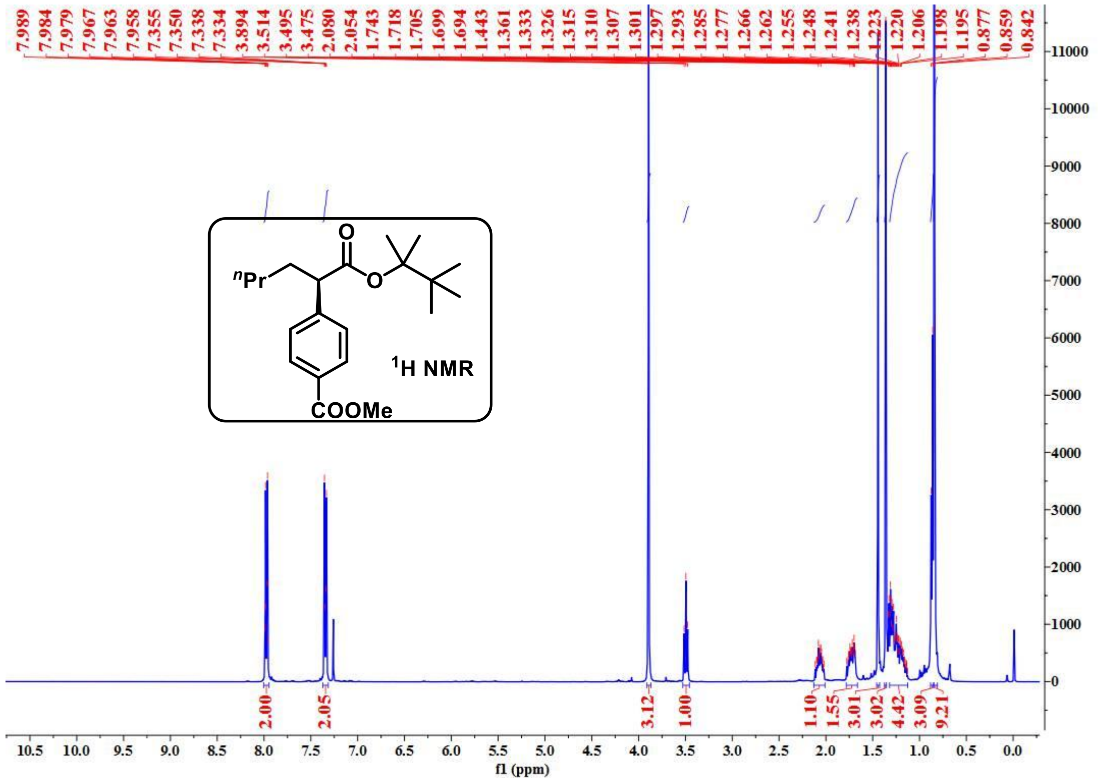

Supplementary Figure 127. ^{13}C NMR Spectrum of Methyl (*R*)-4-(1-oxo-1-((2,3,3-trimethylbutan-2-yl)oxy)hextan-2-yl)benzoate (101 MHz, $CDCl_3$)

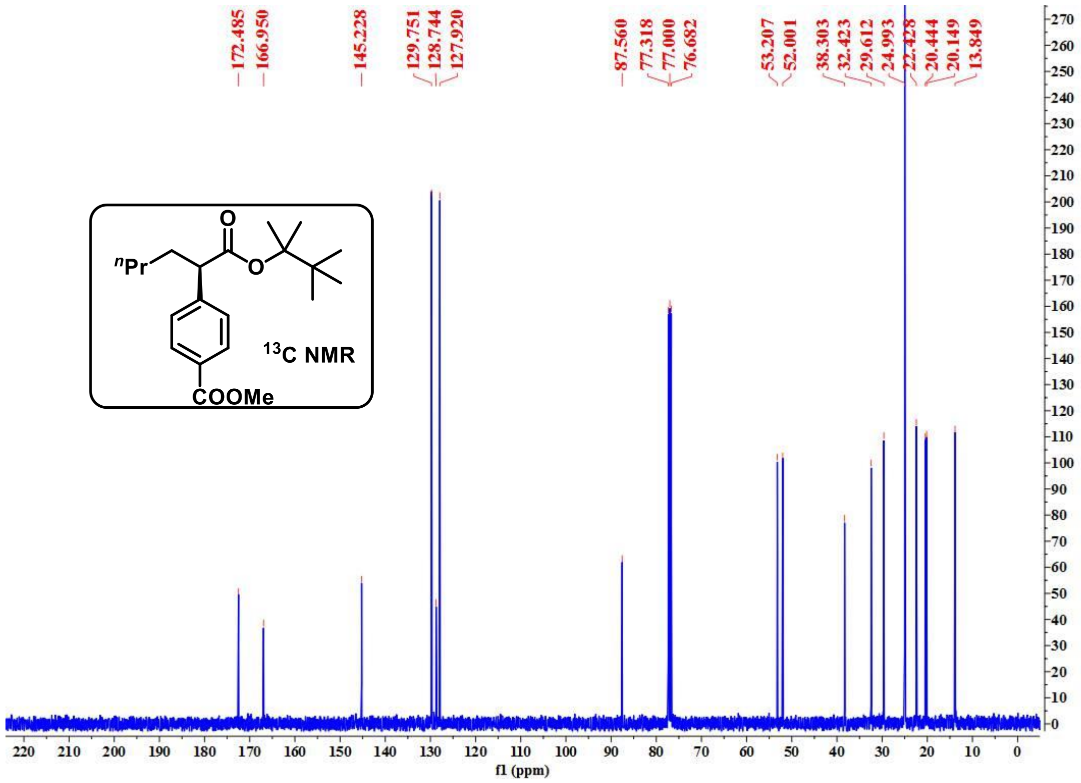

Supplementary Figure 128. ^{1}H NMR Spectrum of Methyl (*R*)-4-(4-methyl-1-oxo-1-((2,3,3-trimethylbutan-2-yl)oxy)pentan-2-yl)benzoate (400 MHz, $CDCl_3$)

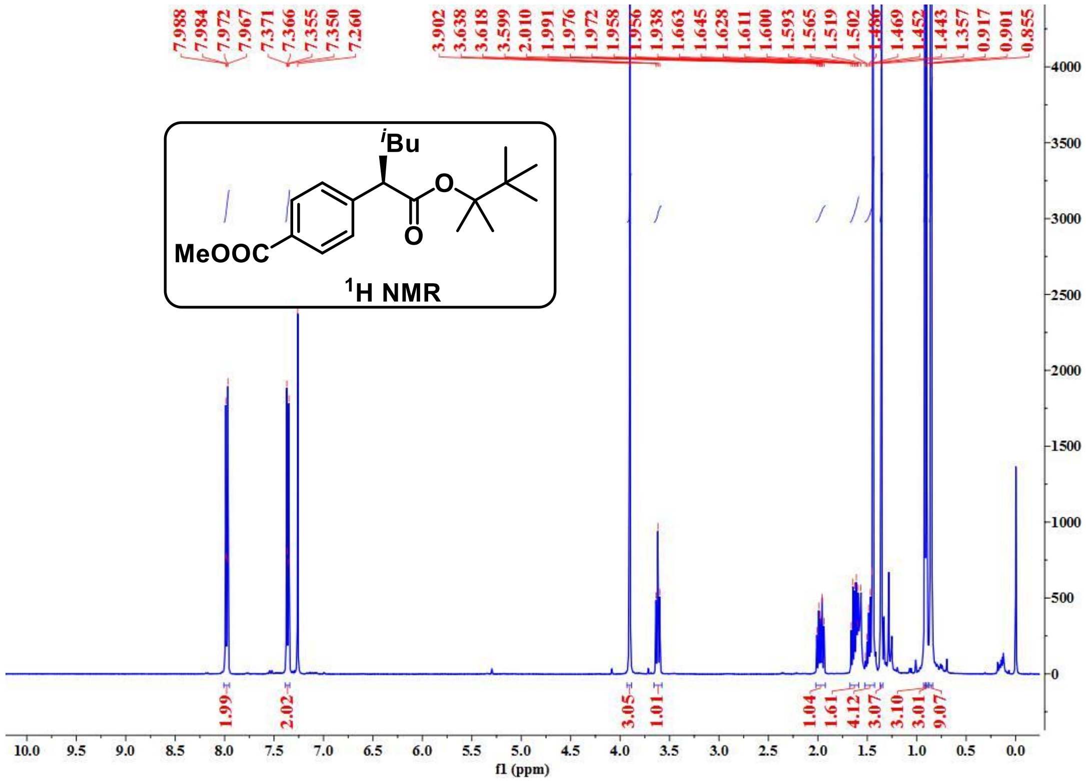

Supplementary Figure 129. ^{13}C NMR Spectrum of Methyl (*R*)-4-(4-methyl-1-oxo-1-((2,3,3-trimethylbutan-2-yl)oxy)pentan-2-yl)benzoate (101 MHz, $CDCl_3$)

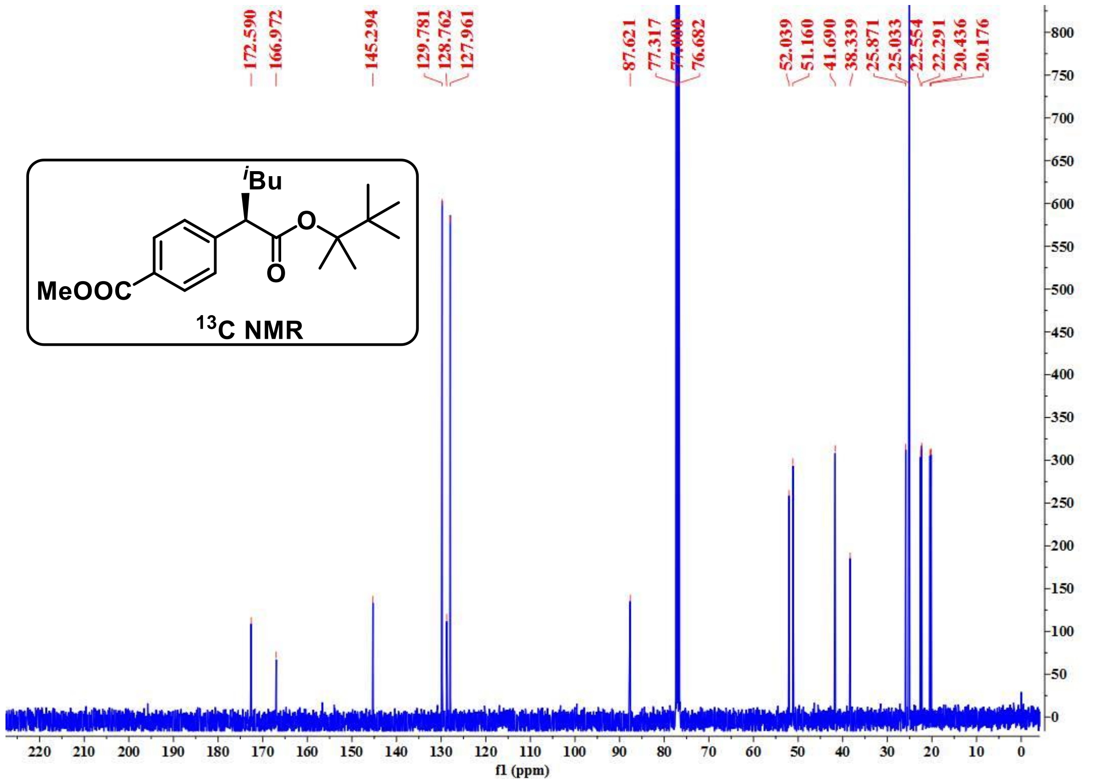

Supplementary Figure 130. ^{1}**H NMR Spectrum of Methyl (*R*)-4-(3-methyl-1-oxo-1-((2,3,3-trimethylbutan-2-yl)oxy)butan-2-yl)benzoate (400 MHz, CDCl$_3$)**

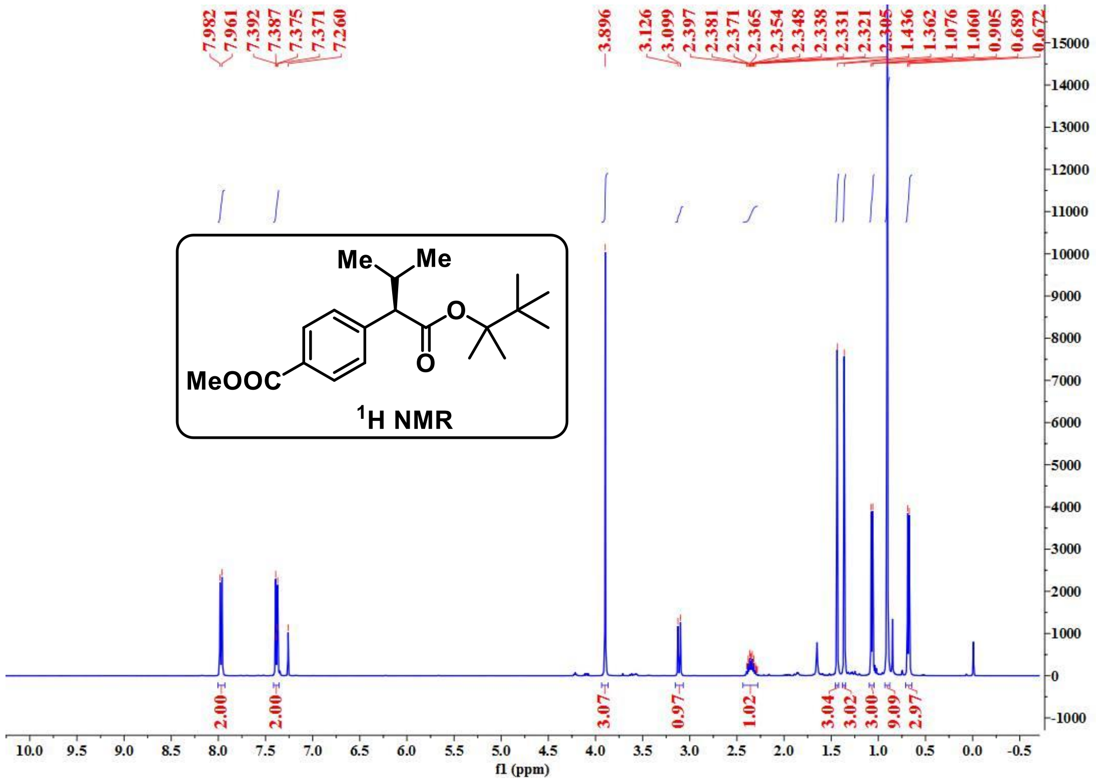

Supplementary Figure 131. ^{13}**C NMR Spectrum of Methyl (*R*)-4-(3-methyl-1-oxo-1-((2,3,3-trimethylbutan-2-yl)oxy)butan-2-yl)benzoate (101 MHz, CDCl$_3$)**

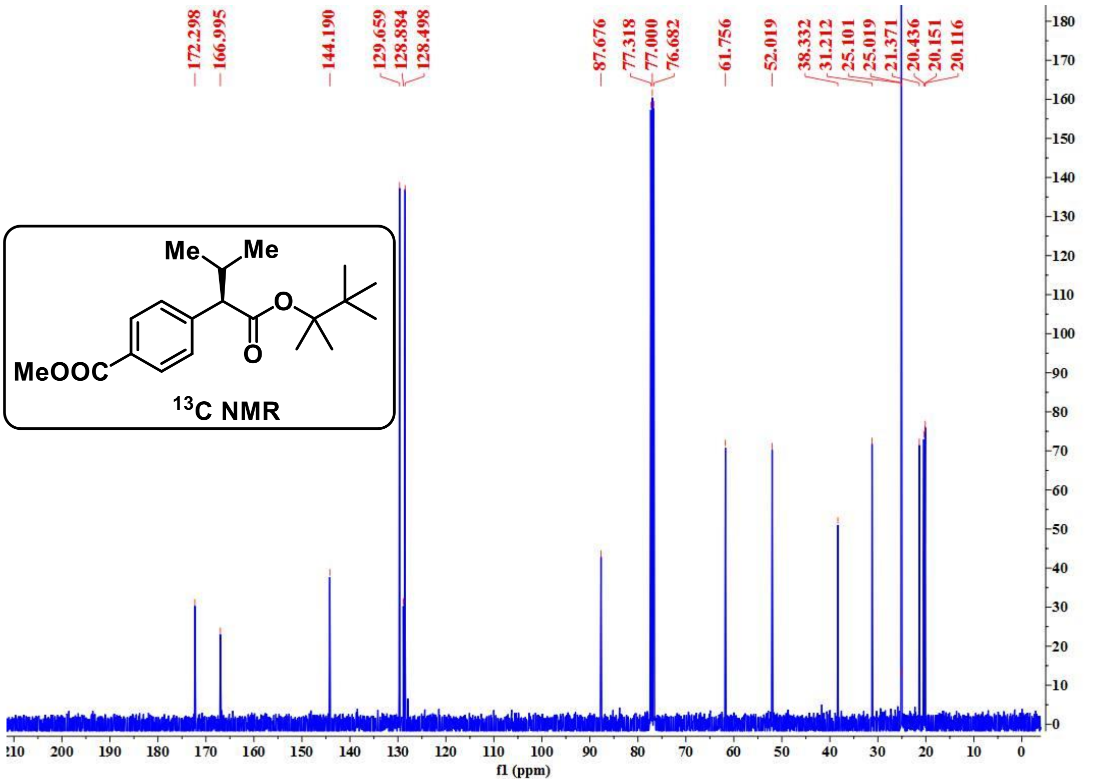

Supplementary Figure 132. ^{1}H NMR Spectrum of Methyl (*R*)-4-(1-cyclopentyl-2-oxo-2-((2,3,3-trimethylbutan-2-yl)oxy)ethyl)benzoate (400 MHz, $CDCl_3$)

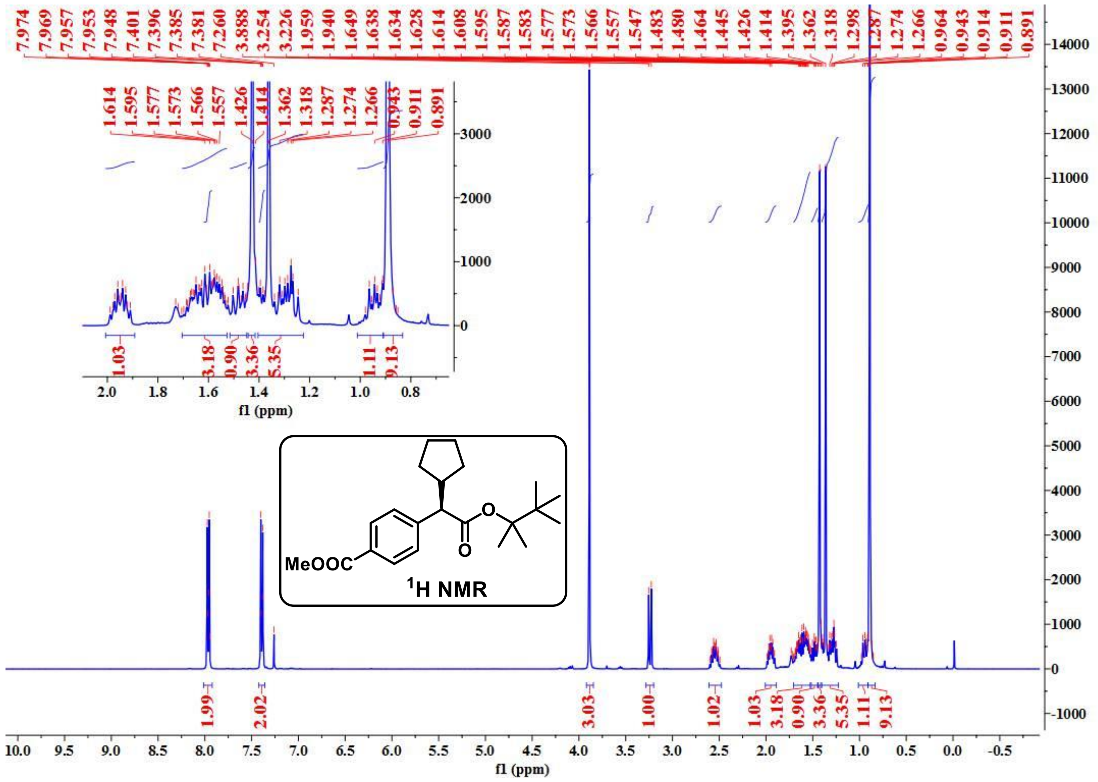

Supplementary Figure 133. ^{13}C NMR Spectrum of Methyl (*R*)-4-(1-cyclopentyl-2-oxo-2-((2,3,3-trimethylbutan-2-yl)oxy)ethyl)benzoate (101 MHz, $CDCl_3$)

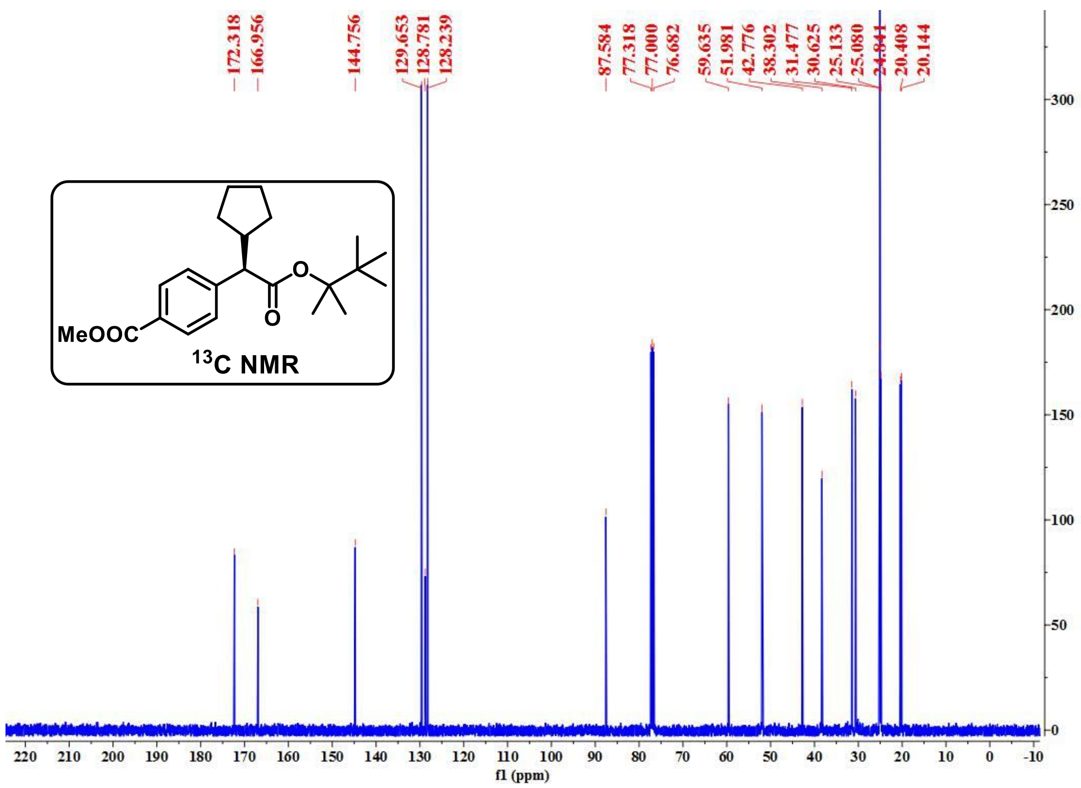

Supplementary Figure 134. ^{1}H NMR Spectrum of Methyl (*R*)-4-(1-cyclohexyl-2-oxo-2-((2,3,3-trimethylbutan-2-yl)oxy)ethyl)benzoate (400 MHz, $CDCl_3$)

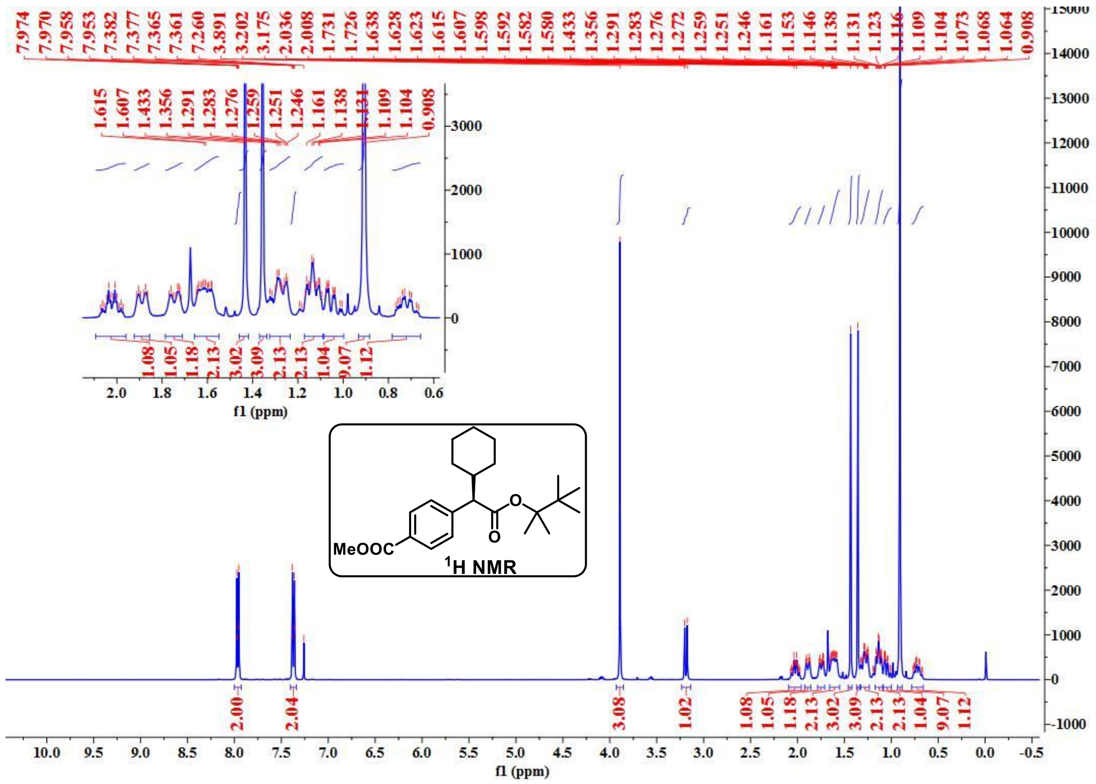

Supplementary Figure 135. ^{13}C NMR Spectrum of Methyl (*R*)-4-(1-cyclohexyl-2-oxo-2-((2,3,3-trimethylbutan-2-yl)oxy)ethyl)benzoate (101 MHz, $CDCl_3$)

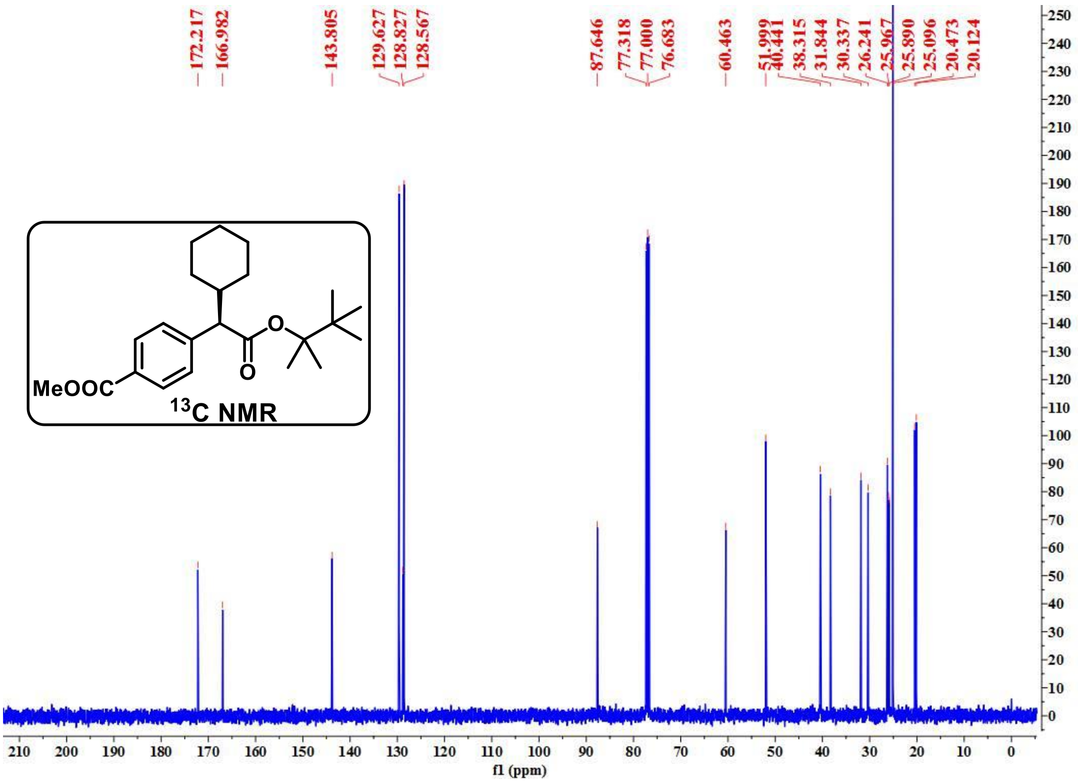
172.217
166.982
143.805
129.627
128.827
128.567
87.646
77.318
77.000
76.683
60.463
51.999
40.441
38.315
31.844
30.337
26.241
25.967
25.890
25.096
20.473
20.124
MeOOC
O
O
^{13}C NMR
f1 (ppm)

Supplementary Figure 136. ^{1}H NMR Spectrum of Methyl (*E*)-4-(5-ethoxy-5-oxopent-3-en-1-yl)benzoate (400 MHz, $CDCl_3$)

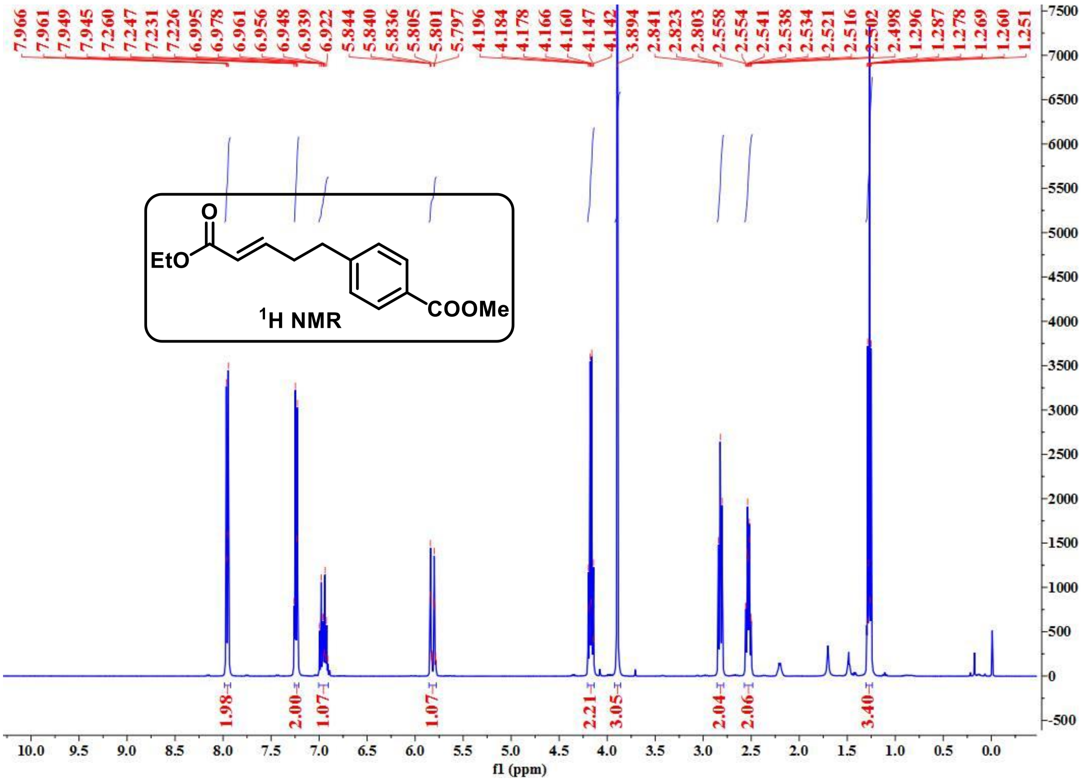

Supplementary Figure 137. ^{13}C NMR Spectrum of Methyl (*E*)-4-(5-ethoxy-5-oxopent-3-en-1-yl)benzoate (101 MHz, $CDCl_3$)

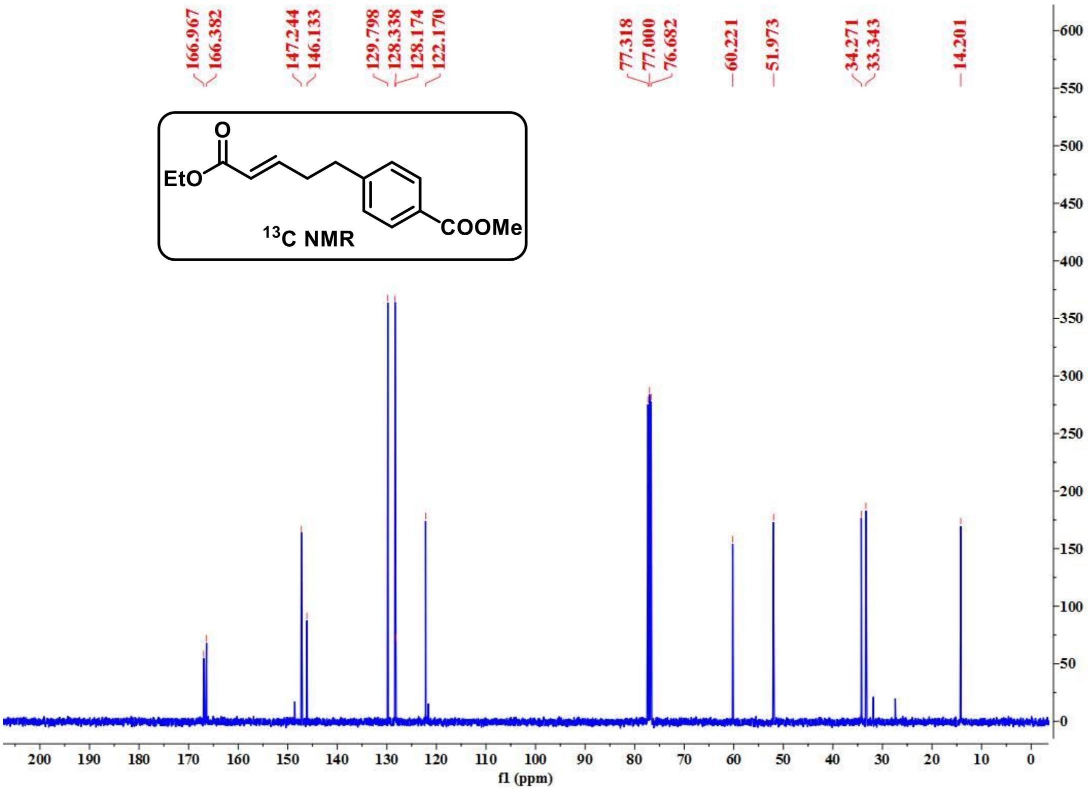

Supplementary Figure 138. ^{1}**H NMR Spectrum of (*R*)-2-(2-fluoro-[1,1'-biphenyl]-4-yl)propanoic acid (400 MHz, CDCl$_3$)**

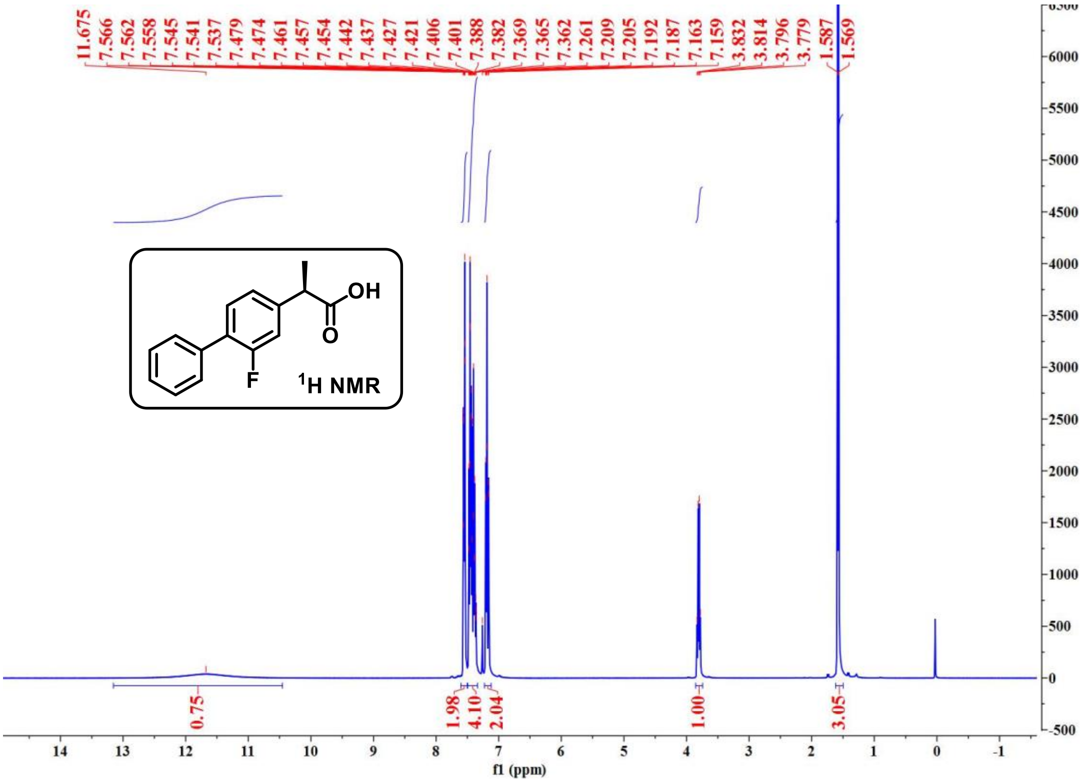

Supplementary Figure 139. ^{19}**F NMR Spectrum of (*R*)-2-(2-fluoro-[1,1'-biphenyl]-4-yl)propanoic acid (377 MHz, CDCl$_3$)**

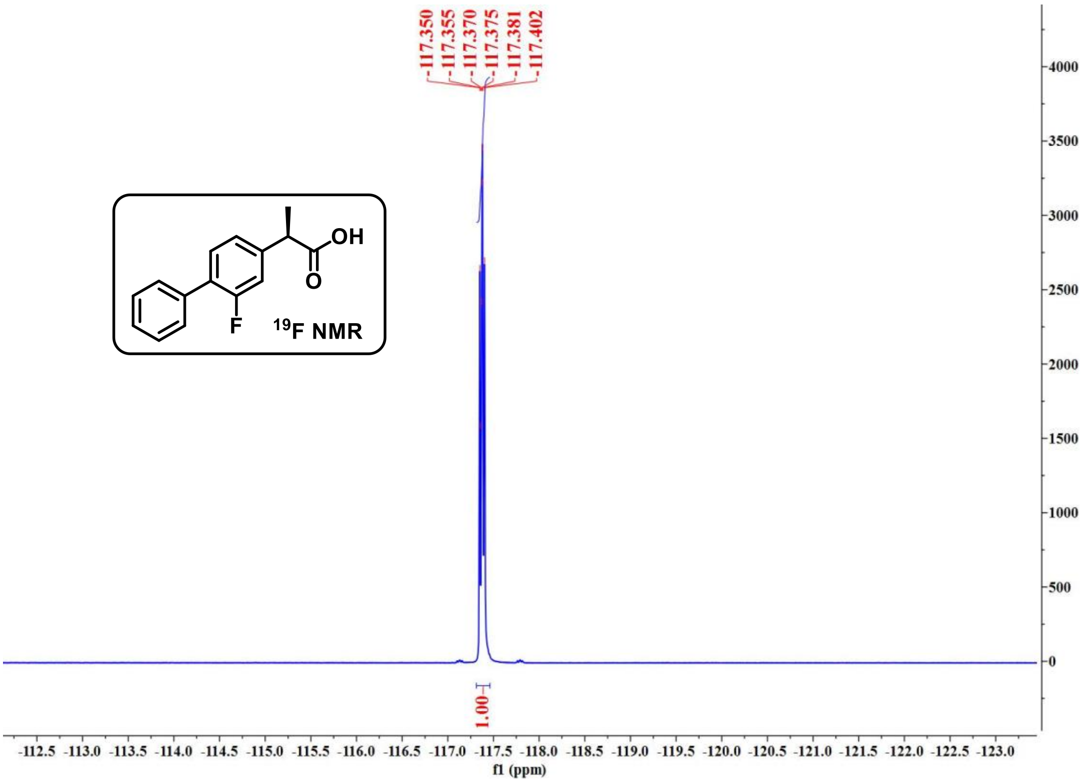

Supplementary Figure 140. ^{13}C NMR Spectrum of (*R*)-2-(2-fluoro-[1,1'-biphenyl]-4-yl)propanoic acid (101 MHz, CDCl$_3$)

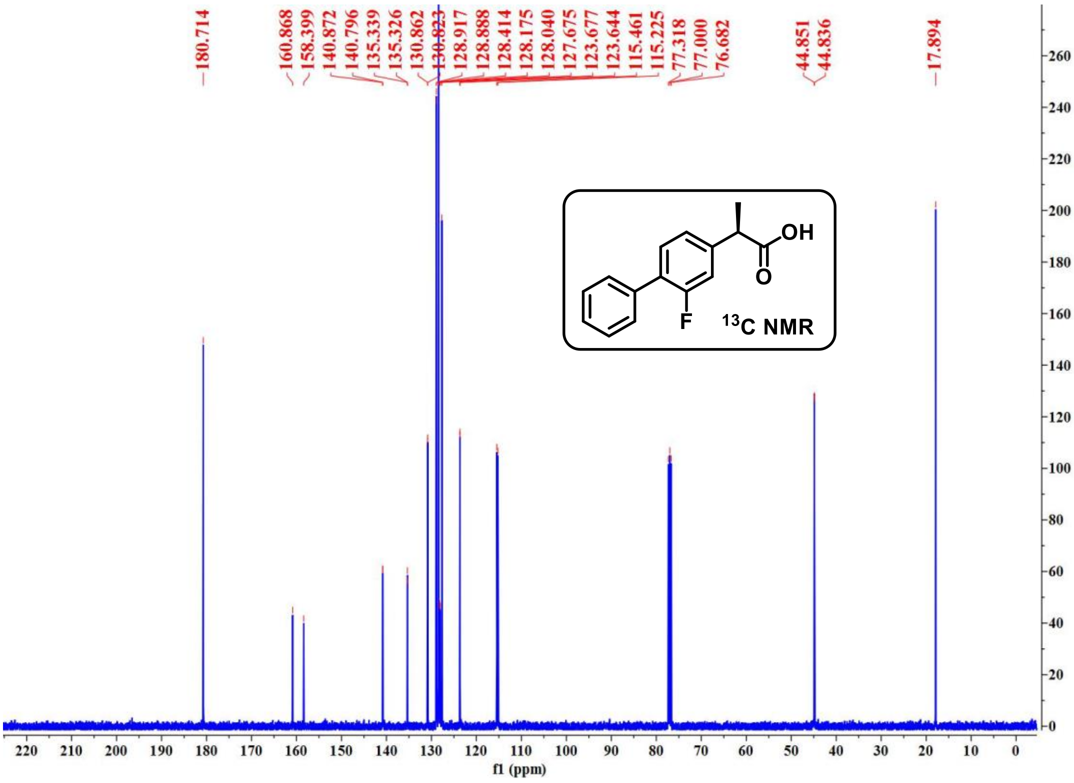

Supplementary Figure 141. ^{1}**H NMR Spectrum of Methyl 4-(((1*R*,2*S*)-2-(((2,3,3-trimethylbutan-2-yl)oxy)carbonyl)cyclopentyl)methyl)benzoate (600 MHz, CDCl₃)**

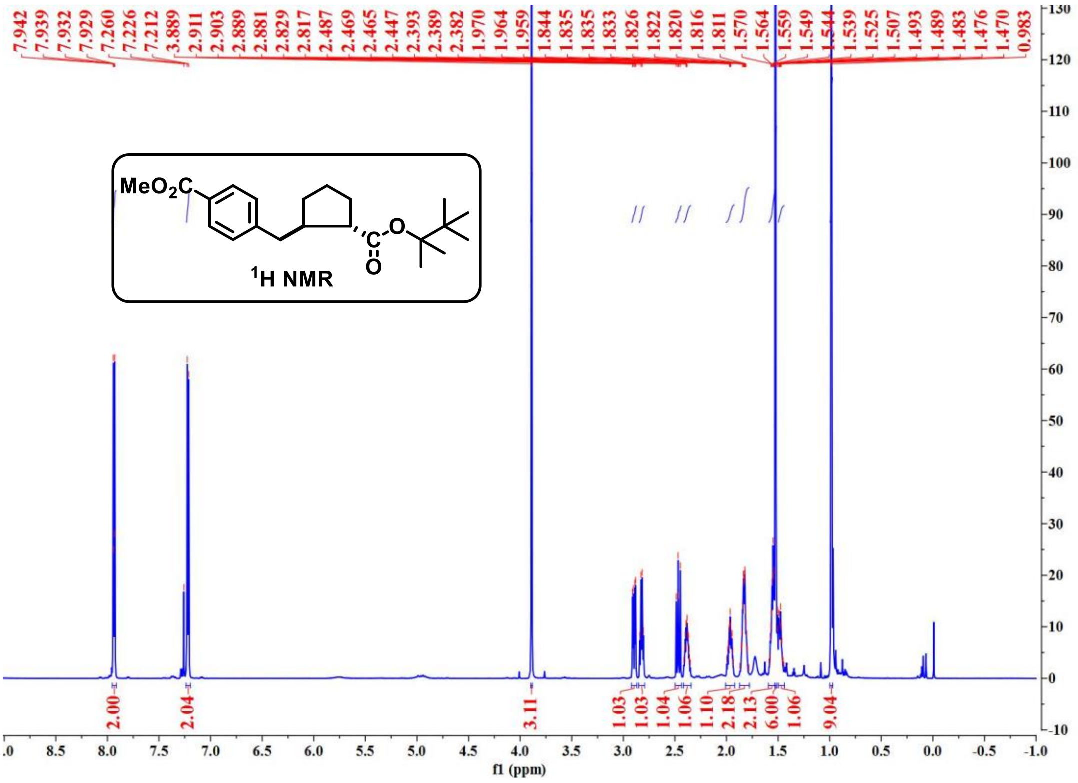

Supplementary Figure 142. ^{13}**C NMR Spectrum of Methyl 4-(((1*R*,2*S*)-2-(((2,3,3-trimethylbutan-2-yl)oxy)carbonyl)cyclopentyl)methyl)benzoate (151 MHz, CDCl₃)**

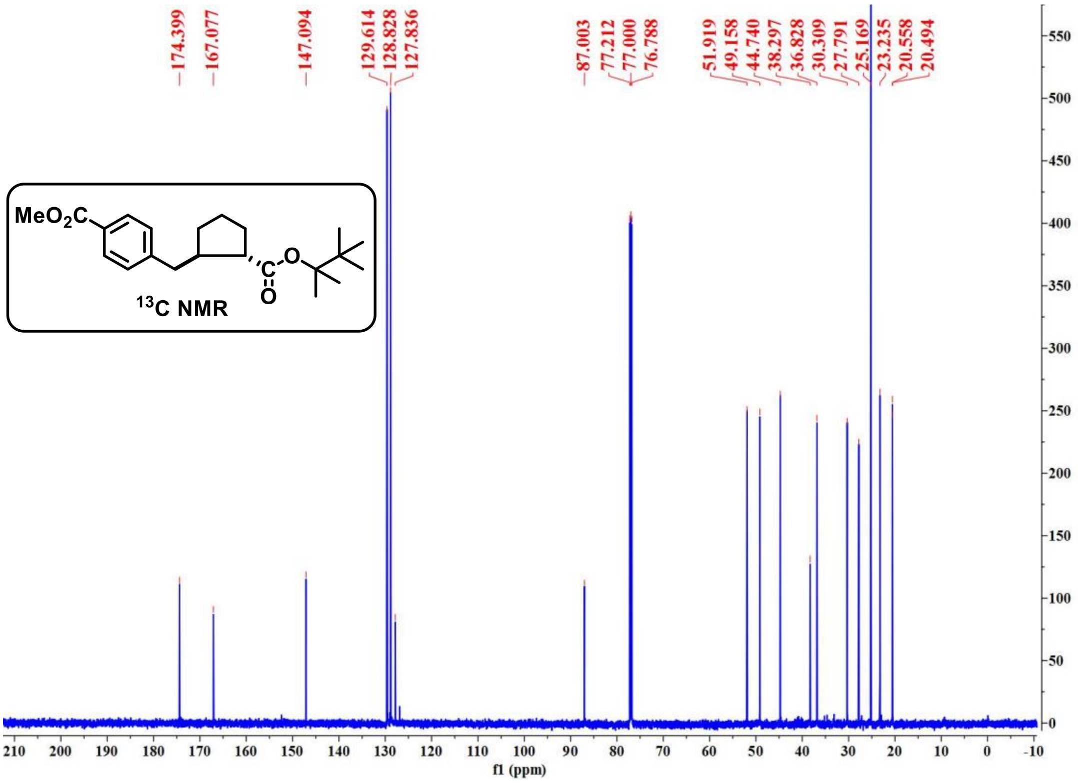

Supplementary Figure 143. ^{1}H NMR Spectrum of Methyl 4-(((1*R*, 2*R*)-2-(((2,3,3-trimethylbutan-2-yl)oxy)carbonyl)cyclopentyl)methyl)benzoate (600 MHz, $CDCl_3$)

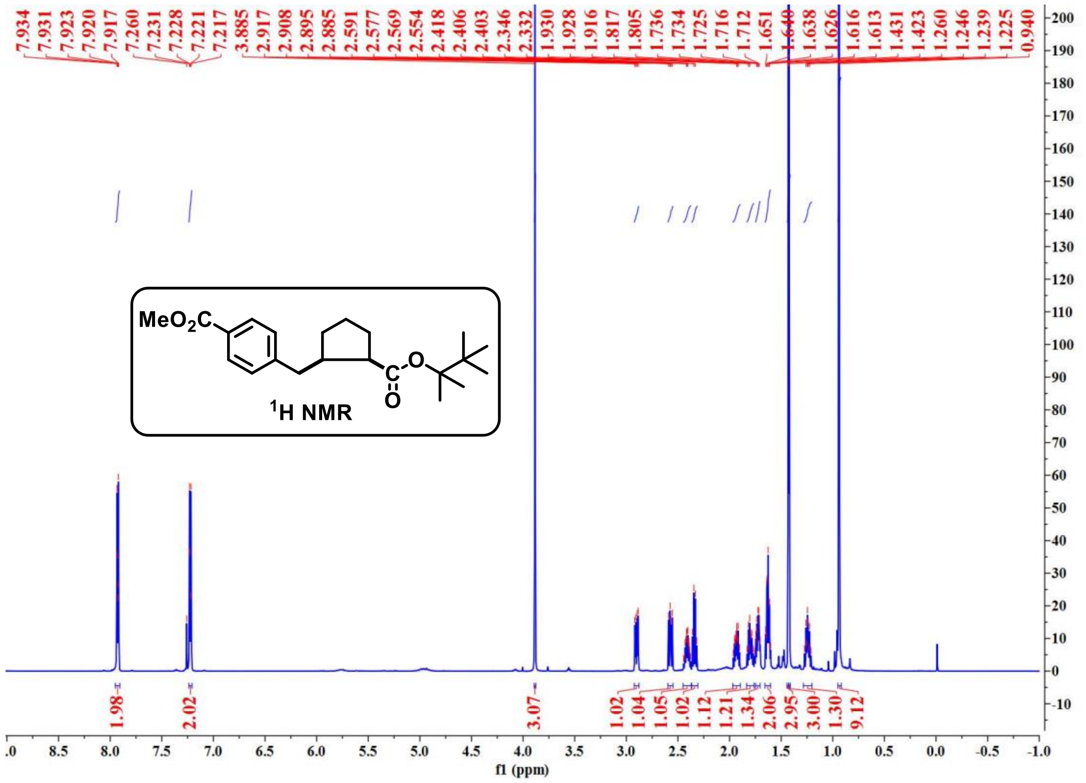

Supplementary Figure 144. ^{13}C NMR Spectrum of Methyl 4-(((1*R*, 2*R*)-2-(((2,3,3-trimethylbutan-2-yl)oxy)carbonyl)cyclopentyl)methyl)benzoate (151 MHz, $CDCl_3$)

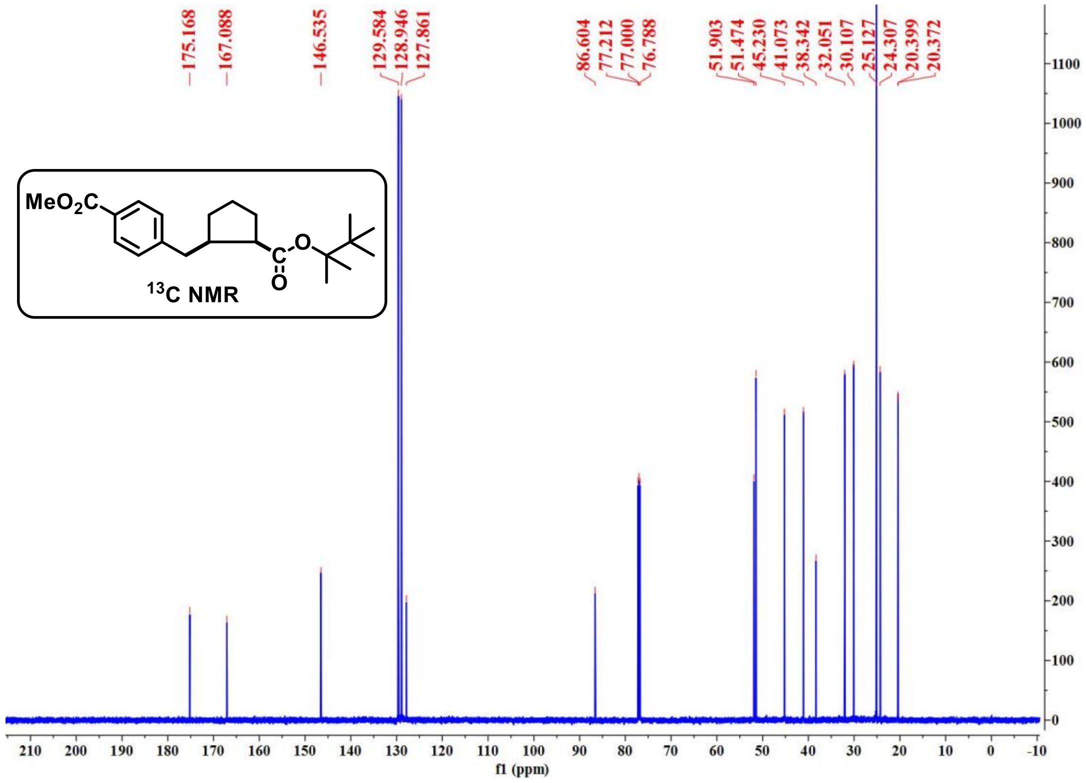

Supplementary: Characterization data for the Suboptimal substrates products

Supplementary Figure 145. ^{1}H NMR Spectrum of 2,3,3-trimethylbutan-2-yl (*R,E*)-2-methyl-4-phenylbut-3-enoate (400 MHz, $CDCl_3$)

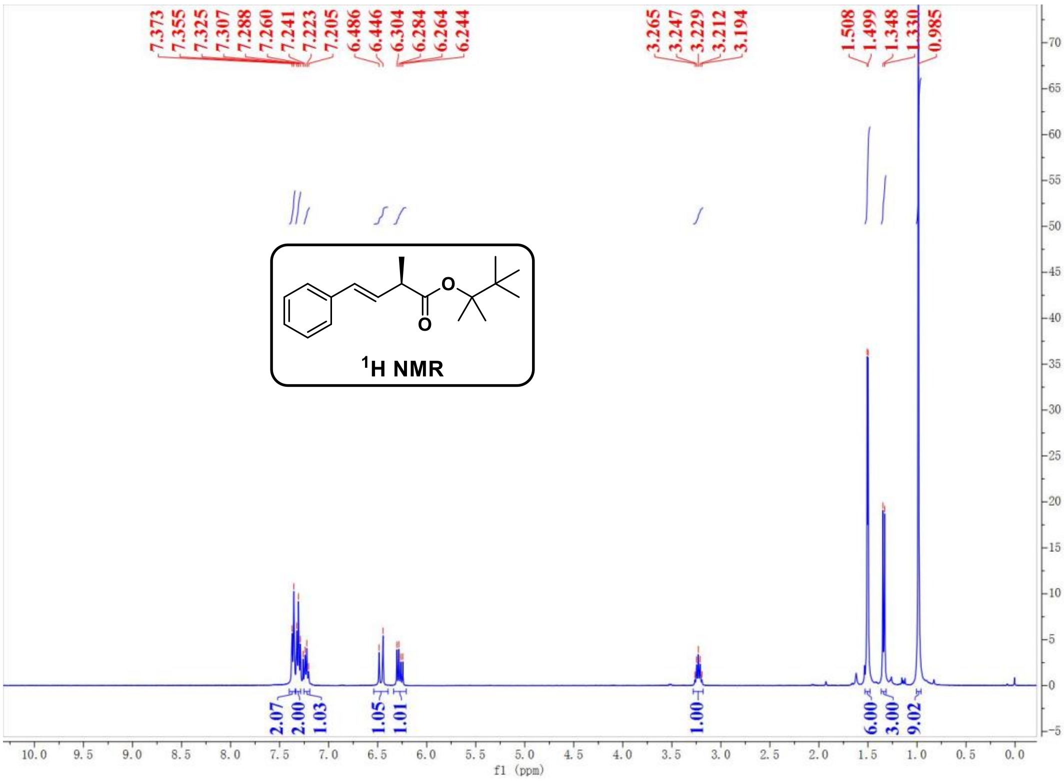

Supplementary Figure 146. ^{13}C NMR Spectrum of 2,3,3-trimethylbutan-2-yl (*R,E*)-2-methyl-4-phenylbut-3-enoate (101 MHz, $CDCl_3$)

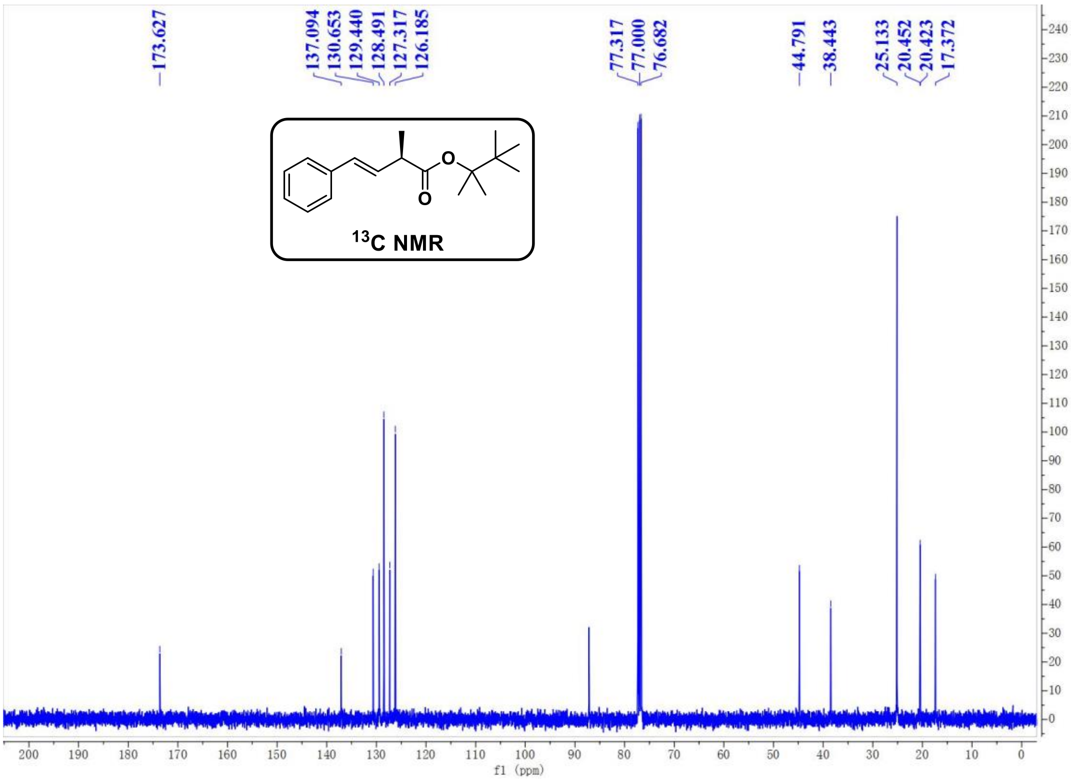

Supplementary Figure 147. ^{1}**H NMR Spectrum of 2,3,3-trimethylbutan-2-yl (*R*)-2-(1*H*-inden-2-yl)propanoate (400 MHz, CDCl**$_3$**)**

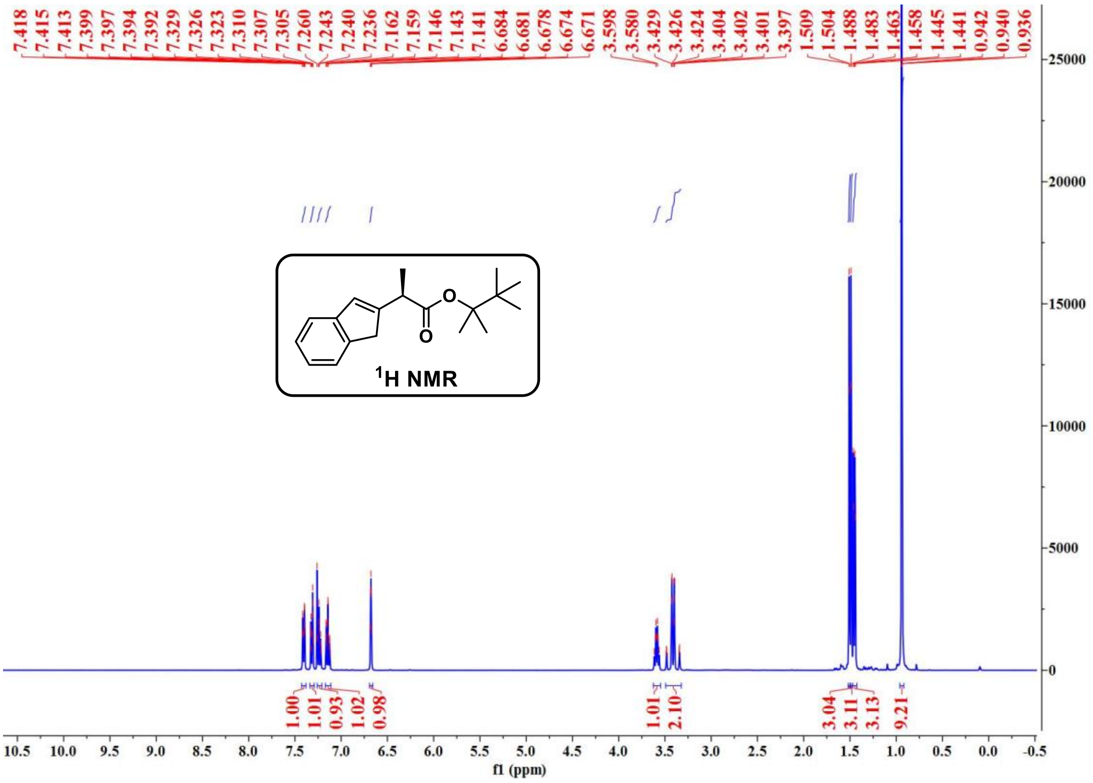

Supplementary Figure 148. ^{13}**C NMR Spectrum of 2,3,3-trimethylbutan-2-yl (*R*)-2-(1*H*-inden-2-yl)propanoate (101 MHz, CDCl**$_3$**)**

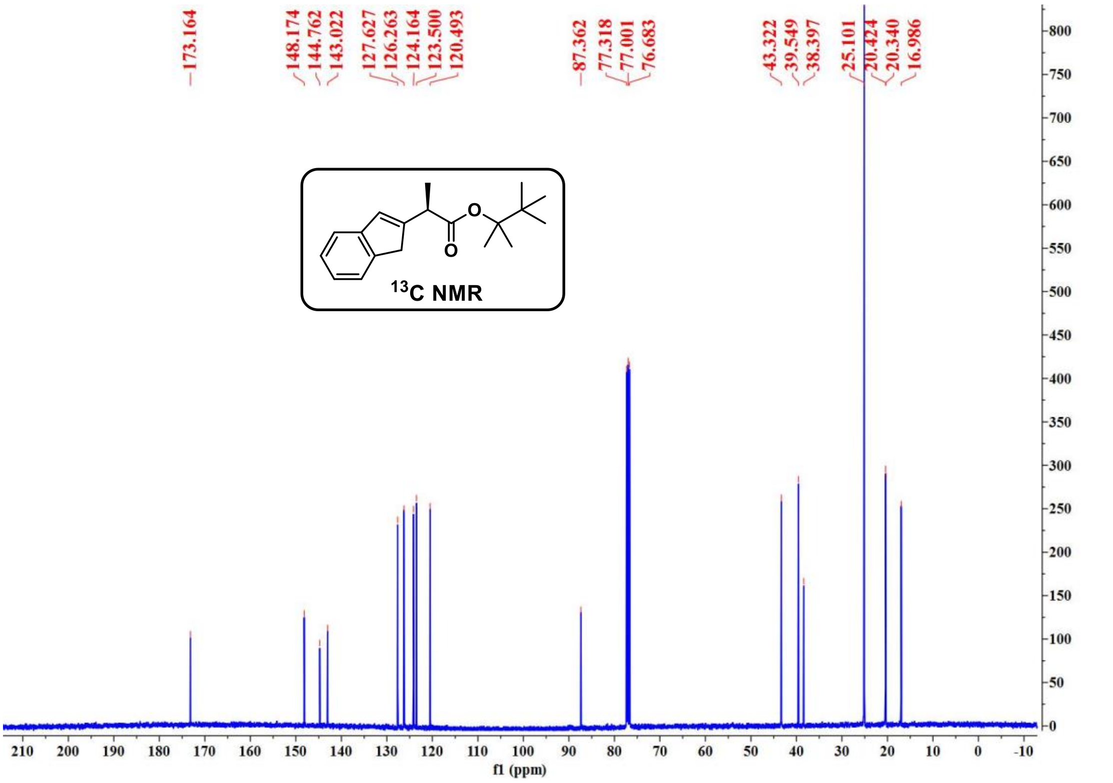

Supplementary Figure 149. ^{1}H NMR Spectrum of 2,3,3-trimethylbutan-2-yl (*R*)-2-(pyridin-3-yl)propanoate (400 MHz, $CDCl_3$)

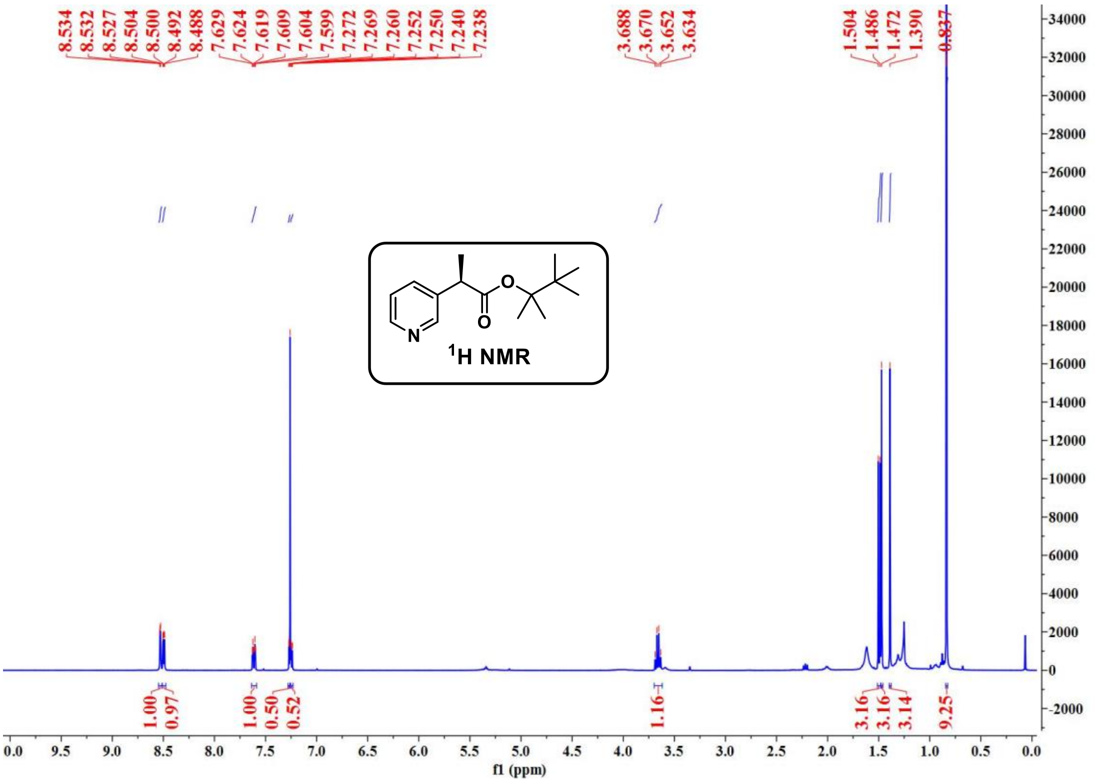

Supplementary Figure 150. ^{13}C NMR Spectrum of 2,3,3-trimethylbutan-2-yl (*R*)-2-(pyridin-3-yl)propanoate (101 MHz, $CDCl_3$)

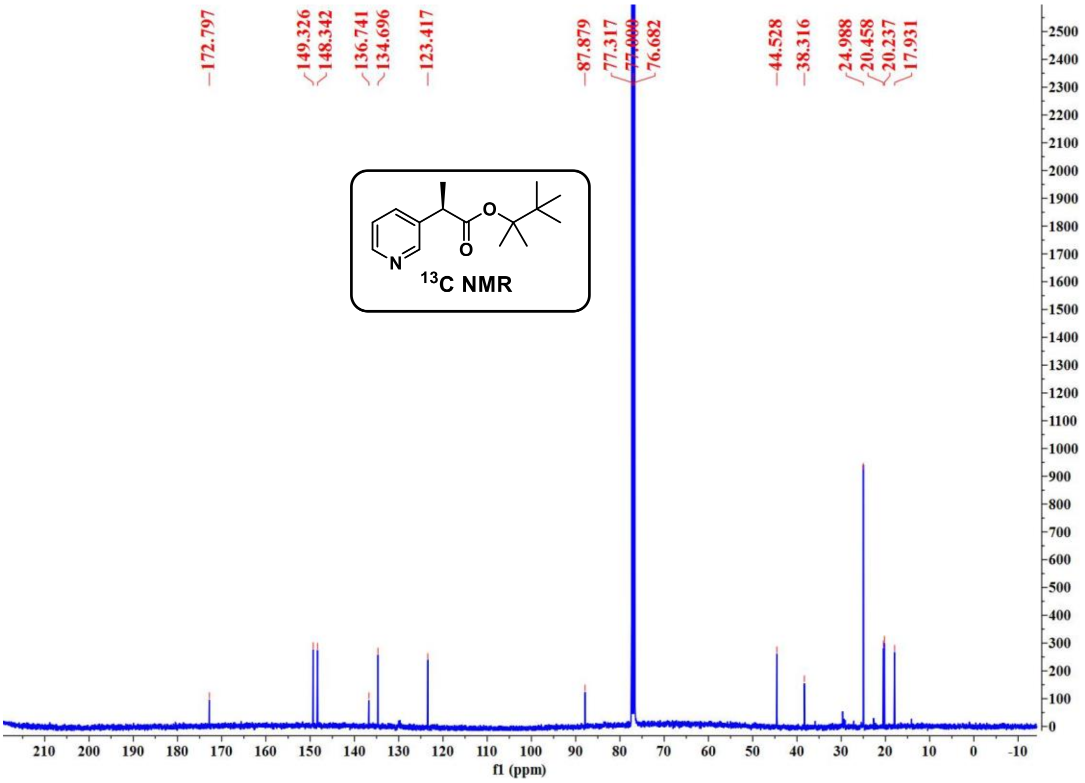

Supplementary Figure 151. ^{1}H NMR Spectrum of methyl 4-((*S*)-1-(((1*R*,2*S*,5*R*)-2-isopropyl-5-methylcyclohexyl)oxy)-1-oxopropan-2-yl)benzoate (400 MHz, CDCl$_3$)

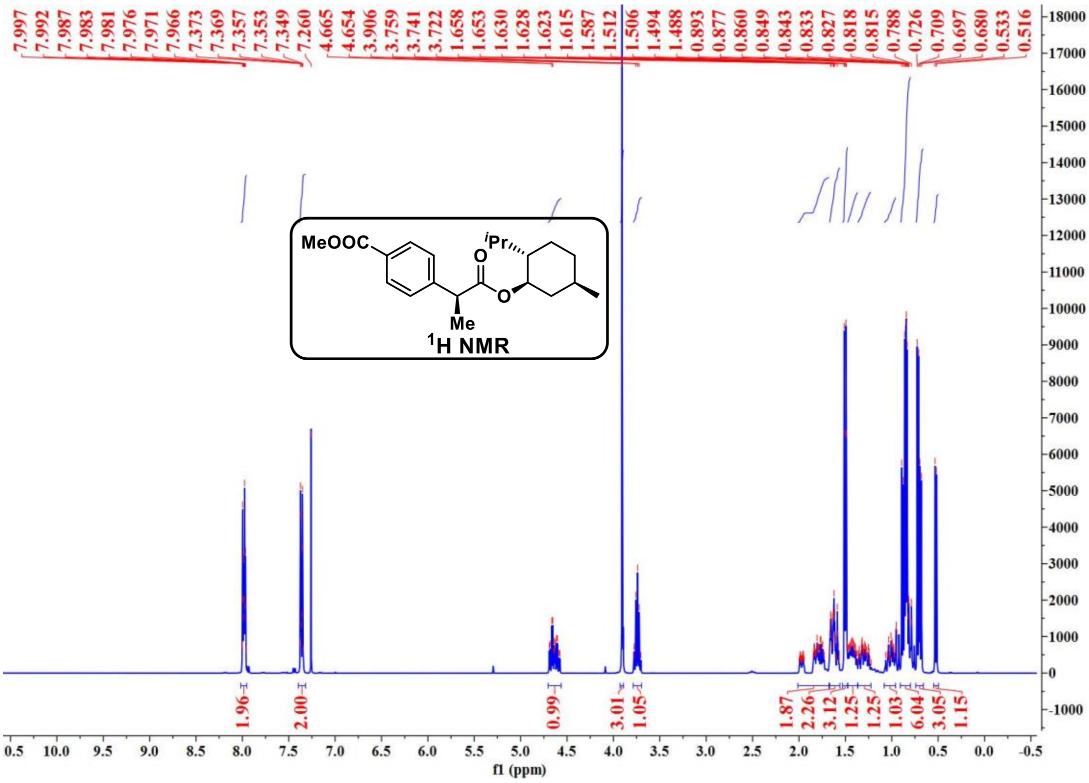

Supplementary Figure 152. ^{13}C NMR Spectrum of methyl 4-((*S*)-1-(((1*R*,2*S*,5*R*)-2-isopropyl-5-methylcyclohexyl)oxy)-1-oxopropan-2-yl)benzoate (101 Hz, CDCl$_3$)

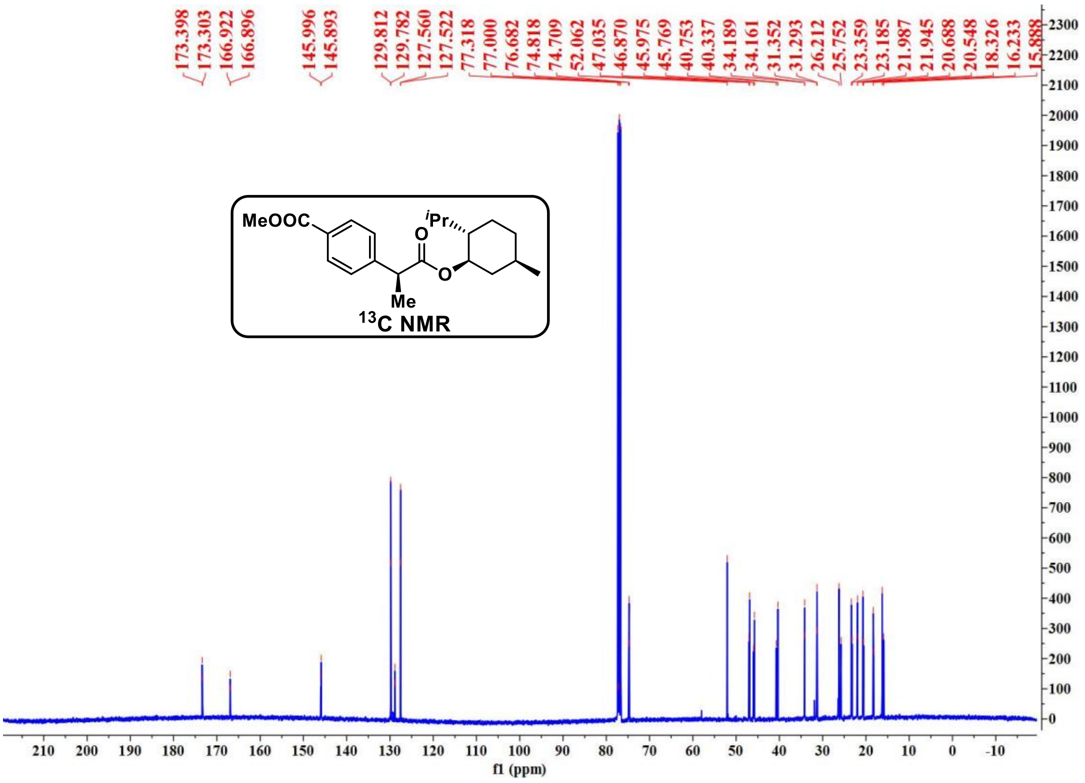

3.3 HPLC Spectra Data

Supplementary Figure 153. HPLC Chromatography of the Racemic Methyl 4-(1-oxo-1-((2,3,3-trimethylbutan-2-yl)oxy)propan-2-yl)benzoate **(**Daicel Chiralpak IC-H Column, *n*-Hexane: *i*-PrOH = 98:2, flow rate 1.0 mL/min, T = 25 °C, λ = 214 nm**)**

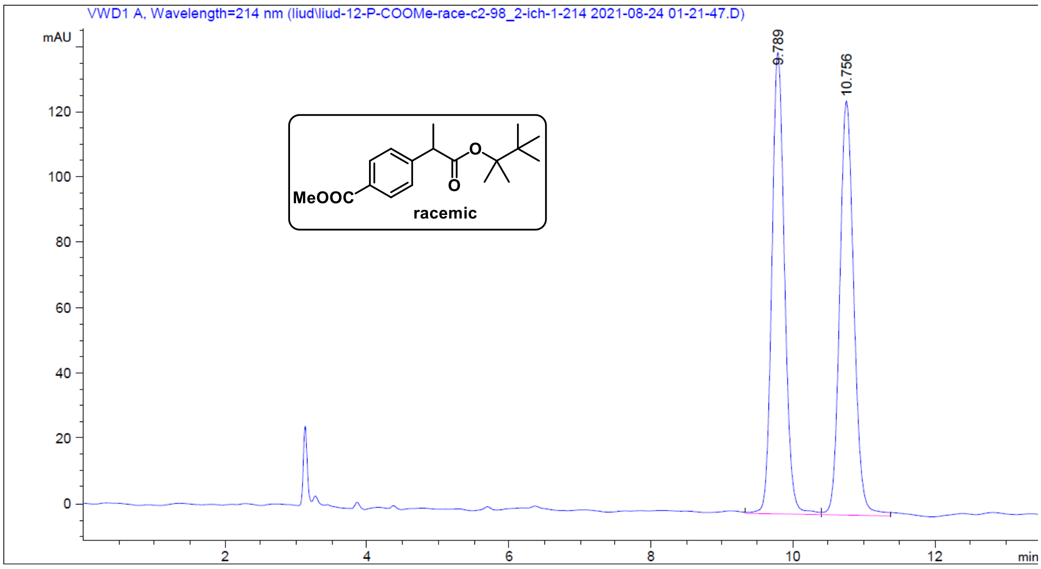

Peak	Ret Time [min]	Height [mAU]	Area %
1	9.789	141.26912	49.9
2	10.756	126.74439	50.1

Supplementary Figure 154. HPLC Chromatography of the Methyl (*R*)-4-(1-oxo-1-((2,3,3-trimethylbutan-2-yl)oxy)propan-2-yl)benzoate

(Daicel Chiralpak IC-H Column, *n*-Hexane: *i*-PrOH = 98:2, flow rate 1.0 mL/min, T = 25 °C, λ = 214 nm**)**

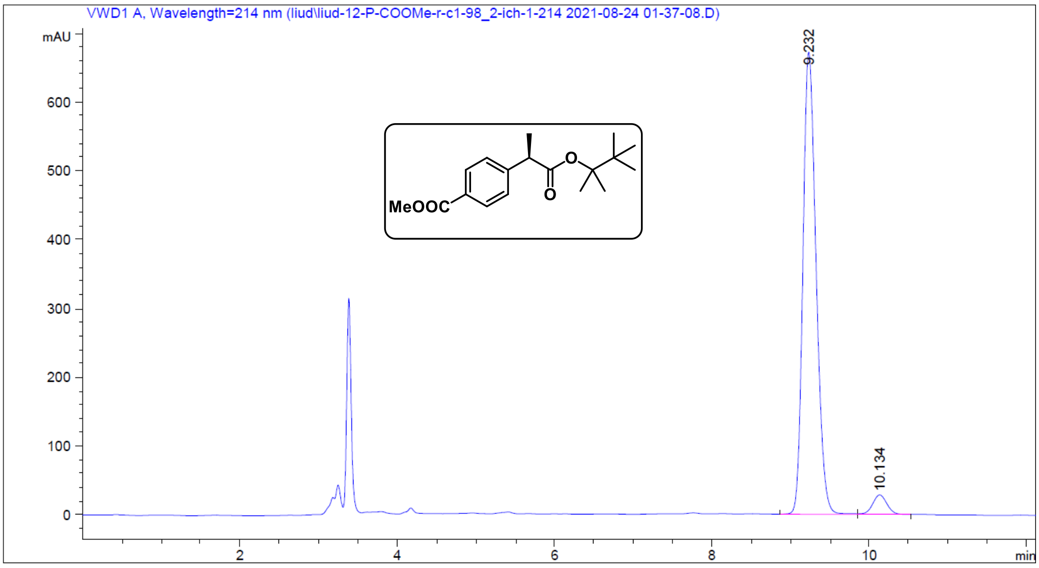

Peak	Ret Time [min]	Height [mAU]	Area %

1	9.232	671.72278	95.6006
2	10.134	28.40587	4.3994

Supplementary Figure 155. HPLC Chromatography of the Racemic Methyl 3-(1-oxo-1-((2,3,3-trimethylbutan-2-yl)oxy)propan-2-yl)benzoate **(**Daicel Chiralpak IC-H Column, *n*-Hexane: *i*-PrOH = 99:2, flow rate 1.0 mL/min, T = 25 °C, λ = 214 nm**)**

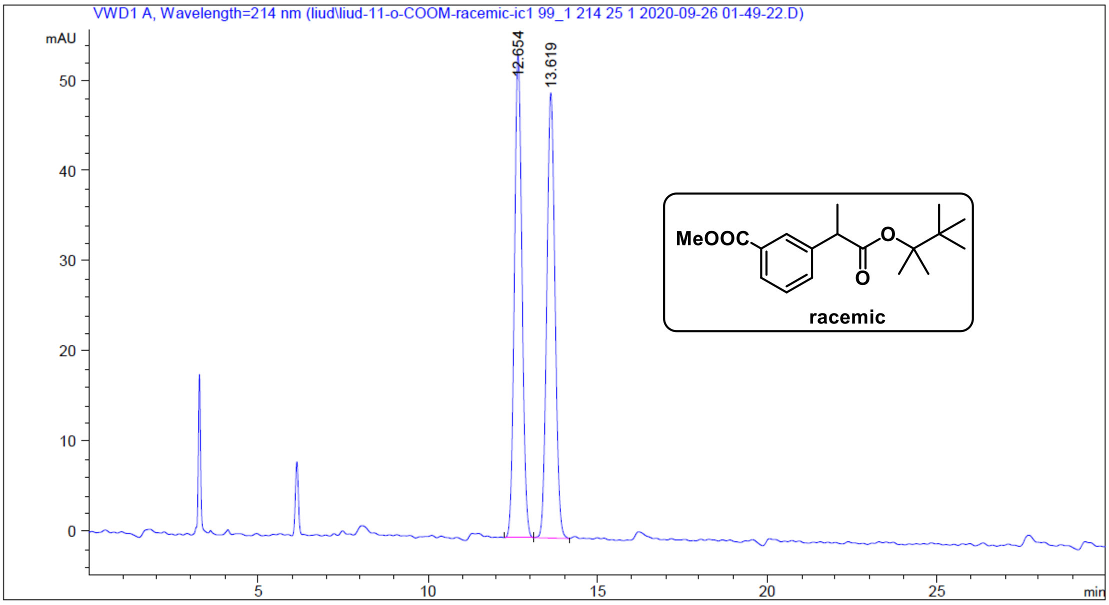

Peak	Ret Time [min]	Height [mAU]	Area %
1	12.654	53.52522	50.0500
2	13.619	49.39088	49.9500

Supplementary Figure 156. HPLC Chromatography of the Methyl (*R*)-3-(1-oxo-1-((2,3,3-trimethylbutan-2-yl)oxy)propan-2-yl)benzoate **(**Daicel Chiralpak IC-H Column, *n*-Hexane: *i*-PrOH = 98:2, flow rate 1.0 mL/min, T = 25 °C, λ = 214 nm**)**

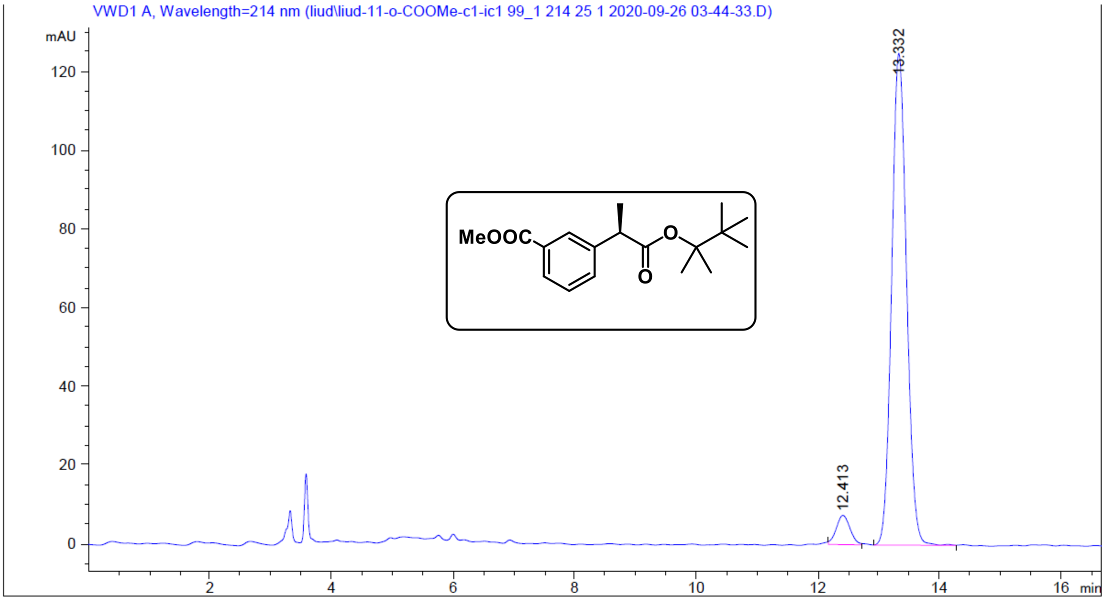

Peak	Ret Time [min]	Height [mAU]	Area %
1	12.413	7.44144	5.2398
2	13.332	125.03499	94.7602

Supplementary Figure 157. HPLC Chromatography of the Racemic Methyl 2-(1-oxo-1-((2,3,3-trimethylbutan-2-yl)oxy)propan-2-yl)benzoate **(**Daicel Chiralpak IC-H Column, *n*-Hexane: *i*-PrOH = 99:1, flow rate 1.0 mL/min, T = 25 °C, λ = 214 nm**)**

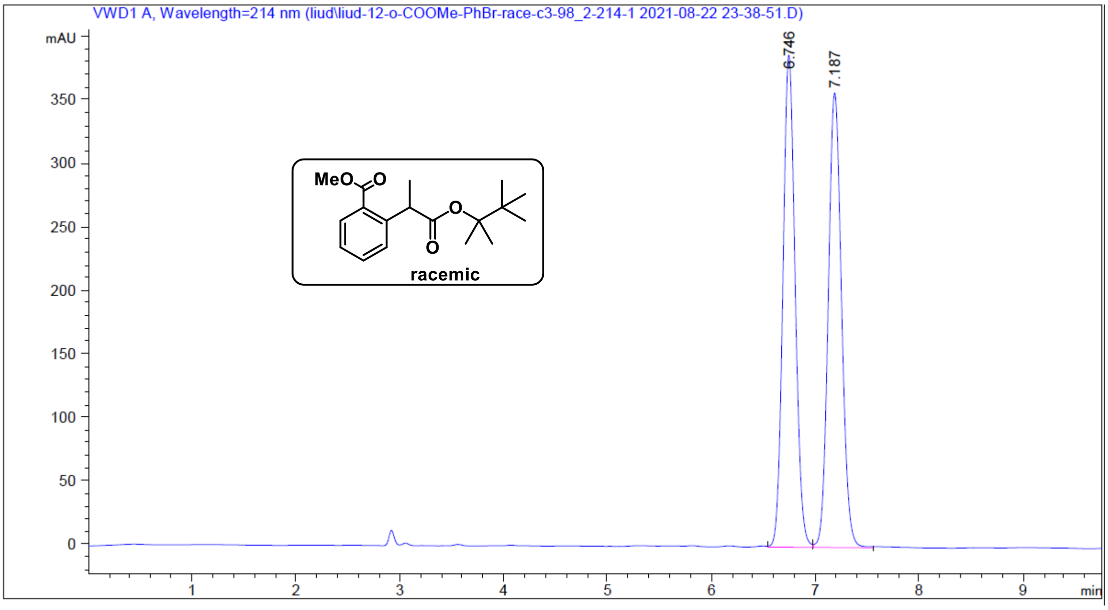

Peak	Ret Time [min]	Height [mAU]	Area %
1	6.746	387.88788	49.9371
2	7.187	358.35306	50.0629

Supplementary Figure 158. HPLC Chromatography of the Methyl (*R*)-2-(1-oxo-1-((2,3,3-trimethylbutan-2-yl)oxy)propan-2-yl)benzoate **(**Daicel Chiralpak IC-H Column, *n*-Hexane: *i*-PrOH = 98:2, flow rate 1.0 mL/min, T = 25 °C, λ = 214 nm**)**

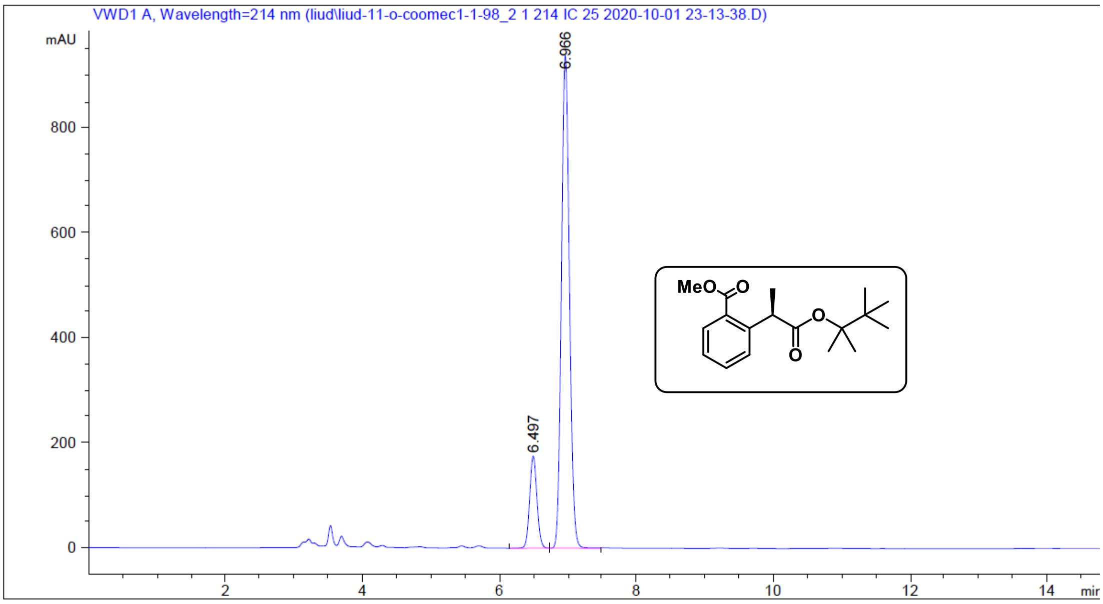

Peak	Ret Time [min]	Height [mAU]	Area %
1	6.497	174.68623	14.5701
2	6.966	940.44019	85.4299

Supplementary Figure 159. HPLC Chromatography of the Racemic tert-butyl 4-(1-oxo-1-((2,3,3-trimethylbutan-2-yl)oxy)propan-2-yl)benzoate (Daicel Chiralpak IC-H Column, *n*-Hexane: *i*-PrOH = 98:2, flow rate 1.0 mL/min, T = 25 °C, λ = 214 nm)

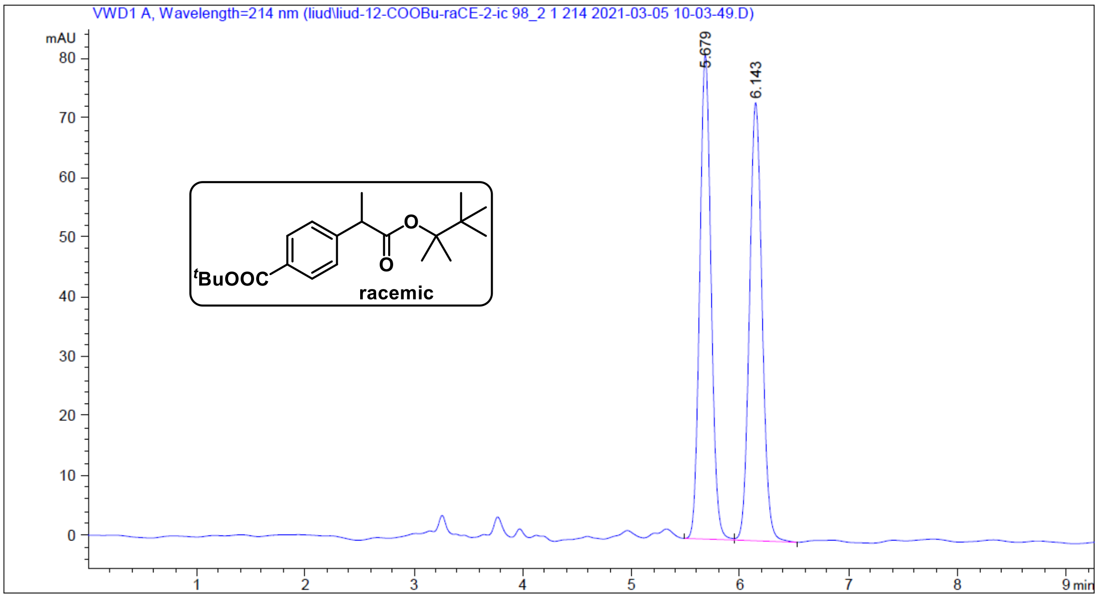

Peak	Ret Time [min]	Height [mAU]	Area %
1	5.679	81.24709	49.7772
2	6.143	73.52419	50.2228

Supplementary Figure 160. HPLC Chromatography of the tert-butyl (*R*)-4-(1-oxo-1-((2,3,3-trimethylbutan-2-

yl)oxy)propan-2-yl)benzoate (Daicel Chiralpak IC-H Column, *n*-Hexane: *i*-PrOH = 98:2, flow rate 1.0 mL/min, T = 25 °C, λ = 214 nm)

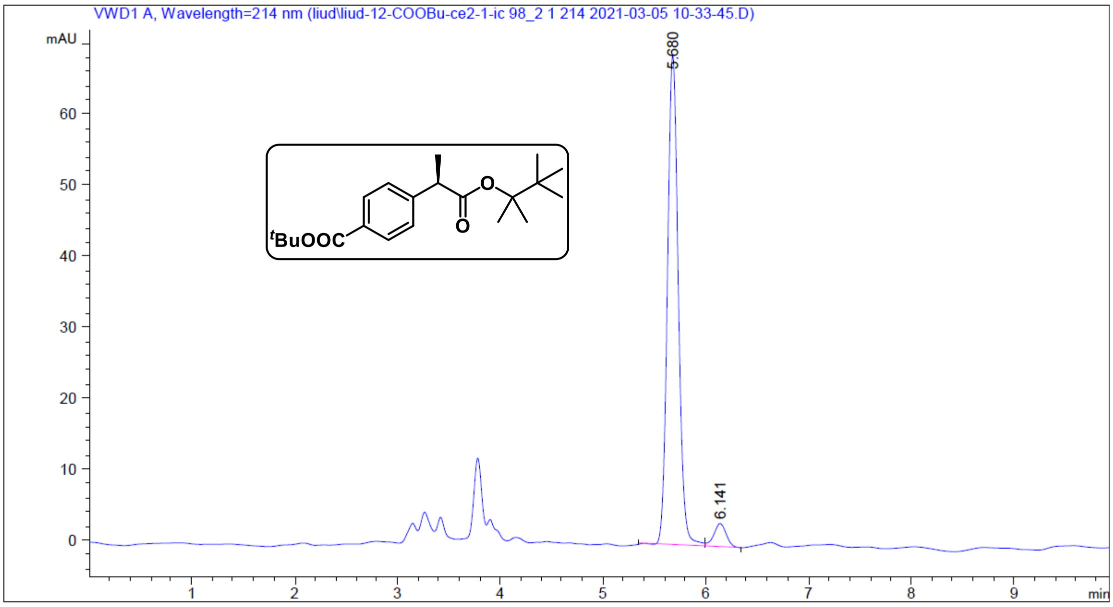

Peak	Ret Time [min]	Height [mAU]	Area %
1	5.6800	68.83549	94.9611
2	6.141	3.22974	5.0389

Supplementary Figure 161. HPLC Chromatography of the Racemic 2,3,3-trimethylbutan-2-yl 2-(4-benzoylphenyl)propanoate

(Daicel Chiralpak IC-H Column, *n*-Hexane: *i*-PrOH = 98:2, flow rate 0.7 mL/min, T = 25 °C, λ = 214 nm)

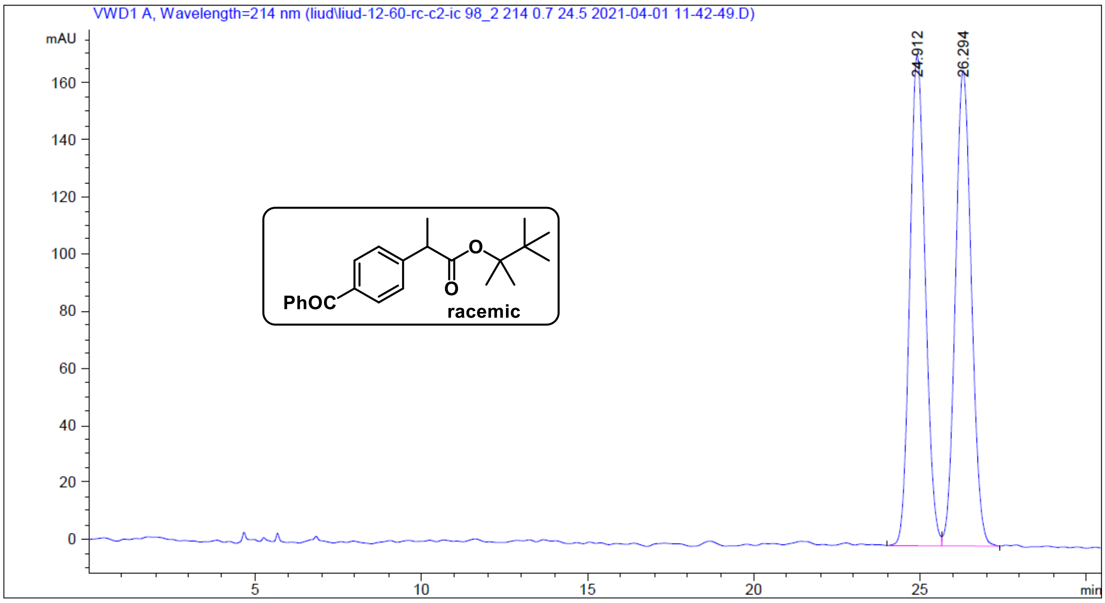

Peak	Ret Time [min]	Height [mAU]	Area %
1	24.912	171.88701	49.8524

2	26.294	166.60721	50.1476

Figure 162. HPLC Chromatography of the 2,3,3-trimethylbutan-2-yl (*R*)-2-(4-benzoylphenyl)propanoate (Daicel Chiralpak IC-H Column, *n*-Hexane: *i*-PrOH = 98:2, flow rate 0.7 mL/min, T = 25 °C, λ = 214 nm)

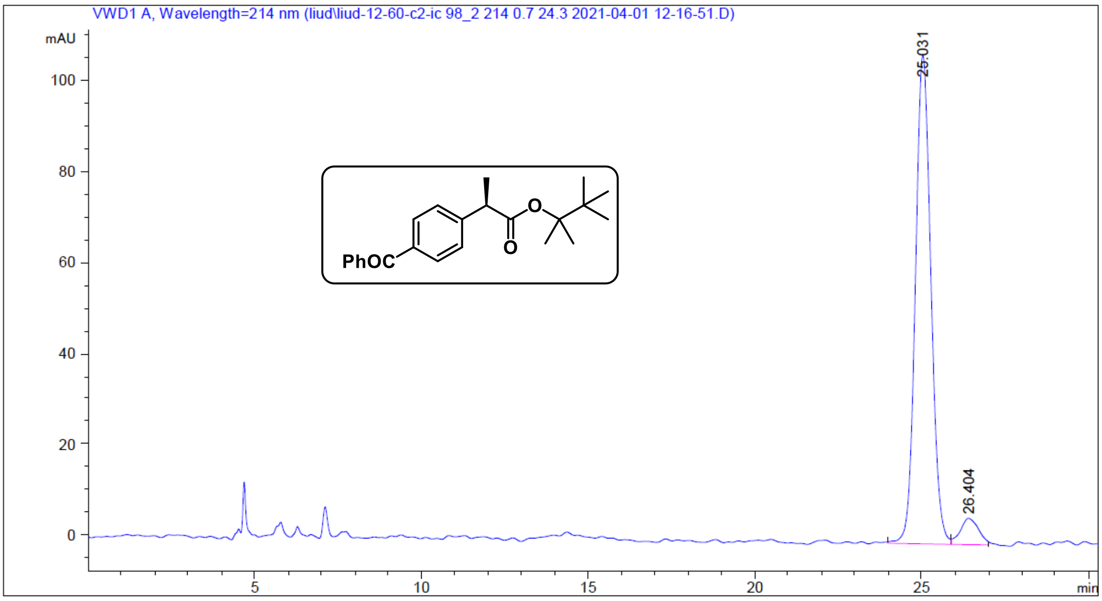

Peak	Ret Time [min]	Height [mAU]	Area %
1	25.031	107.29478	94.0639
2	26.404	5.75726	5.9361

Figure 163. HPLC Chromatography of the Racemic 2,3,3-trimethylbutan-2-yl 2-(4-acetylphenyl)propanoate **(**Daicel Chiralpak IC-H Column, *n*-Hexane: *i*-PrOH = 99:1, flow rate 1.0 mL/min, T = 25 °C, λ = 214 nm**)**

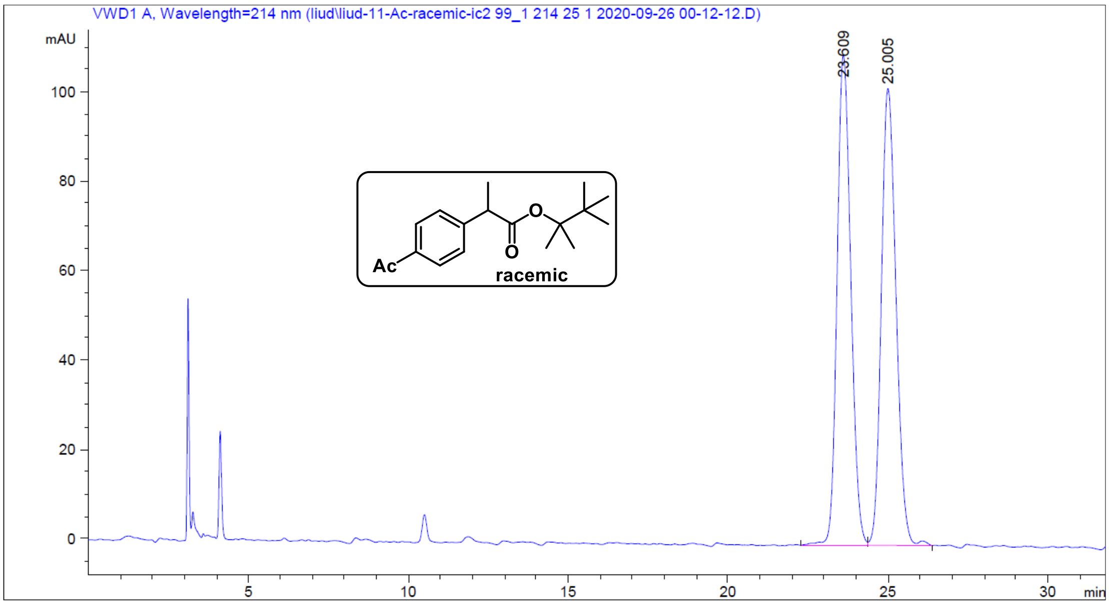

Peak	Ret Time [min]	Height [mAU]	Area %
1	23.609	109.59139	50.0808

2	25.005	102.17345	49.9192

Figure 164. HPLC Chromatography of the 2,3,3-trimethylbutan-2-yl (*R*)-2-(4-acetylphenyl)propanoate **(**Daicel Chiralpak IC-H Column, *n*-Hexane: *i*-PrOH = 99:1, flow rate 1.0 mL/min, T = 25 °C, λ = 214 nm**)**

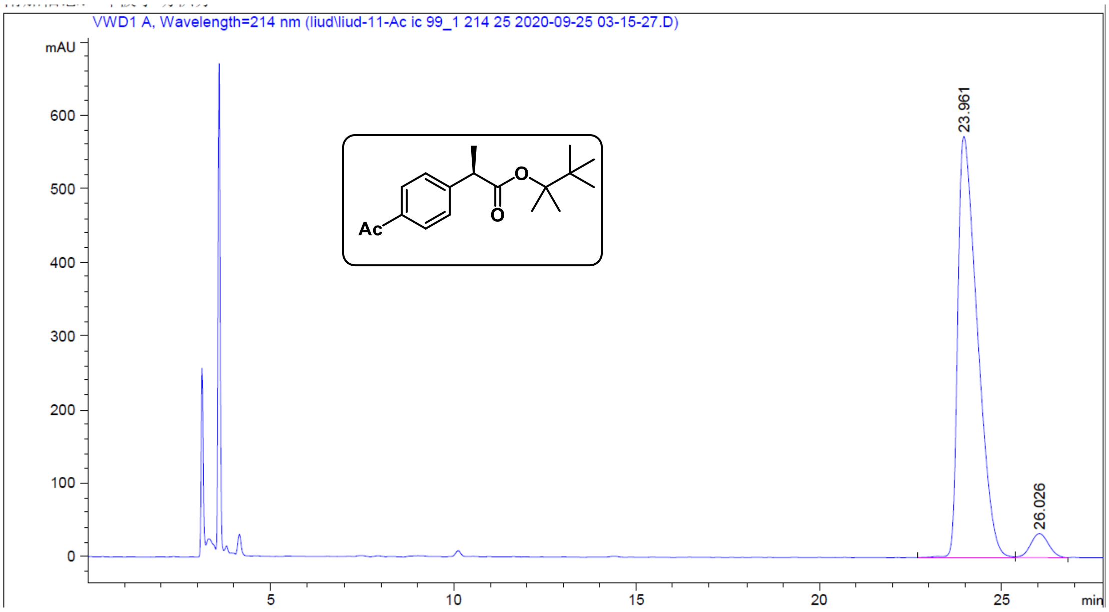

Peak	Ret Time [min]	Height [mAU]	Area %
1	23.961	572.73358	95.0117
2	26.026	32.62696	4.9883

Figure 165. HPLC Chromatography of the 2,3,3-trimethylbutan-2-yl 2-(4-(dimethylcarbamoyl)phenyl)propanoate (Daicel Chiralpak AD-H Column, *n*-Hexane: *i*-PrOH = 95:5, flow rate 1.0 mL/min, T = 25 °C, λ = 214 nm)

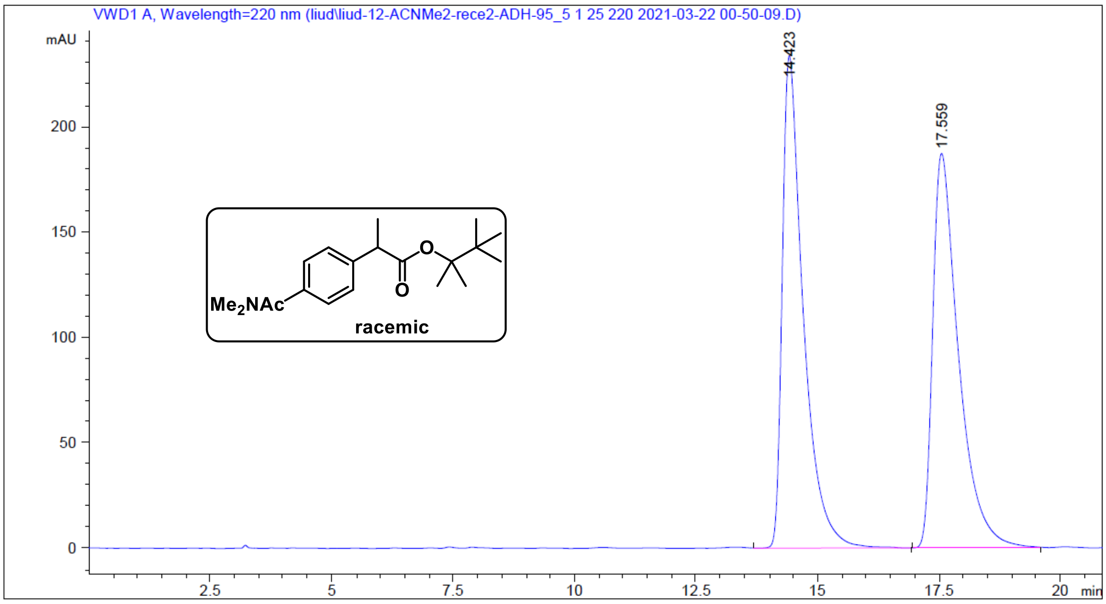

Peak	Ret Time [min]	Height [mAU]	Area %
1	14.423	233.32059	50.3498

2	17.559	186.87086	49.6502

Figure 166. HPLC Chromatography of the 2,3,3-trimethylbutan-2-yl (*R*)-2-(4-(dimethylcarbamoyl)phenyl)propanoate

(Daicel Chiralpak AD-H Column, *n*-Hexane: *i*-PrOH = 95:5, flow rate 1.0 mL/min, T = 25 °C, λ = 214 nm)

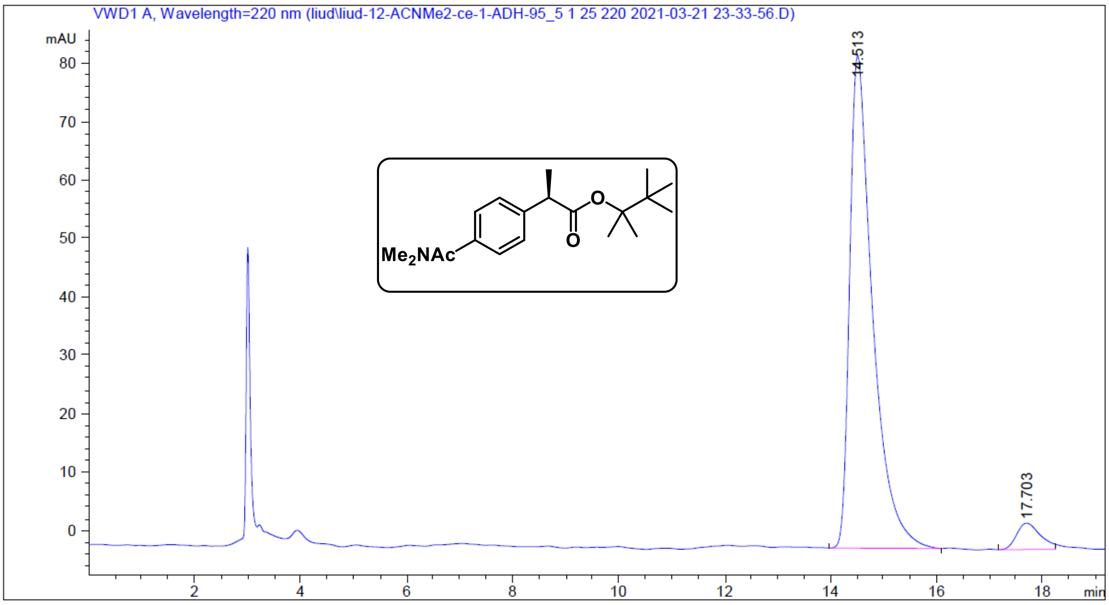

Peak	Ret Time [min]	Height [mAU]	Area %
1	14.513	84.41722	94.7125
2	17.703	4.52220	5.2875

Figure 167. HPLC Chromatography of the Racemic 2-(methylthio)ethyl-4-(1-oxo-1-((2,3,3-trimethylbutan-2-yl)oxy)propan-2-yl)benzoate (Daicel Chiralpak IC-H Column, *n*-Hexane: *i*-PrOH = 98:2, flow rate 0.5 mL/min, T = 25 °C, λ = 214 nm)

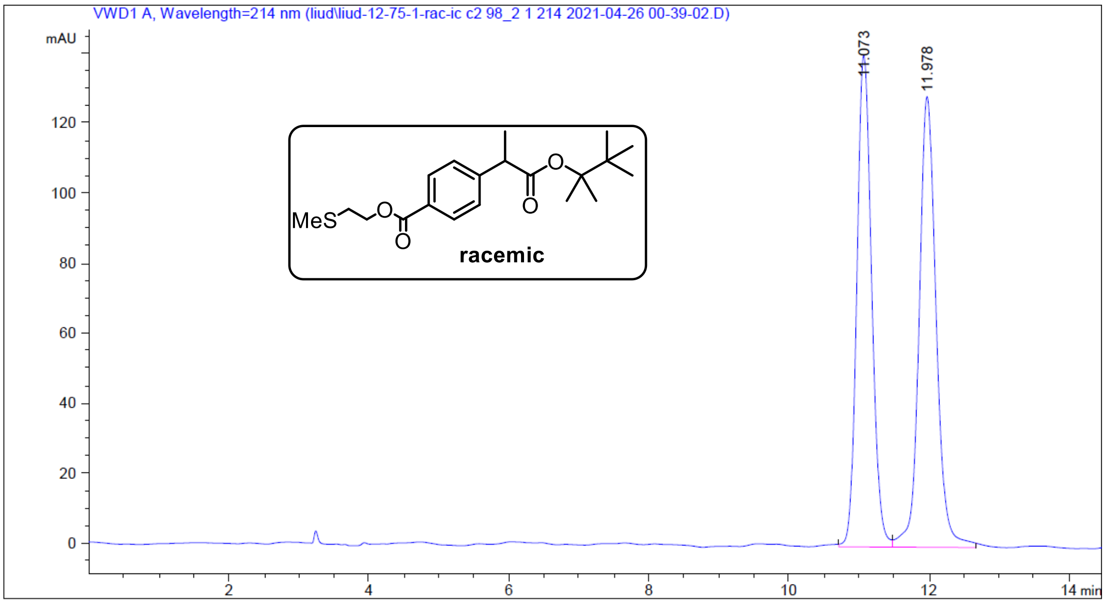

Peak	Ret Time [min]	Height [mAU]	Area %
1	11.073	140.54128	48.5621
2	11.978	128.78978	51.4379

Figure 168. HPLC Chromatography of the 2-(methylthio)ethyl (*R*)-4-(1-oxo-1-((2,3,3-trimethylbutan-2-yl)oxy)propan-2-yl)benzoate

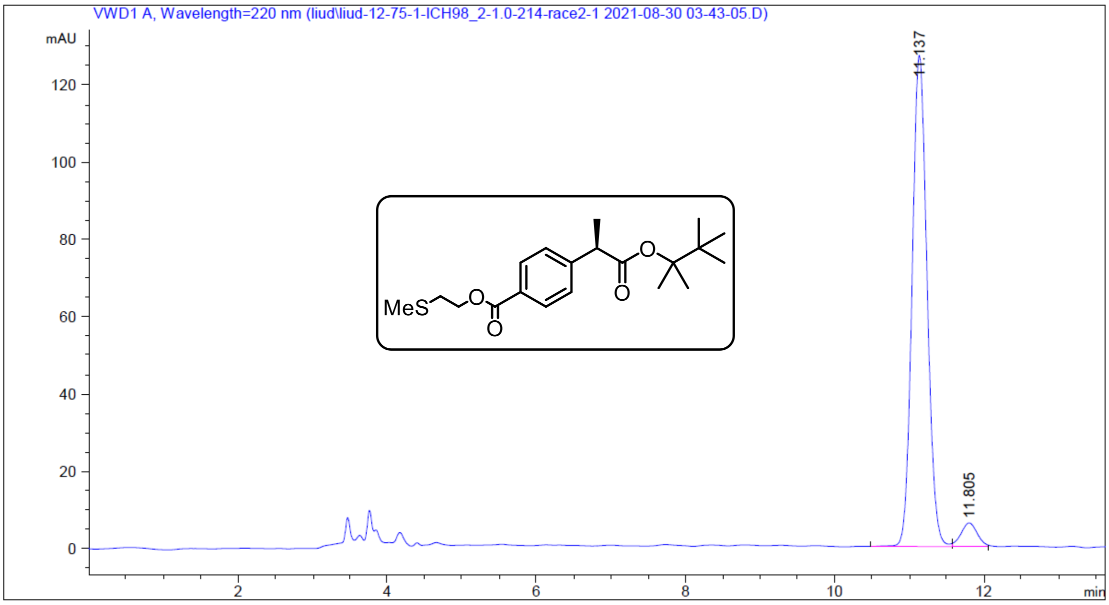

Peak	Ret Time [min]	Height [mAU]	Area %
1	11.137	127.12115	95.0365
2	11.805	6.11883	4.9635

Figure 169. HPLC Chromatography of the Racemic 2,3,3-trimethylbutan-2-yl 2-(3-methoxy-4-methylphenyl)propanoate (Daicel Chiralpak OJ-H Column, *n*-Hexane: *i*-PrOH = 99.5:0.5, flow rate 0.5 mL/min, T = 25 °C, λ = 214 nm)

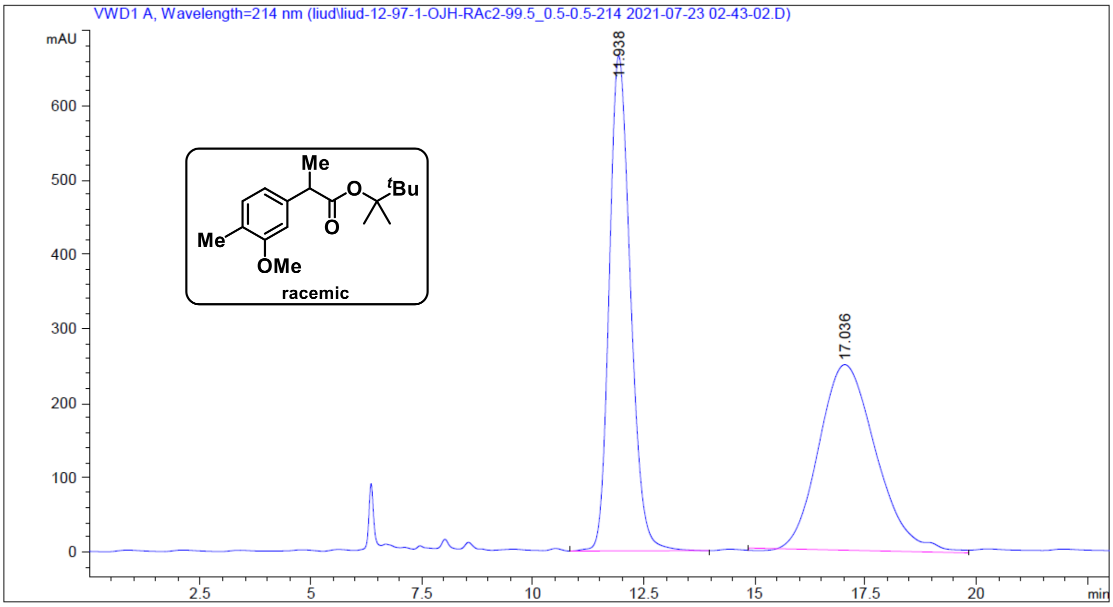

Peak	Ret Time [min]	Height [mAU]	Area %
1	11.938	665.92261	49.7915
2	17.036	249.51141	50.2085

Supplementary Figure 170. HPLC Chromatography of the 2,3,3-trimethylbutan-2-yl (*R*)-2-(3-methoxy-4-methylphenyl)propanoate

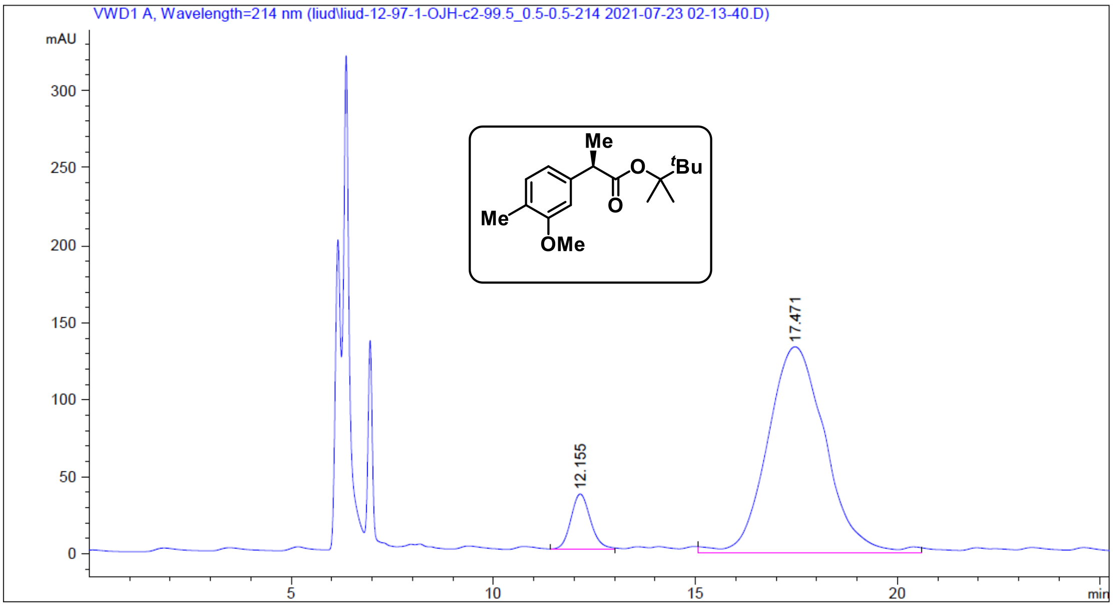

Peak	Ret Time [min]	Height [mAU]	Area %
1	12.155	35.53997	8.2020
2	17.471	133.46281	91.7980

Supplementary Figure 171. HPLC Chromatography of the Racemic 2,3,3-trimethylbutan-2-yl 2-(4-(tert-butyl)phenyl)propanoate

(Daicel Chiralpak OJ-H Column, *n*-Hexane: *i*-PrOH = 99.5:0.5, flow rate 0.5 mL/min, T = 25 °C, λ = 214 nm)

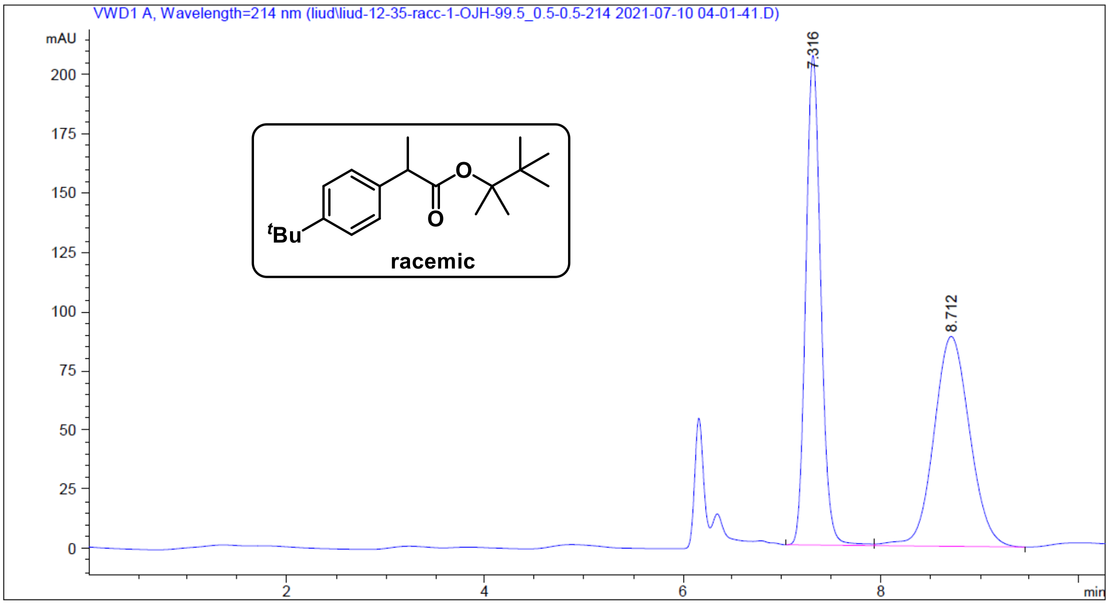

Peak	Ret Time [min]	Height [mAU]	Area %
1	7.316	206.69406	49.3656
2	8.712	88.58203	50.6344

Supplementary Figure 172. HPLC Chromatography of the 2,3,3-trimethylbutan-2-yl (*R*)-2-(4-(tert-butyl)phenyl)propanoate

(Daicel Chiralpak OJ-H Column, *n*-Hexane: *i*-PrOH = 99.5:0.5, flow rate 0.5 mL/min, T = 25 °C, λ = 214 nm)

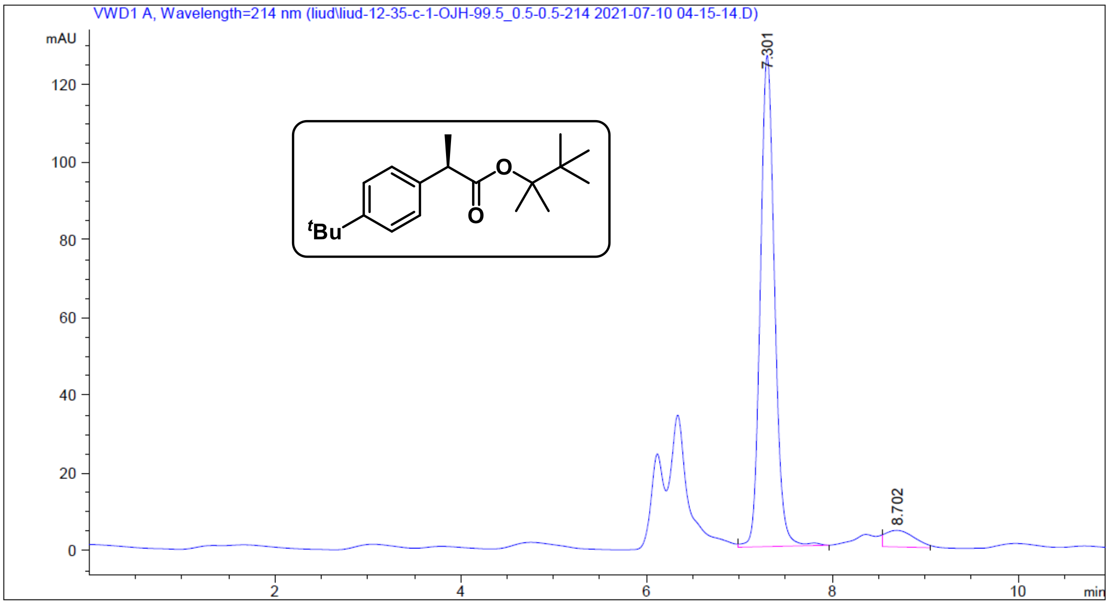

Peak	Ret Time [min]	Height [mAU]	Area %
1	7.301	126.50109	93.7245
2	8.702	4.25858	6.2755

Supplementary Figure 173. HPLC Chromatography of the Racemic 2,3,3-trimethylbutan-2-yl 2-([1,1'-biphenyl]-4-yl)propanoate (Daicel Chiralpak IC-H Column, *n*-Hexane: *i*-PrOH = 99:1, flow rate 1.0 mL/min, T = 25 °C, λ = 214 nm)

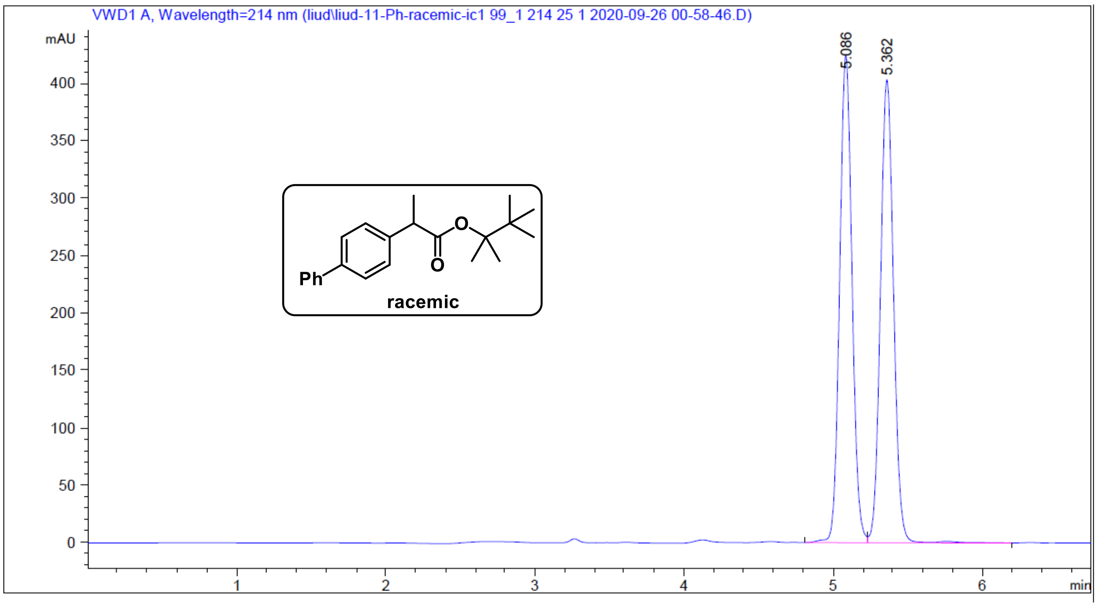

Peak	Ret Time [min]	Height [mAU]	Area %
1	5.086	424.37100	49.8905
2	5.362	403.37738	50.1095

Supplementary Figure 174. HPLC Chromatography of the 2,3,3-trimethylbutan-2-yl (*R*)-2-([1,1'-biphenyl]-4-yl)propanoate (Daicel Chiralpak IC-H Column, *n*-Hexane: *i*-PrOH = 99:1, flow rate 1.0 mL/min, T = 25 °C, λ = 214 nm)

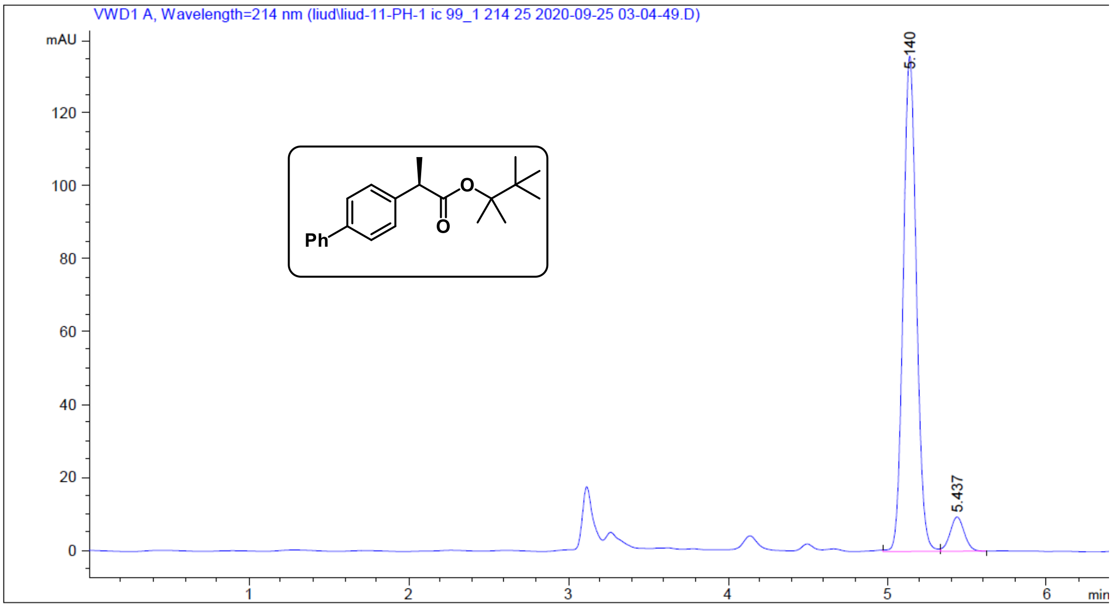

Peak	Ret Time [min]	Height [mAU]	Area %

1	5.140	136.66783	93.1034
2	5.437	9.45816	6.8966

Supplementary Figure 175. HPLC Chromatography of the Racemic 2,3,3-trimethylbutan-2-yl 2-(4-(methylthio)phenyl)propanoate

(Daicel Chiralpak IC-H Column, *n*-Hexane: *i*-PrOH = 98:2, flow rate 0.7 mL/min, T = 25 °C, λ = 214 nm)

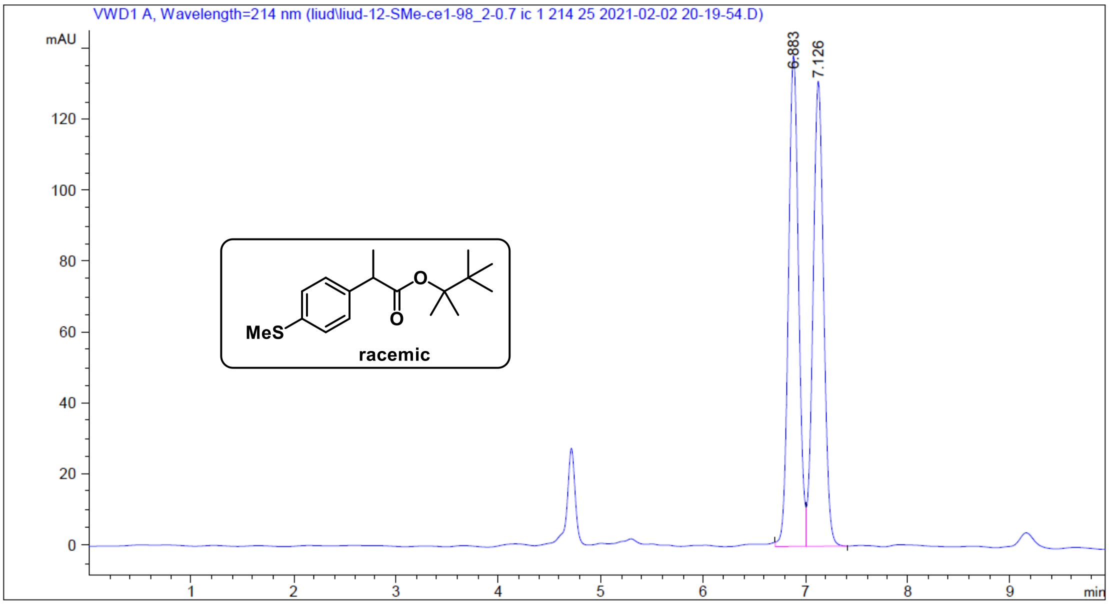

Peak	Ret Time [min]	Height [mAU]	Area %
1	6.883	138.72688	50.1629
2	7.126	130.60764	49.8371

Supplementary Figure 176. HPLC Chromatography of the 2,3,3-trimethylbutan-2-yl (*R*)-2-(4-(methylthio)phenyl)propanoate

(Daicel Chiralpak IC-H Column, *n*-Hexane: *i*-PrOH = 98:2, flow rate 0.7 mL/min, T = 25 °C, λ = 214 nm)

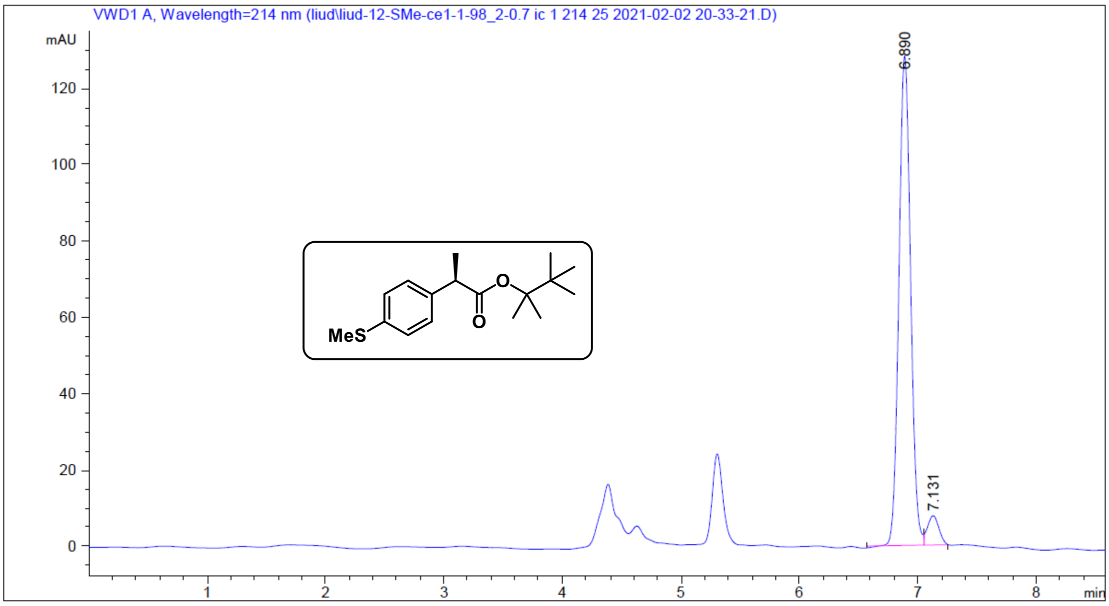

Peak	Ret Time [min]	Height [mAU]	Area %
1	6.890	128.67206	94.5104
2	7.131	7.70847	5.4896

Supplementary Figure 177. HPLC Chromatography of the Racemic 2,3,3-trimethylbutan-2-yl 2-(4-((trifluoromethyl)thio)phenyl)propanoate **(**Daicel Chiralpak IC-H Column, *n*-Hexane: *i*-PrOH = 100:0, flow rate 0.4 mL/min, T = 25 °C, λ = 214 nm**)**

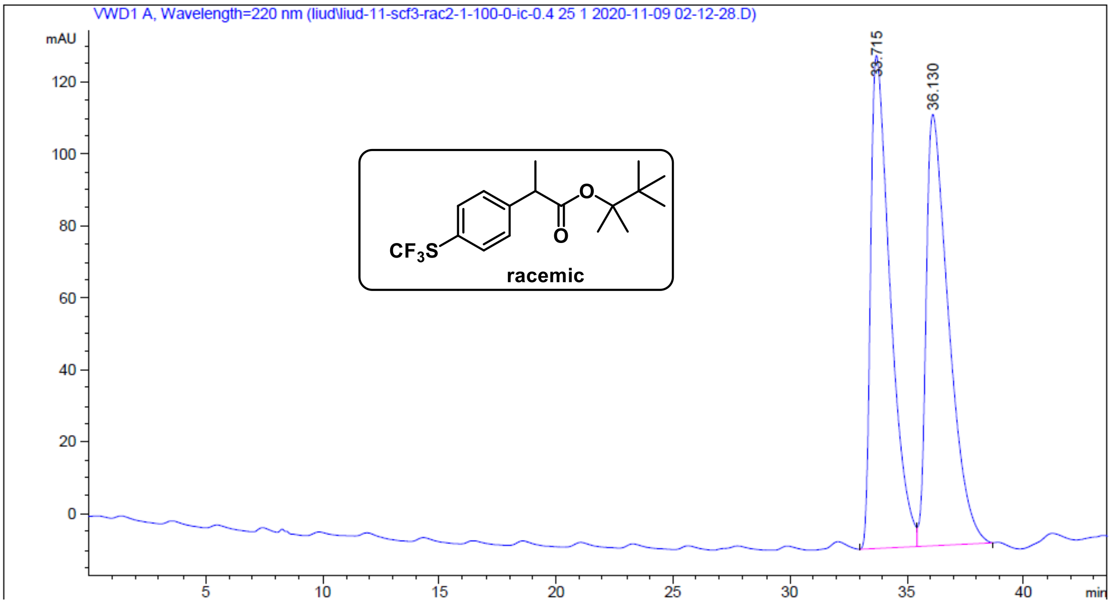

Peak	Ret Time [min]	Height [mAU]	Area %
1	33.715	137.07243	49.7899
2	36.130	119.92829	50.2101

Supplementary Figure 178. HPLC Chromatography of the 2,3,3-trimethylbutan-2-yl (*R*)-2-(4-((trifluoromethyl)thio)phenyl)propanoate **(**Daicel Chiralpak IC-H Column, *n*-Hexane: *i*-PrOH = 100:0, flow rate 0.4 mL/min, T = 25 °C, λ = 214 nm**)**

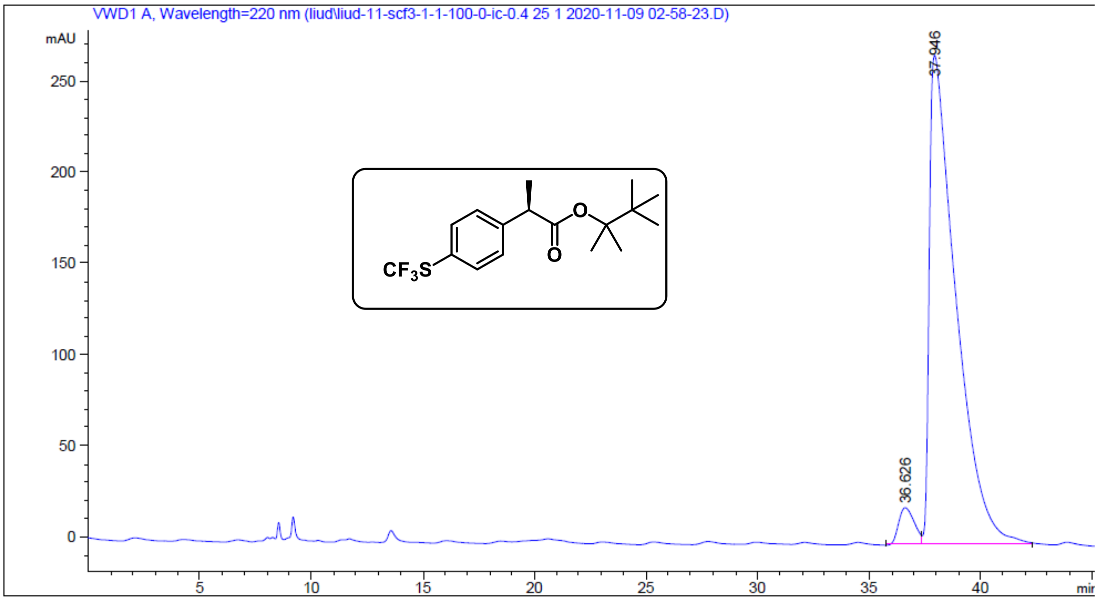

Peak	Ret Time [min]	Height [mAU]	Area %
1	36.626	19.80121	4.1354
2	37.946	269.94867	95.8646

Supplementary Figure 179. HPLC Chromatography of the Racemic 2,3,3-trimethylbutan-2-yl 2-(4-(phenylsulfonyl)phenyl)propanoate (Daicel Chiralpak AD-H Column, *n*-Hexane: *i*-PrOH = 98:2, flow rate 1.0 mL/min, T = 25 °C, λ = 254 nm**)**

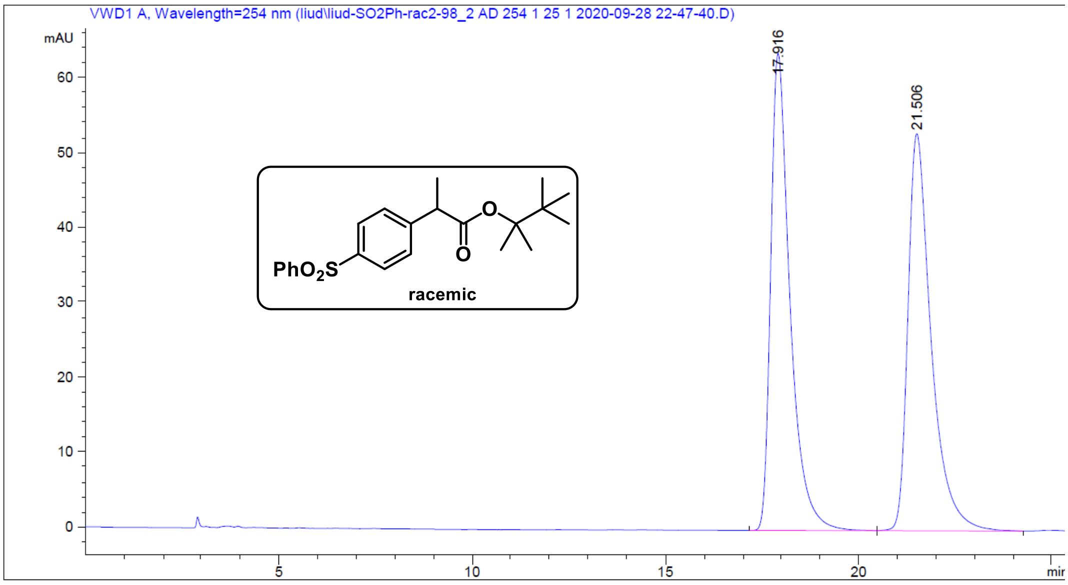

Peak	Ret Time [min]	Height [mAU]	Area %

1	17.916	63.68225	49.9498
2	21.506	53.01313	50.0502

Supplementary Figure 180. HPLC Chromatography of the 2,3,3-trimethylbutan-2-yl (*R*)-2-(4-(phenylsulfonyl)phenyl)propanoate **(**Daicel Chiralpak AD-H Column, *n*-Hexane: *i*-PrOH = 98:2, flow rate 1.0 mL/min, T = 25 °C, λ = 254 nm**)**

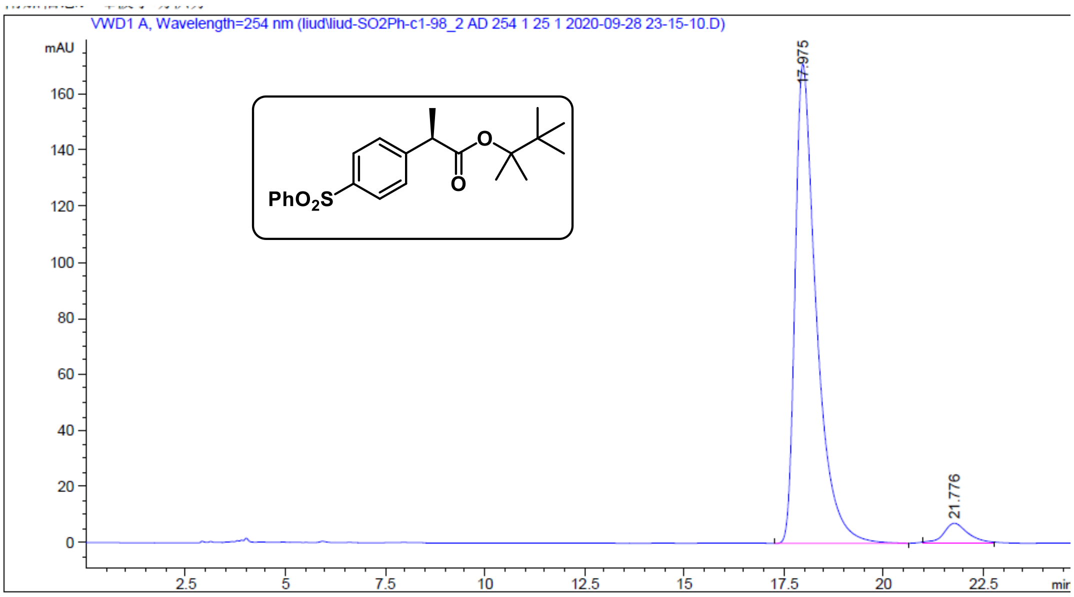

Peak	Ret Time [min]	Height [mAU]	Area %
1	17.975	170.95142	95.3153
2	21.776	7.11616	4.6847

Supplementary Figure 181. HPLC Chromatography of the Racemic 2,3,3-trimethylbutan-2-yl 2-(4-cyanophenyl)propanoate **(**Daicel Chiralpak AD-H Column, *n*-Hexane: *i*-PrOH = 98:2, flow rate 0.5 mL/min, T = 25 °C, λ = 250 nm**)**

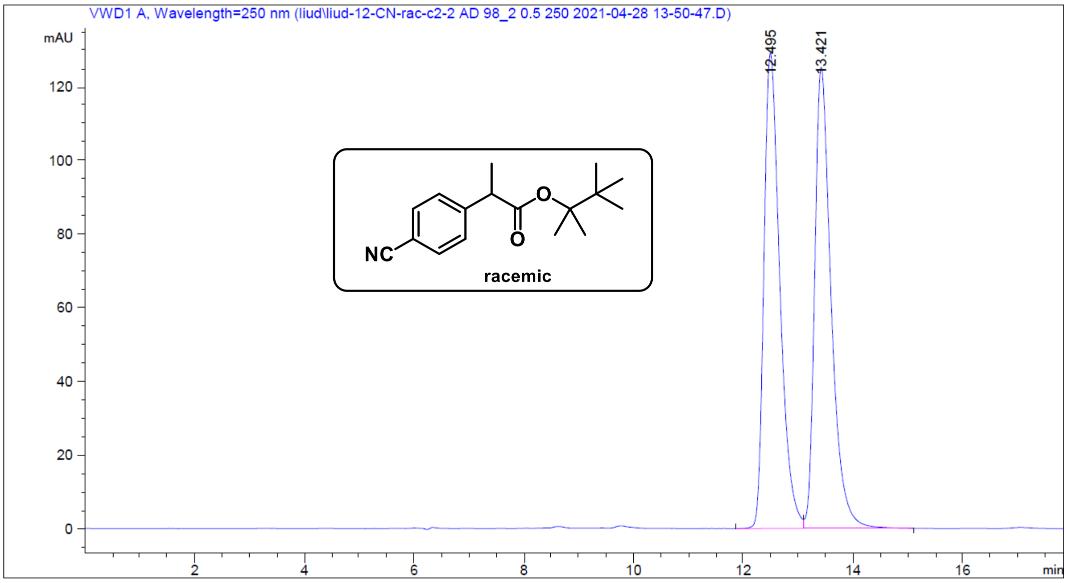

Peak	Ret Time [min]	Height [mAU]	Area %
1	12.495	129.10062	49.9988
2	13.421	125.25349	50.0012

Supplementary Figure 182. HPLC Chromatography of the 2,3,3-trimethylbutan-2-yl (*R*)-2-(4-cyanophenyl)propanoate **(**Daicel Chiralpak AD-H Column, *n*-Hexane: *i*-PrOH = 98:2, flow rate 0.5 mL/min, T = 25 °C, λ = 250 nm**)**

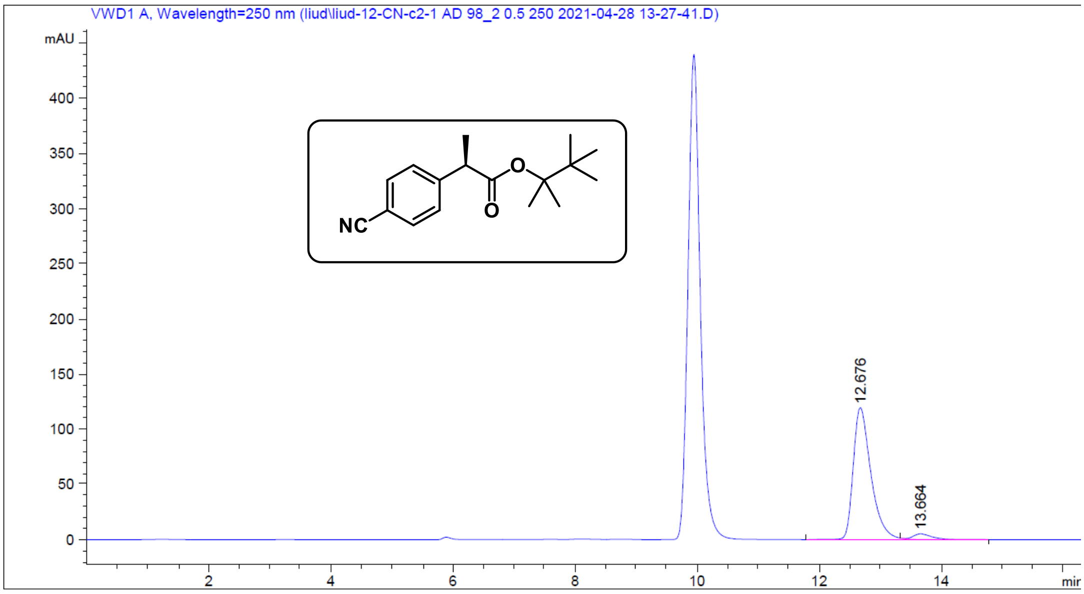

Peak	Ret Time [min]	Height [mAU]	Area %
1	12.676	119.52631	95.7249
2	13.664	5.08006	4.2751

Supplementary Figure 183. HPLC Chromatography of the Racemic 2,3,3-trimethylbutan-2-yl 2-(4-((tert-butoxycarbonyl)amino)phenyl)propanoate (Daicel Chiralpak AD-H Column, *n*-Hexane: *i*-PrOH = 98:2, flow rate 1.0 mL/min, T = 25 °C, λ = 220 nm)

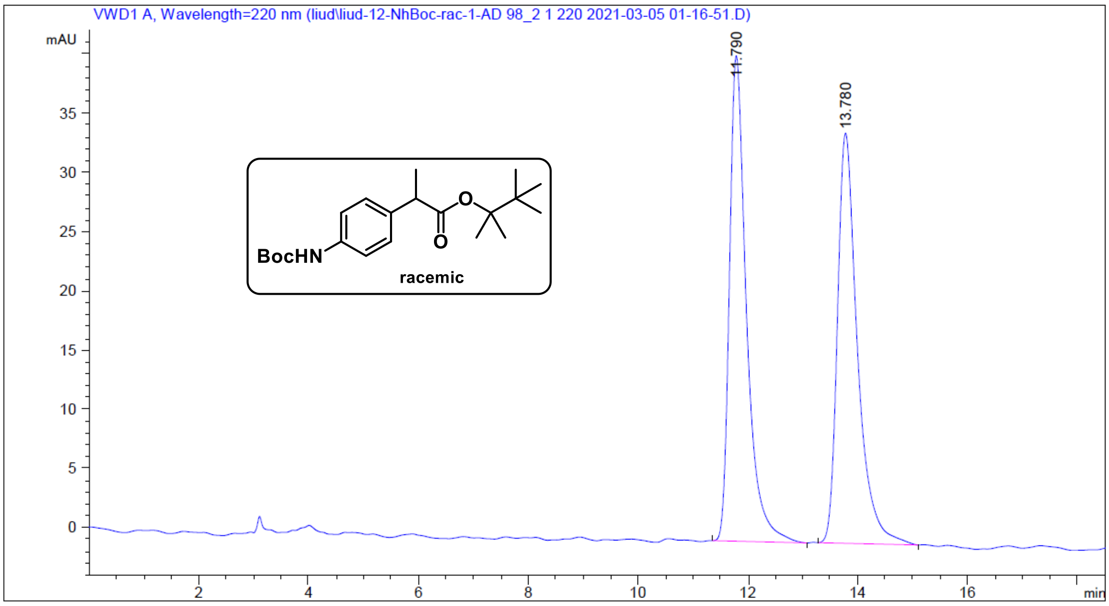

Peak	Ret Time [min]	Height [mAU]	Area %
1	11.790	41.01945	50.2653
2	13.780	34.64718	49.7347

Supplementary Figure 184. HPLC Chromatography of the 2,3,3-trimethylbutan-2-yl (*R*)-2-(4-((tert-butoxycarbonyl)amino)phenyl)propanoate (Daicel Chiralpak AD-H Column, *n*-Hexane: *i*-PrOH = 98:2, flow rate 1.0 mL/min, T = 25 °C, λ = 220 nm)

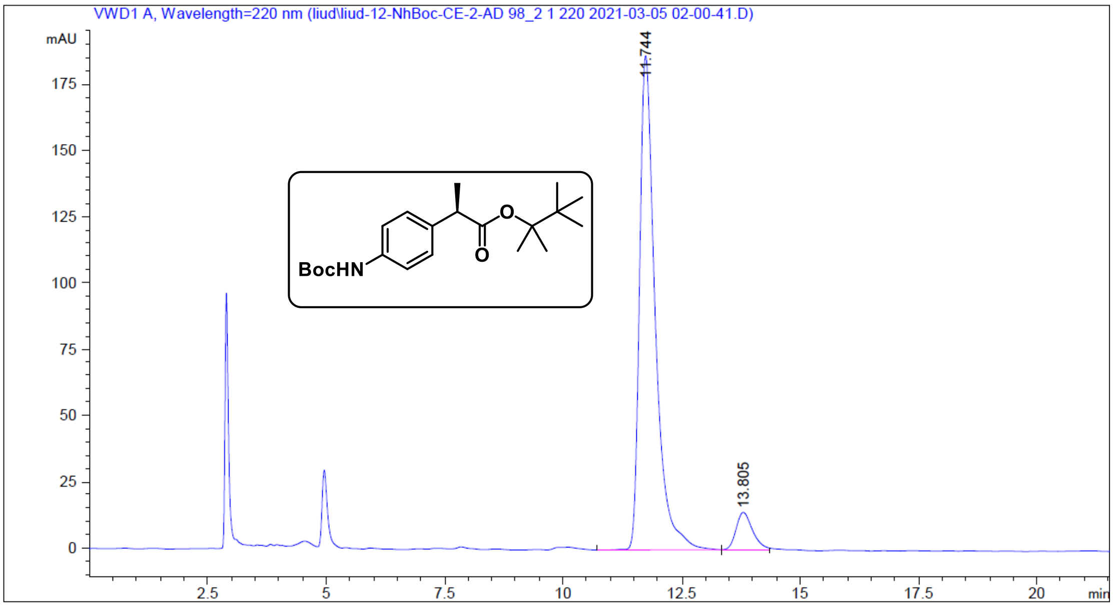

Peak	Ret Time [min]	Height [mAU]	Area %
1	11.744	186.3952	92.6492

2	13.805	14.07713	7.3508

Supplementary Figure 185. HPLC Chromatography of the racemic 2,3,3-trimethylbutan-2-yl 2-(4-acetamidophenyl)propanoate

(Daicel Chiralpak AD-H Column, *n*-Hexane: *i*-PrOH = 98:2, flow rate 0.6 mL/min, T = 25 °C, λ = 254 nm)

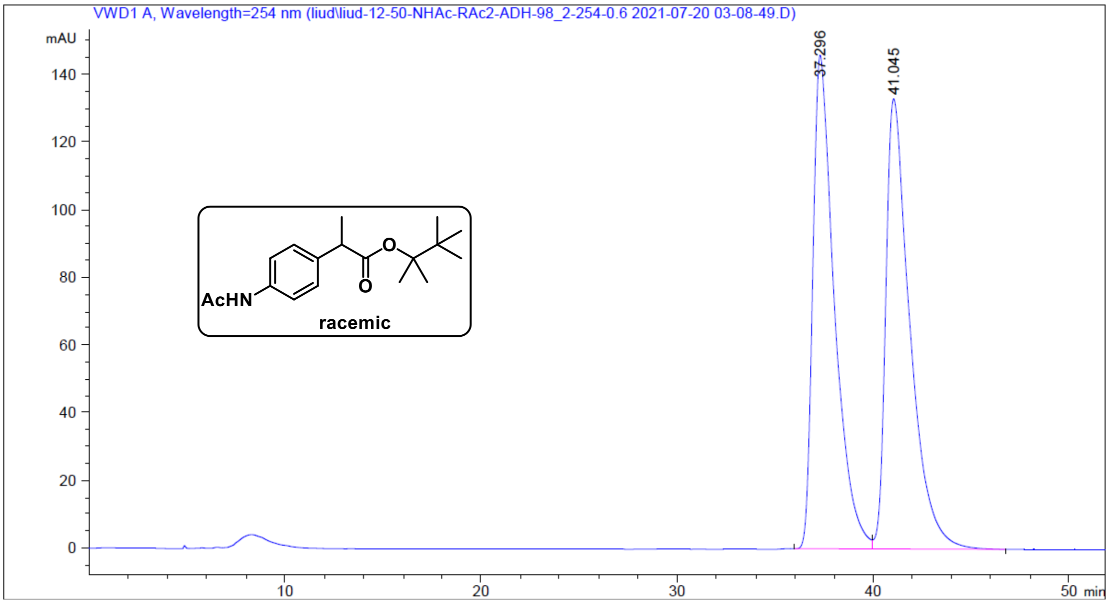

Peak	Ret Time [min]	Height [mAU]	Area %
1	37.296	145.98878	49.5205
2	41.045	133.10606	50.4795

Supplementary Figure 186. HPLC Chromatography of the 2,3,3-trimethylbutan-2-yl (*R*)-2-(4-acetamidophenyl)propanoate

(Daicel Chiralpak AD-H Column, *n*-Hexane: *i*-PrOH = 98:2, flow rate 0.6 mL/min, T = 25 °C, λ = 254 nm)

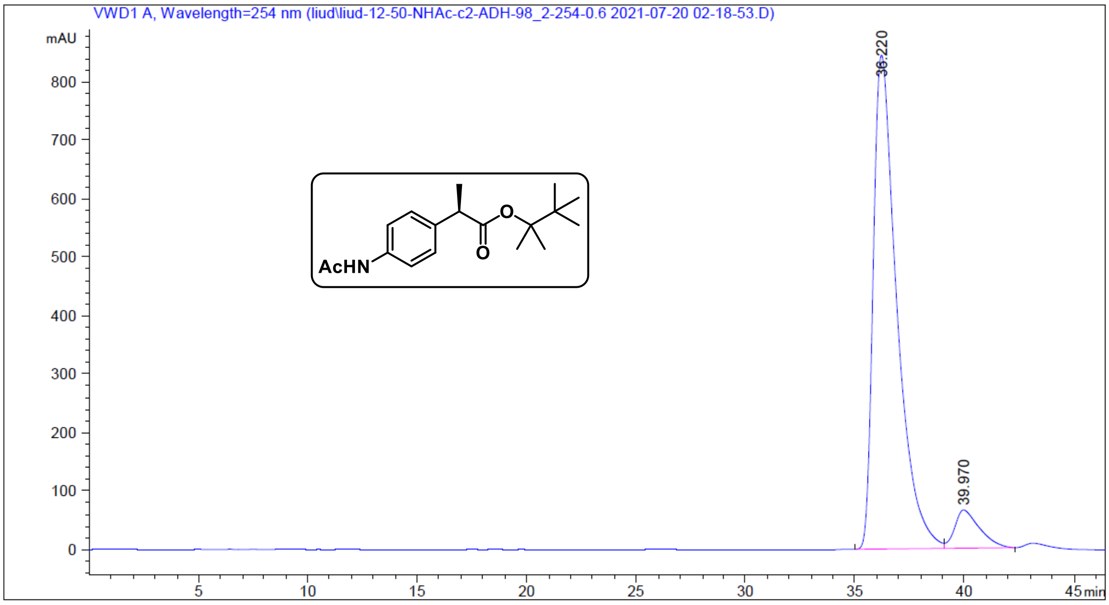

Peak	Ret Time [min]	Height [mAU]	Area %
1	36.220	844.55066	92.4640
2	39.970	65.45313	7.5360

Supplementary Figure 187. HPLC Chromatography of the Racemic 2,3,3-trimethylbutan-2-yl 2-(4-(2-oxopyrrolidin-1-yl)phenyl)propanoate (Daicel Chiralpak AD-H Column, *n*-Hexane: *i*-PrOH = 90:10, flow rate 1.0 mL/min, T = 25 °C, λ = 254 nm)

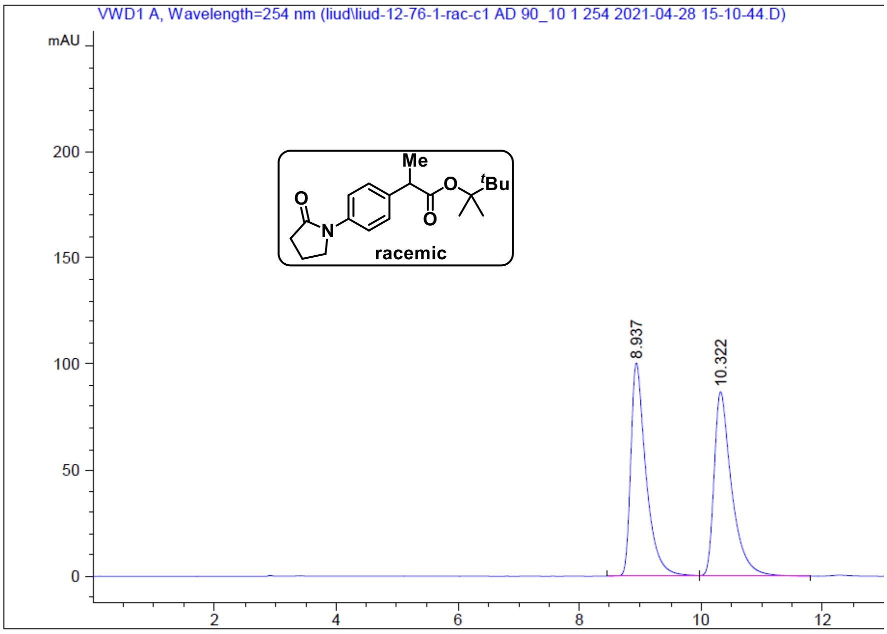

Peak	Ret Time [min]	Height [mAU]	Area %
1	8.937	100.42989	49.9866
2	10.322	86.86388	50.0134

Supplementary Figure 188. HPLC Chromatography of the 2,3,3-trimethylbutan-2-yl (*R*)-2-(4-(2-oxopyrrolidin-1-yl)phenyl)propanoate (Daicel Chiralpak AD-H Column, *n*-Hexane: *i*-PrOH = 90:10, flow rate 1.0 mL/min, T = 25 °C, λ = 254 nm)

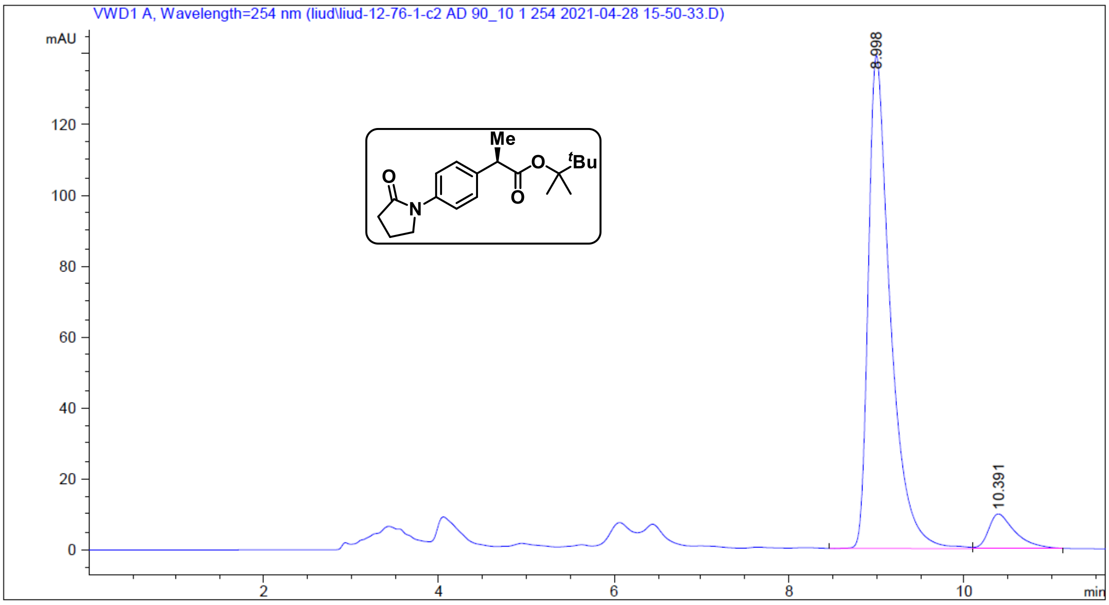

Peak	Ret Time [min]	Height [mAU]	Area %
1	8.998	139.11676	92.1328
2	10.391	9.79229	7.8672

Supplementary Figure 189. HPLC Chromatography of the Racemic 2,3,3-trimethylbutan-2-yl 2-(4-((trimethylsilyl)ethynyl)phenyl)propanoate

(Daicel Chiralpak IG-H Column, *n*-Hexane: *i*-PrOH = 99.5:0.5, flow rate 0.3 mL/min, T = 25 °C, λ = 214 nm)

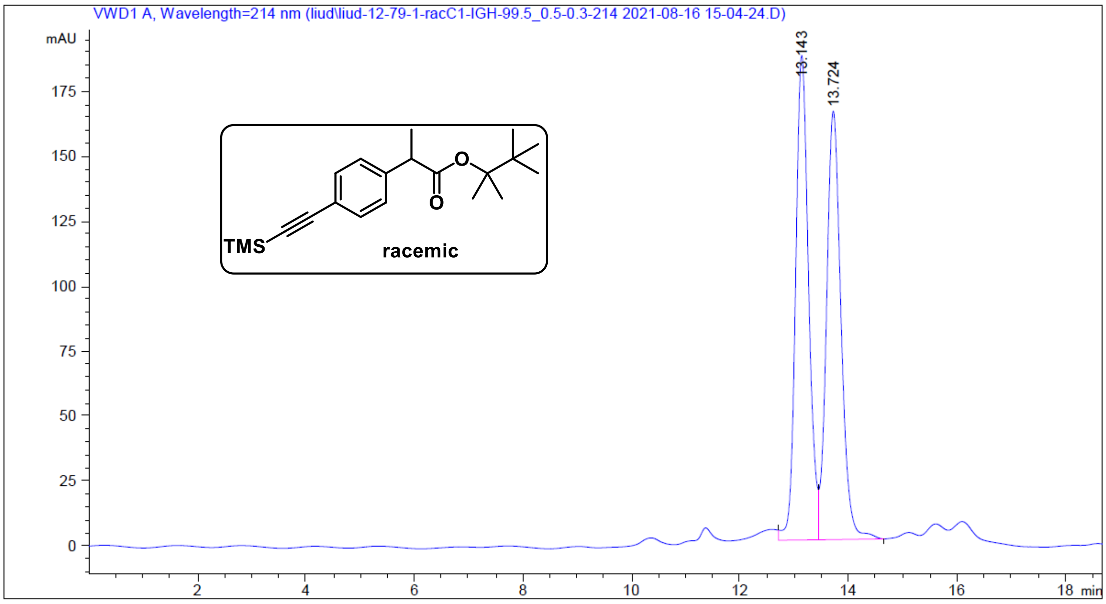

Peak	Ret Time [min]	Height [mAU]	Area %
1	13.143	186.57332	50.3368
2	13.724	164.93050	49.6632

Supplementary Figure 190. HPLC Chromatography of the 2,3,3-trimethylbutan-2-yl (*R*)-2-(4-((trimethylsilyl)ethynyl)phenyl)propanoate

(Daicel Chiralpak IG-H Column, *n*-Hexane: *i*-PrOH = 99.5:0.5, flow rate 0.3 mL/min, T = 25 °C, λ = 214 nm)

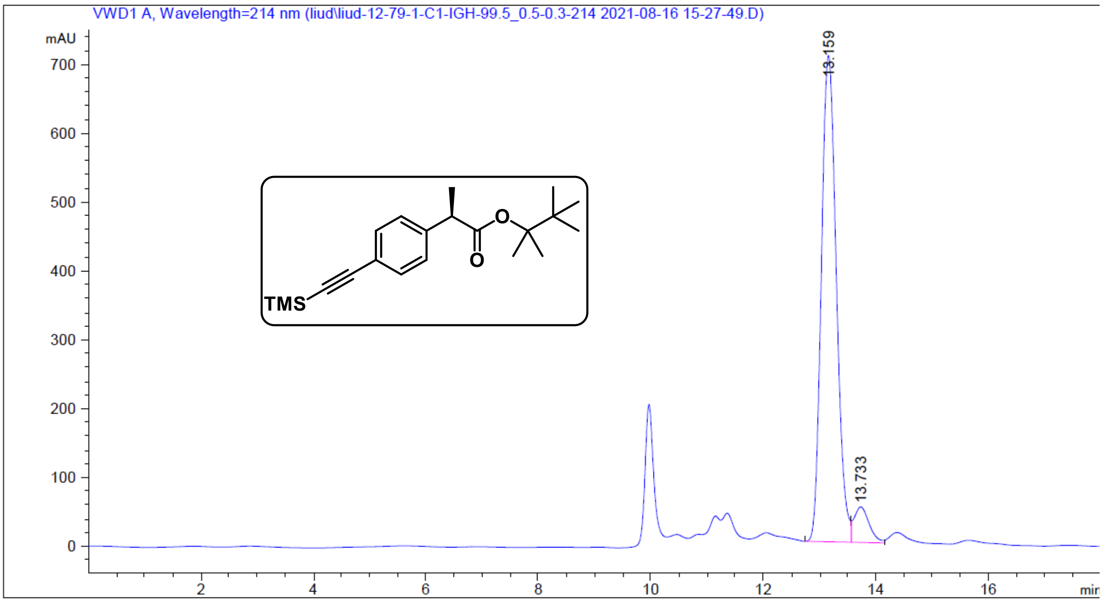

Peak	Ret Time [min]	Height [mAU]	Area %
1	13.159	706.87347	93.1100
2	13.733	51.56929	6.8900

Supplementary Figure 191. HPLC Chromatography of the Racemic 2,3,3-trimethylbutan-2-yl 2-(4-(4,4,5,5-tetramethyl-1,3,2-dioxaborolan-2-yl)phenyl)propanoate (Daicel Chiralpak IC-H Column, *n*-Hexane: *i*-PrOH = 98:2, flow rate 0.5 mL/min, T = 25 °C, λ = 214 nm)

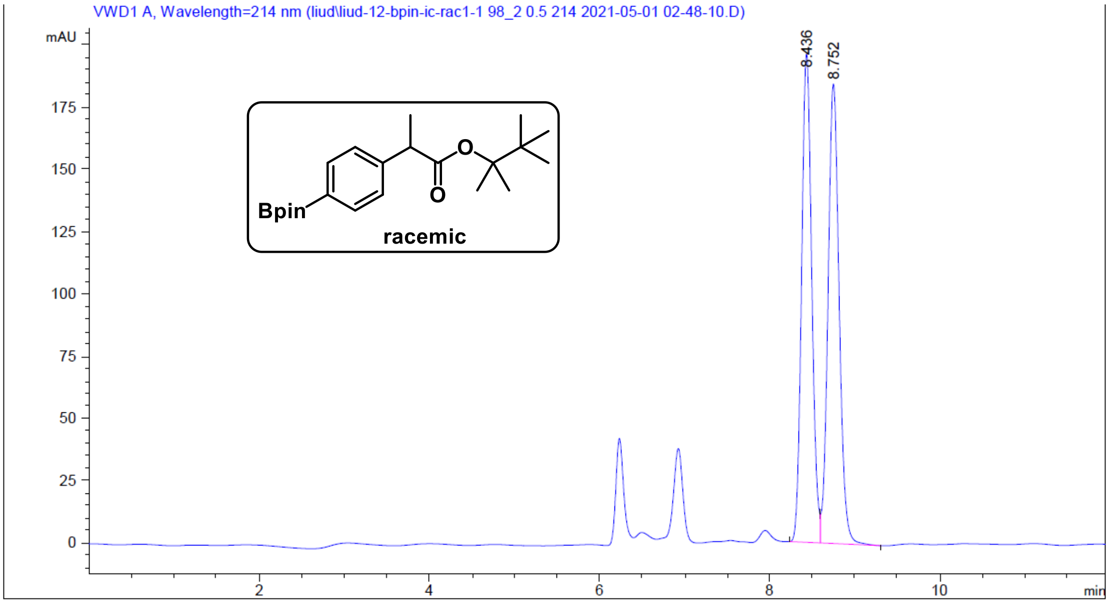

Peak	Ret Time [min]	Height [mAU]	Area %
1	8.436	196.54277	49.2095
2	28.063	184.84178	50.7905

Supplementary Figure 192. HPLC Chromatography of the 2,3,3-trimethylbutan-2-yl (*R*)-2-(4-(4,4,5,5-tetramethyl-1,3,2-dioxaborolan-2-yl)phenyl)propanoate

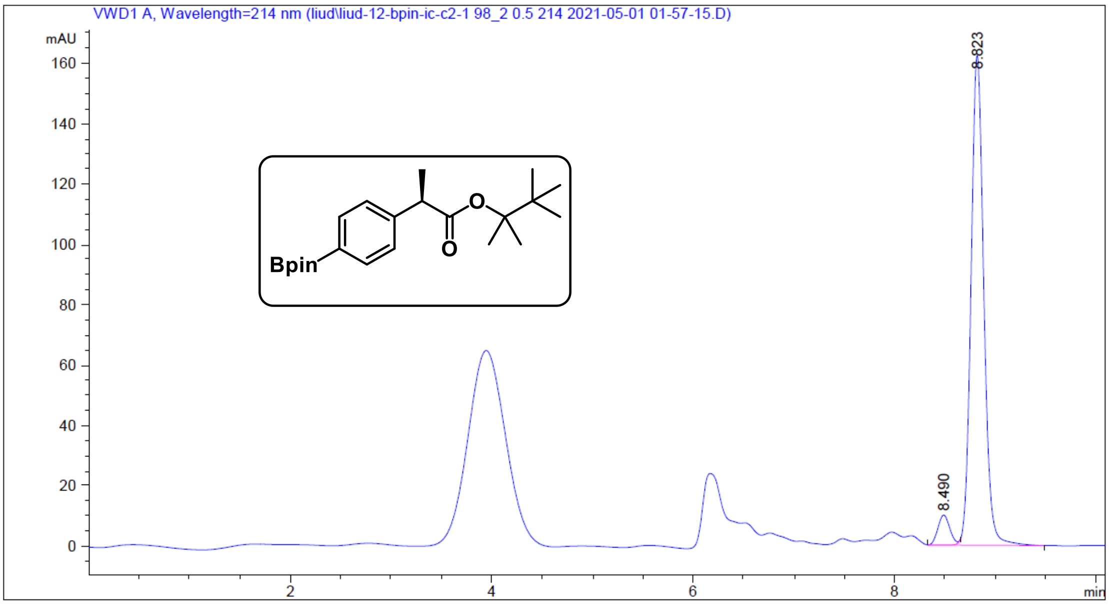

Peak	Ret Time [min]	Height [mAU]	Area %
1	8.490	9.91930	5.3656
2	8.823	162.42603	94.6344

Supplementary Figure 193. HPLC Chromatography of the Racemic 2,3,3-trimethylbutan-2-yl 2-(4-oxochroman-7-yl)propanoate

(Daicel Chiralpak AD-H Column, *n*-Hexane: *i*-PrOH = 98:2, flow rate 1.0 mL/min, T = 25 °C, λ = 220 nm)

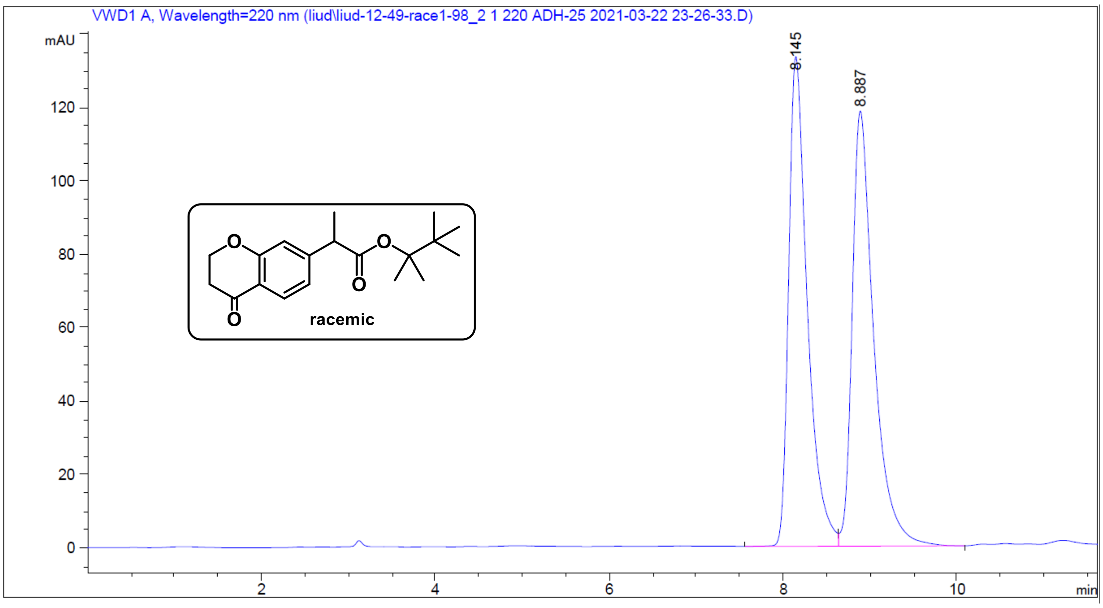

Peak	Ret Time [min]	Height [mAU]	Area %
1	8.145	133.47990	49.3821
2	8.887	118.58511	50.6179

Supplementary Figure 194. HPLC Chromatography of the 2,3,3-trimethylbutan-2-yl (*R*)-2-(4-oxochroman-7-yl)propanoate

(Daicel Chiralpak AD-H Column, *n*-Hexane: *i*-PrOH = 98:2, flow rate 1.0 mL/min, T = 25 °C, λ = 220 nm)

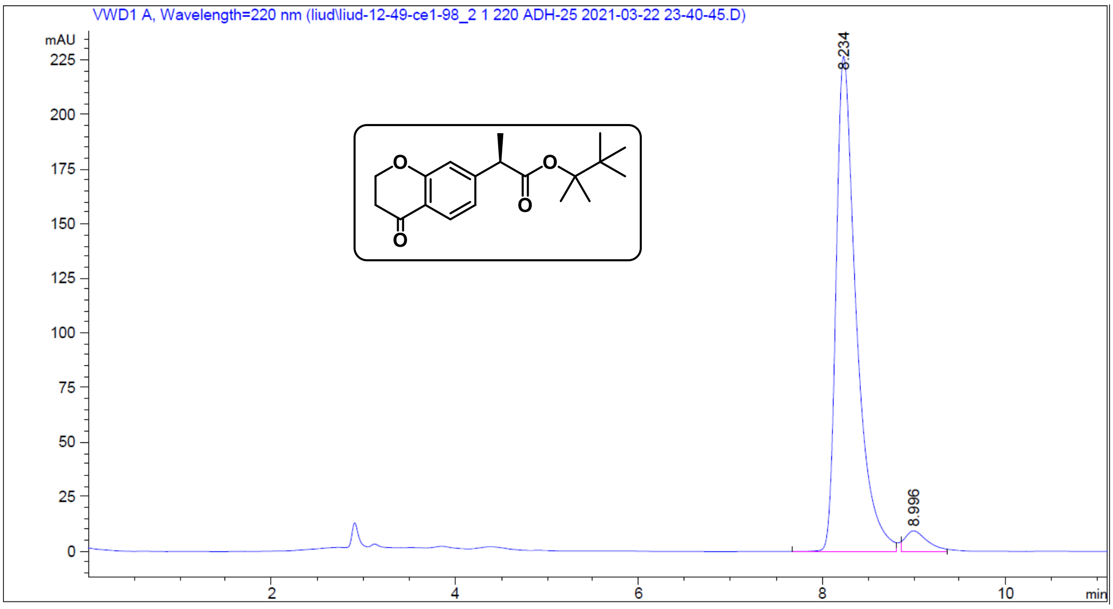

Peak	Ret Time [min]	Height [mAU]	Area %
1	8.234	226.46191	95.6397
2	8.996	9.39463	4.3603

Supplementary Figure 195. HPLC Chromatography of the Racemic ethyl 5-(1-oxo-1-((2,3,3-trimethylbutan-2-yl)oxy)propan-2-yl)benzofuran-2-carboxylate (Daicel Chiralpak IC-H Column, *n*-Hexane: *i*-PrOH = 99:1, flow rate 0.5 mL/min, T = 25 °C, λ = 220 nm)

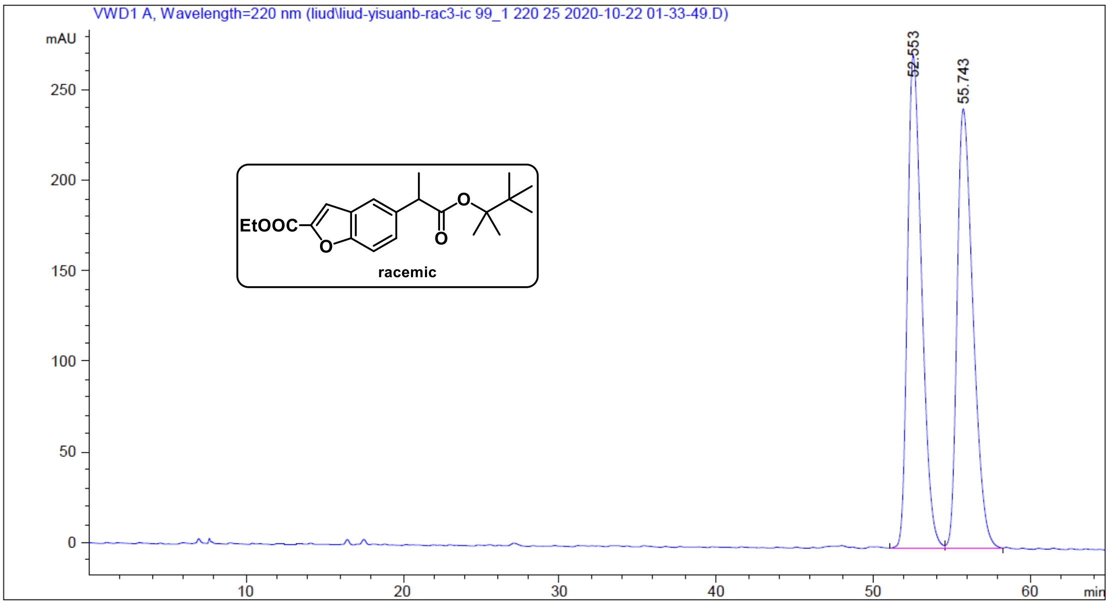

Peak	Ret Time [min]	Height [mAU]	Area %
1	52.553	272.10599	50.0842

2	55.743	242.56136	49.9158

Supplementary Figure 196. HPLC Chromatography of the Ethyl (*R*)-5-(1-oxo-1-((2,3,3-trimethylbutan-2-yl)oxy)propan-2-yl)benzofuran-2-carboxylate

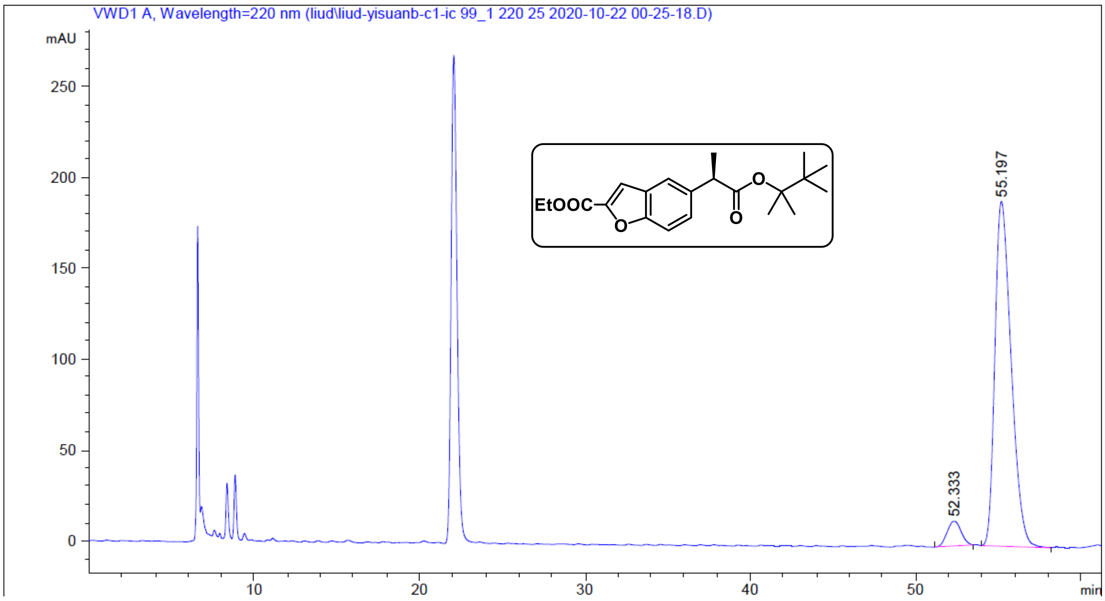

Peak	Ret Time [min]	Height [mAU]	Area %
1	52.333	13.62852	5.8082
2	55.197	189.77037	94.1918

Supplementary Figure 197. HPLC Chromatography of the Racemic 2,3,3-trimethylbutan-2-yl 2-(benzofuran-3-yl)propanoate

(Daicel Chiralpak IG-H Column, *n*-Hexane: *i*-PrOH = 99.5:0.5, flow rate 1.0 mL/min, T = 25 °C, λ = 214 nm)

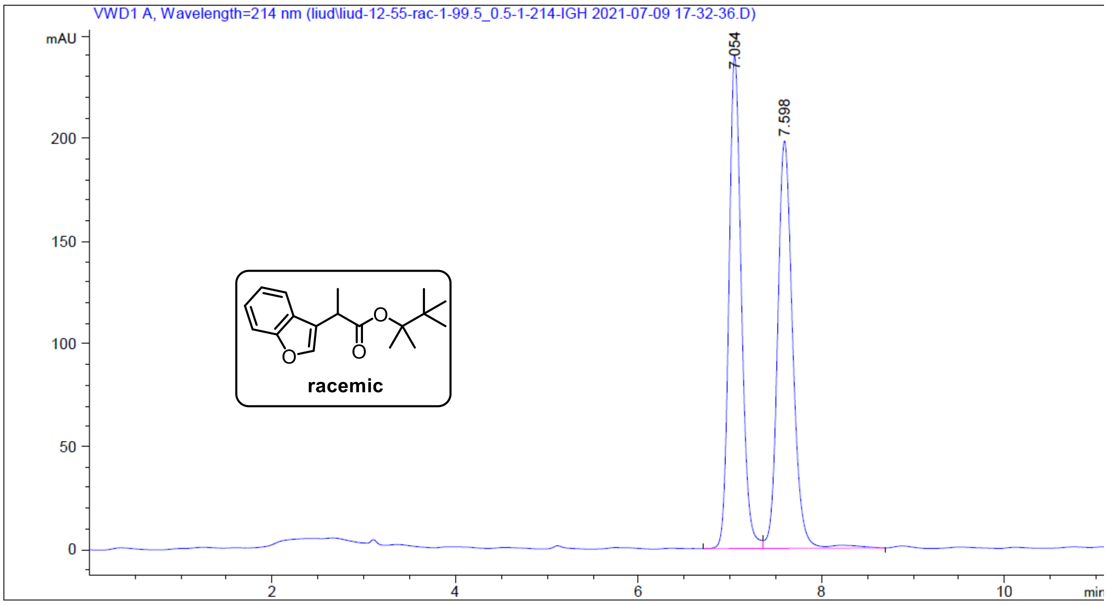

Peak	Ret Time [min]	Height [mAU]	Area %
1	7.054	239.97792	49.8399
2	7.598	198.38847	50.1601

Supplementary Figure 198. HPLC Chromatography of the 2,3,3-trimethylbutan-2-yl (*R*)-2-(benzofuran-3-yl)propanoate

(Daicel Chiralpak IG-H Column, *n*-Hexane: *i*-PrOH = 99.5:0.5, flow rate 1.0 mL/min, T = 25 °C, λ = 214 nm)

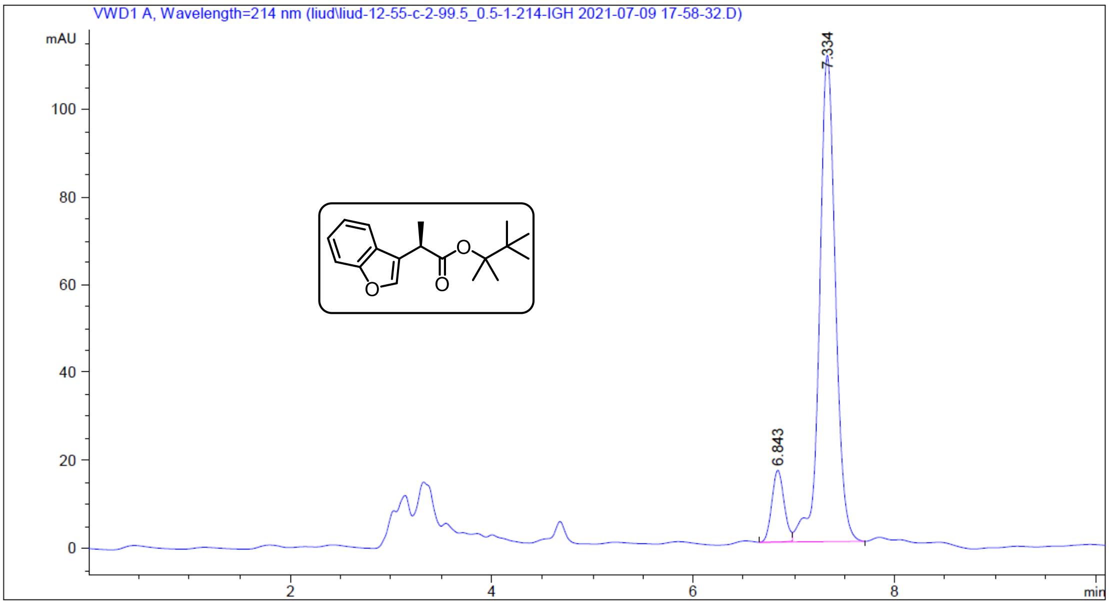

Peak	Ret Time [min]	Height [mAU]	Area %
1	6.843	16.33647	10.2167
2	7.334	110.77312	89.7833

Supplementary Figure 199. HPLC Chromatography of the Racemic 2,3,3-trimethylbutan-2-yl 2-(dibenzo[*b,d*]thiophen-3-yl)propanoate

(Daicel Chiralpak IC-H Column, *n*-Hexane: *i*-PrOH = 98:2, flow rate 1.0 mL/min, T = 25 °C, λ = 214 nm)

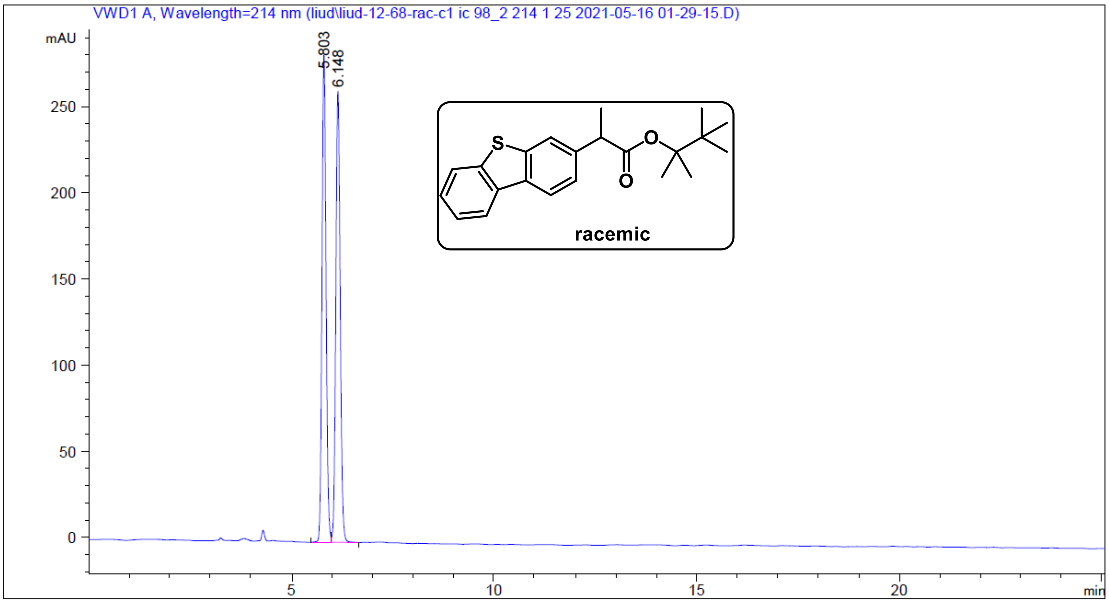

Peak	Ret Time [min]	Height [mAU]	Area %
1	5.803	282.72635	50.0004
2	6.148	261.77188	49.9996

Supplementary Figure 200. HPLC Chromatography of the 2,3,3-trimethylbutan-2-yl (*R*)-2-(dibenzo[*b,d*]thiophen-3-yl)propanoate

(Daicel Chiralpak IC-H Column, *n*-Hexane: *i*-PrOH = 98:2, flow rate 1.0 mL/min, T = 25 °C, λ = 214 nm)

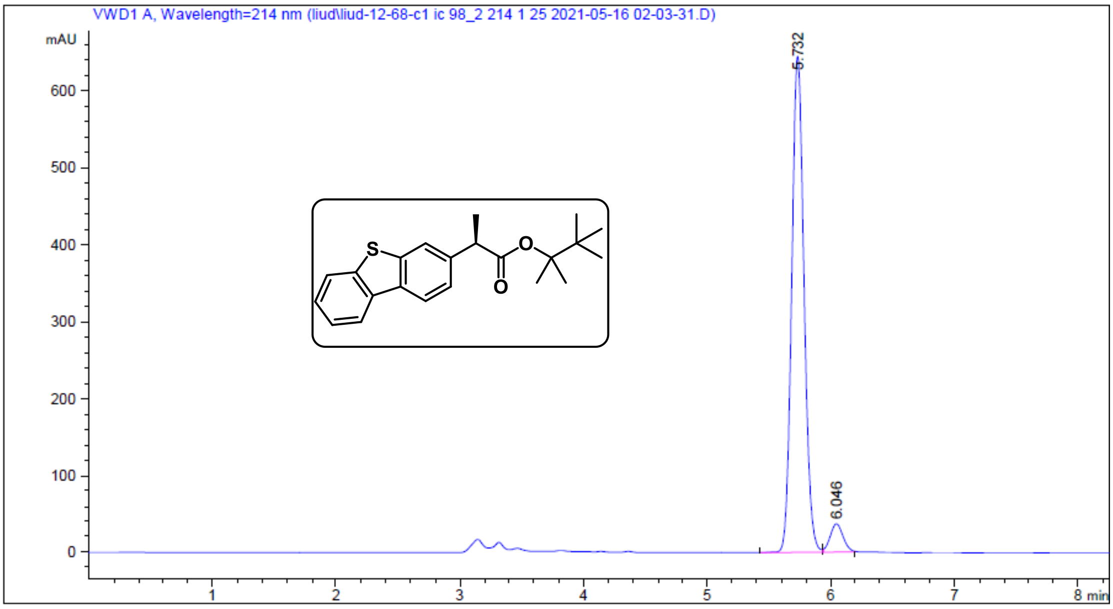

Peak	Ret Time [min]	Height [mAU]	Area %
1	5.732	645.33966	94.3323
2	6.046	36.80598	5.6677

Supplementary Figure 201. HPLC Chromatography of the Racemic 2,3,3-trimethylbutan-2-yl 2-(2-(methylthio)pyrimidin-5-yl)propanoate

(Daicel Chiralpak IC-H Column, *n*-Hexane: *i*-PrOH = 98:2, flow rate 1.0 mL/min, T = 25 °C, λ = 254 nm)

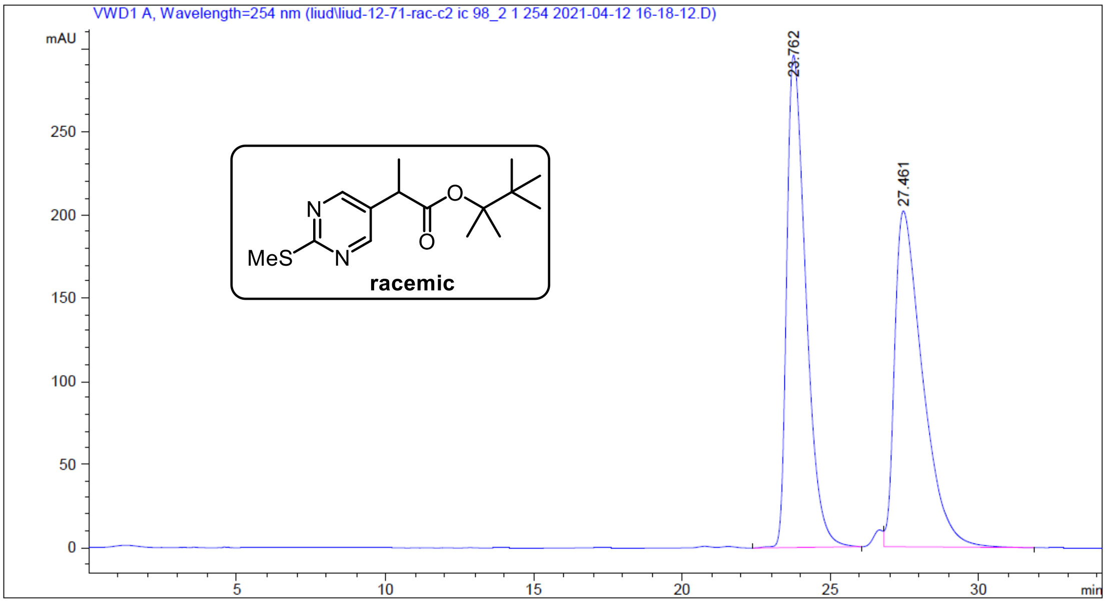

Peak	Ret Time [min]	Height [mAU]	Area %
1	23.762	296.02667	50.0593
2	27.461	201.97737	49.9407

Supplementary Figure 202. HPLC Chromatography of the 2,3,3-trimethylbutan-2-yl (*R*)-2-(2-(methylthio)pyrimidin-5-yl)propanoate

(Daicel Chiralpak IC-H Column, *n*-Hexane: *i*-PrOH = 98:2, flow rate 1.0 mL/min, T = 25 °C, λ = 254 nm)

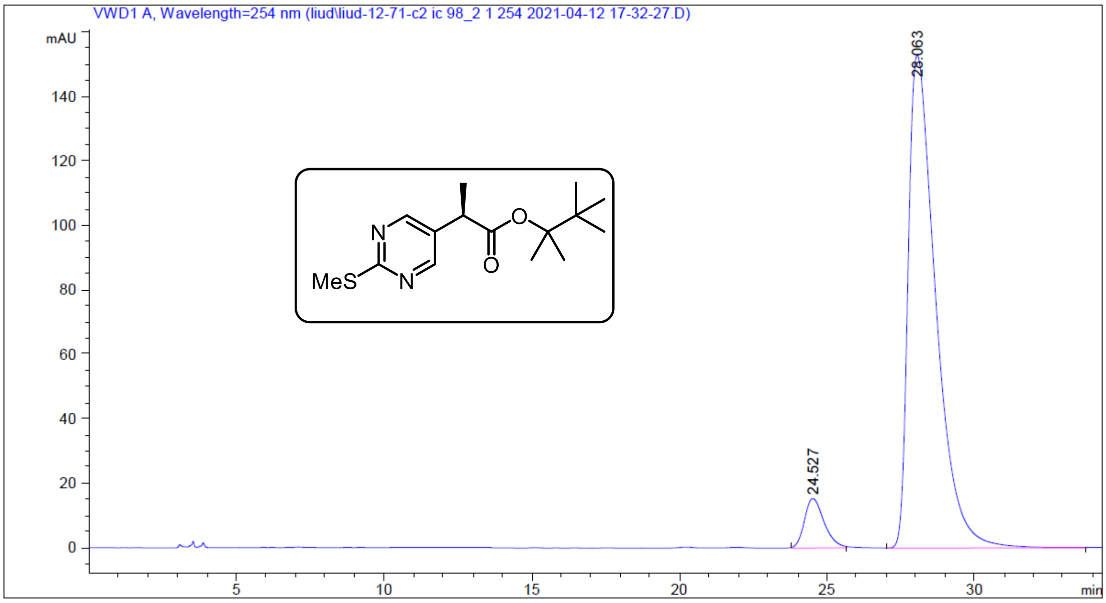

Peak	Ret Time [min]	Height [mAU]	Area %

1	24.527	15.33794	6.7110
2	28.063	152.92226	93.2890

Supplementary Figure 203. HPLC Chromatography of the Racemic 2,3,3-trimethylbutan-2-yl 2-(9*H*-fluoren-2-yl)propanoate (Daicel Chiralpak IC-H Column, *n*-Hexane: *i*-PrOH = 98:2, flow rate 1.0 mL/min, T = 25 °C, λ = 214 nm)

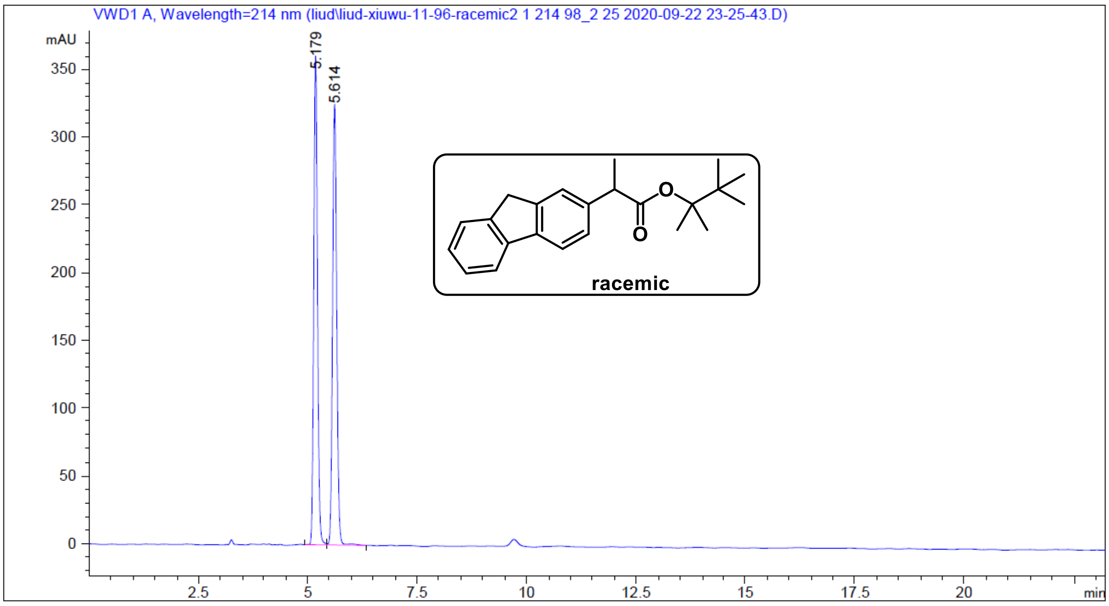

Peak	Ret Time [min]	Height [mAU]	Area %
1	5.179	359.10635	49.9204
2	5.614	322.60553	50.0796

Supplementary Figure 204. HPLC Chromatography of the 2,3,3-trimethylbutan-2-yl (*R*)-2-(9*H*-fluoren-2-yl)propanoate (Daicel Chiralpak IC-H Column, *n*-Hexane: *i*-PrOH = 98:2, flow rate 1.0 mL/min, T = 25 °C, λ = 214 nm)

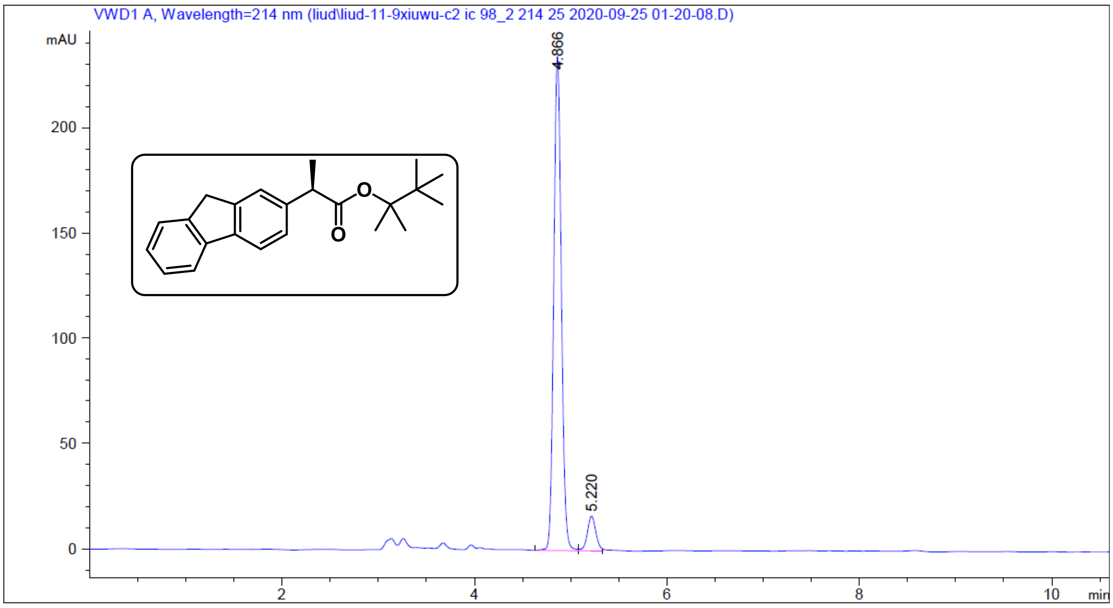

Peak	Ret Time [min]	Height [mAU]	Area %
1	4.866	232.89577	92.6693
2	5.220	16.58659	7.3307

Supplementary Figure 205. HPLC Chromatography of 2,3,3-trimethylbutan-2-yl (*E*)-4-(4-methoxyphenyl)-2-methylbut-3-enoate (Daicel Chiralpak OJ-H Column, *n*-Hexane: *i*-PrOH = 98:2, flow rate 0.4 mL/min, T = 25 ℃, λ = 214 nm)

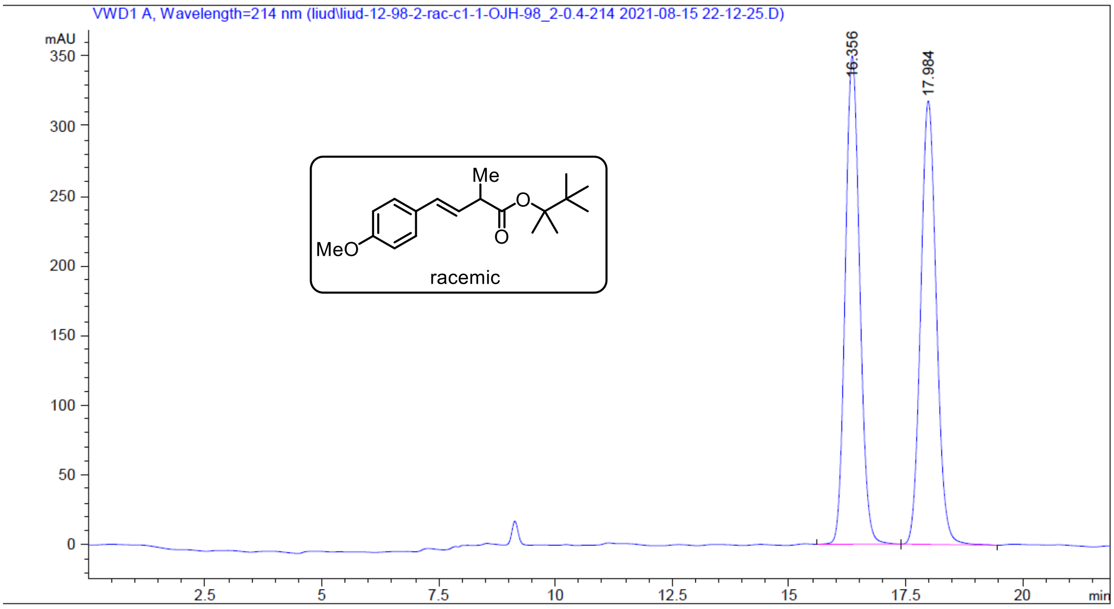

Peak	Ret Time [min]	Height [mAU]	Area %
1	16.356	350.34717	50.3296
2	17.984	318.17834	49.6704

Supplementary Figure 206. HPLC Chromatography of 2,3,3-trimethylbutan-2-yl (*R*,*E*)-4-(4-methoxyphenyl)-2-methylbut-3-enoate (400 MHz, $CDCl_3$)

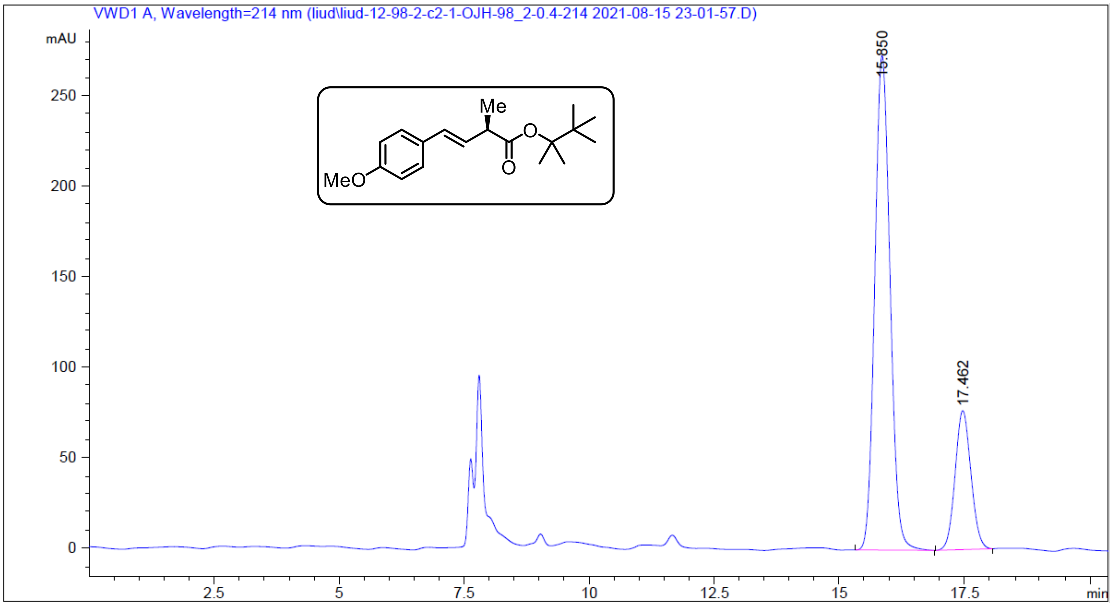

Peak	Ret Time [min]	Height [mAU]	Area %
1	15.850	273.46252	77.4467
2	17.462	76.77203	22.5533

Supplementary Figure 207. HPLC Chromatography of the (*R*)-2,5,7,8-tetramethyl-2-((4*R*,8*R*)-4,8,12-trimethyltridecyl)chroman-6-yl 4-(1-oxo-1-((2,3,3-trimethylbutan-2-yl)oxy)propan-2-yl)benzoate (Daicel Chiralpak IG-H Column, *n*-Hexane: *i*-PrOH = 98:2, flow rate 1.0 mL/min, T = 25 °C, λ = 214 nm)

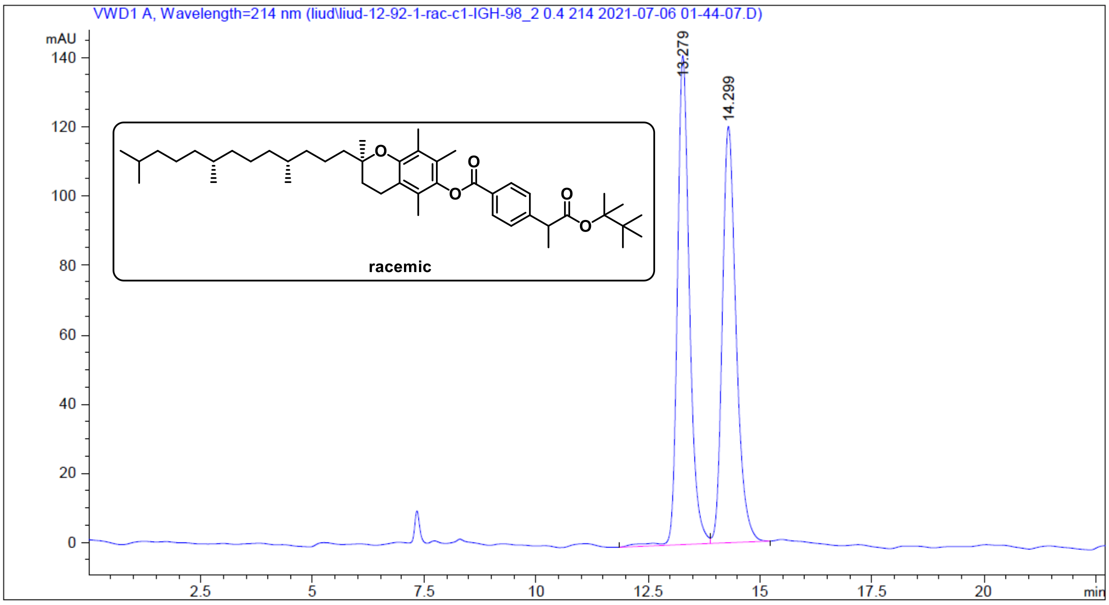

Peak	Ret Time [min]	Height [mAU]	Area %
1	13.279	141.24454	50.6866

2	14.299	120.26295	49.3134

Supplementary Figure 208. HPLC Chromatography of the (*R*)-2,5,7,8-tetramethyl-2-((4*R*,8*R*)-4,8,12-trimethyltridecyl)chroman-6-yl 4-((*R*)-1-oxo-1-((2,3,3-trimethylbutan-2-yl)oxy)propan-2-yl)benzoate

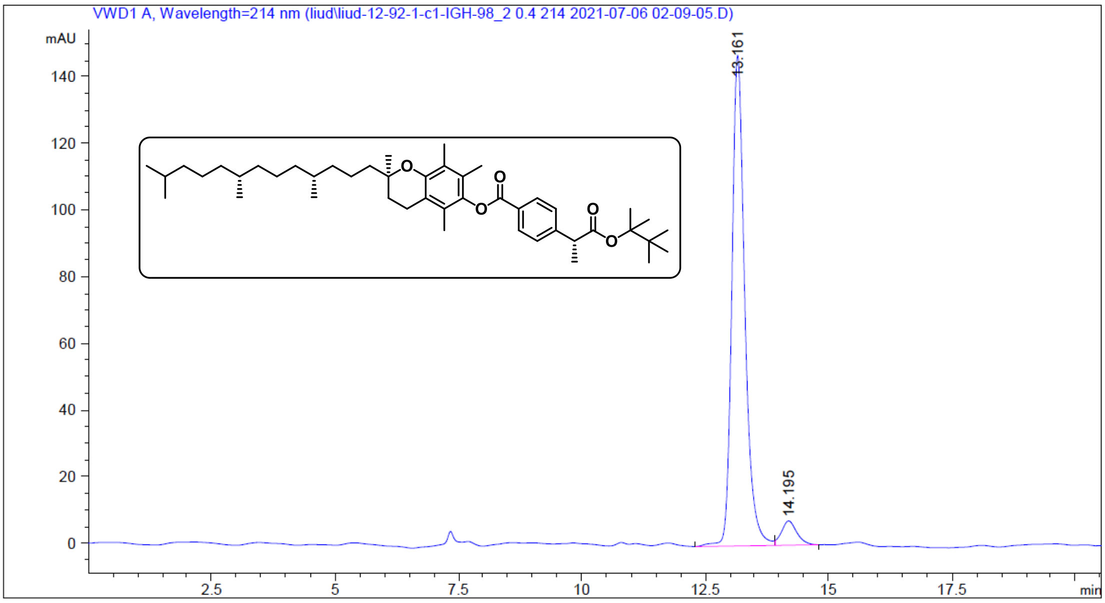

Peak	Ret Time [min]	Height [mAU]	Area %
1	13.161	147.17885	94.5396
2	14.195	7.24733	5.4604

Supplementary Figure 209. HPLC Chromatography of the Racemic 2,3,3-trimethylbutan-2-yl (*R*)-2-(3-((5-(4-fluorophenyl)thiophen-2-yl)methyl)-4-methylphenyl)propanoate (Daicel Chiralpak IG-H Column, *n*-Hexane: *i*-PrOH = 98:2, flow rate 0.4 mL/min, T = 25 °C, λ = 214 nm)

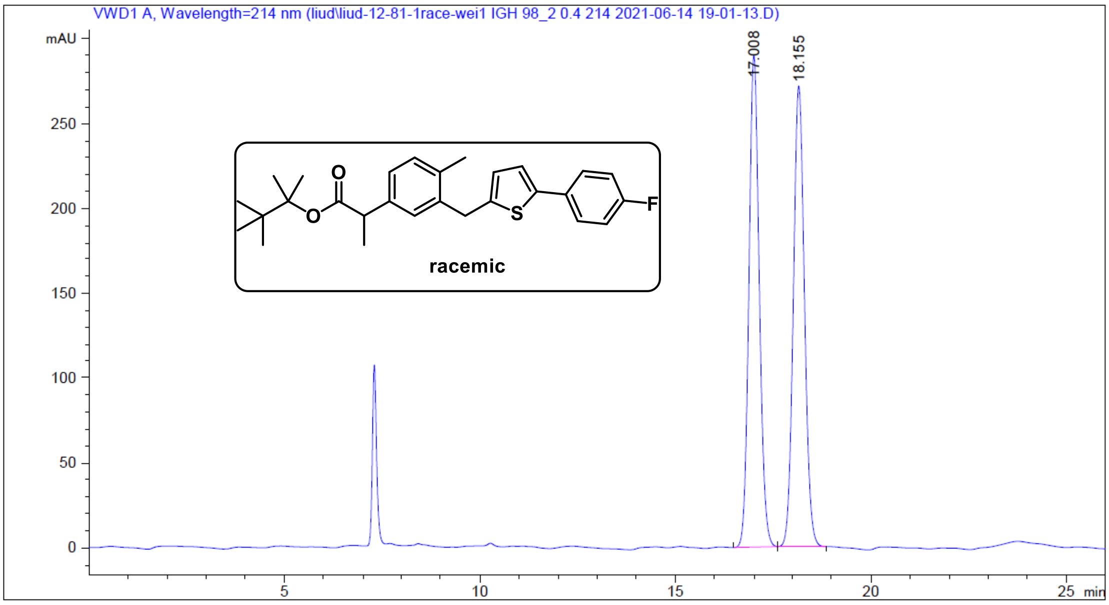

Peak	Ret Time [min]	Height [mAU]	Area %
1	17.008	290.07639	49.8822

2	18.155	271.69058	50.1178

Supplementary Figure 210. HPLC Chromatography of the 2,3,3-trimethylbutan-2-yl (*R*)-2-(3-((5-(4-fluorophenyl)thiophen-2-yl)methyl)-4-methylphenyl)propanoate

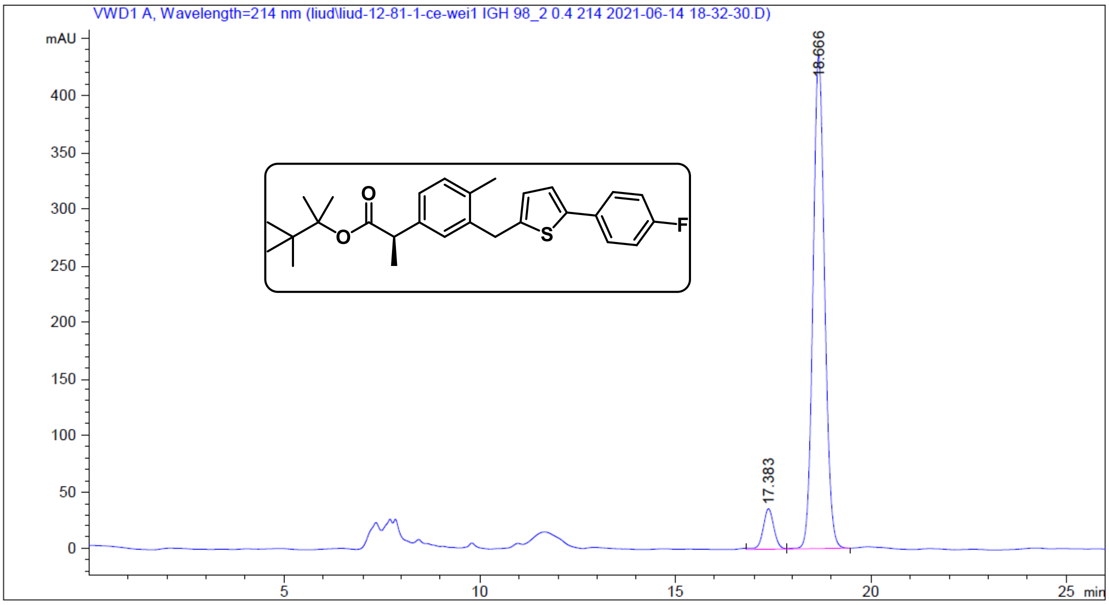

Peak	Ret Time [min]	Height [mAU]	Area %
1	17.383	35.68076	7.0600
2	18.666	435.53029	92.9400

Supplementary Figure 211. HPLC Chromatography of the Racemic 2,3,3-trimethylbutan-2-yl 2-(4-(*N*-(2,6-dimethoxypyrimidin-4-yl)-*N*-methylsulfamoyl)phenyl)propanoate (Daicel Chiralpak IC-H Column, *n*-Hexane: *i*-PrOH = 90:10, flow rate 1.0 mL/min, T = 25 °C, λ = 254 nm)

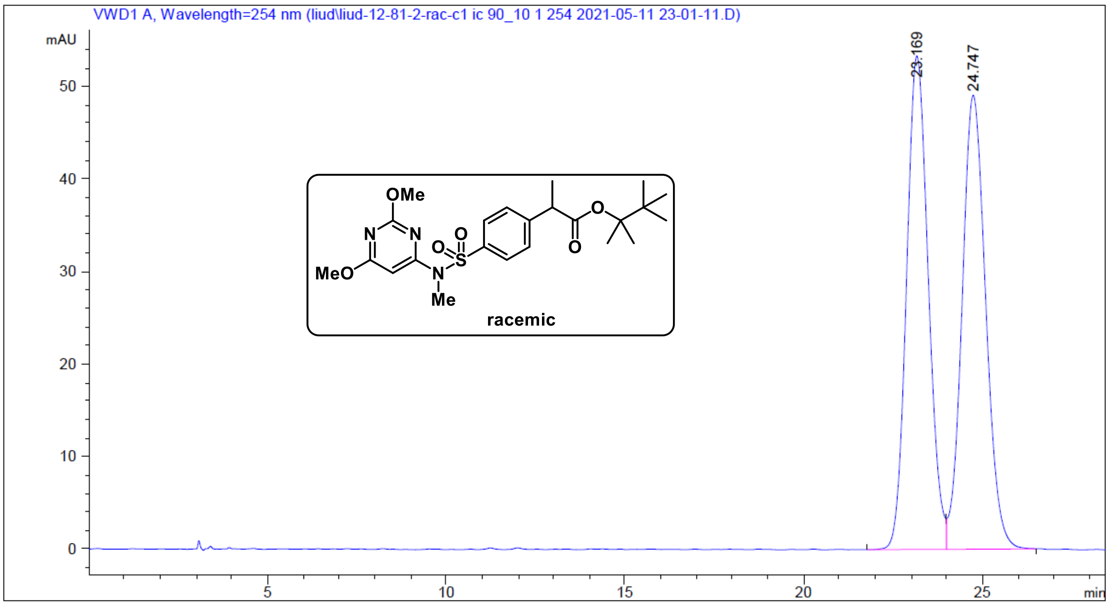

Peak	Ret Time [min]	Height [mAU]	Area %
1	23.169	53.33131	49.9769

2	24.747	49.05706	50.0231

Supplementary Figure 212. HPLC Chromatography of the 2,3,3-trimethylbutan-2-yl (*R*)-2-(4-(*N*-(2,6-dimethoxypyrimidin-4-yl)-*N*-methylsulfamoyl)phenyl)propanoate

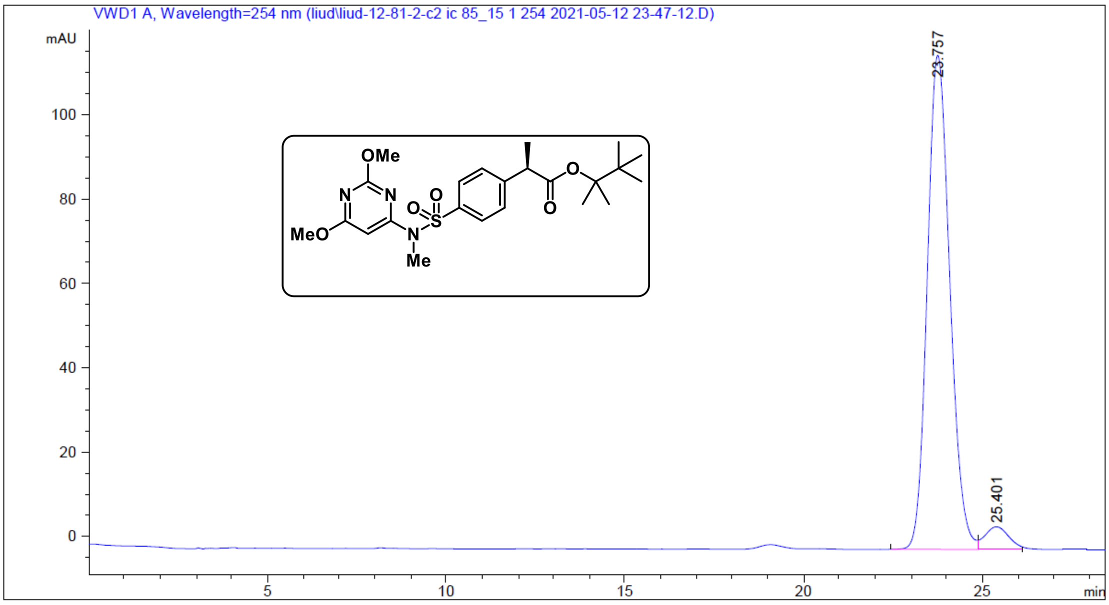

Peak	Ret Time [min]	Height [mAU]	Area %
1	23.757	117.23232	95.4055
2	25.401	5.37030	4.5945

Supplementary Figure 213. HPLC Chromatography of the Racemic 2,3,3-trimethylbutan-2-yl 2-(4-(5-methoxy-3-(2-methoxy-2-oxoethyl)-2-methyl-1*H*-indole-1-carbonyl)phenyl)propanoate (Daicel Chiralpak AD-H Column, *n*-Hexane: *i*-PrOH = 90:10, flow rate 1.0 mL/min, T = 25 °C, λ = 250 nm)

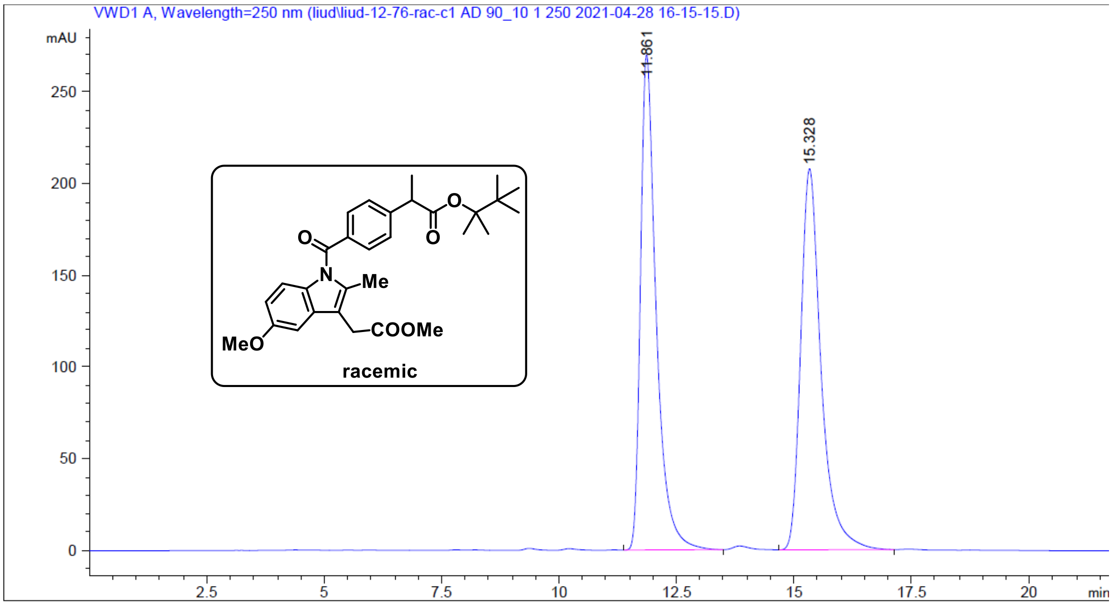

Peak	Ret Time [min]	Height [mAU]	Area %
1	11.861	269.96207	50.0148

2	15.328	207.70793	49.9852

Supplementary Figure 214. HPLC Chromatography of the 2,3,3-trimethylbutan-2-yl (*R*)-2-(4-(5-methoxy-3-(2-methoxy-2-oxoethyl)-2-methyl-1*H*-indole-1-carbonyl)phenyl)propanoate

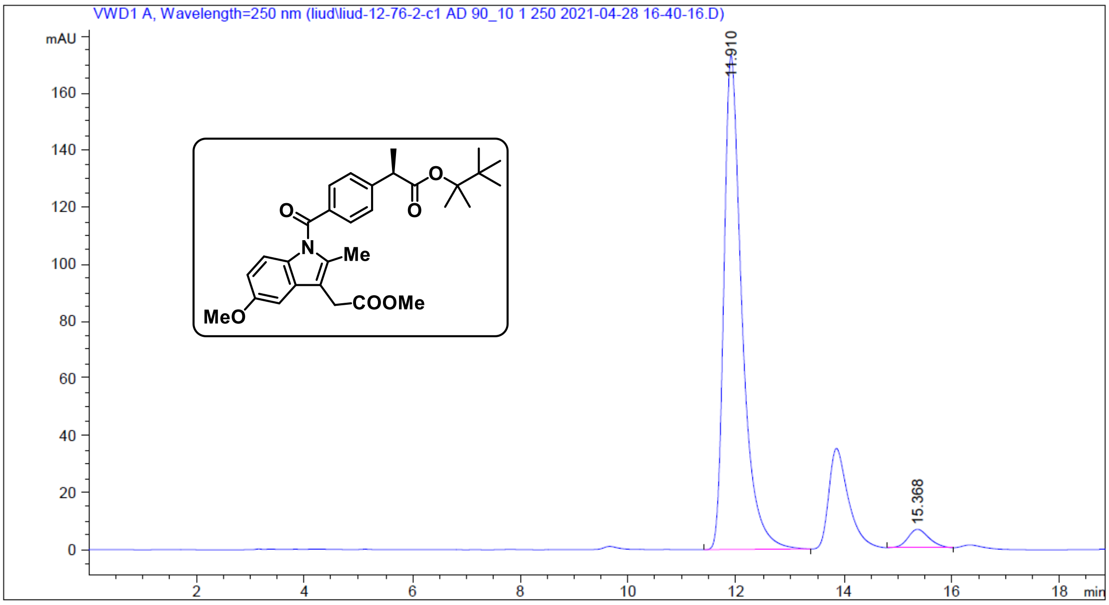

Peak	Ret Time [min]	Height [mAU]	Area %
1	11.910	173.16544	95.8361
2	15.368	6.45716	4.1639

Supplementary Figure 215. HPLC Chromatography of the (1*R*,2*S*,5*R*)-2-isopropyl-5-methylcyclohexyl 4-(1-oxo-1-((2,3,3-trimethylbutan-2-yl)oxy)propan-2-yl)benzoate (Daicel Chiralpak IG-H Column, *n*-Hexane: *i*-PrOH = 98:2, flow rate 1.0 mL/min, T = 25 °C, λ = 214 nm)

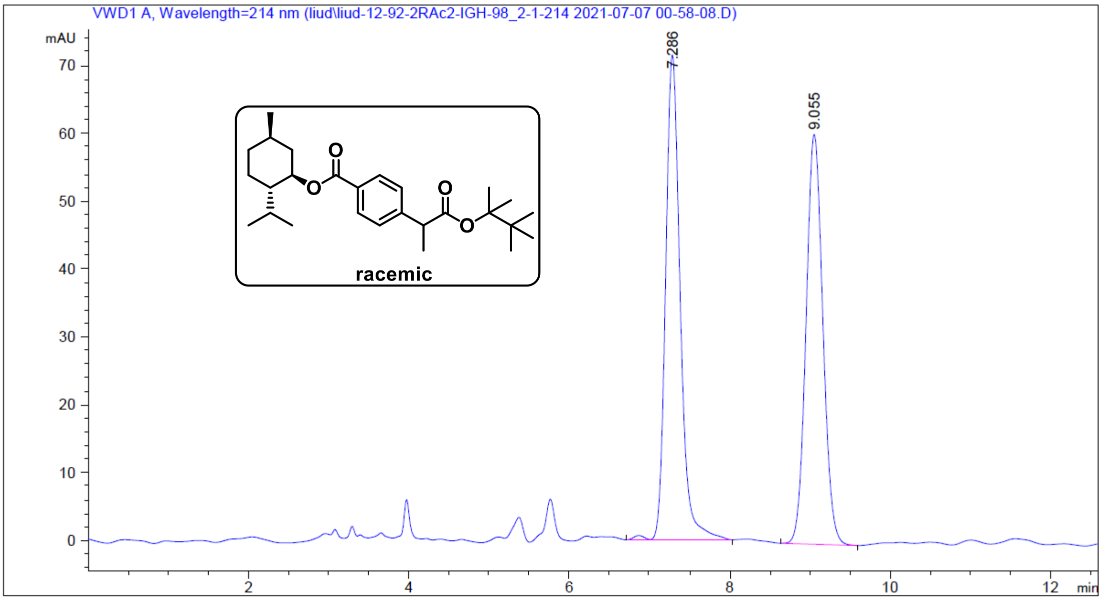

Peak	Ret Time [min]	Height [mAU]	Area %
1	7.286	71.49884	50.7532

2	9.055	60.39587	49.2468

Supplementary Figure 216. HPLC Chromatography of the (1*R*,2*S*,5*R*)-2-isopropyl-5-methylcyclohexyl 4-((*R*)-1-oxo-1-((2,3,3-trimethylbutan-2-yl)oxy)propan-2-yl)benzoate

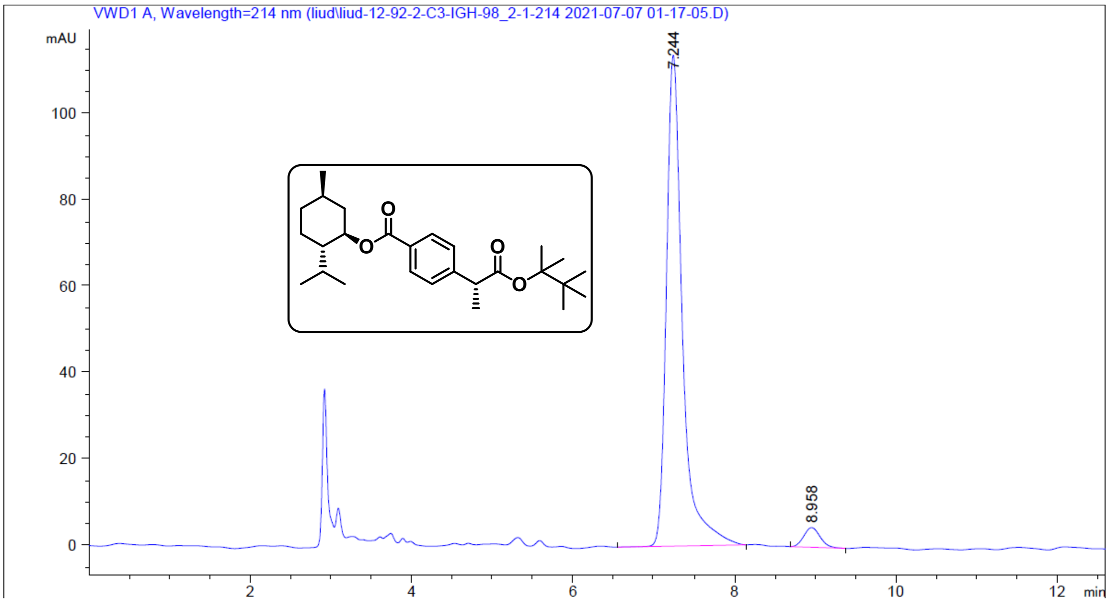

Peak	Ret Time [min]	Height [mAU]	Area %
1	7.244	113.67895	96.0668
2	8.958	4.54767	3.9332

Supplementary Figure 217. HPLC Chromatography of the (3a*R*,5*R*,6*S*,6a*R*)-5-((*R*)-2,2-dimethyl-1,3-dioxolan-4-yl)-2,2-dimethyltetrahydrofuro[2,3-*d*][1,3]dioxol-6-yl 4-(1-oxo-1-((2,3,3-trimethylbutan-2-yl)oxy)propan-2-yl)benzoate

(Daicel Chiralpak AD-H Column, *n*-Hexane: *i*-PrOH = 98:2, flow rate 0.7 mL/min, T = 25 °C, λ = 214 nm)

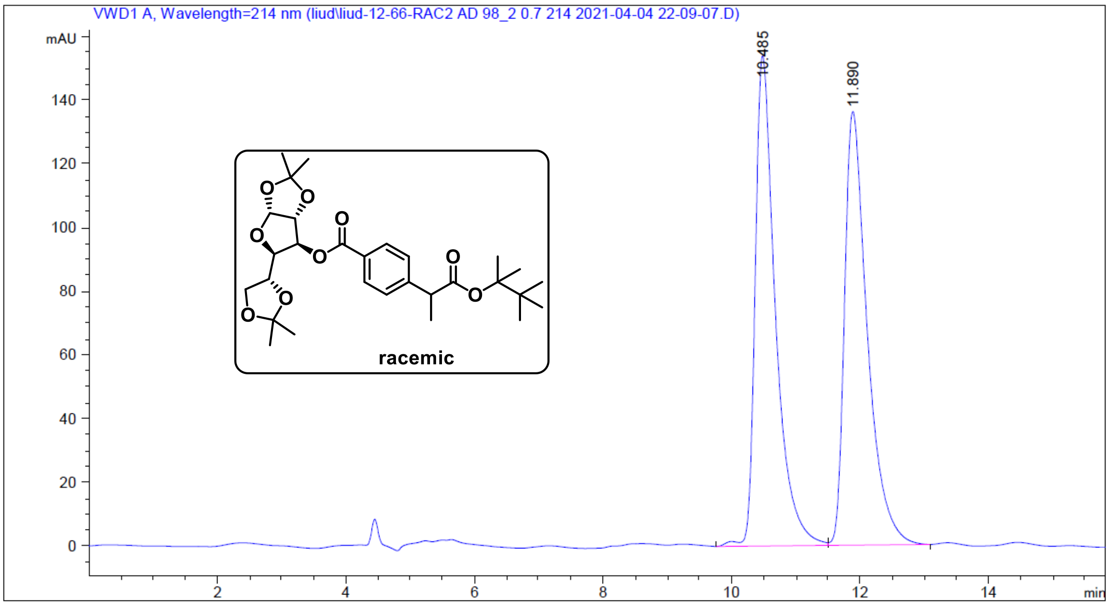

Peak	Ret Time [min]	Height [mAU]	Area %

1	10.485	154.05833	50.1728
2	11.890	136.23669	49.8272

Supplementary Figure 218. HPLC Chromatography of the (3a*R*,5*R*,6*S*,6a*R*)-5-((*R*)-2,2-dimethyl-1,3-dioxolan-4-yl)-2,2-dimethyltetrahydrofuro[2,3-*d*][1,3]dioxol-6-yl 4-((*R*)-1-oxo-1-((2,3,3-trimethylbutan-2-yl)oxy)propan-2-yl)benzoate

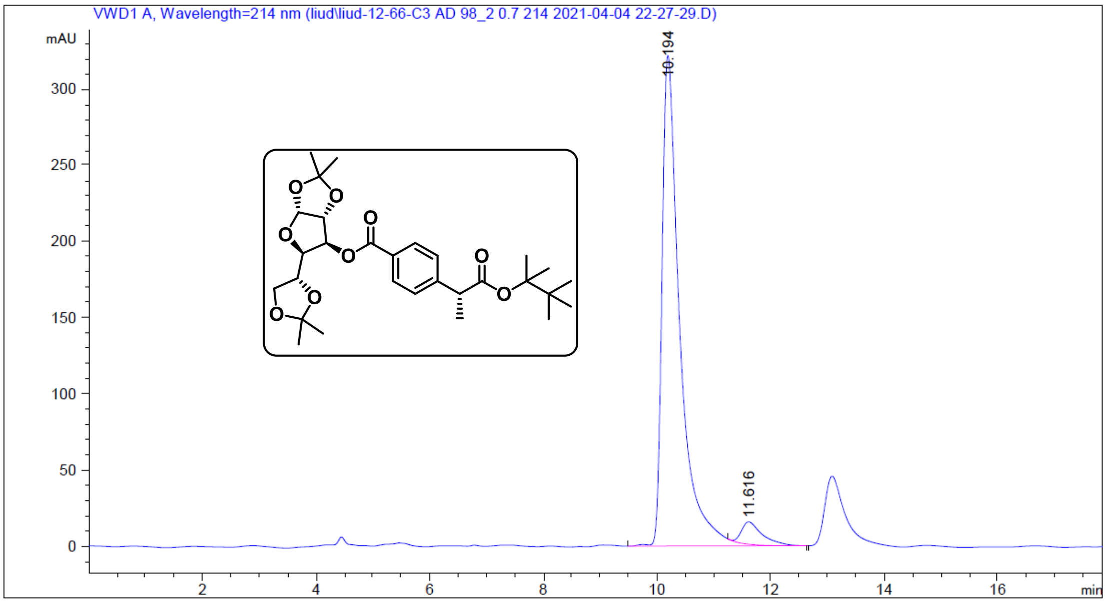

Peak	Ret Time [min]	Height [mAU]	Area %
1	10.194	321.90137	94.8951
2	11.616	14.70322	5.1049

Supplementary Figure 219. HPLC Chromatography of the Racemic 2,3,3-trimethylbutan-2-yl 2-(6-methoxynaphthalen-2-yl)propanoate (Daicel Chiralpak IC-H Column, *n*-Hexane: *i*-PrOH = 99:1, flow rate 0.5 mL/min, T = 25 °C, λ = 230 nm)

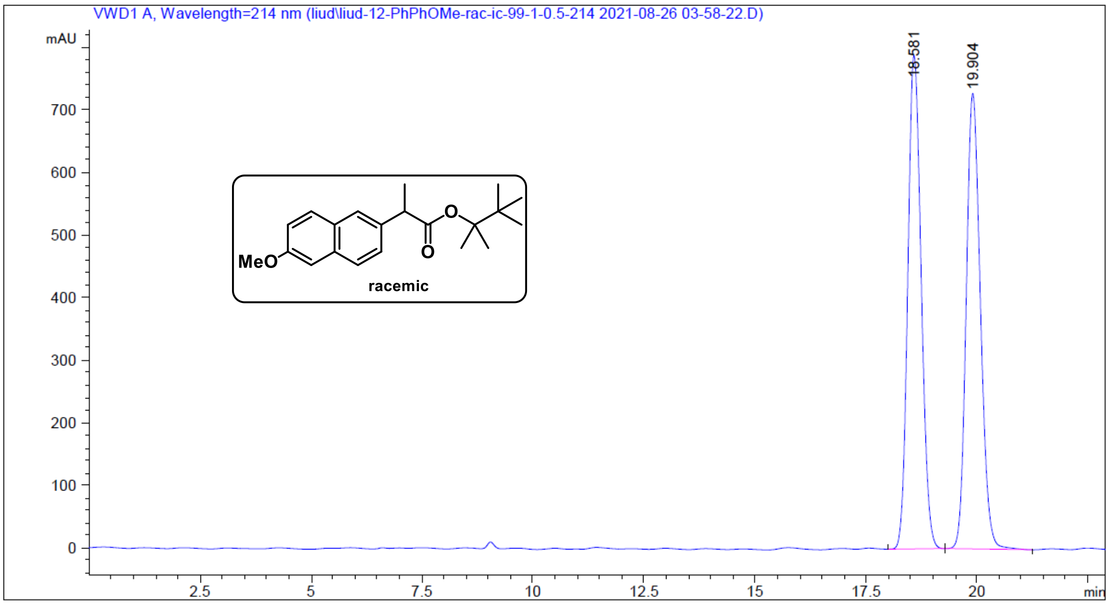

Peak	Ret Time [min]	Height [mAU]	Area %
1	18.851	788.51031	50.4583
2	19.904	728.04468	49.5417

Supplementary Figure 220. HPLC Chromatography of the 2,3,3-trimethylbutan-2-yl (*R*)-2-(6-methoxynaphthalen-2-yl)propanoate

(Daicel Chiralpak IC-H Column, *n*-Hexane: *i*-PrOH = 99:1, flow rate 0.5 mL/min, T = 25 °C, λ = 230 nm)

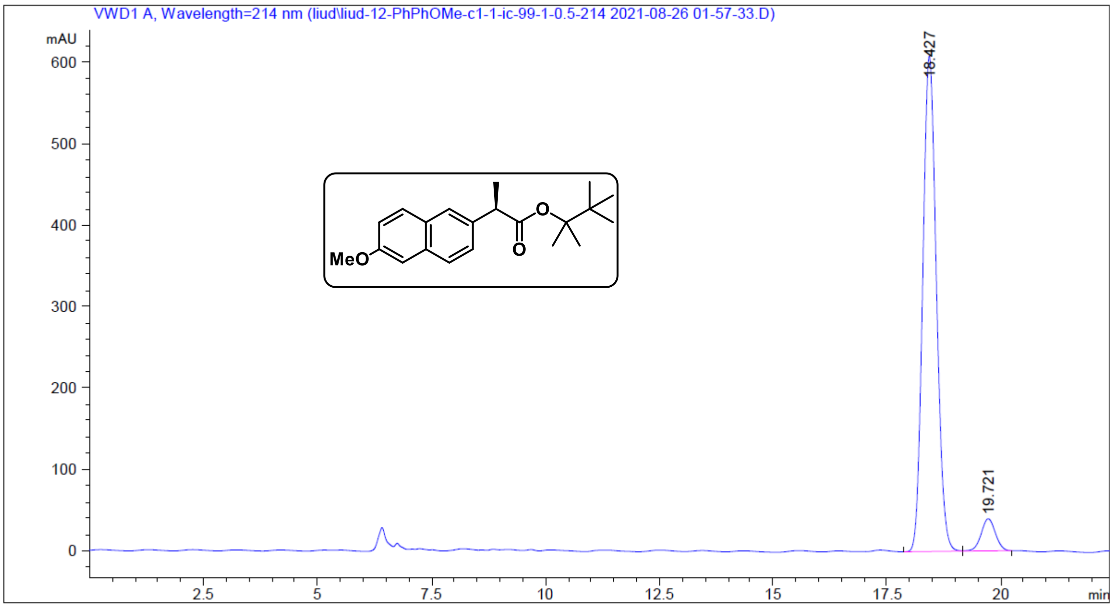

Peak	Ret Time [min]	Height [mAU]	Area %
1	18.427	609.16150	93.6668
2	19.721	39.42362	6.3332

Supplementary Figure 221. HPLC Chromatography of the Racemic 2,3,3-trimethylbutan-2-yl 2-(4-isobutylphenyl)propanoate

(Daicel Chiralpak IC-H Column, *n*-Hexane: *i*-PrOH = 98:2, flow rate 0.5 mL/min, T = 25 °C, λ = 214 nm)

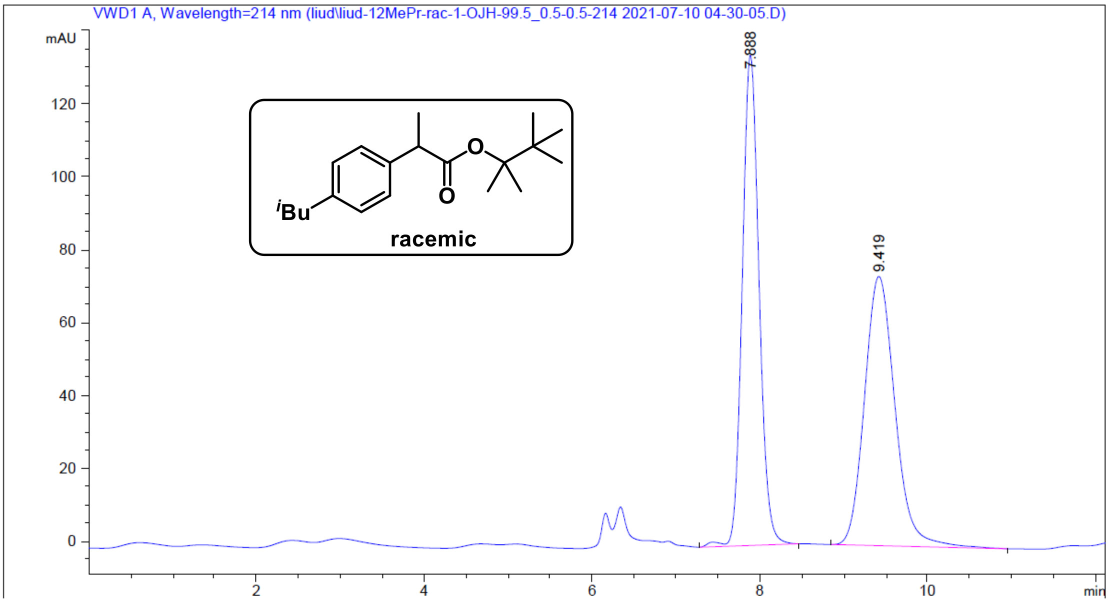

Peak	Ret Time [min]	Height [mAU]	Area %
1	7.888	134.57541	50.0766
2	9.419	73.90546	49.9234

Supplementary Figure 222. HPLC Chromatography of the 2,3,3-trimethylbutan-2-yl (*R*)-2-(4-isobutylphenyl)propanoate

(Daicel Chiralpak IC-H Column, *n*-Hexane: *i*-PrOH = 98:2, flow rate 0.5 mL/min, T = 25 °C, λ = 214 nm)

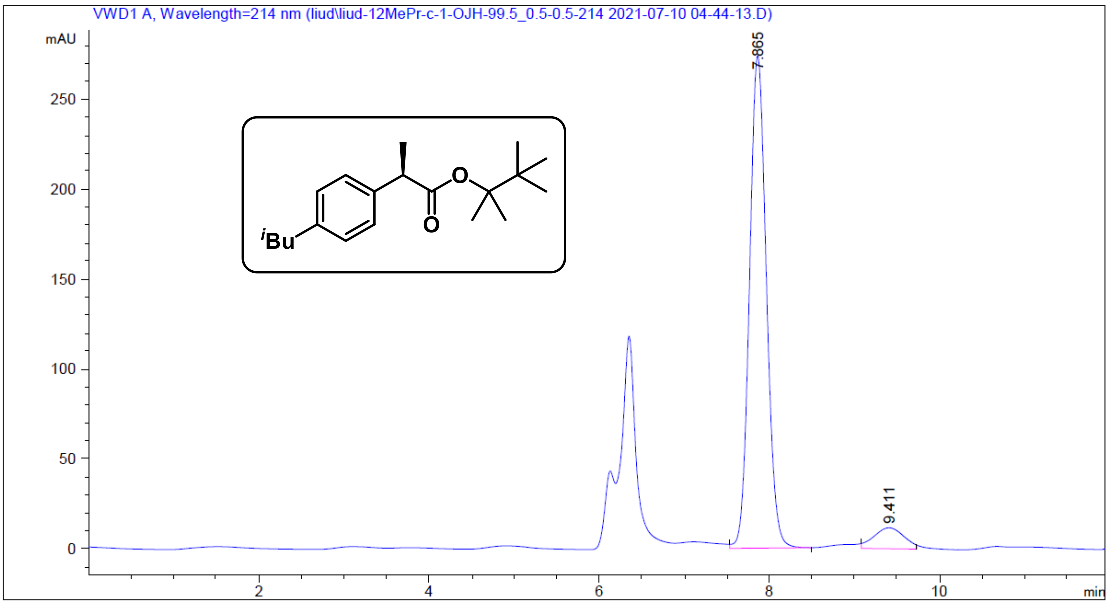

Peak	Ret Time [min]	Height [mAU]	Area %
1	7.865	273.95337	92.92145
2	9.411	11.66593	7.0785

Supplementary Figure 223. HPLC Chromatography of the Racemic 2,3,3-trimethylbutan-2-yl 2-(2-fluoro-[1,1'-biphenyl]-4-yl)propanoate (Daicel Chiralpak AD-H Column, *n*-Hexane: *i*-PrOH = 99.8:0.2, flow rate 0.5 mL/min, T = 25 °C, λ = 254 nm)

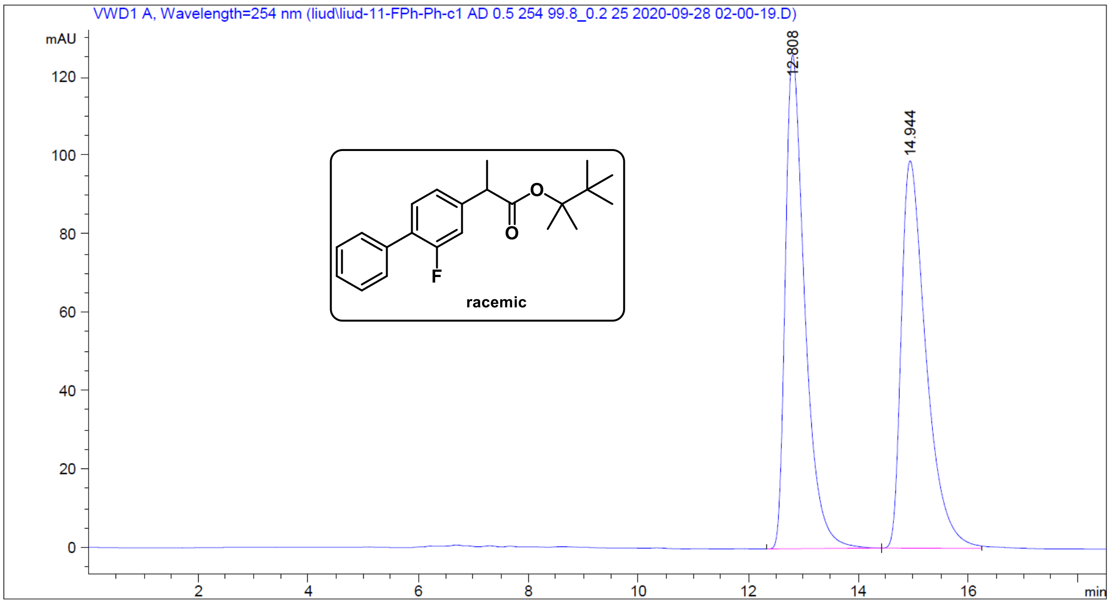

Peak	Ret Time [min]	Height [mAU]	Area %
1	12.808	125.98444	50.1471
2	14.944	98.84000	49.8529

Supplementary Figure 224. HPLC Chromatography of the 2,3,3-trimethylbutan-2-yl (*R*)-2-(2-fluoro-[1,1'-biphenyl]-4-yl)propanoate

(Daicel Chiralpak AD-H Column, *n*-Hexane: *i*-PrOH = 99.8:0.2, flow rate 0.5 mL/min, T = 25 °C, λ = 254 nm)

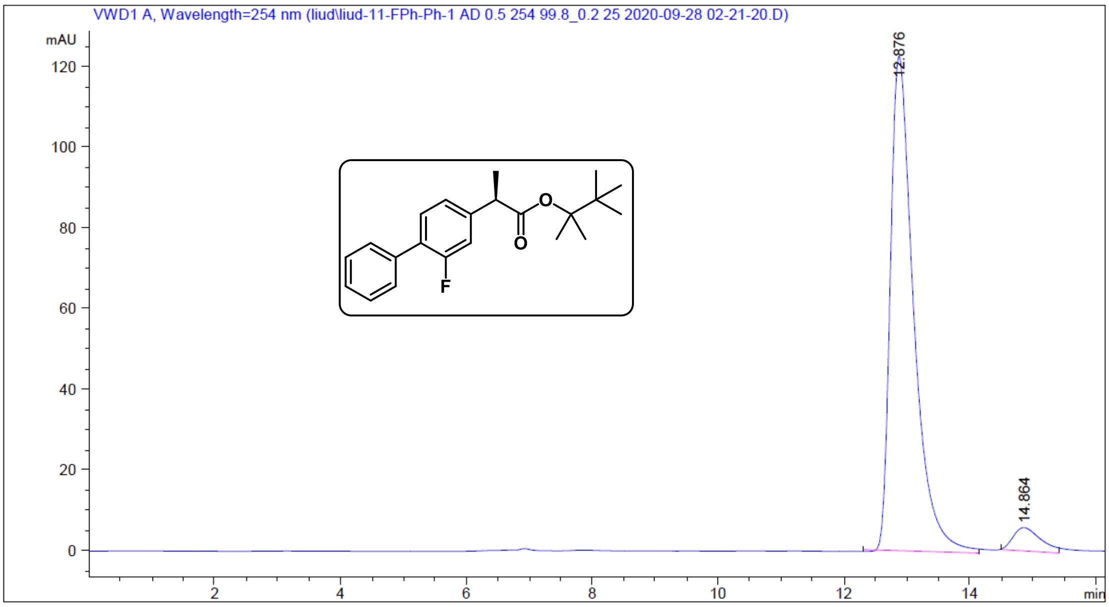

Peak	Ret Time [min]	Height [mAU]	Area %

1	12.876	122.64368	94.6879
2	14.864	5.77946	5.3121

Supplementary Figure 225. HPLC Chromatography of the Racemic 2,3,3-trimethylbutan-2-yl 2-(3-benzoylphenyl)propanoate

(Daicel Chiralpak IC-H Column, *n*-Hexane: *i*-PrOH = 98:2, flow rate 1.0 mL/min, T = 25 °C, λ = 214 nm)

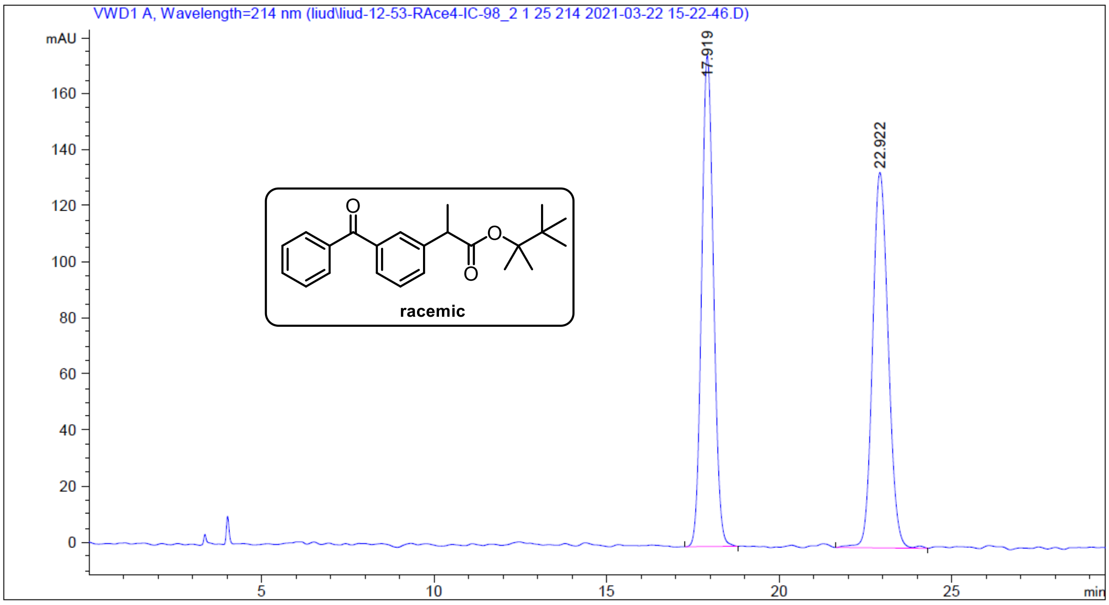

Peak	Ret Time [min]	Height [mAU]	Area %
1	17.919	175.22163	49.7472
2	22.922	133.89783	50.2528

Supplementary Figure 226. HPLC Chromatography of the 2,3,3-trimethylbutan-2-yl *(R)*-2-(4-benzoylphenyl)propanoate

(Daicel Chiralpak IC-H Column, *n*-Hexane: *i*-PrOH = 98:2, flow rate 1.0 mL/min, T = 25 °C, λ = 214 nm)

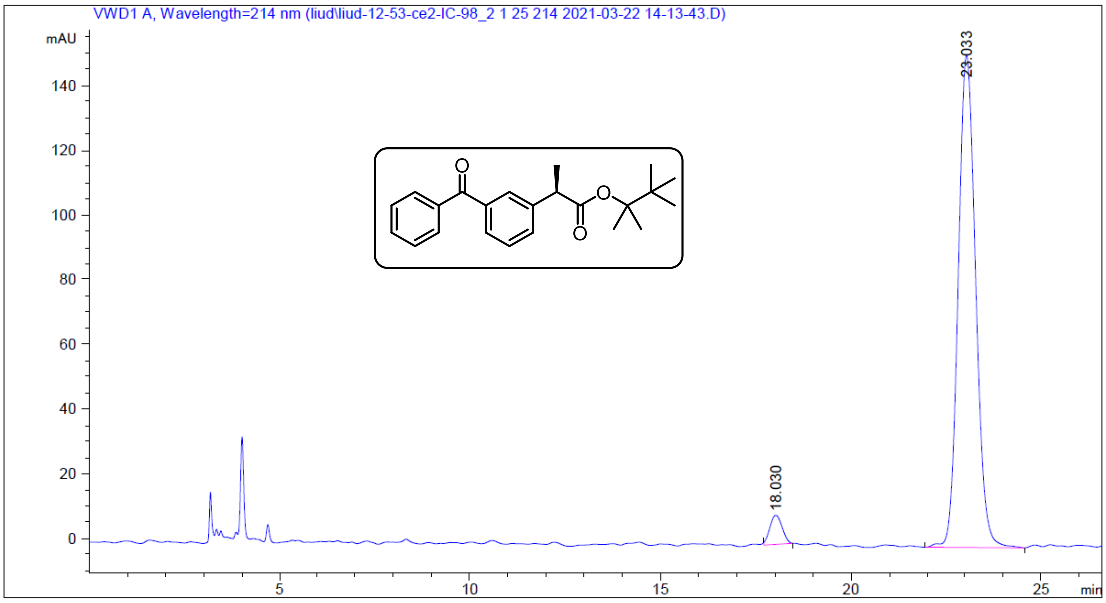

Peak	Ret Time [min]	Height [mAU]	Area %
1	18.030	8.98928	3.9993
2	23.033	152.14337	96.0007

Supplementary Figure 227. HPLC Chromatography of the Racemic 2,3,3-trimethylbutan-2-yl 2-(3-phenoxyphenyl)propanoate

(Daicel Chiralpak IC-H Column, *n*-Hexane: *i*-PrOH = 98:2, flow rate 1.0 mL/min, T = 25 °C, λ = 214 nm)

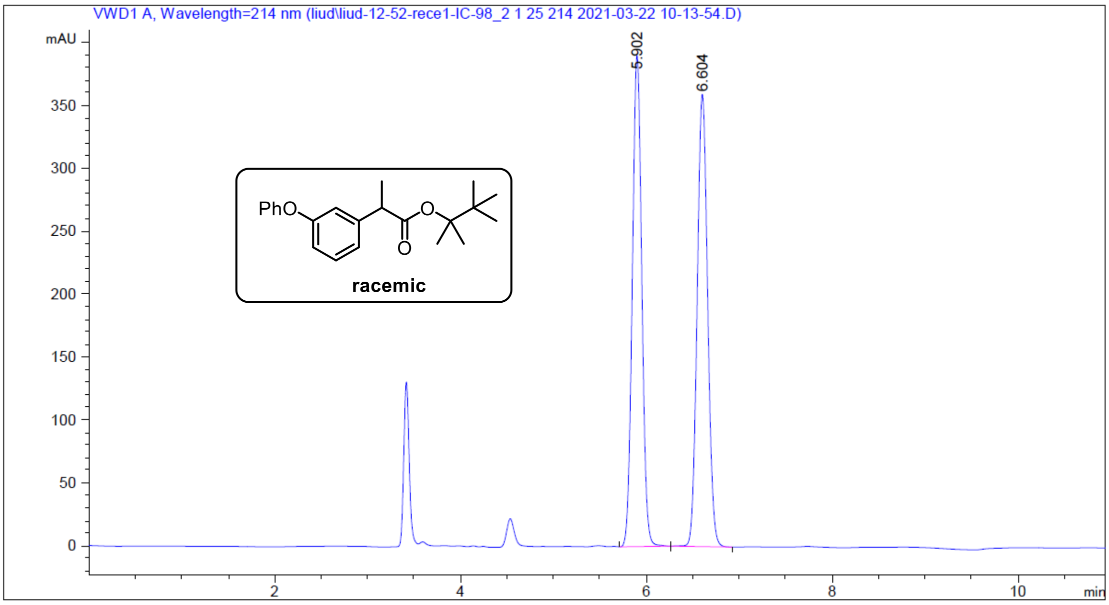

Peak	Ret Time [min]	Height [mAU]	Area %
1	5.902	389.15707	49.5570
2	6.604	358.48422	50.4430

Supplementary Figure 228. HPLC Chromatography of the 2,3,3-trimethylbutan-2-yl (*R*)-2-(3-phenoxyphenyl)propanoate

(Daicel Chiralpak IC-H Column, *n*-Hexane: *i*-PrOH = 98:2, flow rate 1.0 mL/min, T = 25 °C, λ = 214 nm)

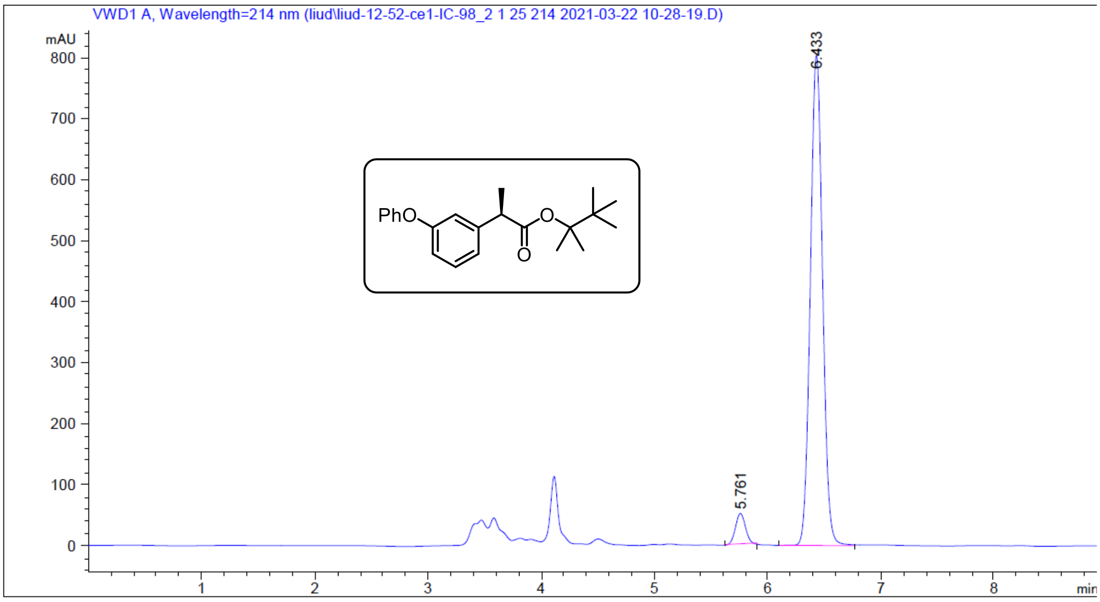

Peak	Ret Time [min]	Height [mAU]	Area %
1	5.761	49.73722	4.6583
2	6.433	805.83868	95.3417

Supplementary Figure 229. HPLC Chromatography of the Racemic 2,3,3-trimethylbutan-2-yl 2-(4-phenoxyphenyl)propanoate

(Daicel Chiralpak IC-H Column, *n*-Hexane: *i*-PrOH = 99:1, flow rate 0.5 mL/min, T = 25 °C, λ = 214 nm)

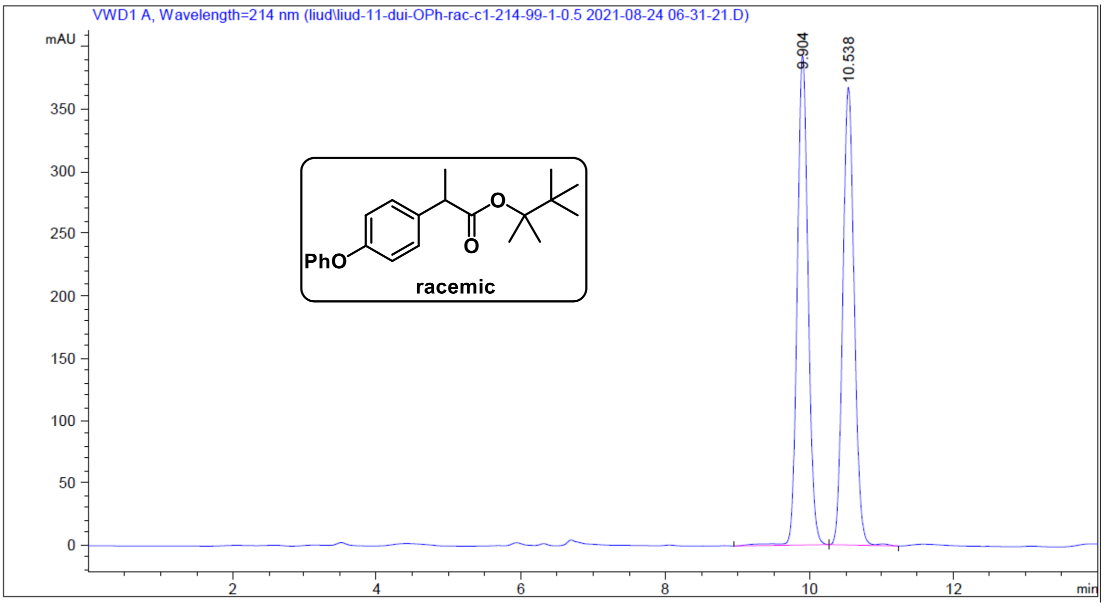

Peak	Ret Time [min]	Height [mAU]	Area %

1	9.904	392.76123	50.4189
2	10.538	367.45563	49.5811

Supplementary Figure 230. HPLC Chromatography of the 2,3,3-trimethylbutan-2-yl (*R*)-2-(4-phenoxyphenyl)propanoate (Daicel Chiralpak IC-H Column, *n*-Hexane: *i*-PrOH = 99:1, flow rate 1.0 mL/min, T = 25 °C, λ = 214 nm)

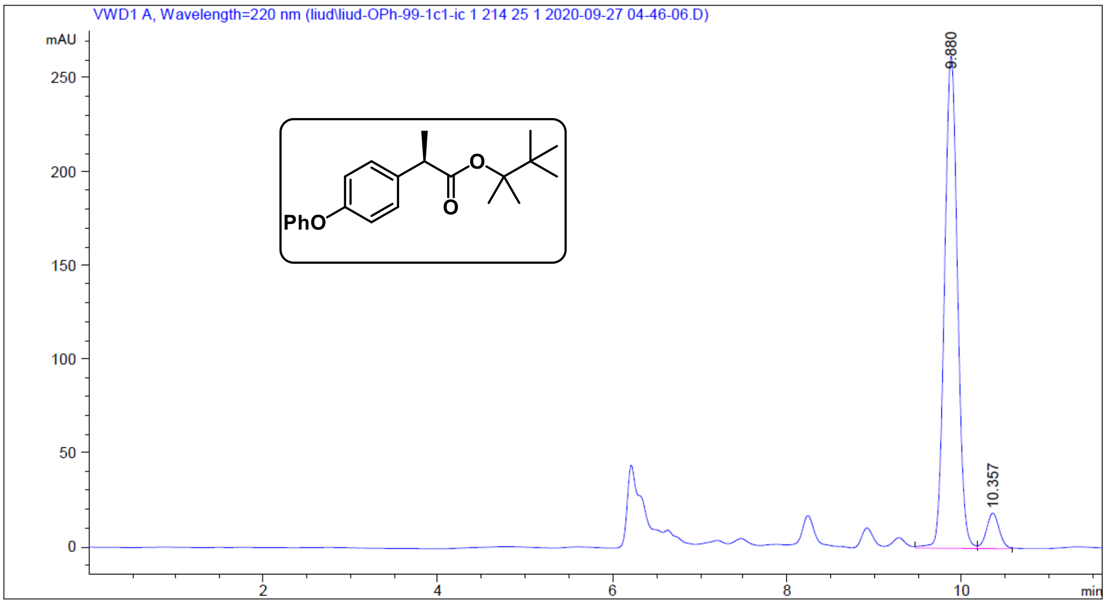

Peak	Ret Time [min]	Height [mAU]	Area %
1	9.880	263.26236	93.4022
2	10.357	18.93966	6.5978

Supplementary Figure 231. HPLC Chromatography of the Racemic Methyl 4-(3-(benzyloxy)-1-oxo-1-((2,3,3-trimethylbutan-2-yl)oxy)propan-2-yl)benzoate(Daicel Chiralpak IG-H Column, *n*-Hexane: *i*-PrOH = 98:2, flow rate 1.0 mL/min, T = 25 °C, λ = 214 nm)

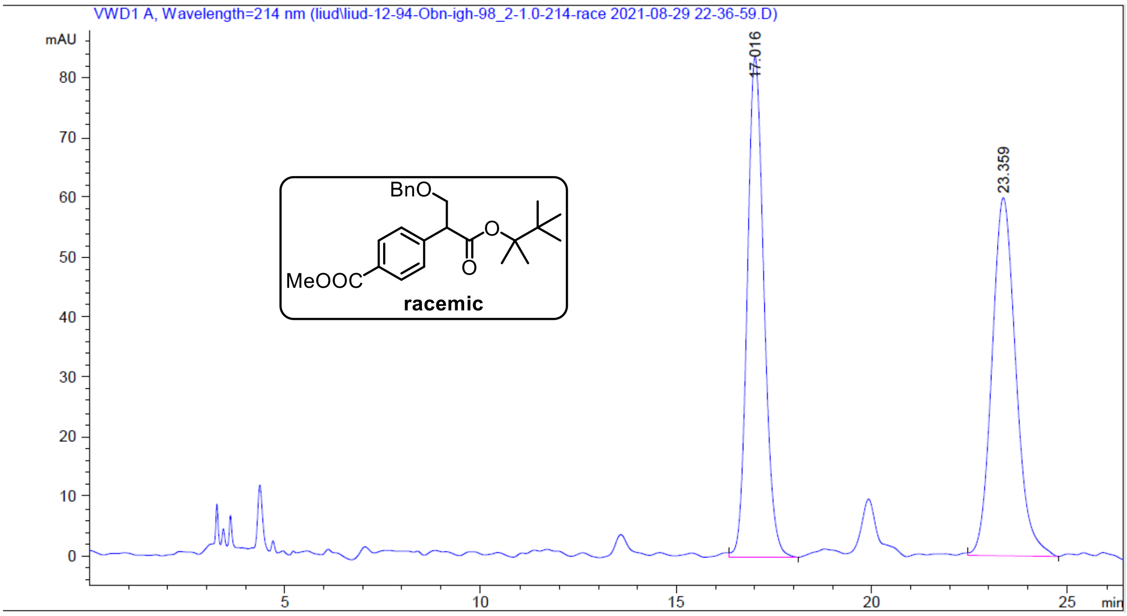

Peak	Ret Time [min]	Height [mAU]	Area %
1	17.016	83.67194	49.3615
2	23.359	59.81961	50.6385

Supplementary Figure 232. HPLC Chromatography of the Methyl (*S*)-4-(3-(benzyloxy)-1-oxo-1-((2,3,3-trimethylbutan-2-yl)oxy)propan-2-yl)benzoate

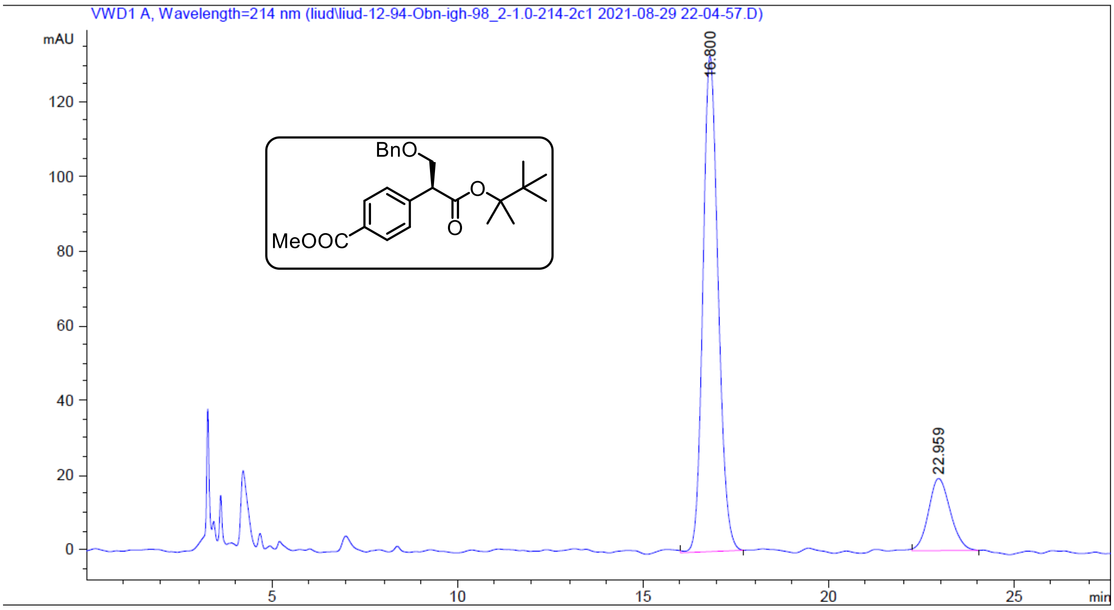

Peak	Ret Time [min]	Height [mAU]	Area %
1	16.800	132.98958	82.7178
2	22.959	19.35618	17.2822

Supplementary Figure 233. HPLC Chromatography of the Methyl 4-(3-(4-methoxyphenyl)-1-oxo-1-((2,3,3-trimethylbutan-2-yl)oxy)propan-2-yl)benzoate (Daicel Chiralpak IG-H Column, *n*-Hexane: *i*-PrOH = 98:2, flow rate 0.8 mL/min, T = 25 °C, λ = 214 nm)

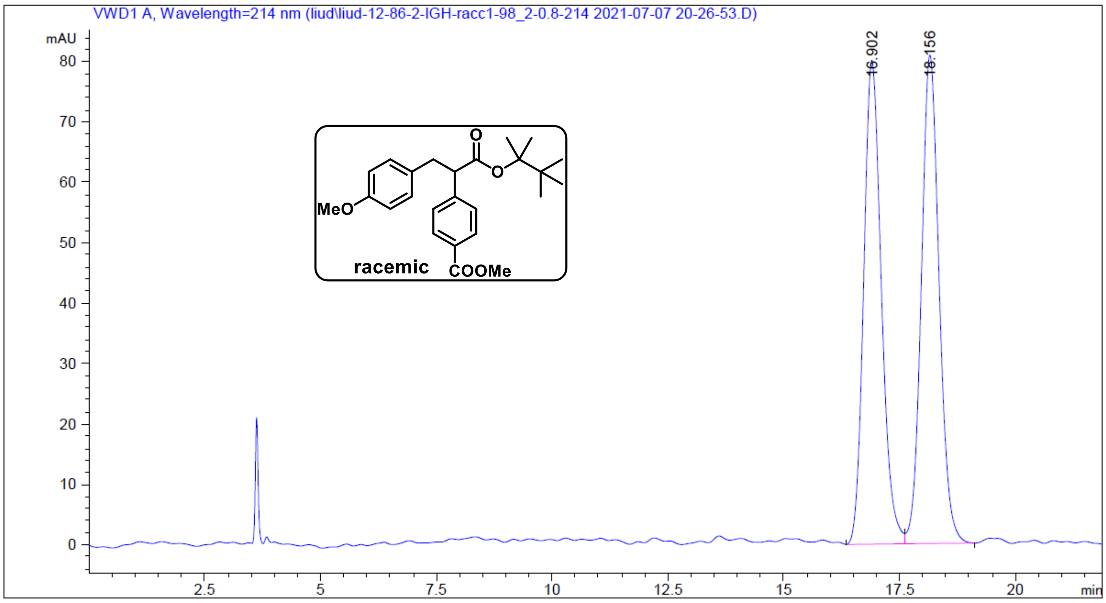

Peak	Ret Time [min]	Height [mAU]	Area %
1	16.902	80.07359	49.5074
2	18.156	80.81141	50.4926

Supplementary Figure 234. HPLC Chromatography of the Methyl (*R*)-4-(3-(4-methoxyphenyl)-1-oxo-1-((2,3,3-trimethylbutan-2-yl)oxy)propan-2-yl)benzoate

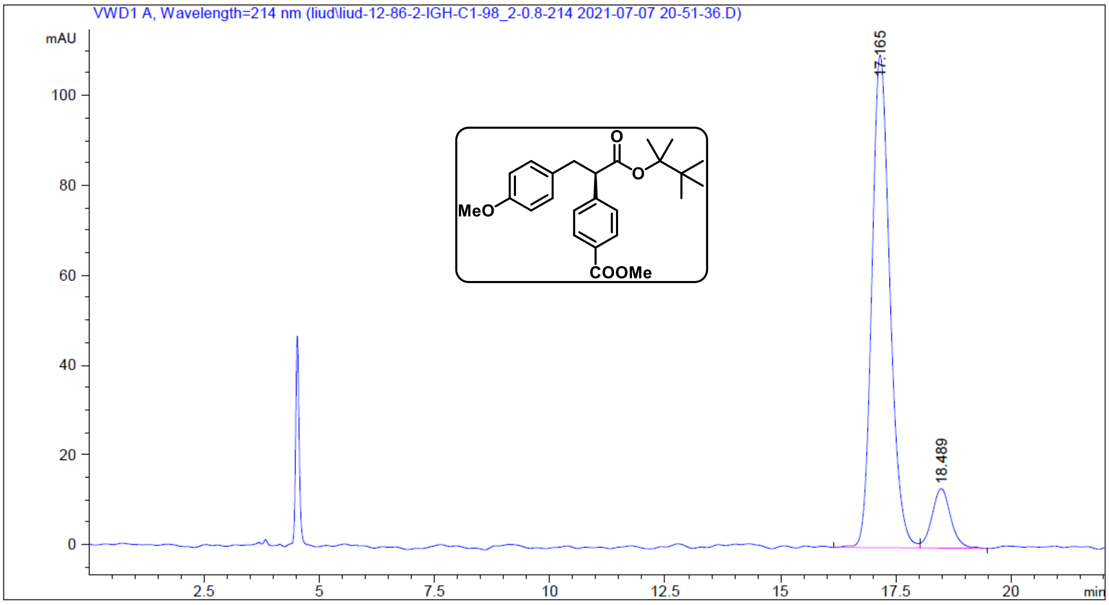

Peak	Ret Time [min]	Height [mAU]	Area %
1	17.165	109.70339	88.8398
2	18.489	13.25058	11.1602

Supplementary Figure 235. HPLC Chromatography of the Methyl 4-(1-oxo-3-phenyl-1-((2,3,3-trimethylbutan-2-yl)oxy)propan-2-yl)benzoate (Daicel Chiralpak IC-H Column, *n*-Hexane: *i*-PrOH = 98:2, flow rate 1.0 mL/min, T = 25 °C, λ = 214 nm)

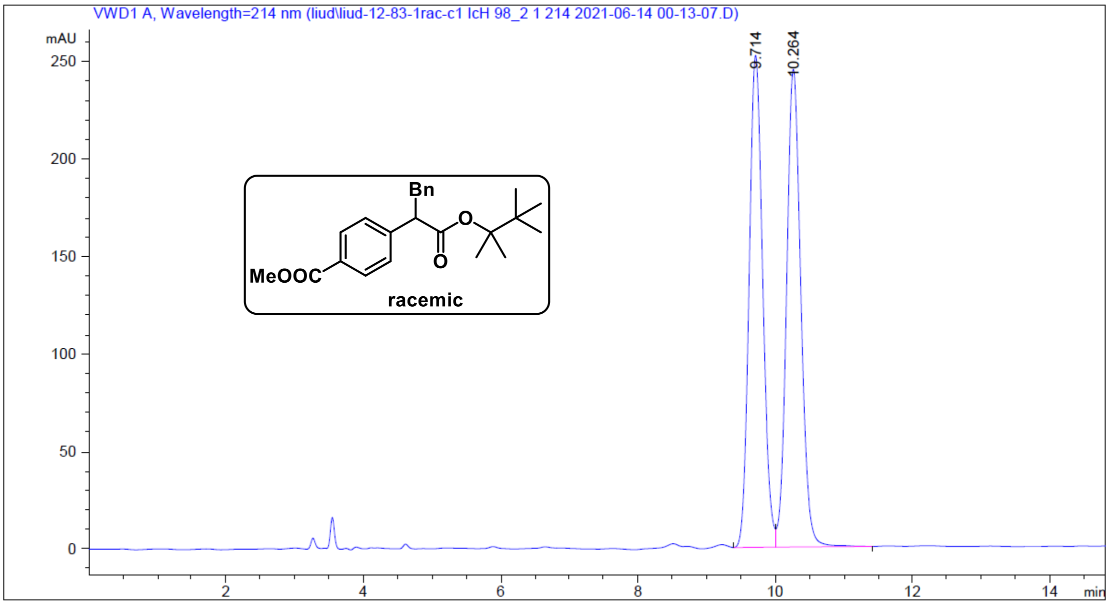

Peak	Ret Time [min]	Height [mAU]	Area %
1	9.714	252.49284	49.5207
2	10.264	245.17847	50.4793

Supplementary Figure 236. HPLC Chromatography of the Methyl (*R*)-4-(1-oxo-3-phenyl-1-((2,3,3-trimethylbutan-2-yl)oxy)propan-2-yl)benzoate

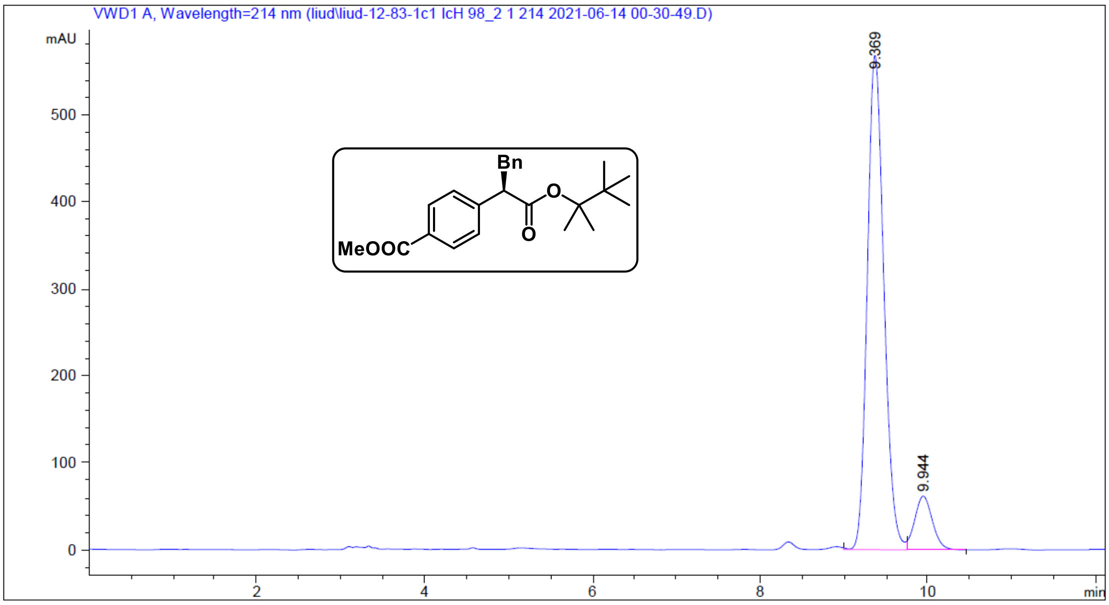

Peak	Ret Time [min]	Height [mAU]	Area %
1	9.369	567.95636	89.6666
2	9.944	61.61660	10.3334

Supplementary Figure 237. HPLC Chromatography of the Methyl 4-(1-oxo-4-phenyl-1-((2,3,3-trimethylbutan-2-yl)oxy)butan-2-yl)benzoate

(Daicel Chiralpak IC-H Column, *n*-Hexane: *i*-PrOH = 98:2, flow rate 1.0 mL/min, T = 25 °C, λ = 214 nm)

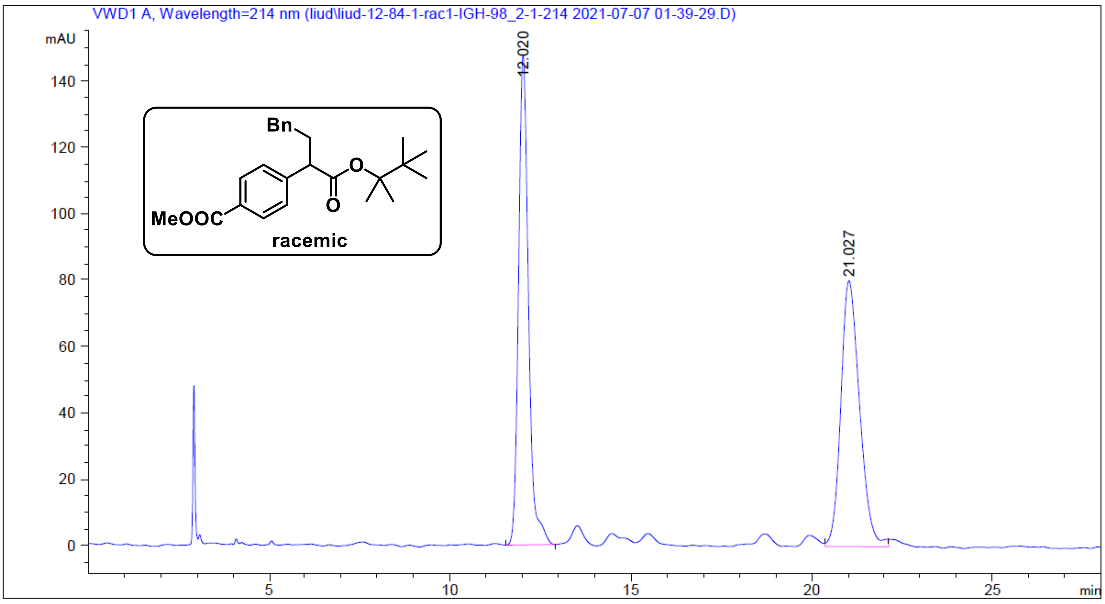

Peak	Ret Time [min]	Height [mAU]	Area %
1	12.020	147.55556	49.4371
2	21.027	80.16261	50.5629

Supplementary Figure 238. HPLC Chromatography of the Methyl (*R*)-4-(1-oxo-4-phenyl-1-((2,3,3-trimethylbutan-2-yl)oxy)butan-2-yl)benzoate

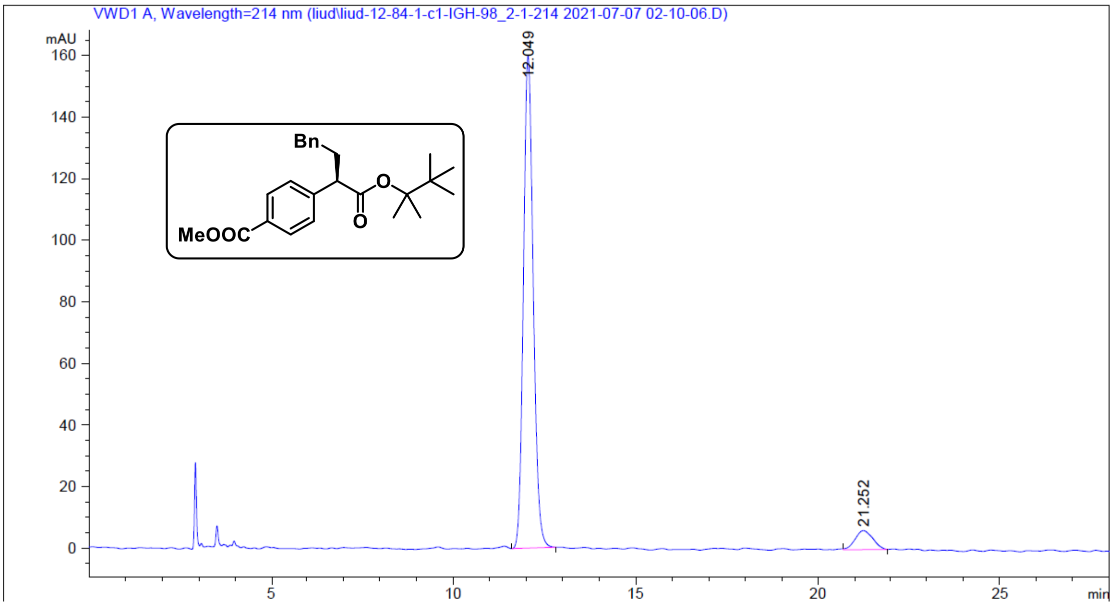

Peak	Ret Time [min]	Height [mAU]	Area %
1	12.049	159.64944	93.5613
2	21.252	6.14400	6.4387

Supplementary Figure 239. HPLC Chromatography of the Methyl 4-(4-(methylthio)-1-oxo-1-((2,3,3-trimethylbutan-2-yl)oxy)butan-2-yl)benzoate (Daicel Chiralpak IG-H Column, *n*-Hexane: *i*-PrOH = 98:2, flow rate 0.9 mL/min, T = 25 °C, λ = 214 nm)

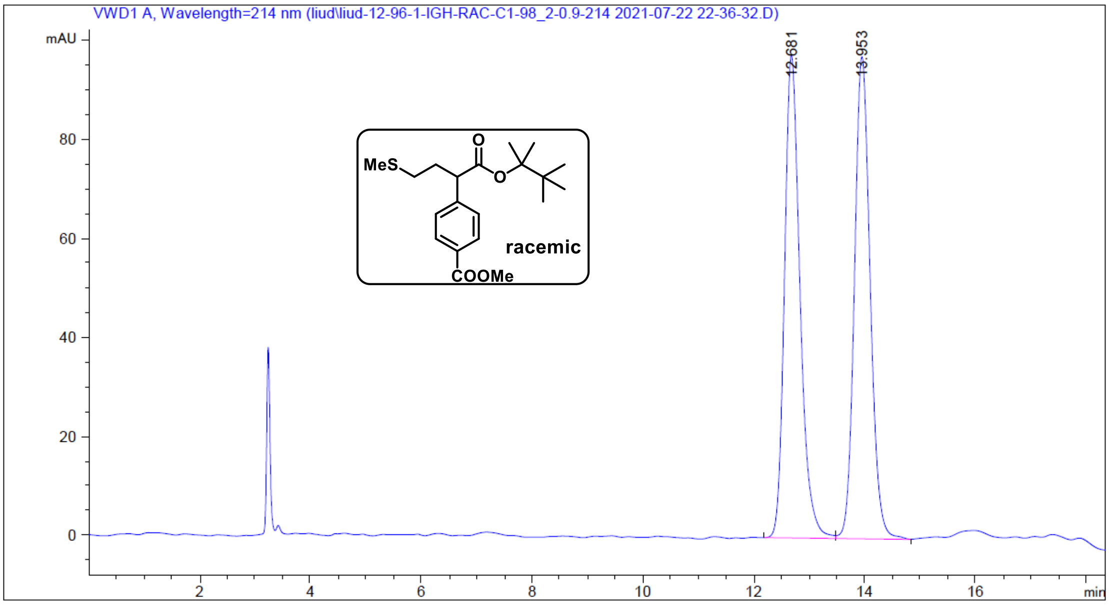

Peak	Ret Time [min]	Height [mAU]	Area %
1	12.681	97.61547	49.6865
2	13.953	97.49183	50.3135

Supplementary Figure 240. HPLC Chromatography of the Methyl (*R*)-4-(4-(methylthio)-1-oxo-1-((2,3,3-trimethylbutan-2-yl)oxy)butan-2-yl)benzoate

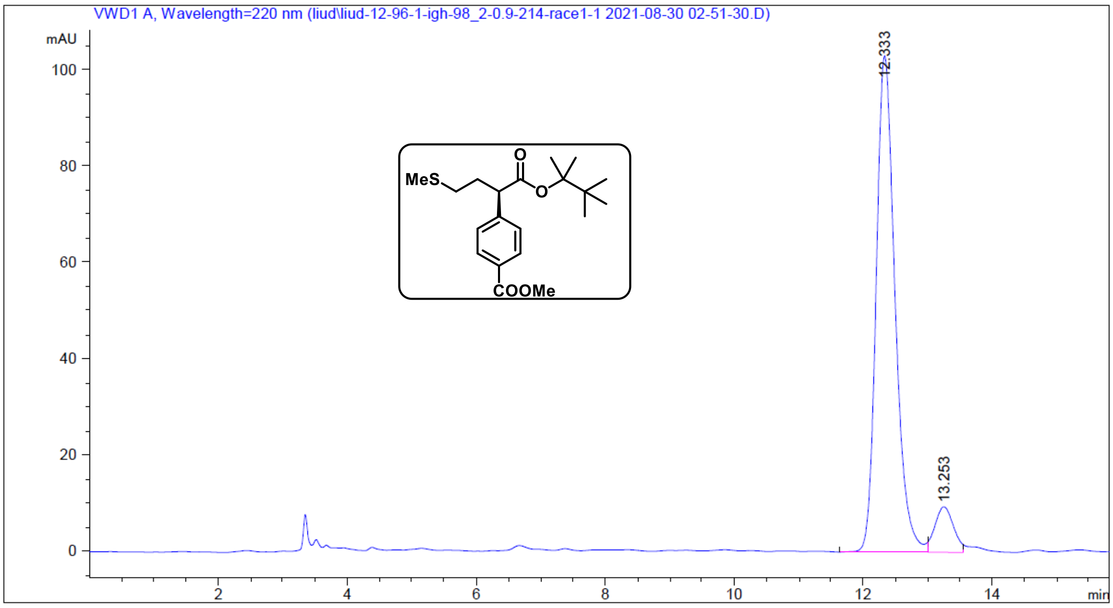

Peak	Ret Time [min]	Height [mAU]	Area %
1	12.333	103.13371	91.7095
2	13.253	9.45558	8.2905

Supplementary Figure 241. HPLC Chromatography of the 4-(1-oxo-1-((2,3,3-trimethylbutan-2-yl)oxy)hept-6-en-2-yl)benzoate

(Daicel Chiralpak IG-H Column, *n*-Hexane: *i*-PrOH = 98:2, flow rate 0.8 mL/min, T = 25 °C, λ = 214 nm)

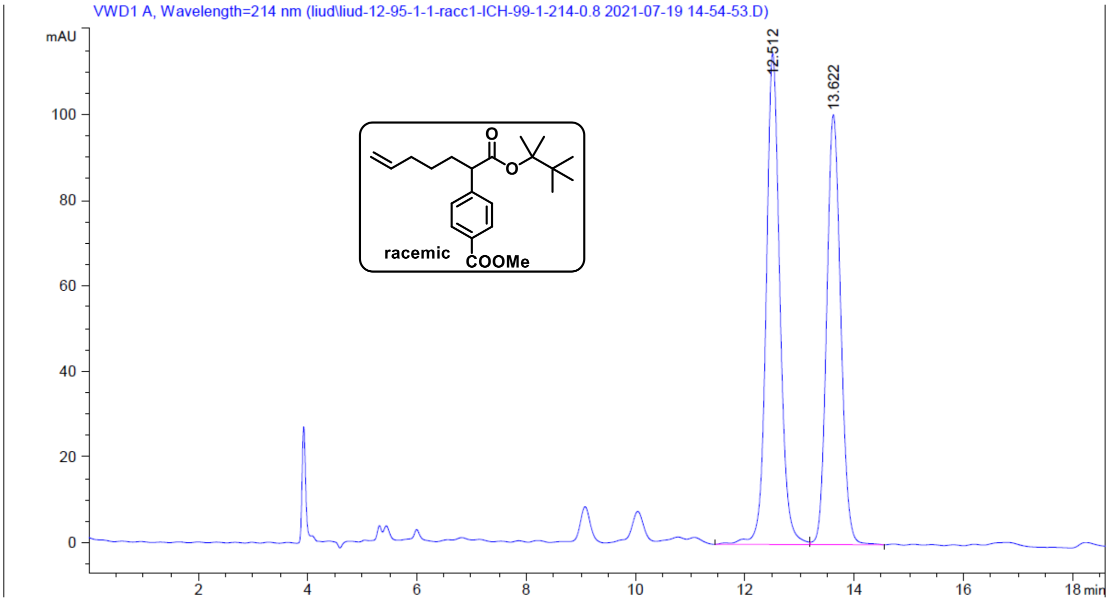

Peak	Ret Time [min]	Height [mAU]	Area %
1	12.512	114.86185	52.8112
2	13.622	100.53183	47.1888

Supplementary Figure 242. HPLC Chromatography of the (*R*)-4-(1-oxo-1-((2,3,3-trimethylbutan-2-yl)oxy)hept-6-en-2-yl)benzoate

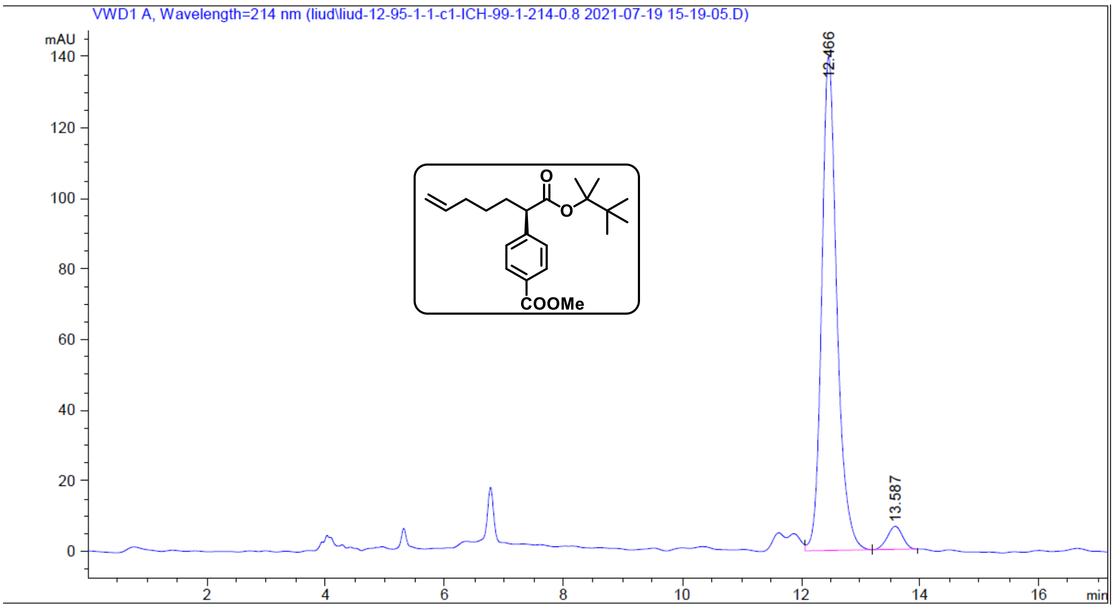

Peak	Ret Time [min]	Height [mAU]	Area %
1	12.466	139.99232	95.5411

2	13.587	6.53249	4.4589

Supplementary Figure 243. HPLC Chromatography of the Methyl 4-(1-oxo-1-((2,3,3-trimethylbutan-2-yl)oxy)butan-2-yl)benzoate

(Daicel Chiralpak IC-H Column, *n*-Hexane: *i*-PrOH = 98:2, flow rate 0.5 mL/min, T = 25 °C, λ = 214 nm)

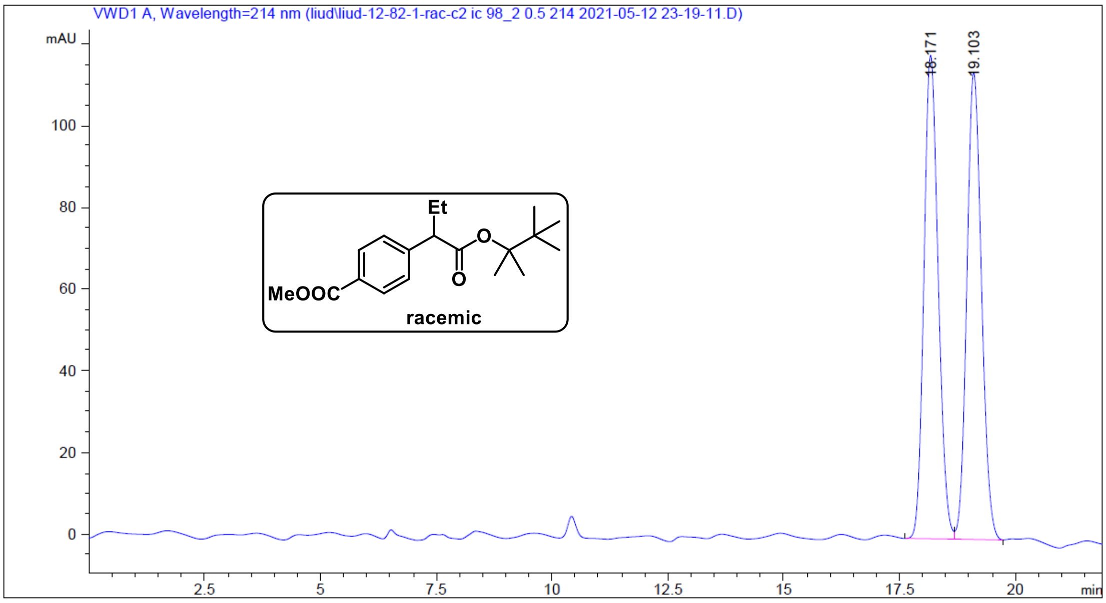

Peak	Ret Time [min]	Height [mAU]	Area %
1	18.171	118.32067	50.1839
2	19.103	114.19765	49.8161

Supplementary Figure 244. HPLC Chromatography of the Methyl (*R*)-4-(1-oxo-1-((2,3,3-trimethylbutan-2-yl)oxy)butan-2-yl)benzoate

(Daicel Chiralpak IC-H Column, *n*-Hexane: *i*-PrOH = 98:2, flow rate 0.5 mL/min, T = 25 °C, λ = 214 nm)

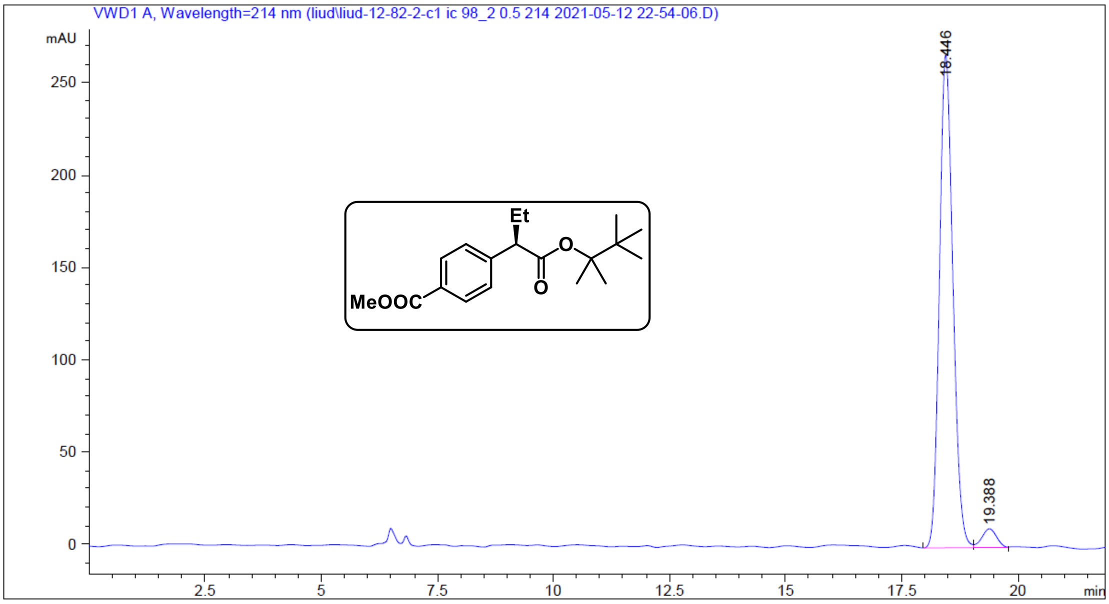

Peak	Ret Time [min]	Height [mAU]	Area %
1	18.446	266.69272	95.9747
2	19.388	10.23030	4.0253

Supplementary Figure 245. HPLC Chromatography of the Methyl 4-(1-oxo-1-((2,3,3-trimethylbutan-2-yl)oxy)hexan-2-yl)benzoate

(Daicel Chiralpak IG-H Column, *n*-Hexane: *i*-PrOH = 98:2, flow rate 0.5 mL/min, T = 25 °C, λ = 214 nm)

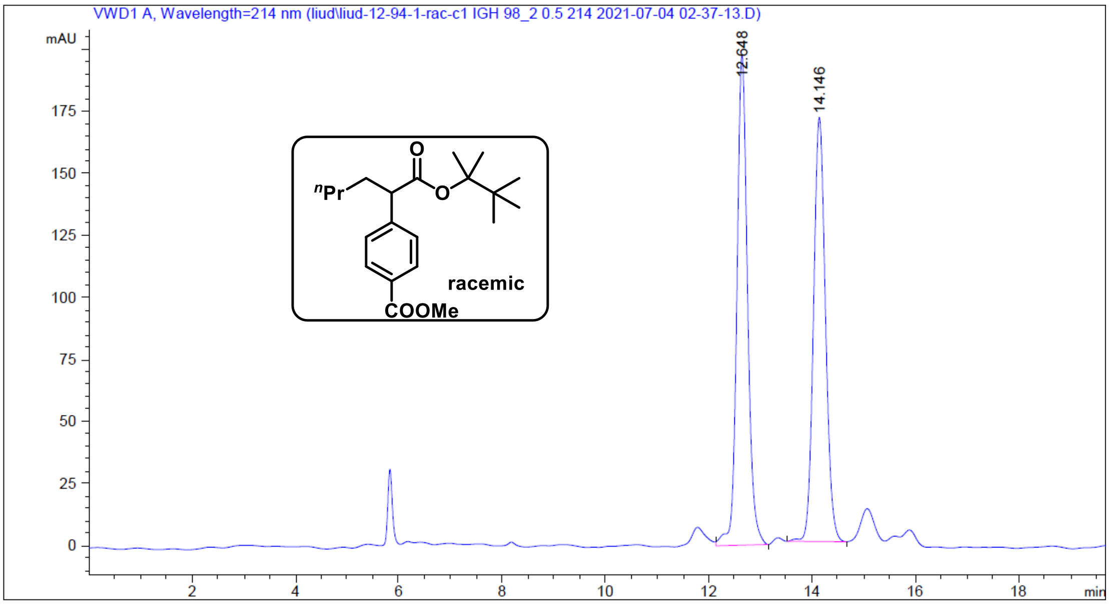

Peak	Ret Time [min]	Height [mAU]	Area %
1	12.648	197.39616	50.8869
2	14.146	170.96561	49.1131

Supplementary Figure 246. HPLC Chromatography of the Methyl (*R*)-4-(1-oxo-1-((2,3,3-trimethylbutan-2-yl)oxy)hexan-2-yl)benzoate

(Daicel Chiralpak IG-H Column, *n*-Hexane: *i*-PrOH = 98:2, flow rate 0.5 mL/min, T = 25 °C, λ = 214 nm)

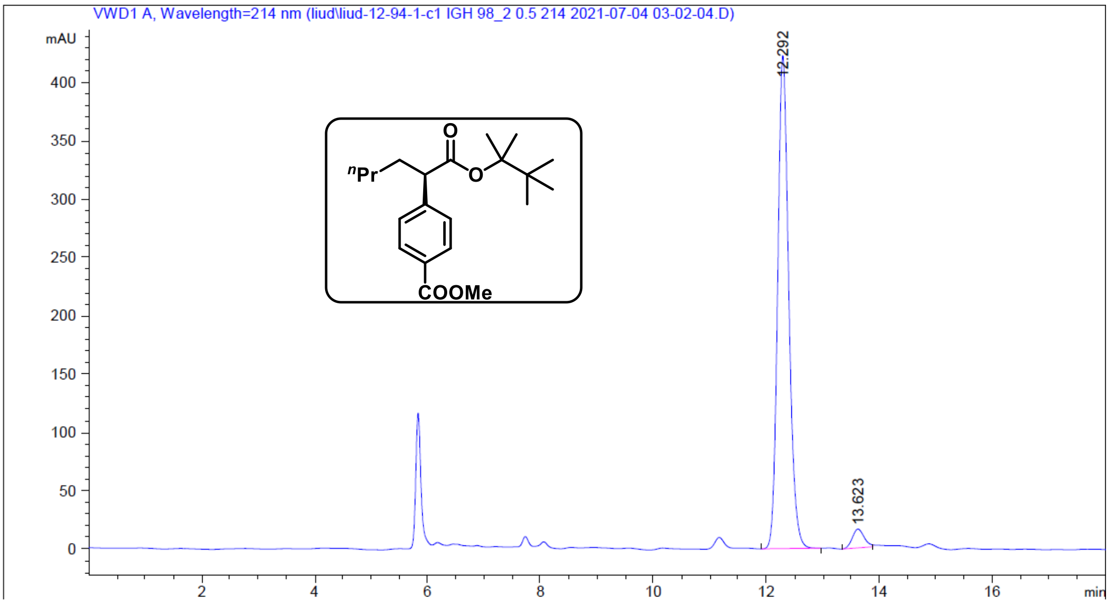

Peak	Ret Time [min]	Height [mAU]	Area %
1	12.292	422.72397	96.0545
2	13.623	16.15827	3.9455

Supplementary Figure 247. HPLC Chromatography of the Methyl 4-(4-methyl-1-oxo-1-((2,3,3-trimethylbutan-2-yl)oxy)pentan-2-yl)benzoate

(Daicel Chiralpak IC-H Column, *n*-Hexane: *i*-PrOH = 99:1, flow rate 0.4 mL/min, T = 25 °C, λ = 214 nm)

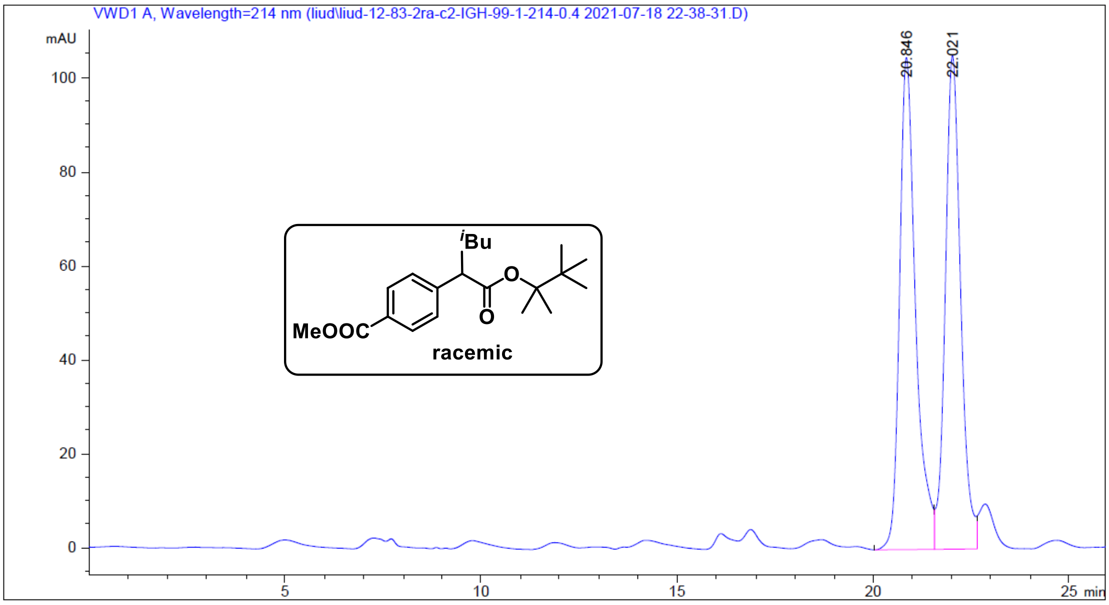

Peak	Ret Time [min]	Height [mAU]	Area %

1	20.846	104.70322	50.9225
2	22.021	105.08278	49.0775

Supplementary Figure 248. HPLC Chromatography of the Methyl (*R*)-4-(4-methyl-1-oxo-1-((2,3,3-trimethylbutan-2-yl)oxy)pentan-2-yl)benzoate

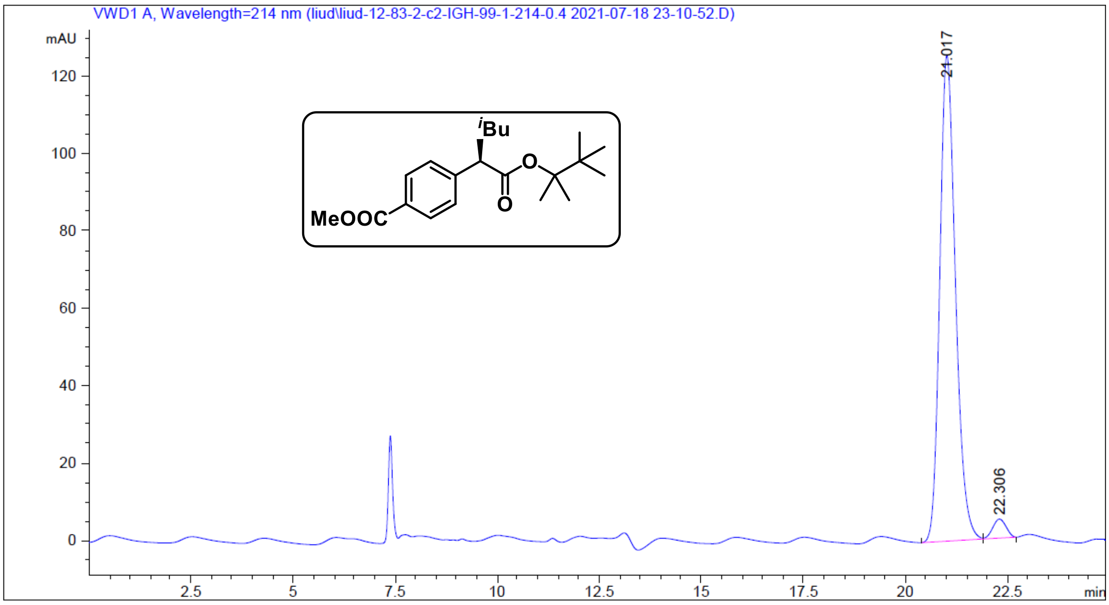

Peak	Ret Time [min]	Height [mAU]	Area %
1	21.017	125.47861	96.7863
2	22.306	4.92390	3.2137

Supplementary Figure 249. HPLC Chromatography of the Methyl 4-(3-methyl-1-oxo-1-((2,3,3-trimethylbutan-2-yl)oxy)butan-2-yl)benzoate

(Daicel Chiralpak IC-H Column, *n*-Hexane: *i*-PrOH = 99:1, flow rate 0.5 mL/min, T = 25 °C, λ = 214 nm)

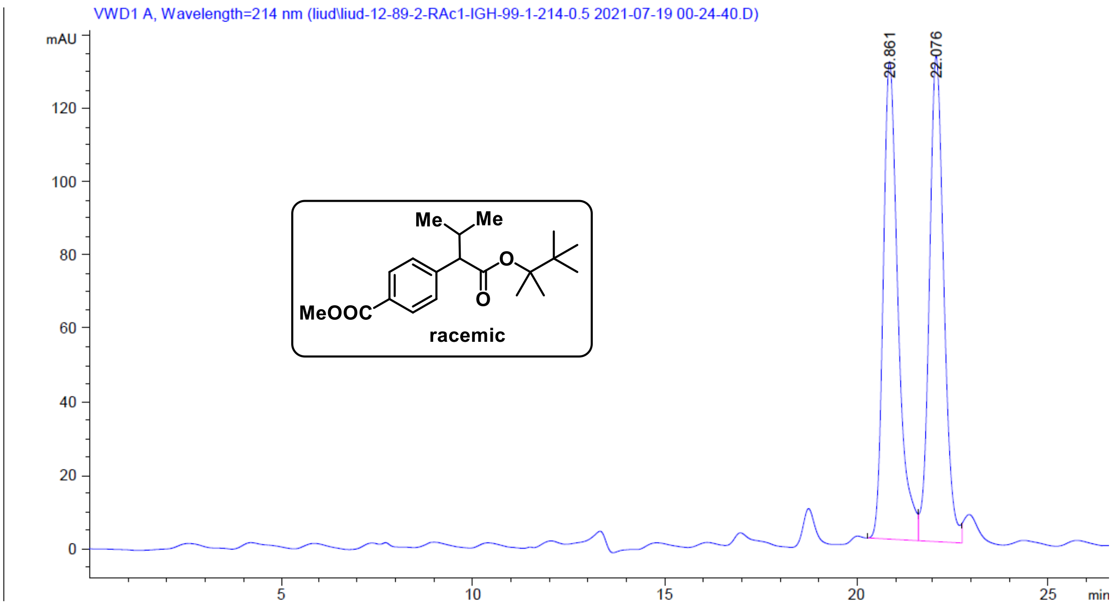

Peak	Ret Time [min]	Height [mAU]	Area %
1	20.861	130.16118	50.0693
2	22.076	132.31940	49.9307

Supplementary Figure 250. HPLC Chromatography of the Methyl (*R*)-4-(3-methyl-1-oxo-1-((2,3,3-trimethylbutan-2-yl)oxy)butan-2-yl)benzoate

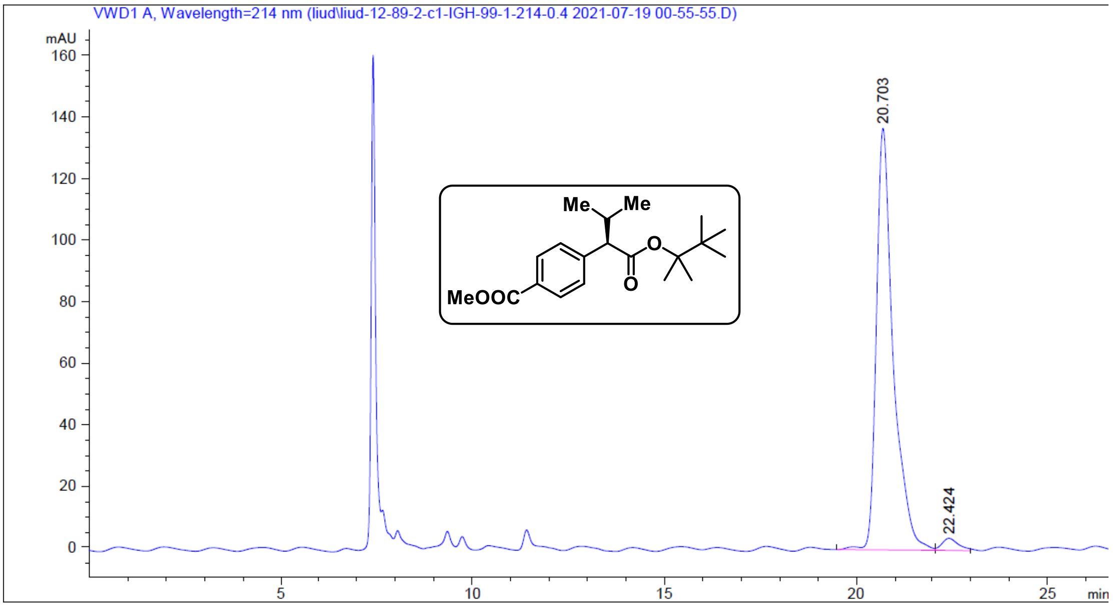

Peak	Ret Time [min]	Height [mAU]	Area %
1	20.703	137.11394	97.2850
2	22.424	3.94822	2.7150

Supplementary Figure 251. HPLC Chromatography of the Methyl 4-(1-cyclopentyl-2-oxo-2-((2,3,3-trimethylbutan-2-yl)oxy)ethyl)benzoate

(Daicel Chiralpak IG-H Column, *n*-Hexane: *i*-PrOH = 98:2, flow rate 0.5 mL/min, T = 25 °C, λ = 214 nm)

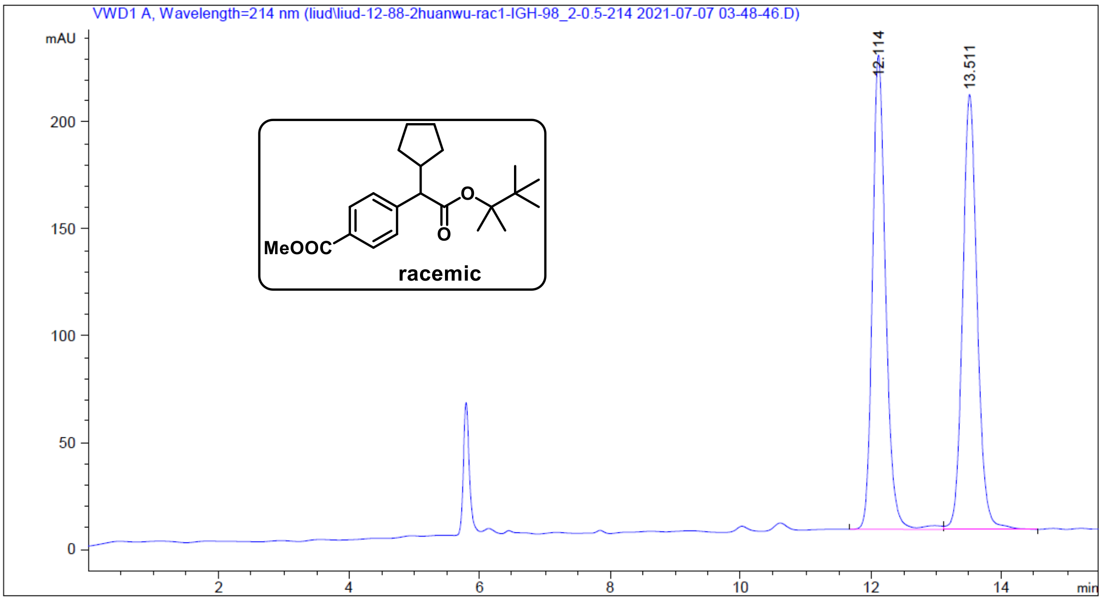

Peak	Ret Time [min]	Height [mAU]	Area %
1	12.114	222.16972	49.7235
2	13.511	203.72284	50.2765

Supplementary Figure 252. HPLC Chromatography of the Methyl (*R*)-4-(1-cyclopentyl-2-oxo-2-((2,3,3-trimethylbutan-2-yl)oxy)ethyl)benzoate

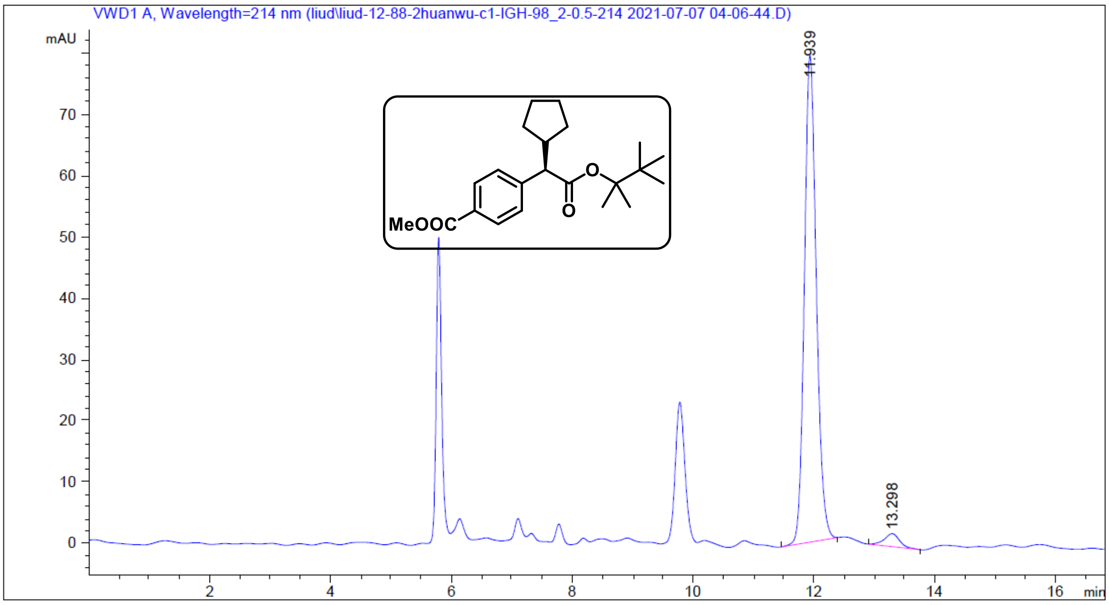

Peak	Ret Time [min]	Height [mAU]	Area %
1	11.939	79.47903	96.7106
2	13.298	2.11271	3.2894

Supplementary Figure 253. HPLC Chromatography of the Methyl 4-(1-cyclohexyl-2-oxo-2-((2,3,3-trimethylbutan-2-yl)oxy)ethyl)benzoate

(Daicel Chiralpak IC-H Column, *n*-Hexane: *i*-PrOH = 99:1, flow rate 1.0 mL/min, T = 25 °C, λ = 214 nm)

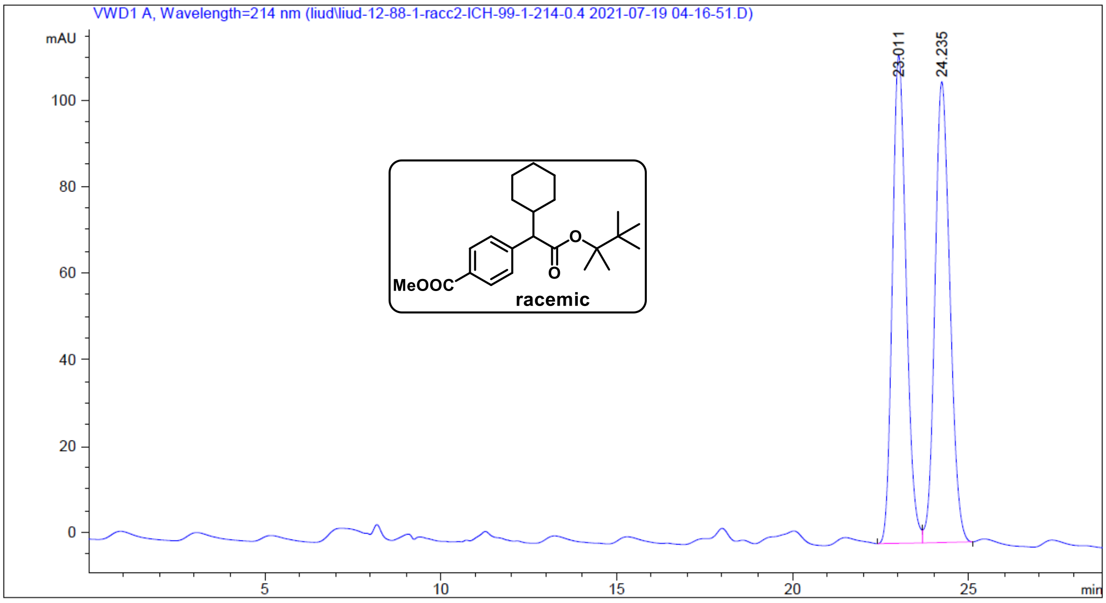

Peak	Ret Time [min]	Height [mAU]	Area %
1	23.011	112.87428	49.3600
2	24.235	106.54940	50.6400

Supplementary Figure 254. HPLC Chromatography of the Methyl (*R*)-4-(1-cyclohexyl-2-oxo-2-((2,3,3-trimethylbutan-2-yl)oxy)ethyl)benzoate

(Daicel Chiralpak IC-H Column, *n*-Hexane: *i*-PrOH = 99:1, flow rate 1.0 mL/min, T = 25 °C, λ = 214 nm)

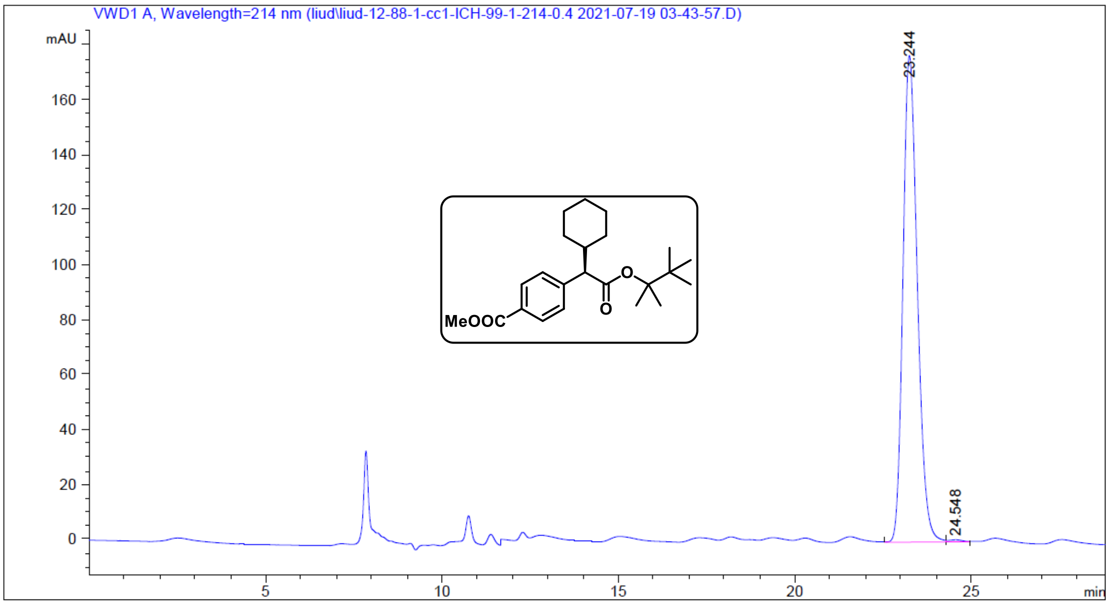

Peak	Ret Time [min]	Height [mAU]	Area %
1	23.244	177.15358	99.5174
2	24.548	8.33065e-1	0.4826

Supplementary Figure 255. HPLC Chromatography of the 4-(1-oxo-1-((2,3,3-trimethylbutan-2-yl)oxy)hept-6-en-2-yl)benzoate

(Daicel Chiralpak IC-H Column, *n*-Hexane: *i*-PrOH = 99:1, flow rate 1.0 mL/min, T = 25 °C, λ = 214 nm)

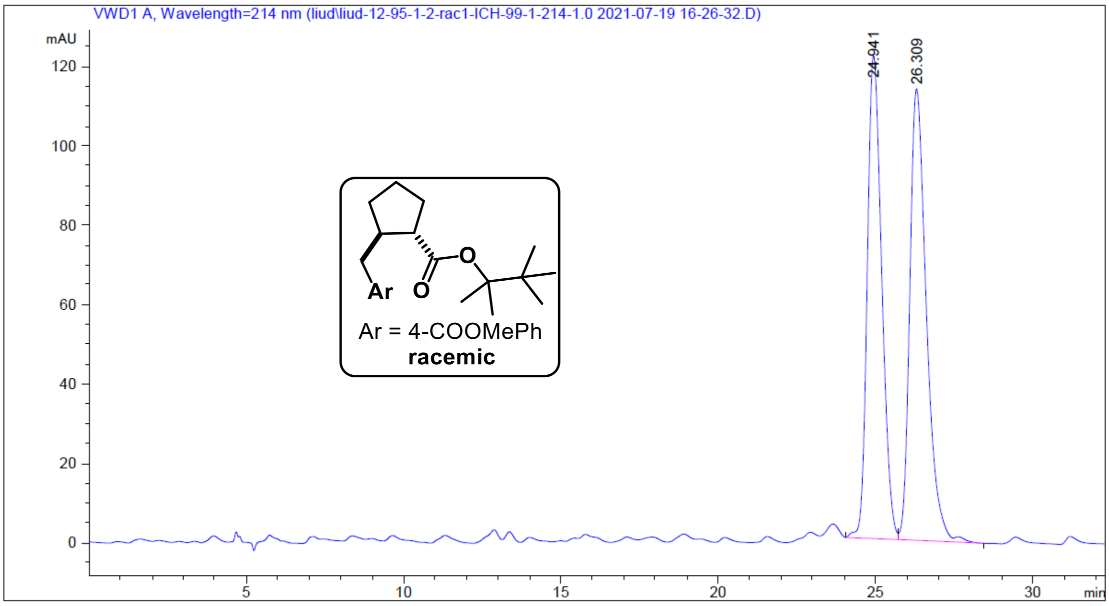

Peak	Ret Time [min]	Height [mAU]	Area %
1	24.941	121.65689	48.2383
2	26.309	113.74099	51.7617

Supplementary Figure 256. HPLC Chromatography of the Methyl 4-(((1*R*, 2*S*)-2-(((2,3,3-trimethylbutan-2-yl)oxy)carbonyl)cyclopentyl)methyl)benzoate (Daicel Chiralpak IC-H Column, *n*-Hexane: *i*-PrOH = 99.0:1.0, flow rate 1.0 mL/min, T = 25 °C, λ = 214 nm): t_R = 24.94 min (major), t_R = 26.30 min (minor)

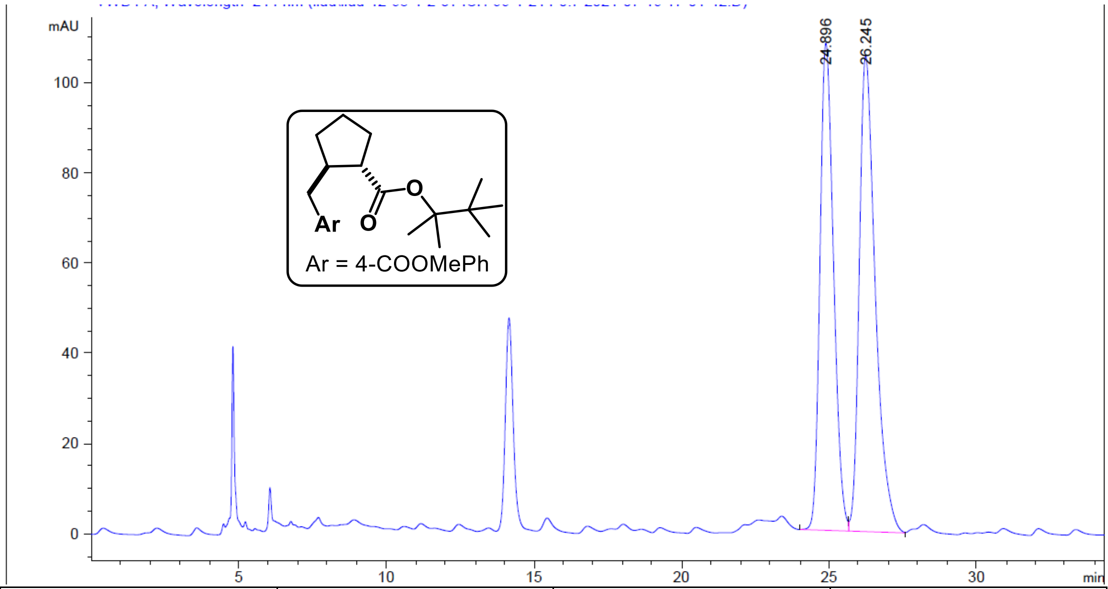

Peak	Ret Time [min]	Height [mAU]	Area %

1	24.896	108.21426	46.3759
2	26.245	105.56319	53.6241

Supplementary Figure 257. HPLC Chromatography of the Methyl 4-(((1*R*, 2*R*)-2-(((2,3,3-trimethylbutan-2-yl)oxy)carbonyl)cyclopentyl)methyl)benzoate (Daicel Chiralpak IG-H Column, *n*-Hexane: *i*-PrOH = 99:1, flow rate 0.7 mL/min, T = 25 °C, λ = 214 nm)

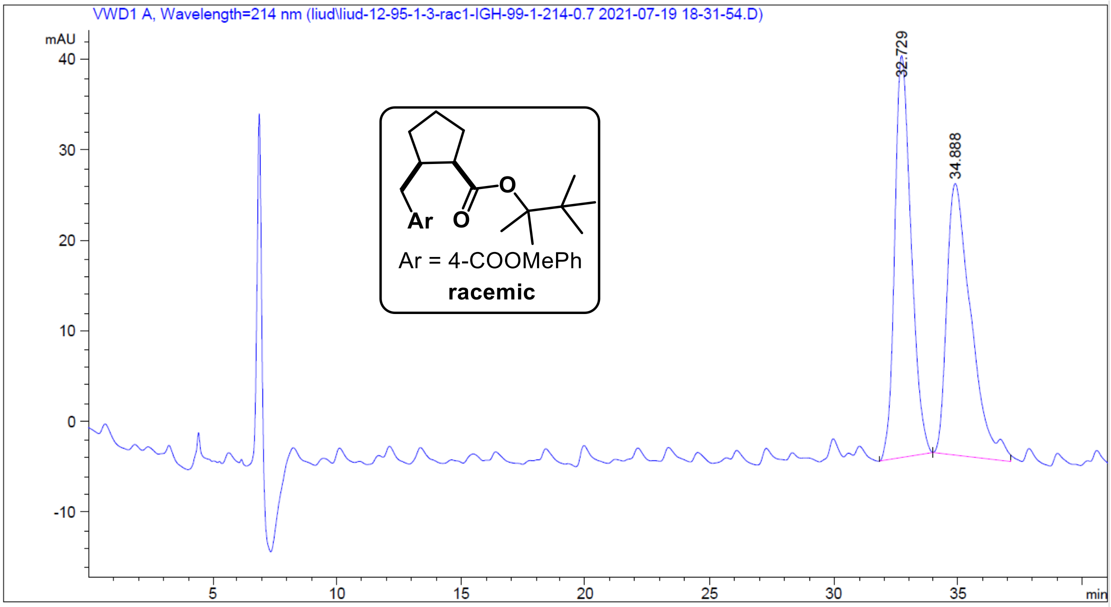

Peak	Ret Time [min]	Height [mAU]	Area %
1	32.729	44.38659	50.1372
2	34.888	29.97759	49.8628

Supplementary Figure 258. HPLC Chromatography of the Racemic 2-(2-fluoro-[1,1'-biphenyl]-4-yl)propanoic acid

(Daicel Chiralpak AD-H Column, *n*-Hexane: *i*-PrOH = 90:10, flow rate 1.0 mL/min, T = 25 °C, λ = 254 nm)

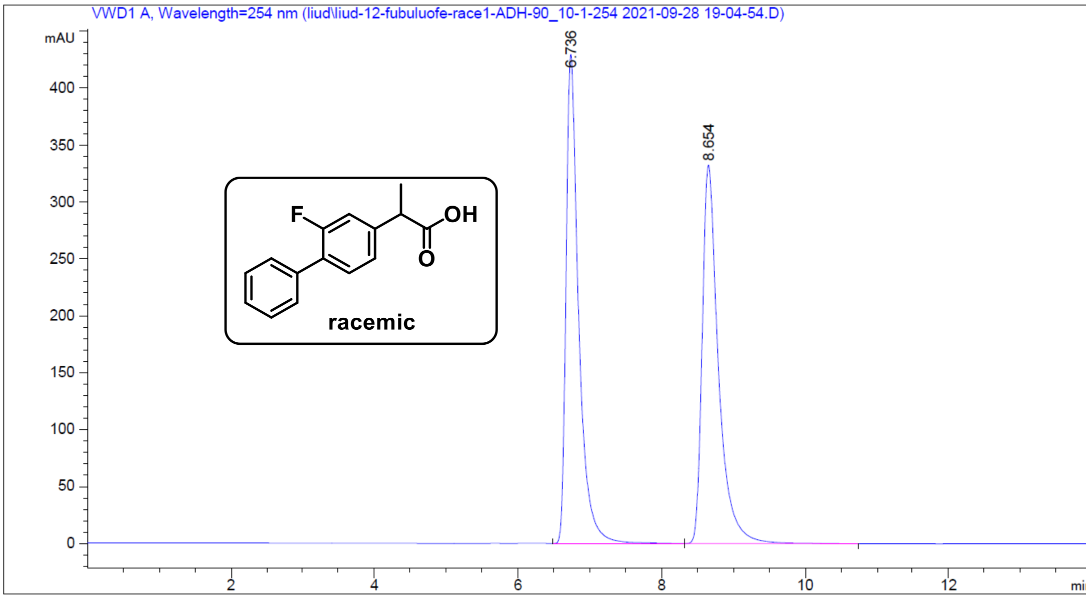

Peak	Ret Time [min]	Height [mAU]	Area %
1	6.736	430.26535	50.0177
2	8.654	332.82675	49.9823

Supplementary Figure 259. HPLC Chromatography of the Racemic (*R*)-2-(2-fluoro-[1,1'-biphenyl]-4-yl)propanoic acid

(Daicel Chiralpak AD-H Column, *n*-Hexane: *i*-PrOH = 90:10, flow rate 1.0 mL/min, T = 25 °C, λ = 254 nm)

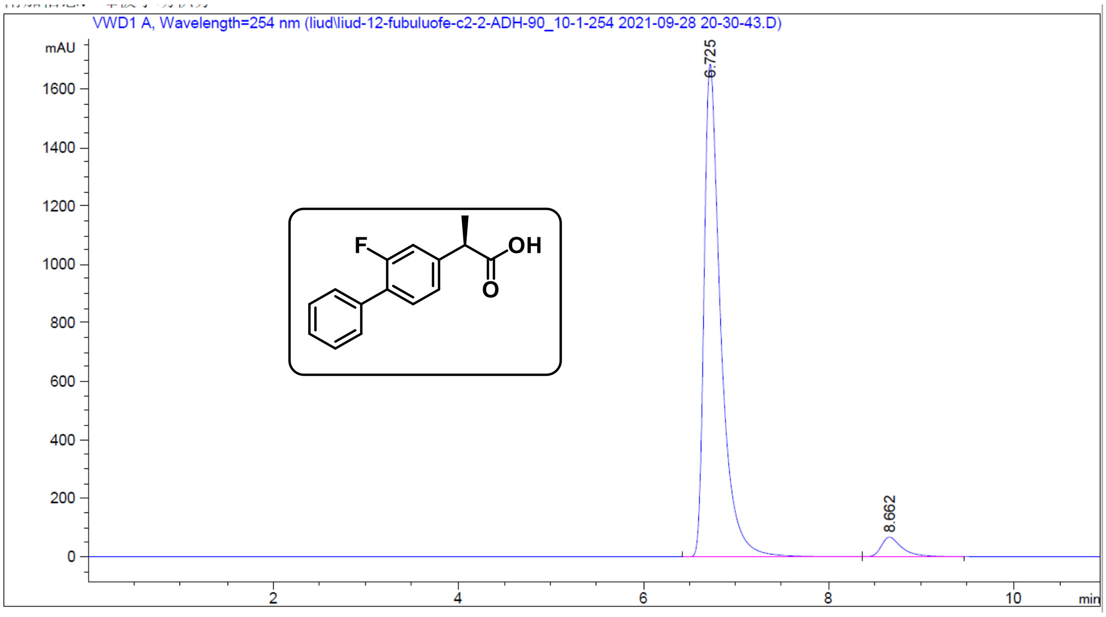

Peak	Ret Time [min]	Height [mAU]	Area %
1	6.725	1685.18445	95.2648

2	8.662	66.73416	4.7352

Supplementary: HPLC data for the Suboptimal substrates products

Supplementary Figure 260. HPLC Chromatography of the Racemic 2,3,3-trimethylbutan-2-yl (*E*)-2-methyl-4-phenylbut-3-enoate

(Daicel Chiralpak Cellulose-3 Column, MeCN: H_2O = 85:15, flow rate 0.7 mL/min, T = 25 °C, λ = 214 nm)

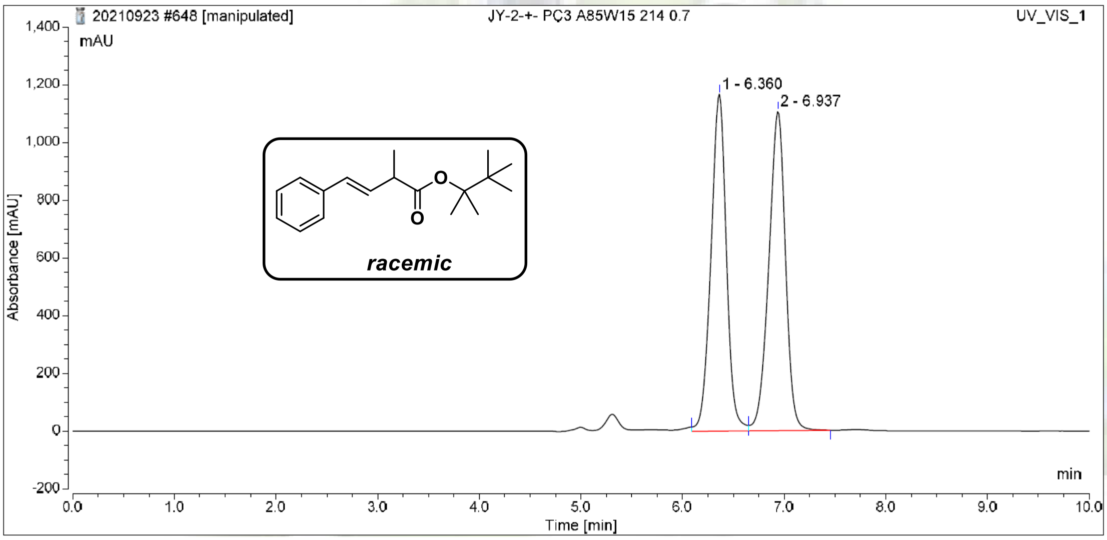

Integration Results								
No.	Retention Time min	Area mAU*min	Height mAU	Relative Area %	Resolution (EP)	Asymmetry (EP)	K'	Plates (EP)
n.a.	6.360	203.1607	1168.3172	49.043	2.06	0.97	n.a.	9136
n.a.	6.937	211.0918	1107.7794	50.957	n.a.	0.93	n.a.	8835
Total:		414.253	1401.998	100.000				

Supplementary Figure 261. HPLC Chromatography of the 2,3,3-trimethylbutan-2-yl (*R,E*)-2-methyl-4-phenylbut-3-enoate

(Daicel Chiralpak Cellulose-3 Column, MeCN: H_2O = 85:15, flow rate 0.7 mL/min, T = 25 °C, λ = 214 nm)

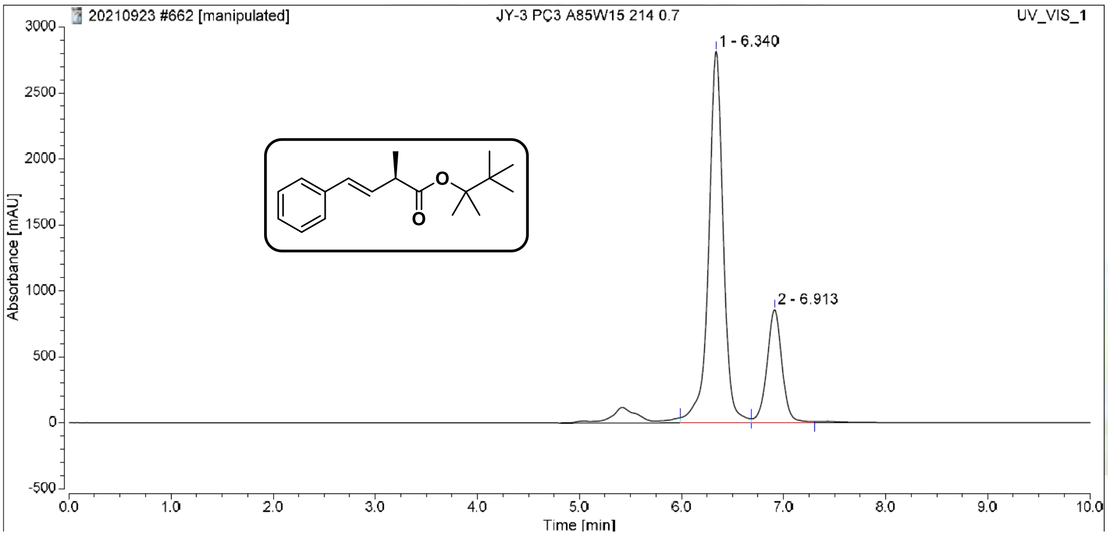

Integration Results								
No.	Retention Time min	Area mAU*min	Height mAU	Relative Area %	Resolution (EP)	Asymmetry (EP)	K'	Plates (EP)
n.a.	6.340	468.4721	2817.4503	76.028	2.30	0.90	n.a.	11046
n.a.	6.913	147.7128	860.5009	23.972	n.a.	0.97	n.a.	11517
**Total:**		**616.185**	**1401.998**	**100.000**				

Supplementary Figure 262. HPLC Chromatography of the Racemic 2,3,3-trimethylbutan-2-yl 2-(pyridin-3-yl)propanoate

(Daicel Chiralpak OD-H Column, *n*-Hexane: *i*-PrOH = 95:5, flow rate 1.0 mL/min, T = 25 °C, λ = 254 nm)

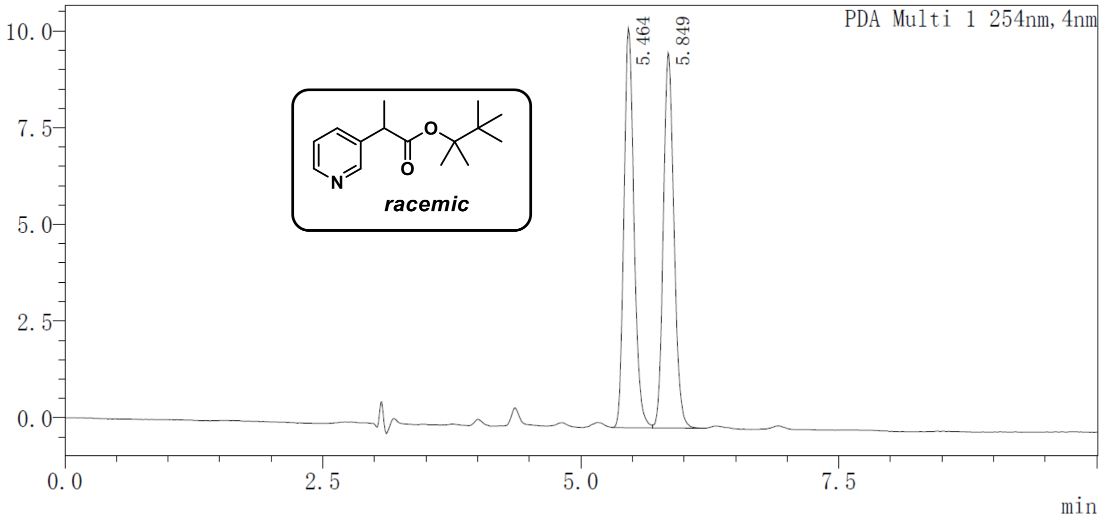

Peak	Ret Time [min]	Height [mAU]	Area %
1	5.464	10331	50.217
2	5.849	9705	49.783

Supplementary Figure 263. HPLC Chromatography of the 2,3,3-trimethylbutan-2-yl (*R*)-2-(pyridin-3-yl)propanoate

(Daicel Chiralpak OD-H Column, *n*-Hexane: *i*-PrOH = 95:5, flow rate 1.0 mL/min, T = 25 °C, λ = 254 nm)

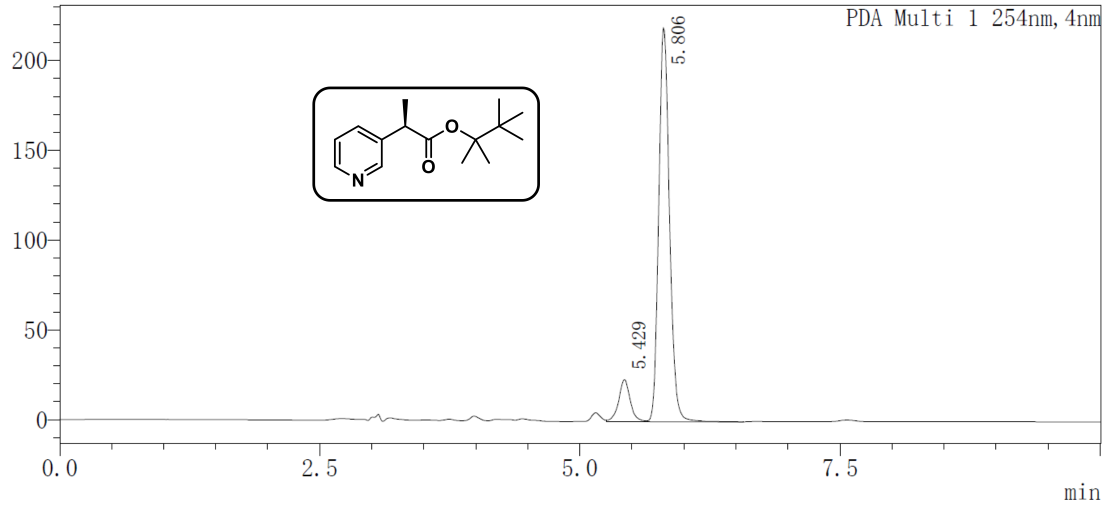

Peak	Ret Time [min]	Height [mAU]	Area %
1	5.429	23440	10.243
2	5.806	219559	89.757

Supplementary Figure 264. SFC Chromatography of the Racemic 2,3,3-trimethylbutan-2-yl 2-(1*H*-inden-2-yl)propanoate

SFC (Daicel Chiralpak IF-3 Column, 2% *i*-PrOH in supercritical CO_2, 2.5 mL/min, T = 25 °C, λ = 230 nm)

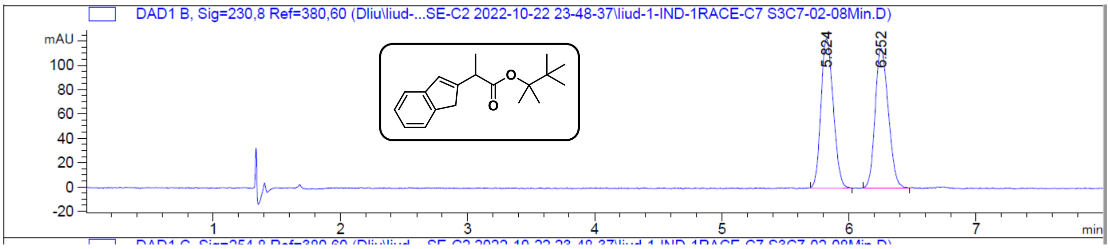

Signal 2: DAD1 B, Sig=230,8 Ref=380,60

Peak #	RetTime [min]	Type	Width [min]	Area [mAU*s]	Height [mAU]	Area %
1	5.824	BB	0.1034	825.38147	122.78715	49.9215
2	6.252	BB	0.1132	827.97888	114.78175	50.0785
Totals :				1653.36035	237.56889	

Supplementary Figure 265. SFC Chromatography of the 2,3,3-trimethylbutan-2-yl (*R*)-2-(1*H*-inden-2-yl)propanoate

SFC (Daicel Chiralpak IF-3 Column, 2% *i*-PrOH in supercritical CO_2, 2.5 mL/min, T = 25 °C, λ = 230 nm)

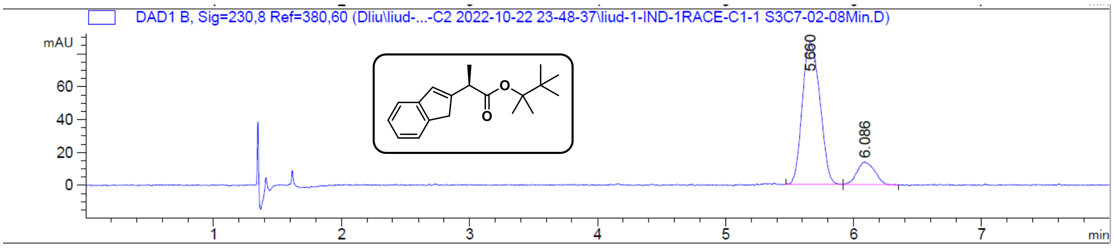

Signal 2: DAD1 B, Sig=230,8 Ref=380,60

Peak #	RetTime [min]	Type	Width [min]	Area [mAU*s]	Height [mAU]	Area %
1	5.660	BB	0.1365	824.23224	86.26016	86.3072
2	6.086	BB	0.1147	130.76608	13.78380	13.6928
Totals :				954.99832	100.04397	

Supplementary Figure 266. SFC Chromatography of the Racemic methyl 4-(1-(((1*R*,2*S*,5*R*)-2-isopropyl-5-methylcyclohexyl)oxy)-1-oxopropan-2-yl)benzoate

SFC (Daicel Chiralpak IF-3 Column, 2% *i*-PrOH in supercritical CO_2, 2.5 mL/min, T = 25 °C, λ = 230 nm)

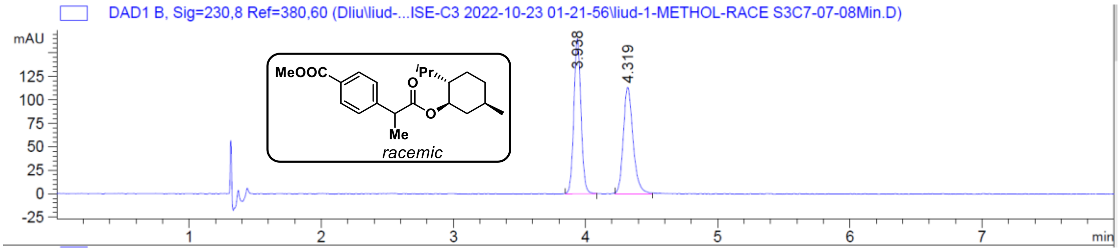

Signal 2: DAD1 B, Sig=230,8 Ref=380,60

Peak #	RetTime [min]	Type	Width [min]	Area [mAU*s]	Height [mAU]	Area %
1	3.938	BB	0.0577	619.15247	165.28320	51.6222
2	4.319	BB	0.0788	580.23987	113.29196	48.3778

Totals : 1199.39233 278.57516

Supplementary Figure 267. SFC Chromatography of the methyl 4-((*S*)-1-(((1*R*,2*S*,5*R*)-2-isopropyl-5-methylcyclohexyl)oxy)-1-oxopropan-2-yl)benzoate

SFC (Daicel Chiralpak IF-3 Column, 2% *i*-PrOH in supercritical CO_2, 2.5 mL/min, T = 25 °C, λ = 230 nm)

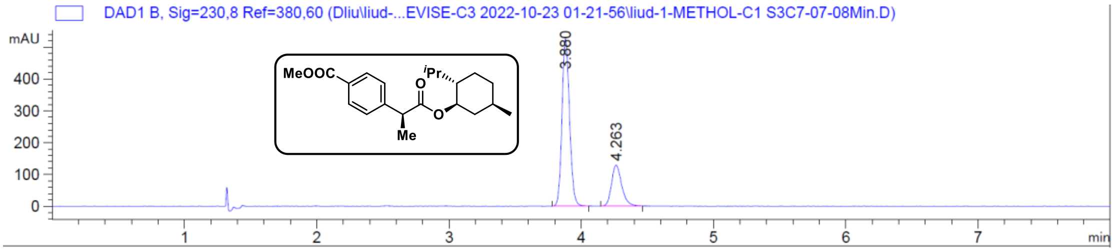

Signal 2: DAD1 B, Sig=230,8 Ref=380,60

Peak #	RetTime [min]	Type	Width [min]	Area [mAU*s]	Height [mAU]	Area %
1	3.880	BB	0.0614	2089.59009	525.83655	75.0373
2	4.263	BB	0.0828	695.14496	129.04030	24.9627

Totals : 2784.73505 654.87685

4. Supplementary References

[1]. Jin, M.; Adak, L.; Nakamura, M. *J. Am. Chem. Soc.* **2015**, *137*, 7128-7134.

[2]. Guan, H.; Zhang, Q.; Walsh, P. J.; Mao, J. *Angew. Chem. Int. Ed.*, **2020**, *59*(13), 5172-5177.

[3]. Xia, H.; Zhang, F.; Ye, T.; Wang, Y. *Angew. Chem. Int. Ed.*, **2018**, *57*, 11770-11775.
